# Analyzing Active Compounds in *Elateriospermum tapos* Yogurt for Maternal Obesity: A Network Pharmacology and Molecular Docking Study

**DOI:** 10.3390/foods12193575

**Published:** 2023-09-26

**Authors:** Ruth Naomi, Soo Huat Teoh, Hashim Embong, Santhra Segaran Balan, Fezah Othman, Kamalludin Mamat-Hamidi, Hasnah Bahari, Muhammad Dain Yazid

**Affiliations:** 1 Department of Human Anatomy, Faculty of Medicine and Health Sciences, Universiti Putra Malaysia, Serdang 43400, Malaysia; 2 Advanced Medical and Dental Institute, Universiti Sains Malaysia, Penang 13200, Malaysia; soohuat@usm.my; 3 Department of Emergency Medicine, Faculty of Medicine, Universiti Kebangsaan Malaysia, Kuala Lumpur 56000, Malaysia; hashimembong77@ukm.edu.my; 4 Department of Diagnostic and Allied Health Sciences, Faculty of Health and Life Sciences, Management and Science University, Shah Alam 40100, Malaysia; santhra@msu.edu.my; 5 Department of Biomedical Sciences, Faculty of Medicine and Health Sciences, Universiti Putra Malaysia, Serdang 43400, Malaysia; fezah@upm.edu.my; 6 Institute of Tropical Agriculture and Food Security, Universiti Putra Malaysia, Selangor 43400, Malaysia; mamath@upm.edu.my; 7 Department of Animal Science, Faculty of Agriculture, Universiti Putra Malaysia, UPM Serdang, Selangor 43400, Malaysia; 8 Centre for Tissue Engineering and Regenerative Medicine (CTERM), Universiti Kebangsaan Malaysia, Kuala Lumpur 56000, Malaysia

**Keywords:** *Elateriospermum tapos* yogurt, maternal programming, obesity, network pharmacology, molecular docking

## Abstract

Maternal obesity, characterized by an elevated body mass index (BMI) during pregnancy, is known to have adverse effects on the offspring. However, a recent study suggests that *Elateriospermum tapos* (*E. tapos*) yogurt may hold potential in mitigating excessive weight retention post-pregnancy. Thus, this study aims to employ network pharmacology to explore the pharmacological effects of the bioactive compounds present in *E. tapos* yogurt against maternal obesity. Initially, a screening process is conducted to identify the bioactive compounds in *E. tapos* yogurt, followed by the prediction of potential gene targets for these compounds using Swiss Target Prediction and the SuperPred databases. Maternal obesity-associated genes are sourced from the OMIM, DisGeNet, and GeneCards databases. The interaction between the identified compounds and maternal obesity genes is established via protein–protein interaction analysis, gene ontology examination, and KEGG pathway analysis. To validate the results, molecular docking studies are conducted using AutoDock Tools software. The findings reveal that out of the 64 compounds analyzed, three meet the screening criteria, resulting in a total of 380 potential gene targets. Among these targets, 240 are shared with maternal obesity-related genes. Further analysis demonstrates the favorable affinity of these active compounds with key targets, linking them to biological processes involving protein phosphorylation, inflammation, as well as the pathways related to lipid metabolism, atherosclerosis, and the other signaling pathways. In conclusion, this study provides valuable insights into the potential pharmacological effects of the bioactive compounds found in *E. tapos* yogurt against maternal obesity. These findings open avenues for further exploration and potential therapeutic interventions targeting maternal obesity.

## 1. Introduction

Maternal obesity refers to obesity during the childbearing age and is associated with serious adverse outcomes. Increased neonatal morbidity and mortality are some of the common notable consequences [1]. Increased risk of metabolic dysfunction, lipotoxicity, fetal overgrowth, and an impaired neurodevelopmental process in the F1 generation are some other common negative impacts of maternal obesity [1]. There is increasing evidence shows that maternal obesity contributes to the inheritance of obesity to the child, which means pregnant mothers can inherit their epigenome to their offspring by transferring the parent cell’s germline to their child. This eventually modifies the epigenome of the embryo stem, causing obesity development in their child sooner or later [2]. In maternal obesity, the production of pro-inflammatory factors such as tumor necrosis factor (TNF)-α, interleukin (IL)-6, and adipokine hormones may increase. Dysregulation in the release of inflammatory factors will enhance the oversupply of nutrients to the fetus, leading to fetus overgrowth [3] and thereby increasing the risk of developing metabolic diseases in their later life [4]. The prevalence of maternal obesity across the global are in alarming state. Globally, at least 10–30% of pregnant mothers are reported to be obese [5]. A cross-sectional study performed among various countries shows that the majority of pregnant mothers with obesity existed in low-income countries and increases with higher parity [6]. Malaysia has been classified as the fattest nation in the south-east Asia and one of the countries with the highest pregnant mothers. A survey conducted by the national health morbidity agency in the year of 2016 shows that the prevalence rate of maternal obesity in Malaysia is the highest among the advanced age between 45 to 49, followed by ethnic Malay and Indians, which is 69.2%, 16.8%, and 15.6%, respectively. According to the maternal morbidity confidential enquiries, they certified that the three most common complications of maternal obesity, such as pregnancy hypertension, obstetric embolism, and postpartum hemorrhage, are the main cause of maternal deaths in Malaysia up to the year 2011. The Malaysian National Obstetric Registry data shows that pregnant obese mothers have a high risk of having stillbirths, shoulder dystocia, macrosomia, and cesarean delivery [7].

The current available modern medicine for weight loss is not recommended during pregnancy. This is because the fetal development period is considered critical and any form of interventions for weight loss may carry potential adverse effects on both the obese maternal subject and the developing fetus. For instance, one of the most common weight loss medicine known as Phentermine taken during the first trimester of pregnancy resulted in structural anomalies such as increased birth weight and head circumference [8]. Similarly, orlistat is contraindicated during the gestation period as orlistat has absolute contraindications in the cases of chronic malabsorption syndrome and cholestasis [9]. Therefore, there is an urgency to develop complementary and alternative medications to curb maternal obesity. In these, a currently developed *Elateriospermum tapos* (*E. tapos*) yogurt has shown promising effect in maternal obesity [10] and the improvement of body composition in male and female offspring [11]. *E. tapos* yogurt has been found to possess a rich concentration of antioxidants and various other bioactive compounds. However, the composition in *E. tapos* yogurt is considered to be so intricate that it makes it difficult to fully explain its underlying mechanism through the literature research. Hence, it is essential to direct attention towards exploring the potential system-level mechanisms through which *E. tapos* yogurt can be effective in managing maternal obesity. 

**Figure 1:**
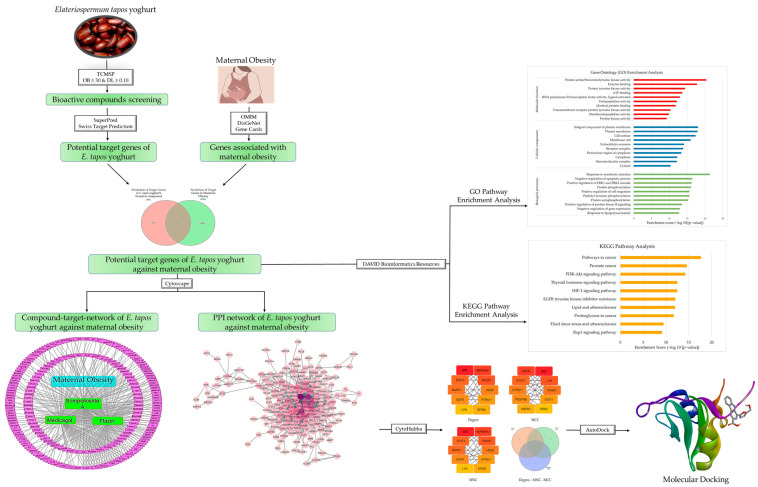
The flow of the research process.

Network pharmacology, as an emerging approach, leverages the advancements in bioinformatics and the related databases. It has become a widely utilized tool in drug research for uncovering the intricate interrelationships between drugs, their targets, the pathways, and the associated diseases [12]. By integrating network pharmacology and molecular docking, a more comprehensive understanding of the molecular mechanisms of *E. tapos* yogurt can be obtained. Therefore, this study employed network pharmacology to investigate the molecular targets and mechanisms by which *E. tapos* yogurt alleviates maternal obesity. Initially, network pharmacology was employed to predict the target proteins and pathways associated with the therapeutic effects of *E. tapos* yogurt in managing maternal obesity. Next, molecular docking techniques were used to verify the potential targets of the selected bioactive compounds of *E. tapos* yogurt against maternal obesity. The flow of the research process is summarized in Figure 1.

## 2. Materials and Methods

### 2.1. Screening of Bioactive Compounds of E. tapos Yogurt

The bioactive compounds of *E. tapos* yogurt were obtained via our preliminary study published by Naomi et al., 2023 [13,14] and attached in the Supplementary Files (Supplementary Table S1). All the listed bioactive compounds of *E. tapos* yogurt were then screened for absorption, distribution, metabolism, and excretion (ADME) properties. In order to assess these parameters, a computational integrative ADME model was employed, utilizing the Traditional Chinese Medicine Systems Pharmacology Database. The database (https://tcmsp-e.com/load_intro.php?id=43) was assessed on 1 June 2023 [15]. For further analysis, the compounds meeting the criteria set by the TCMSP database, specifically with an oral bioavailability of ≥30 and a drug likeness of ≥0.18, were selected [16]. 

### 2.2. Prediction of Target Genes in E. tapos Yogurt’s Bioactive Compounds

The Swiss Target Prediction database (http://www.swisstargetprediction.ch/, accessed on 4 June 2023) [17] (Supplementary Table S2) and the SuperPred database (https://prediction.charite.de/index.php, accessed on 4 June 2023) [18] (Supplementary Table S3) were used to identify the target genes of the selected bioactive compounds present in *E. tapos* yogurt. To ensure the applicability to human biology, the analysis was limited to the target genes found in homo sapiens, and any duplicated entries were removed, resulting in a final list.

### 2.3. Targets of Disease-Related Compounds

The related genes were collected from the GeneCards (http://www.genecards.org/ accessed on 8 June 2023) [19] (Supplementary Table S4), OMIM (https://www.omim.org/accessed on 9 June 2023) [20] (Supplementary Table S5), and DisGeNet (https://www.disgenet.org/ accessed on 9 June 2023) [21] databases (Supplementary Table S6). The target protein names were converted to their official gene names using the UniProt database (https://www.sparql.uniprot.org/accessed on 9 June 2023) [22]. The inquiry focused on the keyword “maternal obesity” and any redundant genes were excluded to ensure a comprehensive and non-duplicative list. 

### 2.4. Construction of Venn Diagrams

The overlapping potential target genes of *E. tapos* yogurt against maternal obesity were analyzed using InteractiVenn (http://www.interactivenn.net/, accessed on 16 June 2023) [23]. The results of the analysis were visualized in a Venn diagram, illustrating the common potential target genes shared between the selected bioactive compounds of *E. tapos* yogurt against maternal obesity. 

### 2.5. Construction of Compound–Pathway–Target Network

The Cytoscape software version 3.9.1 (https://cytoscape.org/, accessed on 16 June 2023) [24] was used to construct a compound–pathway–target network to visualize the interactions between the selected bioactive compounds of *E. tapos* yogurt against maternal obesity in a more effective manner. The data on the active components of *E. tapos* yogurt and their respective target information were integrated into Cytoscape. The network nodes were represented by the common target genes of both *E. tapos* yogurt’s bioactive compounds and maternal obesity, and the connections between these nodes were depicted using connecting lines. This process resulted in the formation of a compound–pathway–target network.

### 2.6. Construction of Protein–Protein Interaction (PPI) Network 

The protein–protein interaction network analysis was conducted to examine the overlap between the target genes of bioactive compounds in *E. tapos* yogurt against maternal obesity. This analysis utilized the STRING database version 11.5 (https://string-db.org/, accessed on 16 June 2023) [25]. Only proteins within homo sapiens were considered, and a confidence score of at least 0.900 was applied as a threshold for network visualization. The resulting network was visualized using the Cytoscape platform, integrating the data obtained from the STRING database. To identify the most significant genes in the network, the CytoHubba plugin was employed [26]. Three algorithms, namely the maximal clique centrality (MCC), maximum neighborhood component (MNC), and degree, were utilized to rank the genes. The top 10 hub genes were determined based on their scores by each algorithm. The results of the three algorithms were then compared to find the intersection, yielding the final set of hub genes. These hub genes represent the potential key targets within the network.

### 2.7. Construction of Gene Ontology and Pathway Enrichment 

To analyze the functional enrichment of genes, a gene ontology (GO) and Kyoto Encyclopedia of Genes and Genomes (KEGG) pathway enrichment analysis was performed using the online software David version 2021 (https://david.ncifcrf.gov/home.jsp, accessed on 16 June 2023) [27]. The analysis focused on GO biological processes, GO molecular functions, GO cellular components, and the KEGG pathways [28,29,30]. Statistical significance was determined by considering only *p* < 0.05 as statistically significant. 

### 2.8. Molecular Docking 

The study used a molecular docking approach to examine the interactions between the specific bioactive compounds found in *E. tapos* yogurt and the identified hub genes to understand the binding modes and affinities between the receptors and ligands by predicting their binding interactions. The crystal structures of eight hub genes (PIK3R1, HRAS, MAPK1, STAT3, EGFR, LYN, PTPN11, and SRC) were obtained from the RCSB Protein Data Bank (https://www.rcsb.org/, accessed on 20 June 2023) [30], while the 3D structures of the selected bioactive compounds (Flazin, Medicagol, and Scropolioside A) from *E. tapos* yogurt were retrieved from the PubChem database (https://pubchem.ncbi.nlm.nih.gov/, accessed on 20 June 2023) [31]. The compounds served as ligands, and the hub genes acted as receptors. The receptor structures were prepared by removing the water molecules and existing ligands using Biovia Discovery Studio 2021. The binding conformation between the ligands and receptors was predicted using AutoDockTools Version 4.2 (http://autodock.scripps.edu/, accessed on 21 June 2023) [32], and the resulting binding energy, a measure of the molecular docking outcome, was used to evaluate the potential of the ligand–receptor binding (typically ≤ −5 kcal/mol). Finally, the Discovery Studio Visualizer 2021 was used to visualize the molecular interactions between the proteins and ligands. The summarized Table 1 below presents the selection of PDB IDs refined for the homo sapiens species. 

## 3. Results

### 3.1. Selection of Potential Bioactive Compounds 

The preliminary study has identified that there are 64 bioactive compounds in *E. tapos* yogurt (Supplementary Table S1) [13,14]. These are the three selected compounds Scropolioside A, Flazin, and Medicagol which have been identified as potential bioactive compounds that fulfill the necessary criteria for drug screening (ADME), as shown in Table 2. 

### 3.2. Target Gene Prediction of E. tapos Yogurt against Maternal Obesity 

**Figure 2:**
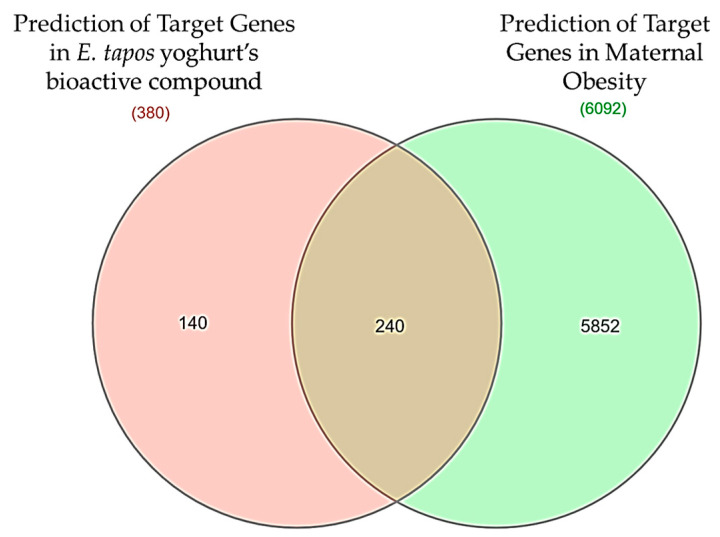
Venn diagram of potential targets for *E. tapos* yogurt and genes associated with maternal obesity.

By utilizing the Swiss Target Prediction and SuperPred databases (Supplementary Table S2), a total of 380 target genes associated with the three bioactive compounds in *E. tapos* yogurt were identified. Furthermore, an extensive search of the three databases, namely OMIM, DisGeNet, and GeneCards, resulted in the discovery of 6092 target genes that are linked to maternal obesity (Supplementary Table S3). Figure 2 shows the target of *E. tapos* yogurt against maternal obesity represented by a Venn diagram. The 240 disease-related compound targets were obtained via a Venn diagram.

### 3.3. Construction of Compound–Pathway–Target Network

**Figure 3:**
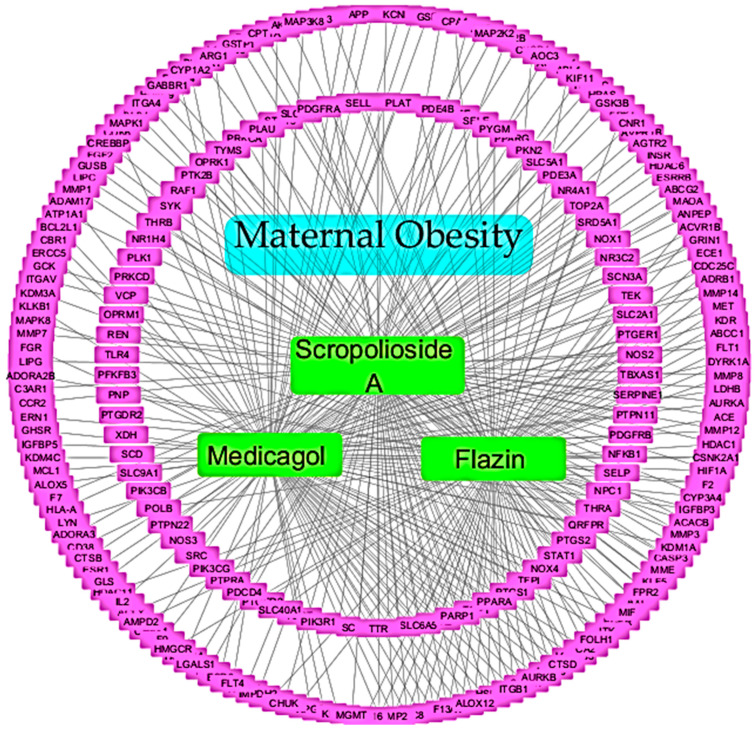
The compound–target network of the selected bioactive compounds in *E. tapos* yogurt compounds against maternal obesity.

The compound–target network, as shown in Figure 3, was created using Cytoscape software to represent the interaction between the selected bioactive compounds in *E. tapos* yogurt and its potential targets for maternal obesity. This network visually illustrates the relationship between the three bioactive compounds (Scropolioside A, Flazin, and Medicagol) found in *E. tapos* yogurt and their corresponding targets, highlighting their potential role in managing maternal obesity. The network consists of 244 nodes and 348 edges.

### 3.4. Construction of PPI Network 

**Figure 4:**
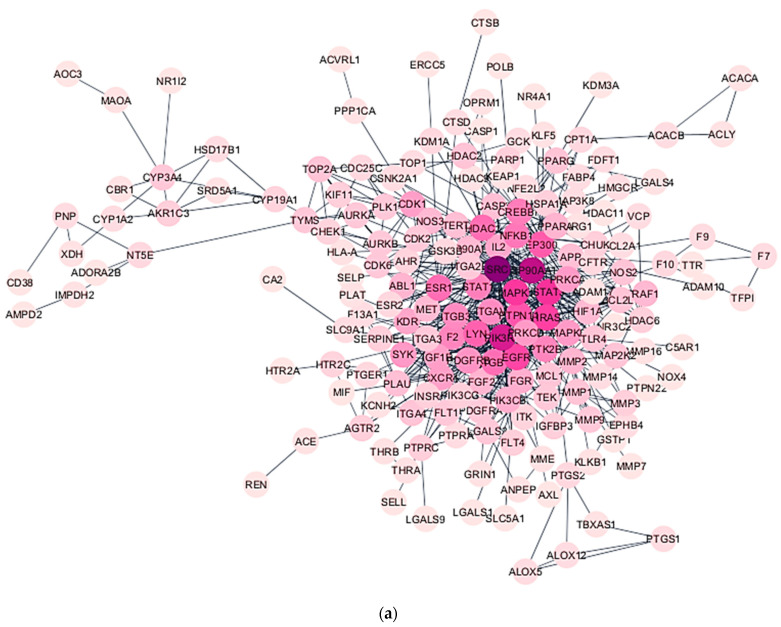

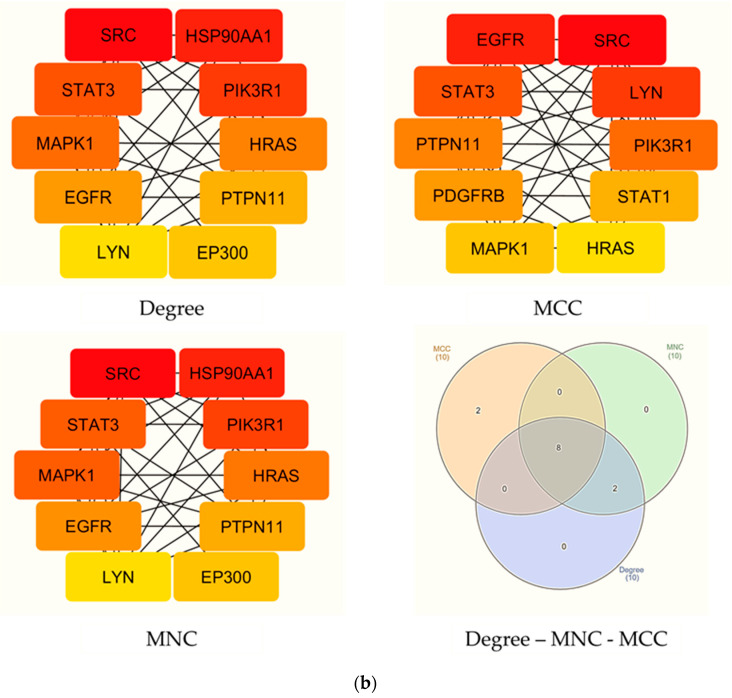
(**a**) PPI network of the selected bioactive compounds in *E. tapos* yogurt against maternal obesity. Node sizes correspond to their degrees, with larger nodes representing higher degrees. Additionally, the importance of nodes is depicted using darker colors, where darker shades indicate more significant nodes. (**b**) The hub gene networks of the selected bioactive compounds in *E. tapos* yogurt’s bioactive compounds in managing maternal obesity were identified using three algorithms: MCC, MNC, and node degree. The top ten hub genes were selected based on these algorithms. The intersections of the three algorithms were visualized using a Venn diagram, revealing eight hub genes that were common across all three algorithms. The importance of nodes is depicted using darker colors, where darker shades indicate more significant nodes.

Figure 4a shows the PPI network of the selected bioactive compounds in *E. tapos* yogurt compounds against maternal obesity. For this, the 240 compound targets associated with diseases were imported into the String database to construct the PPI network. Using the CytoHubba plug-in of Cytoscape 3.9.1, the PPI network was analyzed to identify the top 10 hub genes. This selection was based on three criteria: maximum neighborhood component (MNC), maximal clique centrality (MCC), and degree. The CytoHubba plug-in facilitated the mapping of these hub genes, allowing for their visual representation within the network. To identify similar genes, an interactive Venn analysis was conducted. Figure 4b displays the results, revealing the top-ranked genes as PIK3R1, MAPK1, LYN, SRC, EGFR, PTPN11, STAT3, and HRAS. 

### 3.5. Construction of GO and KEGG Pathway 

**Figure 5:**
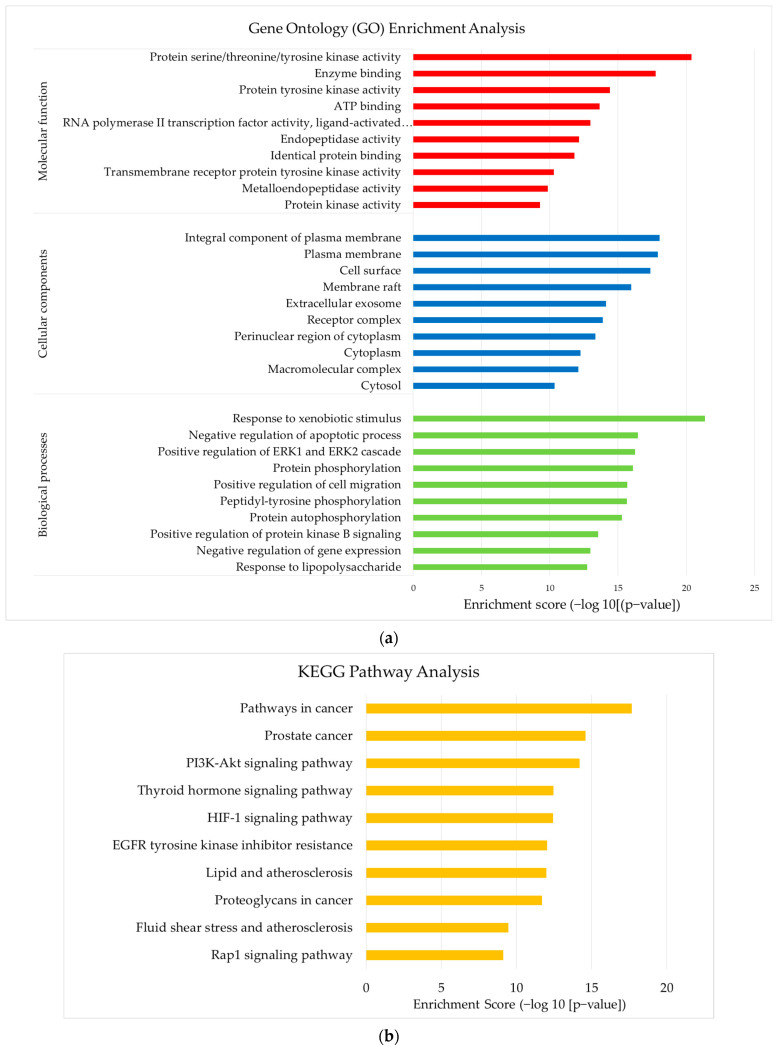
(**a**) The top ten targets of *E. tapos* yogurt’s bioactive compounds against the GO parameters in maternal obesity. (**b**) The top ten targets of *E. tapos* yogurt’s bioactive compounds against the KEGG pathway analysis in maternal obesity.

**Figure 6:**
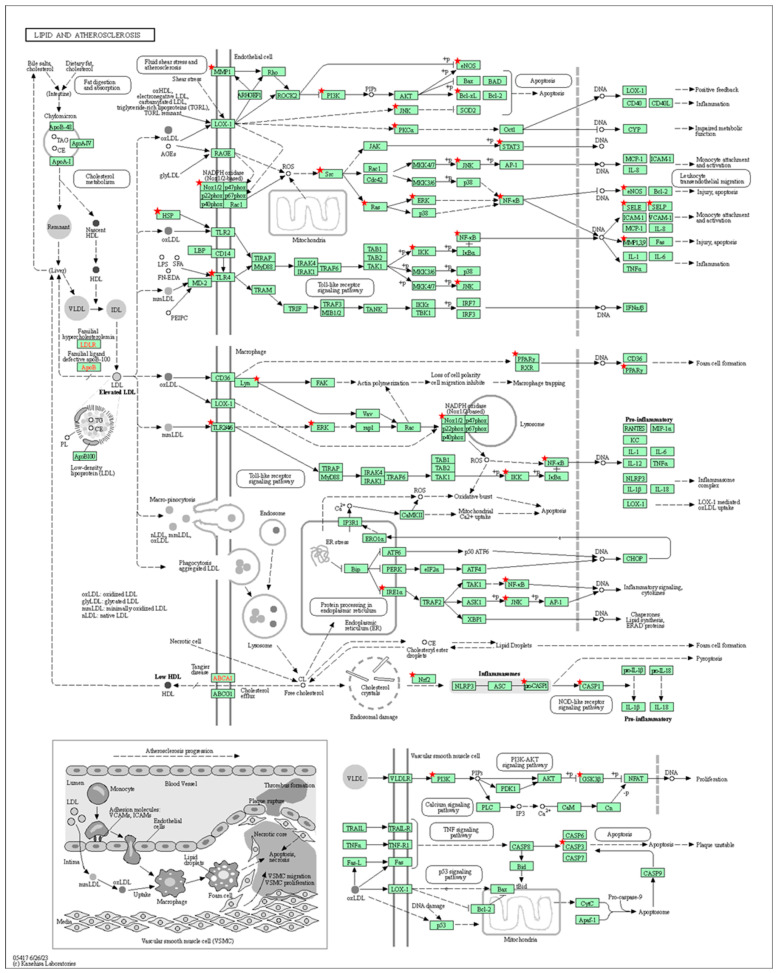
The potential targets and mechanism of bioactive compounds in *E. tapos* yogurt against maternal obesity. Red stars indicate the potential target genes of bioactive compounds in *E. tapos* yogurt against maternal obesity. +p indicates positive feedback.

Figure 5a,b shows the results of the GO and KEGG pathway analyses. In Figure 5a, the top targets of BP for *E. tapos* yogurt are focused on the response to lipopolysaccharide, the negative regulation of gene expression, and the positive regulation of protein kinase B signaling. Regarding CC, the focus is primarily on the cytosol, macromolecular complex, and cytoplasm. The MF analysis reveals a focus on protein kinase activity, metalloendopeptidase activity, and transmembrane receptor protein tyrosine kinase activity. As shown in Figure 5b, the targets of *E. tapos* yogurt’s bioactive compounds against maternal obesity are primarily associated with the fluid shear stress and atherosclerosis, lipid and atherosclerosis, and the PI3K-Akt signaling pathway. Figure 6 summarizes the potential targets and mechanism of bioactive compounds in *E. tapos* yogurt against maternal obesity.

### 3.6. Molecular Docking

**Figure 7:**
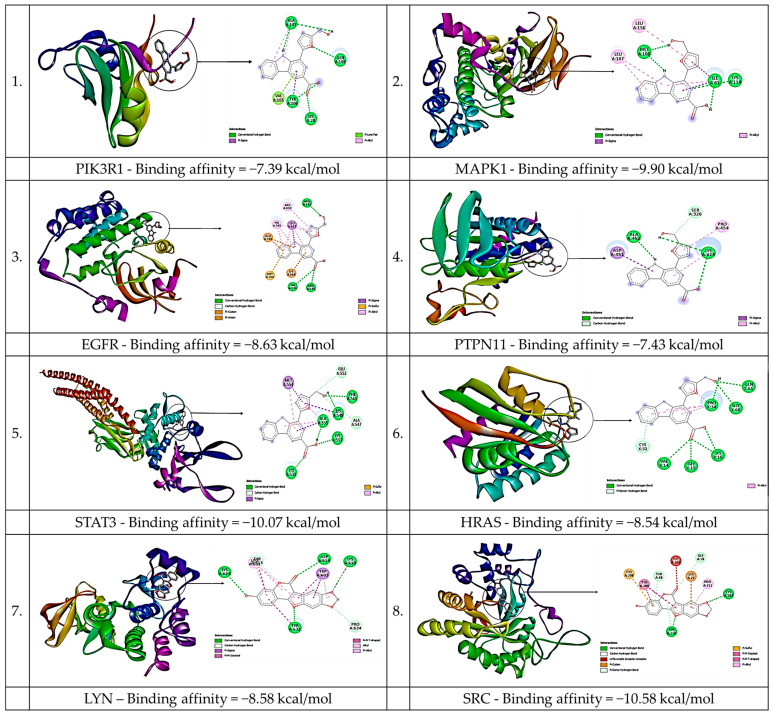
Molecular docking results of the lowest binding energy in each target with the selected bioactive compound of *E. tapos* yogurt.

The results presented in Figure 7 show the outcomes of molecular docking analyses carried out between specific bioactive compounds extracted from *E. tapos* yogurt and the most significant genes identified using three algorithms (MCC, MNC, and degree). Table 3, on the other hand, showcases the binding energy against the aforementioned prominent genes. A total of 24 docking results were obtained, and among them, 18 exhibited binding energies lower than −5.0 kcal/mol, indicating a robust binding interaction between the compounds and the proteins. As a result, Flazin and Medicagol emerge as crucial elements in alleviating maternal obesity. This significance arises from their high affinity to each target, as evidenced by their binding energies below −5.0 kcal/mol.

## 4. Discussion

Maternal obesity, a transgenerational condition, impacts the fetal epigenome modifications such as histone modification, DNA methylation, and microRNAs [4], and it is one of the prime causes for childhood obesity. This is because maternal obesity induces changes in the prenatal environment due to placental overnutrition, causing excessive levels of inflammatory cytokines and varying levels of metabolic hormones to reach the growing fetus [33], thereby affecting their growth. Therefore, excessive gestational weight gain and maternal obesity have been found to be positively correlated with the high body weight of the offspring. This association is particularly prominent among male offspring, as male fetal growth is characterized by rapid development and heightened susceptibility to prenatal insults. Consequently, male offspring are more likely to encounter a greater extent of adverse effects in utero compared to their female counterparts [34]. Considering the potential side effects associated with modern medicine, researchers led by Naomi et al., (2023) have developed a novel plant extract (*E. tapos*)-integrated yogurt that demonstrates transgenerational effects specifically targeting maternal obesity [10]. Despite its efficacy, the precise molecular mechanism underlying this effect remains largely unexplored. As a result, the present study has been designed with the aim of addressing this fundamental question. Via the analysis of protein–protein interaction networks, the study identifies several key targets of *E. tapos* yogurt in maternal obesity, including PIK3R1, MAPK1, LYN, SRC, EGFR, PTPN11, STAT3, and HRAS. These targets are involved in various pathways that play a crucial role in regulating lipid metabolism. 

In this study, the influence of *E. tapos* yogurt on a specific KEGG pathway related to the lipid- and atherosclerosis-signaling mechanism was investigated, highlighting its role as a primary target. It was observed that epigenetic modifications resulting from obese dams could elevate the risk of childhood obesity, potentially leading to increased fat deposition [35]. Common conditions associated with increased levels of fat deposition include microvascular disease, ischemic events, and atherosclerosis [36]. As a consequence, the obese dams tend to inherit the obese genes to their offspring [37]. However, as in this study, the focus of the selected bioactive compounds of *E. tapos* yogurt lies on the modulation of cholesterol homeostasis, lipid signaling mediators, lipid rafts, and membrane fluidity through the targeting of proteins involved in lipid metabolism and atherosclerosis. These factors play a crucial role in managing maternal obesity. For instance, synaptic activity is influenced by cholesterol in neurons, and glial cells support neuronal metabolism by transferring the substances and molecules that regulate their functions. The transfer of these molecules may rely on cholesterol (e.g., miRNAs) or occur independently (e.g., 24-OHC), thereby ensuring sufficient cholesterol for optimal neuronal function, specifically in terms of cholesterol homeostasis [38].

Aside from this, as shown in the KEGG pathway targets, the bioactive compounds in *E. tapos* yogurt’s target signaling pathways, such as PI3K-Akt, hypoxia-inducible factor (HIF-1), and EGFR, work in managing maternal obesity. The PI3K signaling pathway is crucial in regulating adipocyte metabolism, specifically in the insulin-dependent control of glucose and lipid metabolism. Furthermore, it plays a role in recruiting neutrophils and macrophages in the context of regulating inflammation induced by obesity, and it also contributes to the central nervous system’s control of food intake and energy expenditure [39]. Alike, elevated levels of HIF-1 are frequently observed in adipose tissue during obesity, contributing to the recruitment of M1 macrophages responsible for driving inflammation associated with obesity [40]. The stimulation of the EGFR signaling pathway is strongly linked to obesity and insulin resistance. This occurs because a HFD intake can lead to an upregulation of EGFR expression and its ligand and amphiregulin in adipose tissue macrophages. This, in turn, triggers the proliferation of monocytes and their subsequent infiltration into adipose tissue, ultimately contributing to obesity [41]. 

The study’s findings were reinforced by conducting molecular docking, providing additional evidence that flazin firmly attaches to the active pocket of the key target with a binding energy below −5.0 kcal/mol. This substantiates the hypothesis that these specific compounds play a crucial role in the pathway responsible for mitigating maternal obesity. Flazin, a β-carboline-derived alkaloid [42], is renowned for its capacity in lipid regulation [43] and is a promising agent as an Nrf2 pathway activator [42]. 

In addition, the study’s molecular docking data reveal the binding activity of all three selected compounds from *E. tapos* yogurt to SRC, with STAT3 displaying the lowest binding score. This pattern is evident in flazin, medicagol, and scropolioside. As the binding energy decreases, the stability of the ligand–receptor binding conformation improves, indicating a greater potential for interaction. These findings strongly suggest that SRC and STAT3 could be primary targets for *E. tapos* yogurt in managing maternal obesity. 

## 5. Uses of Selected Bioactive Compounds

Flazin, medicagol, and scropolioside A are all natural compounds found in various plant species. They have biological functions. Table 4 shows an overview of each of these compounds:

## 6. Conclusions

In conclusion, this study highlights the potential mechanism of bioactive compounds in *E. tapos* yogurt against maternal obesity via the network pharmacology and molecular docking. The results from the computational analysis show that flazin, medicagol, and Scropolioside A might have a crucial role in managing maternal obesity via their effects on key biological targets, including PIK3R1, MAPK1, LYN, SRC, EGFR, PTPN11, STAT3, and HRAS. The molecular docking score further proves that the selected bioactive compounds from *E. tapos* yogurt could interact effectively with these targets. However, further investigation, such as experimental verification, is required to prove the underlying mechanism as hypothesized via the computational analysis in this study.

## 7. Advantages and Constraints of Ligands with Weak-Binding Affinity

In this study, several compounds exhibit weak binding affinity, measured at <10 kcal/mol, as shown in Table 3. There are several merits and limitations associated with weak-binding ligands. Weak-binding ligands offer advantages in the study of dynamic interactions between molecules. Their low-binding energies enable rapid association and dissociation, making them suitable for probing the transient interactions in biological systems [46]. Additionally, weak-binding ligands serve as a foundation for optimization, allowing for chemical modifications to enhance the binding affinity, selectivity, and pharmacokinetic properties [47]. They can also be employed to map the binding sites on target proteins or receptors. Using a weak-binding ligand as a probe enables the identification of potential binding pockets and provides insights into the structural and functional aspects of the target [48]. On the flip side, weak-binding affinity does possess some limitations. Weak-binding ligands may lack specificity, as they can interact with multiple targets. This lack of selectivity can hinder their use in drug development when highly specific interactions are required and they might not elicit the desired biological response due to their low-binding affinity [49]. Nonetheless, experimental techniques used to study weak-binding interactions must be highly sensitive and precise, which can be technically demanding and costly.

## 8. Merits and Limitations of Similar Computation Methods

This study uses network pharmacology to explore the potential pharmacological effects of bioactive compounds in *E. tapos* yogurt against maternal obesity. While this approach has several merits, it also has limitations. Additionally, there are related in silico techniques, such as QSAR (Quantitative Structure–Activity Relationship) studies and pharmacophore studies that have been used in similar research areas. The utilization of network pharmacology provides comprehensive analysis of the interactions between bioactive compounds and their potential gene targets, which provide a holistic view of the potential mechanisms of action. Since, the network pharmacology integrates data from various sources, such as the compound databases, gene databases, and pathway databases, it enables a multidimensional analysis. In addition, network pharmacology can identify the key targets and pathways associated with a specific condition, in this case, maternal obesity, which can guide further experimental research, and by predicting potential gene targets and pathways, we can prioritize which bioactive compounds are most promising for further investigation [50]. In addition, the usage of molecular docking studies provides a way to validate the potential interactions between compounds and target proteins, adding a level of confidence to the predictions [51]. Despite numerous beneficial effects, network pharmacology does have some limitations such as the accuracy of predictions in network pharmacology heavily relies on the quality and reliability of the data used from the various databases. Inaccurate or incomplete data can lead to unreliable results. While network pharmacology can provide valuable insights and hypotheses, experimental validation is necessary to confirm the actual pharmacological effects of bioactive compounds.

## Figures and Tables

**Table 1:** Selection of PDB IDs refined for homo sapiens species for the identified vibrant genes.

Hub Gene	PDB ID
PIK3R1	1BFJ
MAPK1	2Y9Q
LYN	3SXS
SRC	3BCJ
EGFR	3POZ
PTPN11	3O5X
STAT3	6NJS
HRAS	2CE2

**Table 2:** The pharmacokinetic properties of the bioactive compounds found in *E. tapos* yogurt based on TCMSP database.

	Compound	Formula	Oral Bioavailability (OB) ≥ 30%	Drug Likeness (DL) ≥ 0.18
1	Scropolioside A	C_35_H_44_O_18_	38.63	0.77
2	Flazin	C_17_H_12_N_2_O_4_	94.28	0.39
3	Medicagol	C_16_H_8_O_6_	57.49	0.60

**Table 3:** Docking scores of the selected bioactive compounds of *E. tapos* yogurt against the targeted genes in maternal obesity.

	Binding Energy							
Compounds	PIK3R1	MAPK1	LYN	SRC	EGFR	PTPN11	STAT3	HRAS
Flazin	−7.39	−9.90	−7.78	−10.4	−8.63	−7.43	−10.1	−8.54
Medicagol	−6.86	−7.85	−8.58	−10.6	−8.07	−7.32	−7.15	−7.87
Scropolioside A	−3.54	−5.05	−4.51	−8.70	−5.59	−4.37	−3.22	−4.43

**Table 4:** Functions of flazin, Medicagol, and Scropolioside A.

Compound	Uses
Flazin [43]	-Lipid Droplet Regulator -Reduce triglyceride levels -Upregulates mRNA expression in adipose triglyceride lipase -Suppressive effect on lipogenesis
Medicagol [44]	-Induce apoptosis -Modulate the activity of various enzymes and transcription factors involved in cell signaling pathways
Scropolioside A [45]	-Anti-inflammatory

## Data Availability

The datasets generated during and/or analyzed during the current study are available from the corresponding author on reasonable request.

## Supplementary Materials

The following supporting information can be downloaded at: https://www.mdpi.com/article/10.3390/foods12193575/s1, Table S1: Bioactive compounds and the ADME properties of *E. tapos* yogurt; Table S2: Target gene of *E. tapos* yogurt from Swiss Target Prediction database; Table S3: Target gene of *E. tapos* yogurt from SuperPred database; Table S4: Targets of genes associated with maternal obesity from GeneCards; Table S5: Targets of genes associated with maternal obesity from OMIM; and Table S6: Targets of genes associated with maternal obesity from disGeNet.
