# Supplementary material for: Analyzing Active Compounds in Elateriospermum tapos Yogurt for Maternal Obesity: A Network Pharmacology and Molecular Docking Study
Source: Foods. 2023 Sep 26;12(19):3575. doi: 10.3390/foods12193575 (PMC10572448; doi:10.3390/foods12193575)
Supplement: Supplementary file 1 [file foods-12-03575-s001.zip › foods-2617982-supplementary.pdf]

Supplementary table S1. Bioactive compounds of *E. tapos* yoghurt (Naomi et al., 2023) and the oral bioavailability and drug likeness based on TCMSP database.

No	Molecule	Formula	Oral bioavailability	Drug likeness
1	1,2,3,4,6-Penta-O-galloyl-β-D-glucopyranoside	$C_{41}H_{32}O_{26}$	Unidentified	Unidentified
2	1,2,3,6-Tetra-O-galloyl-β-D-glucopyranoside	$C_{34}H_{28}O_{22}$	Unidentified	Unidentified
3	α-D-(6-O-4-Methyl-3,5-dimethoxycinnamoyl)-glucopyranosyl(1→2)-β-D-(3-O-sinapoyl)-fructofuranose	$C_{35}H_{44}O_{18}$	Unidentified	Unidentified
4	Scropolioside A	$C_{35}H_{44}O_{18}$	38.63	0.77
5	Loroglossin	$C_{34}H_{46}O_{18}$	7.33	0.44
6	Yadanzioside A	$C_{32}H_{44}O_{16}$	7.63	0.32
7	1,3,5-O-Tricaffeoyl-quinic acid	$C_{34}H_{30}O_{15}$	Unidentified	Unidentified
8	Picrasinoside H	$C_{30}H_{44}O_{13}$	Unidentified	Unidentified
9	Kaempferol 3-O-α-L-rhamnopyranosyl-(1→2)-β-D-glucuronopyranoside	$C_{27}H_{28}O_{16}$	Unidentified	Unidentified
10	5′-Methoxy-bilobetin	$C_{32}H_{22}O_{11}$	22.38	0.60
11	3',4',7-Tribenzylepisappanol	$C_{37}H_{34}O_{6}$	Unidentified	Unidentified
12	Picrasinoside B	$C_{28}H_{40}O_{11}$	Unidentified	Unidentified

13	Ligustrosidic acid	$C_{26}H_{32}O_{13}$	Unidentified	Unidentified
14	Loganic acid-6'-O-β-D-glucoside	$C_{22}H_{34}O_{15}$	4.92	0.40
15	Ephedradine C	$C_{30}H_{40}N_4O_5$	Unidentified	Unidentified
16	Rehmannioside A	$C_{21}H_{32}O_{15}$	25.95	0.87
17	Ephedradine B	$C_{29}H_{38}N_4O_5$	Unidentified	Unidentified
18	Indigoticoside A	$C_{26}H_{34}O_{11}$	Unidentified	Unidentified
19	Mannotriose	$C_{18}H_{32}O_{16}$	Unidentified	Unidentified
20	19β-Glucosyl-14-deoxyandrographoside	$C_{26}H_{40}O_9$	Unidentified	Unidentified
21	Ephedradine A	$C_{28}H_{36}N_4O_4$	Unidentified	Unidentified
22	Acacetin-7-O-(6"-O-acetyl)-β-D-glucopyranoside	$C_{24}H_{24}O_{11}$	8.47	0.70
23	Segetalin B	$C_{24}H_{32}N_6O_5$	4.10	0.56
24	Bruceine B	$C_{23}H_{28}O_{11}$	16.46	0.81
25	Forsythoside D	$C_{20}H_{30}O_{13}$	5.26	0.67
26	2-Methoxy-4-acetylphenol 1-O-α-L-rhamnopyranosyl-(1''→6')-β-D-glucopyranoside	$C_{21}H_{30}O_{12}$	Unidentified	Unidentified

27	2,3,5,4'-Tetrahydroxystilbene-2-O-(6''-O-acetyl)-β-D-glucopyranoside	$C_{22}H_{24}O_{10}$	2.99	0.55
28	(Z)-(1S,5R)-β-Pinen-10-yl-β-vicianoside	$C_{21}H_{34}O_{10}$	50.32	0.06
29	4-Methoxybenzal-dehyde-2-O-β-D-xylosyl(1→6)β-D-glucopyranoside	$C_{19}H_{26}O_{12}$	Unidentified	Unidentified
30	Asperulosidic acid	$C_{18}H_{24}O_{12}$	8.73	0.58
31	Neolinustatin	$C_{17}H_{29}NO_{11}$	Unidentified	Unidentified
32	Fuzinoside	$C_{15}H_{28}O_{13}$	Unidentified	Unidentified
33	Asperuloside	$C_{18}H_{22}O_{11}$	26.50	0.17
34	Bruceine E_1	$C_{20}H_{28}O_{9}$	10.59	0.74
35	Linustatin	$C_{16}H_{27}NO_{11}$	2.54	0.41
36	Erythro-dihydroxyde-hydrodiconiferyl alcohol	$C_{20}H_{24}O_{8}$	27.85	0.47
37	Apocynoside I	$C_{19}H_{30}O_{8}$	Unidentified	Unidentified
38	Calycanthoside	$C_{17}H_{20}O_{10}$	Unidentified	Unidentified
39	Sinapaldehyde glucoside	$C_{17}H_{22}O_{9}$	20.91	0.33
40	Isomaltose	$C_{12}H_{22}O_{11}$	Unidentified	Unidentified
41	1-O-Caffeoyl-β-D-glucopyranoside	$C_{15}H_{18}O_{9}$	Unidentified	Unidentified

42	Sanleng acid	$C_{18}H_{34}O_5$	13.37	0.20
43	Piscidic aciddiethyl ester	$C_{15}H_{20}O_7$	Unidentified	Unidentified
44	9,16-Dioxyhydroxy-10,12,14-triene-18 carbonic acid	$C_{18}H_{30}O_4$	Unidentified	Unidentified
45	Flazin	$C_{17}H_{12}N_2O_4$	94.28	0.39
46	Fawcettiine	$C_{18}H_{29}NO_3$	Unidentified	Unidentified
47	Medicagol	$C_{16}H_8O_6$	57.49	0.60
48	Astragaline E	$C_{14}H_{16}N_2O_5$	14.03	0.74
49	2′-Hydroxy-4′,6′-dimethoxydihydrochalcone	C17H18O4	Unidentified	Unidentified
50	Tribulusterine	$C_{16}H_{12}N_2O_2$	Unidentified	Unidentified
51	Sinapic acid	$C_{11}H_{12}O_5$	64.15	0.08
52	Arteamisinine I	$C_{13}H_{18}O_2$	Unidentified	Unidentified
53	Sebacic acid	$C_{10}H_{18}O_4$	16.23	0.05
54	Eucommiol	$C_9H_{16}O_4$	40.17	0.05
55	Tyrosine	$C_9H_{11}NO_3$	57.55	0.05
56	Galactose	$C_6H_{12}O_6$	10.22	0.55

57	Mesoinositol	$C_6H_{12}O_6$	15.55	0.05
58	Phenylpropionic acid	$C_9H_{11}NO_2$	35.83	0.03
59	E-p-Coumatic acid	$C_9H_8O_3$	Unidentified	Unidentified
60	Guanine	$C_5H_5N_5O$	42.45	0.04
61	Pentose	$C_5H_{10}O_5$	40.44	0.02
62	Mono-ethyl fumarate	$C_6H_8O_4$	69.77	0.02
63	Methylsuccinic acid	$C_5H_8O_4$	72.07	0.02
64	5R-5-Hydroxymethyl-2(5H)-furanone	$C_5H_6O_3$	Unidentified	Unidentified

Supplementary table S2. Target gene of flazin, Medicagol, and Scropolioside A. Data retrieved from Swiss Target Prediction database (http://www.swisstargetprediction.ch/)

Target	Common name	Uniprot ID	ChEMBL ID	Target Class
G protein-coupled receptor 44	PTGDR2	Q9Y5Y4	CHEMBL5071	Family A G protein-coupled receptor
Aldo-keto-reductase family 1 member C3	AKR1C3	P42330	CHEMBL4681	Enzyme
Transitional endoplasmic reticulum ATPase	VCP	P55072	CHEMBL1075145	Primary active transporter
Insulin-like growth factor binding protein 3	IGFBP3	P17936	CHEMBL3997	Secreted protein
Aldose reductase	AKR1B1	P15121	CHEMBL1900	Enzyme
P2X purinoceptor 3	P2RX3	P56373	CHEMBL2998	Ligand-gated ion channel
Insulin-like growth factor binding protein 5	IGFBP5	P24593	CHEMBL2665	Secreted protein
Matrix metalloproteinase 9	MMP9	P14780	CHEMBL321	Protease
Matrix metalloproteinase 1	MMP1	P03956	CHEMBL332	Protease
Matrix metalloproteinase 2	MMP2	P08253	CHEMBL333	Protease
Matrix metalloproteinase 14	MMP14	P50281	CHEMBL3869	Protease
Matrix metalloproteinase 8	MMP8	P22894	CHEMBL4588	Protease
Neprilysin (by homology)	MME	P08473	CHEMBL1944	Protease
Carbonic anhydrase II	CA2	P00918	CHEMBL205	Lyase
Liver glycogen phosphorylase	PYGL	P06737	CHEMBL2568	Enzyme
Muscle glycogen phosphorylase	PYGM	P11217	CHEMBL3526	Enzyme
Carbonic anhydrase I	CA1	P00915	CHEMBL261	Lyase
Angiotensin-converting enzyme (by homology)	ACE	P12821	CHEMBL1808	Protease
AMP deaminase 3	AMPD3	Q01432	CHEMBL2912	Enzyme
Aminopeptidase N	ANPEP	P15144	CHEMBL1907	Protease
Leukotriene A4 hydrolase	LTA4H	P09960	CHEMBL4618	Protease
Endothelin-converting enzyme 1	ECE1	P42892	CHEMBL4791	Protease
HMG-CoA reductase	HMGCR	P04035	CHEMBL402	Oxidoreductase
Renin	REN	P00797	CHEMBL286	Protease
Casein kinase II alpha	CSNK2A1	P68400	CHEMBL3629	Kinase

MAP kinase signal-integrating kinase 2	MKNK2	Q9HBH9	CHEMBL4204	Kinase
Intercellular adhesion molecule (ICAM-1), Integrin alpha-L/beta-2	ITGAL ICAM1 ITGB2	P20701 P05362 P05107	CHEMBL2096661	Membrane receptor
Solute carrier family 22 member 12	SLC22A12	Q96S37	CHEMBL6120	Electrochemical transporter
Methionine aminopeptidase 2	METAP2	P50579	CHEMBL3922	Protease
Lysine-specific demethylase 4A	KDM4A	O75164	CHEMBL5896	Eraser
Purine nucleoside phosphorylase	PNP	P00491	CHEMBL4338	Enzyme
CREB-binding protein/p53	CREBBP	Q92793	CHEMBL5747	Writer
NADPH oxidase 4	NOX4	Q9NPH5	CHEMBL1250375	Enzyme
Epidermal growth factor receptor erbB1	EGFR	P00533	CHEMBL203	Kinase
Hydroxycarboxylic acid receptor 2	HCAR2	Q8TDS4	CHEMBL3785	Family A G protein-coupled receptor
Dual specificity phosphatase Cdc25B	CDC25B	P30305	CHEMBL4804	Phosphatase
CaM-kinase kinase beta	CAMKK2	Q96RR4	CHEMBL5284	Kinase
Glutamate carboxypeptidase II	FOLH1	Q04609	CHEMBL1892	Protease
Matrix metalloproteinase 16	MMP16	P51512	CHEMBL2200	Protease
Lysine-specific demethylase 3A	KDM3A	Q9Y4C1	CHEMBL1938209	Eraser
Lysine-specific demethylase 5B	KDM5B	Q9UGL1	CHEMBL3774295	Eraser
Lysine-specific demethylase 4D	KDM4D	Q6B0I6	CHEMBL6138	Eraser
Lysine-specific demethylase 4C	KDM4C	Q9H3R0	CHEMBL6175	Eraser
Matrix metalloproteinase 3	MMP3	P08254	CHEMBL283	Protease
Selectin E	SELE	P16581	CHEMBL3890	Adhesion
P-selectin	SELP	P16109	CHEMBL5378	Adhesion
Bcl-2-related protein A1	BCL2A1	Q16548	CHEMBL6044	Unclassified protein
Serine/threonine-protein kinase/endoribonuclease IRE1	ERN1	O75460	CHEMBL1163101	Enzyme
Carnitine O-palmitoyltransferase 1, liver isoform	CPT1A	P50416	CHEMBL1293194	Enzyme
Peroxisome proliferator-activated receptor alpha	PPARA	Q07869	CHEMBL239	Nuclear receptor
Heat shock protein HSP 90-alpha	HSP90AA1	P07900	CHEMBL3880	Other cytosolic protein
Heat shock protein HSP 90-beta	HSP90AB1	P08238	CHEMBL4303	Other cytosolic protein

Hepatocyte growth factor receptor	MET	P08581	CHEMBL3717	Kinase
c-Jun N-terminal kinase 1	MAPK8	P45983	CHEMBL2276	Kinase
Cyclooxygenase-2	PTGS2	P35354	CHEMBL230	Oxidoreductase
Integrin alpha-4/beta-1	ITGB1 ITGA4	P05556 P13612	CHEMBL1907599	Membrane receptor
Sodium/glucose cotransporter 2	SLC5A2	P31639	CHEMBL3884	Electrochemical transporter
Squalene synthetase (by homology)	FDFT1	P37268	CHEMBL3338	Enzyme
Serine/threonine-protein kinase WEE1	WEE1	P30291	CHEMBL5491	Kinase
6-phosphofructo-2-kinase/fructose-2,6-bisphosphatase 4	PFKFB4	Q16877	CHEMBL3721311	Enzyme
Fructose-1,6-bisphosphatase	FBP1	P09467	CHEMBL3975	Enzyme
Poly [ADP-ribose] polymerase-1	PARP1	P09874	CHEMBL3105	Enzyme
Fatty acid binding protein adipocyte	FABP4	P15090	CHEMBL2083	Fatty acid binding protein family
Cyclooxygenase-1	PTGS1	P23219	CHEMBL221	Oxidoreductase
Transthyretin	TTR	P02766	CHEMBL3194	Secreted protein
Aldo-keto reductase family 1 member C1	AKR1C1	Q04828	CHEMBL5905	Enzyme
PI3-kinase p110-gamma subunit	PIK3CG	P48736	CHEMBL3267	Enzyme
Eukaryotic translation initiation factor 4H	EIF4H	Q15056	CHEMBL1293274	Other nuclear protein
Polyadenylate-binding protein 1	PABPC1	P11940	CHEMBL1293286	Unclassified protein
Leukocyte adhesion molecule-1	SELL	P14151	CHEMBL3161	Adhesion
AMP deaminase 2	AMPD2	Q01433	CHEMBL2997	Enzyme
Thyroid hormone receptor alpha	THRA	P10827	CHEMBL1860	Nuclear receptor
Thyroid hormone receptor beta-1	THRB	P10828	CHEMBL1947	Nuclear receptor
Insulin-like growth factor I receptor	IGF1R	P08069	CHEMBL1957	Kinase
Carbonic anhydrase XIV	CA14	Q9ULX7	CHEMBL3510	Lyase
Carbonic anhydrase IV	CA4	P22748	CHEMBL3729	Lyase
Histone acetyltransferase p300	EP300	Q09472	CHEMBL3784	Writer
Carbonic anhydrase XIII (by homology)	CA13	Q8N1Q1	CHEMBL3912	Lyase
Tyrosine-protein kinase TIE-2	TEK	Q02763	CHEMBL4128	Kinase
Carbonic anhydrase VA	CA5A	P35218	CHEMBL4789	Lyase

Tyrosinase	TYR	P14679	CHEMBL1973	Oxidoreductase
Dopamine transporter	SLC6A3	Q01959	CHEMBL238	Electrochemical transporter
G-protein coupled receptor 35	GPR35	Q9HC97	CHEMBL1293267	Family A G protein-coupled receptor
DNA polymerase beta	POLB	P06746	CHEMBL2392	Enzyme
Carbonic anhydrase XII	CA12	O43570	CHEMBL3242	Lyase
Carbonic anhydrase IX	CA9	Q16790	CHEMBL3594	Lyase
Vascular endothelial growth factor receptor 1	FLT1	P17948	CHEMBL1868	Kinase
Stem cell growth factor receptor	KIT	P10721	CHEMBL1936	Kinase
Vascular endothelial growth factor receptor 2	KDR	P35968	CHEMBL279	Kinase
Thrombin	F2	P00734	CHEMBL204	Protease
Interleukin-8 receptor B	CXCR2	P25025	CHEMBL2434	Family A G protein-coupled receptor
Tyrosine-protein kinase LCK	LCK	P06239	CHEMBL258	Kinase
ATP-citrate synthase	ACLY	P53396	CHEMBL3720	Enzyme
Cystic fibrosis transmembrane conductance regulator	CFTR	P13569	CHEMBL4051	Other ion channel
GABA-B receptor (by homology)	GABBR1	Q9UBS5	CHEMBL2064	Family C G protein-coupled receptor
Carboxypeptidase A1	CPA1	P15085	CHEMBL2088	Protease
Prostanoid EP1 receptor	PTGER1	P34995	CHEMBL1811	Family A G protein-coupled receptor
Thromboxane-A synthase	TBXAS1	P24557	CHEMBL1835	Cytochrome P450
Caspase-3	CASP3	P42574	CHEMBL2334	Protease
AICAR transformylase	ATIC	P31939	CHEMBL2518	Enzyme
Arachidonate 5-lipoxygenase	ALOX5	P09917	CHEMBL215	Oxidoreductase
Carbonyl reductase [NADPH] 1	CBR1	P16152	CHEMBL5586	Enzyme
Estrogen receptor alpha	ESR1	P03372	CHEMBL206	Nuclear receptor
Estrogen receptor beta	ESR2	Q92731	CHEMBL242	Nuclear receptor
Carbonic anhydrase XII	CA12	O43570	CHEMBL3242	Lyase
Carbonic anhydrase VII	CA7	P43166	CHEMBL2326	Lyase
Carbonic anhydrase II	CA2	P00918	CHEMBL205	Lyase
Tyrosinase (by homology)	TYR	P14679	CHEMBL1973	Oxidoreductase

Voltage-gated potassium channel subunit Kv1.5	KCNA5	P22460	CHEMBL4306	Voltage-gated ion channel
Voltage-gated potassium channel subunit Kv1.3	KCNA3	P22001	CHEMBL4633	Voltage-gated ion channel
Carbonic anhydrase IV	CA4	P22748	CHEMBL3729	Lyase
Protein-tyrosine phosphatase 4A3	PTP4A3	O75365	CHEMBL4162	Phosphatase
Multidrug resistance-associated protein 1	ABCC1	P33527	CHEMBL3004	Primary active transporter
Thromboxane-A synthase	TBXAS1	P24557	CHEMBL1835	Cytochrome P450
Maltase-glucoamylase	MGAM	O43451	CHEMBL2074	Hydrolase
Serotonin 2a (5-HT2a) receptor	HTR2A	P28223	CHEMBL224	Family A G protein-coupled receptor
Serotonin 2c (5-HT2c) receptor	HTR2C	P28335	CHEMBL225	Family A G protein-coupled receptor
Estradiol 17-beta-dehydrogenase 1	HSD17B1	P14061	CHEMBL3181	Enzyme
Estrogen-related receptor alpha	ESRRA	P11474	CHEMBL3429	Nuclear receptor
Estrogen-related receptor beta	ESRRB	O95718	CHEMBL3751	Nuclear receptor
ATP-binding cassette sub-family G member 2	ABCG2	Q9UNQ0	CHEMBL5393	Primary active transporter
Poly [ADP-ribose] polymerase-1	PARP1	P09874	CHEMBL3105	Enzyme
Tankyrase-2	TNKS2	Q9H2K2	CHEMBL6154	Enzyme
Carbonic anhydrase IX	CA9	Q16790	CHEMBL3594	Lyase
CDC7/DBF4 (Cell division cycle 7-related protein kinase/Activator of S phase kinase)	CDC7	O00311	CHEMBL5443	Kinase
Carbonic anhydrase VB	CA5B	Q9Y2D0	CHEMBL3969	Lyase
Cyclin-dependent kinase 1/cyclin B	CCNB3 CDK1 CCNB1 CCNB2	Q8WWL7 P06493 P14635 O95067	CHEMBL2094127	Other cytosolic protein
Cyclin-dependent kinase 6	CDK6	Q00534	CHEMBL2508	Kinase
Cyclin-dependent kinase 2	CDK2	P24941	CHEMBL301	Kinase
Arachidonate 12-lipoxygenase	ALOX12	P18054	CHEMBL3687	Enzyme
DNA excision repair protein ERCC-5	ERCC5	P28715	CHEMBL4736	Other nuclear protein
Flap endonuclease 1	FEN1	P39748	CHEMBL5027	Enzyme
Arginase-1 (by homology)	ARG1	P05089	CHEMBL1075097	Enzyme
NADPH oxidase 4	NOX4	Q9NPH5	CHEMBL1250375	Enzyme
Cytochrome P450 19A1	CYP19A1	P11511	CHEMBL1978	Cytochrome P450

Tyrosine-protein kinase SYK	SYK	P43405	CHEMBL2599	Kinase
Cystic fibrosis transmembrane conductance regulator	CFTR	P13569	CHEMBL4051	Other ion channel
Aldo-keto reductase family 1 member B10	AKR1B10	O60218	CHEMBL5983	Enzyme
Cyclin-dependent kinase 4/cyclin D1	CCND1 CDK4	P24385 P11802	CHEMBL1907601	Kinase
Platelet-derived growth factor receptor beta	PDGFRB	P09619	CHEMBL1913	Kinase
Vascular endothelial growth factor receptor 3	FLT4	P35916	CHEMBL1955	Kinase
Insulin receptor	INSR	P06213	CHEMBL1981	Kinase
Cyclin-dependent kinase 2/cyclin A	CDK2 CCNA1 CCNA2	P24941 P78396 P20248	CHEMBL2094128	Other cytosolic protein
Serine/threonine-protein kinase PLK4	PLK4	O00444	CHEMBL3788	Kinase
Tyrosine-protein kinase TIE-2	TEK	Q02763	CHEMBL4128	Kinase
Serine/threonine-protein kinase Aurora-A	AURKA	O14965	CHEMBL4722	Kinase
Mitogen-activated protein kinase kinase kinase 8	MAP3K8	P41279	CHEMBL4899	Kinase
Ephrin receptor	EPHB4	P54760	CHEMBL5147	Kinase
Heat shock 70 kDa protein 1	HSPA1A	P0DMV8	CHEMBL5460	Other cytosolic protein
Squalene monooxygenase (by homology)	SQLE	Q14534	CHEMBL3592	Enzyme
Tyrosine-protein kinase FGR (by homology)	FGR	P09769	CHEMBL4454	Kinase
Tyrosine-protein kinase Lyn (by homology)	LYN	P07948	CHEMBL3905	Kinase
Telomerase reverse transcriptase	TERT	O14746	CHEMBL2916	Enzyme
Carbonic anhydrase XIII	CA13	Q8N1Q1	CHEMBL3912	Lyase
Lymphocyte differentiation antigen CD38	CD38	P28907	CHEMBL4660	Enzyme
Cyclooxygenase-2	PTGS2	P35354	CHEMBL230	Oxidoreductase
Casein kinase II alpha	CSNK2A1	P68400	CHEMBL3629	Kinase
Estradiol 17-beta-dehydrogenase 2	HSD17B2	P37059	CHEMBL2789	Enzyme
Epidermal growth factor receptor erbB1	EGFR	P00533	CHEMBL203	Kinase
Aryl hydrocarbon receptor	AHR	P35869	CHEMBL3201	Transcription factor
Induced myeloid leukemia cell differentiation protein Mcl-1	MCL1	Q07820	CHEMBL4361	Other cytosolic protein

Tyrosine-protein kinase SRC	SRC	P12931	CHEMBL267	Kinase
Glyoxalase I	GLO1	Q04760	CHEMBL2424	Enzyme
Beta amyloid A4 protein	APP	P05067	CHEMBL2487	Membrane receptor
Matrix metalloproteinase 9	MMP9	P14780	CHEMBL321	Protease
Matrix metalloproteinase 2	MMP2	P08253	CHEMBL333	Protease
Matrix metalloproteinase 12	MMP12	P39900	CHEMBL4393	Protease
DNA topoisomerase I (by homology)	TOP1	P11387	CHEMBL1781	Isomerase
Insulin-like growth factor I receptor	IGF1R	P08069	CHEMBL1957	Kinase
Serine/threonine-protein kinase Aurora-B	AURKB	Q96GD4	CHEMBL2185	Kinase
Vascular endothelial growth factor receptor 2	KDR	P35968	CHEMBL279	Kinase
Serine/threonine-protein kinase PLK1	PLK1	P53350	CHEMBL3024	Kinase
Hepatocyte growth factor receptor	MET	P08581	CHEMBL3717	Kinase
Aldose reductase	AKR1B1	P15121	CHEMBL1900	Enzyme
Monoamine oxidase A	MAOA	P21397	CHEMBL1951	Oxidoreductase
Serine/threonine-protein kinase Chk1	CHEK1	O14757	CHEMBL4630	Kinase
Glutathione reductase	GSR	P00390	CHEMBL2755	Oxidoreductase
Xanthine dehydrogenase	XDH	P47989	CHEMBL1929	Oxidoreductase
6-phosphofructo-2-kinase/fructose-2,6-bisphosphatase 3	PFKFB3	Q16875	CHEMBL2331053	Enzyme
Insulin-like growth factor binding protein 3	IGFBP3	P17936	CHEMBL3997	Secreted protein
Plasma kallikrein	KLKB1	P03952	CHEMBL2000	Protease
Tissue-type plasminogen activator	PLAT	P00750	CHEMBL1873	Protease
Dihydroorotate dehydrogenase (by homology)	DHODH	Q02127	CHEMBL1966	Oxidoreductase
Thrombin	F2	P00734	CHEMBL204	Protease
Thrombin and coagulation factor X	F10	P00742	CHEMBL244	Protease
Urokinase-type plasminogen activator	PLAU	P00749	CHEMBL3286	Protease
Carbonic anhydrase I	CA1	P00915	CHEMBL261	Lyase
Alpha-synuclein	SNCA	P37840	CHEMBL6152	Unclassified protein
Peroxisome proliferator-activated receptor alpha	PPARA	Q07869	CHEMBL239	Nuclear receptor

Apoptosis regulator Bcl-X	BCL2L1	Q07817	CHEMBL4625	Other ion channel
Glycogen synthase kinase-3 beta	GSK3B	P49841	CHEMBL262	Kinase
Cytochrome P450 1A2	CYP1A2	P05177	CHEMBL3356	Cytochrome P450
Signal transducer and activator of transcription 3	STAT3	P40763	CHEMBL4026	Transcription factor
Serine/threonine-protein kinase RAF	RAF1	P04049	CHEMBL1906	Kinase
Nerve growth factor receptor Trk-A	NTRK1	P04629	CHEMBL2815	Kinase
Cyclin-dependent kinase 1	CDK1	P06493	CHEMBL308	Kinase
Serine/threonine-protein kinase PIM2	PIM2	Q9P1W9	CHEMBL4523	Kinase
Serine/threonine-protein kinase PIM3	PIM3	Q86V86	CHEMBL5407	Kinase
Steroid 5-alpha-reductase 1	SRD5A1	P18405	CHEMBL1787	Oxidoreductase
Protein farnesyltransferase	FNTA FNTB	P49354 P49356	CHEMBL2094108	Enzyme
Heat shock protein HSP 90-alpha	HSP90AA1	P07900	CHEMBL3880	Other cytosolic protein
Matrix metalloproteinase 2	MMP2	P08253	CHEMBL333	Protease
Matrix metalloproteinase 12	MMP12	P39900	CHEMBL4393	Protease
Galectin-3	LGALS3	P17931	CHEMBL4531	Other cytosolic protein
Galectin-1	LGALS1	P09382	CHEMBL4915	Other cytosolic protein
P47929	LGALS7	P47929	CHEMBL5008	Other cytosolic protein
Galectin-9	LGALS9	O00182	CHEMBL5474	Other cytosolic protein
Steroid 5-alpha-reductase 1	SRD5A1	P18405	CHEMBL1787	Oxidoreductase
Sodium/glucose cotransporter 1	SLC5A1	P13866	CHEMBL4979	Electrochemical transporter
Adenosine A1 receptor	ADORA1	P30542	CHEMBL226	Family A G protein-coupled receptor
Adenosine A2a receptor	ADORA2A	P29274	CHEMBL251	Family A G protein-coupled receptor
Adenosine A3 receptor	ADORA3	P0DMS8	CHEMBL256	Family A G protein-coupled receptor
Protein kinase C alpha	PRKCA	P17252	CHEMBL299	Kinase
Sodium/potassium-transporting ATPase alpha-1 chain	ATP1A1	P05023	CHEMBL1807	Primary active transporter
DNA topoisomerase I	TOP1	P11387	CHEMBL1781	Isomerase
Poly [ADP-ribose] polymerase-1	PARP1	P09874	CHEMBL3105	Enzyme
Matrix metalloproteinase 9	MMP9	P14780	CHEMBL321	Protease

Matrix metalloproteinase 8	MMP8	P22894	CHEMBL4588	Protease
Receptor-type tyrosine-protein phosphatase alpha	PTPRA	P18433	CHEMBL3918	Phosphatase
Caspase-3	CASP3	P42574	CHEMBL2334	Protease
Coagulation factor VII	F7	P08709	CHEMBL3991	Protease
Integrin alpha-V/beta-3	ITGAV ITGB3	P06756 P05106	CHEMBL1907598	Membrane receptor
Serine/threonine-protein kinase Aurora-B	AURKB	Q96GD4	CHEMBL2185	Kinase
Serine/threonine-protein kinase Aurora-A	AURKA	O14965	CHEMBL4722	Kinase
Beta-glucocerebrosidase (by homology)	GBA	P04062	CHEMBL2179	Enzyme
Mu opioid receptor (by homology)	OPRM1	P35372	CHEMBL233	Family A G protein-coupled receptor
Delta opioid receptor (by homology)	OPRD1	P41143	CHEMBL236	Family A G protein-coupled receptor
CD22	CD22	P20273	CHEMBL3218	Adhesion
Neurokinin 1 receptor	TACR1	P25103	CHEMBL249	Family A G protein-coupled receptor
Growth factor receptor-bound protein 2	GRB2	P62993	CHEMBL3663	Other cytosolic protein
Galectin-4	LGALS4	P56470	CHEMBL1671608	Other cytosolic protein
Angiotensin-converting enzyme (by homology)	ACE	P12821	CHEMBL1808	Protease
Galectin-8	LGALS8	O00214	CHEMBL5475	Other cytosolic protein
Integrin alpha-V/beta-6	ITGAV ITGB6	P06756 P18564	CHEMBL2111416	Membrane receptor
Thymidylate synthase (by homology)	TYMS	P04818	CHEMBL1952	Transferase
Programmed cell death protein 4	PDCD4	Q53EL6	CHEMBL1781868	Unclassified protein
Caspase-1	CASP1	P29466	CHEMBL4801	Protease
Protein kinase C delta (by homology)	PRKCD	Q05655	CHEMBL2996	Kinase
Glutamate carboxypeptidase II	FOLH1	Q04609	CHEMBL1892	Protease
P-selectin	SELP	P16109	CHEMBL5378	Adhesion
3-phosphoinositide dependent protein kinase-1	PDPK1	O15530	CHEMBL2534	Kinase
Isoleucyl-tRNA synthetase	IARS	P41252	CHEMBL3235	Enzyme
Histone deacetylase 6	HDAC6	Q9UBN7	CHEMBL1865	Eraser
Histone deacetylase 8	HDAC8	Q9BY41	CHEMBL3192	Eraser
Histone deacetylase 1	HDAC1	Q13547	CHEMBL325	Eraser

Tyrosinase	TYR	P14679	CHEMBL1973	Oxidoreductase
Inosine-5'-monophosphate dehydrogenase 2	IMPDH2	P12268	CHEMBL2002	Oxidoreductase
6-O-methylguanine-DNA methyltransferase	MGMT	P16455	CHEMBL2864	Enzyme
Trypsin I	PRSS1	P07477	CHEMBL209	Protease
Myelin-associated glycoprotein	MAG	P20916	CHEMBL5807	Unclassified protein
Neprilysin	MME	P08473	CHEMBL1944	Protease
Endothelin-converting enzyme 1	ECE1	P42892	CHEMBL4791	Protease
Integrin alpha-IIb/beta-3	ITGA2B ITGB3	P08514 P05106	CHEMBL2093869	Membrane receptor
Protein farnesyltransferase	FNTA FNTB	P49354 P49356	CHEMBL2094108	Enzyme
Integrin alpha-5/beta-1	ITGB1 ITGA5	P05556 P08648	CHEMBL2095226	Membrane receptor
Angiotensin II receptor	AGTR2	P50052	CHEMBL4607	Family A G protein-coupled receptor
Cathepsin D	CTSD	P07339	CHEMBL2581	Protease
Beta-glucosidase	GBA2	Q9HCG7	CHEMBL3761	Enzyme
Neurokinin 2 receptor	TACR2	P21452	CHEMBL2327	Family A G protein-coupled receptor
Basic fibroblast growth factor	FGF2	P09038	CHEMBL3107	Secreted protein
Kappa Opioid receptor	OPRK1	P41145	CHEMBL237	Family A G protein-coupled receptor
Integrin alpha-4	ITGA4	P13612	CHEMBL278	Membrane receptor
Coagulation factor VII/tissue factor	F3 F7	P13726 P08709	CHEMBL2095194	Protease
Receptor-type tyrosine-protein phosphatase F (LAR)	PTPRF	P10586	CHEMBL3521	Membrane receptor
Methionine aminopeptidase 2	METAP2	P50579	CHEMBL3922	Protease
Insulin-like growth factor I receptor	IGF1R	P08069	CHEMBL1957	Kinase
Coagulation factor IX	F9	P00740	CHEMBL2016	Protease
Integrin alpha-3	ITGA3	P26006	CHEMBL3525	Membrane receptor
Squalene synthetase	FDFT1	P37268	CHEMBL3338	Enzyme
Neurotensin receptor 1	NTSR1	P30989	CHEMBL4123	Family A G protein-coupled receptor
Aldo-keto reductase family 1 member B10	AKR1B10	O60218	CHEMBL5983	Enzyme
Purinergic receptor P2Y12	P2RY12	Q9H244	CHEMBL2001	Family A G protein-coupled receptor
Renin	REN	P00797	CHEMBL286	Protease

Hematopoietic cell protein-tyrosine phosphatase 70Z-PEP	PTPN22	Q9Y2R2	CHEMBL2889	Phosphatase
Interleukin-2	IL2	P60568	CHEMBL5880	Secreted protein
Integrin alpha-4/beta-1	ITGB1 ITGA4	P05556 P13612	CHEMBL1907599	Membrane receptor
Cholecystokinin B receptor	CCKBR	P32239	CHEMBL298	Family A G protein-coupled receptor
Nuclear receptor ROR-gamma	RORC	P51449	CHEMBL1741186	Nuclear receptor
HLA class I histocompatibility antigen A-3	HLA-A	P04439	CHEMBL2632	Surface antigen
Calpain 1	CAPN1	P07384	CHEMBL3891	Protease
Cathepsin (B and K)	CTSB	P07858	CHEMBL4072	Protease
Carbonic anhydrase VI	CA6	P23280	CHEMBL3025	Lyase
Carbonic anhydrase XIII	CA13	Q8N1Q1	CHEMBL3912	Lyase
Carbonic anhydrase VB	CA5B	Q9Y2D0	CHEMBL3969	Lyase
Serine/threonine-protein phosphatase	PPP5C	Q9BPW0	CHEMBL1293265	Phosphatase
Serine/threonine protein phosphatase PP1-alpha catalytic subunit	PPP1CA	P62136	CHEMBL2164	Phosphatase
Transforming protein p21/H-Ras-1	HRAS	P01112	CHEMBL2167	Other cytosolic protein
Peroxisome proliferator-activated receptor gamma	PPARG	P37231	CHEMBL235	Nuclear receptor
Peroxisome proliferator-activated receptor alpha	PPARA	Q07869	CHEMBL239	Nuclear receptor
Ribonucleoside-diphosphate reductase M1 chain (by homology)	RRM1	P23921	CHEMBL1830	Oxidoreductase
Leukocyte common antigen	PTPRC	P08575	CHEMBL3243	Enzyme
Gamma-secretase	PSEN2 PSENEN NCSTN APH1A PSEN1 APH1B	P49810 Q9NZ42 Q92542 Q96BI3 P49768 Q8WW43	CHEMBL2094135	Protease
Hepatic lipase	LIPC	P11150	CHEMBL2127	Enzyme
Phosphodiesterase 4B	PDE4B	Q07343	CHEMBL275	Phosphodiesterase
C3a anaphylatoxin chemotactic receptor	C3AR1	Q16581	CHEMBL4761	Family A G protein-coupled receptor
Endothelial lipase	LIPG	Q9Y5X9	CHEMBL5080	Hydrolase
Glyoxalase I	GLO1	Q04760	CHEMBL2424	Enzyme

Matrix metalloproteinase 3	MMP3	P08254	CHEMBL283	Protease
Matrix metalloproteinase 14	MMP14	P50281	CHEMBL3869	Protease
Matrix metalloproteinase 7	MMP7	P09237	CHEMBL4073	Protease

Supplementary table S3. Target gene of flazin, Medicagol, and Scropolioside A. Data retrieved from SuperPred database (https://prediction.charite.de/index.php).

Target Name	ChEMBL-ID	UniProt ID	PDB Visualization	TTD ID
DNA-(apurinic or apyrimidinic site) lyase	CHEMBL5619	P27695	6BOW	T13348
DNA topoisomerase I	CHEMBL1781	P11387	1K4T	T09826
Tyrosyl-DNA phosphodiesterase 1	CHEMBL1075138	Q9NUW8	6N0D	Not Available
Prostanoid EP1 receptor	CHEMBL1811	P34995	Not Available	T15497
Muscarinic acetylcholine receptor M5	CHEMBL2035	P08912	6OL9	T79961
Dual specificity protein phosphatase 3	CHEMBL2635	P51452	3F81	Not Available
Casein kinase II alpha/beta	CHEMBL3038477	P67870	6TLS	T51565
Histone deacetylase 7	CHEMBL2716	Q8WUI4	3C10	Not Available
Peptidyl-prolyl cis-trans isomerase NIMA-interacting 1	CHEMBL2288	Q13526	1PIN	T16308
Transcription intermediary factor 1-alpha	CHEMBL3108638	O15164	4YBM	Not Available
Glycine transporter 2	CHEMBL3060	Q9Y345	Not Available	Not Available
DNA topoisomerase II alpha	CHEMBL1806	P11388	6ZY5	T17048
Kruppel-like factor 5	CHEMBL1293249	Q13887	Not Available	Not Available
Cathepsin D	CHEMBL2581	P07339	4OD9	T67102
Nuclear receptor ROR-beta	CHEMBL3091268	Q92753	Not Available	Not Available
Nuclear factor NF-kappa-B p105 subunit	CHEMBL3251	P19838	1SVC	Not Available
Pregnane X receptor	CHEMBL3401	O75469	6TFI	T82702
Thyroid hormone receptor alpha	CHEMBL1860	P10827	3ILZ	T79591
Bile acid receptor FXR	CHEMBL2047	Q96RI1	6HL1	Not Available
Dual specificity protein kinase CLK4	CHEMBL4203	Q9HAZ1	6FYV	Not Available
Histone deacetylase 5	CHEMBL2563	Q9UQL6	5UWI	Not Available
Integrin alpha-5/beta-1	CHEMBL2095226	P05556	7NWL	T01851
Dipeptidyl peptidase IX	CHEMBL4793	Q86TI2	6EOR	Not Available
Nuclear factor erythroid 2-related factor 2	CHEMBL1075094	Q16236	2FLU	Not Available
Dual specificity phosphatase Cdc25C	CHEMBL2378	P30307	3OP3	Not Available

Glutathione S-transferase Pi	CHEMBL3902	P09211	5J41	T21669
Niemann-Pick C1 protein	CHEMBL1293277	O15118	6W5S	Not Available
Histone deacetylase 11	CHEMBL3310	Q96DB2	Not Available	Not Available
Adenosine A2b receptor	CHEMBL255	P29275	Not Available	T86679
ADAM10	CHEMBL5028	O14672	6BE6	T31902
MAP kinase ERK2	CHEMBL4040	P28482	6SLG	Not Available
Protein-tyrosine phosphatase LC-PTP	CHEMBL2219	P35236	1ZC0	Not Available
Endoplasmic reticulum aminopeptidase 1	CHEMBL5939	Q9NZ08	6Q4R	Not Available
NADPH oxidase 1	CHEMBL1287628	Q9Y5S8	Not Available	Not Available
C5a anaphylatoxin chemotactic receptor	CHEMBL2373	P21730	6C1R	T15439
Beta-1 adrenergic receptor	CHEMBL213	P08588	7BVQ	T44068
Dual specificity phosphatase Cdc25B	CHEMBL4804	P30305	1QB0	Not Available
Arachidonate 12-lipoxygenase	CHEMBL3687	P18054	3D3L	Not Available
Phosphodiesterase 11A	CHEMBL2717	Q9HCR9	Not Available	Not Available
Vascular endothelial growth factor receptor 1	CHEMBL1868	P17948	5T89	Not Available
Proteasome subunit beta type-9	CHEMBL1944495	P28065	6E5B	Not Available
Protein Mdm4	CHEMBL1255126	O15151	6Q9Y	T36741
G-protein coupled receptor 55	CHEMBL1075322	Q9Y2T6	Not Available	T87670
Galectin-3	CHEMBL4531	P17931	6FOF	T72038
Cysteine protease ATG4B	CHEMBL1741221	Q9Y4P1	2CY7	Not Available
Multidrug resistance-associated protein 1	CHEMBL3004	P33527	4C3Z	T11288
Histone deacetylase 3	CHEMBL1829	O15379	4A69	T05090
Beta-glucuronidase	CHEMBL2728	P08236	3HN3	T96413
Prostanoid EP2 receptor	CHEMBL1881	P43116	7CX2	T38529
Histone deacetylase 9	CHEMBL4145	Q9UKV0	Not Available	Not Available
Coagulation factor XIII	CHEMBL4530	P00488	4KTY	Not Available
Lysosomal Pro-X carboxypeptidase	CHEMBL2335	P42785	3N2Z	Not Available
Glutamate receptor ionotropic, AMPA 2	CHEMBL4016	P42262	2WJW	T42392
Muscarinic acetylcholine receptor M1	CHEMBL216	P11229	6OIJ	T28893

Plasminogen activator inhibitor-1	CHEMBL3475	P05121	3CVM	T15556
Glycogen synthase kinase-3 alpha	CHEMBL2850	P49840	Not Available	Not Available
G protein-coupled receptor kinase 5	CHEMBL5678	P34947	4TND	Not Available
Glucose transporter	CHEMBL2535	P11166	6THA	Not Available
Ras-related protein Rab-9A	CHEMBL1293294	P51151	1WMS	T66350
Activin receptor type-1B	CHEMBL5310	P36896	Not Available	Not Available
Histone deacetylase 8	CHEMBL3192	Q9BY41	5VI6	T28887
Integrin alpha-V/beta-3	CHEMBL1907598	P05106	6UJA	T67103
Tyrosine-protein kinase ITK/TSK	CHEMBL2959	Q08881	4HCU	T91761
Dipeptidyl peptidase II	CHEMBL3976	Q9UHL4	4EBB	Not Available
Cystic fibrosis transmembrane conductance regulator	CHEMBL4051	P13569	6MSM	T55654
Lipoxin A4 receptor	CHEMBL4227	P25090	6OMM	Not Available
Muscarinic acetylcholine receptor M4	CHEMBL1821	P08173	5DSG	T20709
Acetyl-CoA carboxylase 2	CHEMBL4829	O00763	3TDC	T08922
Proteasome component C5	CHEMBL4208	P20618	6KWY	Not Available
Cyclin-dependent kinase 1/cyclin B	CHEMBL2094127	P06493	6GU2	T49898
Signal transducer and activator of transcription 1-alpha/beta	CHEMBL6101	P42224	1YVL	T64205
Nuclear receptor subfamily 4 group A member 1	CHEMBL1293229	P22736	4RZF	Not Available
C-X-C chemokine receptor type 4	CHEMBL2107	P61073	3ODU	T96079
Carbonic anhydrase III	CHEMBL2885	P07451	3UYQ	Not Available
Mitogen-activated protein kinase kinase kinase 11	CHEMBL2708	Q16584	5K26	Not Available
Bcr/Abl fusion protein	CHEMBL2096618	P00519	5N7E	Not Available
Solute carrier family 40 member 1	CHEMBL3392948	Q9NP59	6WBV	T86264
Tyrosine-protein kinase receptor UFO	CHEMBL4895	P30530	5U6B	T82383
Formyl peptide receptor 1	CHEMBL3359	P21462	Not Available	T87831
Caspase-6	CHEMBL3308	P55212	2WDP	Not Available
Sodium channel protein type III alpha subunit	CHEMBL5163	Q9NY46	Not Available	T76937
Aurora kinase B/Inner centromere protein	CHEMBL3430907	Q96GD4	6YIH	T46781
Dual-specificity tyrosine-phosphorylation regulated kinase 1A	CHEMBL2292	Q13627	6S14	T92803

Growth factor receptor-bound protein 2	CHEMBL3663	P62993	1GRI	Not Available
Cathepsin S	CHEMBL2954	P25774	2C0Y	T68290
DNA-(apurinic or apyrimidinic site) lyase	CHEMBL5619	P27695	6BOW	T13348
Arachidonate 12-lipoxygenase	CHEMBL3687	P18054	3D3L	Not Available
Cathepsin D	CHEMBL2581	P07339	4OD9	T67102
Dual specificity protein kinase CLK4	CHEMBL4203	Q9HAZ1	6FYV	Not Available
DNA topoisomerase II alpha	CHEMBL1806	P11388	6ZY5	T17048
Transcription intermediary factor 1-alpha	CHEMBL3108638	O15164	4YBM	Not Available
Dual-specificity tyrosine-phosphorylation regulated kinase 1A	CHEMBL2292	Q13627	6S14	T92803
Heat shock protein HSP 90-beta	CHEMBL4303	P08238	5FWK	Not Available
Casein kinase II alpha/beta	CHEMBL3038477	P67870	6TLS	T51565
Kruppel-like factor 5	CHEMBL1293249	Q13887	Not Available	Not Available
15-hydroxyprostaglandin dehydrogenase [NAD+]	CHEMBL1293255	P15428	2GDZ	Not Available
Endoplasmic reticulum-associated amyloid beta-peptide-binding protein	CHEMBL4159	Q99714	2O23	Not Available
Pregnane X receptor	CHEMBL3401	O75469	6TFI	T82702
Nitric oxide synthase, inducible	CHEMBL4481	P35228	3E7G	T02703
Glycine receptor subunit alpha-1	CHEMBL5845	P23415	4X5T	T50269
Thyroid hormone receptor alpha	CHEMBL1860	P10827	3ILZ	T79591
Glucose transporter	CHEMBL2535	P11166	6THA	Not Available
Transthyretin	CHEMBL3194	P02766	6SUG	T86462
Dual specificity protein phosphatase 3	CHEMBL2635	P51452	3F81	Not Available
Estrogen receptor beta	CHEMBL242	Q92731	1QKM	T80896
Dual specificity phosphatase Cdc25B	CHEMBL4804	P30305	1QB0	Not Available
C5a anaphylatoxin chemotactic receptor	CHEMBL2373	P21730	6C1R	T15439
Galectin-3	CHEMBL4531	P17931	6FOF	T72038
LSD1/CoREST complex	CHEMBL3137262	O60341	5L3D	Not Available
Monoamine oxidase A	CHEMBL1951	P21397	2Z5Y	Not Available

Phosphodiesterase 11A	CHEMBL2717	Q9HCR9	Not Available	Not Available
NT-3 growth factor receptor	CHEMBL5608	Q16288	6KZD	Not Available
Glycine transporter 2	CHEMBL3060	Q9Y345	Not Available	Not Available
Proteasome component C5	CHEMBL4208	P20618	6KWY	Not Available
Protein-tyrosine phosphatase 2C	CHEMBL3864	Q06124	5EHR	T13057
Coagulation factor XIII	CHEMBL4530	P00488	4KTY	Not Available
Dual specificity phosphatase Cdc25C	CHEMBL2378	P30307	3OP3	Not Available
L-lactate dehydrogenase B chain	CHEMBL4940	P07195	1I0Z	Not Available
Calpain 1	CHEMBL3891	P07384	1ZCM	Not Available
Protein kinase N1	CHEMBL3384	Q16512	4OTH	Not Available
Pyroglutamylated RFamide peptide receptor	CHEMBL5852	Q96P65	Not Available	Not Available
Mineralocorticoid receptor	CHEMBL1994	P08235	4PF3	Not Available
Peptidyl-prolyl cis-trans isomerase NIMA-interacting 1	CHEMBL2288	Q13526	1PIN	T16308
HERG	CHEMBL240	Q12809	5VA1	T20251
T-cell protein-tyrosine phosphatase	CHEMBL3807	P17706	1L8K	Not Available
Activin receptor type-1B	CHEMBL5310	P36896	Not Available	Not Available
Protein kinase N2	CHEMBL3032	Q16513	4CRS	Not Available
IgG receptor FcRn large subunit p51	CHEMBL5966	P55899	6FGB	Not Available
Nuclear receptor ROR-beta	CHEMBL3091268	Q92753	Not Available	Not Available
Macrophage migration inhibitory factor	CHEMBL2085	P14174	6B1K	T39977
Tissue factor pathway inhibitor	CHEMBL3713062	P10646	5NMV	T78890
Prostanoid EP1 receptor	CHEMBL1811	P34995	Not Available	T15497
Acetyl-CoA carboxylase 1	CHEMBL3351	Q13085	6G2H	Not Available
Serotonin 2c (5-HT2c) receptor	CHEMBL225	P28335	6BQH	T83813
Lysosomal Pro-X carboxypeptidase	CHEMBL2335	P42785	3N2Z	Not Available
Nuclear receptor subfamily 4 group A member 1	CHEMBL1293229	P22736	4RZF	Not Available
Heat shock protein HSP 90-alpha	CHEMBL3880	P07900	3Q6M	Not Available
Cytochrome P450 3A4	CHEMBL340	P08684	5VCC	T37848
Glutaminase kidney isoform, mitochondrial	CHEMBL2146302	O94925	3UO9	T86734

Beta-1 adrenergic receptor	CHEMBL213	P08588	7BVQ	T44068
Histone deacetylase 8	CHEMBL3192	Q9BY41	5VI6	T28887
Tyrosine-protein kinase ZAP-70	CHEMBL2803	P43403	2OZO	Not Available
E3 SUMO-protein ligase CBX4	CHEMBL3232685	O00257	5EPL	Not Available
Protein kinase C zeta	CHEMBL3438	Q05513	Not Available	T59190
Matrix metalloproteinase 12	CHEMBL4393	P39900	3BA0	Not Available
G-protein coupled receptor 6	CHEMBL3714130	P46095	Not Available	Not Available
Serine/threonine-protein kinase receptor R3	CHEMBL5311	P37023	3MY0	T36959
Dual specificity protein kinase CLK2	CHEMBL4225	P49760	6KHE	Not Available
Stimulator of interferon genes protein	CHEMBL4523377	Q86WV6	6NT5	Not Available
Glutamate NMDA receptor; GRIN1/GRIN2B	CHEMBL1907603	Q05586	5EWM	Not Available
Dual specificity tyrosine-phosphorylation-regulated kinase 1B	CHEMBL5543	Q9Y463	Not Available	Not Available
Ribosomal protein S6 kinase alpha 1	CHEMBL2553	Q15418	2Z7Q	Not Available
Neurokinin 2 receptor	CHEMBL2327	P21452	Not Available	T52790
Urokinase-type plasminogen activator	CHEMBL3286	P00749	1GJ7	T17758
DNA topoisomerase I	CHEMBL1781	P11387	1K4T	T09826
Dual specificity mitogen-activated protein kinase kinase 2	CHEMBL2964	P36507	1S9I	Not Available
NADPH oxidase 1	CHEMBL1287628	Q9Y5S8	Not Available	Not Available
Integrin alpha-5/beta-1	CHEMBL2095226	P05556	7NWL	T01851
Endoplasmic reticulum aminopeptidase 1	CHEMBL5939	Q9NZ08	6Q4R	Not Available
Beta-glucosidase	CHEMBL3761	Q9HCG7	Not Available	Not Available
Proteasome subunit beta type-9	CHEMBL1944495	P28065	6E5B	Not Available
Dual specificty protein kinase CLK1	CHEMBL4224	P49759	6KHD	Not Available
Dipeptidyl peptidase IX	CHEMBL4793	Q86TI2	6EOR	Not Available
Growth factor receptor-bound protein 2	CHEMBL3663	P62993	1GRI	Not Available
Sodium/hydrogen exchanger 1	CHEMBL2781	P19634	7DSX	T82028
Nitric-oxide synthase, endothelial	CHEMBL4803	P29474	4D1P	T06046
Cyclin-dependent kinase 1/cyclin B	CHEMBL2094127	P06493	6GU2	T49898
ADAM17	CHEMBL3706	P78536	2I47	T82393

Nuclear factor erythroid 2-related factor 2	CHEMBL1075094	Q16236	2FLU	Not Available
Ghrelin receptor	CHEMBL4616	Q92847	6KO5	T59604
Kelch-like ECH-associated protein 1	CHEMBL2069156	Q14145	6WCQ	Not Available
Neurotensin receptor 2	CHEMBL2514	O95665	Not Available	Not Available
ADAM10	CHEMBL5028	O14672	6BE6	T31902
Tyrosine-protein kinase FGR	CHEMBL4454	P09769	Not Available	Not Available
Hypoxia-inducible factor 1 alpha	CHEMBL4261	Q16665	4H6J	Not Available
Cathepsin S	CHEMBL2954	P25774	2C0Y	T68290
Formyl peptide receptor 1	CHEMBL3359	P21462	Not Available	T87831
Uracil nucleotide/cysteinyl leukotriene receptor	CHEMBL1075162	Q13304	Not Available	Not Available
Tyrosine-protein kinase ITK/TSK	CHEMBL2959	Q08881	4HCU	T91761
DNA-(apurinic or apyrimidinic site) lyase	CHEMBL5619	P27695	6BOW	T13348
Kruppel-like factor 5	CHEMBL1293249	Q13887	Not Available	Not Available
Nuclear factor erythroid 2-related factor 2	CHEMBL1075094	Q16236	2FLU	Not Available
Hypoxia-inducible factor 1 alpha	CHEMBL4261	Q16665	4H6J	Not Available
Nuclear factor NF-kappa-B p105 subunit	CHEMBL3251	P19838	1SVC	Not Available
Transcription intermediary factor 1-alpha	CHEMBL3108638	O15164	4YBM	Not Available
Platelet-derived growth factor receptor	CHEMBL2095189	P09619	3MJG	T53524
DNA topoisomerase II alpha	CHEMBL1806	P11388	6ZY5	T17048
Proteasome component C5	CHEMBL4208	P20618	6KWY	Not Available
G-protein coupled receptor 6	CHEMBL3714130	P46095	Not Available	Not Available
Ras-related protein Rab-9A	CHEMBL1293294	P51151	1WMS	T66350
Glutamate receptor ionotropic, AMPA 2	CHEMBL4016	P42262	2WJW	T42392
Glycine transporter 2	CHEMBL3060	Q9Y345	Not Available	Not Available
Pregnane X receptor	CHEMBL3401	O75469	6TFI	T82702
Glutamate NMDA receptor; GRIN1/GRIN2B	CHEMBL1907603	Q05586	5EWM	Not Available
Dual specificity protein phosphatase 3	CHEMBL2635	P51452	3F81	Not Available
Cathepsin D	CHEMBL2581	P07339	4OD9	T67102
Dual specificity protein kinase CLK4	CHEMBL4203	Q9HAZ1	6FYV	Not Available

Niemann-Pick C1 protein	CHEMBL1293277	O15118	6W5S	Not Available
LSD1/CoREST complex	CHEMBL3137262	O60341	5L3D	Not Available
Platelet-derived growth factor receptor alpha	CHEMBL2007	P16234	7LBF	T53524
Adenosine A1 receptor	CHEMBL226	P30542	5N2S	T92072
Muscarinic acetylcholine receptor M4	CHEMBL1821	P08173	5DSG	T20709
C5a anaphylatoxin chemotactic receptor	CHEMBL2373	P21730	6C1R	T15439
Nitric oxide synthase, inducible	CHEMBL4481	P35228	3E7G	T02703
Nuclear receptor ROR-beta	CHEMBL3091268	Q92753	Not Available	Not Available
Cyclin-dependent kinase 1/cyclin B1	CHEMBL1907602	P06493	6GU2	T49898
NT-3 growth factor receptor	CHEMBL5608	Q16288	6KZD	Not Available
Phosphodiesterase 3A	CHEMBL241	Q14432	7LRC	T88975
Cytochrome P450 3A4	CHEMBL340	P08684	5VCC	T37848
Hexokinase type IV	CHEMBL3820	P35557	3F9M	T87166
Sodium channel protein type IV alpha subunit	CHEMBL2072	P35499	6AGF	T02546
Lysosomal Pro-X carboxypeptidase	CHEMBL2335	P42785	3N2Z	Not Available
Cyclooxygenase-1	CHEMBL221	P23219	6Y3C	Not Available
PI3-kinase p110-alpha/p85-alpha	CHEMBL2111367	P27986	4JPS	T80276
Vascular endothelial growth factor receptor 1	CHEMBL1868	P17948	5T89	Not Available
Muscarinic acetylcholine receptor M2	CHEMBL211	P08172	5ZKC	T46185
T-cell protein-tyrosine phosphatase	CHEMBL3807	P17706	1L8K	Not Available
Inhibitor of nuclear factor kappa B kinase alpha subunit	CHEMBL3476	O15111	5EBZ	Not Available
Histone deacetylase 5	CHEMBL2563	Q9UQL6	5UWI	Not Available
G protein-coupled receptor kinase 5	CHEMBL5678	P34947	4TND	Not Available
ADAM10	CHEMBL5028	O14672	6BE6	T31902
Mineralocorticoid receptor	CHEMBL1994	P08235	4PF3	Not Available
Toll-like receptor 4	CHEMBL5255	O00206	4G8A	T81443
Monoamine oxidase A	CHEMBL1951	P21397	2Z5Y	Not Available
Sphingosine 1-phosphate receptor Edg-8	CHEMBL2274	Q9H228	Not Available	T50089
Histone deacetylase 2	CHEMBL1937	Q92769	7KBG	T51191

Aminopeptidase N	CHEMBL1907	P15144	4FYT	T67272
Arachidonate 12-lipoxygenase	CHEMBL3687	P18054	3D3L	Not Available
Signal transducer and activator of transcription 1-alpha/beta	CHEMBL6101	P42224	1YVL	T64205
Acetyl-CoA carboxylase 2	CHEMBL4829	O00763	3TDC	T08922
Trypsin I	CHEMBL209	P07477	2RA3	T27602
Sodium channel protein type III alpha subunit	CHEMBL5163	Q9NY46	Not Available	T76937
Cystic fibrosis transmembrane conductance regulator	CHEMBL4051	P13569	6MSM	T55654
Tissue factor pathway inhibitor	CHEMBL3713062	P10646	5NMV	T78890
Dipeptidyl peptidase IX	CHEMBL4793	Q86TI2	6EOR	Not Available
Tyrosine-protein kinase receptor UFO	CHEMBL4895	P30530	5U6B	T82383
Coagulation factor XIII	CHEMBL4530	P00488	4KTY	Not Available
Kinesin-like protein 1	CHEMBL4581	P52732	6TIW	T28484
Methionine aminopeptidase 2	CHEMBL3922	P50579	1B6A	T75596
E3 SUMO-protein ligase CBX4	CHEMBL3232685	O00257	5EPL	Not Available
Lipoxin A4 receptor	CHEMBL4227	P25090	6OMM	Not Available
Casein kinase II alpha/beta	CHEMBL3038477	P67870	6TLS	T51565
Voltage-gated N-type calcium channel alpha-1B subunit	CHEMBL4478	Q00975	Not Available	T38338
PI3-kinase p110-beta subunit	CHEMBL3145	P42338	Not Available	T05031
Protein tyrosine kinase 2 beta	CHEMBL5469	Q14289	4EKU	T07087
Kallikrein 7	CHEMBL2443	P49862	2QXI	Not Available
Aurora kinase B/Inner centromere protein	CHEMBL3430907	Q96GD4	6YIH	T46781
Muscarinic acetylcholine receptor M1	CHEMBL216	P11229	6OIJ	T28893
Pyroglutamylated RFamide peptide receptor	CHEMBL5852	Q96P65	Not Available	Not Available
Growth factor receptor-bound protein 2	CHEMBL3663	P62993	1GRI	Not Available
Plasminogen activator inhibitor-1	CHEMBL3475	P05121	3CVM	T15556
Phosphodiesterase 3B	CHEMBL290	Q13370	1SO2	Not Available
Heat shock protein HSP 90-alpha	CHEMBL3880	P07900	3Q6M	Not Available
Heat shock protein HSP 90-beta	CHEMBL4303	P08238	5FWK	Not Available
Acyl-CoA desaturase	CHEMBL5555	O00767	4ZYO	T10897

Macrophage migration inhibitory factor	CHEMBL2085	P14174	6B1K	T39977
Amine oxidase, copper containing	CHEMBL3437	Q16853	2C10	T69619
5'-nucleotidase	CHEMBL5957	P21589	6TVE	Not Available
Cannabinoid CB1 receptor	CHEMBL218	P21554	6N4B	Not Available
Muscarinic acetylcholine receptor M5	CHEMBL2035	P08912	6OL9	T79961
Excitatory amino acid transporter 1	CHEMBL3085	P43003	5LM4	Not Available
Histone deacetylase 7	CHEMBL2716	Q8WUI4	3C10	Not Available
Transthyretin	CHEMBL3194	P02766	6SUG	T86462
Phosphodiesterase 11A	CHEMBL2717	Q9HCR9	Not Available	Not Available
Protein-tyrosine phosphatase 2C	CHEMBL3864	Q06124	5EHR	T13057
L-lactate dehydrogenase B chain	CHEMBL4940	P07195	1I0Z	Not Available
C-C chemokine receptor type 2	CHEMBL4015	P41597	5T1A	T89988
Vasopressin V1b receptor	CHEMBL1921	P47901	Not Available	Not Available
Formyl peptide receptor 1	CHEMBL3359	P21462	Not Available	T87831
Proteasome Macropain subunit	CHEMBL3492	P49721	5LE5	Not Available

Supplementary table S4. Targets of genes associated in maternal obesity from GeneCards (http://www.genecards.org/).

Gene Symbol	Description	Category	Uniprot ID	Gifts	GC Id	Relevance score	GeneCards Link
LEP	Leptin	Protein Coding	P41159	51	GC07P128241	88.26277	https://www.genecards.org/cgi-bin/carddisp.pl?gene=LEP
MC4R	Melanocortin 4 Receptor	Protein Coding	P32245	50	GC18M060371	87.43517	https://www.genecards.org/cgi-bin/carddisp.pl?gene=MC4R
POMC	Proopiomelanocortin	Protein Coding	P01189	52	GC02M025160	74.34994	https://www.genecards.org/cgi-bin/carddisp.pl?gene=POMC
PPARG	Peroxisome Proliferator Activated Receptor Gamma	Protein Coding	P37231	57	GC03P012287	68.84069	https://www.genecards.org/cgi-bin/carddisp.pl?gene=PPARG
LEPR	Leptin Receptor	Protein Coding	P48357	54	GC01P065429	63.43594	https://www.genecards.org/cgi-bin/carddisp.pl?gene=LEPR
FTO	FTO Alpha-Ketoglutarate Dependent Dioxygenase	Protein Coding	Q9C0B1	51	GC16P055858	57.3583	https://www.genecards.org/cgi-bin/carddisp.pl?gene=FTO
PCSK1	Proprotein Convertase Subtilisin/Kexin Type 1	Protein Coding	P29120	53	GC05M096391	51.63431	https://www.genecards.org/cgi-bin/carddisp.pl?gene=PCSK1
INS	Insulin	Protein Coding	P01308	51	GC11M002159	49.77531	https://www.genecards.org/cgi-bin/carddisp.pl?gene=INS
H19	H19 Imprinted Maternally Expressed Transcript	RNA Gene		28	GC11M001995	48.26858	https://www.genecards.org/cgi-bin/carddisp.pl?gene=H19
GHRL	Ghrelin And Obestatin Prepropeptide	Protein Coding	Q9UBU3	45	GC03M010285	47.42544	https://www.genecards.org/cgi-bin/carddisp.pl?gene=GHRL
BDNF-AS	BDNF Antisense RNA	RNA Gene		22	GC11P027627	47.15899	https://www.genecards.org/cgi-bin/carddisp.pl?gene=BDNF-AS

SMAD5-AS1	SMAD5 Antisense RNA 1	RNA Gene	Q9Y6J3	23	GC05M136129	46.29656	https://www.genecards.org/cgi-bin/carddisp.pl?gene=SMAD5-AS1
GNAS	GNAS Complex Locus	Protein Coding	P84996	54	GC20P058839	45.34686	https://www.genecards.org/cgi-bin/carddisp.pl?gene=GNAS
ENPP1	Ectonucleotide Pyrophosphatase/Phosphodiesterase 1	Protein Coding	P22413	53	GC06P131808	44.23174	https://www.genecards.org/cgi-bin/carddisp.pl?gene=ENPP1
ADIPOQ	Adiponectin, C1Q And Collagen Domain Containing	Protein Coding	Q15848	50	GC03P186842	43.80775	https://www.genecards.org/cgi-bin/carddisp.pl?gene=ADIPOQ
MEG3	Maternally Expressed 3	RNA Gene		29	GC14P113576	43.60498	https://www.genecards.org/cgi-bin/carddisp.pl?gene=MEG3
ADRB3	Adrenoceptor Beta 3	Protein Coding	P13945	47	GC08M037962	43.34451	https://www.genecards.org/cgi-bin/carddisp.pl?gene=ADRB3
UCP2	Uncoupling Protein 2	Protein Coding	P55851	50	GC11M073974	42.69788	https://www.genecards.org/cgi-bin/carddisp.pl?gene=UCP2
LOC101929710	Uncharacterized LOC101929710	RNA Gene		14	GC05P095962	38.22956	https://www.genecards.org/cgi-bin/carddisp.pl?gene=LOC101929710
BDNF	Brain Derived Neurotrophic Factor	Protein Coding	P23560	53	GC11M027654	38.10145	https://www.genecards.org/cgi-bin/carddisp.pl?gene=BDNF
MC3R	Melanocortin 3 Receptor	Protein Coding	P41968	45	GC20P056248	36.63493	https://www.genecards.org/cgi-bin/carddisp.pl?gene=MC3R
SIM1	SIM BHLH Transcription Factor 1	Protein Coding	P81133	46	GC06M100386	35.7021	https://www.genecards.org/cgi-bin/carddisp.pl?gene=SIM1
IL6	Interleukin 6	Protein Coding	P05231	55	GC07P022725	34.05007	https://www.genecards.org/cgi-bin/carddisp.pl?gene=IL6

AGRP	Agouti Related Neuropeptide	Protein Coding	O00253	45	GC16M067482	32.44409	https://www.genecards.org/cgi-bin/carddisp.pl?gene=AGRP
RETN	Resistin	Protein Coding	Q9HD89	46	GC19P007669	30.71975	https://www.genecards.org/cgi-bin/carddisp.pl?gene=RETN
TNF	Tumor Necrosis Factor	Protein Coding	P01375	55	GC06P111998	29.94992	https://www.genecards.org/cgi-bin/carddisp.pl?gene=TNF
ABCC8	ATP Binding Cassette Subfamily C Member 8	Protein Coding	Q09428	50	GC11M017392	29.03263	https://www.genecards.org/cgi-bin/carddisp.pl?gene=ABCC8
SLC52A1	Solute Carrier Family 52 Member 1	Protein Coding	Q9NWF4	44	GC17M005032	28.8852	https://www.genecards.org/cgi-bin/carddisp.pl?gene=SLC52A1
MT-CYB	Mitochondrially Encoded Cytochrome B	Protein Coding	P00156	36	GCMTP014749	28.04844	https://www.genecards.org/cgi-bin/carddisp.pl?gene=MT-CYB
CRP	C-Reactive Protein	Protein Coding	P02741	50	GC01M159733	27.91251	https://www.genecards.org/cgi-bin/carddisp.pl?gene=CRP
IGF1	Insulin Like Growth Factor 1	Protein Coding	P05019	52	GC12M102395	27.78274	https://www.genecards.org/cgi-bin/carddisp.pl?gene=IGF1
NR0B2	Nuclear Receptor Subfamily 0 Group B Member 2	Protein Coding	Q15466	45	GC01M029764	27.47163	https://www.genecards.org/cgi-bin/carddisp.pl?gene=NR0B2
KHDC3L	KH Domain Containing 3 Like, Subcortical Maternal Complex Member	Protein Coding	Q587J8	36	GC06P073362	27.03028	https://www.genecards.org/cgi-bin/carddisp.pl?gene=KHDC3L
MTHFR	Methylenetetrahydrofolate Reductase	Protein Coding	P42898	52	GC01M011785	26.70115	https://www.genecards.org/cgi-bin/carddisp.pl?gene=MTHFR
NTRK2	Neurotrophic Receptor Tyrosine Kinase 2	Protein Coding	Q16620	57	GC09P084668	26.03415	https://www.genecards.org/cgi-bin/carddisp.pl?gene=NTRK2

CARTPT	CART Prepropeptide	Protein Coding	Q16568	46	GC05P071719	25.5757	https://www.genecards.org/cgi-bin/carddisp.pl?gene=CARTPT
SERPINE1	Serpin Family E Member 1	Protein Coding	P05121	54	GC07P101127	24.99662	https://www.genecards.org/cgi-bin/carddisp.pl?gene=SERPINE1
PRMT7	Protein Arginine Methyltransferase 7	Protein Coding	Q9NVM4	47	GC16P068836	24.67075	https://www.genecards.org/cgi-bin/carddisp.pl?gene=PRMT7
IGF2	Insulin Like Growth Factor 2	Protein Coding	P01344	53	GC11M009008	24.08531	https://www.genecards.org/cgi-bin/carddisp.pl?gene=IGF2
DLK1	Delta Like Non-Canonical Notch Ligand 1	Protein Coding	P80370	49	GC14P113020	23.97094	https://www.genecards.org/cgi-bin/carddisp.pl?gene=DLK1
SH2B1	SH2B Adaptor Protein 1	Protein Coding	Q9NRF2	46	GC16P053995	23.82881	https://www.genecards.org/cgi-bin/carddisp.pl?gene=SH2B1
NUDC	Nuclear Distribution C, Dynein Complex Regulator	Protein Coding	Q9Y266	47	GC01P030272	23.12025	https://www.genecards.org/cgi-bin/carddisp.pl?gene=NUDC
SNRPN	Small Nuclear Ribonucleoprotein Polypeptide N	Protein Coding	P63162	48	GC15P024823	23.10315	https://www.genecards.org/cgi-bin/carddisp.pl?gene=SNRPN
PHIP	Pleckstrin Homology Domain Interacting Protein	Protein Coding	Q8WWQ0	45	GC06M078934	23.02861	https://www.genecards.org/cgi-bin/carddisp.pl?gene=PHIP
MT-TL1	Mitochondrially Encoded TRNA-Leu (UUA/G) 1	RNA Gene		18	GCMTP003232	22.30338	https://www.genecards.org/cgi-bin/carddisp.pl?gene=MT-TL1
LOC108167315	POMC Promoter Region	Functional Element		4	GC02P025170	22.24967	https://www.genecards.org/cgi-bin/carddisp.pl?gene=LOC108167315
FFAR4	Free Fatty Acid Receptor 4	Protein Coding	Q5NUL3	44	GC10P093566	22.24725	https://www.genecards.org/cgi-bin/carddisp.pl?gene=FFAR4

APOB	Apolipoprotein B	Protein Coding	P04114	50	GC02M020956	22.17986	https://www.genecards.org/cgi-bin/carddisp.pl?gene=APOB
MTTP	Microsomal Triglyceride Transfer Protein	Protein Coding	P55157	49	GC04P099563	22.01443	https://www.genecards.org/cgi-bin/carddisp.pl?gene=MTTP
POLG	DNA Polymerase Gamma, Catalytic Subunit	Protein Coding	P54098	51	GC15M137554	22.01308	https://www.genecards.org/cgi-bin/carddisp.pl?gene=POLG
LINC01672	Long Intergenic Non-Protein Coding RNA 1672	RNA Gene		13	GC01P007342	21.91381	https://www.genecards.org/cgi-bin/carddisp.pl?gene=LINC01672
INPP5E	Inositol Polyphosphate-5-Phosphatase E	Protein Coding	Q9NRR6	45	GC09M136428	21.82102	https://www.genecards.org/cgi-bin/carddisp.pl?gene=INPP5E
MAGEL2	MAGE Family Member L2	Protein Coding	Q9UJ55	41	GC15M023643	21.73219	https://www.genecards.org/cgi-bin/carddisp.pl?gene=MAGEL2
CDKAL1	CDK5 Regulatory Subunit Associated Protein 1 Like 1	Protein Coding	Q5VV42	44	GC06P020534	21.61253	https://www.genecards.org/cgi-bin/carddisp.pl?gene=CDKAL1
PYY	Peptide YY	Protein Coding	P10082	46	GC17M043952	21.59005	https://www.genecards.org/cgi-bin/carddisp.pl?gene=PYY
LPL	Lipoprotein Lipase	Protein Coding	P06858	53	GC08P019901	21.29872	https://www.genecards.org/cgi-bin/carddisp.pl?gene=LPL
NR3C1	Nuclear Receptor Subfamily 3 Group C Member 1	Protein Coding	P04150	53	GC05M143277	21.21834	https://www.genecards.org/cgi-bin/carddisp.pl?gene=NR3C1
SDC3	Syndecan 3	Protein Coding	O75056	47	GC01M030869	21.159	https://www.genecards.org/cgi-bin/carddisp.pl?gene=SDC3
MELK	Maternal Embryonic Leucine Zipper Kinase	Protein Coding	Q14680	50	GC09P036572	20.99791	https://www.genecards.org/cgi-bin/carddisp.pl?gene=MELK

DNMT3A	DNA Methyltransferase 3 Alpha	Protein Coding	Q9Y6K1	55	GC02M025228	20.91785	https://www.genecards.org/cgi-bin/carddisp.pl?gene=DNMT3A
LEPQTL1	Leptin, Serum Levels Of	Genetic Locus		3	GC02U903086	20.86082	https://www.genecards.org/cgi-bin/carddisp.pl?gene=LEPQTL1
HSD11B1	Hydroxysteroid 11-Beta Dehydrogenase 1	Protein Coding	P28845	54	GC01P209686	20.83038	https://www.genecards.org/cgi-bin/carddisp.pl?gene=HSD11B1
ADRB2	Adrenoceptor Beta 2	Protein Coding	P07550	52	GC05P149032	20.76446	https://www.genecards.org/cgi-bin/carddisp.pl?gene=ADRB2
NOS3	Nitric Oxide Synthase 3	Protein Coding	P29474	53	GC07P153537	20.70619	https://www.genecards.org/cgi-bin/carddisp.pl?gene=NOS3
NPY	Neuropeptide Y	Protein Coding	P01303	48	GC07P024290	20.59429	https://www.genecards.org/cgi-bin/carddisp.pl?gene=NPY
GCK	Glucokinase	Protein Coding	P35557	53	GC07M044887	20.473	https://www.genecards.org/cgi-bin/carddisp.pl?gene=GCK
HADHA	Hydroxyacyl-CoA Dehydrogenase Trifunctional Multienzyme Complex Subunit Alpha	Protein Coding	P40939	51	GC02M026190	20.35906	https://www.genecards.org/cgi-bin/carddisp.pl?gene=HADHA
UBE3A	Ubiquitin Protein Ligase E3A	Protein Coding	Q05086	51	GC15M025333	20.23441	https://www.genecards.org/cgi-bin/carddisp.pl?gene=UBE3A
RTL1	Retrotransposon Gag Like 1	Protein Coding	A6NKG5	34	GC14M116468	19.98217	https://www.genecards.org/cgi-bin/carddisp.pl?gene=RTL1
BRCA2	BRCA2 DNA Repair Associated	Protein Coding	P51587	52	GC13P032315	19.97779	https://www.genecards.org/cgi-bin/carddisp.pl?gene=BRCA2
KCNQ1OT1	KCNQ1 Opposite Strand/Antisense Transcript 1	RNA Gene		28	GC11M009033	19.87074	https://www.genecards.org/cgi-bin/carddisp.pl?gene=KCNQ1OT1

GCG	Glucagon	Protein Coding	P01275	46	GC02M162142	19.83318	https://www.genecards.org/cgi-bin/carddisp.pl?gene=GCG
UCP1	Uncoupling Protein 1	Protein Coding	P25874	47	GC04M140559	19.6952	https://www.genecards.org/cgi-bin/carddisp.pl?gene=UCP1
MKRN3	Makorin Ring Finger Protein 3	Protein Coding	Q13064	44	GC15P059573	19.48784	https://www.genecards.org/cgi-bin/carddisp.pl?gene=MKRN3
NAMPT	Nicotinamide Phosphoribosyltransferase	Protein Coding	P43490	52	GC07M106248	19.36914	https://www.genecards.org/cgi-bin/carddisp.pl?gene=NAMPT
PPARGC1A	PPARG Coactivator 1 Alpha	Protein Coding	Q9UBK2	51	GC04M023755	19.36907	https://www.genecards.org/cgi-bin/carddisp.pl?gene=PPARGC1A
OXT	Oxytocin/Neurophysin I Prepropeptide	Protein Coding	P01178	42	GC20P006757	19.36691	https://www.genecards.org/cgi-bin/carddisp.pl?gene=OXT
ALB	Albumin	Protein Coding	P02768	53	GC04P073397	19.26724	https://www.genecards.org/cgi-bin/carddisp.pl?gene=ALB
GRB10	Growth Factor Receptor Bound Protein 10	Protein Coding	Q13322	47	GC07M050590	19.05449	https://www.genecards.org/cgi-bin/carddisp.pl?gene=GRB10
POLGARF	POLG Alternative Reading Frame	Protein Coding	A0A3B3IS91	11	GC15M140354	18.7235	https://www.genecards.org/cgi-bin/carddisp.pl?gene=POLGARF
PDSS1	Decaprenyl Diphosphate Synthase Subunit 1	Protein Coding	Q5T2R2	46	GC10P026697	18.56379	https://www.genecards.org/cgi-bin/carddisp.pl?gene=PDSS1
IGFBP1	Insulin Like Growth Factor Binding Protein 1	Protein Coding	P08833	48	GC07P048837	18.49575	https://www.genecards.org/cgi-bin/carddisp.pl?gene=IGFBP1
IL1B	Interleukin 1 Beta	Protein Coding	P01584	51	GC02M112829	18.30032	https://www.genecards.org/cgi-bin/carddisp.pl?gene=IL1B

DNM1L	Dynamin 1 Like	Protein Coding	O00429	52	GC12P032679	18.27155	https://www.genecards.org/cgi-bin/carddisp.pl?gene=DNM1L
IRS1	Insulin Receptor Substrate 1	Protein Coding	P35568	51	GC02M226731	18.26258	https://www.genecards.org/cgi-bin/carddisp.pl?gene=IRS1
PRL	Prolactin	Protein Coding	P01236	48	GC06M022287	18.01373	https://www.genecards.org/cgi-bin/carddisp.pl?gene=PRL
IPW	Imprinted In Prader-Willi Syndrome	RNA Gene		17	GC15P025116	18.00114	https://www.genecards.org/cgi-bin/carddisp.pl?gene=IPW
INSR	Insulin Receptor	Protein Coding	P06213	58	GC19M007112	17.98509	https://www.genecards.org/cgi-bin/carddisp.pl?gene=INSR
ACE	Angiotensin I Converting Enzyme	Protein Coding	P12821	55	GC17P063477	17.97792	https://www.genecards.org/cgi-bin/carddisp.pl?gene=ACE
MIR122	MicroRNA 122	RNA Gene		22	GC18P058451	17.91723	https://www.genecards.org/cgi-bin/carddisp.pl?gene=MIR122
SLC2A4	Solute Carrier Family 2 Member 4	Protein Coding	P14672	51	GC17P013998	17.87424	https://www.genecards.org/cgi-bin/carddisp.pl?gene=SLC2A4
HNF1A	HNF1 Homeobox A	Protein Coding	P20823	51	GC12P120978	17.82582	https://www.genecards.org/cgi-bin/carddisp.pl?gene=HNF1A
FLT1	Fms Related Receptor Tyrosine Kinase 1	Protein Coding	P17948	55	GC13M028300	17.80359	https://www.genecards.org/cgi-bin/carddisp.pl?gene=FLT1
MT-ATP6	Mitochondrially Encoded ATP Synthase Membrane Subunit 6	Protein Coding	P00846	36	GCMTP008531	17.69695	https://www.genecards.org/cgi-bin/carddisp.pl?gene=MT-ATP6
ALMS1	ALMS1 Centrosome And Basal Body Associated Protein	Protein Coding	Q8TCU4	45	GC02P073385	17.59045	https://www.genecards.org/cgi-bin/carddisp.pl?gene=ALMS1

KCNH2	Potassium Voltage-Gated Channel Subfamily H Member 2	Protein Coding	Q12809	54	GC07M150944	17.56662	https://www.genecards.org/cgi-bin/carddisp.pl?gene=KCNH2
TP53	Tumor Protein P53	Protein Coding	P04637	57	GC17M007661	17.56162	https://www.genecards.org/cgi-bin/carddisp.pl?gene=TP53
CCL2	C-C Motif Chemokine Ligand 2	Protein Coding	P13500	53	GC17P034255	17.55315	https://www.genecards.org/cgi-bin/carddisp.pl?gene=CCL2
OCA2	OCA2 Melanosomal Transmembrane Protein	Protein Coding	Q04671	47	GC15M027754	17.46606	https://www.genecards.org/cgi-bin/carddisp.pl?gene=OCA2
NDN	Necdin, MAGE Family Member	Protein Coding	Q99608	46	GC15M025943	17.34953	https://www.genecards.org/cgi-bin/carddisp.pl?gene=NDN
MT-TK	Mitochondrially Encoded TRNA-Lys (AAA/G)	RNA Gene		15	GCMTP008297	17.34819	https://www.genecards.org/cgi-bin/carddisp.pl?gene=MT-TK
MT-ND1	Mitochondrially Encoded NADH:Ubiquinone Oxidoreductase Core Subunit 1	Protein Coding	P03886	34	GCMTP003309	17.32558	https://www.genecards.org/cgi-bin/carddisp.pl?gene=MT-ND1
SCN1A	Sodium Voltage-Gated Channel Alpha Subunit 1	Protein Coding	P35498	52	GC02M165989	17.3114	https://www.genecards.org/cgi-bin/carddisp.pl?gene=SCN1A
GH1	Growth Hormone 1	Protein Coding	P01241	47	GC17M063917	17.23219	https://www.genecards.org/cgi-bin/carddisp.pl?gene=GH1
KCNJ11	Potassium Inwardly Rectifying Channel Subfamily J Member 11	Protein Coding	Q14654	50	GC11M017823	17.21778	https://www.genecards.org/cgi-bin/carddisp.pl?gene=KCNJ11
PWAR1	Prader Willi/Angelman Region RNA 1	RNA Gene		16	GC15P025135	17.15276	https://www.genecards.org/cgi-bin/carddisp.pl?gene=PWAR1
APOE	Apolipoprotein E	Protein Coding	P02649	54	GC19P089740	17.121	https://www.genecards.org/cgi-bin/carddisp.pl?gene=APOE

CYP19A1	Cytochrome P450 Family 19 Subfamily A Member 1	Protein Coding	P11511	52	GC15M051208	17.09549	https://www.genecards.org/cgi-bin/carddisp.pl?gene=CYP19A1
PPARA	Peroxisome Proliferator Activated Receptor Alpha	Protein Coding	Q07869	48	GC22P046150	17.08477	https://www.genecards.org/cgi-bin/carddisp.pl?gene=PPARA
RBP4	Retinol Binding Protein 4	Protein Coding	P02753	49	GC10M093591	17.05139	https://www.genecards.org/cgi-bin/carddisp.pl?gene=RBP4
CNR1	Cannabinoid Receptor 1	Protein Coding	P21554	51	GC06M088139	17.01676	https://www.genecards.org/cgi-bin/carddisp.pl?gene=CNR1
NPAP1	Nuclear Pore Associated Protein 1	Protein Coding	Q9NZP6	34	GC15P024675	16.91708	https://www.genecards.org/cgi-bin/carddisp.pl?gene=NPAP1
GHR	Growth Hormone Receptor	Protein Coding	P10912	50	GC05P042429	16.79181	https://www.genecards.org/cgi-bin/carddisp.pl?gene=GHR
IRAK1BP1	Interleukin 1 Receptor Associated Kinase 1 Binding Protein 1	Protein Coding	Q5VVH5	35	GC06P078867	16.73723	https://www.genecards.org/cgi-bin/carddisp.pl?gene=IRAK1BP1
SHBG	Sex Hormone Binding Globulin	Protein Coding	P04278	46	GC17P007613	16.65593	https://www.genecards.org/cgi-bin/carddisp.pl?gene=SHBG
GNB3	G Protein Subunit Beta 3	Protein Coding	P16520	51	GC12P006839	16.6367	https://www.genecards.org/cgi-bin/carddisp.pl?gene=GNB3
MIR143	MicroRNA 143	RNA Gene		25	GC05P149470	16.4246	https://www.genecards.org/cgi-bin/carddisp.pl?gene=MIR143
BBS1	Bardet-Biedl Syndrome 1	Protein Coding	Q8NFJ9	44	GC11P079938	16.39797	https://www.genecards.org/cgi-bin/carddisp.pl?gene=BBS1
GLDC	Glycine Decarboxylase	Protein Coding	P23378	51	GC09M006522	16.35766	https://www.genecards.org/cgi-bin/carddisp.pl?gene=GLDC

IL10	Interleukin 10	Protein Coding	P22301	52	GC01M206767	16.33609	https://www.genecards.org/cgi-bin/carddisp.pl?gene=IL10
NRXN1	Neurexin 1	Protein Coding	Q9ULB1	52	GC02M049918	16.32223	https://www.genecards.org/cgi-bin/carddisp.pl?gene=NRXN1
AGT	Angiotensinogen	Protein Coding	P01019	53	GC01M230690	16.11377	https://www.genecards.org/cgi-bin/carddisp.pl?gene=AGT
LIPC	Lipase C, Hepatic Type	Protein Coding	P11150	50	GC15P058410	15.9655	https://www.genecards.org/cgi-bin/carddisp.pl?gene=LIPC
MYH9	Myosin Heavy Chain 9	Protein Coding	P35579	52	GC22M036281	15.90576	https://www.genecards.org/cgi-bin/carddisp.pl?gene=MYH9
MIR493HG	MIR493 Cluster Host Gene	RNA Gene		13	GC14P113611	15.8982	https://www.genecards.org/cgi-bin/carddisp.pl?gene=MIR493HG
COMT	Catechol-O-Methyltransferase	Protein Coding	P21964	55	GC22P019941	15.85969	https://www.genecards.org/cgi-bin/carddisp.pl?gene=COMT
SOD2-OT1	SOD2 Overlapping Transcript 1	RNA Gene		13	GC06M159772	15.76086	https://www.genecards.org/cgi-bin/carddisp.pl?gene=SOD2-OT1
LIPE	Lipase E, Hormone Sensitive Type	Protein Coding	Q05469	51	GC19M042401	15.69086	https://www.genecards.org/cgi-bin/carddisp.pl?gene=LIPE
CRH	Corticotropin Releasing Hormone	Protein Coding	P06850	47	GC08M066176	15.68692	https://www.genecards.org/cgi-bin/carddisp.pl?gene=CRH
VPS13B	Vacuolar Protein Sorting 13 Homolog B	Protein Coding	Q7Z7G8	44	GC08P099011	15.67846	https://www.genecards.org/cgi-bin/carddisp.pl?gene=VPS13B
HNF4A	Hepatocyte Nuclear Factor 4 Alpha	Protein Coding	P41235	53	GC20P044355	15.54478	https://www.genecards.org/cgi-bin/carddisp.pl?gene=HNF4A

MIR33A	MicroRNA 33a	RNA Gene		20	GC22P041900	15.5258 1	https://www.genecards.org/cgi-bin/carddisp.pl?gene=MIR33A
PAH	Phenylalanine Hydroxylase	Protein Coding	P00439	52	GC12M10283 6	15.4984 4	https://www.genecards.org/cgi-bin/carddisp.pl?gene=PAH
VEGFA	Vascular Endothelial Growth Factor A	Protein Coding	P15692	53	GC06P043770	15.3792 5	https://www.genecards.org/cgi-bin/carddisp.pl?gene=VEGFA
BBS2	Bardet-Biedl Syndrome 2	Protein Coding	Q9BXC9	45	GC16M05646 7	15.2575 1	https://www.genecards.org/cgi-bin/carddisp.pl?gene=BBS2
PON1	Paraoxonase 1	Protein Coding	P27169	51	GC07M09529 7	15.23	https://www.genecards.org/cgi-bin/carddisp.pl?gene=PON1
MKKS	MKKS Centrosomal Shuttling Protein	Protein Coding	Q9NPJ1	45	GC20M01042 4	15.0499 6	https://www.genecards.org/cgi-bin/carddisp.pl?gene=MKKS
CCK	Cholecystokinin	Protein Coding	P06307	46	GC03M04227 4	15.0287 5	https://www.genecards.org/cgi-bin/carddisp.pl?gene=CCK
HCRT	Hypocretin Neuropeptide Precursor	Protein Coding	O43612	44	GC17M06416 1	15.0120 9	https://www.genecards.org/cgi-bin/carddisp.pl?gene=HCRT
IL18	Interleukin 18	Protein Coding	Q14116	48	GC11M11214 3	14.9500 4	https://www.genecards.org/cgi-bin/carddisp.pl?gene=IL18
MIR27B	MicroRNA 27b	RNA Gene		22	GC09P096188	14.7975	https://www.genecards.org/cgi-bin/carddisp.pl?gene=MIR27B
IGFBP3	Insulin Like Growth Factor Binding Protein 3	Protein Coding	P17936	51	GC07M04591 2	14.7942 7	https://www.genecards.org/cgi-bin/carddisp.pl?gene=IGFBP3
SNHG14	Small Nucleolar RNA Host Gene 14	RNA Gene		19	GC15P056584	14.7504 3	https://www.genecards.org/cgi-bin/carddisp.pl?gene=SNHG14

GPT	Glutamic--Pyruvic Transaminase	Protein Coding	P24298	46	GC08P144502	14.68955	https://www.genecards.org/cgi-bin/carddisp.pl?gene=GPT
F2	Coagulation Factor II, Thrombin	Protein Coding	P00734	54	GC11P047030	14.60334	https://www.genecards.org/cgi-bin/carddisp.pl?gene=F2
ENSG00000276919		RNA Gene		9	GC14P113028	14.59099	https://www.genecards.org/cgi-bin/carddisp.pl?gene=ENSG00000276919
CXCL8	C-X-C Motif Chemokine Ligand 8	Protein Coding	P10145	48	GC04P073740	14.45553	https://www.genecards.org/cgi-bin/carddisp.pl?gene=CXCL8
TRAPPC9	Trafficking Protein Particle Complex Subunit 9	Protein Coding	Q96Q05	44	GC08M139728	14.44374	https://www.genecards.org/cgi-bin/carddisp.pl?gene=TRAPPC9
RARRES2	Retinoic Acid Receptor Responder 2	Protein Coding	Q99969	44	GC07M150333	14.37283	https://www.genecards.org/cgi-bin/carddisp.pl?gene=RARRES2
TCF7L2	Transcription Factor 7 Like 2	Protein Coding	Q9NQB0	51	GC10P112950	14.33788	https://www.genecards.org/cgi-bin/carddisp.pl?gene=TCF7L2
IFNG	Interferon Gamma	Protein Coding	P01579	54	GC12M068154	14.32376	https://www.genecards.org/cgi-bin/carddisp.pl?gene=IFNG
PAPPA	Pappalysin 1	Protein Coding	Q13219	46	GC09P121616	14.31222	https://www.genecards.org/cgi-bin/carddisp.pl?gene=PAPPA
HLA-G	Major Histocompatibility Complex, Class I, G	Protein Coding	P17693	47	GC06P111943	14.19074	https://www.genecards.org/cgi-bin/carddisp.pl?gene=HLA-G
BBS10	Bardet-Biedl Syndrome 10	Protein Coding	Q8TAM1	45	GC12M076344	14.09896	https://www.genecards.org/cgi-bin/carddisp.pl?gene=BBS10
NLRP5	NLR Family Pyrin Domain Containing 5	Protein Coding	P59047	42	GC19P090224	13.96716	https://www.genecards.org/cgi-bin/carddisp.pl?gene=NLRP5

PWRN1	Prader-Willi Region Non-Protein Coding RNA 1	RNA Gene		20	GC15P059930	13.9466	https://www.genecards.org/cgi-bin/carddisp.pl?gene=PWRN1
IRS2	Insulin Receptor Substrate 2	Protein Coding	Q9Y4H2	48	GC13M109752	13.92813	https://www.genecards.org/cgi-bin/carddisp.pl?gene=IRS2
SREBF1	Sterol Regulatory Element Binding Transcription Factor 1	Protein Coding	P36956	52	GC17M017810	13.92161	https://www.genecards.org/cgi-bin/carddisp.pl?gene=SREBF1
ICAM1	Intercellular Adhesion Molecule 1	Protein Coding	P05362	54	GC19P088778	13.85501	https://www.genecards.org/cgi-bin/carddisp.pl?gene=ICAM1
EDN1	Endothelin 1	Protein Coding	P05305	53	GC06P012236	13.84491	https://www.genecards.org/cgi-bin/carddisp.pl?gene=EDN1
APOA1	Apolipoprotein A1	Protein Coding	P02647	54	GC11M116835	13.82141	https://www.genecards.org/cgi-bin/carddisp.pl?gene=APOA1
MMP9	Matrix Metallopeptidase 9	Protein Coding	P14780	57	GC20P046008	13.77961	https://www.genecards.org/cgi-bin/carddisp.pl?gene=MMP9
CDKN1C	Cyclin Dependent Kinase Inhibitor 1C	Protein Coding	P49918	49	GC11M009036	13.75148	https://www.genecards.org/cgi-bin/carddisp.pl?gene=CDKN1C
MT-TE	Mitochondrially Encoded TRNA-Glu (GAA/G)	RNA Gene		13	GCMTM014676	13.72492	https://www.genecards.org/cgi-bin/carddisp.pl?gene=MT-TE
SLC6A4	Solute Carrier Family 6 Member 4	Protein Coding	P31645	51	GC17M030194	13.65821	https://www.genecards.org/cgi-bin/carddisp.pl?gene=SLC6A4
GSTM1	Glutathione S-Transferase Mu 1	Protein Coding	P09488	46	GC01P109687	13.63768	https://www.genecards.org/cgi-bin/carddisp.pl?gene=GSTM1
CGA	Glycoprotein Hormones, Alpha Polypeptide	Protein Coding	P01215	50	GC06M087085	13.58	https://www.genecards.org/cgi-bin/carddisp.pl?gene=CGA

CTNNB1	Catenin Beta 1	Protein Coding	P35222	57	GC03P041194	13.47028	https://www.genecards.org/cgi-bin/carddisp.pl?gene=CTNNB1
AKT1	AKT Serine/Threonine Kinase 1	Protein Coding	P31749	57	GC14M104769	13.46262	https://www.genecards.org/cgi-bin/carddisp.pl?gene=AKT1
CERNA3	Competing Endogenous LncRNA 3 For MiR-645	RNA Gene		13	GC08P056092	13.45987	https://www.genecards.org/cgi-bin/carddisp.pl?gene=CERNA3
MIR125A	MicroRNA 125a	RNA Gene		22	GC19P090041	13.44672	https://www.genecards.org/cgi-bin/carddisp.pl?gene=MIR125A
MT-ND6	Mitochondrially Encoded NADH:Ubiquinone Oxidoreductase Core Subunit 6	Protein Coding	P03923	35	GCMTM014151	13.43169	https://www.genecards.org/cgi-bin/carddisp.pl?gene=MT-ND6
FGFR1	Fibroblast Growth Factor Receptor 1	Protein Coding	P11362	59	GC08M038400	13.34153	https://www.genecards.org/cgi-bin/carddisp.pl?gene=FGFR1
MT-ND2	Mitochondrially Encoded NADH:Ubiquinone Oxidoreductase Core Subunit 2	Protein Coding	P03891	38	GCMTP004472	13.34006	https://www.genecards.org/cgi-bin/carddisp.pl?gene=MT-ND2
ESR1	Estrogen Receptor 1	Protein Coding	P03372	58	GC06P151656	13.27585	https://www.genecards.org/cgi-bin/carddisp.pl?gene=ESR1
BBS7	Bardet-Biedl Syndrome 7	Protein Coding	Q8IWZ6	43	GC04M121824	13.18027	https://www.genecards.org/cgi-bin/carddisp.pl?gene=BBS7
TLR4	Toll Like Receptor 4	Protein Coding	O00206	55	GC09P117704	13.05175	https://www.genecards.org/cgi-bin/carddisp.pl?gene=TLR4
MT-ND5	Mitochondrially Encoded NADH:Ubiquinone Oxidoreductase Core Subunit 5	Protein Coding	P03915	37	GCMTP012339	13.02516	https://www.genecards.org/cgi-bin/carddisp.pl?gene=MT-ND5

CHKB-CPT1B	CHKB-CPT1B Readthrough (NMD Candidate)	RNA Gene		19	GC22M050568	12.95258	https://www.genecards.org/cgi-bin/carddisp.pl?gene=CHKB-CPT1B
XIST	X Inactive Specific Transcript	RNA Gene		24	GC0XM073820	12.92152	https://www.genecards.org/cgi-bin/carddisp.pl?gene=XIST
HSD11B2	Hydroxysteroid 11-Beta Dehydrogenase 2	Protein Coding	P80365	49	GC16P067433	12.9007	https://www.genecards.org/cgi-bin/carddisp.pl?gene=HSD11B2
DRD2	Dopamine Receptor D2	Protein Coding	P14416	53	GC11M113409	12.82816	https://www.genecards.org/cgi-bin/carddisp.pl?gene=DRD2
LINC02605	Long Intergenic Non-Protein Coding RNA 2605	RNA Gene		13	GC08P078838	12.74423	https://www.genecards.org/cgi-bin/carddisp.pl?gene=LINC02605
MT-ND4	Mitochondrially Encoded NADH:Ubiquinone Oxidoreductase Core Subunit 4	Protein Coding	P03905	35	GCMTP010762	12.74281	https://www.genecards.org/cgi-bin/carddisp.pl?gene=MT-ND4
DRD4	Dopamine Receptor D4	Protein Coding	P21917	49	GC11P004458	12.70188	https://www.genecards.org/cgi-bin/carddisp.pl?gene=DRD4
AFP	Alpha Fetoprotein	Protein Coding	P02771	51	GC04P073431	12.67694	https://www.genecards.org/cgi-bin/carddisp.pl?gene=AFP
PGR-AS1	PGR Antisense RNA 1	RNA Gene		11	GC11P101131	12.63347	https://www.genecards.org/cgi-bin/carddisp.pl?gene=PGR-AS1
PTPN1	Protein Tyrosine Phosphatase Non-Receptor Type 1	Protein Coding	P18031	54	GC20P050510	12.57398	https://www.genecards.org/cgi-bin/carddisp.pl?gene=PTPN1
PNPLA3	Patatin Like Phospholipase Domain Containing 3	Protein Coding	Q9NST1	46	GC22P043923	12.51208	https://www.genecards.org/cgi-bin/carddisp.pl?gene=PNPLA3
MT-TP	Mitochondrially Encoded TRNA-Pro (CCN)	RNA Gene		11	GCMTM015957	12.39883	https://www.genecards.org/cgi-bin/carddisp.pl?gene=MT-TP

F5	Coagulation Factor V	Protein Coding	P12259	51	GC01M169511	12.30501	https://www.genecards.org/cgi-bin/carddisp.pl?gene=F5
SNORD116-1	Small Nucleolar RNA, C/D Box 116-1	RNA Gene		16	GC15P056593	12.24378	https://www.genecards.org/cgi-bin/carddisp.pl?gene=SNORD116-1
FGF21	Fibroblast Growth Factor 21	Protein Coding	Q9NSA1	43	GC19P089879	12.22424	https://www.genecards.org/cgi-bin/carddisp.pl?gene=FGF21
STAT3	Signal Transducer And Activator Of Transcription 3	Protein Coding	P40763	58	GC17M042313	12.22073	https://www.genecards.org/cgi-bin/carddisp.pl?gene=STAT3
PGF	Placental Growth Factor	Protein Coding	P49763	48	GC14M074941	12.21047	https://www.genecards.org/cgi-bin/carddisp.pl?gene=PGF
SLC6A3	Solute Carrier Family 6 Member 3	Protein Coding	Q01959	54	GC05M001392	12.14725	https://www.genecards.org/cgi-bin/carddisp.pl?gene=SLC6A3
MEG8	Maternally Expressed 8, Small Nucleolar RNA Host Gene	RNA Gene		21	GC14P113577	12.12432	https://www.genecards.org/cgi-bin/carddisp.pl?gene=MEG8
ADIPOR1	Adiponectin Receptor 1	Protein Coding	Q96A54	47	GC01M202940	12.08464	https://www.genecards.org/cgi-bin/carddisp.pl?gene=ADIPOR1
CPE	Carboxypeptidase E	Protein Coding	P16870	52	GC04P165361	12.07212	https://www.genecards.org/cgi-bin/carddisp.pl?gene=CPE
APOA5	Apolipoprotein A5	Protein Coding	Q6Q788	47	GC11M116789	12.06424	https://www.genecards.org/cgi-bin/carddisp.pl?gene=APOA5
CBS	Cystathionine Beta-Synthase	Protein Coding	P35520	54	GC21M043053	12.04462	https://www.genecards.org/cgi-bin/carddisp.pl?gene=CBS
SNORD115-1	Small Nucleolar RNA, C/D Box 115-1	RNA Gene		15	GC15P056601	12.03839	https://www.genecards.org/cgi-bin/carddisp.pl?gene=SNORD115-1

PLIN1	Perilipin 1	Protein Coding	O60240	51	GC15M089664	11.99052	https://www.genecards.org/cgi-bin/carddisp.pl?gene=PLIN1
CGB3	Chorionic Gonadotropin Subunit Beta 3	Protein Coding	P0DN86	38	GC19M082846	11.97741	https://www.genecards.org/cgi-bin/carddisp.pl?gene=CGB3
MT-TS1	Mitochondrially Encoded TRNA-Ser (UCN) 1	RNA Gene		16	GCMTM007447	11.97148	https://www.genecards.org/cgi-bin/carddisp.pl?gene=MT-TS1
MT-TW	Mitochondrially Encoded TRNA-Trp (UGA/G)	RNA Gene		15	GCMTP005514	11.96417	https://www.genecards.org/cgi-bin/carddisp.pl?gene=MT-TW
CETP	Cholesteryl Ester Transfer Protein	Protein Coding	P11597	50	GC16P056961	11.94951	https://www.genecards.org/cgi-bin/carddisp.pl?gene=CETP
REN	Renin	Protein Coding	P00797	53	GC01M204154	11.93156	https://www.genecards.org/cgi-bin/carddisp.pl?gene=REN
FASN	Fatty Acid Synthase	Protein Coding	P49327	53	GC17M082078	11.91311	https://www.genecards.org/cgi-bin/carddisp.pl?gene=FASN
MT-ND3	Mitochondrially Encoded NADH:Ubiquinone Oxidoreductase Core Subunit 3	Protein Coding	P03897	36	GCMTP010061	11.84316	https://www.genecards.org/cgi-bin/carddisp.pl?gene=MT-ND3
BGLAP	Bone Gamma-Carboxyglutamate Protein	Protein Coding	P02818	44	GC01P156242	11.82324	https://www.genecards.org/cgi-bin/carddisp.pl?gene=BGLAP
TMEM18	Transmembrane Protein 18	Protein Coding	Q96B42	39	GC02M000660	11.7838	https://www.genecards.org/cgi-bin/carddisp.pl?gene=TMEM18
GHSR	Growth Hormone Secretagogue Receptor	Protein Coding	Q92847	50	GC03M172443	11.77873	https://www.genecards.org/cgi-bin/carddisp.pl?gene=GHSR
HERC2	HECT And RLD Domain Containing E3 Ubiquitin Protein Ligase 2	Protein Coding	O95714	48	GC15M028111	11.76594	https://www.genecards.org/cgi-bin/carddisp.pl?gene=HERC2

FABP2	Fatty Acid Binding Protein 2	Protein Coding	P12104	45	GC04M119317	11.76406	https://www.genecards.org/cgi-bin/carddisp.pl?gene=FABP2
CD36	CD36 Molecule	Protein Coding	P16671	53	GC07P080369	11.74412	https://www.genecards.org/cgi-bin/carddisp.pl?gene=CD36
ITLN1	Intelectin 1	Protein Coding	Q8WWA0	43	GC01M160876	11.73931	https://www.genecards.org/cgi-bin/carddisp.pl?gene=ITLN1
TLR2	Toll Like Receptor 2	Protein Coding	O60603	55	GC04P153684	11.70844	https://www.genecards.org/cgi-bin/carddisp.pl?gene=TLR2
FNDC5	Fibronectin Type III Domain Containing 5	Protein Coding	Q8NAU1	39	GC01M033726	11.64519	https://www.genecards.org/cgi-bin/carddisp.pl?gene=FNDC5
LMNA	Lamin A/C	Protein Coding	P02545	54	GC01P156082	11.55861	https://www.genecards.org/cgi-bin/carddisp.pl?gene=LMNA
MT-TV	Mitochondrially Encoded TRNA-Val (GUN)	RNA Gene		16	GCMTP001605	11.55434	https://www.genecards.org/cgi-bin/carddisp.pl?gene=MT-TV
C3	Complement C3	Protein Coding	P01024	53	GC19M006677	11.49132	https://www.genecards.org/cgi-bin/carddisp.pl?gene=C3
IGFBP2	Insulin Like Growth Factor Binding Protein 2	Protein Coding	P18065	47	GC02P216632	11.47481	https://www.genecards.org/cgi-bin/carddisp.pl?gene=IGFBP2
VDR	Vitamin D Receptor	Protein Coding	P11473	54	GC12M047841	11.46424	https://www.genecards.org/cgi-bin/carddisp.pl?gene=VDR
APLN	Apelin	Protein Coding	Q9ULZ1	41	GC0XM129645	11.4502	https://www.genecards.org/cgi-bin/carddisp.pl?gene=APLN
SOCS3	Suppressor Of Cytokine Signaling 3	Protein Coding	O14543	47	GC17M078356	11.44409	https://www.genecards.org/cgi-bin/carddisp.pl?gene=SOCS3

WT1	WT1 Transcription Factor	Protein Coding	P19544	52	GC11M032365	11.42321	https://www.genecards.org/cgi-bin/carddisp.pl?gene=WT1
PPARD	Peroxisome Proliferator Activated Receptor Delta	Protein Coding	Q03181	50	GC06P112139	11.35766	https://www.genecards.org/cgi-bin/carddisp.pl?gene=PPARD
COL1A1	Collagen Type I Alpha 1 Chain	Protein Coding	P02452	53	GC17M064687	11.35283	https://www.genecards.org/cgi-bin/carddisp.pl?gene=COL1A1
CAPN10	Calpain 10	Protein Coding	Q9HC96	44	GC02P240586	11.3489	https://www.genecards.org/cgi-bin/carddisp.pl?gene=CAPN10
HDAC8	Histone Deacetylase 8	Protein Coding	Q9BY41	52	GC0XM072329	11.32704	https://www.genecards.org/cgi-bin/carddisp.pl?gene=HDAC8
ADIPOR2	Adiponectin Receptor 2	Protein Coding	Q86V24	45	GC12P001670	11.31074	https://www.genecards.org/cgi-bin/carddisp.pl?gene=ADIPOR2
GHRH	Growth Hormone Releasing Hormone	Protein Coding	P01286	42	GC20M037251	11.30512	https://www.genecards.org/cgi-bin/carddisp.pl?gene=GHRH
MTOR	Mechanistic Target Of Rapamycin Kinase	Protein Coding	P42345	59	GC01M011106	11.30073	https://www.genecards.org/cgi-bin/carddisp.pl?gene=MTOR
SLC17A5	Solute Carrier Family 17 Member 5	Protein Coding	Q9NRA2	48	GC06M073593	11.28125	https://www.genecards.org/cgi-bin/carddisp.pl?gene=SLC17A5
SCD	Stearoyl-CoA Desaturase	Protein Coding	O00767	53	GC10P100347	11.21815	https://www.genecards.org/cgi-bin/carddisp.pl?gene=SCD
EIF2S3	Eukaryotic Translation Initiation Factor 2 Subunit Gamma	Protein Coding	P41091	50	GC0XP024054	11.21439	https://www.genecards.org/cgi-bin/carddisp.pl?gene=EIF2S3
APOC3	Apolipoprotein C3	Protein Coding	P02656	49	GC11P116829	11.1905	https://www.genecards.org/cgi-bin/carddisp.pl?gene=APOC3

AGTR1	Angiotensin II Receptor Type 1	Protein Coding	P30556	55	GC03P148697	11.14232	https://www.genecards.org/cgi-bin/carddisp.pl?gene=AGTR1
MIR21	MicroRNA 21	RNA Gene		25	GC17P086838	11.11288	https://www.genecards.org/cgi-bin/carddisp.pl?gene=MIR21
OXTR	Oxytocin Receptor	Protein Coding	P30559	51	GC03M008767	10.92819	https://www.genecards.org/cgi-bin/carddisp.pl?gene=OXTR
TMX2-CTNND1	TMX2-CTNND1 Readthrough (NMD Candidate)	RNA Gene		17	GC11P057712	10.89342	https://www.genecards.org/cgi-bin/carddisp.pl?gene=TMX2-CTNND1
PNPLA2	Patatin Like Phospholipase Domain Containing 2	Protein Coding	Q96AD5	48	GC11P004473	10.89113	https://www.genecards.org/cgi-bin/carddisp.pl?gene=PNPLA2
MT-RNR1	Mitochondrially Encoded 12S RRNA	RNA Gene	A0A0C5B5G6	17	GCMTP000642	10.87583	https://www.genecards.org/cgi-bin/carddisp.pl?gene=MT-RNR1
BBS12	Bardet-Biedl Syndrome 12	Protein Coding	Q6ZW61	39	GC04P122702	10.86555	https://www.genecards.org/cgi-bin/carddisp.pl?gene=BBS12
MKRN3-AS1	MKRN3 Antisense RNA 1	RNA Gene		6	GC15U901326	10.85435	https://www.genecards.org/cgi-bin/carddisp.pl?gene=MKRN3-AS1
FABP4	Fatty Acid Binding Protein 4	Protein Coding	P15090	47	GC08M081478	10.84122	https://www.genecards.org/cgi-bin/carddisp.pl?gene=FABP4
MFN2	Mitofusin 2	Protein Coding	O95140	52	GC01P011980	10.82184	https://www.genecards.org/cgi-bin/carddisp.pl?gene=MFN2
TGFB1	Transforming Growth Factor Beta 1	Protein Coding	P01137	57	GC19M041301	10.70702	https://www.genecards.org/cgi-bin/carddisp.pl?gene=TGFB1
MECP2	Methyl-CpG Binding Protein 2	Protein Coding	P51608	51	GC0XM154021	10.6109	https://www.genecards.org/cgi-bin/carddisp.pl?gene=MECP2

MIR126	MicroRNA 126	RNA Gene		23	GC09P136670	10.5505 2	https://www.genecards.org/cgi-bin/carddisp.pl?gene=MIR126
CCL5	C-C Motif Chemokine Ligand 5	Protein Coding	P13501	48	GC17M03587 1	10.4242 5	https://www.genecards.org/cgi-bin/carddisp.pl?gene=CCL5
SIRT1	Sirtuin 1	Protein Coding	Q96EB6	53	GC10P067884	10.4096 2	https://www.genecards.org/cgi-bin/carddisp.pl?gene=SIRT1
FMR1	Fragile X Messenger Ribonucleoprotein 1	Protein Coding	Q06787	48	GC0XP14797 2	10.3960 5	https://www.genecards.org/cgi-bin/carddisp.pl?gene=FMR1
INSIG2	Insulin Induced Gene 2	Protein Coding	Q9Y5U4	43	GC02P118088	10.3852 3	https://www.genecards.org/cgi-bin/carddisp.pl?gene=INSIG2
MT-CO1	Mitochondrially Encoded Cytochrome C Oxidase I	Protein Coding	P00395	36	GCMTP00590 6	10.3548 6	https://www.genecards.org/cgi-bin/carddisp.pl?gene=MT-CO1
HNF1B	HNF1 Homeobox B	Protein Coding	P35680	47	GC17M03768 6	10.3538 8	https://www.genecards.org/cgi-bin/carddisp.pl?gene=HNF1B
MIR146B	MicroRNA 146b	RNA Gene		22	GC10P102436	10.3432 3	https://www.genecards.org/cgi-bin/carddisp.pl?gene=MIR146B
ABCA1	ATP Binding Cassette Subfamily A Member 1	Protein Coding	O95477	52	GC09M10478 1	10.3422	https://www.genecards.org/cgi-bin/carddisp.pl?gene=ABCA1
KCNQ1	Potassium Voltage-Gated Channel Subfamily Q Member 1	Protein Coding	P51787	53	GC11P002444	10.3135 3	https://www.genecards.org/cgi-bin/carddisp.pl?gene=KCNQ1
SNORD15A	Small Nucleolar RNA, C/D Box 15A	RNA Gene		17	GC11P075400	10.3102 9	https://www.genecards.org/cgi-bin/carddisp.pl?gene=SNORD15A
HTR2A	5-Hydroxytryptamine Receptor 2A	Protein Coding	P28223	51	GC13M04683 1	10.3054 4	https://www.genecards.org/cgi-bin/carddisp.pl?gene=HTR2A

MIR140	MicroRNA 140	RNA Gene		25	GC16P069934	10.2542 9	https://www.genecards.org/cgi-bin/carddisp.pl?gene=MIR140
PRKAR1A	Protein Kinase CAMP-Dependent Type I Regulatory Subunit Alpha	Protein Coding	P10644	55	GC17P087070	10.2342 2	https://www.genecards.org/cgi-bin/carddisp.pl?gene=PRKAR1A
CYP2E1	Cytochrome P450 Family 2 Subfamily E Member 1	Protein Coding	P05181	50	GC10P133520	10.2248 8	https://www.genecards.org/cgi-bin/carddisp.pl?gene=CYP2E1
IAPP	Islet Amyloid Polypeptide	Protein Coding	P10997	44	GC12P021354	10.1627 1	https://www.genecards.org/cgi-bin/carddisp.pl?gene=IAPP
DPP4	Dipeptidyl Peptidase 4	Protein Coding	P27487	53	GC02M16199 2	10.1404 9	https://www.genecards.org/cgi-bin/carddisp.pl?gene=DPP4
IL6R	Interleukin 6 Receptor	Protein Coding	P08887	54	GC01P154405	10.1381 7	https://www.genecards.org/cgi-bin/carddisp.pl?gene=IL6R
LCN2	Lipocalin 2	Protein Coding	P80188	49	GC09P128149	10.1195 9	https://www.genecards.org/cgi-bin/carddisp.pl?gene=LCN2
SHH	Sonic Hedgehog Signaling Molecule	Protein Coding	Q15465	54	GC07M15579 9	10.1178 7	https://www.genecards.org/cgi-bin/carddisp.pl?gene=SHH
PLAGL1	PLAG1 Like Zinc Finger 1	Protein Coding	Q9UM63	46	GC06M14394 0	10.1032 5	https://www.genecards.org/cgi-bin/carddisp.pl?gene=PLAGL1
CGB5	Chorionic Gonadotropin Subunit Beta 5	Protein Coding	P0DN86	35	GC19P049043	10.0332 7	https://www.genecards.org/cgi-bin/carddisp.pl?gene=CGB5
AHSG	Alpha 2-HS Glycoprotein	Protein Coding	P02765	48	GC03P186658	10.0319 5	https://www.genecards.org/cgi-bin/carddisp.pl?gene=AHSG
IL4	Interleukin 4	Protein Coding	P05112	51	GC05P132673	10.0209 6	https://www.genecards.org/cgi-bin/carddisp.pl?gene=IL4

GGT1	Gamma-Glutamyltransferase 1	Protein Coding	P19440	53	GC22P024583	10.0132 1	https://www.genecards.org/cgi-bin/carddisp.pl?gene=GGT1
HTR2C	5-Hydroxytryptamine Receptor 2C	Protein Coding	P28335	48	GC0XP11458 4	9.98122 5	https://www.genecards.org/cgi-bin/carddisp.pl?gene=HTR2C
NMB	Neuromedin B	Protein Coding	P08949	44	GC15M08465 5	9.90971 4	https://www.genecards.org/cgi-bin/carddisp.pl?gene=NMB
SELE	Selectin E	Protein Coding	P16581	47	GC01M16972 2	9.87749 8	https://www.genecards.org/cgi-bin/carddisp.pl?gene=SELE
IL1A	Interleukin 1 Alpha	Protein Coding	P01583	50	GC02M11277 3	9.79131 7	https://www.genecards.org/cgi-bin/carddisp.pl?gene=IL1A
CYP1A1	Cytochrome P450 Family 1 Subfamily A Member 1	Protein Coding	P04798	51	GC15M07471 9	9.78262 2	https://www.genecards.org/cgi-bin/carddisp.pl?gene=CYP1A1
OOEP	Oocyte Expressed Protein	Protein Coding	A6NGQ2	33	GC06M07336 8	9.77828 3	https://www.genecards.org/cgi-bin/carddisp.pl?gene=OOEP
GNRH1	Gonadotropin Releasing Hormone 1	Protein Coding	P01148	45	GC08M02541 9	9.76536 4	https://www.genecards.org/cgi-bin/carddisp.pl?gene=GNRH1
LAS1L	LAS1 Like Ribosome Biogenesis Factor	Protein Coding	Q9Y4W2	42	GC0XM06544 0	9.73753 4	https://www.genecards.org/cgi-bin/carddisp.pl?gene=LAS1L
NPPA	Natriuretic Peptide A	Protein Coding	P01160	49	GC01M01184 6	9.72327 1	https://www.genecards.org/cgi-bin/carddisp.pl?gene=NPPA
MIR223	MicroRNA 223	RNA Gene		22	GC0XP06601 8	9.66927 1	https://www.genecards.org/cgi-bin/carddisp.pl?gene=MIR223
CLOCK	Clock Circadian Regulator	Protein Coding	O15516	48	GC04M05542 7	9.64640 7	https://www.genecards.org/cgi-bin/carddisp.pl?gene=CLOCK

TNFRSF1B	TNF Receptor Superfamily Member 1B	Protein Coding	P20333	53	GC01P012471	9.641573	https://www.genecards.org/cgi-bin/carddisp.pl?gene=TNFRSF1B
MTRR	5-Methyltetrahydrofolate-Homocysteine Methyltransferase Reductase	Protein Coding	Q9UBK8	48	GC05P007851	9.637688	https://www.genecards.org/cgi-bin/carddisp.pl?gene=MTRR
PIK3CA	Phosphatidylinositol-4,5-Bisphosphate 3-Kinase Catalytic Subunit Alpha	Protein Coding	P42336	56	GC03P179148	9.612315	https://www.genecards.org/cgi-bin/carddisp.pl?gene=PIK3CA
SLC2A1	Solute Carrier Family 2 Member 1	Protein Coding	P11166	57	GC01M042925	9.564754	https://www.genecards.org/cgi-bin/carddisp.pl?gene=SLC2A1
MME	Membrane Metalloendopeptidase	Protein Coding	P08473	54	GC03P155024	9.554605	https://www.genecards.org/cgi-bin/carddisp.pl?gene=MME
GSTT1	Glutathione S-Transferase Theta 1	Protein Coding	P30711	36	GC22Mi00270	9.478709	https://www.genecards.org/cgi-bin/carddisp.pl?gene=GSTT1
HIF1A	Hypoxia Inducible Factor 1 Subunit Alpha	Protein Coding	Q16665	52	GC14P061695	9.469829	https://www.genecards.org/cgi-bin/carddisp.pl?gene=HIF1A
RYR1	Ryanodine Receptor 1	Protein Coding	P21817	52	GC19P089493	9.451361	https://www.genecards.org/cgi-bin/carddisp.pl?gene=RYR1
CREBBP	CREB Binding Protein	Protein Coding	Q92793	58	GC16M013493	9.425735	https://www.genecards.org/cgi-bin/carddisp.pl?gene=CREBBP
AR	Androgen Receptor	Protein Coding	P10275	55	GC0XP067544	9.425255	https://www.genecards.org/cgi-bin/carddisp.pl?gene=AR
ITGB3	Integrin Subunit Beta 3	Protein Coding	P05106	56	GC17P086612	9.42301	https://www.genecards.org/cgi-bin/carddisp.pl?gene=ITGB3
PDX1	Pancreatic And Duodenal Homeobox 1	Protein Coding	P52945	50	GC13P027921	9.357768	https://www.genecards.org/cgi-bin/carddisp.pl?gene=PDX1

GSTP1	Glutathione S-Transferase Pi 1	Protein Coding	P09211	54	GC11P067583	9.345621	https://www.genecards.org/cgi-bin/carddisp.pl?gene=GSTP1
ACP1	Acid Phosphatase 1	Protein Coding	P24666	48	GC02P000541	9.343059	https://www.genecards.org/cgi-bin/carddisp.pl?gene=ACP1
GAD2	Glutamate Decarboxylase 2	Protein Coding	Q05329	48	GC10P026216	9.342784	https://www.genecards.org/cgi-bin/carddisp.pl?gene=GAD2
ZFP57	ZFP57 Zinc Finger Protein	Protein Coding	Q9NU63	42	GC06M029672	9.315833	https://www.genecards.org/cgi-bin/carddisp.pl?gene=ZFP57
MALAT1	Metastasis Associated Lung Adenocarcinoma Transcript 1	RNA Gene		25	GC11P079854	9.315371	https://www.genecards.org/cgi-bin/carddisp.pl?gene=MALAT1
LOC110806262	Solute Carrier Family 6 Member 4 Gene Promoter	Functional Element		3	GC17P030235	9.309656	https://www.genecards.org/cgi-bin/carddisp.pl?gene=LOC110806262
IGF1R	Insulin Like Growth Factor 1 Receptor	Protein Coding	P08069	58	GC15P098648	9.300042	https://www.genecards.org/cgi-bin/carddisp.pl?gene=IGF1R
CEP290	Centrosomal Protein 290	Protein Coding	O15078	46	GC12M088049	9.274328	https://www.genecards.org/cgi-bin/carddisp.pl?gene=CEP290
PTEN	Phosphatase And Tensin Homolog	Protein Coding	P60484	55	GC10P100942	9.238496	https://www.genecards.org/cgi-bin/carddisp.pl?gene=PTEN
ATRX	ATRX Chromatin Remodeler	Protein Coding	P46100	51	GC0XM077504	9.210809	https://www.genecards.org/cgi-bin/carddisp.pl?gene=ATRX
FAAH	Fatty Acid Amide Hydrolase	Protein Coding	O00519	51	GC01P046394	9.209714	https://www.genecards.org/cgi-bin/carddisp.pl?gene=FAAH
IL15	Interleukin 15	Protein Coding	P40933	47	GC04P141636	9.184642	https://www.genecards.org/cgi-bin/carddisp.pl?gene=IL15

TIMP1	TIMP Metallopeptidase Inhibitor 1	Protein Coding	P01033	49	GC0XP052432	9.183694	https://www.genecards.org/cgi-bin/carddisp.pl?gene=TIMP1
MAOA	Monoamine Oxidase A	Protein Coding	P21397	53	GC0XP043654	9.161522	https://www.genecards.org/cgi-bin/carddisp.pl?gene=MAOA
MIR34A	MicroRNA 34a	RNA Gene		23	GC01M010872	9.135346	https://www.genecards.org/cgi-bin/carddisp.pl?gene=MIR34A
GAL	Galanin And GMAP Prepropeptide	Protein Coding	P22466	48	GC11P080053	9.117929	https://www.genecards.org/cgi-bin/carddisp.pl?gene=GAL
NFE2L2	NFE2 Like BZIP Transcription Factor 2	Protein Coding	Q16236	55	GC02M177227	9.109505	https://www.genecards.org/cgi-bin/carddisp.pl?gene=NFE2L2
VCAM1	Vascular Cell Adhesion Molecule 1	Protein Coding	P19320	50	GC01P100719	9.108341	https://www.genecards.org/cgi-bin/carddisp.pl?gene=VCAM1
GNAS-AS1	GNAS Antisense RNA 1	RNA Gene		25	GC20M058811	9.04405	https://www.genecards.org/cgi-bin/carddisp.pl?gene=GNAS-AS1
PAX6	Paired Box 6	Protein Coding	P26367	52	GC11M031784	9.04118	https://www.genecards.org/cgi-bin/carddisp.pl?gene=PAX6
SOD2	Superoxide Dismutase 2	Protein Coding	P04179	51	GC06M159669	9.038123	https://www.genecards.org/cgi-bin/carddisp.pl?gene=SOD2
PRDM16	PR/SET Domain 16	Protein Coding	Q9HAZ2	49	GC01P006147	9.035675	https://www.genecards.org/cgi-bin/carddisp.pl?gene=PRDM16
GAPDH	Glyceraldehyde-3-Phosphate Dehydrogenase	Protein Coding	P04406	54	GC12P030471	9.029325	https://www.genecards.org/cgi-bin/carddisp.pl?gene=GAPDH
STOX1	Storkhead Box 1	Protein Coding	Q6ZVD7	42	GC10P068827	9.012944	https://www.genecards.org/cgi-bin/carddisp.pl?gene=STOX1

AKT2	AKT Serine/Threonine Kinase 2	Protein Coding	P31751	58	GC19M040230	9.00355	https://www.genecards.org/cgi-bin/carddisp.pl?gene=AKT2
GNPDA2	Glucosamine-6-Phosphate Deaminase 2	Protein Coding	Q8TDQ7	43	GC04M044684	8.972185	https://www.genecards.org/cgi-bin/carddisp.pl?gene=GNPDA2
GIP	Gastric Inhibitory Polypeptide	Protein Coding	P09681	41	GC17M048958	8.960449	https://www.genecards.org/cgi-bin/carddisp.pl?gene=GIP
NR1H2	Nuclear Receptor Subfamily 1 Group H Member 2	Protein Coding	P55055	50	GC19P050329	8.955606	https://www.genecards.org/cgi-bin/carddisp.pl?gene=NR1H2
LIF	LIF Interleukin 6 Family Cytokine	Protein Coding	P15018	48	GC22M030240	8.934807	https://www.genecards.org/cgi-bin/carddisp.pl?gene=LIF
CGB7	Chorionic Gonadotropin Subunit Beta 7	Protein Coding	P0DN87	35	GC19M049054	8.933521	https://www.genecards.org/cgi-bin/carddisp.pl?gene=CGB7
SERPINA12	Serpin Family A Member 12	Protein Coding	Q8IW75	44	GC14M094487	8.930112	https://www.genecards.org/cgi-bin/carddisp.pl?gene=SERPINA12
NLRP7	NLR Family Pyrin Domain Containing 7	Protein Coding	Q8WX94	46	GC19M054923	8.917601	https://www.genecards.org/cgi-bin/carddisp.pl?gene=NLRP7
MIR155	MicroRNA 155	RNA Gene		22	GC21P025573	8.883368	https://www.genecards.org/cgi-bin/carddisp.pl?gene=MIR155
MEST	Mesoderm Specific Transcript	Protein Coding	Q5EB52	44	GC07P130486	8.853587	https://www.genecards.org/cgi-bin/carddisp.pl?gene=MEST
IGF2R	Insulin Like Growth Factor 2 Receptor	Protein Coding	P11717	50	GC06P159969	8.843941	https://www.genecards.org/cgi-bin/carddisp.pl?gene=IGF2R
FST	Follistatin	Protein Coding	P19883	52	GC05P053480	8.842514	https://www.genecards.org/cgi-bin/carddisp.pl?gene=FST

CDKN2A	Cyclin Dependent Kinase Inhibitor 2A	Protein Coding	Q8N726	55	GC09M021967	8.840081	https://www.genecards.org/cgi-bin/carddisp.pl?gene=CDKN2A
MIR146A	MicroRNA 146a	RNA Gene		23	GC05P160485	8.818419	https://www.genecards.org/cgi-bin/carddisp.pl?gene=MIR146A
FADS1	Fatty Acid Desaturase 1	Protein Coding	O60427	46	GC11M061799	8.809001	https://www.genecards.org/cgi-bin/carddisp.pl?gene=FADS1
IL17A	Interleukin 17A	Protein Coding	Q16552	47	GC06P052186	8.799455	https://www.genecards.org/cgi-bin/carddisp.pl?gene=IL17A
H6PD	Hexose-6-Phosphate Dehydrogenase/Glucose 1-Dehydrogenase	Protein Coding	O95479	48	GC01P009234	8.795363	https://www.genecards.org/cgi-bin/carddisp.pl?gene=H6PD
SERPINA6	Serpin Family A Member 6	Protein Coding	P08185	50	GC14M116393	8.78326	https://www.genecards.org/cgi-bin/carddisp.pl?gene=SERPINA6
F7	Coagulation Factor VII	Protein Coding	P08709	52	GC13P113105	8.771629	https://www.genecards.org/cgi-bin/carddisp.pl?gene=F7
CRHR1	Corticotropin Releasing Hormone Receptor 1	Protein Coding	P34998	47	GC17P045784	8.768337	https://www.genecards.org/cgi-bin/carddisp.pl?gene=CRHR1
SLC2A2	Solute Carrier Family 2 Member 2	Protein Coding	P11168	53	GC03M170996	8.724615	https://www.genecards.org/cgi-bin/carddisp.pl?gene=SLC2A2
SLC30A8	Solute Carrier Family 30 Member 8	Protein Coding	Q8IWU4	46	GC08P116950	8.690069	https://www.genecards.org/cgi-bin/carddisp.pl?gene=SLC30A8
HLA-DRB1	Major Histocompatibility Complex, Class II, DR Beta 1	Protein Coding	P01911	51	GC06M084001	8.660913	https://www.genecards.org/cgi-bin/carddisp.pl?gene=HLA-DRB1
ACE2	Angiotensin Converting Enzyme 2	Protein Coding	Q9BYF1	53	GC0XM015494	8.657719	https://www.genecards.org/cgi-bin/carddisp.pl?gene=ACE2

GLI2	GLI Family Zinc Finger 2	Protein Coding	P10070	53	GC02P120735	8.617661	https://www.genecards.org/cgi-bin/carddisp.pl?gene=GLI2
CYP3A4	Cytochrome P450 Family 3 Subfamily A Member 4	Protein Coding	P08684	52	GC07M099834	8.601633	https://www.genecards.org/cgi-bin/carddisp.pl?gene=CYP3A4
MIR29A	MicroRNA 29a	RNA Gene		22	GC07M130876	8.592558	https://www.genecards.org/cgi-bin/carddisp.pl?gene=MIR29A
INPPL1	Inositol Polyphosphate Phosphatase Like 1	Protein Coding	O15357	52	GC11P072223	8.557547	https://www.genecards.org/cgi-bin/carddisp.pl?gene=INPPL1
NLRP3	NLR Family Pyrin Domain Containing 3	Protein Coding	Q96P20	52	GC01P247415	8.548294	https://www.genecards.org/cgi-bin/carddisp.pl?gene=NLRP3
MIRLET7C	MicroRNA Let-7c	RNA Gene		23	GC21P016559	8.544577	https://www.genecards.org/cgi-bin/carddisp.pl?gene=MIRLET7C
LCAT	Lecithin-Cholesterol Acyltransferase	Protein Coding	P04180	51	GC16M067939	8.52624	https://www.genecards.org/cgi-bin/carddisp.pl?gene=LCAT
ENSG00000277577		RNA Gene		5	GC0XM073831	8.516251	https://www.genecards.org/cgi-bin/carddisp.pl?gene=ENSG00000277577
APOA4	Apolipoprotein A4	Protein Coding	P06727	45	GC11M116820	8.51537	https://www.genecards.org/cgi-bin/carddisp.pl?gene=APOA4
TBX3	T-Box Transcription Factor 3	Protein Coding	O15119	48	GC12M114670	8.463013	https://www.genecards.org/cgi-bin/carddisp.pl?gene=TBX3
MIR222	MicroRNA 222	RNA Gene		21	GC0XM045747	8.457954	https://www.genecards.org/cgi-bin/carddisp.pl?gene=MIR222
MCHR1	Melanin Concentrating Hormone Receptor 1	Protein Coding	Q99705	46	GC22P040679	8.433916	https://www.genecards.org/cgi-bin/carddisp.pl?gene=MCHR1

CORIN	Corin, Serine Peptidase	Protein Coding	Q9Y5Q5	48	GC04M047596	8.425174	https://www.genecards.org/cgi-bin/carddisp.pl?gene=CORIN
MMP2	Matrix Metallopeptidase 2	Protein Coding	P08253	57	GC16P058548	8.419318	https://www.genecards.org/cgi-bin/carddisp.pl?gene=MMP2
MLXIPL	MLX Interacting Protein Like	Protein Coding	Q9NP71	47	GC07M073593	8.387573	https://www.genecards.org/cgi-bin/carddisp.pl?gene=MLXIPL
LPA	Lipoprotein(A)	Protein Coding	P08519	44	GC06M160531	8.386879	https://www.genecards.org/cgi-bin/carddisp.pl?gene=LPA
PLA2G7	Phospholipase A2 Group VII	Protein Coding	Q13093	54	GC06M046704	8.374076	https://www.genecards.org/cgi-bin/carddisp.pl?gene=PLA2G7
HMOX1	Heme Oxygenase 1	Protein Coding	P09601	56	GC22P035380	8.370421	https://www.genecards.org/cgi-bin/carddisp.pl?gene=HMOX1
SST	Somatostatin	Protein Coding	P61278	45	GC03M187668	8.363668	https://www.genecards.org/cgi-bin/carddisp.pl?gene=SST
THBS1	Thrombospondin 1	Protein Coding	P07996	50	GC15P039581	8.350809	https://www.genecards.org/cgi-bin/carddisp.pl?gene=THBS1
SOX2	SRY-Box Transcription Factor 2	Protein Coding	P48431	50	GC03P181711	8.349218	https://www.genecards.org/cgi-bin/carddisp.pl?gene=SOX2
GAS5	Growth Arrest Specific 5	RNA Gene		25	GC01M173947	8.346437	https://www.genecards.org/cgi-bin/carddisp.pl?gene=GAS5
MIR25	MicroRNA 25	RNA Gene		21	GC07M100093	8.3316	https://www.genecards.org/cgi-bin/carddisp.pl?gene=MIR25
IL1RN	Interleukin 1 Receptor Antagonist	Protein Coding	P18510	54	GC02P127092	8.312719	https://www.genecards.org/cgi-bin/carddisp.pl?gene=IL1RN

NPPB	Natriuretic Peptide B	Protein Coding	P16860	47	GC01M011858	8.312021	https://www.genecards.org/cgi-bin/carddisp.pl?gene=NPPB
IL2	Interleukin 2	Protein Coding	P60568	50	GC04M122451	8.305912	https://www.genecards.org/cgi-bin/carddisp.pl?gene=IL2
HESX1	HESX Homeobox 1	Protein Coding	Q9UBX0	43	GC03M057207	8.281311	https://www.genecards.org/cgi-bin/carddisp.pl?gene=HESX1
TRH	Thyrotropin Releasing Hormone	Protein Coding	P20396	45	GC03P129974	8.268794	https://www.genecards.org/cgi-bin/carddisp.pl?gene=TRH
ATP10A	ATPase Phospholipid Transporting 10A (Putative)	Protein Coding	O60312	45	GC15M025976	8.264635	https://www.genecards.org/cgi-bin/carddisp.pl?gene=ATP10A
ENPP2	Ectonucleotide Pyrophosphatase/Phosphodiesterase 2	Protein Coding	Q13822	49	GC08M119556	8.262974	https://www.genecards.org/cgi-bin/carddisp.pl?gene=ENPP2
ADRB1	Adrenoceptor Beta 1	Protein Coding	P08588	52	GC10P114044	8.224579	https://www.genecards.org/cgi-bin/carddisp.pl?gene=ADRB1
GCKR	Glucokinase Regulator	Protein Coding	Q14397	44	GC02P027496	8.201373	https://www.genecards.org/cgi-bin/carddisp.pl?gene=GCKR
NHLH2	Nescient Helix-Loop-Helix 2	Protein Coding	Q02577	43	GC01M115836	8.191518	https://www.genecards.org/cgi-bin/carddisp.pl?gene=NHLH2
PTGS2	Prostaglandin-Endoperoxide Synthase 2	Protein Coding	P35354	53	GC01M186671	8.183451	https://www.genecards.org/cgi-bin/carddisp.pl?gene=PTGS2
CPT1A	Carnitine Palmitoyltransferase 1A	Protein Coding	P50416	51	GC11M068754	8.177008	https://www.genecards.org/cgi-bin/carddisp.pl?gene=CPT1A
LDLR	Low Density Lipoprotein Receptor	Protein Coding	P01130	55	GC19P088799	8.166985	https://www.genecards.org/cgi-bin/carddisp.pl?gene=LDLR

NR3C2	Nuclear Receptor Subfamily 3 Group C Member 2	Protein Coding	P08235	50	GC04M148078	8.136854	https://www.genecards.org/cgi-bin/carddisp.pl?gene=NR3C2
MTR	5-Methyltetrahydrofolate-Homocysteine Methyltransferase	Protein Coding	Q99707	52	GC01P236795	8.12805	https://www.genecards.org/cgi-bin/carddisp.pl?gene=MTR
H2AC18	H2A Clustered Histone 18	Protein Coding	Q6FI13	33	GC01M157473	8.126132	https://www.genecards.org/cgi-bin/carddisp.pl?gene=H2AC18
FGFR3	Fibroblast Growth Factor Receptor 3	Protein Coding	P22607	59	GC04P002199	8.120055	https://www.genecards.org/cgi-bin/carddisp.pl?gene=FGFR3
UCN	Urocortin	Protein Coding	P55089	40	GC02M027308	8.101561	https://www.genecards.org/cgi-bin/carddisp.pl?gene=UCN
MIR148A	MicroRNA 148a	RNA Gene		21	GC07M025950	8.091084	https://www.genecards.org/cgi-bin/carddisp.pl?gene=MIR148A
TEMPS	Temple Syndrome	Genetic Locus		2	GC14U901824	8.087459	https://www.genecards.org/cgi-bin/carddisp.pl?gene=TEMPS
MIRLET7D	MicroRNA Let-7d	RNA Gene		23	GC09P094178	8.078259	https://www.genecards.org/cgi-bin/carddisp.pl?gene=MIRLET7D
NEUROD1	Neuronal Differentiation 1	Protein Coding	Q13562	47	GC02M181673	8.069888	https://www.genecards.org/cgi-bin/carddisp.pl?gene=NEUROD1
KISS1	KiSS-1 Metastasis Suppressor	Protein Coding	Q15726	45	GC01M204190	8.06178	https://www.genecards.org/cgi-bin/carddisp.pl?gene=KISS1
SOCS1	Suppressor Of Cytokine Signaling 1	Protein Coding	O15524	50	GC16M013683	8.041486	https://www.genecards.org/cgi-bin/carddisp.pl?gene=SOCS1
ADAM12	ADAM Metallopeptidase Domain 12	Protein Coding	O43184	50	GC10M126012	8.035421	https://www.genecards.org/cgi-bin/carddisp.pl?gene=ADAM12

MC2R	Melanocortin 2 Receptor	Protein Coding	Q01718	50	GC18M031026	8.013258	https://www.genecards.org/cgi-bin/carddisp.pl?gene=MC2R
ENG	Endoglin	Protein Coding	P17813	52	GC09M127815	8.006724	https://www.genecards.org/cgi-bin/carddisp.pl?gene=ENG
MIR221	MicroRNA 221	RNA Gene		21	GC0XM045746	7.996093	https://www.genecards.org/cgi-bin/carddisp.pl?gene=MIR221
MMP3	Matrix Metallopeptidase 3	Protein Coding	P08254	53	GC11M102835	7.981177	https://www.genecards.org/cgi-bin/carddisp.pl?gene=MMP3
SCARB1	Scavenger Receptor Class B Member 1	Protein Coding	Q8WTV0	51	GC12M124776	7.963764	https://www.genecards.org/cgi-bin/carddisp.pl?gene=SCARB1
EMSLR	E2F1 MRNA Stabilizing LncRNA	RNA Gene		14	GC07P103778	7.942617	https://www.genecards.org/cgi-bin/carddisp.pl?gene=EMSLR
MKS1	MKS Transition Zone Complex Subunit 1	Protein Coding	Q9NXB0	45	GC17M058205	7.924444	https://www.genecards.org/cgi-bin/carddisp.pl?gene=MKS1
PIK3R1	Phosphoinositide-3-Kinase Regulatory Subunit 1	Protein Coding	P27986	55	GC05P068215	7.922079	https://www.genecards.org/cgi-bin/carddisp.pl?gene=PIK3R1
IL1R1	Interleukin 1 Receptor Type 1	Protein Coding	P14778	50	GC02P102136	7.908522	https://www.genecards.org/cgi-bin/carddisp.pl?gene=IL1R1
BRAF	B-Raf Proto-Oncogene, Serine/Threonine Kinase	Protein Coding	P15056	58	GC07M140754	7.893148	https://www.genecards.org/cgi-bin/carddisp.pl?gene=BRAF
MIR199A1	MicroRNA 199a-1	RNA Gene		20	GC19M010817	7.881879	https://www.genecards.org/cgi-bin/carddisp.pl?gene=MIR199A1
NR1H4	Nuclear Receptor Subfamily 1 Group H Member 4	Protein Coding	Q96RI1	52	GC12P100473	7.879417	https://www.genecards.org/cgi-bin/carddisp.pl?gene=NR1H4

HBEGF	Heparin Binding EGF Like Growth Factor	Protein Coding	Q99075	48	GC05M140332	7.875729	https://www.genecards.org/cgi-bin/carddisp.pl?gene=HBEGF
AGER	Advanced Glycosylation End-Product Specific Receptor	Protein Coding	Q15109	51	GC06M032180	7.866925	https://www.genecards.org/cgi-bin/carddisp.pl?gene=AGER
VWF	Von Willebrand Factor	Protein Coding	P04275	52	GC12M006194	7.864074	https://www.genecards.org/cgi-bin/carddisp.pl?gene=VWF
ACACB	Acetyl-CoA Carboxylase Beta	Protein Coding	O00763	49	GC12P109116	7.856925	https://www.genecards.org/cgi-bin/carddisp.pl?gene=ACACB
MIR17	MicroRNA 17	RNA Gene		20	GC13P091350	7.855249	https://www.genecards.org/cgi-bin/carddisp.pl?gene=MIR17
SOD1	Superoxide Dismutase 1	Protein Coding	P00441	57	GC21P031659	7.836621	https://www.genecards.org/cgi-bin/carddisp.pl?gene=SOD1
ITGAM	Integrin Subunit Alpha M	Protein Coding	P11215	51	GC16P054241	7.829775	https://www.genecards.org/cgi-bin/carddisp.pl?gene=ITGAM
PRKAB1	Protein Kinase AMP-Activated Non-Catalytic Subunit Beta 1	Protein Coding	Q9Y478	52	GC12P119632	7.813005	https://www.genecards.org/cgi-bin/carddisp.pl?gene=PRKAB1
IL13	Interleukin 13	Protein Coding	P35225	47	GC05P132656	7.807267	https://www.genecards.org/cgi-bin/carddisp.pl?gene=IL13
CYP21A2	Cytochrome P450 Family 21 Subfamily A Member 2	Protein Coding	P08686	47	GC06P112024	7.787591	https://www.genecards.org/cgi-bin/carddisp.pl?gene=CYP21A2
PTH	Parathyroid Hormone	Protein Coding	P01270	49	GC11M013492	7.774494	https://www.genecards.org/cgi-bin/carddisp.pl?gene=PTH
MIR27A	MicroRNA 27a	RNA Gene		24	GC19M015892	7.747231	https://www.genecards.org/cgi-bin/carddisp.pl?gene=MIR27A

DIO2	Iodothyronine Deiodinase 2	Protein Coding	Q92813	44	GC14M080197	7.743362	https://www.genecards.org/cgi-bin/carddisp.pl?gene=DIO2
MIR181A1	MicroRNA 181a-1	RNA Gene		20	GC01M198860	7.741215	https://www.genecards.org/cgi-bin/carddisp.pl?gene=MIR181A1
MYT1L	Myelin Transcription Factor 1 Like	Protein Coding	Q9UL68	46	GC02M001789	7.718694	https://www.genecards.org/cgi-bin/carddisp.pl?gene=MYT1L
LINC-ROR	Long Intergenic Non-Protein Coding RNA, Regulator Of Reprogramming	RNA Gene		17	GC18M057054	7.704679	https://www.genecards.org/cgi-bin/carddisp.pl?gene=LINC-ROR
MIR342	MicroRNA 342	RNA Gene		21	GC14P100109	7.701105	https://www.genecards.org/cgi-bin/carddisp.pl?gene=MIR342
PLAT	Plasminogen Activator, Tissue Type	Protein Coding	P00750	53	GC08M042174	7.684385	https://www.genecards.org/cgi-bin/carddisp.pl?gene=PLAT
AVP	Arginine Vasopressin	Protein Coding	P01185	49	GC20M003082	7.668502	https://www.genecards.org/cgi-bin/carddisp.pl?gene=AVP
HFE	Homeostatic Iron Regulator	Protein Coding	Q30201	50	GC06P026087	7.665728	https://www.genecards.org/cgi-bin/carddisp.pl?gene=HFE
HP	Haptoglobin	Protein Coding	P00738	50	GC16P073656	7.63188	https://www.genecards.org/cgi-bin/carddisp.pl?gene=HP
XBP1	X-Box Binding Protein 1	Protein Coding	P17861	50	GC22M028794	7.62996	https://www.genecards.org/cgi-bin/carddisp.pl?gene=XBP1
CNTF	Ciliary Neurotrophic Factor	Protein Coding	P26441	46	GC11P058622	7.626538	https://www.genecards.org/cgi-bin/carddisp.pl?gene=CNTF
GDF15	Growth Differentiation Factor 15	Protein Coding	Q99988	47	GC19P089116	7.623967	https://www.genecards.org/cgi-bin/carddisp.pl?gene=GDF15

NFKB1	Nuclear Factor Kappa B Subunit 1	Protein Coding	P19838	57	GC04P102501	7.605326	https://www.genecards.org/cgi-bin/carddisp.pl?gene=NFKB1
SLC6A2	Solute Carrier Family 6 Member 2	Protein Coding	P23975	50	GC16P058908	7.585956	https://www.genecards.org/cgi-bin/carddisp.pl?gene=SLC6A2
MIR378A	MicroRNA 378a	RNA Gene		22	GC05P149732	7.581855	https://www.genecards.org/cgi-bin/carddisp.pl?gene=MIR378A
RET	Ret Proto-Oncogene	Protein Coding	P07949	58	GC10P043671	7.558823	https://www.genecards.org/cgi-bin/carddisp.pl?gene=RET
FAS	Fas Cell Surface Death Receptor	Protein Coding	P25445	54	GC10P100956	7.558285	https://www.genecards.org/cgi-bin/carddisp.pl?gene=FAS
MTNR1B	Melatonin Receptor 1B	Protein Coding	P49286	48	GC11P092969	7.557594	https://www.genecards.org/cgi-bin/carddisp.pl?gene=MTNR1B
PRKAA1	Protein Kinase AMP-Activated Catalytic Subunit Alpha 1	Protein Coding	Q13131	51	GC05M040759	7.545566	https://www.genecards.org/cgi-bin/carddisp.pl?gene=PRKAA1
SLC19A1	Solute Carrier Family 19 Member 1	Protein Coding	P41440	50	GC21M045493	7.544477	https://www.genecards.org/cgi-bin/carddisp.pl?gene=SLC19A1
LOC106728418	LEP 5' Regulatory Region	Functional Element		3	GC07P128238	7.541408	https://www.genecards.org/cgi-bin/carddisp.pl?gene=LOC106728418
CS	Citrate Synthase	Protein Coding	O75390	48	GC12M056271	7.539825	https://www.genecards.org/cgi-bin/carddisp.pl?gene=CS
MIR532	MicroRNA 532	RNA Gene		17	GC0XP052674	7.522225	https://www.genecards.org/cgi-bin/carddisp.pl?gene=MIR532
MIR7-3HG	MIR7-3 Host Gene	RNA Gene	Q8N6C7	27	GC19P008873	7.519425	https://www.genecards.org/cgi-bin/carddisp.pl?gene=MIR7-3HG

ADRA2A	Adrenoceptor Alpha 2A	Protein Coding	P08913	50	GC10P111077	7.501094	https://www.genecards.org/cgi-bin/carddisp.pl?gene=ADRA2A
FOXC2	Forkhead Box C2	Protein Coding	Q99958	47	GC16P086631	7.495262	https://www.genecards.org/cgi-bin/carddisp.pl?gene=FOXC2
PTPN11	Protein Tyrosine Phosphatase Non-Receptor Type 11	Protein Coding	Q06124	58	GC12P112418	7.485782	https://www.genecards.org/cgi-bin/carddisp.pl?gene=PTPN11
FN1	Fibronectin 1	Protein Coding	P02751	54	GC02M215360	7.481535	https://www.genecards.org/cgi-bin/carddisp.pl?gene=FN1
EP300	E1A Binding Protein P300	Protein Coding	Q09472	56	GC22P056779	7.46075	https://www.genecards.org/cgi-bin/carddisp.pl?gene=EP300
MIR335	MicroRNA 335	RNA Gene		21	GC07P130496	7.457879	https://www.genecards.org/cgi-bin/carddisp.pl?gene=MIR335
RXRA	Retinoid X Receptor Alpha	Protein Coding	P19793	53	GC09P134317	7.403785	https://www.genecards.org/cgi-bin/carddisp.pl?gene=RXRA
ARNT2	Aryl Hydrocarbon Receptor Nuclear Translocator 2	Protein Coding	Q9HBZ2	48	GC15P080404	7.402759	https://www.genecards.org/cgi-bin/carddisp.pl?gene=ARNT2
ACACA	Acetyl-CoA Carboxylase Alpha	Protein Coding	Q13085	52	GC17M037084	7.394349	https://www.genecards.org/cgi-bin/carddisp.pl?gene=ACACA
LIPG	Lipase G, Endothelial Type	Protein Coding	Q9Y5X9	46	GC18P049560	7.391283	https://www.genecards.org/cgi-bin/carddisp.pl?gene=LIPG
TMEM67	Transmembrane Protein 67	Protein Coding	Q5HYA8	45	GC08P093754	7.385023	https://www.genecards.org/cgi-bin/carddisp.pl?gene=TMEM67
CYP1A2	Cytochrome P450 Family 1 Subfamily A Member 2	Protein Coding	P05177	49	GC15P074748	7.373768	https://www.genecards.org/cgi-bin/carddisp.pl?gene=CYP1A2

HDAC4	Histone Deacetylase 4	Protein Coding	P56524	56	GC02M239048	7.369762	https://www.genecards.org/cgi-bin/carddisp.pl?gene=HDAC4
LINC01258	Long Intergenic Non-Protein Coding RNA 1258	RNA Gene		15	GC04M038423	7.353703	https://www.genecards.org/cgi-bin/carddisp.pl?gene=LINC01258
LINC01725	Long Intergenic Non-Protein Coding RNA 1725	RNA Gene		15	GC01M083348	7.353703	https://www.genecards.org/cgi-bin/carddisp.pl?gene=LINC01725
CFD	Complement Factor D	Protein Coding	P00746	48	GC19P000859	7.349652	https://www.genecards.org/cgi-bin/carddisp.pl?gene=CFD
PAX4	Paired Box 4	Protein Coding	O43316	45	GC07M127610	7.318776	https://www.genecards.org/cgi-bin/carddisp.pl?gene=PAX4
ANGPTL4	Angiopoietin Like 4	Protein Coding	Q9BY76	48	GC19P008363	7.312982	https://www.genecards.org/cgi-bin/carddisp.pl?gene=ANGPTL4
HLA-DQB1	Major Histocompatibility Complex, Class II, DQ Beta 1	Protein Coding	P01920	46	GC06M084010	7.312504	https://www.genecards.org/cgi-bin/carddisp.pl?gene=HLA-DQB1
FBN1	Fibrillin 1	Protein Coding	P35555	51	GC15M048408	7.297145	https://www.genecards.org/cgi-bin/carddisp.pl?gene=FBN1
IDO1	Indoleamine 2,3-Dioxygenase 1	Protein Coding	P14902	51	GC08P039891	7.295714	https://www.genecards.org/cgi-bin/carddisp.pl?gene=IDO1
TFAM	Transcription Factor A, Mitochondrial	Protein Coding	Q00059	48	GC10P058385	7.276613	https://www.genecards.org/cgi-bin/carddisp.pl?gene=TFAM
FGF8	Fibroblast Growth Factor 8	Protein Coding	P55075	52	GC10M101770	7.268078	https://www.genecards.org/cgi-bin/carddisp.pl?gene=FGF8
MLN	Motilin	Protein Coding	P12872	38	GC06M033794	7.256998	https://www.genecards.org/cgi-bin/carddisp.pl?gene=MLN

SELL	Selectin L	Protein Coding	P14151	47	GC01M169690	7.246827	https://www.genecards.org/cgi-bin/carddisp.pl?gene=SELL
TRA-TGC7-1	TRNA-Ala (Anticodon TGC) 7-1	RNA Gene		10	GC06M083771	7.243946	https://www.genecards.org/cgi-bin/carddisp.pl?gene=TRA-TGC7-1
TRA-TGC5-1	TRNA-Ala (Anticodon TGC) 5-1	RNA Gene		9	GC06M028817	7.243946	https://www.genecards.org/cgi-bin/carddisp.pl?gene=TRA-TGC5-1
MIRLET7B	MicroRNA Let-7b	RNA Gene		21	GC22P056922	7.236205	https://www.genecards.org/cgi-bin/carddisp.pl?gene=MIRLET7B
PTX3	Pentraxin 3	Protein Coding	P26022	47	GC03P157436	7.229889	https://www.genecards.org/cgi-bin/carddisp.pl?gene=PTX3
HADHB	Hydroxyacyl-CoA Dehydrogenase Trifunctional Multienzyme Complex Subunit Beta	Protein Coding	P55084	50	GC02P026243	7.225973	https://www.genecards.org/cgi-bin/carddisp.pl?gene=HADHB
ACTB	Actin Beta	Protein Coding	P60709	53	GC07M005527	7.192475	https://www.genecards.org/cgi-bin/carddisp.pl?gene=ACTB
MT-CO3	Mitochondrially Encoded Cytochrome C Oxidase III	Protein Coding	P00414	33	GCMTP009209	7.154419	https://www.genecards.org/cgi-bin/carddisp.pl?gene=MT-CO3
MSTN	Myostatin	Protein Coding	O14793	50	GC02M190055	7.150717	https://www.genecards.org/cgi-bin/carddisp.pl?gene=MSTN
G6PC1	Glucose-6-Phosphatase Catalytic Subunit 1	Protein Coding	P35575	48	GC17P087745	7.148647	https://www.genecards.org/cgi-bin/carddisp.pl?gene=G6PC1
F2R	Coagulation Factor II Thrombin Receptor	Protein Coding	P25116	51	GC05P076716	7.143438	https://www.genecards.org/cgi-bin/carddisp.pl?gene=F2R
LINC00504	Long Intergenic Non-Protein Coding RNA 504	RNA Gene		19	GC04M014480	7.121239	https://www.genecards.org/cgi-bin/carddisp.pl?gene=LINC00504

ARF4-AS1	ARF4 Antisense RNA 1	RNA Gene		15	GC03P057597	7.121239	https://www.genecards.org/cgi-bin/carddisp.pl?gene=ARF4-AS1
LINC02882	Long Intergenic Non-Protein Coding RNA 2882	RNA Gene		15	GC12M073212	7.121239	https://www.genecards.org/cgi-bin/carddisp.pl?gene=LINC02882
LINC02955	Long Intergenic Non-Protein Coding RNA 2955	RNA Gene		15	GC12P030918	7.121239	https://www.genecards.org/cgi-bin/carddisp.pl?gene=LINC02955
LINC02895	Long Intergenic Non-Protein Coding RNA 2895	RNA Gene		13	GC15M038012	7.121239	https://www.genecards.org/cgi-bin/carddisp.pl?gene=LINC02895
ENSG00000230490	Novel Transcript	RNA Gene		11	GC13M038796	7.121239	https://www.genecards.org/cgi-bin/carddisp.pl?gene=ENSG00000230490
ENSG00000245768	Novel Transcript	RNA Gene		11	GC16P058733	7.121239	https://www.genecards.org/cgi-bin/carddisp.pl?gene=ENSG00000245768
ENSG00000250519	Novel Transcript	RNA Gene		11	GC11P094182	7.121239	https://www.genecards.org/cgi-bin/carddisp.pl?gene=ENSG00000250519
ENSG00000258081	Novel Transcript	RNA Gene		11	GC14P040192	7.121239	https://www.genecards.org/cgi-bin/carddisp.pl?gene=ENSG00000258081
ENSG00000235450	Novel Transcript	RNA Gene		10	GC07M091366	7.121239	https://www.genecards.org/cgi-bin/carddisp.pl?gene=ENSG00000235450
ENSG00000251216	Novel Transcript	RNA Gene		9	GC04M173924	7.121239	https://www.genecards.org/cgi-bin/carddisp.pl?gene=ENSG00000251216

LOC101927560	Uncharacterized LOC101927560	RNA Gene		9	GC01M083801	7.121239	https://www.genecards.org/cgi-bin/carddisp.pl?gene=LOC101927560
ENSG00000253288	Novel Transcript	RNA Gene		8	GC08M137749	7.121239	https://www.genecards.org/cgi-bin/carddisp.pl?gene=ENSG00000253288
lnc-IQCM-2		RNA Gene		8	GC04M149353	7.121239	https://www.genecards.org/cgi-bin/carddisp.pl?gene=lnc-IQCM-2
lnc-HMGXB4-8		RNA Gene		7	GC22P056544	7.121239	https://www.genecards.org/cgi-bin/carddisp.pl?gene=lnc-HMGXB4-8
MK280073-023		RNA Gene		6	GC01P173326	7.121239	https://www.genecards.org/cgi-bin/carddisp.pl?gene=MK280073-023
MK280073-058		RNA Gene		6	GC10P112107	7.121239	https://www.genecards.org/cgi-bin/carddisp.pl?gene=MK280073-058
MK280073-008		RNA Gene		5	GC01P059371	7.121239	https://www.genecards.org/cgi-bin/carddisp.pl?gene=MK280073-008
MK280073-020		RNA Gene		5	GC01P092548	7.121239	https://www.genecards.org/cgi-bin/carddisp.pl?gene=MK280073-020
MK280073-022		RNA Gene		5	GC01M172595	7.121239	https://www.genecards.org/cgi-bin/carddisp.pl?gene=MK280073-022
MK280073-049		RNA Gene		5	GC10M076146	7.121239	https://www.genecards.org/cgi-bin/carddisp.pl?gene=MK280073-049
MK280073-055		RNA Gene		5	GC10M094717	7.121239	https://www.genecards.org/cgi-bin/carddisp.pl?gene=MK280073-055
MK280073-060		RNA Gene		5	GC10M117083	7.121239	https://www.genecards.org/cgi-bin/carddisp.pl?gene=MK280073-060

MK280073-063		RNA Gene		5	GC10P129576	7.121239	https://www.genecards.org/cgi-bin/carddisp.pl?gene=MK280073-063
MK280073-083		RNA Gene		5	GC11P086701	7.121239	https://www.genecards.org/cgi-bin/carddisp.pl?gene=MK280073-083
MK280073-093		RNA Gene		5	GC11P128143	7.121239	https://www.genecards.org/cgi-bin/carddisp.pl?gene=MK280073-093
MK280073-007		RNA Gene		4	GC01M055497	7.121239	https://www.genecards.org/cgi-bin/carddisp.pl?gene=MK280073-007
MK280073-013		RNA Gene		4	GC01M067179	7.121239	https://www.genecards.org/cgi-bin/carddisp.pl?gene=MK280073-013
MK280073-018		RNA Gene		4	GC01P090554	7.121239	https://www.genecards.org/cgi-bin/carddisp.pl?gene=MK280073-018
MK280073-025		RNA Gene		4	GC01P185641	7.121239	https://www.genecards.org/cgi-bin/carddisp.pl?gene=MK280073-025
MK280073-027		RNA Gene		4	GC01M211406	7.121239	https://www.genecards.org/cgi-bin/carddisp.pl?gene=MK280073-027
MK280073-028		RNA Gene		4	GC01P216489	7.121239	https://www.genecards.org/cgi-bin/carddisp.pl?gene=MK280073-028
MK280073-030		RNA Gene		4	GC01M234053	7.121239	https://www.genecards.org/cgi-bin/carddisp.pl?gene=MK280073-030
MK280073-040		RNA Gene		4	GC10P031285	7.121239	https://www.genecards.org/cgi-bin/carddisp.pl?gene=MK280073-040
MK280073-044		RNA Gene		4	GC10P058233	7.121239	https://www.genecards.org/cgi-bin/carddisp.pl?gene=MK280073-044

MK280073-052		RNA Gene		4	GC10P102887	7.121239	https://www.genecards.org/cgi-bin/carddisp.pl?gene=MK280073-052
MK280073-059		RNA Gene		4	GC10P112254	7.121239	https://www.genecards.org/cgi-bin/carddisp.pl?gene=MK280073-059
MK280073-072		RNA Gene		4	GC11P030182	7.121239	https://www.genecards.org/cgi-bin/carddisp.pl?gene=MK280073-072
MK280073-081		RNA Gene		4	GC11P080353	7.121239	https://www.genecards.org/cgi-bin/carddisp.pl?gene=MK280073-081
MK280073-082		RNA Gene		4	GC11M115207	7.121239	https://www.genecards.org/cgi-bin/carddisp.pl?gene=MK280073-082
MK280073-090		RNA Gene		4	GC11P113716	7.121239	https://www.genecards.org/cgi-bin/carddisp.pl?gene=MK280073-090
MK280073-091		RNA Gene		4	GC11P114545	7.121239	https://www.genecards.org/cgi-bin/carddisp.pl?gene=MK280073-091
MK280073-001		RNA Gene		3	GC01P004042	7.121239	https://www.genecards.org/cgi-bin/carddisp.pl?gene=MK280073-001
MK280073-002		RNA Gene		3	GC01P013083	7.121239	https://www.genecards.org/cgi-bin/carddisp.pl?gene=MK280073-002
MK280073-003		RNA Gene		3	GC01P034270	7.121239	https://www.genecards.org/cgi-bin/carddisp.pl?gene=MK280073-003
MK280073-004		RNA Gene		3	GC01M035057	7.121239	https://www.genecards.org/cgi-bin/carddisp.pl?gene=MK280073-004
MK280073-005		RNA Gene		3	GC01M037378	7.121239	https://www.genecards.org/cgi-bin/carddisp.pl?gene=MK280073-005

MK280073-006		RNA Gene		3	GC01M050889	7.121239	https://www.genecards.org/cgi-bin/carddisp.pl?gene=MK280073-006
MK280073-009		RNA Gene		3	GC01P060381	7.121239	https://www.genecards.org/cgi-bin/carddisp.pl?gene=MK280073-009
MK280073-010		RNA Gene		3	GC01P060780	7.121239	https://www.genecards.org/cgi-bin/carddisp.pl?gene=MK280073-010
MK280073-011		RNA Gene		3	GC01M066187	7.121239	https://www.genecards.org/cgi-bin/carddisp.pl?gene=MK280073-011
MK280073-012		RNA Gene		3	GC01M066201	7.121239	https://www.genecards.org/cgi-bin/carddisp.pl?gene=MK280073-012
MK280073-014		RNA Gene		3	GC01P075700	7.121239	https://www.genecards.org/cgi-bin/carddisp.pl?gene=MK280073-014
MK280073-015		RNA Gene		3	GC01P075850	7.121239	https://www.genecards.org/cgi-bin/carddisp.pl?gene=MK280073-015
MK280073-016		RNA Gene		3	GC01M086628	7.121239	https://www.genecards.org/cgi-bin/carddisp.pl?gene=MK280073-016
MK280073-017		RNA Gene		3	GC01P086821	7.121239	https://www.genecards.org/cgi-bin/carddisp.pl?gene=MK280073-017
MK280073-019		RNA Gene		3	GC01M092236	7.121239	https://www.genecards.org/cgi-bin/carddisp.pl?gene=MK280073-019
MK280073-021		RNA Gene		3	GC01P113343	7.121239	https://www.genecards.org/cgi-bin/carddisp.pl?gene=MK280073-021
MK280073-024		RNA Gene		3	GC01M185092	7.121239	https://www.genecards.org/cgi-bin/carddisp.pl?gene=MK280073-024

MK280073-026		RNA Gene		3	GC01M188760	7.121239	https://www.genecards.org/cgi-bin/carddisp.pl?gene=MK280073-026
MK280073-029		RNA Gene		3	GC01M222136	7.121239	https://www.genecards.org/cgi-bin/carddisp.pl?gene=MK280073-029
MK280073-031		RNA Gene		3	GC01P248767	7.121239	https://www.genecards.org/cgi-bin/carddisp.pl?gene=MK280073-031
MK280073-032		RNA Gene		3	GC10P001503	7.121239	https://www.genecards.org/cgi-bin/carddisp.pl?gene=MK280073-032
MK280073-033		RNA Gene		3	GC10M005088	7.121239	https://www.genecards.org/cgi-bin/carddisp.pl?gene=MK280073-033
MK280073-034		RNA Gene		3	GC10M005188	7.121239	https://www.genecards.org/cgi-bin/carddisp.pl?gene=MK280073-034
MK280073-035		RNA Gene		3	GC10M010073	7.121239	https://www.genecards.org/cgi-bin/carddisp.pl?gene=MK280073-035
MK280073-036		RNA Gene		3	GC10M015668	7.121239	https://www.genecards.org/cgi-bin/carddisp.pl?gene=MK280073-036
MK280073-037		RNA Gene		3	GC10M023670	7.121239	https://www.genecards.org/cgi-bin/carddisp.pl?gene=MK280073-037
MK280073-038		RNA Gene		3	GC10P027422	7.121239	https://www.genecards.org/cgi-bin/carddisp.pl?gene=MK280073-038
MK280073-039		RNA Gene		3	GC10P027847	7.121239	https://www.genecards.org/cgi-bin/carddisp.pl?gene=MK280073-039
MK280073-041		RNA Gene		3	GC10P043835	7.121239	https://www.genecards.org/cgi-bin/carddisp.pl?gene=MK280073-041

MK280073-042		RNA Gene		3	GC10P053115	7.121239	https://www.genecards.org/cgi-bin/carddisp.pl?gene=MK280073-042
MK280073-043		RNA Gene		3	GC10M057008	7.121239	https://www.genecards.org/cgi-bin/carddisp.pl?gene=MK280073-043
MK280073-045		RNA Gene		3	GC10P063146	7.121239	https://www.genecards.org/cgi-bin/carddisp.pl?gene=MK280073-045
MK280073-046		RNA Gene		3	GC10M064015	7.121239	https://www.genecards.org/cgi-bin/carddisp.pl?gene=MK280073-046
MK280073-047		RNA Gene		3	GC10P072439	7.121239	https://www.genecards.org/cgi-bin/carddisp.pl?gene=MK280073-047
MK280073-048		RNA Gene		3	GC10M075564	7.121239	https://www.genecards.org/cgi-bin/carddisp.pl?gene=MK280073-048
MK280073-050		RNA Gene		3	GC10M083071	7.121239	https://www.genecards.org/cgi-bin/carddisp.pl?gene=MK280073-050
MK280073-051		RNA Gene		3	GC10M088249	7.121239	https://www.genecards.org/cgi-bin/carddisp.pl?gene=MK280073-051
MK280073-053		RNA Gene		3	GC10M091735	7.121239	https://www.genecards.org/cgi-bin/carddisp.pl?gene=MK280073-053
MK280073-054		RNA Gene		3	GC10P102890	7.121239	https://www.genecards.org/cgi-bin/carddisp.pl?gene=MK280073-054
MK280073-056		RNA Gene		3	GC10M098725	7.121239	https://www.genecards.org/cgi-bin/carddisp.pl?gene=MK280073-056
MK280073-057		RNA Gene		3	GC10P101263	7.121239	https://www.genecards.org/cgi-bin/carddisp.pl?gene=MK280073-057

MK280073-061		RNA Gene		3	GC10M120345	7.121239	https://www.genecards.org/cgi-bin/carddisp.pl?gene=MK280073-061
MK280073-062		RNA Gene		3	GC10P125651	7.121239	https://www.genecards.org/cgi-bin/carddisp.pl?gene=MK280073-062
MK280073-064		RNA Gene		3	GC11M009442	7.121239	https://www.genecards.org/cgi-bin/carddisp.pl?gene=MK280073-064
MK280073-065		RNA Gene		3	GC11P006984	7.121239	https://www.genecards.org/cgi-bin/carddisp.pl?gene=MK280073-065
MK280073-066		RNA Gene		3	GC11M009551	7.121239	https://www.genecards.org/cgi-bin/carddisp.pl?gene=MK280073-066
MK280073-068		RNA Gene		3	GC11M014172	7.121239	https://www.genecards.org/cgi-bin/carddisp.pl?gene=MK280073-068
MK280073-069		RNA Gene		3	GC11P018904	7.121239	https://www.genecards.org/cgi-bin/carddisp.pl?gene=MK280073-069
MK280073-070		RNA Gene		3	GC11M025647	7.121239	https://www.genecards.org/cgi-bin/carddisp.pl?gene=MK280073-070
MK280073-071		RNA Gene		3	GC11P025835	7.121239	https://www.genecards.org/cgi-bin/carddisp.pl?gene=MK280073-071
MK280073-073		RNA Gene		3	GC11P032735	7.121239	https://www.genecards.org/cgi-bin/carddisp.pl?gene=MK280073-073
MK280073-074		RNA Gene		3	GC11M034762	7.121239	https://www.genecards.org/cgi-bin/carddisp.pl?gene=MK280073-074
MK280073-075		RNA Gene		3	GC11P035706	7.121239	https://www.genecards.org/cgi-bin/carddisp.pl?gene=MK280073-075

MK280073-076		RNA Gene		3	GC11M115205	7.121239	https://www.genecards.org/cgi-bin/carddisp.pl?gene=MK280073-076
MK280073-077		RNA Gene		3	GC11M115206	7.121239	https://www.genecards.org/cgi-bin/carddisp.pl?gene=MK280073-077
MK280073-078		RNA Gene		3	GC11P080252	7.121239	https://www.genecards.org/cgi-bin/carddisp.pl?gene=MK280073-078
MK280073-079		RNA Gene		3	GC11P080274	7.121239	https://www.genecards.org/cgi-bin/carddisp.pl?gene=MK280073-079
MK280073-080		RNA Gene		3	GC11P081504	7.121239	https://www.genecards.org/cgi-bin/carddisp.pl?gene=MK280073-080
MK280073-084		RNA Gene		3	GC11M115208	7.121239	https://www.genecards.org/cgi-bin/carddisp.pl?gene=MK280073-084
MK280073-085		RNA Gene		3	GC11M115209	7.121239	https://www.genecards.org/cgi-bin/carddisp.pl?gene=MK280073-085
MK280073-086		RNA Gene		3	GC11M115210	7.121239	https://www.genecards.org/cgi-bin/carddisp.pl?gene=MK280073-086
MK280073-087		RNA Gene		3	GC11M115211	7.121239	https://www.genecards.org/cgi-bin/carddisp.pl?gene=MK280073-087
MK280073-088		RNA Gene		3	GC11P101642	7.121239	https://www.genecards.org/cgi-bin/carddisp.pl?gene=MK280073-088
MK280073-089		RNA Gene		3	GC11M114268	7.121239	https://www.genecards.org/cgi-bin/carddisp.pl?gene=MK280073-089
MK280073-092		RNA Gene		3	GC11M115899	7.121239	https://www.genecards.org/cgi-bin/carddisp.pl?gene=MK280073-092

MK280073-094		RNA Gene		3	GC12P033192	7.121239	https://www.genecards.org/cgi-bin/carddisp.pl?gene=MK280073-094
MK280073-095		RNA Gene		3	GC12P033193	7.121239	https://www.genecards.org/cgi-bin/carddisp.pl?gene=MK280073-095
MK280073-096		RNA Gene		3	GC12P033194	7.121239	https://www.genecards.org/cgi-bin/carddisp.pl?gene=MK280073-096
MK280073-097		RNA Gene		3	GC12P033195	7.121239	https://www.genecards.org/cgi-bin/carddisp.pl?gene=MK280073-097
MK280073-098		RNA Gene		3	GC12M026427	7.121239	https://www.genecards.org/cgi-bin/carddisp.pl?gene=MK280073-098
MK280073-101		RNA Gene		3	GC12P033197	7.121239	https://www.genecards.org/cgi-bin/carddisp.pl?gene=MK280073-101
MK280073-102		RNA Gene		3	GC12P031092	7.121239	https://www.genecards.org/cgi-bin/carddisp.pl?gene=MK280073-102
MK280073-103		RNA Gene		3	GC12P031106	7.121239	https://www.genecards.org/cgi-bin/carddisp.pl?gene=MK280073-103
MK280073-104		RNA Gene		3	GC12M030524	7.121239	https://www.genecards.org/cgi-bin/carddisp.pl?gene=MK280073-104
MK280073-105		RNA Gene		3	GC12P033236	7.121239	https://www.genecards.org/cgi-bin/carddisp.pl?gene=MK280073-105
MK280073-106		RNA Gene		3	GC12P045374	7.121239	https://www.genecards.org/cgi-bin/carddisp.pl?gene=MK280073-106
MK280073-107		RNA Gene		3	GC12M046425	7.121239	https://www.genecards.org/cgi-bin/carddisp.pl?gene=MK280073-107

MK280073-108		RNA Gene		3	GC12M047455	7.121239	https://www.genecards.org/cgi-bin/carddisp.pl?gene=MK280073-108
MK280073-109		RNA Gene		3	GC12M048324	7.121239	https://www.genecards.org/cgi-bin/carddisp.pl?gene=MK280073-109
MK280073-110		RNA Gene		3	GC12M055438	7.121239	https://www.genecards.org/cgi-bin/carddisp.pl?gene=MK280073-110
lnc-LRP5L-15		RNA Gene		3	GC22M071093	7.121239	https://www.genecards.org/cgi-bin/carddisp.pl?gene=lnc-LRP5L-15
MK280073-067		RNA Gene		2	GC11M013627	7.121239	https://www.genecards.org/cgi-bin/carddisp.pl?gene=MK280073-067
MK280073-099		RNA Gene		2	GC12M026428	7.121239	https://www.genecards.org/cgi-bin/carddisp.pl?gene=MK280073-099
MK280073-100		RNA Gene		2	GC12P033196	7.121239	https://www.genecards.org/cgi-bin/carddisp.pl?gene=MK280073-100
MTHFD1	Methylenetetrahydrofolate Dehydrogenase, Cyclohydrolase And Formyltetrahydrofolate Synthetase 1	Protein Coding	P11586	48	GC14P064388	7.11319	https://www.genecards.org/cgi-bin/carddisp.pl?gene=MTHFD1
MIR22	MicroRNA 22	RNA Gene		22	GC17M001713	7.105328	https://www.genecards.org/cgi-bin/carddisp.pl?gene=MIR22
ACADM	Acyl-CoA Dehydrogenase Medium Chain	Protein Coding	P11310	50	GC01P075724	7.10247	https://www.genecards.org/cgi-bin/carddisp.pl?gene=ACADM
PWAR6	Prader Willi/Angelman Region RNA 6	RNA Gene		13	GC15P025031	7.095987	https://www.genecards.org/cgi-bin/carddisp.pl?gene=PWAR6
SERPINC1	Serpin Family C Member 1	Protein Coding	P01008	54	GC01M174813	7.086983	https://www.genecards.org/cgi-bin/carddisp.pl?gene=SERPINC1

SELP	Selectin P	Protein Coding	P16109	49	GC01M169558	7.081635	https://www.genecards.org/cgi-bin/carddisp.pl?gene=SELP
MIF	Macrophage Migration Inhibitory Factor	Protein Coding	P14174	52	GC22P023894	7.075693	https://www.genecards.org/cgi-bin/carddisp.pl?gene=MIF
FUZ	Fuzzy Planar Cell Polarity Protein	Protein Coding	Q9BT04	43	GC19M049806	7.07168	https://www.genecards.org/cgi-bin/carddisp.pl?gene=FUZ
MIR30A	MicroRNA 30a	RNA Gene		20	GC06M071403	7.064877	https://www.genecards.org/cgi-bin/carddisp.pl?gene=MIR30A
CRHR2	Corticotropin Releasing Hormone Receptor 2	Protein Coding	Q13324	47	GC07M030651	7.043779	https://www.genecards.org/cgi-bin/carddisp.pl?gene=CRHR2
CCR2	C-C Motif Chemokine Receptor 2	Protein Coding	P41597	48	GC03P053749	7.038938	https://www.genecards.org/cgi-bin/carddisp.pl?gene=CCR2
MIR18A	MicroRNA 18a	RNA Gene		20	GC13P091636	7.031627	https://www.genecards.org/cgi-bin/carddisp.pl?gene=MIR18A
WFS1	Wolframin ER Transmembrane Glycoprotein	Protein Coding	O76024	50	GC04P006269	7.010735	https://www.genecards.org/cgi-bin/carddisp.pl?gene=WFS1
TFRC	Transferrin Receptor	Protein Coding	P02786	54	GC03M196170	6.998893	https://www.genecards.org/cgi-bin/carddisp.pl?gene=TFRC
ADM	Adrenomedullin	Protein Coding	P35318	49	GC11P010304	6.997169	https://www.genecards.org/cgi-bin/carddisp.pl?gene=ADM
MK280073-197		RNA Gene		6	GC15P057700	6.97178	https://www.genecards.org/cgi-bin/carddisp.pl?gene=MK280073-197
MK280073-346		RNA Gene		6	GC02M060134	6.97178	https://www.genecards.org/cgi-bin/carddisp.pl?gene=MK280073-346

MK280073-501		RNA Gene		6	GC04M181631	6.97178	https://www.genecards.org/cgi-bin/carddisp.pl?gene=MK280073-501
MK280073-120		RNA Gene		5	GC12P089444	6.97178	https://www.genecards.org/cgi-bin/carddisp.pl?gene=MK280073-120
MK280073-121		RNA Gene		5	GC12P094826	6.97178	https://www.genecards.org/cgi-bin/carddisp.pl?gene=MK280073-121
MK280073-164		RNA Gene		5	GC14P041088	6.97178	https://www.genecards.org/cgi-bin/carddisp.pl?gene=MK280073-164
MK280073-202		RNA Gene		5	GC15M137107	6.97178	https://www.genecards.org/cgi-bin/carddisp.pl?gene=MK280073-202
MK280073-300		RNA Gene		5	GC18M055802	6.97178	https://www.genecards.org/cgi-bin/carddisp.pl?gene=MK280073-300
MK280073-334		RNA Gene		5	GC19P093548	6.97178	https://www.genecards.org/cgi-bin/carddisp.pl?gene=MK280073-334
MK280073-475		RNA Gene		5	GC03M193609	6.97178	https://www.genecards.org/cgi-bin/carddisp.pl?gene=MK280073-475
MK280073-493		RNA Gene		5	GC04M074249	6.97178	https://www.genecards.org/cgi-bin/carddisp.pl?gene=MK280073-493
MK280073-510		RNA Gene		5	GC05M033352	6.97178	https://www.genecards.org/cgi-bin/carddisp.pl?gene=MK280073-510
MK280073-521		RNA Gene		5	GC05P078685	6.97178	https://www.genecards.org/cgi-bin/carddisp.pl?gene=MK280073-521
MK280073-571		RNA Gene		5	GC07P032182	6.97178	https://www.genecards.org/cgi-bin/carddisp.pl?gene=MK280073-571

MK280073-115		RNA Gene		4	GC12M066098	6.97178	https://www.genecards.org/cgi-bin/carddisp.pl?gene=MK280073-115
MK280073-117		RNA Gene		4	GC12P072448	6.97178	https://www.genecards.org/cgi-bin/carddisp.pl?gene=MK280073-117
MK280073-123		RNA Gene		4	GC12M097607	6.97178	https://www.genecards.org/cgi-bin/carddisp.pl?gene=MK280073-123
MK280073-140		RNA Gene		4	GC13M066024	6.97178	https://www.genecards.org/cgi-bin/carddisp.pl?gene=MK280073-140
MK280073-149		RNA Gene		4	GC13P094375	6.97178	https://www.genecards.org/cgi-bin/carddisp.pl?gene=MK280073-149
MK280073-150		RNA Gene		4	GC13P094874	6.97178	https://www.genecards.org/cgi-bin/carddisp.pl?gene=MK280073-150
MK280073-153		RNA Gene		4	GC13P110050	6.97178	https://www.genecards.org/cgi-bin/carddisp.pl?gene=MK280073-153
MK280073-175		RNA Gene		4	GC14P054342	6.97178	https://www.genecards.org/cgi-bin/carddisp.pl?gene=MK280073-175
MK280073-176		RNA Gene		4	GC14P056234	6.97178	https://www.genecards.org/cgi-bin/carddisp.pl?gene=MK280073-176
MK280073-180		RNA Gene		4	GC14M066694	6.97178	https://www.genecards.org/cgi-bin/carddisp.pl?gene=MK280073-180
MK280073-182		RNA Gene		4	GC14M072413	6.97178	https://www.genecards.org/cgi-bin/carddisp.pl?gene=MK280073-182
MK280073-199		RNA Gene		4	GC15M138480	6.97178	https://www.genecards.org/cgi-bin/carddisp.pl?gene=MK280073-199

MK280073-203		RNA Gene		4	GC15P140062	6.97178	https://www.genecards.org/cgi-bin/carddisp.pl?gene=MK280073-203
MK280073-205		RNA Gene		4	GC15P140063	6.97178	https://www.genecards.org/cgi-bin/carddisp.pl?gene=MK280073-205
MK280073-206		RNA Gene		4	GC15M138483	6.97178	https://www.genecards.org/cgi-bin/carddisp.pl?gene=MK280073-206
MK280073-207		RNA Gene		4	GC15P140064	6.97178	https://www.genecards.org/cgi-bin/carddisp.pl?gene=MK280073-207
MK280073-216		RNA Gene		4	GC15P140068	6.97178	https://www.genecards.org/cgi-bin/carddisp.pl?gene=MK280073-216
MK280073-243		RNA Gene		4	GC16P065557	6.97178	https://www.genecards.org/cgi-bin/carddisp.pl?gene=MK280073-243
MK280073-273		RNA Gene		4	GC17M069617	6.97178	https://www.genecards.org/cgi-bin/carddisp.pl?gene=MK280073-273
MK280073-283		RNA Gene		4	GC18M031418	6.97178	https://www.genecards.org/cgi-bin/carddisp.pl?gene=MK280073-283
MK280073-295		RNA Gene		4	GC18P049427	6.97178	https://www.genecards.org/cgi-bin/carddisp.pl?gene=MK280073-295
MK280073-296		RNA Gene		4	GC18M051972	6.97178	https://www.genecards.org/cgi-bin/carddisp.pl?gene=MK280073-296
MK280073-351		RNA Gene		4	GC02P084228	6.97178	https://www.genecards.org/cgi-bin/carddisp.pl?gene=MK280073-351
MK280073-353		RNA Gene		4	GC02P097939	6.97178	https://www.genecards.org/cgi-bin/carddisp.pl?gene=MK280073-353

MK280073-354		RNA Gene		4	GC02P148383	6.97178	https://www.genecards.org/cgi-bin/carddisp.pl?gene=MK280073-354
MK280073-359		RNA Gene		4	GC02M157401	6.97178	https://www.genecards.org/cgi-bin/carddisp.pl?gene=MK280073-359
MK280073-389		RNA Gene		4	GC20P024515	6.97178	https://www.genecards.org/cgi-bin/carddisp.pl?gene=MK280073-389
MK280073-453		RNA Gene		4	GC03M040957	6.97178	https://www.genecards.org/cgi-bin/carddisp.pl?gene=MK280073-453
MK280073-456		RNA Gene		4	GC03M070795	6.97178	https://www.genecards.org/cgi-bin/carddisp.pl?gene=MK280073-456
MK280073-460		RNA Gene		4	GC03M101539	6.97178	https://www.genecards.org/cgi-bin/carddisp.pl?gene=MK280073-460
MK280073-461		RNA Gene		4	GC03M107696	6.97178	https://www.genecards.org/cgi-bin/carddisp.pl?gene=MK280073-461
MK280073-464		RNA Gene		4	GC03P122736	6.97178	https://www.genecards.org/cgi-bin/carddisp.pl?gene=MK280073-464
MK280073-468		RNA Gene		4	GC03M141299	6.97178	https://www.genecards.org/cgi-bin/carddisp.pl?gene=MK280073-468
MK280073-470		RNA Gene		4	GC03P158037	6.97178	https://www.genecards.org/cgi-bin/carddisp.pl?gene=MK280073-470
MK280073-473		RNA Gene		4	GC03P174608	6.97178	https://www.genecards.org/cgi-bin/carddisp.pl?gene=MK280073-473
MK280073-490		RNA Gene		4	GC04M054533	6.97178	https://www.genecards.org/cgi-bin/carddisp.pl?gene=MK280073-490

MK280073-513		RNA Gene		4	GC05P051640	6.97178	https://www.genecards.org/cgi-bin/carddisp.pl?gene=MK280073-513
MK280073-519		RNA Gene		4	GC05P067783	6.97178	https://www.genecards.org/cgi-bin/carddisp.pl?gene=MK280073-519
MK280073-522		RNA Gene		4	GC05M080531	6.97178	https://www.genecards.org/cgi-bin/carddisp.pl?gene=MK280073-522
MK280073-523		RNA Gene		4	GC05P080568	6.97178	https://www.genecards.org/cgi-bin/carddisp.pl?gene=MK280073-523
MK280073-524		RNA Gene		4	GC05M081057	6.97178	https://www.genecards.org/cgi-bin/carddisp.pl?gene=MK280073-524
MK280073-525		RNA Gene		4	GC05P081595	6.97178	https://www.genecards.org/cgi-bin/carddisp.pl?gene=MK280073-525
MK280073-527		RNA Gene		4	GC05P090242	6.97178	https://www.genecards.org/cgi-bin/carddisp.pl?gene=MK280073-527
MK280073-533		RNA Gene		4	GC05M158442	6.97178	https://www.genecards.org/cgi-bin/carddisp.pl?gene=MK280073-533
MK280073-544		RNA Gene		4	GC06M085585	6.97178	https://www.genecards.org/cgi-bin/carddisp.pl?gene=MK280073-544
MK280073-557		RNA Gene		4	GC06M112631	6.97178	https://www.genecards.org/cgi-bin/carddisp.pl?gene=MK280073-557
MK280073-561		RNA Gene		4	GC06P117877	6.97178	https://www.genecards.org/cgi-bin/carddisp.pl?gene=MK280073-561
MK280073-586		RNA Gene		4	GC07P091510	6.97178	https://www.genecards.org/cgi-bin/carddisp.pl?gene=MK280073-586

MK280073-111		RNA Gene		3	GC12M055433	6.97178	https://www.genecards.org/cgi-bin/carddisp.pl?gene=MK280073-111
MK280073-112		RNA Gene		3	GC12P058709	6.97178	https://www.genecards.org/cgi-bin/carddisp.pl?gene=MK280073-112
MK280073-113		RNA Gene		3	GC12M058880	6.97178	https://www.genecards.org/cgi-bin/carddisp.pl?gene=MK280073-113
MK280073-114		RNA Gene		3	GC12M060796	6.97178	https://www.genecards.org/cgi-bin/carddisp.pl?gene=MK280073-114
MK280073-116		RNA Gene		3	GC12M070474	6.97178	https://www.genecards.org/cgi-bin/carddisp.pl?gene=MK280073-116
MK280073-118		RNA Gene		3	GC12P086845	6.97178	https://www.genecards.org/cgi-bin/carddisp.pl?gene=MK280073-118
MK280073-119		RNA Gene		3	GC12M088712	6.97178	https://www.genecards.org/cgi-bin/carddisp.pl?gene=MK280073-119
MK280073-122		RNA Gene		3	GC12M096727	6.97178	https://www.genecards.org/cgi-bin/carddisp.pl?gene=MK280073-122
MK280073-124		RNA Gene		3	GC13P018186	6.97178	https://www.genecards.org/cgi-bin/carddisp.pl?gene=MK280073-124
MK280073-125		RNA Gene		3	GC13P021757	6.97178	https://www.genecards.org/cgi-bin/carddisp.pl?gene=MK280073-125
MK280073-126		RNA Gene		3	GC13M038941	6.97178	https://www.genecards.org/cgi-bin/carddisp.pl?gene=MK280073-126
MK280073-127		RNA Gene		3	GC13M038942	6.97178	https://www.genecards.org/cgi-bin/carddisp.pl?gene=MK280073-127

MK280073-128		RNA Gene		3	GC13P034388	6.97178	https://www.genecards.org/cgi-bin/carddisp.pl?gene=MK280073-128
MK280073-131		RNA Gene		3	GC13P038291	6.97178	https://www.genecards.org/cgi-bin/carddisp.pl?gene=MK280073-131
MK280073-132		RNA Gene		3	GC13M045893	6.97178	https://www.genecards.org/cgi-bin/carddisp.pl?gene=MK280073-132
MK280073-133		RNA Gene		3	GC13P047717	6.97178	https://www.genecards.org/cgi-bin/carddisp.pl?gene=MK280073-133
MK280073-134		RNA Gene		3	GC13M050559	6.97178	https://www.genecards.org/cgi-bin/carddisp.pl?gene=MK280073-134
MK280073-135		RNA Gene		3	GC13M051697	6.97178	https://www.genecards.org/cgi-bin/carddisp.pl?gene=MK280073-135
MK280073-136		RNA Gene		3	GC13P052272	6.97178	https://www.genecards.org/cgi-bin/carddisp.pl?gene=MK280073-136
MK280073-137		RNA Gene		3	GC13M052564	6.97178	https://www.genecards.org/cgi-bin/carddisp.pl?gene=MK280073-137
MK280073-138		RNA Gene		3	GC13M053599	6.97178	https://www.genecards.org/cgi-bin/carddisp.pl?gene=MK280073-138
MK280073-139		RNA Gene		3	GC13P054874	6.97178	https://www.genecards.org/cgi-bin/carddisp.pl?gene=MK280073-139
MK280073-141		RNA Gene		3	GC13M071388	6.97178	https://www.genecards.org/cgi-bin/carddisp.pl?gene=MK280073-141
MK280073-142		RNA Gene		3	GC13P072614	6.97178	https://www.genecards.org/cgi-bin/carddisp.pl?gene=MK280073-142

MK280073-143		RNA Gene		3	GC13P078529	6.97178	https://www.genecards.org/cgi-bin/carddisp.pl?gene=MK280073-143
MK280073-144		RNA Gene		3	GC13M079822	6.97178	https://www.genecards.org/cgi-bin/carddisp.pl?gene=MK280073-144
MK280073-145		RNA Gene		3	GC13P079843	6.97178	https://www.genecards.org/cgi-bin/carddisp.pl?gene=MK280073-145
MK280073-146		RNA Gene		3	GC13M081068	6.97178	https://www.genecards.org/cgi-bin/carddisp.pl?gene=MK280073-146
MK280073-147		RNA Gene		3	GC13M091972	6.97178	https://www.genecards.org/cgi-bin/carddisp.pl?gene=MK280073-147
MK280073-148		RNA Gene		3	GC13P092284	6.97178	https://www.genecards.org/cgi-bin/carddisp.pl?gene=MK280073-148
MK280073-151		RNA Gene		3	GC13P106217	6.97178	https://www.genecards.org/cgi-bin/carddisp.pl?gene=MK280073-151
MK280073-152		RNA Gene		3	GC13M106782	6.97178	https://www.genecards.org/cgi-bin/carddisp.pl?gene=MK280073-152
MK280073-154		RNA Gene		3	GC13P111137	6.97178	https://www.genecards.org/cgi-bin/carddisp.pl?gene=MK280073-154
MK280073-155		RNA Gene		3	GC14P041084	6.97178	https://www.genecards.org/cgi-bin/carddisp.pl?gene=MK280073-155
MK280073-156		RNA Gene		3	GC14P041085	6.97178	https://www.genecards.org/cgi-bin/carddisp.pl?gene=MK280073-156
MK280073-157		RNA Gene		3	GC14M025888	6.97178	https://www.genecards.org/cgi-bin/carddisp.pl?gene=MK280073-157

MK280073-158		RNA Gene		3	GC14M025889	6.97178	https://www.genecards.org/cgi-bin/carddisp.pl?gene=MK280073-158
MK280073-159		RNA Gene		3	GC14P041086	6.97178	https://www.genecards.org/cgi-bin/carddisp.pl?gene=MK280073-159
MK280073-160		RNA Gene		3	GC14M025837	6.97178	https://www.genecards.org/cgi-bin/carddisp.pl?gene=MK280073-160
MK280073-161		RNA Gene		3	GC14P041087	6.97178	https://www.genecards.org/cgi-bin/carddisp.pl?gene=MK280073-161
MK280073-162		RNA Gene		3	GC14M028639	6.97178	https://www.genecards.org/cgi-bin/carddisp.pl?gene=MK280073-162
MK280073-163		RNA Gene		3	GC14M029006	6.97178	https://www.genecards.org/cgi-bin/carddisp.pl?gene=MK280073-163
MK280073-165		RNA Gene		3	GC14P041089	6.97178	https://www.genecards.org/cgi-bin/carddisp.pl?gene=MK280073-165
MK280073-168		RNA Gene		3	GC14P040404	6.97178	https://www.genecards.org/cgi-bin/carddisp.pl?gene=MK280073-168
MK280073-169		RNA Gene		3	GC14P040409	6.97178	https://www.genecards.org/cgi-bin/carddisp.pl?gene=MK280073-169
MK280073-170		RNA Gene		3	GC14P040410	6.97178	https://www.genecards.org/cgi-bin/carddisp.pl?gene=MK280073-170
MK280073-171		RNA Gene		3	GC14M039357	6.97178	https://www.genecards.org/cgi-bin/carddisp.pl?gene=MK280073-171
MK280073-172		RNA Gene		3	GC14M046099	6.97178	https://www.genecards.org/cgi-bin/carddisp.pl?gene=MK280073-172

MK280073-173		RNA Gene		3	GC14M046181	6.97178	https://www.genecards.org/cgi-bin/carddisp.pl?gene=MK280073-173
MK280073-174		RNA Gene		3	GC14M049148	6.97178	https://www.genecards.org/cgi-bin/carddisp.pl?gene=MK280073-174
MK280073-177		RNA Gene		3	GC14P056672	6.97178	https://www.genecards.org/cgi-bin/carddisp.pl?gene=MK280073-177
MK280073-178		RNA Gene		3	GC14P058044	6.97178	https://www.genecards.org/cgi-bin/carddisp.pl?gene=MK280073-178
MK280073-179		RNA Gene		3	GC14P062288	6.97178	https://www.genecards.org/cgi-bin/carddisp.pl?gene=MK280073-179
MK280073-181		RNA Gene		3	GC14M071894	6.97178	https://www.genecards.org/cgi-bin/carddisp.pl?gene=MK280073-181
MK280073-183		RNA Gene		3	GC14M085022	6.97178	https://www.genecards.org/cgi-bin/carddisp.pl?gene=MK280073-183
MK280073-184		RNA Gene		3	GC14M117059	6.97178	https://www.genecards.org/cgi-bin/carddisp.pl?gene=MK280073-184
MK280073-185		RNA Gene		3	GC14M117060	6.97178	https://www.genecards.org/cgi-bin/carddisp.pl?gene=MK280073-185
MK280073-186		RNA Gene		3	GC15M020036	6.97178	https://www.genecards.org/cgi-bin/carddisp.pl?gene=MK280073-186
MK280073-187		RNA Gene		3	GC15P056388	6.97178	https://www.genecards.org/cgi-bin/carddisp.pl?gene=MK280073-187
MK280073-188		RNA Gene		3	GC15P059563	6.97178	https://www.genecards.org/cgi-bin/carddisp.pl?gene=MK280073-188

MK280073-189		RNA Gene		3	GC15P059564	6.97178	https://www.genecards.org/cgi-bin/carddisp.pl?gene=MK280073-189
MK280073-190		RNA Gene		3	GC15P059565	6.97178	https://www.genecards.org/cgi-bin/carddisp.pl?gene=MK280073-190
MK280073-191		RNA Gene		3	GC15P059566	6.97178	https://www.genecards.org/cgi-bin/carddisp.pl?gene=MK280073-191
MK280073-192		RNA Gene		3	GC15M050988	6.97178	https://www.genecards.org/cgi-bin/carddisp.pl?gene=MK280073-192
MK280073-193		RNA Gene		3	GC15P057633	6.97178	https://www.genecards.org/cgi-bin/carddisp.pl?gene=MK280073-193
MK280073-194		RNA Gene		3	GC15P057638	6.97178	https://www.genecards.org/cgi-bin/carddisp.pl?gene=MK280073-194
MK280073-195		RNA Gene		3	GC15M138477	6.97178	https://www.genecards.org/cgi-bin/carddisp.pl?gene=MK280073-195
MK280073-196		RNA Gene		3	GC15M138478	6.97178	https://www.genecards.org/cgi-bin/carddisp.pl?gene=MK280073-196
MK280073-198		RNA Gene		3	GC15M138479	6.97178	https://www.genecards.org/cgi-bin/carddisp.pl?gene=MK280073-198
MK280073-200		RNA Gene		3	GC15M138481	6.97178	https://www.genecards.org/cgi-bin/carddisp.pl?gene=MK280073-200
MK280073-201		RNA Gene		3	GC15P140061	6.97178	https://www.genecards.org/cgi-bin/carddisp.pl?gene=MK280073-201
MK280073-204		RNA Gene		3	GC15M138482	6.97178	https://www.genecards.org/cgi-bin/carddisp.pl?gene=MK280073-204

MK280073-208		RNA Gene		3	GC15M138484	6.97178	https://www.genecards.org/cgi-bin/carddisp.pl?gene=MK280073-208
MK280073-209		RNA Gene		3	GC15P140065	6.97178	https://www.genecards.org/cgi-bin/carddisp.pl?gene=MK280073-209
MK280073-210		RNA Gene		3	GC15M138485	6.97178	https://www.genecards.org/cgi-bin/carddisp.pl?gene=MK280073-210
MK280073-211		RNA Gene		3	GC15M138486	6.97178	https://www.genecards.org/cgi-bin/carddisp.pl?gene=MK280073-211
MK280073-212		RNA Gene		3	GC15P140066	6.97178	https://www.genecards.org/cgi-bin/carddisp.pl?gene=MK280073-212
MK280073-213		RNA Gene		3	GC15M138487	6.97178	https://www.genecards.org/cgi-bin/carddisp.pl?gene=MK280073-213
MK280073-214		RNA Gene		3	GC15M138488	6.97178	https://www.genecards.org/cgi-bin/carddisp.pl?gene=MK280073-214
MK280073-215		RNA Gene		3	GC15P140067	6.97178	https://www.genecards.org/cgi-bin/carddisp.pl?gene=MK280073-215
MK280073-217		RNA Gene		3	GC16M013929	6.97178	https://www.genecards.org/cgi-bin/carddisp.pl?gene=MK280073-217
MK280073-218		RNA Gene		3	GC16M013652	6.97178	https://www.genecards.org/cgi-bin/carddisp.pl?gene=MK280073-218
MK280073-219		RNA Gene		3	GC16M013934	6.97178	https://www.genecards.org/cgi-bin/carddisp.pl?gene=MK280073-219
MK280073-220		RNA Gene		3	GC16M016875	6.97178	https://www.genecards.org/cgi-bin/carddisp.pl?gene=MK280073-220

MK280073-221		RNA Gene		3	GC16M020760	6.97178	https://www.genecards.org/cgi-bin/carddisp.pl?gene=MK280073-221
MK280073-222		RNA Gene		3	GC16M021057	6.97178	https://www.genecards.org/cgi-bin/carddisp.pl?gene=MK280073-222
MK280073-224		RNA Gene		3	GC16P058968	6.97178	https://www.genecards.org/cgi-bin/carddisp.pl?gene=MK280073-224
MK280073-225		RNA Gene		3	GC16M042167	6.97178	https://www.genecards.org/cgi-bin/carddisp.pl?gene=MK280073-225
MK280073-226		RNA Gene		3	GC16P058969	6.97178	https://www.genecards.org/cgi-bin/carddisp.pl?gene=MK280073-226
MK280073-227		RNA Gene		3	GC16P058970	6.97178	https://www.genecards.org/cgi-bin/carddisp.pl?gene=MK280073-227
MK280073-228		RNA Gene		3	GC16P058971	6.97178	https://www.genecards.org/cgi-bin/carddisp.pl?gene=MK280073-228
MK280073-229		RNA Gene		3	GC16P058972	6.97178	https://www.genecards.org/cgi-bin/carddisp.pl?gene=MK280073-229
MK280073-230		RNA Gene		3	GC16M047812	6.97178	https://www.genecards.org/cgi-bin/carddisp.pl?gene=MK280073-230
MK280073-231		RNA Gene		3	GC16M048032	6.97178	https://www.genecards.org/cgi-bin/carddisp.pl?gene=MK280073-231
MK280073-232		RNA Gene		3	GC16M048783	6.97178	https://www.genecards.org/cgi-bin/carddisp.pl?gene=MK280073-232
MK280073-233		RNA Gene		3	GC16P058973	6.97178	https://www.genecards.org/cgi-bin/carddisp.pl?gene=MK280073-233

MK280073-234		RNA Gene		3	GC16M052159	6.97178	https://www.genecards.org/cgi-bin/carddisp.pl?gene=MK280073-234
MK280073-235		RNA Gene		3	GC16P054762	6.97178	https://www.genecards.org/cgi-bin/carddisp.pl?gene=MK280073-235
MK280073-236		RNA Gene		3	GC16P055791	6.97178	https://www.genecards.org/cgi-bin/carddisp.pl?gene=MK280073-236
MK280073-237		RNA Gene		3	GC16P059382	6.97178	https://www.genecards.org/cgi-bin/carddisp.pl?gene=MK280073-237
MK280073-238		RNA Gene		3	GC16M058784	6.97178	https://www.genecards.org/cgi-bin/carddisp.pl?gene=MK280073-238
MK280073-239		RNA Gene		3	GC16P059315	6.97178	https://www.genecards.org/cgi-bin/carddisp.pl?gene=MK280073-239
MK280073-240		RNA Gene		3	GC16M059570	6.97178	https://www.genecards.org/cgi-bin/carddisp.pl?gene=MK280073-240
MK280073-241		RNA Gene		3	GC16M060830	6.97178	https://www.genecards.org/cgi-bin/carddisp.pl?gene=MK280073-241
MK280073-242		RNA Gene		3	GC16P063152	6.97178	https://www.genecards.org/cgi-bin/carddisp.pl?gene=MK280073-242
MK280073-244		RNA Gene		3	GC16M072271	6.97178	https://www.genecards.org/cgi-bin/carddisp.pl?gene=MK280073-244
MK280073-245		RNA Gene		3	GC16P073897	6.97178	https://www.genecards.org/cgi-bin/carddisp.pl?gene=MK280073-245
MK280073-246		RNA Gene		3	GC16P073672	6.97178	https://www.genecards.org/cgi-bin/carddisp.pl?gene=MK280073-246

MK280073-247		RNA Gene		3	GC16P074517	6.97178	https://www.genecards.org/cgi-bin/carddisp.pl?gene=MK280073-247
MK280073-248		RNA Gene		3	GC17M014727	6.97178	https://www.genecards.org/cgi-bin/carddisp.pl?gene=MK280073-248
MK280073-249		RNA Gene		3	GC17M014728	6.97178	https://www.genecards.org/cgi-bin/carddisp.pl?gene=MK280073-249
MK280073-250		RNA Gene		3	GC17P015174	6.97178	https://www.genecards.org/cgi-bin/carddisp.pl?gene=MK280073-250
MK280073-251		RNA Gene		3	GC17M014729	6.97178	https://www.genecards.org/cgi-bin/carddisp.pl?gene=MK280073-251
MK280073-252		RNA Gene		3	GC17P013917	6.97178	https://www.genecards.org/cgi-bin/carddisp.pl?gene=MK280073-252
MK280073-253		RNA Gene		3	GC17M014357	6.97178	https://www.genecards.org/cgi-bin/carddisp.pl?gene=MK280073-253
MK280073-255		RNA Gene		3	GC17M014531	6.97178	https://www.genecards.org/cgi-bin/carddisp.pl?gene=MK280073-255
MK280073-257		RNA Gene		3	GC17M066332	6.97178	https://www.genecards.org/cgi-bin/carddisp.pl?gene=MK280073-257
MK280073-258		RNA Gene		3	GC17P091333	6.97178	https://www.genecards.org/cgi-bin/carddisp.pl?gene=MK280073-258
MK280073-259		RNA Gene		3	GC17M066333	6.97178	https://www.genecards.org/cgi-bin/carddisp.pl?gene=MK280073-259
MK280073-260		RNA Gene		3	GC17M066334	6.97178	https://www.genecards.org/cgi-bin/carddisp.pl?gene=MK280073-260

MK280073-261		RNA Gene		3	GC17P091334	6.97178	https://www.genecards.org/cgi-bin/carddisp.pl?gene=MK280073-261
MK280073-262		RNA Gene		3	GC17P091335	6.97178	https://www.genecards.org/cgi-bin/carddisp.pl?gene=MK280073-262
MK280073-264		RNA Gene		3	GC17P091336	6.97178	https://www.genecards.org/cgi-bin/carddisp.pl?gene=MK280073-264
MK280073-265		RNA Gene		3	GC17M066336	6.97178	https://www.genecards.org/cgi-bin/carddisp.pl?gene=MK280073-265
MK280073-266		RNA Gene		3	GC17M066337	6.97178	https://www.genecards.org/cgi-bin/carddisp.pl?gene=MK280073-266
MK280073-267		RNA Gene		3	GC17M066338	6.97178	https://www.genecards.org/cgi-bin/carddisp.pl?gene=MK280073-267
MK280073-268		RNA Gene		3	GC17P091337	6.97178	https://www.genecards.org/cgi-bin/carddisp.pl?gene=MK280073-268
MK280073-269		RNA Gene		3	GC17P091338	6.97178	https://www.genecards.org/cgi-bin/carddisp.pl?gene=MK280073-269
MK280073-270		RNA Gene		3	GC17P091339	6.97178	https://www.genecards.org/cgi-bin/carddisp.pl?gene=MK280073-270
MK280073-271		RNA Gene		3	GC17P091340	6.97178	https://www.genecards.org/cgi-bin/carddisp.pl?gene=MK280073-271
MK280073-272		RNA Gene		3	GC17M069023	6.97178	https://www.genecards.org/cgi-bin/carddisp.pl?gene=MK280073-272
MK280073-274		RNA Gene		3	GC17M071368	6.97178	https://www.genecards.org/cgi-bin/carddisp.pl?gene=MK280073-274

MK280073-275		RNA Gene		3	GC17P087152	6.97178	https://www.genecards.org/cgi-bin/carddisp.pl?gene=MK280073-275
MK280073-276		RNA Gene		3	GC18M000992	6.97178	https://www.genecards.org/cgi-bin/carddisp.pl?gene=MK280073-276
MK280073-277		RNA Gene		3	GC18P001339	6.97178	https://www.genecards.org/cgi-bin/carddisp.pl?gene=MK280073-277
MK280073-278		RNA Gene		3	GC18M001536	6.97178	https://www.genecards.org/cgi-bin/carddisp.pl?gene=MK280073-278
MK280073-279		RNA Gene		3	GC18P002344	6.97178	https://www.genecards.org/cgi-bin/carddisp.pl?gene=MK280073-279
MK280073-280		RNA Gene		3	GC18M002864	6.97178	https://www.genecards.org/cgi-bin/carddisp.pl?gene=MK280073-280
MK280073-281		RNA Gene		3	GC18M005860	6.97178	https://www.genecards.org/cgi-bin/carddisp.pl?gene=MK280073-281
MK280073-282		RNA Gene		3	GC18P020177	6.97178	https://www.genecards.org/cgi-bin/carddisp.pl?gene=MK280073-282
MK280073-284		RNA Gene		3	GC18P022889	6.97178	https://www.genecards.org/cgi-bin/carddisp.pl?gene=MK280073-284
MK280073-285		RNA Gene		3	GC18P032398	6.97178	https://www.genecards.org/cgi-bin/carddisp.pl?gene=MK280073-285
MK280073-286		RNA Gene		3	GC18P034898	6.97178	https://www.genecards.org/cgi-bin/carddisp.pl?gene=MK280073-286
MK280073-287		RNA Gene		3	GC18P035764	6.97178	https://www.genecards.org/cgi-bin/carddisp.pl?gene=MK280073-287

MK280073-288		RNA Gene		3	GC18P035795	6.97178	https://www.genecards.org/cgi-bin/carddisp.pl?gene=MK280073-288
MK280073-289		RNA Gene		3	GC18P037077	6.97178	https://www.genecards.org/cgi-bin/carddisp.pl?gene=MK280073-289
MK280073-290		RNA Gene		3	GC18M037928	6.97178	https://www.genecards.org/cgi-bin/carddisp.pl?gene=MK280073-290
MK280073-291		RNA Gene		3	GC18P042664	6.97178	https://www.genecards.org/cgi-bin/carddisp.pl?gene=MK280073-291
MK280073-292		RNA Gene		3	GC18P043289	6.97178	https://www.genecards.org/cgi-bin/carddisp.pl?gene=MK280073-292
MK280073-293		RNA Gene		3	GC18P044445	6.97178	https://www.genecards.org/cgi-bin/carddisp.pl?gene=MK280073-293
MK280073-294		RNA Gene		3	GC18P046745	6.97178	https://www.genecards.org/cgi-bin/carddisp.pl?gene=MK280073-294
MK280073-297		RNA Gene		3	GC18M052416	6.97178	https://www.genecards.org/cgi-bin/carddisp.pl?gene=MK280073-297
MK280073-298		RNA Gene		3	GC18M054403	6.97178	https://www.genecards.org/cgi-bin/carddisp.pl?gene=MK280073-298
MK280073-299		RNA Gene		3	GC18M054573	6.97178	https://www.genecards.org/cgi-bin/carddisp.pl?gene=MK280073-299
MK280073-301		RNA Gene		3	GC18P057227	6.97178	https://www.genecards.org/cgi-bin/carddisp.pl?gene=MK280073-301
MK280073-302		RNA Gene		3	GC18M060801	6.97178	https://www.genecards.org/cgi-bin/carddisp.pl?gene=MK280073-302

MK280073-303		RNA Gene		3	GC18M062052	6.97178	https://www.genecards.org/cgi-bin/carddisp.pl?gene=MK280073-303
MK280073-304		RNA Gene		3	GC18P070739	6.97178	https://www.genecards.org/cgi-bin/carddisp.pl?gene=MK280073-304
MK280073-305		RNA Gene		3	GC18M070858	6.97178	https://www.genecards.org/cgi-bin/carddisp.pl?gene=MK280073-305
MK280073-306		RNA Gene		3	GC18M077536	6.97178	https://www.genecards.org/cgi-bin/carddisp.pl?gene=MK280073-306
MK280073-307		RNA Gene		3	GC18M079288	6.97178	https://www.genecards.org/cgi-bin/carddisp.pl?gene=MK280073-307
MK280073-308		RNA Gene		3	GC19M011418	6.97178	https://www.genecards.org/cgi-bin/carddisp.pl?gene=MK280073-308
MK280073-309		RNA Gene		3	GC19P088859	6.97178	https://www.genecards.org/cgi-bin/carddisp.pl?gene=MK280073-309
MK280073-310		RNA Gene		3	GC19M016259	6.97178	https://www.genecards.org/cgi-bin/carddisp.pl?gene=MK280073-310
MK280073-311		RNA Gene		3	GC19M016001	6.97178	https://www.genecards.org/cgi-bin/carddisp.pl?gene=MK280073-311
MK280073-312		RNA Gene		3	GC19M084122	6.97178	https://www.genecards.org/cgi-bin/carddisp.pl?gene=MK280073-312
MK280073-313		RNA Gene		3	GC19M084123	6.97178	https://www.genecards.org/cgi-bin/carddisp.pl?gene=MK280073-313
MK280073-314		RNA Gene		3	GC19P093537	6.97178	https://www.genecards.org/cgi-bin/carddisp.pl?gene=MK280073-314

MK280073-315		RNA Gene		3	GC19M084124	6.97178	https://www.genecards.org/cgi-bin/carddisp.pl?gene=MK280073-315
MK280073-316		RNA Gene		3	GC19P093538	6.97178	https://www.genecards.org/cgi-bin/carddisp.pl?gene=MK280073-316
MK280073-317		RNA Gene		3	GC19P093539	6.97178	https://www.genecards.org/cgi-bin/carddisp.pl?gene=MK280073-317
MK280073-318		RNA Gene		3	GC19P093540	6.97178	https://www.genecards.org/cgi-bin/carddisp.pl?gene=MK280073-318
MK280073-319		RNA Gene		3	GC19M084125	6.97178	https://www.genecards.org/cgi-bin/carddisp.pl?gene=MK280073-319
MK280073-320		RNA Gene		3	GC19M084126	6.97178	https://www.genecards.org/cgi-bin/carddisp.pl?gene=MK280073-320
MK280073-321		RNA Gene		3	GC19M084127	6.97178	https://www.genecards.org/cgi-bin/carddisp.pl?gene=MK280073-321
MK280073-322		RNA Gene		3	GC19M084128	6.97178	https://www.genecards.org/cgi-bin/carddisp.pl?gene=MK280073-322
MK280073-323		RNA Gene		3	GC19P093541	6.97178	https://www.genecards.org/cgi-bin/carddisp.pl?gene=MK280073-323
MK280073-324		RNA Gene		3	GC19M084129	6.97178	https://www.genecards.org/cgi-bin/carddisp.pl?gene=MK280073-324
MK280073-325		RNA Gene		3	GC19P093542	6.97178	https://www.genecards.org/cgi-bin/carddisp.pl?gene=MK280073-325
MK280073-326		RNA Gene		3	GC19P093543	6.97178	https://www.genecards.org/cgi-bin/carddisp.pl?gene=MK280073-326

MK280073-328		RNA Gene		3	GC19M084131	6.97178	https://www.genecards.org/cgi-bin/carddisp.pl?gene=MK280073-328
MK280073-329		RNA Gene		3	GC19P093544	6.97178	https://www.genecards.org/cgi-bin/carddisp.pl?gene=MK280073-329
MK280073-330		RNA Gene		3	GC19M084132	6.97178	https://www.genecards.org/cgi-bin/carddisp.pl?gene=MK280073-330
MK280073-331		RNA Gene		3	GC19P093545	6.97178	https://www.genecards.org/cgi-bin/carddisp.pl?gene=MK280073-331
MK280073-332		RNA Gene		3	GC19P093546	6.97178	https://www.genecards.org/cgi-bin/carddisp.pl?gene=MK280073-332
MK280073-333		RNA Gene		3	GC19P093547	6.97178	https://www.genecards.org/cgi-bin/carddisp.pl?gene=MK280073-333
MK280073-335		RNA Gene		3	GC19P093549	6.97178	https://www.genecards.org/cgi-bin/carddisp.pl?gene=MK280073-335
MK280073-336		RNA Gene		3	GC02P003086	6.97178	https://www.genecards.org/cgi-bin/carddisp.pl?gene=MK280073-336
MK280073-337		RNA Gene		3	GC02P003373	6.97178	https://www.genecards.org/cgi-bin/carddisp.pl?gene=MK280073-337
MK280073-338		RNA Gene		3	GC02M004413	6.97178	https://www.genecards.org/cgi-bin/carddisp.pl?gene=MK280073-338
MK280073-339		RNA Gene		3	GC02P004653	6.97178	https://www.genecards.org/cgi-bin/carddisp.pl?gene=MK280073-339
MK280073-340		RNA Gene		3	GC02P008130	6.97178	https://www.genecards.org/cgi-bin/carddisp.pl?gene=MK280073-340

MK280073-341		RNA Gene		3	GC02M014506	6.97178	https://www.genecards.org/cgi-bin/carddisp.pl?gene=MK280073-341
MK280073-342		RNA Gene		3	GC02P016879	6.97178	https://www.genecards.org/cgi-bin/carddisp.pl?gene=MK280073-342
MK280073-343		RNA Gene		3	GC02M021908	6.97178	https://www.genecards.org/cgi-bin/carddisp.pl?gene=MK280073-343
MK280073-344		RNA Gene		3	GC02P033199	6.97178	https://www.genecards.org/cgi-bin/carddisp.pl?gene=MK280073-344
MK280073-345		RNA Gene		3	GC02M035323	6.97178	https://www.genecards.org/cgi-bin/carddisp.pl?gene=MK280073-345
MK280073-347		RNA Gene		3	GC02M063091	6.97178	https://www.genecards.org/cgi-bin/carddisp.pl?gene=MK280073-347
MK280073-348		RNA Gene		3	GC02M066351	6.97178	https://www.genecards.org/cgi-bin/carddisp.pl?gene=MK280073-348
MK280073-349		RNA Gene		3	GC02M076652	6.97178	https://www.genecards.org/cgi-bin/carddisp.pl?gene=MK280073-349
MK280073-350		RNA Gene		3	GC02M076726	6.97178	https://www.genecards.org/cgi-bin/carddisp.pl?gene=MK280073-350
MK280073-352		RNA Gene		3	GC02M100055	6.97178	https://www.genecards.org/cgi-bin/carddisp.pl?gene=MK280073-352
MK280073-355		RNA Gene		3	GC02M149406	6.97178	https://www.genecards.org/cgi-bin/carddisp.pl?gene=MK280073-355
MK280073-356		RNA Gene		3	GC02P155969	6.97178	https://www.genecards.org/cgi-bin/carddisp.pl?gene=MK280073-356

MK280073-357		RNA Gene		3	GC02M156966	6.97178	https://www.genecards.org/cgi-bin/carddisp.pl?gene=MK280073-357
MK280073-358		RNA Gene		3	GC02M157101	6.97178	https://www.genecards.org/cgi-bin/carddisp.pl?gene=MK280073-358
MK280073-360		RNA Gene		3	GC02P158226	6.97178	https://www.genecards.org/cgi-bin/carddisp.pl?gene=MK280073-360
MK280073-361		RNA Gene		3	GC02M179756	6.97178	https://www.genecards.org/cgi-bin/carddisp.pl?gene=MK280073-361
MK280073-362		RNA Gene		3	GC02P183795	6.97178	https://www.genecards.org/cgi-bin/carddisp.pl?gene=MK280073-362
MK280073-363		RNA Gene		3	GC02P210354	6.97178	https://www.genecards.org/cgi-bin/carddisp.pl?gene=MK280073-363
MK280073-364		RNA Gene		3	GC02P213678	6.97178	https://www.genecards.org/cgi-bin/carddisp.pl?gene=MK280073-364
MK280073-365		RNA Gene		3	GC02P216458	6.97178	https://www.genecards.org/cgi-bin/carddisp.pl?gene=MK280073-365
MK280073-366		RNA Gene		3	GC02P223015	6.97178	https://www.genecards.org/cgi-bin/carddisp.pl?gene=MK280073-366
MK280073-367		RNA Gene		3	GC02M237847	6.97178	https://www.genecards.org/cgi-bin/carddisp.pl?gene=MK280073-367
MK280073-368		RNA Gene		3	GC20P001220	6.97178	https://www.genecards.org/cgi-bin/carddisp.pl?gene=MK280073-368
MK280073-369		RNA Gene		3	GC20M003887	6.97178	https://www.genecards.org/cgi-bin/carddisp.pl?gene=MK280073-369

MK280073-370		RNA Gene		3	GC20M004287	6.97178	https://www.genecards.org/cgi-bin/carddisp.pl?gene=MK280073-370
MK280073-371		RNA Gene		3	GC20P007114	6.97178	https://www.genecards.org/cgi-bin/carddisp.pl?gene=MK280073-371
MK280073-372		RNA Gene		3	GC20M009276	6.97178	https://www.genecards.org/cgi-bin/carddisp.pl?gene=MK280073-372
MK280073-373		RNA Gene		3	GC20M009277	6.97178	https://www.genecards.org/cgi-bin/carddisp.pl?gene=MK280073-373
MK280073-374		RNA Gene		3	GC20M009278	6.97178	https://www.genecards.org/cgi-bin/carddisp.pl?gene=MK280073-374
MK280073-375		RNA Gene		3	GC20M012128	6.97178	https://www.genecards.org/cgi-bin/carddisp.pl?gene=MK280073-375
MK280073-376		RNA Gene		3	GC20M012363	6.97178	https://www.genecards.org/cgi-bin/carddisp.pl?gene=MK280073-376
MK280073-377		RNA Gene		3	GC20M012454	6.97178	https://www.genecards.org/cgi-bin/carddisp.pl?gene=MK280073-377
MK280073-378		RNA Gene		3	GC20P012495	6.97178	https://www.genecards.org/cgi-bin/carddisp.pl?gene=MK280073-378
MK280073-379		RNA Gene		3	GC20P014274	6.97178	https://www.genecards.org/cgi-bin/carddisp.pl?gene=MK280073-379
MK280073-380		RNA Gene		3	GC20M014465	6.97178	https://www.genecards.org/cgi-bin/carddisp.pl?gene=MK280073-380
MK280073-381		RNA Gene		3	GC20P015135	6.97178	https://www.genecards.org/cgi-bin/carddisp.pl?gene=MK280073-381

MK280073-382		RNA Gene		3	GC20M018873	6.97178	https://www.genecards.org/cgi-bin/carddisp.pl?gene=MK280073-382
MK280073-383		RNA Gene		3	GC20M021487	6.97178	https://www.genecards.org/cgi-bin/carddisp.pl?gene=MK280073-383
MK280073-384		RNA Gene		3	GC20M021956	6.97178	https://www.genecards.org/cgi-bin/carddisp.pl?gene=MK280073-384
MK280073-385		RNA Gene		3	GC20P021930	6.97178	https://www.genecards.org/cgi-bin/carddisp.pl?gene=MK280073-385
MK280073-386		RNA Gene		3	GC20P022013	6.97178	https://www.genecards.org/cgi-bin/carddisp.pl?gene=MK280073-386
MK280073-387		RNA Gene		3	GC20P023622	6.97178	https://www.genecards.org/cgi-bin/carddisp.pl?gene=MK280073-387
MK280073-388		RNA Gene		3	GC20M023901	6.97178	https://www.genecards.org/cgi-bin/carddisp.pl?gene=MK280073-388
MK280073-390		RNA Gene		3	GC20P024990	6.97178	https://www.genecards.org/cgi-bin/carddisp.pl?gene=MK280073-390
MK280073-391		RNA Gene		3	GC20P025544	6.97178	https://www.genecards.org/cgi-bin/carddisp.pl?gene=MK280073-391
MK280073-395		RNA Gene		3	GC20P031337	6.97178	https://www.genecards.org/cgi-bin/carddisp.pl?gene=MK280073-395
MK280073-396		RNA Gene		3	GC20P041208	6.97178	https://www.genecards.org/cgi-bin/carddisp.pl?gene=MK280073-396
MK280073-397		RNA Gene		3	GC20M042068	6.97178	https://www.genecards.org/cgi-bin/carddisp.pl?gene=MK280073-397

MK280073-398		RNA Gene		3	GC20P054789	6.97178	https://www.genecards.org/cgi-bin/carddisp.pl?gene=MK280073-398
MK280073-399		RNA Gene		3	GC20M055278	6.97178	https://www.genecards.org/cgi-bin/carddisp.pl?gene=MK280073-399
MK280073-402		RNA Gene		3	GC21P015797	6.97178	https://www.genecards.org/cgi-bin/carddisp.pl?gene=MK280073-402
MK280073-403		RNA Gene		3	GC21M010354	6.97178	https://www.genecards.org/cgi-bin/carddisp.pl?gene=MK280073-403
MK280073-404		RNA Gene		3	GC21P015798	6.97178	https://www.genecards.org/cgi-bin/carddisp.pl?gene=MK280073-404
MK280073-405		RNA Gene		3	GC21M015128	6.97178	https://www.genecards.org/cgi-bin/carddisp.pl?gene=MK280073-405
MK280073-406		RNA Gene		3	GC21P015799	6.97178	https://www.genecards.org/cgi-bin/carddisp.pl?gene=MK280073-406
MK280073-407		RNA Gene		3	GC21P015355	6.97178	https://www.genecards.org/cgi-bin/carddisp.pl?gene=MK280073-407
MK280073-409		RNA Gene		3	GC21M015918	6.97178	https://www.genecards.org/cgi-bin/carddisp.pl?gene=MK280073-409
MK280073-410		RNA Gene		3	GC21P017166	6.97178	https://www.genecards.org/cgi-bin/carddisp.pl?gene=MK280073-410
MK280073-411		RNA Gene		3	GC21M018438	6.97178	https://www.genecards.org/cgi-bin/carddisp.pl?gene=MK280073-411
MK280073-412		RNA Gene		3	GC21M018896	6.97178	https://www.genecards.org/cgi-bin/carddisp.pl?gene=MK280073-412

MK280073-413		RNA Gene		3	GC21P019814	6.97178	https://www.genecards.org/cgi-bin/carddisp.pl?gene=MK280073-413
MK280073-414		RNA Gene		3	GC21M021709	6.97178	https://www.genecards.org/cgi-bin/carddisp.pl?gene=MK280073-414
MK280073-415		RNA Gene		3	GC21P025923	6.97178	https://www.genecards.org/cgi-bin/carddisp.pl?gene=MK280073-415
MK280073-416		RNA Gene		3	GC21P028763	6.97178	https://www.genecards.org/cgi-bin/carddisp.pl?gene=MK280073-416
MK280073-417		RNA Gene		3	GC21M033988	6.97178	https://www.genecards.org/cgi-bin/carddisp.pl?gene=MK280073-417
MK280073-418		RNA Gene		3	GC21M035497	6.97178	https://www.genecards.org/cgi-bin/carddisp.pl?gene=MK280073-418
MK280073-419		RNA Gene		3	GC21P036620	6.97178	https://www.genecards.org/cgi-bin/carddisp.pl?gene=MK280073-419
MK280073-420		RNA Gene		3	GC21P037112	6.97178	https://www.genecards.org/cgi-bin/carddisp.pl?gene=MK280073-420
MK280073-427		RNA Gene		3	GC22P015499	6.97178	https://www.genecards.org/cgi-bin/carddisp.pl?gene=MK280073-427
MK280073-428		RNA Gene		3	GC22P059635	6.97178	https://www.genecards.org/cgi-bin/carddisp.pl?gene=MK280073-428
MK280073-430		RNA Gene		3	GC22P059637	6.97178	https://www.genecards.org/cgi-bin/carddisp.pl?gene=MK280073-430
MK280073-431		RNA Gene		3	GC22P059638	6.97178	https://www.genecards.org/cgi-bin/carddisp.pl?gene=MK280073-431

MK280073-432		RNA Gene		3	GC22M017297	6.97178	https://www.genecards.org/cgi-bin/carddisp.pl?gene=MK280073-432
MK280073-433		RNA Gene		3	GC22P059639	6.97178	https://www.genecards.org/cgi-bin/carddisp.pl?gene=MK280073-433
MK280073-434		RNA Gene		3	GC22P059640	6.97178	https://www.genecards.org/cgi-bin/carddisp.pl?gene=MK280073-434
MK280073-435		RNA Gene		3	GC22P059641	6.97178	https://www.genecards.org/cgi-bin/carddisp.pl?gene=MK280073-435
MK280073-436		RNA Gene		3	GC22P059642	6.97178	https://www.genecards.org/cgi-bin/carddisp.pl?gene=MK280073-436
MK280073-437		RNA Gene		3	GC22M071232	6.97178	https://www.genecards.org/cgi-bin/carddisp.pl?gene=MK280073-437
MK280073-438		RNA Gene		3	GC22M071233	6.97178	https://www.genecards.org/cgi-bin/carddisp.pl?gene=MK280073-438
MK280073-439		RNA Gene		3	GC22P059643	6.97178	https://www.genecards.org/cgi-bin/carddisp.pl?gene=MK280073-439
MK280073-440		RNA Gene		3	GC22M071234	6.97178	https://www.genecards.org/cgi-bin/carddisp.pl?gene=MK280073-440
MK280073-441		RNA Gene		3	GC22M071235	6.97178	https://www.genecards.org/cgi-bin/carddisp.pl?gene=MK280073-441
MK280073-442		RNA Gene		3	GC22M071236	6.97178	https://www.genecards.org/cgi-bin/carddisp.pl?gene=MK280073-442
MK280073-443		RNA Gene		3	GC22P059644	6.97178	https://www.genecards.org/cgi-bin/carddisp.pl?gene=MK280073-443

MK280073-444		RNA Gene		3	GC22M071237	6.97178	https://www.genecards.org/cgi-bin/carddisp.pl?gene=MK280073-444
MK280073-445		RNA Gene		3	GC03M002428	6.97178	https://www.genecards.org/cgi-bin/carddisp.pl?gene=MK280073-445
MK280073-446		RNA Gene		3	GC03M002670	6.97178	https://www.genecards.org/cgi-bin/carddisp.pl?gene=MK280073-446
MK280073-447		RNA Gene		3	GC03P008925	6.97178	https://www.genecards.org/cgi-bin/carddisp.pl?gene=MK280073-447
MK280073-448		RNA Gene		3	GC03M011941	6.97178	https://www.genecards.org/cgi-bin/carddisp.pl?gene=MK280073-448
MK280073-449		RNA Gene		3	GC03M024722	6.97178	https://www.genecards.org/cgi-bin/carddisp.pl?gene=MK280073-449
MK280073-450		RNA Gene		3	GC03P019789	6.97178	https://www.genecards.org/cgi-bin/carddisp.pl?gene=MK280073-450
MK280073-451		RNA Gene		3	GC03P026886	6.97178	https://www.genecards.org/cgi-bin/carddisp.pl?gene=MK280073-451
MK280073-452		RNA Gene		3	GC03P035230	6.97178	https://www.genecards.org/cgi-bin/carddisp.pl?gene=MK280073-452
MK280073-454		RNA Gene		3	GC03P042243	6.97178	https://www.genecards.org/cgi-bin/carddisp.pl?gene=MK280073-454
MK280073-455		RNA Gene		3	GC03P061366	6.97178	https://www.genecards.org/cgi-bin/carddisp.pl?gene=MK280073-455
MK280073-457		RNA Gene		3	GC03M074454	6.97178	https://www.genecards.org/cgi-bin/carddisp.pl?gene=MK280073-457

MK280073-458		RNA Gene		3	GC03P076396	6.97178	https://www.genecards.org/cgi-bin/carddisp.pl?gene=MK280073-458
MK280073-459		RNA Gene		3	GC03P098410	6.97178	https://www.genecards.org/cgi-bin/carddisp.pl?gene=MK280073-459
MK280073-462		RNA Gene		3	GC03M109512	6.97178	https://www.genecards.org/cgi-bin/carddisp.pl?gene=MK280073-462
MK280073-463		RNA Gene		3	GC03M120377	6.97178	https://www.genecards.org/cgi-bin/carddisp.pl?gene=MK280073-463
MK280073-465		RNA Gene		3	GC03P137242	6.97178	https://www.genecards.org/cgi-bin/carddisp.pl?gene=MK280073-465
MK280073-466		RNA Gene		3	GC03P139594	6.97178	https://www.genecards.org/cgi-bin/carddisp.pl?gene=MK280073-466
MK280073-467		RNA Gene		3	GC03P140991	6.97178	https://www.genecards.org/cgi-bin/carddisp.pl?gene=MK280073-467
MK280073-469		RNA Gene		3	GC03M157195	6.97178	https://www.genecards.org/cgi-bin/carddisp.pl?gene=MK280073-469
MK280073-471		RNA Gene		3	GC03M170693	6.97178	https://www.genecards.org/cgi-bin/carddisp.pl?gene=MK280073-471
MK280073-472		RNA Gene		3	GC03P171628	6.97178	https://www.genecards.org/cgi-bin/carddisp.pl?gene=MK280073-472
MK280073-474		RNA Gene		3	GC03M175788	6.97178	https://www.genecards.org/cgi-bin/carddisp.pl?gene=MK280073-474
MK280073-476		RNA Gene		3	GC04P002231	6.97178	https://www.genecards.org/cgi-bin/carddisp.pl?gene=MK280073-476

MK280073-477		RNA Gene		3	GC04M005066	6.97178	https://www.genecards.org/cgi-bin/carddisp.pl?gene=MK280073-477
MK280073-478		RNA Gene		3	GC04M005175	6.97178	https://www.genecards.org/cgi-bin/carddisp.pl?gene=MK280073-478
MK280073-479		RNA Gene		3	GC04M005541	6.97178	https://www.genecards.org/cgi-bin/carddisp.pl?gene=MK280073-479
MK280073-480		RNA Gene		3	GC04M014424	6.97178	https://www.genecards.org/cgi-bin/carddisp.pl?gene=MK280073-480
MK280073-481		RNA Gene		3	GC04M018602	6.97178	https://www.genecards.org/cgi-bin/carddisp.pl?gene=MK280073-481
MK280073-482		RNA Gene		3	GC04P022683	6.97178	https://www.genecards.org/cgi-bin/carddisp.pl?gene=MK280073-482
MK280073-483		RNA Gene		3	GC04P022298	6.97178	https://www.genecards.org/cgi-bin/carddisp.pl?gene=MK280073-483
MK280073-484		RNA Gene		3	GC04P022467	6.97178	https://www.genecards.org/cgi-bin/carddisp.pl?gene=MK280073-484
MK280073-485		RNA Gene		3	GC04P024942	6.97178	https://www.genecards.org/cgi-bin/carddisp.pl?gene=MK280073-485
MK280073-486		RNA Gene		3	GC04P037274	6.97178	https://www.genecards.org/cgi-bin/carddisp.pl?gene=MK280073-486
MK280073-487		RNA Gene		3	GC04M037942	6.97178	https://www.genecards.org/cgi-bin/carddisp.pl?gene=MK280073-487
MK280073-488		RNA Gene		3	GC04P039250	6.97178	https://www.genecards.org/cgi-bin/carddisp.pl?gene=MK280073-488

MK280073-491		RNA Gene		3	GC04P059185	6.97178	https://www.genecards.org/cgi-bin/carddisp.pl?gene=MK280073-491
MK280073-492		RNA Gene		3	GC04P059210	6.97178	https://www.genecards.org/cgi-bin/carddisp.pl?gene=MK280073-492
MK280073-494		RNA Gene		3	GC04P097781	6.97178	https://www.genecards.org/cgi-bin/carddisp.pl?gene=MK280073-494
MK280073-495		RNA Gene		3	GC04M099115	6.97178	https://www.genecards.org/cgi-bin/carddisp.pl?gene=MK280073-495
MK280073-496		RNA Gene		3	GC04P117926	6.97178	https://www.genecards.org/cgi-bin/carddisp.pl?gene=MK280073-496
MK280073-497		RNA Gene		3	GC04M122943	6.97178	https://www.genecards.org/cgi-bin/carddisp.pl?gene=MK280073-497
MK280073-498		RNA Gene		3	GC04M123002	6.97178	https://www.genecards.org/cgi-bin/carddisp.pl?gene=MK280073-498
MK280073-499		RNA Gene		3	GC04P158477	6.97178	https://www.genecards.org/cgi-bin/carddisp.pl?gene=MK280073-499
MK280073-500		RNA Gene		3	GC04P164547	6.97178	https://www.genecards.org/cgi-bin/carddisp.pl?gene=MK280073-500
MK280073-502		RNA Gene		3	GC04P187054	6.97178	https://www.genecards.org/cgi-bin/carddisp.pl?gene=MK280073-502
MK280073-503		RNA Gene		3	GC04P189967	6.97178	https://www.genecards.org/cgi-bin/carddisp.pl?gene=MK280073-503
MK280073-504		RNA Gene		3	GC05P002523	6.97178	https://www.genecards.org/cgi-bin/carddisp.pl?gene=MK280073-504

MK280073-505		RNA Gene		3	GC05P008734	6.97178	https://www.genecards.org/cgi-bin/carddisp.pl?gene=MK280073-505
MK280073-506		RNA Gene		3	GC05P010784	6.97178	https://www.genecards.org/cgi-bin/carddisp.pl?gene=MK280073-506
MK280073-507		RNA Gene		3	GC05M022028	6.97178	https://www.genecards.org/cgi-bin/carddisp.pl?gene=MK280073-507
MK280073-508		RNA Gene		3	GC05M025414	6.97178	https://www.genecards.org/cgi-bin/carddisp.pl?gene=MK280073-508
MK280073-509		RNA Gene		3	GC05M030427	6.97178	https://www.genecards.org/cgi-bin/carddisp.pl?gene=MK280073-509
MK280073-511		RNA Gene		3	GC05P035078	6.97178	https://www.genecards.org/cgi-bin/carddisp.pl?gene=MK280073-511
MK280073-512		RNA Gene		3	GC05P041746	6.97178	https://www.genecards.org/cgi-bin/carddisp.pl?gene=MK280073-512
MK280073-514		RNA Gene		3	GC05M052100	6.97178	https://www.genecards.org/cgi-bin/carddisp.pl?gene=MK280073-514
MK280073-515		RNA Gene		3	GC05M052974	6.97178	https://www.genecards.org/cgi-bin/carddisp.pl?gene=MK280073-515
MK280073-516		RNA Gene		3	GC05P059693	6.97178	https://www.genecards.org/cgi-bin/carddisp.pl?gene=MK280073-516
MK280073-517		RNA Gene		3	GC05P063489	6.97178	https://www.genecards.org/cgi-bin/carddisp.pl?gene=MK280073-517
MK280073-518		RNA Gene		3	GC05P065409	6.97178	https://www.genecards.org/cgi-bin/carddisp.pl?gene=MK280073-518

MK280073-526		RNA Gene		3	GC05M083797	6.97178	https://www.genecards.org/cgi-bin/carddisp.pl?gene=MK280073-526
MK280073-528		RNA Gene		3	GC05M113933	6.97178	https://www.genecards.org/cgi-bin/carddisp.pl?gene=MK280073-528
MK280073-529		RNA Gene		3	GC05M133184	6.97178	https://www.genecards.org/cgi-bin/carddisp.pl?gene=MK280073-529
MK280073-530		RNA Gene		3	GC05M144054	6.97178	https://www.genecards.org/cgi-bin/carddisp.pl?gene=MK280073-530
MK280073-531		RNA Gene		3	GC05M144827	6.97178	https://www.genecards.org/cgi-bin/carddisp.pl?gene=MK280073-531
MK280073-532		RNA Gene		3	GC05P155172	6.97178	https://www.genecards.org/cgi-bin/carddisp.pl?gene=MK280073-532
MK280073-534		RNA Gene		3	GC05P160596	6.97178	https://www.genecards.org/cgi-bin/carddisp.pl?gene=MK280073-534
MK280073-535		RNA Gene		3	GC05M171062	6.97178	https://www.genecards.org/cgi-bin/carddisp.pl?gene=MK280073-535
MK280073-536		RNA Gene		3	GC06M001469	6.97178	https://www.genecards.org/cgi-bin/carddisp.pl?gene=MK280073-536
MK280073-537		RNA Gene		3	GC06P023809	6.97178	https://www.genecards.org/cgi-bin/carddisp.pl?gene=MK280073-537
MK280073-538		RNA Gene		3	GC06P116608	6.97178	https://www.genecards.org/cgi-bin/carddisp.pl?gene=MK280073-538
MK280073-540		RNA Gene		3	GC06P116609	6.97178	https://www.genecards.org/cgi-bin/carddisp.pl?gene=MK280073-540

MK280073-541		RNA Gene		3	GC06M085584	6.97178	https://www.genecards.org/cgi-bin/carddisp.pl?gene=MK280073-541
MK280073-542		RNA Gene		3	GC06P116611	6.97178	https://www.genecards.org/cgi-bin/carddisp.pl?gene=MK280073-542
MK280073-543		RNA Gene		3	GC06P116612	6.97178	https://www.genecards.org/cgi-bin/carddisp.pl?gene=MK280073-543
MK280073-545		RNA Gene		3	GC06M085586	6.97178	https://www.genecards.org/cgi-bin/carddisp.pl?gene=MK280073-545
MK280073-546		RNA Gene		3	GC06M085587	6.97178	https://www.genecards.org/cgi-bin/carddisp.pl?gene=MK280073-546
MK280073-547		RNA Gene		3	GC06M085588	6.97178	https://www.genecards.org/cgi-bin/carddisp.pl?gene=MK280073-547
MK280073-548		RNA Gene		3	GC06M085589	6.97178	https://www.genecards.org/cgi-bin/carddisp.pl?gene=MK280073-548
MK280073-549		RNA Gene		3	GC06P116613	6.97178	https://www.genecards.org/cgi-bin/carddisp.pl?gene=MK280073-549
MK280073-550		RNA Gene		3	GC06P116614	6.97178	https://www.genecards.org/cgi-bin/carddisp.pl?gene=MK280073-550
MK280073-551		RNA Gene		3	GC06P116615	6.97178	https://www.genecards.org/cgi-bin/carddisp.pl?gene=MK280073-551
MK280073-552		RNA Gene		3	GC06P116617	6.97178	https://www.genecards.org/cgi-bin/carddisp.pl?gene=MK280073-552
MK280073-553		RNA Gene		3	GC06M086714	6.97178	https://www.genecards.org/cgi-bin/carddisp.pl?gene=MK280073-553

MK280073-554		RNA Gene		3	GC06P116619	6.97178	https://www.genecards.org/cgi-bin/carddisp.pl?gene=MK280073-554
MK280073-555		RNA Gene		3	GC06M099044	6.97178	https://www.genecards.org/cgi-bin/carddisp.pl?gene=MK280073-555
MK280073-556		RNA Gene		3	GC06M102807	6.97178	https://www.genecards.org/cgi-bin/carddisp.pl?gene=MK280073-556
MK280073-558		RNA Gene		3	GC06P118180	6.97178	https://www.genecards.org/cgi-bin/carddisp.pl?gene=MK280073-558
MK280073-559		RNA Gene		3	GC06M117099	6.97178	https://www.genecards.org/cgi-bin/carddisp.pl?gene=MK280073-559
MK280073-560		RNA Gene		3	GC06P117253	6.97178	https://www.genecards.org/cgi-bin/carddisp.pl?gene=MK280073-560
MK280073-562		RNA Gene		3	GC06P120821	6.97178	https://www.genecards.org/cgi-bin/carddisp.pl?gene=MK280073-562
MK280073-563		RNA Gene		3	GC06P124729	6.97178	https://www.genecards.org/cgi-bin/carddisp.pl?gene=MK280073-563
MK280073-564		RNA Gene		3	GC06M126239	6.97178	https://www.genecards.org/cgi-bin/carddisp.pl?gene=MK280073-564
MK280073-565		RNA Gene		3	GC06M153696	6.97178	https://www.genecards.org/cgi-bin/carddisp.pl?gene=MK280073-565
MK280073-566		RNA Gene		3	GC06P165371	6.97178	https://www.genecards.org/cgi-bin/carddisp.pl?gene=MK280073-566
MK280073-567		RNA Gene		3	GC06M167085	6.97178	https://www.genecards.org/cgi-bin/carddisp.pl?gene=MK280073-567

MK280073-568		RNA Gene		3	GC07P008614	6.97178	https://www.genecards.org/cgi-bin/carddisp.pl?gene=MK280073-568
MK280073-569		RNA Gene		3	GC07P025748	6.97178	https://www.genecards.org/cgi-bin/carddisp.pl?gene=MK280073-569
MK280073-570		RNA Gene		3	GC07M031511	6.97178	https://www.genecards.org/cgi-bin/carddisp.pl?gene=MK280073-570
MK280073-572		RNA Gene		3	GC07P032468	6.97178	https://www.genecards.org/cgi-bin/carddisp.pl?gene=MK280073-572
MK280073-573		RNA Gene		3	GC07M037808	6.97178	https://www.genecards.org/cgi-bin/carddisp.pl?gene=MK280073-573
MK280073-574		RNA Gene		3	GC07M043778	6.97178	https://www.genecards.org/cgi-bin/carddisp.pl?gene=MK280073-574
MK280073-575		RNA Gene		3	GC07P045319	6.97178	https://www.genecards.org/cgi-bin/carddisp.pl?gene=MK280073-575
MK280073-577		RNA Gene		3	GC07P049653	6.97178	https://www.genecards.org/cgi-bin/carddisp.pl?gene=MK280073-577
MK280073-578		RNA Gene		3	GC07P051672	6.97178	https://www.genecards.org/cgi-bin/carddisp.pl?gene=MK280073-578
MK280073-579		RNA Gene		3	GC07M053030	6.97178	https://www.genecards.org/cgi-bin/carddisp.pl?gene=MK280073-579
MK280073-580		RNA Gene		3	GC07M068309	6.97178	https://www.genecards.org/cgi-bin/carddisp.pl?gene=MK280073-580
MK280073-581		RNA Gene		3	GC07M078163	6.97178	https://www.genecards.org/cgi-bin/carddisp.pl?gene=MK280073-581

MK280073-582		RNA Gene		3	GC07P088875	6.97178	https://www.genecards.org/cgi-bin/carddisp.pl?gene=MK280073-582
MK280073-583		RNA Gene		3	GC07M089484	6.97178	https://www.genecards.org/cgi-bin/carddisp.pl?gene=MK280073-583
MK280073-584		RNA Gene		3	GC07M091060	6.97178	https://www.genecards.org/cgi-bin/carddisp.pl?gene=MK280073-584
MK280073-585		RNA Gene		3	GC07P091217	6.97178	https://www.genecards.org/cgi-bin/carddisp.pl?gene=MK280073-585
MK280073-588		RNA Gene		3	GC07P106228	6.97178	https://www.genecards.org/cgi-bin/carddisp.pl?gene=MK280073-588
MK280073-589		RNA Gene		3	GC07M109419	6.97178	https://www.genecards.org/cgi-bin/carddisp.pl?gene=MK280073-589
MK280073-590		RNA Gene		3	GC07P109911	6.97178	https://www.genecards.org/cgi-bin/carddisp.pl?gene=MK280073-590
MK280073-591		RNA Gene		3	GC07M110673	6.97178	https://www.genecards.org/cgi-bin/carddisp.pl?gene=MK280073-591
MK280073-592		RNA Gene		3	GC07M112721	6.97178	https://www.genecards.org/cgi-bin/carddisp.pl?gene=MK280073-592
MK280073-593		RNA Gene		3	GC07P119465	6.97178	https://www.genecards.org/cgi-bin/carddisp.pl?gene=MK280073-593
MK280073-594		RNA Gene		3	GC07M120238	6.97178	https://www.genecards.org/cgi-bin/carddisp.pl?gene=MK280073-594
MK280073-595		RNA Gene		3	GC07P121251	6.97178	https://www.genecards.org/cgi-bin/carddisp.pl?gene=MK280073-595

MK280073-596		RNA Gene		3	GC07M121711	6.97178	https://www.genecards.org/cgi-bin/carddisp.pl?gene=MK280073-596
MK280073-597		RNA Gene		3	GC07M145239	6.97178	https://www.genecards.org/cgi-bin/carddisp.pl?gene=MK280073-597
MK280073-598		RNA Gene		3	GC07P153529	6.97178	https://www.genecards.org/cgi-bin/carddisp.pl?gene=MK280073-598
MK280073-599		RNA Gene		3	GC08P016692	6.97178	https://www.genecards.org/cgi-bin/carddisp.pl?gene=MK280073-599
MK280073-600		RNA Gene		3	GC08M023398	6.97178	https://www.genecards.org/cgi-bin/carddisp.pl?gene=MK280073-600
MK280073-129		RNA Gene		2	GC13M038943	6.97178	https://www.genecards.org/cgi-bin/carddisp.pl?gene=MK280073-129
MK280073-130		RNA Gene		2	GC13M038829	6.97178	https://www.genecards.org/cgi-bin/carddisp.pl?gene=MK280073-130
MK280073-166		RNA Gene		2	GC14P040363	6.97178	https://www.genecards.org/cgi-bin/carddisp.pl?gene=MK280073-166
MK280073-167		RNA Gene		2	GC14M036896	6.97178	https://www.genecards.org/cgi-bin/carddisp.pl?gene=MK280073-167
MK280073-223		RNA Gene		2	GC16M042166	6.97178	https://www.genecards.org/cgi-bin/carddisp.pl?gene=MK280073-223
MK280073-254		RNA Gene		2	GC17M014517	6.97178	https://www.genecards.org/cgi-bin/carddisp.pl?gene=MK280073-254
MK280073-256		RNA Gene		2	GC17P091332	6.97178	https://www.genecards.org/cgi-bin/carddisp.pl?gene=MK280073-256

MK280073-263		RNA Gene		2	GC17M066335	6.97178	https://www.genecards.org/cgi-bin/carddisp.pl?gene=MK280073-263
MK280073-327		RNA Gene		2	GC19M084130	6.97178	https://www.genecards.org/cgi-bin/carddisp.pl?gene=MK280073-327
MK280073-392		RNA Gene		2	GC20P029334	6.97178	https://www.genecards.org/cgi-bin/carddisp.pl?gene=MK280073-392
MK280073-393		RNA Gene		2	GC20M029470	6.97178	https://www.genecards.org/cgi-bin/carddisp.pl?gene=MK280073-393
MK280073-394		RNA Gene		2	GC20P029771	6.97178	https://www.genecards.org/cgi-bin/carddisp.pl?gene=MK280073-394
MK280073-400		RNA Gene		2	GC21P005353	6.97178	https://www.genecards.org/cgi-bin/carddisp.pl?gene=MK280073-400
MK280073-401		RNA Gene		2	GC21P007284	6.97178	https://www.genecards.org/cgi-bin/carddisp.pl?gene=MK280073-401
MK280073-408		RNA Gene		2	GC21P015376	6.97178	https://www.genecards.org/cgi-bin/carddisp.pl?gene=MK280073-408
MK280073-421		RNA Gene		2	GC22P010585	6.97178	https://www.genecards.org/cgi-bin/carddisp.pl?gene=MK280073-421
MK280073-422		RNA Gene		2	GC22M011365	6.97178	https://www.genecards.org/cgi-bin/carddisp.pl?gene=MK280073-422
MK280073-423		RNA Gene		2	GC22M011431	6.97178	https://www.genecards.org/cgi-bin/carddisp.pl?gene=MK280073-423
MK280073-424		RNA Gene		2	GC22M011593	6.97178	https://www.genecards.org/cgi-bin/carddisp.pl?gene=MK280073-424

MK280073-425		RNA Gene		2	GC22P012392	6.97178	https://www.genecards.org/cgi-bin/carddisp.pl?gene=MK280073-425
MK280073-426		RNA Gene		2	GC22M015219	6.97178	https://www.genecards.org/cgi-bin/carddisp.pl?gene=MK280073-426
MK280073-429		RNA Gene		2	GC22P059636	6.97178	https://www.genecards.org/cgi-bin/carddisp.pl?gene=MK280073-429
MK280073-489		RNA Gene		2	GC04P054045	6.97178	https://www.genecards.org/cgi-bin/carddisp.pl?gene=MK280073-489
MK280073-520		RNA Gene		2	GC05M068624	6.97178	https://www.genecards.org/cgi-bin/carddisp.pl?gene=MK280073-520
MK280073-539		RNA Gene		2	GC06M085583	6.97178	https://www.genecards.org/cgi-bin/carddisp.pl?gene=MK280073-539
MK280073-576		RNA Gene		2	GC07P048877	6.97178	https://www.genecards.org/cgi-bin/carddisp.pl?gene=MK280073-576
MK280073-587		RNA Gene		2	GC07M097262	6.97178	https://www.genecards.org/cgi-bin/carddisp.pl?gene=MK280073-587
FOXO1	Forkhead Box O1	Protein Coding	Q12778	55	GC13M040555	6.953826	https://www.genecards.org/cgi-bin/carddisp.pl?gene=FOXO1
BCHE	Butyrylcholinesterase	Protein Coding	P06276	54	GC03M165772	6.953368	https://www.genecards.org/cgi-bin/carddisp.pl?gene=BCHE
CAV1	Caveolin 1	Protein Coding	Q03135	52	GC07P116524	6.940887	https://www.genecards.org/cgi-bin/carddisp.pl?gene=CAV1
NTS	Neurotensin	Protein Coding	P30990	45	GC12P085876	6.923548	https://www.genecards.org/cgi-bin/carddisp.pl?gene=NTS

OTC	Ornithine Transcarbamylase	Protein Coding	P00480	50	GC0XP03833 0	6.87773 9	https://www.genecards.org/cgi-bin/carddisp.pl?gene=OTC
CCL11	C-C Motif Chemokine Ligand 11	Protein Coding	P51671	49	GC17P034285	6.86995 3	https://www.genecards.org/cgi-bin/carddisp.pl?gene=CCL11
MYC	MYC Proto-Oncogene, BHLH Transcription Factor	Protein Coding	P01106	56	GC08P127735	6.86258 7	https://www.genecards.org/cgi-bin/carddisp.pl?gene=MYC
TBX1	T-Box Transcription Factor 1	Protein Coding	O43435	46	GC22P056009	6.84819 5	https://www.genecards.org/cgi-bin/carddisp.pl?gene=TBX1
NEGR1	Neuronal Growth Regulator 1	Protein Coding	Q7Z3B1	44	GC01M07139 5	6.83210 4	https://www.genecards.org/cgi-bin/carddisp.pl?gene=NEGR1
HMGCR	3-Hydroxy-3-Methylglutaryl-CoA Reductase	Protein Coding	P04035	51	GC05P075336	6.83069 8	https://www.genecards.org/cgi-bin/carddisp.pl?gene=HMGCR
MIR192	MicroRNA 192	RNA Gene		22	GC11M06489 1	6.82556	https://www.genecards.org/cgi-bin/carddisp.pl?gene=MIR192
MIR517A	MicroRNA 517a	RNA Gene		16	GC19P053712	6.81164 1	https://www.genecards.org/cgi-bin/carddisp.pl?gene=MIR517A
PLA2G4A	Phospholipase A2 Group IVA	Protein Coding	P47712	52	GC01P186798	6.80341 8	https://www.genecards.org/cgi-bin/carddisp.pl?gene=PLA2G4A
TH	Tyrosine Hydroxylase	Protein Coding	P07101	54	GC11M00216 3	6.80195 7	https://www.genecards.org/cgi-bin/carddisp.pl?gene=TH
HOTAIR	HOX Transcript Antisense RNA	RNA Gene		25	GC12M05396 2	6.78833 5	https://www.genecards.org/cgi-bin/carddisp.pl?gene=HOTAIR
CST3	Cystatin C	Protein Coding	P01034	48	GC20M02381 4	6.78209	https://www.genecards.org/cgi-bin/carddisp.pl?gene=CST3

TIMP2	TIMP Metallopeptidase Inhibitor 2	Protein Coding	P16035	45	GC17M078852	6.773983	https://www.genecards.org/cgi-bin/carddisp.pl?gene=TIMP2
IGF2BP2	Insulin Like Growth Factor 2 MRNA Binding Protein 2	Protein Coding	Q9Y6M1	48	GC03M185643	6.772669	https://www.genecards.org/cgi-bin/carddisp.pl?gene=IGF2BP2
MIR142	MicroRNA 142	RNA Gene		22	GC17M058331	6.76712	https://www.genecards.org/cgi-bin/carddisp.pl?gene=MIR142
SPP1	Secreted Phosphoprotein 1	Protein Coding	P10451	50	GC04P087975	6.761188	https://www.genecards.org/cgi-bin/carddisp.pl?gene=SPP1
PGR	Progesterone Receptor	Protein Coding	P06401	53	GC11M114256	6.754402	https://www.genecards.org/cgi-bin/carddisp.pl?gene=PGR
SIRT3	Sirtuin 3	Protein Coding	Q9NTG7	51	GC11M000215	6.751866	https://www.genecards.org/cgi-bin/carddisp.pl?gene=SIRT3
PTCH1	Patched 1	Protein Coding	Q13635	55	GC09M095442	6.751506	https://www.genecards.org/cgi-bin/carddisp.pl?gene=PTCH1
MIR214	MicroRNA 214	RNA Gene		23	GC01M172234	6.744717	https://www.genecards.org/cgi-bin/carddisp.pl?gene=MIR214
MT-TL2	Mitochondrially Encoded TRNA-Leu (CUN) 2	RNA Gene		15	GCMTP012268	6.730167	https://www.genecards.org/cgi-bin/carddisp.pl?gene=MT-TL2
NDUFS4	NADH:Ubiquinone Oxidoreductase Subunit S4	Protein Coding	O43181	47	GC05P053560	6.72819	https://www.genecards.org/cgi-bin/carddisp.pl?gene=NDUFS4
TNFRSF1A	TNF Receptor Superfamily Member 1A	Protein Coding	P19438	54	GC12M006328	6.724816	https://www.genecards.org/cgi-bin/carddisp.pl?gene=TNFRSF1A
TULP1	TUB Like Protein 1	Protein Coding	O00294	46	GC06M084085	6.718503	https://www.genecards.org/cgi-bin/carddisp.pl?gene=TULP1

CIDEC	Cell Death Inducing DFFA Like Effector C	Protein Coding	Q96AQ7	45	GC03M009866	6.716619	https://www.genecards.org/cgi-bin/carddisp.pl?gene=CIDEC
ERBB2	Erb-B2 Receptor Tyrosine Kinase 2	Protein Coding	P04626	59	GC17P039687	6.713375	https://www.genecards.org/cgi-bin/carddisp.pl?gene=ERBB2
TF	Transferrin	Protein Coding	P02787	54	GC03P136876	6.693521	https://www.genecards.org/cgi-bin/carddisp.pl?gene=TF
KNG1	Kininogen 1	Protein Coding	P01042	52	GC03P186717	6.691689	https://www.genecards.org/cgi-bin/carddisp.pl?gene=KNG1
DNAJC30	DnaJ Heat Shock Protein Family (Hsp40) Member C30	Protein Coding	Q96LL9	40	GC07M073680	6.690156	https://www.genecards.org/cgi-bin/carddisp.pl?gene=DNAJC30
MT-TI	Mitochondrially Encoded TRNA-Ile (AUU/C)	RNA Gene		15	GCMTP004265	6.672063	https://www.genecards.org/cgi-bin/carddisp.pl?gene=MT-TI
JAK2	Janus Kinase 2	Protein Coding	O60674	57	GC09P004985	6.661477	https://www.genecards.org/cgi-bin/carddisp.pl?gene=JAK2
CD4	CD4 Molecule	Protein Coding	P01730	53	GC12P006786	6.657209	https://www.genecards.org/cgi-bin/carddisp.pl?gene=CD4
AMH	Anti-Mullerian Hormone	Protein Coding	P03971	49	GC19P008599	6.651268	https://www.genecards.org/cgi-bin/carddisp.pl?gene=AMH
MIRLET7A1	MicroRNA Let-7a-1	RNA Gene		23	GC09P094175	6.63818	https://www.genecards.org/cgi-bin/carddisp.pl?gene=MIRLET7A1
TTTY10	Testis Expressed Transcript, Y-Linked 10	RNA Gene	Q9BZA0	17	GC0YM020375	6.621843	https://www.genecards.org/cgi-bin/carddisp.pl?gene=TTTY10
MIR210	MicroRNA 210	RNA Gene		22	GC11M008890	6.621014	https://www.genecards.org/cgi-bin/carddisp.pl?gene=MIR210

MK280073-623		RNA Gene		5	GC08P108062	6.61095 4	https://www.genecards.org/cgi-bin/carddisp.pl?gene=MK280073-623
MK280073-609		RNA Gene		4	GC08M06765 4	6.61095 4	https://www.genecards.org/cgi-bin/carddisp.pl?gene=MK280073-609
MK280073-621		RNA Gene		4	GC08M10496 2	6.61095 4	https://www.genecards.org/cgi-bin/carddisp.pl?gene=MK280073-621
MK280073-606		RNA Gene		3	GC08P055464	6.61095 4	https://www.genecards.org/cgi-bin/carddisp.pl?gene=MK280073-606
MK280073-615		RNA Gene		3	GC08M08885 8	6.61095 4	https://www.genecards.org/cgi-bin/carddisp.pl?gene=MK280073-615
MK280073-618		RNA Gene		3	GC08M09704 3	6.61095 4	https://www.genecards.org/cgi-bin/carddisp.pl?gene=MK280073-618
MK280073-624		RNA Gene		3	GC08P115374	6.61095 4	https://www.genecards.org/cgi-bin/carddisp.pl?gene=MK280073-624
MK280073-625		RNA Gene		3	GC08M12019 2	6.61095 4	https://www.genecards.org/cgi-bin/carddisp.pl?gene=MK280073-625
MK280073-629		RNA Gene		3	GC08M13619 4	6.61095 4	https://www.genecards.org/cgi-bin/carddisp.pl?gene=MK280073-629
MK280073-654		RNA Gene		3	GC09P090267	6.61095 4	https://www.genecards.org/cgi-bin/carddisp.pl?gene=MK280073-654
MK280073-655		RNA Gene		3	GC09M10492 2	6.61095 4	https://www.genecards.org/cgi-bin/carddisp.pl?gene=MK280073-655
MK280073-656		RNA Gene		3	GC09M10492 3	6.61095 4	https://www.genecards.org/cgi-bin/carddisp.pl?gene=MK280073-656

MK280073-659		RNA Gene		3	GC09M106185	6.610954	https://www.genecards.org/cgi-bin/carddisp.pl?gene=MK280073-659
MK280073-670		RNA Gene		3	GC0XM027615	6.610954	https://www.genecards.org/cgi-bin/carddisp.pl?gene=MK280073-670
MK280073-674		RNA Gene		3	GC0XP042058	6.610954	https://www.genecards.org/cgi-bin/carddisp.pl?gene=MK280073-674
MK280073-676		RNA Gene		3	GC0XM063601	6.610954	https://www.genecards.org/cgi-bin/carddisp.pl?gene=MK280073-676
MK280073-678		RNA Gene		3	GC0XM067473	6.610954	https://www.genecards.org/cgi-bin/carddisp.pl?gene=MK280073-678
MK280073-683		RNA Gene		3	GC0XP099339	6.610954	https://www.genecards.org/cgi-bin/carddisp.pl?gene=MK280073-683
MK280073-686		RNA Gene		3	GC0XP112722	6.610954	https://www.genecards.org/cgi-bin/carddisp.pl?gene=MK280073-686
MK280073-688		RNA Gene		3	GC0XP137675	6.610954	https://www.genecards.org/cgi-bin/carddisp.pl?gene=MK280073-688
MK280073-689		RNA Gene		3	GC0XM140239	6.610954	https://www.genecards.org/cgi-bin/carddisp.pl?gene=MK280073-689
MK280073-691		RNA Gene		3	GC0XP154942	6.610954	https://www.genecards.org/cgi-bin/carddisp.pl?gene=MK280073-691
MK280073-601		RNA Gene		2	GC08P024563	6.610954	https://www.genecards.org/cgi-bin/carddisp.pl?gene=MK280073-601
MK280073-602		RNA Gene		2	GC08M031235	6.610954	https://www.genecards.org/cgi-bin/carddisp.pl?gene=MK280073-602

MK280073-603		RNA Gene		2	GC08P039811	6.610954	https://www.genecards.org/cgi-bin/carddisp.pl?gene=MK280073-603
MK280073-604		RNA Gene		2	GC08P051496	6.610954	https://www.genecards.org/cgi-bin/carddisp.pl?gene=MK280073-604
MK280073-605		RNA Gene		2	GC08P051785	6.610954	https://www.genecards.org/cgi-bin/carddisp.pl?gene=MK280073-605
MK280073-607		RNA Gene		2	GC08M056533	6.610954	https://www.genecards.org/cgi-bin/carddisp.pl?gene=MK280073-607
MK280073-608		RNA Gene		2	GC08P064087	6.610954	https://www.genecards.org/cgi-bin/carddisp.pl?gene=MK280073-608
MK280073-610		RNA Gene		2	GC08P070812	6.610954	https://www.genecards.org/cgi-bin/carddisp.pl?gene=MK280073-610
MK280073-611		RNA Gene		2	GC08M076901	6.610954	https://www.genecards.org/cgi-bin/carddisp.pl?gene=MK280073-611
MK280073-612		RNA Gene		2	GC08M077605	6.610954	https://www.genecards.org/cgi-bin/carddisp.pl?gene=MK280073-612
MK280073-613		RNA Gene		2	GC08M081329	6.610954	https://www.genecards.org/cgi-bin/carddisp.pl?gene=MK280073-613
MK280073-614		RNA Gene		2	GC08M084619	6.610954	https://www.genecards.org/cgi-bin/carddisp.pl?gene=MK280073-614
MK280073-616		RNA Gene		2	GC08P089514	6.610954	https://www.genecards.org/cgi-bin/carddisp.pl?gene=MK280073-616
MK280073-617		RNA Gene		2	GC08P090048	6.610954	https://www.genecards.org/cgi-bin/carddisp.pl?gene=MK280073-617

MK280073-619		RNA Gene		2	GC08P099743	6.61095<br>4	https://www.genecards.org/cgi-bin/carddisp.pl?gene=MK280073-619
MK280073-620		RNA Gene		2	GC08M10360<br>7	6.61095<br>4	https://www.genecards.org/cgi-bin/carddisp.pl?gene=MK280073-620
MK280073-622		RNA Gene		2	GC08P107244	6.61095<br>4	https://www.genecards.org/cgi-bin/carddisp.pl?gene=MK280073-622
MK280073-626		RNA Gene		2	GC08M12064<br>8	6.61095<br>4	https://www.genecards.org/cgi-bin/carddisp.pl?gene=MK280073-626
MK280073-627		RNA Gene		2	GC08P128347	6.61095<br>4	https://www.genecards.org/cgi-bin/carddisp.pl?gene=MK280073-627
MK280073-628		RNA Gene		2	GC08P131067	6.61095<br>4	https://www.genecards.org/cgi-bin/carddisp.pl?gene=MK280073-628
MK280073-630		RNA Gene		2	GC09P001562	6.61095<br>4	https://www.genecards.org/cgi-bin/carddisp.pl?gene=MK280073-630
MK280073-631		RNA Gene		2	GC09M00157<br>4	6.61095<br>4	https://www.genecards.org/cgi-bin/carddisp.pl?gene=MK280073-631
MK280073-632		RNA Gene		2	GC09M00800<br>7	6.61095<br>4	https://www.genecards.org/cgi-bin/carddisp.pl?gene=MK280073-632
MK280073-633		RNA Gene		2	GC09M00937<br>2	6.61095<br>4	https://www.genecards.org/cgi-bin/carddisp.pl?gene=MK280073-633
MK280073-634		RNA Gene		2	GC09M01090<br>2	6.61095<br>4	https://www.genecards.org/cgi-bin/carddisp.pl?gene=MK280073-634
MK280073-635		RNA Gene		2	GC09M01164<br>0	6.61095<br>4	https://www.genecards.org/cgi-bin/carddisp.pl?gene=MK280073-635

MK280073-636		RNA Gene		2	GC09M013345	6.610954	https://www.genecards.org/cgi-bin/carddisp.pl?gene=MK280073-636
MK280073-637		RNA Gene		2	GC09P015947	6.610954	https://www.genecards.org/cgi-bin/carddisp.pl?gene=MK280073-637
MK280073-638		RNA Gene		2	GC09M017681	6.610954	https://www.genecards.org/cgi-bin/carddisp.pl?gene=MK280073-638
MK280073-639		RNA Gene		2	GC09P018719	6.610954	https://www.genecards.org/cgi-bin/carddisp.pl?gene=MK280073-639
MK280073-640		RNA Gene		2	GC09M024584	6.610954	https://www.genecards.org/cgi-bin/carddisp.pl?gene=MK280073-640
MK280073-641		RNA Gene		2	GC09P027243	6.610954	https://www.genecards.org/cgi-bin/carddisp.pl?gene=MK280073-641
MK280073-642		RNA Gene		2	GC09P029274	6.610954	https://www.genecards.org/cgi-bin/carddisp.pl?gene=MK280073-642
MK280073-643		RNA Gene		2	GC09P030110	6.610954	https://www.genecards.org/cgi-bin/carddisp.pl?gene=MK280073-643
MK280073-644		RNA Gene		2	GC09M032248	6.610954	https://www.genecards.org/cgi-bin/carddisp.pl?gene=MK280073-644
MK280073-645		RNA Gene		2	GC09P063953	6.610954	https://www.genecards.org/cgi-bin/carddisp.pl?gene=MK280073-645
MK280073-646		RNA Gene		2	GC09P070534	6.610954	https://www.genecards.org/cgi-bin/carddisp.pl?gene=MK280073-646
MK280073-647		RNA Gene		2	GC09M065713	6.610954	https://www.genecards.org/cgi-bin/carddisp.pl?gene=MK280073-647

MK280073-648		RNA Gene		2	GC09M070019	6.610954	https://www.genecards.org/cgi-bin/carddisp.pl?gene=MK280073-648
MK280073-649		RNA Gene		2	GC09M070023	6.610954	https://www.genecards.org/cgi-bin/carddisp.pl?gene=MK280073-649
MK280073-650		RNA Gene		2	GC09P084638	6.610954	https://www.genecards.org/cgi-bin/carddisp.pl?gene=MK280073-650
MK280073-651		RNA Gene		2	GC09P085761	6.610954	https://www.genecards.org/cgi-bin/carddisp.pl?gene=MK280073-651
MK280073-652		RNA Gene		2	GC09P087045	6.610954	https://www.genecards.org/cgi-bin/carddisp.pl?gene=MK280073-652
MK280073-653		RNA Gene		2	GC09P087905	6.610954	https://www.genecards.org/cgi-bin/carddisp.pl?gene=MK280073-653
MK280073-657		RNA Gene		2	GC09P096777	6.610954	https://www.genecards.org/cgi-bin/carddisp.pl?gene=MK280073-657
MK280073-658		RNA Gene		2	GC09P097208	6.610954	https://www.genecards.org/cgi-bin/carddisp.pl?gene=MK280073-658
MK280073-660		RNA Gene		2	GC09M106259	6.610954	https://www.genecards.org/cgi-bin/carddisp.pl?gene=MK280073-660
MK280073-661		RNA Gene		2	GC09P110397	6.610954	https://www.genecards.org/cgi-bin/carddisp.pl?gene=MK280073-661
MK280073-662		RNA Gene		2	GC0XP012110	6.610954	https://www.genecards.org/cgi-bin/carddisp.pl?gene=MK280073-662
MK280073-663		RNA Gene		2	GC0XP014009	6.610954	https://www.genecards.org/cgi-bin/carddisp.pl?gene=MK280073-663

MK280073-664		RNA Gene		2	GC0XP016364	6.610954	https://www.genecards.org/cgi-bin/carddisp.pl?gene=MK280073-664
MK280073-665		RNA Gene		2	GC0XP016501	6.610954	https://www.genecards.org/cgi-bin/carddisp.pl?gene=MK280073-665
MK280073-666		RNA Gene		2	GC0XM017785	6.610954	https://www.genecards.org/cgi-bin/carddisp.pl?gene=MK280073-666
MK280073-667		RNA Gene		2	GC0XP020849	6.610954	https://www.genecards.org/cgi-bin/carddisp.pl?gene=MK280073-667
MK280073-668		RNA Gene		2	GC0XP026057	6.610954	https://www.genecards.org/cgi-bin/carddisp.pl?gene=MK280073-668
MK280073-669		RNA Gene		2	GC0XM027525	6.610954	https://www.genecards.org/cgi-bin/carddisp.pl?gene=MK280073-669
MK280073-671		RNA Gene		2	GC0XM029985	6.610954	https://www.genecards.org/cgi-bin/carddisp.pl?gene=MK280073-671
MK280073-672		RNA Gene		2	GC0XM030984	6.610954	https://www.genecards.org/cgi-bin/carddisp.pl?gene=MK280073-672
MK280073-673		RNA Gene		2	GC0XM031081	6.610954	https://www.genecards.org/cgi-bin/carddisp.pl?gene=MK280073-673
MK280073-675		RNA Gene		2	GC0XM051963	6.610954	https://www.genecards.org/cgi-bin/carddisp.pl?gene=MK280073-675
MK280073-677		RNA Gene		2	GC0XP063650	6.610954	https://www.genecards.org/cgi-bin/carddisp.pl?gene=MK280073-677
MK280073-679		RNA Gene		2	GC0XP068012	6.610954	https://www.genecards.org/cgi-bin/carddisp.pl?gene=MK280073-679

MK280073-680		RNA Gene		2	GC0XP076271	6.610954	https://www.genecards.org/cgi-bin/carddisp.pl?gene=MK280073-680
MK280073-681		RNA Gene		2	GC0XM080155	6.610954	https://www.genecards.org/cgi-bin/carddisp.pl?gene=MK280073-681
MK280073-682		RNA Gene		2	GC0XM091335	6.610954	https://www.genecards.org/cgi-bin/carddisp.pl?gene=MK280073-682
MK280073-684		RNA Gene		2	GC0XP100058	6.610954	https://www.genecards.org/cgi-bin/carddisp.pl?gene=MK280073-684
MK280073-685		RNA Gene		2	GC0XM105041	6.610954	https://www.genecards.org/cgi-bin/carddisp.pl?gene=MK280073-685
MK280073-687		RNA Gene		2	GC0XP133624	6.610954	https://www.genecards.org/cgi-bin/carddisp.pl?gene=MK280073-687
MK280073-690		RNA Gene		2	GC0XM142910	6.610954	https://www.genecards.org/cgi-bin/carddisp.pl?gene=MK280073-690
MK280073-692		RNA Gene		2	GC0XM155303	6.610954	https://www.genecards.org/cgi-bin/carddisp.pl?gene=MK280073-692
MK280073-693		RNA Gene		2	GC0YP004426	6.610954	https://www.genecards.org/cgi-bin/carddisp.pl?gene=MK280073-693
MK280073-694		RNA Gene		2	GC0YP004755	6.610954	https://www.genecards.org/cgi-bin/carddisp.pl?gene=MK280073-694
MK280073-695		RNA Gene		2	GC0YP005300	6.610954	https://www.genecards.org/cgi-bin/carddisp.pl?gene=MK280073-695
MK280073-696		RNA Gene		2	GC0YP005318	6.610954	https://www.genecards.org/cgi-bin/carddisp.pl?gene=MK280073-696

MK280073-697		RNA Gene		2	GC0YM005046	6.610954	https://www.genecards.org/cgi-bin/carddisp.pl?gene=MK280073-697
MK280073-698		RNA Gene		2	GC0YM006152	6.610954	https://www.genecards.org/cgi-bin/carddisp.pl?gene=MK280073-698
MK280073-699		RNA Gene		2	GC0YM008139	6.610954	https://www.genecards.org/cgi-bin/carddisp.pl?gene=MK280073-699
MK280073-700		RNA Gene		2	GC0YP010085	6.610954	https://www.genecards.org/cgi-bin/carddisp.pl?gene=MK280073-700
MK280073-701		RNA Gene		2	GC0YM010333	6.610954	https://www.genecards.org/cgi-bin/carddisp.pl?gene=MK280073-701
MK280073-702		RNA Gene		2	GC0YM010334	6.610954	https://www.genecards.org/cgi-bin/carddisp.pl?gene=MK280073-702
MK280073-703		RNA Gene		2	GC0YM011993	6.610954	https://www.genecards.org/cgi-bin/carddisp.pl?gene=MK280073-703
MK280073-704		RNA Gene		2	GC0YP012139	6.610954	https://www.genecards.org/cgi-bin/carddisp.pl?gene=MK280073-704
MK280073-705		RNA Gene		2	GC0YM013150	6.610954	https://www.genecards.org/cgi-bin/carddisp.pl?gene=MK280073-705
MK280073-706		RNA Gene		2	GC0YM013518	6.610954	https://www.genecards.org/cgi-bin/carddisp.pl?gene=MK280073-706
MK280073-707		RNA Gene		2	GC0YP014167	6.610954	https://www.genecards.org/cgi-bin/carddisp.pl?gene=MK280073-707
MK280073-708		RNA Gene		2	GC0YM013870	6.610954	https://www.genecards.org/cgi-bin/carddisp.pl?gene=MK280073-708

MK280073-709		RNA Gene		2	GC0YP014150	6.610954	https://www.genecards.org/cgi-bin/carddisp.pl?gene=MK280073-709
MK280073-710		RNA Gene		2	GC0YM014028	6.610954	https://www.genecards.org/cgi-bin/carddisp.pl?gene=MK280073-710
MK280073-711		RNA Gene		2	GC0YP014285	6.610954	https://www.genecards.org/cgi-bin/carddisp.pl?gene=MK280073-711
MK280073-712		RNA Gene		2	GC0YP014925	6.610954	https://www.genecards.org/cgi-bin/carddisp.pl?gene=MK280073-712
MK280073-713		RNA Gene		2	GC0YP014956	6.610954	https://www.genecards.org/cgi-bin/carddisp.pl?gene=MK280073-713
MK280073-714		RNA Gene		2	GC0YP015026	6.610954	https://www.genecards.org/cgi-bin/carddisp.pl?gene=MK280073-714
MK280073-715		RNA Gene		2	GC0YM015158	6.610954	https://www.genecards.org/cgi-bin/carddisp.pl?gene=MK280073-715
MK280073-716		RNA Gene		2	GC0YM015589	6.610954	https://www.genecards.org/cgi-bin/carddisp.pl?gene=MK280073-716
MK280073-717		RNA Gene		2	GC0YP015889	6.610954	https://www.genecards.org/cgi-bin/carddisp.pl?gene=MK280073-717
MK280073-718		RNA Gene		2	GC0YP016029	6.610954	https://www.genecards.org/cgi-bin/carddisp.pl?gene=MK280073-718
MK280073-719		RNA Gene		2	GC0YP017217	6.610954	https://www.genecards.org/cgi-bin/carddisp.pl?gene=MK280073-719
MK280073-720		RNA Gene		2	GC0YM019584	6.610954	https://www.genecards.org/cgi-bin/carddisp.pl?gene=MK280073-720

MK280073-721		RNA Gene		2	GC0YM019752	6.610954	https://www.genecards.org/cgi-bin/carddisp.pl?gene=MK280073-721
MK280073-722		RNA Gene		2	GC0YP019911	6.610954	https://www.genecards.org/cgi-bin/carddisp.pl?gene=MK280073-722
MTFMT	Mitochondrial Methionyl-TRNA Formyltransferase	Protein Coding	Q96DP5	44	GC15M065001	6.610591	https://www.genecards.org/cgi-bin/carddisp.pl?gene=MTFMT
SMAD4	SMAD Family Member 4	Protein Coding	Q13485	57	GC18P051028	6.609214	https://www.genecards.org/cgi-bin/carddisp.pl?gene=SMAD4
RAI1	Retinoic Acid Induced 1	Protein Coding	Q7Z5J4	44	GC17P085602	6.599614	https://www.genecards.org/cgi-bin/carddisp.pl?gene=RAI1
BCL2	BCL2 Apoptosis Regulator	Protein Coding	P10415	55	GC18M063123	6.565952	https://www.genecards.org/cgi-bin/carddisp.pl?gene=BCL2
EDNRA	Endothelin Receptor Type A	Protein Coding	P25101	53	GC04P147480	6.551305	https://www.genecards.org/cgi-bin/carddisp.pl?gene=EDNRA
MYD88	MYD88 Innate Immune Signal Transduction Adaptor	Protein Coding	Q99836	53	GC03P038211	6.548284	https://www.genecards.org/cgi-bin/carddisp.pl?gene=MYD88
MIR30E	MicroRNA 30e	RNA Gene		23	GC01P040754	6.533655	https://www.genecards.org/cgi-bin/carddisp.pl?gene=MIR30E
ALDH2	Aldehyde Dehydrogenase 2 Family Member	Protein Coding	P05091	54	GC12P111766	6.52742	https://www.genecards.org/cgi-bin/carddisp.pl?gene=ALDH2
SMARCA4	SWI/SNF Related, Matrix Associated, Actin Dependent Regulator Of Chromatin, Subfamily A, Member 4	Protein Coding	P51532	55	GC19P088794	6.517673	https://www.genecards.org/cgi-bin/carddisp.pl?gene=SMARCA4
PVT1	Pvt1 Oncogene	RNA Gene		27	GC08P127945	6.491403	https://www.genecards.org/cgi-bin/carddisp.pl?gene=PVT1

OTX2	Orthodenticle Homeobox 2	Protein Coding	P32243	50	GC14M056799	6.470625	https://www.genecards.org/cgi-bin/carddisp.pl?gene=OTX2
RPS6KB1	Ribosomal Protein S6 Kinase B1	Protein Coding	P23443	53	GC17P059893	6.462429	https://www.genecards.org/cgi-bin/carddisp.pl?gene=RPS6KB1
CPT2	Carnitine Palmitoyltransferase 2	Protein Coding	P23786	53	GC01P053196	6.462142	https://www.genecards.org/cgi-bin/carddisp.pl?gene=CPT2
MIR193A	MicroRNA 193a	RNA Gene		21	GC17P031559	6.457162	https://www.genecards.org/cgi-bin/carddisp.pl?gene=MIR193A
MIR23A	MicroRNA 23a	RNA Gene		22	GC19M015893	6.453777	https://www.genecards.org/cgi-bin/carddisp.pl?gene=MIR23A
CAT	Catalase	Protein Coding	P04040	55	GC11P034460	6.453213	https://www.genecards.org/cgi-bin/carddisp.pl?gene=CAT
MTCH2	Mitochondrial Carrier 2	Protein Coding	Q9Y6C9	44	GC11M047604	6.45049	https://www.genecards.org/cgi-bin/carddisp.pl?gene=MTCH2
SDHA	Succinate Dehydrogenase Complex Flavoprotein Subunit A	Protein Coding	P31040	52	GC05P000220	6.446855	https://www.genecards.org/cgi-bin/carddisp.pl?gene=SDHA
ANGPT2	Angiopoietin 2	Protein Coding	O15123	53	GC08M006499	6.445929	https://www.genecards.org/cgi-bin/carddisp.pl?gene=ANGPT2
MIR130B	MicroRNA 130b	RNA Gene		20	GC22P059633	6.439576	https://www.genecards.org/cgi-bin/carddisp.pl?gene=MIR130B
MIR10B	MicroRNA 10b	RNA Gene		23	GC02P176150	6.427578	https://www.genecards.org/cgi-bin/carddisp.pl?gene=MIR10B
MIR136	MicroRNA 136	RNA Gene		21	GC14P112884	6.425566	https://www.genecards.org/cgi-bin/carddisp.pl?gene=MIR136

SERPINA3	Serpin Family A Member 3	Protein Coding	P01011	47	GC14P094612	6.425559	https://www.genecards.org/cgi-bin/carddisp.pl?gene=SERPINA3
TFAP2A	Transcription Factor AP-2 Alpha	Protein Coding	P05549	52	GC06M010393	6.423513	https://www.genecards.org/cgi-bin/carddisp.pl?gene=TFAP2A
MIR423	MicroRNA 423	RNA Gene		19	GC17P030117	6.416428	https://www.genecards.org/cgi-bin/carddisp.pl?gene=MIR423
GRP	Gastrin Releasing Peptide	Protein Coding	P07492	45	GC18P059220	6.408967	https://www.genecards.org/cgi-bin/carddisp.pl?gene=GRP
MIR200C	MicroRNA 200c	RNA Gene		23	GC12P030502	6.407468	https://www.genecards.org/cgi-bin/carddisp.pl?gene=MIR200C
MEG9	Maternally Expressed 9	RNA Gene		17	GC14P113578	6.401046	https://www.genecards.org/cgi-bin/carddisp.pl?gene=MEG9
NKX2-5	NK2 Homeobox 5	Protein Coding	P52952	49	GC05M173232	6.400319	https://www.genecards.org/cgi-bin/carddisp.pl?gene=NKX2-5
MIR150	MicroRNA 150	RNA Gene		22	GC19M049500	6.398856	https://www.genecards.org/cgi-bin/carddisp.pl?gene=MIR150
TSHR	Thyroid Stimulating Hormone Receptor	Protein Coding	P16473	52	GC14P080954	6.393366	https://www.genecards.org/cgi-bin/carddisp.pl?gene=TSHR
SOX3	SRY-Box Transcription Factor 3	Protein Coding	P41225	47	GC0XM140502	6.381923	https://www.genecards.org/cgi-bin/carddisp.pl?gene=SOX3
INHA	Inhibin Subunit Alpha	Protein Coding	P05111	47	GC02P219569	6.357432	https://www.genecards.org/cgi-bin/carddisp.pl?gene=INHA
DEAF1	DEAF1 Transcription Factor	Protein Coding	O75398	45	GC11M000644	6.343674	https://www.genecards.org/cgi-bin/carddisp.pl?gene=DEAF1

CPT1B	Carnitine Palmitoyltransferase 1B	Protein Coding	Q92523	47	GC22M070568	6.340668	https://www.genecards.org/cgi-bin/carddisp.pl?gene=CPT1B
EGFR	Epidermal Growth Factor Receptor	Protein Coding	P00533	59	GC07P055019	6.33152	https://www.genecards.org/cgi-bin/carddisp.pl?gene=EGFR
KCNK9	Potassium Two Pore Domain Channel Subfamily K Member 9	Protein Coding	Q9NPC2	50	GC08M139585	6.330558	https://www.genecards.org/cgi-bin/carddisp.pl?gene=KCNK9
CRHBP	Corticotropin Releasing Hormone Binding Protein	Protein Coding	P24387	45	GC05P076957	6.324943	https://www.genecards.org/cgi-bin/carddisp.pl?gene=CRHBP
HMGCL	3-Hydroxy-3-Methylglutaryl-CoA Lyase	Protein Coding	P35914	50	GC01M023801	6.322773	https://www.genecards.org/cgi-bin/carddisp.pl?gene=HMGCL
NOS2	Nitric Oxide Synthase 2	Protein Coding	P35228	53	GC17M027756	6.318491	https://www.genecards.org/cgi-bin/carddisp.pl?gene=NOS2
PROKR2	Prokineticin Receptor 2	Protein Coding	Q8NFJ6	46	GC20M009202	6.312331	https://www.genecards.org/cgi-bin/carddisp.pl?gene=PROKR2
TNFRSF11B	TNF Receptor Superfamily Member 11b	Protein Coding	O00300	51	GC08M118923	6.299234	https://www.genecards.org/cgi-bin/carddisp.pl?gene=TNFRSF11B
TTR	Transthyretin	Protein Coding	P02766	52	GC18P031557	6.287827	https://www.genecards.org/cgi-bin/carddisp.pl?gene=TTR
PTS	6-Pyruvoyltetrahydropterin Synthase	Protein Coding	Q03393	50	GC11P112226	6.28451	https://www.genecards.org/cgi-bin/carddisp.pl?gene=PTS
SGK1	Serum/Glucocorticoid Regulated Kinase 1	Protein Coding	O00141	53	GC06M134169	6.284202	https://www.genecards.org/cgi-bin/carddisp.pl?gene=SGK1
FOS	Fos Proto-Oncogene, AP-1 Transcription Factor Subunit	Protein Coding	P01100	55	GC14P075278	6.283136	https://www.genecards.org/cgi-bin/carddisp.pl?gene=FOS

MT-TN	Mitochondrially Encoded TRNA-Asn (AAU/C)	RNA Gene		15	GCMTM005659	6.280298	https://www.genecards.org/cgi-bin/carddisp.pl?gene=MT-TN
VPS13D	Vacuolar Protein Sorting 13 Homolog D	Protein Coding	Q5THJ4	39	GC01P012475	6.266567	https://www.genecards.org/cgi-bin/carddisp.pl?gene=VPS13D
SDHD	Succinate Dehydrogenase Complex Subunit D	Protein Coding	O14521	48	GC11P112238	6.266519	https://www.genecards.org/cgi-bin/carddisp.pl?gene=SDHD
MIR320A	MicroRNA 320a	RNA Gene		22	GC08M022292	6.244102	https://www.genecards.org/cgi-bin/carddisp.pl?gene=MIR320A
CALCA	Calcitonin Related Polypeptide Alpha	Protein Coding	P06881	47	GC11M014966	6.243373	https://www.genecards.org/cgi-bin/carddisp.pl?gene=CALCA
MIR33B	MicroRNA 33b	RNA Gene		20	GC17M017813	6.240946	https://www.genecards.org/cgi-bin/carddisp.pl?gene=MIR33B
F3	Coagulation Factor III, Tissue Factor	Protein Coding	P13726	50	GC01M094743	6.238801	https://www.genecards.org/cgi-bin/carddisp.pl?gene=F3
CFH	Complement Factor H	Protein Coding	P08603	51	GC01P196621	6.236829	https://www.genecards.org/cgi-bin/carddisp.pl?gene=CFH
FGFR2	Fibroblast Growth Factor Receptor 2	Protein Coding	P21802	59	GC10M121478	6.221732	https://www.genecards.org/cgi-bin/carddisp.pl?gene=FGFR2
MIR99A	MicroRNA 99a	RNA Gene		22	GC21P016539	6.221079	https://www.genecards.org/cgi-bin/carddisp.pl?gene=MIR99A
RPGRIP1L	RPGRIP1 Like	Protein Coding	Q68CZ1	45	GC16M053638	6.213163	https://www.genecards.org/cgi-bin/carddisp.pl?gene=RPGRIP1L
LINC01554	Long Intergenic Non-Protein Coding RNA 1554	RNA Gene	Q52M75	22	GC05P095838	6.208461	https://www.genecards.org/cgi-bin/carddisp.pl?gene=LINC01554

MIR125B1	MicroRNA 125b-1	RNA Gene		22	GC11M122100	6.193895	https://www.genecards.org/cgi-bin/carddisp.pl?gene=MIR125B1
HTR1A	5-Hydroxytryptamine Receptor 1A	Protein Coding	P08908	50	GC05M063960	6.162921	https://www.genecards.org/cgi-bin/carddisp.pl?gene=HTR1A
ESR2	Estrogen Receptor 2	Protein Coding	Q92731	53	GC14M064084	6.16078	https://www.genecards.org/cgi-bin/carddisp.pl?gene=ESR2
BMAL1	Basic Helix-Loop-Helix ARNT Like 1	Protein Coding	O00327	46	GC11P013277	6.159341	https://www.genecards.org/cgi-bin/carddisp.pl?gene=BMAL1
NPR3	Natriuretic Peptide Receptor 3	Protein Coding	P17342	50	GC05P032689	6.156561	https://www.genecards.org/cgi-bin/carddisp.pl?gene=NPR3
NPR1	Natriuretic Peptide Receptor 1	Protein Coding	P16066	51	GC01P157005	6.150833	https://www.genecards.org/cgi-bin/carddisp.pl?gene=NPR1
VANGL2	VANGL Planar Cell Polarity Protein 2	Protein Coding	Q9ULK5	47	GC01P160400	6.147169	https://www.genecards.org/cgi-bin/carddisp.pl?gene=VANGL2
PLTP	Phospholipid Transfer Protein	Protein Coding	P55058	48	GC20M045898	6.147152	https://www.genecards.org/cgi-bin/carddisp.pl?gene=PLTP
VANGL1	VANGL Planar Cell Polarity Protein 1	Protein Coding	Q8TAA9	46	GC01P115641	6.138273	https://www.genecards.org/cgi-bin/carddisp.pl?gene=VANGL1
CASP3	Caspase 3	Protein Coding	P42574	53	GC04M184627	6.126686	https://www.genecards.org/cgi-bin/carddisp.pl?gene=CASP3
MIR127	MicroRNA 127	RNA Gene		22	GC14P112881	6.12166	https://www.genecards.org/cgi-bin/carddisp.pl?gene=MIR127
TERC	Telomerase RNA Component	RNA Gene		30	GC03M169765	6.119247	https://www.genecards.org/cgi-bin/carddisp.pl?gene=TERC

RNU1-1	RNA, U1 Small Nuclear 1	RNA Gene		16	GC01M016514	6.10938	https://www.genecards.org/cgi-bin/carddisp.pl?gene=RNU1-1
RNU1-4	RNA, U1 Small Nuclear 4	RNA Gene		15	GC01P016740	6.10938	https://www.genecards.org/cgi-bin/carddisp.pl?gene=RNU1-4
RNVU1-18	RNA, Variant U1 Small Nuclear 18	RNA Gene		15	GC01M143729	6.10938	https://www.genecards.org/cgi-bin/carddisp.pl?gene=RNVU1-18
RNU1-2	RNA, U1 Small Nuclear 2	RNA Gene		14	GC01P016895	6.10938	https://www.genecards.org/cgi-bin/carddisp.pl?gene=RNU1-2
RNU1-3	RNA, U1 Small Nuclear 3	RNA Gene		13	GC01M018769	6.10938	https://www.genecards.org/cgi-bin/carddisp.pl?gene=RNU1-3
RNVU1-29	RNA, Variant U1 Small Nuclear 29	RNA Gene		8	GC01P156428	6.10938	https://www.genecards.org/cgi-bin/carddisp.pl?gene=RNVU1-29
LOC124904613	U1 Spliceosomal RNA	RNA Gene		7	GC01P157380	6.10938	https://www.genecards.org/cgi-bin/carddisp.pl?gene=LOC124904613
NPY1R	Neuropeptide Y Receptor Y1	Protein Coding	P25929	50	GC04M163323	6.108418	https://www.genecards.org/cgi-bin/carddisp.pl?gene=NPY1R
ENSG00000261069	Novel Transcript, SNORD Host	Uncategorized		9	GC15P056600	6.104661	https://www.genecards.org/cgi-bin/carddisp.pl?gene=ENSG00000261069
EPHX1	Epoxide Hydrolase 1	Protein Coding	P07099	49	GC01P225810	6.097802	https://www.genecards.org/cgi-bin/carddisp.pl?gene=EPHX1
NDUFS1	NADH:Ubiquinone Oxidoreductase Core Subunit S1	Protein Coding	P28331	49	GC02M206114	6.095544	https://www.genecards.org/cgi-bin/carddisp.pl?gene=NDUFS1
MIR139	MicroRNA 139	RNA Gene		22	GC11M072615	6.090942	https://www.genecards.org/cgi-bin/carddisp.pl?gene=MIR139

ABCB1	ATP Binding Cassette Subfamily B Member 1	Protein Coding	P08183	55	GC07M087504	6.090816	https://www.genecards.org/cgi-bin/carddisp.pl?gene=ABCB1
PHLDA2	Pleckstrin Homology Like Domain Family A Member 2	Protein Coding	Q53GA4	41	GC11M002928	6.076399	https://www.genecards.org/cgi-bin/carddisp.pl?gene=PHLDA2
NOS1	Nitric Oxide Synthase 1	Protein Coding	P29475	53	GC12M117208	6.071172	https://www.genecards.org/cgi-bin/carddisp.pl?gene=NOS1
MIR145	MicroRNA 145	RNA Gene		24	GC05P149430	6.070993	https://www.genecards.org/cgi-bin/carddisp.pl?gene=MIR145
MAPK8	Mitogen-Activated Protein Kinase 8	Protein Coding	P45983	53	GC10P048306	6.063323	https://www.genecards.org/cgi-bin/carddisp.pl?gene=MAPK8
NSD1	Nuclear Receptor Binding SET Domain Protein 1	Protein Coding	Q96L73	48	GC05P179789	6.033564	https://www.genecards.org/cgi-bin/carddisp.pl?gene=NSD1
TUG1	Taurine Up-Regulated 1	Protein Coding	A0A6I8PU40	24	GC22P030969	6.026772	https://www.genecards.org/cgi-bin/carddisp.pl?gene=TUG1
FGA	Fibrinogen Alpha Chain	Protein Coding	P02671	53	GC04M154583	6.026484	https://www.genecards.org/cgi-bin/carddisp.pl?gene=FGA
CDH23	Cadherin Related 23	Protein Coding	Q9H251	49	GC10P071396	6.02388	https://www.genecards.org/cgi-bin/carddisp.pl?gene=CDH23
PDE4D	Phosphodiesterase 4D	Protein Coding	Q08499	53	GC05M058969	6.02248	https://www.genecards.org/cgi-bin/carddisp.pl?gene=PDE4D
LGALS1	Galectin 1	Protein Coding	P09382	47	GC22P037675	6.019387	https://www.genecards.org/cgi-bin/carddisp.pl?gene=LGALS1
LRP1	LDL Receptor Related Protein 1	Protein Coding	Q07954	52	GC12P057128	6.012601	https://www.genecards.org/cgi-bin/carddisp.pl?gene=LRP1

AOC3	Amine Oxidase Copper Containing 3	Protein Coding	Q16853	50	GC17P042851	6.011595	https://www.genecards.org/cgi-bin/carddisp.pl?gene=AOC3
STX16	Syntaxin 16	Protein Coding	O14662	46	GC20P058652	6.008109	https://www.genecards.org/cgi-bin/carddisp.pl?gene=STX16
SFTPD	Surfactant Protein D	Protein Coding	P35247	48	GC10M079937	6.001472	https://www.genecards.org/cgi-bin/carddisp.pl?gene=SFTPD
CD14	CD14 Molecule	Protein Coding	P08571	51	GC05M140631	5.972487	https://www.genecards.org/cgi-bin/carddisp.pl?gene=CD14
AHR	Aryl Hydrocarbon Receptor	Protein Coding	P35869	51	GC07P016916	5.969555	https://www.genecards.org/cgi-bin/carddisp.pl?gene=AHR
FADS2	Fatty Acid Desaturase 2	Protein Coding	O95864	48	GC11P061792	5.961236	https://www.genecards.org/cgi-bin/carddisp.pl?gene=FADS2
SFTA3	Surfactant Associated 3	RNA Gene	P0C7M3	34	GC14M036474	5.957445	https://www.genecards.org/cgi-bin/carddisp.pl?gene=SFTA3
MIR26B	MicroRNA 26b	RNA Gene		23	GC02P218402	5.952154	https://www.genecards.org/cgi-bin/carddisp.pl?gene=MIR26B
CLCNKB	Chloride Voltage-Gated Channel Kb	Protein Coding	P51801	47	GC01P016268	5.935228	https://www.genecards.org/cgi-bin/carddisp.pl?gene=CLCNKB
MIR483	MicroRNA 483	RNA Gene		21	GC11M009011	5.926626	https://www.genecards.org/cgi-bin/carddisp.pl?gene=MIR483
THBD	Thrombomodulin	Protein Coding	P07204	50	GC20M023026	5.915219	https://www.genecards.org/cgi-bin/carddisp.pl?gene=THBD
ANXA5	Annexin A5	Protein Coding	P08758	51	GC04M121667	5.899414	https://www.genecards.org/cgi-bin/carddisp.pl?gene=ANXA5

CD40LG	CD40 Ligand	Protein Coding	P29965	54	GC0XP136649	5.899274	https://www.genecards.org/cgi-bin/carddisp.pl?gene=CD40LG
GAD1	Glutamate Decarboxylase 1	Protein Coding	Q99259	54	GC02P170813	5.898643	https://www.genecards.org/cgi-bin/carddisp.pl?gene=GAD1
RARA	Retinoic Acid Receptor Alpha	Protein Coding	P10276	54	GC17P040309	5.898311	https://www.genecards.org/cgi-bin/carddisp.pl?gene=RARA
PAX8	Paired Box 8	Protein Coding	Q06710	49	GC02M113215	5.897869	https://www.genecards.org/cgi-bin/carddisp.pl?gene=PAX8
LNPEP	Leucyl And Cystinyl Aminopeptidase	Protein Coding	Q9UIQ6	50	GC05P096935	5.897647	https://www.genecards.org/cgi-bin/carddisp.pl?gene=LNPEP
SLC2A3	Solute Carrier Family 2 Member 3	Protein Coding	P11169	52	GC12M007919	5.89424	https://www.genecards.org/cgi-bin/carddisp.pl?gene=SLC2A3
SIX3	SIX Homeobox 3	Protein Coding	O95343	46	GC02P044941	5.893159	https://www.genecards.org/cgi-bin/carddisp.pl?gene=SIX3
PDE8B	Phosphodiesterase 8B	Protein Coding	O95263	50	GC05P077161	5.880226	https://www.genecards.org/cgi-bin/carddisp.pl?gene=PDE8B
MIR301A	MicroRNA 301a	RNA Gene		21	GC17M059151	5.869208	https://www.genecards.org/cgi-bin/carddisp.pl?gene=MIR301A
CEBPB	CCAAT Enhancer Binding Protein Beta	Protein Coding	P17676	47	GC20P050190	5.866128	https://www.genecards.org/cgi-bin/carddisp.pl?gene=CEBPB
ETV5	ETS Variant Transcription Factor 5	Protein Coding	P41161	44	GC03M186046	5.865218	https://www.genecards.org/cgi-bin/carddisp.pl?gene=ETV5
DIO3	Iodothyronine Deiodinase 3	Protein Coding	P55073	44	GC14P113019	5.847254	https://www.genecards.org/cgi-bin/carddisp.pl?gene=DIO3

SLC27A1	Solute Carrier Family 27 Member 1	Protein Coding	Q6PCB7	44	GC19P089098	5.845687	https://www.genecards.org/cgi-bin/carddisp.pl?gene=SLC27A1
CNR2	Cannabinoid Receptor 2	Protein Coding	P34972	50	GC01M023870	5.839898	https://www.genecards.org/cgi-bin/carddisp.pl?gene=CNR2
ZNF597	Zinc Finger Protein 597	Protein Coding	Q96LX8	39	GC16M013484	5.835187	https://www.genecards.org/cgi-bin/carddisp.pl?gene=ZNF597
COG2	Component Of Oligomeric Golgi Complex 2	Protein Coding	Q14746	44	GC01P230642	5.826719	https://www.genecards.org/cgi-bin/carddisp.pl?gene=COG2
MIR107	MicroRNA 107	RNA Gene		21	GC10M089604	5.823002	https://www.genecards.org/cgi-bin/carddisp.pl?gene=MIR107
TWIST1	Twist Family BHLH Transcription Factor 1	Protein Coding	Q15672	50	GC07M019020	5.82075	https://www.genecards.org/cgi-bin/carddisp.pl?gene=TWIST1
COX15	Cytochrome C Oxidase Assembly Homolog COX15	Protein Coding	Q7KZN9	47	GC10M099696	5.818985	https://www.genecards.org/cgi-bin/carddisp.pl?gene=COX15
PCSK2	Proprotein Convertase Subtilisin/Kexin Type 2	Protein Coding	P16519	48	GC20P017226	5.816135	https://www.genecards.org/cgi-bin/carddisp.pl?gene=PCSK2
PRKACA	Protein Kinase CAMP-Activated Catalytic Subunit Alpha	Protein Coding	P17612	56	GC19M015906	5.811724	https://www.genecards.org/cgi-bin/carddisp.pl?gene=PRKACA
CYP27B1	Cytochrome P450 Family 27 Subfamily B Member 1	Protein Coding	O15528	50	GC12M058776	5.805134	https://www.genecards.org/cgi-bin/carddisp.pl?gene=CYP27B1
BMP4	Bone Morphogenetic Protein 4	Protein Coding	P12644	53	GC14M053949	5.800486	https://www.genecards.org/cgi-bin/carddisp.pl?gene=BMP4
MAPK1	Mitogen-Activated Protein Kinase 1	Protein Coding	P28482	57	GC22M021759	5.800481	https://www.genecards.org/cgi-bin/carddisp.pl?gene=MAPK1

ELN	Elastin	Protein Coding	P15502	47	GC07P074027	5.787116	https://www.genecards.org/cgi-bin/carddisp.pl?gene=ELN
KMT2D	Lysine Methyltransferase 2D	Protein Coding	O14686	47	GC12M049018	5.778403	https://www.genecards.org/cgi-bin/carddisp.pl?gene=KMT2D
ADH1B	Alcohol Dehydrogenase 1B (Class I), Beta Polypeptide	Protein Coding	P00325	48	GC04M099304	5.778026	https://www.genecards.org/cgi-bin/carddisp.pl?gene=ADH1B
TBX4	T-Box Transcription Factor 4	Protein Coding	P57082	45	GC17P086891	5.760992	https://www.genecards.org/cgi-bin/carddisp.pl?gene=TBX4
ADNP	Activity Dependent Neuroprotector Homeobox	Protein Coding	Q9H2P0	47	GC20M050888	5.738737	https://www.genecards.org/cgi-bin/carddisp.pl?gene=ADNP
PAEP	Progestagen Associated Endometrial Protein	Protein Coding	P09466	44	GC09P135561	5.708981	https://www.genecards.org/cgi-bin/carddisp.pl?gene=PAEP
MIR93	MicroRNA 93	RNA Gene		22	GC07M104208	5.707305	https://www.genecards.org/cgi-bin/carddisp.pl?gene=MIR93
TERT	Telomerase Reverse Transcriptase	Protein Coding	O14746	55	GC05M001253	5.689344	https://www.genecards.org/cgi-bin/carddisp.pl?gene=TERT
CSH1	Chorionic Somatomammotropin Hormone 1	Protein Coding	P0DML2	41	GC17M063894	5.68055	https://www.genecards.org/cgi-bin/carddisp.pl?gene=CSH1
BMP6	Bone Morphogenetic Protein 6	Protein Coding	P22004	49	GC06P007726	5.676769	https://www.genecards.org/cgi-bin/carddisp.pl?gene=BMP6
KDR	Kinase Insert Domain Receptor	Protein Coding	P35968	57	GC04M055078	5.660951	https://www.genecards.org/cgi-bin/carddisp.pl?gene=KDR
MT-CO2	Mitochondrially Encoded Cytochrome C Oxidase II	Protein Coding	P00403	38	GCMTP007587	5.656835	https://www.genecards.org/cgi-bin/carddisp.pl?gene=MT-CO2

H19-ICR	H19/IGF2 Imprinting Control Region	Functional Element		6	GC11P004521	5.656228	https://www.genecards.org/cgi-bin/carddisp.pl?gene=H19-ICR
FGF2	Fibroblast Growth Factor 2	Protein Coding	P09038	50	GC04P122826	5.633986	https://www.genecards.org/cgi-bin/carddisp.pl?gene=FGF2
PLIN2	Perilipin 2	Protein Coding	Q99541	46	GC09M019228	5.631485	https://www.genecards.org/cgi-bin/carddisp.pl?gene=PLIN2
MT-ND4L	Mitochondrially Encoded NADH:Ubiquinone Oxidoreductase Core Subunit 4L	Protein Coding	P03901	31	GCMTP010472	5.630785	https://www.genecards.org/cgi-bin/carddisp.pl?gene=MT-ND4L
MIR451A	MicroRNA 451a	RNA Gene		19	GC17M028861	5.621099	https://www.genecards.org/cgi-bin/carddisp.pl?gene=MIR451A
APOA2	Apolipoprotein A2	Protein Coding	P02652	49	GC01M161222	5.620971	https://www.genecards.org/cgi-bin/carddisp.pl?gene=APOA2
HRAS	HRas Proto-Oncogene, GTPase	Protein Coding	P01112	56	GC11M008886	5.619076	https://www.genecards.org/cgi-bin/carddisp.pl?gene=HRAS
SDHB	Succinate Dehydrogenase Complex Iron Sulfur Subunit B	Protein Coding	P21912	52	GC01M018796	5.617181	https://www.genecards.org/cgi-bin/carddisp.pl?gene=SDHB
NDUFAF2	NADH:Ubiquinone Oxidoreductase Complex Assembly Factor 2	Protein Coding	Q8N183	45	GC05P060945	5.609519	https://www.genecards.org/cgi-bin/carddisp.pl?gene=NDUFAF2
PWAR4	Prader Willi/Angelman Region RNA 4	RNA Gene		10	GC15P025211	5.607897	https://www.genecards.org/cgi-bin/carddisp.pl?gene=PWAR4
NAT2	N-Acetyltransferase 2	Protein Coding	P11245	46	GC08P018391	5.598786	https://www.genecards.org/cgi-bin/carddisp.pl?gene=NAT2
ABCG2	ATP Binding Cassette Subfamily G Member 2 (Junior Blood Group)	Protein Coding	Q9UNQ0	53	GC04M088090	5.590893	https://www.genecards.org/cgi-bin/carddisp.pl?gene=ABCG2

CCR6	C-C Motif Chemokine Receptor 6	Protein Coding	P51684	47	GC06P167111	5.581093	https://www.genecards.org/cgi-bin/carddisp.pl?gene=CCR6
ZIC2	Zic Family Member 2	Protein Coding	O95409	47	GC13P099981	5.577425	https://www.genecards.org/cgi-bin/carddisp.pl?gene=ZIC2
GATA4	GATA Binding Protein 4	Protein Coding	P43694	53	GC08P011676	5.571273	https://www.genecards.org/cgi-bin/carddisp.pl?gene=GATA4
AKR1C3	Aldo-Keto Reductase Family 1 Member C3	Protein Coding	P42330	50	GC10P005035	5.569195	https://www.genecards.org/cgi-bin/carddisp.pl?gene=AKR1C3
CYP2A6	Cytochrome P450 Family 2 Subfamily A Member 6	Protein Coding	P11509	51	GC19M040843	5.56688	https://www.genecards.org/cgi-bin/carddisp.pl?gene=CYP2A6
KIF7	Kinesin Family Member 7	Protein Coding	Q2M1P5	43	GC15M089608	5.566736	https://www.genecards.org/cgi-bin/carddisp.pl?gene=KIF7
LTA	Lymphotoxin Alpha	Protein Coding	P01374	46	GC06P111994	5.555129	https://www.genecards.org/cgi-bin/carddisp.pl?gene=LTA
MIR20A	MicroRNA 20a	RNA Gene		21	GC13P091634	5.553447	https://www.genecards.org/cgi-bin/carddisp.pl?gene=MIR20A
ICOSLG	Inducible T Cell Costimulator Ligand	Protein Coding	O75144	46	GC21M044222	5.542197	https://www.genecards.org/cgi-bin/carddisp.pl?gene=ICOSLG
FGB	Fibrinogen Beta Chain	Protein Coding	P02675	51	GC04P154625	5.524839	https://www.genecards.org/cgi-bin/carddisp.pl?gene=FGB
APOH	Apolipoprotein H	Protein Coding	P02749	48	GC17M066212	5.518464	https://www.genecards.org/cgi-bin/carddisp.pl?gene=APOH
FOXP3	Forkhead Box P3	Protein Coding	Q9BZS1	51	GC0XM049250	5.505087	https://www.genecards.org/cgi-bin/carddisp.pl?gene=FOXP3

TGFA	Transforming Growth Factor Alpha	Protein Coding	P01135	50	GC02M070447	5.493194	https://www.genecards.org/cgi-bin/carddisp.pl?gene=TGFA
SHOX	SHOX Homeobox	Protein Coding	O15266	42	GC0XP000624	5.482034	https://www.genecards.org/cgi-bin/carddisp.pl?gene=SHOX
TG	Thyroglobulin	Protein Coding	P01266	48	GC08P132866	5.479034	https://www.genecards.org/cgi-bin/carddisp.pl?gene=TG
KDM4C	Lysine Demethylase 4C	Protein Coding	Q9H3R0	46	GC09P006720	5.475925	https://www.genecards.org/cgi-bin/carddisp.pl?gene=KDM4C
GJA1	Gap Junction Protein Alpha 1	Protein Coding	P17302	54	GC06P121436	5.475874	https://www.genecards.org/cgi-bin/carddisp.pl?gene=GJA1
CREB1	CAMP Responsive Element Binding Protein 1	Protein Coding	P16220	54	GC02P207529	5.465315	https://www.genecards.org/cgi-bin/carddisp.pl?gene=CREB1
BHMT	Betaine--Homocysteine S-Methyltransferase	Protein Coding	Q93088	46	GC05P079111	5.450967	https://www.genecards.org/cgi-bin/carddisp.pl?gene=BHMT
CD46	CD46 Molecule	Protein Coding	P15529	52	GC01P207752	5.444356	https://www.genecards.org/cgi-bin/carddisp.pl?gene=CD46
CXCL10	C-X-C Motif Chemokine Ligand 10	Protein Coding	P02778	48	GC04M076021	5.440236	https://www.genecards.org/cgi-bin/carddisp.pl?gene=CXCL10
ANGPTL8	Angiopoietin Like 8	Protein Coding	Q6UXH0	37	GC19P088813	5.440227	https://www.genecards.org/cgi-bin/carddisp.pl?gene=ANGPTL8
MIR144	MicroRNA 144	RNA Gene		19	GC17M063263	5.422431	https://www.genecards.org/cgi-bin/carddisp.pl?gene=MIR144
ETFDH	Electron Transfer Flavoprotein Dehydrogenase	Protein Coding	Q16134	50	GC04P158672	5.415405	https://www.genecards.org/cgi-bin/carddisp.pl?gene=ETFDH

SREBF2	Sterol Regulatory Element Binding Transcription Factor 2	Protein Coding	Q12772	47	GC22P041833	5.413977	https://www.genecards.org/cgi-bin/carddisp.pl?gene=SREBF2
CYP17A1	Cytochrome P450 Family 17 Subfamily A Member 1	Protein Coding	P05093	53	GC10M102830	5.407848	https://www.genecards.org/cgi-bin/carddisp.pl?gene=CYP17A1
MIR486-1	MicroRNA 486-1	RNA Gene		19	GC08M041660	5.396894	https://www.genecards.org/cgi-bin/carddisp.pl?gene=MIR486-1
SRC	SRC Proto-Oncogene, Non-Receptor Tyrosine Kinase	Protein Coding	P12931	55	GC20P037344	5.391442	https://www.genecards.org/cgi-bin/carddisp.pl?gene=SRC
CASR	Calcium Sensing Receptor	Protein Coding	P41180	55	GC03P122183	5.383617	https://www.genecards.org/cgi-bin/carddisp.pl?gene=CASR
KCTD15	Potassium Channel Tetramerization Domain Containing 15	Protein Coding	Q96SI1	42	GC19P089376	5.375276	https://www.genecards.org/cgi-bin/carddisp.pl?gene=KCTD15
THRA	Thyroid Hormone Receptor Alpha	Protein Coding	P10827	52	GC17P040058	5.371792	https://www.genecards.org/cgi-bin/carddisp.pl?gene=THRA
NODAL	Nodal Growth Differentiation Factor	Protein Coding	Q96S42	46	GC10M070431	5.365159	https://www.genecards.org/cgi-bin/carddisp.pl?gene=NODAL
LGALS3	Galectin 3	Protein Coding	P17931	49	GC14P055124	5.355496	https://www.genecards.org/cgi-bin/carddisp.pl?gene=LGALS3
RMRP	RNA Component Of Mitochondrial RNA Processing Endoribonuclease	RNA Gene		25	GC09M035655	5.354333	https://www.genecards.org/cgi-bin/carddisp.pl?gene=RMRP
SEC16B	SEC16 Homolog B, Endoplasmic Reticulum Export Factor	Protein Coding	Q96JE7	42	GC01M177923	5.350115	https://www.genecards.org/cgi-bin/carddisp.pl?gene=SEC16B
HK2	Hexokinase 2	Protein Coding	P52789	51	GC02P074833	5.344597	https://www.genecards.org/cgi-bin/carddisp.pl?gene=HK2

CCR3	C-C Motif Chemokine Receptor 3	Protein Coding	P51677	49	GC03P053745	5.344004	https://www.genecards.org/cgi-bin/carddisp.pl?gene=CCR3
CSF2	Colony Stimulating Factor 2	Protein Coding	P04141	49	GC05P132073	5.339021	https://www.genecards.org/cgi-bin/carddisp.pl?gene=CSF2
ARID1A	AT-Rich Interaction Domain 1A	Protein Coding	O14497	49	GC01P026693	5.337782	https://www.genecards.org/cgi-bin/carddisp.pl?gene=ARID1A
INHBB	Inhibin Subunit Beta B	Protein Coding	P09529	46	GC02P127206	5.324619	https://www.genecards.org/cgi-bin/carddisp.pl?gene=INHBB
NPHP1	Nephrocystin 1	Protein Coding	O15259	47	GC02M110122	5.321743	https://www.genecards.org/cgi-bin/carddisp.pl?gene=NPHP1
APC	APC Regulator Of WNT Signaling Pathway	Protein Coding	P25054	53	GC05P112707	5.315515	https://www.genecards.org/cgi-bin/carddisp.pl?gene=APC
MIR133B	MicroRNA 133b	RNA Gene		22	GC06P052148	5.312303	https://www.genecards.org/cgi-bin/carddisp.pl?gene=MIR133B
APPL1	Adaptor Protein, Phosphotyrosine Interacting With PH Domain And Leucine Zipper 1	Protein Coding	Q9UKG1	48	GC03P057227	5.309589	https://www.genecards.org/cgi-bin/carddisp.pl?gene=APPL1
PHF21A	PHD Finger Protein 21A	Protein Coding	Q96BD5	47	GC11M113147	5.302721	https://www.genecards.org/cgi-bin/carddisp.pl?gene=PHF21A
MIR519D	MicroRNA 519d	RNA Gene		19	GC19P053713	5.280291	https://www.genecards.org/cgi-bin/carddisp.pl?gene=MIR519D
G6PD	Glucose-6-Phosphate Dehydrogenase	Protein Coding	P11413	54	GC0XM154583	5.268036	https://www.genecards.org/cgi-bin/carddisp.pl?gene=G6PD
PER2	Period Circadian Regulator 2	Protein Coding	O15055	47	GC02M238244	5.260629	https://www.genecards.org/cgi-bin/carddisp.pl?gene=PER2

PRKG1	Protein Kinase CGMP-Dependent 1	Protein Coding	Q13976	54	GC10P050991	5.258798	https://www.genecards.org/cgi-bin/carddisp.pl?gene=PRKG1
LGALS13	Galectin 13	Protein Coding	Q9UHV8	42	GC19P039602	5.257801	https://www.genecards.org/cgi-bin/carddisp.pl?gene=LGALS13
SUFU	SUFU Negative Regulator Of Hedgehog Signaling	Protein Coding	Q9UMX1	47	GC10P103589	5.251202	https://www.genecards.org/cgi-bin/carddisp.pl?gene=SUFU
MIR4713HG	MIR4713 Host Gene	RNA Gene		16	GC15P057537	5.234513	https://www.genecards.org/cgi-bin/carddisp.pl?gene=MIR4713HG
PIRC66	Piwi-Interacting RNA Cluster 66	RNA Gene		7	GC15U901108	5.234513	https://www.genecards.org/cgi-bin/carddisp.pl?gene=PIRC66
BRS3	Bombesin Receptor Subtype 3	Protein Coding	P32247	45	GC0XP136510	5.230474	https://www.genecards.org/cgi-bin/carddisp.pl?gene=BRS3
HYMAI	Hydatidiform Mole Associated And Imprinted	RNA Gene		23	GC06M144002	5.214425	https://www.genecards.org/cgi-bin/carddisp.pl?gene=HYMAI
IL5	Interleukin 5	Protein Coding	P05113	49	GC05M132541	5.212819	https://www.genecards.org/cgi-bin/carddisp.pl?gene=IL5
BTD	Biotinidase	Protein Coding	P43251	50	GC03P018498	5.211876	https://www.genecards.org/cgi-bin/carddisp.pl?gene=BTD
FABP1	Fatty Acid Binding Protein 1	Protein Coding	P07148	47	GC02M088122	5.21185	https://www.genecards.org/cgi-bin/carddisp.pl?gene=FABP1
HLA-C	Major Histocompatibility Complex, Class I, C	Protein Coding	P10321	48	GC06M083904	5.208886	https://www.genecards.org/cgi-bin/carddisp.pl?gene=HLA-C
XDH	Xanthine Dehydrogenase	Protein Coding	P47989	51	GC02M031334	5.201622	https://www.genecards.org/cgi-bin/carddisp.pl?gene=XDH

NPC1	NPC Intracellular Cholesterol Transporter 1	Protein Coding	O15118	53	GC18M023506	5.192736	https://www.genecards.org/cgi-bin/carddisp.pl?gene=NPC1
S100A9	S100 Calcium Binding Protein A9	Protein Coding	P06702	47	GC01P153357	5.187632	https://www.genecards.org/cgi-bin/carddisp.pl?gene=S100A9
MIR494	MicroRNA 494	RNA Gene		18	GC14P113612	5.184448	https://www.genecards.org/cgi-bin/carddisp.pl?gene=MIR494
FABP3	Fatty Acid Binding Protein 3	Protein Coding	P05413	47	GC01M031365	5.18181	https://www.genecards.org/cgi-bin/carddisp.pl?gene=FABP3
MIR23B	MicroRNA 23b	RNA Gene		21	GC09P095085	5.171393	https://www.genecards.org/cgi-bin/carddisp.pl?gene=MIR23B
GABRB3	Gamma-Aminobutyric Acid Type A Receptor Subunit Beta3	Protein Coding	P28472	53	GC15M026543	5.164236	https://www.genecards.org/cgi-bin/carddisp.pl?gene=GABRB3
TLX1NB	TLX1 Neighbor	RNA Gene	P0CAT3	25	GC10M101090	5.163291	https://www.genecards.org/cgi-bin/carddisp.pl?gene=TLX1NB
ALPP	Alkaline Phosphatase, Placental	Protein Coding	P05187	48	GC02P232378	5.148932	https://www.genecards.org/cgi-bin/carddisp.pl?gene=ALPP
TPO	Thyroid Peroxidase	Protein Coding	P07202	52	GC02P001374	5.147863	https://www.genecards.org/cgi-bin/carddisp.pl?gene=TPO
RHOA	Ras Homolog Family Member A	Protein Coding	P61586	54	GC03M049359	5.141097	https://www.genecards.org/cgi-bin/carddisp.pl?gene=RHOA
CDKN2B	Cyclin Dependent Kinase Inhibitor 2B	Protein Coding	P42772	50	GC09M022002	5.137381	https://www.genecards.org/cgi-bin/carddisp.pl?gene=CDKN2B
PCSK9	Proprotein Convertase Subtilisin/Kexin Type 9	Protein Coding	Q8NBP7	54	GC01P055039	5.136763	https://www.genecards.org/cgi-bin/carddisp.pl?gene=PCSK9

CDH1	Cadherin 1	Protein Coding	P12830	53	GC16P068737	5.120307	https://www.genecards.org/cgi-bin/carddisp.pl?gene=CDH1
MIR200A	MicroRNA 200a	RNA Gene		21	GC01P006058	5.119183	https://www.genecards.org/cgi-bin/carddisp.pl?gene=MIR200A
CCR5	C-C Motif Chemokine Receptor 5	Protein Coding	P51681	50	GC03P053750	5.115474	https://www.genecards.org/cgi-bin/carddisp.pl?gene=CCR5
NR5A1	Nuclear Receptor Subfamily 5 Group A Member 1	Protein Coding	Q13285	53	GC09M124481	5.110287	https://www.genecards.org/cgi-bin/carddisp.pl?gene=NR5A1
UBE2A	Ubiquitin Conjugating Enzyme E2 A	Protein Coding	P49459	48	GC0XP119810	5.108734	https://www.genecards.org/cgi-bin/carddisp.pl?gene=UBE2A
EHMT1	Euchromatic Histone Lysine Methyltransferase 1	Protein Coding	Q9H9B1	50	GC09P137618	5.106753	https://www.genecards.org/cgi-bin/carddisp.pl?gene=EHMT1
MT-TS2	Mitochondrially Encoded TRNA-Ser (AGU/C) 2	RNA Gene		14	GCMTP012215	5.10232	https://www.genecards.org/cgi-bin/carddisp.pl?gene=MT-TS2
CYP11A1	Cytochrome P450 Family 11 Subfamily A Member 1	Protein Coding	P05108	52	GC15M074337	5.098168	https://www.genecards.org/cgi-bin/carddisp.pl?gene=CYP11A1
ITGAX	Integrin Subunit Alpha X	Protein Coding	P20702	50	GC16P054252	5.097096	https://www.genecards.org/cgi-bin/carddisp.pl?gene=ITGAX
MIR151A	MicroRNA 151a	RNA Gene		21	GC08M140857	5.089459	https://www.genecards.org/cgi-bin/carddisp.pl?gene=MIR151A
HLA-DQA1	Major Histocompatibility Complex, Class II, DQ Alpha 1	Protein Coding	P01909	46	GC06P112031	5.088503	https://www.genecards.org/cgi-bin/carddisp.pl?gene=HLA-DQA1
MIR424	MicroRNA 424	RNA Gene		20	GC0XM134736	5.074601	https://www.genecards.org/cgi-bin/carddisp.pl?gene=MIR424

CD8A	CD8 Subunit Alpha	Protein Coding	P01732	51	GC02M086784	5.057577	https://www.genecards.org/cgi-bin/carddisp.pl?gene=CD8A
RB1	RB Transcriptional Corepressor 1	Protein Coding	P06400	52	GC13P048303	5.051846	https://www.genecards.org/cgi-bin/carddisp.pl?gene=RB1
HLA-B	Major Histocompatibility Complex, Class I, B	Protein Coding	P01889	50	GC06M083905	5.051411	https://www.genecards.org/cgi-bin/carddisp.pl?gene=HLA-B
PAX2	Paired Box 2	Protein Coding	Q02962	50	GC10P100735	5.049158	https://www.genecards.org/cgi-bin/carddisp.pl?gene=PAX2
CSF1	Colony Stimulating Factor 1	Protein Coding	P09603	48	GC01P109911	5.047069	https://www.genecards.org/cgi-bin/carddisp.pl?gene=CSF1
CP	Ceruloplasmin	Protein Coding	P00450	52	GC03M149162	5.040213	https://www.genecards.org/cgi-bin/carddisp.pl?gene=CP
ANPEP	Alanyl Aminopeptidase, Membrane	Protein Coding	P15144	53	GC15M089784	5.038225	https://www.genecards.org/cgi-bin/carddisp.pl?gene=ANPEP
SRD5A1	Steroid 5 Alpha-Reductase 1	Protein Coding	P18405	48	GC05P006633	5.033588	https://www.genecards.org/cgi-bin/carddisp.pl?gene=SRD5A1
TET3	Tet Methylcytosine Dioxygenase 3	Protein Coding	O43151	45	GC02P073986	5.026772	https://www.genecards.org/cgi-bin/carddisp.pl?gene=TET3
IL2RA	Interleukin 2 Receptor Subunit Alpha	Protein Coding	P01589	54	GC10M006010	5.021492	https://www.genecards.org/cgi-bin/carddisp.pl?gene=IL2RA
CDKN3	Cyclin Dependent Kinase Inhibitor 3	Protein Coding	Q16667	46	GC14P054398	5.0082	https://www.genecards.org/cgi-bin/carddisp.pl?gene=CDKN3
NAA60	N-Alpha-Acetyltransferase 60, NatF Catalytic Subunit	Protein Coding	Q9H7X0	39	GC16P003443	5.003081	https://www.genecards.org/cgi-bin/carddisp.pl?gene=NAA60

TGFB3	Transforming Growth Factor Beta 3	Protein Coding	P10600	52	GC14M075958	4.999937	https://www.genecards.org/cgi-bin/carddisp.pl?gene=TGFB3
GABRG3	Gamma-Aminobutyric Acid Type A Receptor Subunit Gamma3	Protein Coding	Q99928	47	GC15P026971	4.972946	https://www.genecards.org/cgi-bin/carddisp.pl?gene=GABRG3
CUL4B	Cullin 4B	Protein Coding	Q13620	47	GC0XM120524	4.957582	https://www.genecards.org/cgi-bin/carddisp.pl?gene=CUL4B
AGTR2	Angiotensin II Receptor Type 2	Protein Coding	P50052	47	GC0XP116170	4.938807	https://www.genecards.org/cgi-bin/carddisp.pl?gene=AGTR2
SPX	Spexin Hormone	Protein Coding	Q9BT56	34	GC12P021526	4.928893	https://www.genecards.org/cgi-bin/carddisp.pl?gene=SPX
CDKL5	Cyclin Dependent Kinase Like 5	Protein Coding	O76039	47	GC0XP018425	4.926317	https://www.genecards.org/cgi-bin/carddisp.pl?gene=CDKL5
HLA-A	Major Histocompatibility Complex, Class I, A	Protein Coding	P04439	50	GC06P111952	4.922143	https://www.genecards.org/cgi-bin/carddisp.pl?gene=HLA-A
SLC12A3	Solute Carrier Family 12 Member 3	Protein Coding	P55017	51	GC16P056865	4.918425	https://www.genecards.org/cgi-bin/carddisp.pl?gene=SLC12A3
CTCF	CCCTC-Binding Factor	Protein Coding	P49711	51	GC16P067563	4.903754	https://www.genecards.org/cgi-bin/carddisp.pl?gene=CTCF
FDFT1	Farnesyl-Diphosphate Farnesyltransferase 1	Protein Coding	P37268	50	GC08P011795	4.90321	https://www.genecards.org/cgi-bin/carddisp.pl?gene=FDFT1
PCK1	Phosphoenolpyruvate Carboxykinase 1	Protein Coding	P35558	52	GC20P057561	4.899157	https://www.genecards.org/cgi-bin/carddisp.pl?gene=PCK1
STAR	Steroidogenic Acute Regulatory Protein	Protein Coding	P49675	48	GC08M038179	4.897914	https://www.genecards.org/cgi-bin/carddisp.pl?gene=STAR

KRT18	Keratin 18	Protein Coding	P05783	54	GC12P052948	4.896292	https://www.genecards.org/cgi-bin/carddisp.pl?gene=KRT18
MIR455	MicroRNA 455	RNA Gene		20	GC09P114209	4.893435	https://www.genecards.org/cgi-bin/carddisp.pl?gene=MIR455
CASP1	Caspase 1	Protein Coding	P29466	53	GC11M105025	4.89327	https://www.genecards.org/cgi-bin/carddisp.pl?gene=CASP1
CASP8	Caspase 8	Protein Coding	Q14790	56	GC02P201233	4.886509	https://www.genecards.org/cgi-bin/carddisp.pl?gene=CASP8
LTF	Lactotransferrin	Protein Coding	P02788	50	GC03M046435	4.885273	https://www.genecards.org/cgi-bin/carddisp.pl?gene=LTF
TRE-TTC3-1	TRNA-Glu (Anticodon TTC) 3-1	RNA Gene		10	GC01P018717	4.877283	https://www.genecards.org/cgi-bin/carddisp.pl?gene=TRE-TTC3-1
KRAS	KRAS Proto-Oncogene, GTPase	Protein Coding	P01116	57	GC12M026163	4.877003	https://www.genecards.org/cgi-bin/carddisp.pl?gene=KRAS
JUN	Jun Proto-Oncogene, AP-1 Transcription Factor Subunit	Protein Coding	P05412	53	GC01M058780	4.874602	https://www.genecards.org/cgi-bin/carddisp.pl?gene=JUN
WFDC21P	WAP Four-Disulfide Core Domain 21, Pseudogene	Pseudogene		15	GC17M065753	4.874564	https://www.genecards.org/cgi-bin/carddisp.pl?gene=WFDC21P
COL3A1	Collagen Type III Alpha 1 Chain	Protein Coding	P02461	52	GC02P188974	4.866763	https://www.genecards.org/cgi-bin/carddisp.pl?gene=COL3A1
NF1	Neurofibromin 1	Protein Coding	P21359	52	GC17P031094	4.863272	https://www.genecards.org/cgi-bin/carddisp.pl?gene=NF1
HMGA2	High Mobility Group AT-Hook 2	Protein Coding	P52926	47	GC12P065824	4.853709	https://www.genecards.org/cgi-bin/carddisp.pl?gene=HMGA2

PCAT1	Prostate Cancer Associated Transcript 1	RNA Gene		19	GC08P126553	4.85286	https://www.genecards.org/cgi-bin/carddisp.pl?gene=PCAT1
TGIF1	TGFB Induced Factor Homeobox 1	Protein Coding	Q15583	50	GC18P003411	4.83961	https://www.genecards.org/cgi-bin/carddisp.pl?gene=TGIF1
MIR106A	MicroRNA 106a	RNA Gene		20	GC0XM134399	4.832904	https://www.genecards.org/cgi-bin/carddisp.pl?gene=MIR106A
MEN1	Menin 1	Protein Coding	O00255	48	GC11M064803	4.830105	https://www.genecards.org/cgi-bin/carddisp.pl?gene=MEN1
CISH	Cytokine Inducible SH2 Containing Protein	Protein Coding	Q9NSE2	49	GC03M052771	4.825144	https://www.genecards.org/cgi-bin/carddisp.pl?gene=CISH
FOXF1	Forkhead Box F1	Protein Coding	Q12946	45	GC16P086510	4.82239	https://www.genecards.org/cgi-bin/carddisp.pl?gene=FOXF1
EPO	Erythropoietin	Protein Coding	P01588	47	GC07P100720	4.82097	https://www.genecards.org/cgi-bin/carddisp.pl?gene=EPO
GPBAR1	G Protein-Coupled Bile Acid Receptor 1	Protein Coding	Q8TDU6	44	GC02P218259	4.811228	https://www.genecards.org/cgi-bin/carddisp.pl?gene=GPBAR1
IGF2-AS	IGF2 Antisense RNA	RNA Gene	Q6U949	29	GC11P002140	4.808746	https://www.genecards.org/cgi-bin/carddisp.pl?gene=IGF2-AS
TCN2	Transcobalamin 2	Protein Coding	P20062	47	GC22P056427	4.805315	https://www.genecards.org/cgi-bin/carddisp.pl?gene=TCN2
PLAG1	PLAG1 Zinc Finger	Protein Coding	Q6DJT9	45	GC08M056507	4.798771	https://www.genecards.org/cgi-bin/carddisp.pl?gene=PLAG1
COA3	Cytochrome C Oxidase Assembly Factor 3	Protein Coding	Q9Y2R0	39	GC17M042795	4.7954	https://www.genecards.org/cgi-bin/carddisp.pl?gene=COA3

SETD2	SET Domain Containing 2, Histone Lysine Methyltransferase	Protein Coding	Q9BYW2	51	GC03M047033	4.795158	https://www.genecards.org/cgi-bin/carddisp.pl?gene=SETD2
FABP12	Fatty Acid Binding Protein 12	Protein Coding	A6NFH5	36	GC08M081524	4.791874	https://www.genecards.org/cgi-bin/carddisp.pl?gene=FABP12
HAMP	Hepcidin Antimicrobial Peptide	Protein Coding	P81172	47	GC19P089415	4.782485	https://www.genecards.org/cgi-bin/carddisp.pl?gene=HAMP
HCCAT5	Hepatocellular Carcinoma Associated Transcript 5	RNA Gene		17	GC16P073092	4.761596	https://www.genecards.org/cgi-bin/carddisp.pl?gene=HCCAT5
SMO	Smoothened, Frizzled Class Receptor	Protein Coding	Q99835	55	GC07P133919	4.760953	https://www.genecards.org/cgi-bin/carddisp.pl?gene=SMO
ANCR	Angelman Syndrome Chromosome Region	Uncategorized		5	GC15U990003	4.75512	https://www.genecards.org/cgi-bin/carddisp.pl?gene=ANCR
MIRLET7I	MicroRNA Let-7i	RNA Gene		22	GC12P062638	4.754233	https://www.genecards.org/cgi-bin/carddisp.pl?gene=MIRLET7I
MIR193B	MicroRNA 193b	RNA Gene		22	GC16P052732	4.7433	https://www.genecards.org/cgi-bin/carddisp.pl?gene=MIR193B
TNFSF11	TNF Superfamily Member 11	Protein Coding	O14788	53	GC13P042562	4.737309	https://www.genecards.org/cgi-bin/carddisp.pl?gene=TNFSF11
PHB1	Prohibitin 1	Protein Coding	P35232	50	GC17M064671	4.736535	https://www.genecards.org/cgi-bin/carddisp.pl?gene=PHB1
POR	Cytochrome P450 Oxidoreductase	Protein Coding	P16435	52	GC07P075899	4.725114	https://www.genecards.org/cgi-bin/carddisp.pl?gene=POR
WNT5A	Wnt Family Member 5A	Protein Coding	P41221	55	GC03M055465	4.715602	https://www.genecards.org/cgi-bin/carddisp.pl?gene=WNT5A

UCN2	Urocortin 2	Protein Coding	Q96RP3	36	GC03M048561	4.707733	https://www.genecards.org/cgi-bin/carddisp.pl?gene=UCN2
CHGA	Chromogranin A	Protein Coding	P10645	47	GC14P092967	4.70379	https://www.genecards.org/cgi-bin/carddisp.pl?gene=CHGA
SPRY4-IT1	SPRY4 Intronic Transcript 1	RNA Gene		15	GC05M142318	4.699006	https://www.genecards.org/cgi-bin/carddisp.pl?gene=SPRY4-IT1
NRF1	Nuclear Respiratory Factor 1	Protein Coding	Q16656	48	GC07P129611	4.692935	https://www.genecards.org/cgi-bin/carddisp.pl?gene=NRF1
COL2A1	Collagen Type II Alpha 1 Chain	Protein Coding	P02458	54	GC12M047972	4.684935	https://www.genecards.org/cgi-bin/carddisp.pl?gene=COL2A1
MAPK3	Mitogen-Activated Protein Kinase 3	Protein Coding	P27361	53	GC16M041633	4.674434	https://www.genecards.org/cgi-bin/carddisp.pl?gene=MAPK3
FSHR	Follicle Stimulating Hormone Receptor	Protein Coding	P23945	52	GC02M048962	4.673252	https://www.genecards.org/cgi-bin/carddisp.pl?gene=FSHR
GC	GC Vitamin D Binding Protein	Protein Coding	P02774	48	GC04M071741	4.666814	https://www.genecards.org/cgi-bin/carddisp.pl?gene=GC
ADRA2B	Adrenoceptor Alpha 2B	Protein Coding	P18089	48	GC02M096112	4.666601	https://www.genecards.org/cgi-bin/carddisp.pl?gene=ADRA2B
DMPK	DM1 Protein Kinase	Protein Coding	Q09013	51	GC19M045769	4.661617	https://www.genecards.org/cgi-bin/carddisp.pl?gene=DMPK
MT-TH	Mitochondrially Encoded TRNA-His (CAU/C)	RNA Gene		15	GCMTP012140	4.660393	https://www.genecards.org/cgi-bin/carddisp.pl?gene=MT-TH
SDHC	Succinate Dehydrogenase Complex Subunit C	Protein Coding	Q99643	47	GC01P161314	4.660008	https://www.genecards.org/cgi-bin/carddisp.pl?gene=SDHC

RAB4B-EGLN2	RAB4B-EGLN2 Readthrough (NMD Candidate)	RNA Gene		16	GC19P040778	4.654914	https://www.genecards.org/cgi-bin/carddisp.pl?gene=RAB4B-EGLN2
CALR	Calreticulin	Protein Coding	P27797	55	GC19P012938	4.654411	https://www.genecards.org/cgi-bin/carddisp.pl?gene=CALR
SPINK1	Serine Peptidase Inhibitor Kazal Type 1	Protein Coding	P00995	46	GC05M147825	4.652958	https://www.genecards.org/cgi-bin/carddisp.pl?gene=SPINK1
FASLG	Fas Ligand	Protein Coding	P48023	52	GC01P172659	4.648318	https://www.genecards.org/cgi-bin/carddisp.pl?gene=FASLG
ECHS1	Enoyl-CoA Hydratase, Short Chain 1	Protein Coding	P30084	49	GC10M133362	4.64826	https://www.genecards.org/cgi-bin/carddisp.pl?gene=ECHS1
STAT1	Signal Transducer And Activator Of Transcription 1	Protein Coding	P42224	57	GC02M190908	4.644912	https://www.genecards.org/cgi-bin/carddisp.pl?gene=STAT1
CCL3	C-C Motif Chemokine Ligand 3	Protein Coding	P10147	44	GC17M036088	4.640441	https://www.genecards.org/cgi-bin/carddisp.pl?gene=CCL3
MAOB	Monoamine Oxidase B	Protein Coding	P27338	48	GC0XM043766	4.640275	https://www.genecards.org/cgi-bin/carddisp.pl?gene=MAOB
NIPBL	NIPBL Cohesin Loading Factor	Protein Coding	Q6KC79	47	GC05P036876	4.636902	https://www.genecards.org/cgi-bin/carddisp.pl?gene=NIPBL
CDH2	Cadherin 2	Protein Coding	P19022	55	GC18M031138	4.636435	https://www.genecards.org/cgi-bin/carddisp.pl?gene=CDH2
OLR1	Oxidized Low Density Lipoprotein Receptor 1	Protein Coding	P78380	46	GC12M025972	4.630818	https://www.genecards.org/cgi-bin/carddisp.pl?gene=OLR1
BMP2	Bone Morphogenetic Protein 2	Protein Coding	P12643	50	GC20P006767	4.630643	https://www.genecards.org/cgi-bin/carddisp.pl?gene=BMP2

IGHE	Immunoglobulin Heavy Constant Epsilon	Protein Coding	P01854	35	GC14M116619	4.626828	https://www.genecards.org/cgi-bin/carddisp.pl?gene=IGHE
GATA3	GATA Binding Protein 3	Protein Coding	P23771	54	GC10P008045	4.624721	https://www.genecards.org/cgi-bin/carddisp.pl?gene=GATA3
HAVCR2	Hepatitis A Virus Cellular Receptor 2	Protein Coding	Q8TDQ0	49	GC05M157063	4.623992	https://www.genecards.org/cgi-bin/carddisp.pl?gene=HAVCR2
HMGB1	High Mobility Group Box 1	Protein Coding	P09429	52	GC13M030456	4.623461	https://www.genecards.org/cgi-bin/carddisp.pl?gene=HMGB1
HMGA1	High Mobility Group AT-Hook 1	Protein Coding	P17096	50	GC06P112124	4.616968	https://www.genecards.org/cgi-bin/carddisp.pl?gene=HMGA1
MIR590	MicroRNA 590	RNA Gene		21	GC07P074191	4.615949	https://www.genecards.org/cgi-bin/carddisp.pl?gene=MIR590
CCL4	C-C Motif Chemokine Ligand 4	Protein Coding	P13236	44	GC17P036103	4.615418	https://www.genecards.org/cgi-bin/carddisp.pl?gene=CCL4
BRCA1	BRCA1 DNA Repair Associated	Protein Coding	P38398	54	GC17M043044	4.600372	https://www.genecards.org/cgi-bin/carddisp.pl?gene=BRCA1
RNU6-1	RNA, U6 Small Nuclear 1	RNA Gene		19	GC15M067839	4.599971	https://www.genecards.org/cgi-bin/carddisp.pl?gene=RNU6-1
RNU6-2	RNA, U6 Small Nuclear 2	RNA Gene		16	GC19P008282	4.599971	https://www.genecards.org/cgi-bin/carddisp.pl?gene=RNU6-2
RNU6-9	RNA, U6 Small Nuclear 9	RNA Gene		14	GC19P008272	4.599971	https://www.genecards.org/cgi-bin/carddisp.pl?gene=RNU6-9
OPRM1	Opioid Receptor Mu 1	Protein Coding	P35372	52	GC06P154196	4.597624	https://www.genecards.org/cgi-bin/carddisp.pl?gene=OPRM1

SNURF	SNRPN Upstream Open Reading Frame	Protein Coding	Q9Y675	36	GC15P024954	4.596743	https://www.genecards.org/cgi-bin/carddisp.pl?gene=SNURF
GFPT1	Glutamine--Fructose-6-Phosphate Transaminase 1	Protein Coding	Q06210	50	GC02M069319	4.595881	https://www.genecards.org/cgi-bin/carddisp.pl?gene=GFPT1
MIR103A1	MicroRNA 103a-1	RNA Gene		19	GC05M168560	4.593533	https://www.genecards.org/cgi-bin/carddisp.pl?gene=MIR103A1
PBX1	PBX Homeobox 1	Protein Coding	P40424	52	GC01P164524	4.587352	https://www.genecards.org/cgi-bin/carddisp.pl?gene=PBX1
PSMD12	Proteasome 26S Subunit, Non-ATPase 12	Protein Coding	O00232	46	GC17M067337	4.575547	https://www.genecards.org/cgi-bin/carddisp.pl?gene=PSMD12
HGD	Homogentisate 1,2-Dioxygenase	Protein Coding	Q93099	48	GC03M120628	4.570209	https://www.genecards.org/cgi-bin/carddisp.pl?gene=HGD
RNU6-7	RNA, U6 Small Nuclear 7	RNA Gene		15	GC14P032202	4.568453	https://www.genecards.org/cgi-bin/carddisp.pl?gene=RNU6-7
RNU6-8	RNA, U6 Small Nuclear 8	RNA Gene		15	GC14M032203	4.568453	https://www.genecards.org/cgi-bin/carddisp.pl?gene=RNU6-8
RNU6-1-001		RNA Gene		8	GC10P013218	4.568453	https://www.genecards.org/cgi-bin/carddisp.pl?gene=RNU6-1-001
RNU6-1-002		RNA Gene		7	GC02M174560	4.568453	https://www.genecards.org/cgi-bin/carddisp.pl?gene=RNU6-1-002
RNU6-1-003		RNA Gene		6	GC03P181233	4.568453	https://www.genecards.org/cgi-bin/carddisp.pl?gene=RNU6-1-003
RNU6-1-004		RNA Gene		6	GC0XM141120	4.568453	https://www.genecards.org/cgi-bin/carddisp.pl?gene=RNU6-1-004

MIR574	MicroRNA 574	RNA Gene		21	GC04P039029	4.56816	https://www.genecards.org/cgi-bin/carddisp.pl?gene=MIR574
CTNNBL1	Catenin Beta Like 1	Protein Coding	Q8WYA6	45	GC20P037693	4.561633	https://www.genecards.org/cgi-bin/carddisp.pl?gene=CTNNBL1
SAA1	Serum Amyloid A1	Protein Coding	P0DJI8	43	GC11P018886	4.560456	https://www.genecards.org/cgi-bin/carddisp.pl?gene=SAA1
PRKN	Parkin RBR E3 Ubiquitin Protein Ligase	Protein Coding	O60260	53	GC06M161348	4.554565	https://www.genecards.org/cgi-bin/carddisp.pl?gene=PRKN
MMP1	Matrix Metallopeptidase 1	Protein Coding	P03956	54	GC11M114281	4.554159	https://www.genecards.org/cgi-bin/carddisp.pl?gene=MMP1
BAIAP2L1	BAR/IMD Domain Containing Adaptor Protein 2 Like 1	Protein Coding	Q9UHR4	43	GC07M098294	4.545568	https://www.genecards.org/cgi-bin/carddisp.pl?gene=BAIAP2L1
NUCB2	Nucleobindin 2	Protein Coding	P80303	45	GC11P017655	4.543168	https://www.genecards.org/cgi-bin/carddisp.pl?gene=NUCB2
GJB2	Gap Junction Protein Beta 2	Protein Coding	P29033	50	GC13M020187	4.539543	https://www.genecards.org/cgi-bin/carddisp.pl?gene=GJB2
HTT	Huntingtin	Protein Coding	P42858	48	GC04P003041	4.535595	https://www.genecards.org/cgi-bin/carddisp.pl?gene=HTT
MT-TF	Mitochondrially Encoded TRNA-Phe (UUU/C)	RNA Gene		16	GCMTP000580	4.529284	https://www.genecards.org/cgi-bin/carddisp.pl?gene=MT-TF
MIR196A1	MicroRNA 196a-1	RNA Gene		22	GC17M048632	4.522091	https://www.genecards.org/cgi-bin/carddisp.pl?gene=MIR196A1
MIR212	MicroRNA 212	RNA Gene		21	GC17M002050	4.514486	https://www.genecards.org/cgi-bin/carddisp.pl?gene=MIR212

FKBP5	FKBP Prolyl Isomerase 5	Protein Coding	Q13451	52	GC06M084086	4.511695	https://www.genecards.org/cgi-bin/carddisp.pl?gene=FKBP5
PSG1	Pregnancy Specific Beta-1-Glycoprotein 1	Protein Coding	P11464	42	GC19M042866	4.5075	https://www.genecards.org/cgi-bin/carddisp.pl?gene=PSG1
JPX	JPX Transcript, XIST Activator	RNA Gene		22	GC0XP074063	4.491867	https://www.genecards.org/cgi-bin/carddisp.pl?gene=JPX
CFI	Complement Factor I	Protein Coding	P05156	51	GC04M109732	4.484002	https://www.genecards.org/cgi-bin/carddisp.pl?gene=CFI
F13A1	Coagulation Factor XIII A Chain	Protein Coding	P00488	51	GC06M006144	4.483747	https://www.genecards.org/cgi-bin/carddisp.pl?gene=F13A1
DNMT1	DNA Methyltransferase 1	Protein Coding	P26358	56	GC19M010133	4.475481	https://www.genecards.org/cgi-bin/carddisp.pl?gene=DNMT1
MIR133A1	MicroRNA 133a-1	RNA Gene		17	GC18M031068	4.469907	https://www.genecards.org/cgi-bin/carddisp.pl?gene=MIR133A1
MIR141	MicroRNA 141	RNA Gene		22	GC12P030503	4.469351	https://www.genecards.org/cgi-bin/carddisp.pl?gene=MIR141
CHD7	Chromodomain Helicase DNA Binding Protein 7	Protein Coding	Q9P2D1	49	GC08P060678	4.469001	https://www.genecards.org/cgi-bin/carddisp.pl?gene=CHD7
ZDBF2	Zinc Finger DBF-Type Containing 2	Protein Coding	Q9HCK1	35	GC02P206274	4.468475	https://www.genecards.org/cgi-bin/carddisp.pl?gene=ZDBF2
MIR98	MicroRNA 98	RNA Gene		20	GC0XM053669	4.462134	https://www.genecards.org/cgi-bin/carddisp.pl?gene=MIR98
ITGB2	Integrin Subunit Beta 2	Protein Coding	P05107	54	GC21M044885	4.460977	https://www.genecards.org/cgi-bin/carddisp.pl?gene=ITGB2

ATXN2	Ataxin 2	Protein Coding	Q99700	47	GC12M111443	4.458872	https://www.genecards.org/cgi-bin/carddisp.pl?gene=ATXN2
HHEX	Hematopoietically Expressed Homeobox	Protein Coding	Q03014	45	GC10P092689	4.452312	https://www.genecards.org/cgi-bin/carddisp.pl?gene=HHEX
CD274	CD274 Molecule	Protein Coding	Q9NZQ7	49	GC09P005450	4.452174	https://www.genecards.org/cgi-bin/carddisp.pl?gene=CD274
F9	Coagulation Factor IX	Protein Coding	P00740	52	GC0XP139530	4.449217	https://www.genecards.org/cgi-bin/carddisp.pl?gene=F9
ESRRB	Estrogen Related Receptor Beta	Protein Coding	O95718	52	GC14P076310	4.437549	https://www.genecards.org/cgi-bin/carddisp.pl?gene=ESRRB
GP1BA	Glycoprotein Ib Platelet Subunit Alpha	Protein Coding	P07359	52	GC17P004932	4.430165	https://www.genecards.org/cgi-bin/carddisp.pl?gene=GP1BA
PCBD1	Pterin-4 Alpha-Carbinolamine Dehydratase 1	Protein Coding	P61457	49	GC10M070882	4.429541	https://www.genecards.org/cgi-bin/carddisp.pl?gene=PCBD1
OCRL	OCRL Inositol Polyphosphate-5-Phosphatase	Protein Coding	Q01968	48	GC0XP129539	4.428105	https://www.genecards.org/cgi-bin/carddisp.pl?gene=OCRL
INHBA	Inhibin Subunit Beta A	Protein Coding	P08476	50	GC07M041670	4.426697	https://www.genecards.org/cgi-bin/carddisp.pl?gene=INHBA
NPHP4	Nephrocystin 4	Protein Coding	O75161	45	GC01M010740	4.418208	https://www.genecards.org/cgi-bin/carddisp.pl?gene=NPHP4
MET	MET Proto-Oncogene, Receptor Tyrosine Kinase	Protein Coding	P08581	58	GC07P116672	4.414143	https://www.genecards.org/cgi-bin/carddisp.pl?gene=MET
MIR24-1	MicroRNA 24-1	RNA Gene		19	GC09P095086	4.413852	https://www.genecards.org/cgi-bin/carddisp.pl?gene=MIR24-1

CYP2D6	Cytochrome P450 Family 2 Subfamily D Member 6	Protein Coding	P10635	50	GC22M042126	4.412963	https://www.genecards.org/cgi-bin/carddisp.pl?gene=CYP2D6
CTLA4	Cytotoxic T-Lymphocyte Associated Protein 4	Protein Coding	P16410	52	GC02P203854	4.412017	https://www.genecards.org/cgi-bin/carddisp.pl?gene=CTLA4
MT-TM	Mitochondrially Encoded TRNA-Met (AUA/G)	RNA Gene		14	GCMTP004404	4.404584	https://www.genecards.org/cgi-bin/carddisp.pl?gene=MT-TM
AVPR1A	Arginine Vasopressin Receptor 1A	Protein Coding	P37288	50	GC12M063142	4.40273	https://www.genecards.org/cgi-bin/carddisp.pl?gene=AVPR1A
B2M	Beta-2-Microglobulin	Protein Coding	P61769	53	GC15P044711	4.391316	https://www.genecards.org/cgi-bin/carddisp.pl?gene=B2M
STAT5A	Signal Transducer And Activator Of Transcription 5A	Protein Coding	P42229	52	GC17P042287	4.389139	https://www.genecards.org/cgi-bin/carddisp.pl?gene=STAT5A
PDE5A	Phosphodiesterase 5A	Protein Coding	O76074	49	GC04M119494	4.380291	https://www.genecards.org/cgi-bin/carddisp.pl?gene=PDE5A
FAM50B	Family With Sequence Similarity 50 Member B	Protein Coding	Q9Y247	36	GC06P004005	4.378137	https://www.genecards.org/cgi-bin/carddisp.pl?gene=FAM50B
CXCR4	C-X-C Motif Chemokine Receptor 4	Protein Coding	P61073	56	GC02M136114	4.375343	https://www.genecards.org/cgi-bin/carddisp.pl?gene=CXCR4
MIR30C1	MicroRNA 30c-1	RNA Gene		22	GC01P040757	4.372322	https://www.genecards.org/cgi-bin/carddisp.pl?gene=MIR30C1
DHFR	Dihydrofolate Reductase	Protein Coding	P00374	52	GC05M080626	4.370556	https://www.genecards.org/cgi-bin/carddisp.pl?gene=DHFR
PLG	Plasminogen	Protein Coding	P00747	53	GC06P160702	4.370301	https://www.genecards.org/cgi-bin/carddisp.pl?gene=PLG

LAMB3	Laminin Subunit Beta 3	Protein Coding	Q13751	50	GC01M209614	4.369102	https://www.genecards.org/cgi-bin/carddisp.pl?gene=LAMB3
SI	Sucrase-Isomaltase	Protein Coding	P14410	48	GC03M164978	4.367133	https://www.genecards.org/cgi-bin/carddisp.pl?gene=SI
MIR199B	MicroRNA 199b	RNA Gene		20	GC09M128244	4.366276	https://www.genecards.org/cgi-bin/carddisp.pl?gene=MIR199B
KISS1R	KISS1 Receptor	Protein Coding	Q969F8	51	GC19P000917	4.365171	https://www.genecards.org/cgi-bin/carddisp.pl?gene=KISS1R
WWOX	WW Domain Containing Oxidoreductase	Protein Coding	Q9NZC7	50	GC16P078099	4.36464	https://www.genecards.org/cgi-bin/carddisp.pl?gene=WWOX
GFAP	Glial Fibrillary Acidic Protein	Protein Coding	P14136	52	GC17M064302	4.359559	https://www.genecards.org/cgi-bin/carddisp.pl?gene=GFAP
PTH1R	Parathyroid Hormone 1 Receptor	Protein Coding	Q03431	54	GC03P046877	4.351954	https://www.genecards.org/cgi-bin/carddisp.pl?gene=PTH1R
MIR182	MicroRNA 182	RNA Gene		22	GC07M129770	4.346872	https://www.genecards.org/cgi-bin/carddisp.pl?gene=MIR182
LOC114803475	PPARG EExon Liver Enhancer	Functional Element		3	GC03P012405	4.345178	https://www.genecards.org/cgi-bin/carddisp.pl?gene=LOC114803475
SNORD107	Small Nucleolar RNA, C/D Box 107	RNA Gene		14	GC15P056594	4.343353	https://www.genecards.org/cgi-bin/carddisp.pl?gene=SNORD107
MIR100	MicroRNA 100	RNA Gene		22	GC11M122152	4.34054	https://www.genecards.org/cgi-bin/carddisp.pl?gene=MIR100
CSN1S1	Casein Alpha S1	Protein Coding	P47710	40	GC04P069963	4.338363	https://www.genecards.org/cgi-bin/carddisp.pl?gene=CSN1S1

ERCC6	ERCC Excision Repair 6, Chromatin Remodeling Factor	Protein Coding	Q03468	50	GC10M049454	4.336006	https://www.genecards.org/cgi-bin/carddisp.pl?gene=ERCC6
ADSL	Adenylosuccinate Lyase	Protein Coding	P30566	51	GC22P040346	4.331806	https://www.genecards.org/cgi-bin/carddisp.pl?gene=ADSL
MIR31	MicroRNA 31	RNA Gene		21	GC09M022007	4.327641	https://www.genecards.org/cgi-bin/carddisp.pl?gene=MIR31
MIR9-1	MicroRNA 9-1	RNA Gene		21	GC01M156420	4.314013	https://www.genecards.org/cgi-bin/carddisp.pl?gene=MIR9-1
MPO	Myeloperoxidase	Protein Coding	P05164	57	GC17M058269	4.313744	https://www.genecards.org/cgi-bin/carddisp.pl?gene=MPO
NQO1	NAD(P)H Quinone Dehydrogenase 1	Protein Coding	P15559	52	GC16M069706	4.311739	https://www.genecards.org/cgi-bin/carddisp.pl?gene=NQO1
NR1I3	Nuclear Receptor Subfamily 1 Group I Member 3	Protein Coding	Q14994	48	GC01M161229	4.301867	https://www.genecards.org/cgi-bin/carddisp.pl?gene=NR1I3
PPP1R3A	Protein Phosphatase 1 Regulatory Subunit 3A	Protein Coding	Q16821	46	GC07M113876	4.300763	https://www.genecards.org/cgi-bin/carddisp.pl?gene=PPP1R3A
SLC30A2	Solute Carrier Family 30 Member 2	Protein Coding	Q9BRI3	45	GC01M029701	4.2975	https://www.genecards.org/cgi-bin/carddisp.pl?gene=SLC30A2
PAPPA2	Pappalysin 2	Protein Coding	Q9BXP8	44	GC01P176463	4.293844	https://www.genecards.org/cgi-bin/carddisp.pl?gene=PAPPA2
TIMP3	TIMP Metallopeptidase Inhibitor 3	Protein Coding	P35625	47	GC22P056519	4.29217	https://www.genecards.org/cgi-bin/carddisp.pl?gene=TIMP3
SNORD64	Small Nucleolar RNA, C/D Box 64	RNA Gene		16	GC15P056590	4.291391	https://www.genecards.org/cgi-bin/carddisp.pl?gene=SNORD64

ACADVL	Acyl-CoA Dehydrogenase Very Long Chain	Protein Coding	P49748	50	GC17P013984	4.283237	https://www.genecards.org/cgi-bin/carddisp.pl?gene=ACADVL
ZNF445	Zinc Finger Protein 445	Protein Coding	P59923	39	GC03M044443	4.274405	https://www.genecards.org/cgi-bin/carddisp.pl?gene=ZNF445
WNT4	Wnt Family Member 4	Protein Coding	P56705	52	GC01M022190	4.264064	https://www.genecards.org/cgi-bin/carddisp.pl?gene=WNT4
AKT3	AKT Serine/Threonine Kinase 3	Protein Coding	Q9Y243	58	GC01M243488	4.258487	https://www.genecards.org/cgi-bin/carddisp.pl?gene=AKT3
CASC2	Cancer Susceptibility 2	RNA Gene	Q8IU53	27	GC10P118046	4.256877	https://www.genecards.org/cgi-bin/carddisp.pl?gene=CASC2
CDKN2B-AS1	CDKN2B Antisense RNA 1	RNA Gene		25	GC09P021994	4.255612	https://www.genecards.org/cgi-bin/carddisp.pl?gene=CDKN2B-AS1
TEK	TEK Receptor Tyrosine Kinase	Protein Coding	Q02763	55	GC09P027109	4.248353	https://www.genecards.org/cgi-bin/carddisp.pl?gene=TEK
USH2A	Usherin	Protein Coding	O75445	42	GC01M215622	4.242731	https://www.genecards.org/cgi-bin/carddisp.pl?gene=USH2A
CEL	Carboxyl Ester Lipase	Protein Coding	P19835	50	GC09P133061	4.241645	https://www.genecards.org/cgi-bin/carddisp.pl?gene=CEL
ATM	ATM Serine/Threonine Kinase	Protein Coding	Q13315	57	GC11P108222	4.237419	https://www.genecards.org/cgi-bin/carddisp.pl?gene=ATM
MIR181A2	MicroRNA 181a-2	RNA Gene		21	GC09P124692	4.231216	https://www.genecards.org/cgi-bin/carddisp.pl?gene=MIR181A2
MIR203A	MicroRNA 203a	RNA Gene		22	GC14P112953	4.230114	https://www.genecards.org/cgi-bin/carddisp.pl?gene=MIR203A

MMP8	Matrix Metallopeptidase 8	Protein Coding	P22894	52	GC11M114278	4.2214	https://www.genecards.org/cgi-bin/carddisp.pl?gene=MMP8
TGFBR2	Transforming Growth Factor Beta Receptor 2	Protein Coding	P37173	56	GC03P030623	4.220346	https://www.genecards.org/cgi-bin/carddisp.pl?gene=TGFBR2
SLC5A1	Solute Carrier Family 5 Member 1	Protein Coding	P13866	51	GC22P032043	4.220036	https://www.genecards.org/cgi-bin/carddisp.pl?gene=SLC5A1
SLC7A5	Solute Carrier Family 7 Member 5	Protein Coding	Q01650	48	GC16M087830	4.214041	https://www.genecards.org/cgi-bin/carddisp.pl?gene=SLC7A5
SNORD109B	Small Nucleolar RNA, C/D Box 109B	RNA Gene		15	GC15P056617	4.213103	https://www.genecards.org/cgi-bin/carddisp.pl?gene=SNORD109B
ALDH5A1	Aldehyde Dehydrogenase 5 Family Member A1	Protein Coding	P51649	51	GC06P024494	4.212349	https://www.genecards.org/cgi-bin/carddisp.pl?gene=ALDH5A1
ATP5PO	ATP Synthase Peripheral Stalk Subunit OSCP	Protein Coding	P48047	47	GC21M033904	4.211021	https://www.genecards.org/cgi-bin/carddisp.pl?gene=ATP5PO
DYRK1A	Dual Specificity Tyrosine Phosphorylation Regulated Kinase 1A	Protein Coding	Q13627	54	GC21P037365	4.20133	https://www.genecards.org/cgi-bin/carddisp.pl?gene=DYRK1A
CFB	Complement Factor B	Protein Coding	P00751	51	GC06P031945	4.193879	https://www.genecards.org/cgi-bin/carddisp.pl?gene=CFB
NAT1	N-Acetyltransferase 1	Protein Coding	P18440	48	GC08P018183	4.191219	https://www.genecards.org/cgi-bin/carddisp.pl?gene=NAT1
BCDIN3D	BCDIN3 Domain Containing RNA Methyltransferase	Protein Coding	Q7Z5W3	40	GC12M050620	4.185371	https://www.genecards.org/cgi-bin/carddisp.pl?gene=BCDIN3D
GLI3	GLI Family Zinc Finger 3	Protein Coding	P10071	53	GC07M041960	4.18511	https://www.genecards.org/cgi-bin/carddisp.pl?gene=GLI3

FCER1A	Fc Epsilon Receptor Ia	Protein Coding	P12319	48	GC01P159259	4.181356	https://www.genecards.org/cgi-bin/carddisp.pl?gene=FCER1A
MIR183	MicroRNA 183	RNA Gene		20	GC07M129866	4.179866	https://www.genecards.org/cgi-bin/carddisp.pl?gene=MIR183
CLU	Clusterin	Protein Coding	P10909	51	GC08M027596	4.175991	https://www.genecards.org/cgi-bin/carddisp.pl?gene=CLU
HGF	Hepatocyte Growth Factor	Protein Coding	P14210	55	GC07M081699	4.17553	https://www.genecards.org/cgi-bin/carddisp.pl?gene=HGF
AIF1	Allograft Inflammatory Factor 1	Protein Coding	P55008	43	GC06P111995	4.172383	https://www.genecards.org/cgi-bin/carddisp.pl?gene=AIF1
HBA1	Hemoglobin Subunit Alpha 1	Protein Coding	P69905	46	GC16P052349	4.168638	https://www.genecards.org/cgi-bin/carddisp.pl?gene=HBA1
PDP1	Pyruvate Dehydrogenase Phosphatase Catalytic Subunit 1	Protein Coding	Q9P0J1	51	GC08P093857	4.168387	https://www.genecards.org/cgi-bin/carddisp.pl?gene=PDP1
CFL1	Cofilin 1	Protein Coding	P23528	50	GC11M065823	4.165497	https://www.genecards.org/cgi-bin/carddisp.pl?gene=CFL1
MIR206	MicroRNA 206	RNA Gene		21	GC06P052144	4.165051	https://www.genecards.org/cgi-bin/carddisp.pl?gene=MIR206
APOL1	Apolipoprotein L1	Protein Coding	O14791	48	GC22P036253	4.162313	https://www.genecards.org/cgi-bin/carddisp.pl?gene=APOL1
PPIG	Peptidylprolyl Isomerase G	Protein Coding	Q13427	46	GC02P169584	4.155969	https://www.genecards.org/cgi-bin/carddisp.pl?gene=PPIG
PTHLH	Parathyroid Hormone Like Hormone	Protein Coding	P12272	50	GC12M027959	4.154829	https://www.genecards.org/cgi-bin/carddisp.pl?gene=PTHLH

NGF	Nerve Growth Factor	Protein Coding	P01138	55	GC01M115285	4.147801	https://www.genecards.org/cgi-bin/carddisp.pl?gene=NGF
SZT2	SZT2 Subunit Of KICSTOR Complex	Protein Coding	Q5T011	41	GC01P043389	4.146574	https://www.genecards.org/cgi-bin/carddisp.pl?gene=SZT2
IDH1	Isocitrate Dehydrogenase (NADP(+)) 1	Protein Coding	O75874	57	GC02M208236	4.142429	https://www.genecards.org/cgi-bin/carddisp.pl?gene=IDH1
SHC1	SHC Adaptor Protein 1	Protein Coding	P29353	48	GC01M154962	4.136755	https://www.genecards.org/cgi-bin/carddisp.pl?gene=SHC1
CCND1	Cyclin D1	Protein Coding	P24385	57	GC11P069641	4.12926	https://www.genecards.org/cgi-bin/carddisp.pl?gene=CCND1
TSC2	TSC Complex Subunit 2	Protein Coding	P49815	54	GC16P052432	4.128916	https://www.genecards.org/cgi-bin/carddisp.pl?gene=TSC2
PDGFRA	Platelet Derived Growth Factor Receptor Alpha	Protein Coding	P16234	57	GC04P054229	4.127537	https://www.genecards.org/cgi-bin/carddisp.pl?gene=PDGFRA
ABCB4	ATP Binding Cassette Subfamily B Member 4	Protein Coding	P21439	51	GC07M087365	4.123222	https://www.genecards.org/cgi-bin/carddisp.pl?gene=ABCB4
TLR1	Toll Like Receptor 1	Protein Coding	Q15399	52	GC04M038793	4.120149	https://www.genecards.org/cgi-bin/carddisp.pl?gene=TLR1
PIK3CG	Phosphatidylinositol-4,5-Bisphosphate 3-Kinase Catalytic Subunit Gamma	Protein Coding	P48736	53	GC07P106865	4.119744	https://www.genecards.org/cgi-bin/carddisp.pl?gene=PIK3CG
SPARC	Secreted Protein Acidic And Cysteine Rich	Protein Coding	P09486	53	GC05M151661	4.118906	https://www.genecards.org/cgi-bin/carddisp.pl?gene=SPARC
KLF11	KLF Transcription Factor 11	Protein Coding	O14901	41	GC02P010050	4.115761	https://www.genecards.org/cgi-bin/carddisp.pl?gene=KLF11

NDUFS2	NADH:Ubiquinone Oxidoreductase Core Subunit S2	Protein Coding	O75306	47	GC01P161197	4.115415	https://www.genecards.org/cgi-bin/carddisp.pl?gene=NDUFS2
LIPF	Lipase F, Gastric Type	Protein Coding	P07098	46	GC10P088664	4.114628	https://www.genecards.org/cgi-bin/carddisp.pl?gene=LIPF
LOC124902337	Uncharacterized LOC124902337	Pseudogene		14	GC09P126427	4.11315	https://www.genecards.org/cgi-bin/carddisp.pl?gene=LOC124902337
TLR3	Toll Like Receptor 3	Protein Coding	O15455	55	GC04P186059	4.11261	https://www.genecards.org/cgi-bin/carddisp.pl?gene=TLR3
ACHE	Acetylcholinesterase (Cartwright Blood Group)	Protein Coding	P22303	50	GC07M100889	4.108793	https://www.genecards.org/cgi-bin/carddisp.pl?gene=ACHE
AQP9	Aquaporin 9	Protein Coding	O43315	46	GC15P058138	4.104352	https://www.genecards.org/cgi-bin/carddisp.pl?gene=AQP9
BCYRN1	Brain Cytoplasmic RNA 1	RNA Gene		20	GC02P047253	4.095553	https://www.genecards.org/cgi-bin/carddisp.pl?gene=BCYRN1
KDM5C	Lysine Demethylase 5C	Protein Coding	P41229	51	GC0XM053176	4.094784	https://www.genecards.org/cgi-bin/carddisp.pl?gene=KDM5C
OGG1	8-Oxoguanine DNA Glycosylase	Protein Coding	O15527	51	GC03P018314	4.094779	https://www.genecards.org/cgi-bin/carddisp.pl?gene=OGG1
THOC2	THO Complex Subunit 2	Protein Coding	Q8NI27	45	GC0XM123600	4.093653	https://www.genecards.org/cgi-bin/carddisp.pl?gene=THOC2
MIR211	MicroRNA 211	RNA Gene		22	GC15M031065	4.092962	https://www.genecards.org/cgi-bin/carddisp.pl?gene=MIR211
CDK4	Cyclin Dependent Kinase 4	Protein Coding	P11802	58	GC12M058829	4.092077	https://www.genecards.org/cgi-bin/carddisp.pl?gene=CDK4

MIR185	MicroRNA 185	RNA Gene		23	GC22P056021	4.086402	https://www.genecards.org/cgi-bin/carddisp.pl?gene=MIR185
HLA-E	Major Histocompatibility Complex, Class I, E	Protein Coding	P13747	45	GC06P111966	4.080733	https://www.genecards.org/cgi-bin/carddisp.pl?gene=HLA-E
CSF3	Colony Stimulating Factor 3	Protein Coding	P09919	45	GC17P040015	4.079096	https://www.genecards.org/cgi-bin/carddisp.pl?gene=CSF3
CD109	CD109 Molecule	Protein Coding	Q6YHK3	44	GC06P112560	4.060648	https://www.genecards.org/cgi-bin/carddisp.pl?gene=CD109
EIF2S1	Eukaryotic Translation Initiation Factor 2 Subunit Alpha	Protein Coding	P05198	48	GC14P067359	4.05953	https://www.genecards.org/cgi-bin/carddisp.pl?gene=EIF2S1
THADA	THADA Armadillo Repeat Containing	Protein Coding	Q6YHU6	42	GC02M043233	4.055455	https://www.genecards.org/cgi-bin/carddisp.pl?gene=THADA
PDCD1	Programmed Cell Death 1	Protein Coding	Q15116	52	GC02M241849	4.053653	https://www.genecards.org/cgi-bin/carddisp.pl?gene=PDCD1
FAH	Fumarylacetoacetate Hydrolase	Protein Coding	P16930	51	GC15P080152	4.052756	https://www.genecards.org/cgi-bin/carddisp.pl?gene=FAH
TXNRD2	Thioredoxin Reductase 2	Protein Coding	Q9NNW7	49	GC22M019863	4.048542	https://www.genecards.org/cgi-bin/carddisp.pl?gene=TXNRD2
TPH1	Tryptophan Hydroxylase 1	Protein Coding	P17752	49	GC11M018040	4.035815	https://www.genecards.org/cgi-bin/carddisp.pl?gene=TPH1
GAST	Gastrin	Protein Coding	P01350	44	GC17P041712	4.03185	https://www.genecards.org/cgi-bin/carddisp.pl?gene=GAST
BAMBI	BMP And Activin Membrane Bound Inhibitor	Protein Coding	Q13145	47	GC10P028705	4.031558	https://www.genecards.org/cgi-bin/carddisp.pl?gene=BAMBI

ATP1A2	ATPase Na+/K+ Transporting Subunit Alpha 2	Protein Coding	P50993	52	GC01P160115	4.031436	https://www.genecards.org/cgi-bin/carddisp.pl?gene=ATP1A2
MIRLET7F1	MicroRNA Let-7f-1	RNA Gene		19	GC09P096124	4.022602	https://www.genecards.org/cgi-bin/carddisp.pl?gene=MIRLET7F1
CTSD	Cathepsin D	Protein Coding	P07339	57	GC11M001752	4.022343	https://www.genecards.org/cgi-bin/carddisp.pl?gene=CTSD
PWRN3	Prader-Willi Region Non-Protein Coding RNA 3	RNA Gene		15	GC15P059931	4.021729	https://www.genecards.org/cgi-bin/carddisp.pl?gene=PWRN3
FOXM1	Forkhead Box M1	Protein Coding	Q08050	48	GC12M002857	4.003428	https://www.genecards.org/cgi-bin/carddisp.pl?gene=FOXM1
FABP5	Fatty Acid Binding Protein 5	Protein Coding	Q01469	46	GC08P081282	3.998647	https://www.genecards.org/cgi-bin/carddisp.pl?gene=FABP5
FGF1	Fibroblast Growth Factor 1	Protein Coding	P05230	51	GC05M142555	3.997042	https://www.genecards.org/cgi-bin/carddisp.pl?gene=FGF1
PLAC1	Placenta Enriched 1	Protein Coding	Q9HBJ0	39	GC0XM134565	3.992609	https://www.genecards.org/cgi-bin/carddisp.pl?gene=PLAC1
GRIN2B	Glutamate Ionotropic Receptor NMDA Type Subunit 2B	Protein Coding	Q13224	56	GC12M013437	3.989089	https://www.genecards.org/cgi-bin/carddisp.pl?gene=GRIN2B
F8	Coagulation Factor VIII	Protein Coding	P00451	51	GC0XM154835	3.985748	https://www.genecards.org/cgi-bin/carddisp.pl?gene=F8
ITGA2	Integrin Subunit Alpha 2	Protein Coding	P17301	50	GC05P052989	3.984615	https://www.genecards.org/cgi-bin/carddisp.pl?gene=ITGA2
CFAP418-AS1	CFAP418 Antisense RNA 1	RNA Gene		16	GC08P095205	3.978673	https://www.genecards.org/cgi-bin/carddisp.pl?gene=CFAP418-AS1

HPGD	15-Hydroxyprostaglandin Dehydrogenase	Protein Coding	P15428	51	GC04M174490	3.978642	https://www.genecards.org/cgi-bin/carddisp.pl?gene=HPGD
HSPA1B	Heat Shock Protein Family A (Hsp70) Member 1B	Protein Coding	P0DMV9	43	GC06P112018	3.972207	https://www.genecards.org/cgi-bin/carddisp.pl?gene=HSPA1B
PVALB	Parvalbumin	Protein Coding	P20472	45	GC22M036800	3.963935	https://www.genecards.org/cgi-bin/carddisp.pl?gene=PVALB
MIR16-1	MicroRNA 16-1	RNA Gene		21	GC13M050048	3.963472	https://www.genecards.org/cgi-bin/carddisp.pl?gene=MIR16-1
IQSEC2	IQ Motif And Sec7 Domain ArfGEF 2	Protein Coding	Q5JU85	43	GC0XM053225	3.961516	https://www.genecards.org/cgi-bin/carddisp.pl?gene=IQSEC2
MIR526B	MicroRNA 526b	RNA Gene		19	GC19P053694	3.957114	https://www.genecards.org/cgi-bin/carddisp.pl?gene=MIR526B
MAPK8IP1	Mitogen-Activated Protein Kinase 8 Interacting Protein 1	Protein Coding	Q9UQF2	48	GC11P047010	3.950731	https://www.genecards.org/cgi-bin/carddisp.pl?gene=MAPK8IP1
MT-TG	Mitochondrially Encoded TRNA-Gly (GGN)	RNA Gene		13	GCMTP009993	3.950493	https://www.genecards.org/cgi-bin/carddisp.pl?gene=MT-TG
SERPINF1	Serpin Family F Member 1	Protein Coding	P36955	50	GC17P013679	3.949336	https://www.genecards.org/cgi-bin/carddisp.pl?gene=SERPINF1
TET2	Tet Methylcytosine Dioxygenase 2	Protein Coding	Q6N021	50	GC04P105145	3.945611	https://www.genecards.org/cgi-bin/carddisp.pl?gene=TET2
VTRNA1-1	Vault RNA 1-1	RNA Gene		15	GC05P149542	3.945595	https://www.genecards.org/cgi-bin/carddisp.pl?gene=VTRNA1-1
BAP1	BRCA1 Associated Protein 1	Protein Coding	Q92560	51	GC03M052401	3.944411	https://www.genecards.org/cgi-bin/carddisp.pl?gene=BAP1

ZEB2	Zinc Finger E-Box Binding Homeobox 2	Protein Coding	O60315	51	GC02M144384	3.943755	https://www.genecards.org/cgi-bin/carddisp.pl?gene=ZEB2
LRP5	LDL Receptor Related Protein 5	Protein Coding	O75197	53	GC11P068298	3.941998	https://www.genecards.org/cgi-bin/carddisp.pl?gene=LRP5
MIRLET7E	MicroRNA Let-7e	RNA Gene		21	GC19P090040	3.93861	https://www.genecards.org/cgi-bin/carddisp.pl?gene=MIRLET7E
DCC	DCC Netrin 1 Receptor	Protein Coding	P43146	51	GC18P052340	3.931195	https://www.genecards.org/cgi-bin/carddisp.pl?gene=DCC
BLK	BLK Proto-Oncogene, Src Family Tyrosine Kinase	Protein Coding	P51451	53	GC08P011486	3.925978	https://www.genecards.org/cgi-bin/carddisp.pl?gene=BLK
CYBA	Cytochrome B-245 Alpha Chain	Protein Coding	P13498	51	GC16M088643	3.925188	https://www.genecards.org/cgi-bin/carddisp.pl?gene=CYBA
MIR15A	MicroRNA 15a	RNA Gene		17	GC13M050049	3.925022	https://www.genecards.org/cgi-bin/carddisp.pl?gene=MIR15A
LIMK1	LIM Domain Kinase 1	Protein Coding	P53667	52	GC07P074082	3.920677	https://www.genecards.org/cgi-bin/carddisp.pl?gene=LIMK1
IGFBP6	Insulin Like Growth Factor Binding Protein 6	Protein Coding	P24592	47	GC12P053097	3.915082	https://www.genecards.org/cgi-bin/carddisp.pl?gene=IGFBP6
INS-IGF2	INS-IGF2 Readthrough	Protein Coding	F8WCM5	30	GC11M009010	3.913613	https://www.genecards.org/cgi-bin/carddisp.pl?gene=INS-IGF2
UGT1A1	UDP Glucuronosyltransferase Family 1 Member A1	Protein Coding	P22309	54	GC02P233760	3.91249	https://www.genecards.org/cgi-bin/carddisp.pl?gene=UGT1A1
KIR2DL4	Killer Cell Immunoglobulin Like Receptor, Two Ig Domains And Long Cytoplasmic Tail 4	Protein Coding	Q99706	42	GC19P090184	3.909317	https://www.genecards.org/cgi-bin/carddisp.pl?gene=KIR2DL4

GP1BB	Glycoprotein Ib Platelet Subunit Beta	Protein Coding	P13224	47	GC22P056007	3.907806	https://www.genecards.org/cgi-bin/carddisp.pl?gene=GP1BB
MIR485	MicroRNA 485	RNA Gene		19	GC14P113607	3.906553	https://www.genecards.org/cgi-bin/carddisp.pl?gene=MIR485
PC	Pyruvate Carboxylase	Protein Coding	P11498	51	GC11M066848	3.906334	https://www.genecards.org/cgi-bin/carddisp.pl?gene=PC
IVNS1ABP	Influenza Virus NS1A Binding Protein	Protein Coding	Q9Y6Y0	46	GC01M185307	3.905595	https://www.genecards.org/cgi-bin/carddisp.pl?gene=IVNS1ABP
PEG10	Paternally Expressed 10	Protein Coding	Q86TG7	44	GC07P094656	3.904305	https://www.genecards.org/cgi-bin/carddisp.pl?gene=PEG10
RELA	RELA Proto-Oncogene, NF-KB Subunit	Protein Coding	Q04206	55	GC11M065653	3.899341	https://www.genecards.org/cgi-bin/carddisp.pl?gene=RELA
GPX3	Glutathione Peroxidase 3	Protein Coding	P22352	45	GC05P150997	3.894113	https://www.genecards.org/cgi-bin/carddisp.pl?gene=GPX3
EGF	Epidermal Growth Factor	Protein Coding	P01133	55	GC04P109912	3.893981	https://www.genecards.org/cgi-bin/carddisp.pl?gene=EGF
ISL1	ISL LIM Homeobox 1	Protein Coding	P61371	48	GC05P051383	3.893893	https://www.genecards.org/cgi-bin/carddisp.pl?gene=ISL1
SRY	Sex Determining Region Y	Protein Coding	Q05066	39	GC0YM002698	3.890227	https://www.genecards.org/cgi-bin/carddisp.pl?gene=SRY
MAF	MAF BZIP Transcription Factor	Protein Coding	O75444	49	GC16M079204	3.887938	https://www.genecards.org/cgi-bin/carddisp.pl?gene=MAF
P2RX5-TAX1BP3	P2RX5-TAX1BP3 Readthrough (NMD Candidate)	RNA Gene		15	GC17M014192	3.884363	https://www.genecards.org/cgi-bin/carddisp.pl?gene=P2RX5-TAX1BP3

AMBP	Alpha-1-Microglobulin/Bikunin Precursor	Protein Coding	P02760	48	GC09M114060	3.88392	https://www.genecards.org/cgi-bin/carddisp.pl?gene=AMBP
KIF4A	Kinesin Family Member 4A	Protein Coding	O95239	44	GC0XP070290	3.88221	https://www.genecards.org/cgi-bin/carddisp.pl?gene=KIF4A
TBX5	T-Box Transcription Factor 5	Protein Coding	Q99593	50	GC12M114353	3.871619	https://www.genecards.org/cgi-bin/carddisp.pl?gene=TBX5
AIP	Aryl Hydrocarbon Receptor Interacting Protein	Protein Coding	O00170	47	GC11P067468	3.866271	https://www.genecards.org/cgi-bin/carddisp.pl?gene=AIP
NEUROG3	Neurogenin 3	Protein Coding	Q9Y4Z2	44	GC10M069571	3.862379	https://www.genecards.org/cgi-bin/carddisp.pl?gene=NEUROG3
COQ2	Coenzyme Q2, Polyprenyltransferase	Protein Coding	Q96H96	45	GC04M083261	3.859807	https://www.genecards.org/cgi-bin/carddisp.pl?gene=COQ2
HSPG2	Heparan Sulfate Proteoglycan 2	Protein Coding	P98160	52	GC01M021822	3.857761	https://www.genecards.org/cgi-bin/carddisp.pl?gene=HSPG2
PON2	Paraoxonase 2	Protein Coding	Q15165	47	GC07M095404	3.857625	https://www.genecards.org/cgi-bin/carddisp.pl?gene=PON2
APOC2	Apolipoprotein C2	Protein Coding	P02655	48	GC19P089743	3.852738	https://www.genecards.org/cgi-bin/carddisp.pl?gene=APOC2
HDAC9	Histone Deacetylase 9	Protein Coding	Q9UKV0	52	GC07P018086	3.852083	https://www.genecards.org/cgi-bin/carddisp.pl?gene=HDAC9
POU1F1	POU Class 1 Homeobox 1	Protein Coding	P28069	45	GC03M087259	3.843184	https://www.genecards.org/cgi-bin/carddisp.pl?gene=POU1F1
IL33	Interleukin 33	Protein Coding	O95760	43	GC09P008424	3.827582	https://www.genecards.org/cgi-bin/carddisp.pl?gene=IL33

MT-TQ	Mitochondrially Encoded TRNA-Gln (CAA/G)	RNA Gene		14	GCMTM0043 31	3.82626 6	https://www.genecards.org/cgi-bin/carddisp.pl?gene=MT-TQ
CDK1	Cyclin Dependent Kinase 1	Protein Coding	P06493	52	GC10P060772	3.82521	https://www.genecards.org/cgi-bin/carddisp.pl?gene=CDK1
KL	Klotho	Protein Coding	Q9UEF7	52	GC13P033016	3.81594	https://www.genecards.org/cgi-bin/carddisp.pl?gene=KL
TP73	Tumor Protein P73	Protein Coding	O15350	50	GC01P003652	3.81498 1	https://www.genecards.org/cgi-bin/carddisp.pl?gene=TP73
SNORD44	Small Nucleolar RNA, C/D Box 44	RNA Gene		14	GC01M17480 2	3.81482 7	https://www.genecards.org/cgi-bin/carddisp.pl?gene=SNORD44
ARG1	Arginase 1	Protein Coding	P05089	53	GC06P131473	3.81478 9	https://www.genecards.org/cgi-bin/carddisp.pl?gene=ARG1
FGF23	Fibroblast Growth Factor 23	Protein Coding	Q9GZV9	50	GC12M00436 8	3.80873 3	https://www.genecards.org/cgi-bin/carddisp.pl?gene=FGF23
SFRP5	Secreted Frizzled Related Protein 5	Protein Coding	Q5T4F7	42	GC10M09776 6	3.80349 5	https://www.genecards.org/cgi-bin/carddisp.pl?gene=SFRP5
FGF19	Fibroblast Growth Factor 19	Protein Coding	O95750	47	GC11M11373 9	3.80284 5	https://www.genecards.org/cgi-bin/carddisp.pl?gene=FGF19
MIR16-2	MicroRNA 16-2	RNA Gene		20	GC03P160418	3.80076 5	https://www.genecards.org/cgi-bin/carddisp.pl?gene=MIR16-2
MIR338	MicroRNA 338	RNA Gene		19	GC17M08124 2	3.80072 8	https://www.genecards.org/cgi-bin/carddisp.pl?gene=MIR338
SLC5A2	Solute Carrier Family 5 Member 2	Protein Coding	P31639	51	GC16P054260	3.79795 7	https://www.genecards.org/cgi-bin/carddisp.pl?gene=SLC5A2

MIR675	MicroRNA 675	RNA Gene		20	GC11M009003	3.796404	https://www.genecards.org/cgi-bin/carddisp.pl?gene=MIR675
PEE1	Preeclampsia/Eclampsia 1	Genetic Locus		3	GC04U902284	3.793608	https://www.genecards.org/cgi-bin/carddisp.pl?gene=PEE1
MIR10A	MicroRNA 10a	RNA Gene		22	GC17M048579	3.79204	https://www.genecards.org/cgi-bin/carddisp.pl?gene=MIR10A
SNORD108	Small Nucleolar RNA, C/D Box 108	RNA Gene		16	GC15P056591	3.789625	https://www.genecards.org/cgi-bin/carddisp.pl?gene=SNORD108
MIR181B1	MicroRNA 181b-1	RNA Gene		21	GC01M198858	3.789401	https://www.genecards.org/cgi-bin/carddisp.pl?gene=MIR181B1
FOXP2	Forkhead Box P2	Protein Coding	O15409	48	GC07P114086	3.785665	https://www.genecards.org/cgi-bin/carddisp.pl?gene=FOXP2
GPX1	Glutathione Peroxidase 1	Protein Coding	P07203	49	GC03M052674	3.784441	https://www.genecards.org/cgi-bin/carddisp.pl?gene=GPX1
LGALS9	Galectin 9	Protein Coding	O00182	44	GC17P027629	3.780874	https://www.genecards.org/cgi-bin/carddisp.pl?gene=LGALS9
MMP13	Matrix Metallopeptidase 13	Protein Coding	P45452	54	GC11M102942	3.780837	https://www.genecards.org/cgi-bin/carddisp.pl?gene=MMP13
LOC109623489	Insulin Repeat Instability Region	Functional Element		3	GC11P004527	3.777595	https://www.genecards.org/cgi-bin/carddisp.pl?gene=LOC109623489
HSP90AA1	Heat Shock Protein 90 Alpha Family Class A Member 1	Protein Coding	P07900	55	GC14M102080	3.775304	https://www.genecards.org/cgi-bin/carddisp.pl?gene=HSP90AA1
NLRP2	NLR Family Pyrin Domain Containing 2	Protein Coding	Q9NX02	46	GC19P054953	3.767467	https://www.genecards.org/cgi-bin/carddisp.pl?gene=NLRP2

ENSG00000286110	Novel Transcript	RNA Gene		8	GC15P024558	3.761185	https://www.genecards.org/cgi-bin/carddisp.pl?gene=ENSG00000286110
TSLP	Thymic Stromal Lymphopoietin	Protein Coding	Q969D9	43	GC05P111070	3.758798	https://www.genecards.org/cgi-bin/carddisp.pl?gene=TSLP
PRF1	Perforin 1	Protein Coding	P14222	50	GC10M070597	3.7569	https://www.genecards.org/cgi-bin/carddisp.pl?gene=PRF1
CCAT1	Colon Cancer Associated Transcript 1	RNA Gene		16	GC08M127207	3.755843	https://www.genecards.org/cgi-bin/carddisp.pl?gene=CCAT1
CHAT	Choline O-Acetyltransferase	Protein Coding	P28329	52	GC10P049609	3.754253	https://www.genecards.org/cgi-bin/carddisp.pl?gene=CHAT
SNORD109A	Small Nucleolar RNA, C/D Box 109A	RNA Gene		15	GC15P025041	3.752614	https://www.genecards.org/cgi-bin/carddisp.pl?gene=SNORD109A
IL4R	Interleukin 4 Receptor	Protein Coding	P24394	52	GC16P053264	3.751842	https://www.genecards.org/cgi-bin/carddisp.pl?gene=IL4R
RNA18SN1	RNA, 18S Ribosomal N1	RNA Gene		8	GC21P015820	3.751129	https://www.genecards.org/cgi-bin/carddisp.pl?gene=RNA18SN1
MIR330	MicroRNA 330	RNA Gene		21	GC19M082720	3.747858	https://www.genecards.org/cgi-bin/carddisp.pl?gene=MIR330
DDB1	Damage Specific DNA Binding Protein 1	Protein Coding	Q16531	50	GC11M113342	3.74025	https://www.genecards.org/cgi-bin/carddisp.pl?gene=DDB1
SIRT5	Sirtuin 5	Protein Coding	Q9NXA8	49	GC06P013574	3.736587	https://www.genecards.org/cgi-bin/carddisp.pl?gene=SIRT5
VHL	Von Hippel-Lindau Tumor Suppressor	Protein Coding	P40337	52	GC03P018360	3.735083	https://www.genecards.org/cgi-bin/carddisp.pl?gene=VHL

MMP7	Matrix Metallopeptidase 7	Protein Coding	P09237	51	GC11M114277	3.734896	https://www.genecards.org/cgi-bin/carddisp.pl?gene=MMP7
UCN3	Urocortin 3	Protein Coding	Q969E3	40	GC10P005364	3.726199	https://www.genecards.org/cgi-bin/carddisp.pl?gene=UCN3
PAFAH1B1	Platelet Activating Factor Acetylhydrolase 1b Regulatory Subunit 1	Protein Coding	P43034	51	GC17P002593	3.725832	https://www.genecards.org/cgi-bin/carddisp.pl?gene=PAFAH1B1
CRB1	Crumbs Cell Polarity Complex Component 1	Protein Coding	P82279	48	GC01P197208	3.719563	https://www.genecards.org/cgi-bin/carddisp.pl?gene=CRB1
CYP11B2	Cytochrome P450 Family 11 Subfamily B Member 2	Protein Coding	P19099	51	GC08M142910	3.718772	https://www.genecards.org/cgi-bin/carddisp.pl?gene=CYP11B2
MMP14	Matrix Metallopeptidase 14	Protein Coding	P50281	55	GC14P040147	3.717109	https://www.genecards.org/cgi-bin/carddisp.pl?gene=MMP14
MIR215	MicroRNA 215	RNA Gene		20	GC01M220117	3.715009	https://www.genecards.org/cgi-bin/carddisp.pl?gene=MIR215
MSBP1	Minisatellite Binding Protein 1	Protein Coding		5	GC00U990213	3.714291	https://www.genecards.org/cgi-bin/carddisp.pl?gene=MSBP1
DMD	Dystrophin	Protein Coding	P11532	49	GC0XM031097	3.708718	https://www.genecards.org/cgi-bin/carddisp.pl?gene=DMD
AZGP1	Alpha-2-Glycoprotein 1, Zinc-Binding	Protein Coding	P25311	47	GC07M099967	3.706636	https://www.genecards.org/cgi-bin/carddisp.pl?gene=AZGP1
ALOX5	Arachidonate 5-Lipoxygenase	Protein Coding	P09917	52	GC10P045374	3.704746	https://www.genecards.org/cgi-bin/carddisp.pl?gene=ALOX5
NPHS1	NPHS1 Adhesion Molecule, Nephrin	Protein Coding	O60500	51	GC19M035825	3.695198	https://www.genecards.org/cgi-bin/carddisp.pl?gene=NPHS1

ASAH1	N-Acylsphingosine Amidohydrolase 1	Protein Coding	Q13510	53	GC08M018055	3.687876	https://www.genecards.org/cgi-bin/carddisp.pl?gene=ASAH1
LRP2	LDL Receptor Related Protein 2	Protein Coding	P98164	52	GC02M169127	3.686147	https://www.genecards.org/cgi-bin/carddisp.pl?gene=LRP2
IRF6	Interferon Regulatory Factor 6	Protein Coding	O14896	47	GC01M209785	3.68326	https://www.genecards.org/cgi-bin/carddisp.pl?gene=IRF6
MIR149	MicroRNA 149	RNA Gene		23	GC02P240456	3.679538	https://www.genecards.org/cgi-bin/carddisp.pl?gene=MIR149
HULC	Hepatocellular Carcinoma Up-Regulated Long Non-Coding RNA	RNA Gene		22	GC06P008457	3.677971	https://www.genecards.org/cgi-bin/carddisp.pl?gene=HULC
CHRNA7	Cholinergic Receptor Nicotinic Alpha 7 Subunit	Protein Coding	P36544	48	GC15P031923	3.677	https://www.genecards.org/cgi-bin/carddisp.pl?gene=CHRNA7
RPS27A	Ribosomal Protein S27a	Protein Coding	P62979	47	GC02P055231	3.674498	https://www.genecards.org/cgi-bin/carddisp.pl?gene=RPS27A
MIR125B2	MicroRNA 125b-2	RNA Gene		22	GC21P016590	3.672157	https://www.genecards.org/cgi-bin/carddisp.pl?gene=MIR125B2
DNMT3L	DNA Methyltransferase 3 Like	Protein Coding	Q9UJW3	45	GC21M044246	3.671952	https://www.genecards.org/cgi-bin/carddisp.pl?gene=DNMT3L
GBE1	1,4-Alpha-Glucan Branching Enzyme 1	Protein Coding	Q04446	48	GC03M081489	3.666597	https://www.genecards.org/cgi-bin/carddisp.pl?gene=GBE1
SCN5A	Sodium Voltage-Gated Channel Alpha Subunit 5	Protein Coding	Q14524	54	GC03M038605	3.662187	https://www.genecards.org/cgi-bin/carddisp.pl?gene=SCN5A
PEMT	Phosphatidylethanolamine N-Methyltransferase	Protein Coding	Q9UBM1	43	GC17M017653	3.65309	https://www.genecards.org/cgi-bin/carddisp.pl?gene=PEMT

FLT1P1	FLT1 Pseudogene 1	Pseudogene		9	GC03M046142	3.652639	https://www.genecards.org/cgi-bin/carddisp.pl?gene=FLT1P1
GJA5	Gap Junction Protein Alpha 5	Protein Coding	P36382	48	GC01M147756	3.641056	https://www.genecards.org/cgi-bin/carddisp.pl?gene=GJA5
MBL2	Mannose Binding Lectin 2	Protein Coding	P11226	50	GC10M052760	3.639552	https://www.genecards.org/cgi-bin/carddisp.pl?gene=MBL2
PLAUR	Plasminogen Activator, Urokinase Receptor	Protein Coding	Q03405	49	GC19M043646	3.638134	https://www.genecards.org/cgi-bin/carddisp.pl?gene=PLAUR
IER3IP1	Immediate Early Response 3 Interacting Protein 1	Protein Coding	Q9Y5U9	39	GC18M047152	3.636906	https://www.genecards.org/cgi-bin/carddisp.pl?gene=IER3IP1
SIGLEC6	Sialic Acid Binding Ig Like Lectin 6	Protein Coding	O43699	45	GC19M051517	3.636835	https://www.genecards.org/cgi-bin/carddisp.pl?gene=SIGLEC6
DDX6	DEAD-Box Helicase 6	Protein Coding	P26196	49	GC11M118748	3.628976	https://www.genecards.org/cgi-bin/carddisp.pl?gene=DDX6
BSND	Barttin CLCNK Type Accessory Subunit Beta	Protein Coding	Q8WZ55	44	GC01P054998	3.623671	https://www.genecards.org/cgi-bin/carddisp.pl?gene=BSND
CHI3L1	Chitinase 3 Like 1	Protein Coding	P36222	48	GC01M203148	3.622859	https://www.genecards.org/cgi-bin/carddisp.pl?gene=CHI3L1
SYNE1	Spectrin Repeat Containing Nuclear Envelope Protein 1	Protein Coding	Q8NF91	44	GC06M152121	3.62178	https://www.genecards.org/cgi-bin/carddisp.pl?gene=SYNE1
TRD-GTC9-1	TRNA-Asp (Anticodon GTC) 9-1	RNA Gene		8	GC01P162078	3.619923	https://www.genecards.org/cgi-bin/carddisp.pl?gene=TRD-GTC9-1
ENHO	Energy Homeostasis Associated	Protein Coding	Q6UWT2	35	GC09M034627	3.619222	https://www.genecards.org/cgi-bin/carddisp.pl?gene=ENHO

MIR518B	MicroRNA 518b	RNA Gene		18	GC19P090142	3.614551	https://www.genecards.org/cgi-bin/carddisp.pl?gene=MIR518B
ATP6AP2	ATPase H+ Transporting Accessory Protein 2	Protein Coding	O75787	49	GC0XP040593	3.612411	https://www.genecards.org/cgi-bin/carddisp.pl?gene=ATP6AP2
NCF1	Neutrophil Cytosolic Factor 1	Protein Coding	P14598	51	GC07P081244	3.612102	https://www.genecards.org/cgi-bin/carddisp.pl?gene=NCF1
MIR216A	MicroRNA 216a	RNA Gene		21	GC02M055988	3.610303	https://www.genecards.org/cgi-bin/carddisp.pl?gene=MIR216A
DHCR7	7-Dehydrocholesterol Reductase	Protein Coding	Q9UBM7	51	GC11M071428	3.606601	https://www.genecards.org/cgi-bin/carddisp.pl?gene=DHCR7
RBPJ	Recombination Signal Binding Protein For Immunoglobulin Kappa J Region	Protein Coding	Q06330	51	GC04P026105	3.603954	https://www.genecards.org/cgi-bin/carddisp.pl?gene=RBPJ
CD79A	CD79a Molecule	Protein Coding	P11912	52	GC19P041877	3.599805	https://www.genecards.org/cgi-bin/carddisp.pl?gene=CD79A
IL2RB	Interleukin 2 Receptor Subunit Beta	Protein Coding	P14784	54	GC22M070405	3.598975	https://www.genecards.org/cgi-bin/carddisp.pl?gene=IL2RB
EZH2	Enhancer Of Zeste 2 Polycomb Repressive Complex 2 Subunit	Protein Coding	Q15910	57	GC07M148807	3.597215	https://www.genecards.org/cgi-bin/carddisp.pl?gene=EZH2
IL1RAP	Interleukin 1 Receptor Accessory Protein	Protein Coding	Q9NPH3	50	GC03P190514	3.594562	https://www.genecards.org/cgi-bin/carddisp.pl?gene=IL1RAP
MIR379	MicroRNA 379	RNA Gene		18	GC14P113598	3.594095	https://www.genecards.org/cgi-bin/carddisp.pl?gene=MIR379
SLC22A5	Solute Carrier Family 22 Member 5	Protein Coding	O76082	51	GC05P132369	3.593328	https://www.genecards.org/cgi-bin/carddisp.pl?gene=SLC22A5

GNRHR	Gonadotropin Releasing Hormone Receptor	Protein Coding	P30968	51	GC04M067737	3.590358	https://www.genecards.org/cgi-bin/carddisp.pl?gene=GNRHR
TLR9	Toll Like Receptor 9	Protein Coding	Q9NR96	49	GC03M053009	3.590246	https://www.genecards.org/cgi-bin/carddisp.pl?gene=TLR9
MIR26A1	MicroRNA 26a-1	RNA Gene		22	GC03P037969	3.589419	https://www.genecards.org/cgi-bin/carddisp.pl?gene=MIR26A1
ALPL	Alkaline Phosphatase, Biomineralization Associated	Protein Coding	P05186	55	GC01P021508	3.584877	https://www.genecards.org/cgi-bin/carddisp.pl?gene=ALPL
THPO	Thrombopoietin	Protein Coding	P40225	47	GC03M184371	3.582194	https://www.genecards.org/cgi-bin/carddisp.pl?gene=THPO
ANGPTL3	Angiopoietin Like 3	Protein Coding	Q9Y5C1	51	GC01P062597	3.572357	https://www.genecards.org/cgi-bin/carddisp.pl?gene=ANGPTL3
TACR3	Tachykinin Receptor 3	Protein Coding	P29371	53	GC04M103586	3.567791	https://www.genecards.org/cgi-bin/carddisp.pl?gene=TACR3
IGFBP4	Insulin Like Growth Factor Binding Protein 4	Protein Coding	P22692	47	GC17P040443	3.56526	https://www.genecards.org/cgi-bin/carddisp.pl?gene=IGFBP4
VEGFC	Vascular Endothelial Growth Factor C	Protein Coding	P49767	51	GC04M176683	3.561661	https://www.genecards.org/cgi-bin/carddisp.pl?gene=VEGFC
PCNA	Proliferating Cell Nuclear Antigen	Protein Coding	P12004	54	GC20M005114	3.559426	https://www.genecards.org/cgi-bin/carddisp.pl?gene=PCNA
ASCC1	Activating Signal Cointegrator 1 Complex Subunit 1	Protein Coding	Q8N9N2	46	GC10M072096	3.549222	https://www.genecards.org/cgi-bin/carddisp.pl?gene=ASCC1
YWHAEP7	Tyrosine 3-Monooxygenase/Tryptophan 5-Monooxygenase Activation Protein Epsilon Pseudogene 7	Pseudogene		13	GC17M063871	3.54892	https://www.genecards.org/cgi-bin/carddisp.pl?gene=YWHAEP7

CEACAMP8	CEA Cell Adhesion Molecule Pseudogene 8	Pseudogene		9	GC19M043035	3.54892	https://www.genecards.org/cgi-bin/carddisp.pl?gene=CEACAMP8
IRX2-DT	IRX2 Divergent Transcript	RNA Gene	Q86SI9	30	GC05P002751	3.54872	https://www.genecards.org/cgi-bin/carddisp.pl?gene=IRX2-DT
MIR374A	MicroRNA 374a	RNA Gene		18	GC0XM074300	3.543438	https://www.genecards.org/cgi-bin/carddisp.pl?gene=MIR374A
MEF2A	Myocyte Enhancer Factor 2A	Protein Coding	Q02078	50	GC15P099565	3.539259	https://www.genecards.org/cgi-bin/carddisp.pl?gene=MEF2A
FAS-AS1	FAS Antisense RNA 1	RNA Gene		15	GC10M088991	3.536866	https://www.genecards.org/cgi-bin/carddisp.pl?gene=FAS-AS1
MIR200B	MicroRNA 200b	RNA Gene		22	GC01P001167	3.535926	https://www.genecards.org/cgi-bin/carddisp.pl?gene=MIR200B
CXCR3	C-X-C Motif Chemokine Receptor 3	Protein Coding	P49682	48	GC0XM071615	3.528055	https://www.genecards.org/cgi-bin/carddisp.pl?gene=CXCR3
MYOD1	Myogenic Differentiation 1	Protein Coding	P15172	50	GC11P017719	3.523809	https://www.genecards.org/cgi-bin/carddisp.pl?gene=MYOD1
MIR195	MicroRNA 195	RNA Gene		20	GC17M014367	3.520353	https://www.genecards.org/cgi-bin/carddisp.pl?gene=MIR195
S100B	S100 Calcium Binding Protein B	Protein Coding	P04271	48	GC21M052648	3.519651	https://www.genecards.org/cgi-bin/carddisp.pl?gene=S100B
IL10RA	Interleukin 10 Receptor Subunit Alpha	Protein Coding	Q13651	50	GC11P117987	3.514867	https://www.genecards.org/cgi-bin/carddisp.pl?gene=IL10RA
FOLR1	Folate Receptor Alpha	Protein Coding	P15328	52	GC11P080179	3.514131	https://www.genecards.org/cgi-bin/carddisp.pl?gene=FOLR1

SERPINA1	Serpin Family A Member 1	Protein Coding	P01009	53	GC14M094376	3.50974	https://www.genecards.org/cgi-bin/carddisp.pl?gene=SERPINA1
TRO	Trophinin	Protein Coding	Q12816	41	GC0XP054920	3.508331	https://www.genecards.org/cgi-bin/carddisp.pl?gene=TRO
TGFB2	Transforming Growth Factor Beta 2	Protein Coding	P61812	55	GC01P218345	3.506652	https://www.genecards.org/cgi-bin/carddisp.pl?gene=TGFB2
WRN	WRN RecQ Like Helicase	Protein Coding	Q14191	51	GC08P031033	3.493136	https://www.genecards.org/cgi-bin/carddisp.pl?gene=WRN
MIR302A	MicroRNA 302a	RNA Gene		21	GC04M112869	3.491953	https://www.genecards.org/cgi-bin/carddisp.pl?gene=MIR302A
FH	Fumarate Hydratase	Protein Coding	P07954	51	GC01M241499	3.490158	https://www.genecards.org/cgi-bin/carddisp.pl?gene=FH
GLI1	GLI Family Zinc Finger 1	Protein Coding	P08151	52	GC12P058687	3.489031	https://www.genecards.org/cgi-bin/carddisp.pl?gene=GLI1
LCT	Lactase	Protein Coding	P09848	47	GC02M135787	3.481397	https://www.genecards.org/cgi-bin/carddisp.pl?gene=LCT
PEG3	Paternally Expressed 3	Protein Coding	Q9GZU2	42	GC19M056810	3.480553	https://www.genecards.org/cgi-bin/carddisp.pl?gene=PEG3
NR1H3	Nuclear Receptor Subfamily 1 Group H Member 3	Protein Coding	Q13133	50	GC11P047248	3.480058	https://www.genecards.org/cgi-bin/carddisp.pl?gene=NR1H3
MIR132	MicroRNA 132	RNA Gene		22	GC17M002049	3.47826	https://www.genecards.org/cgi-bin/carddisp.pl?gene=MIR132
STS	Steroid Sulfatase	Protein Coding	P08842	50	GC0XP007146	3.475762	https://www.genecards.org/cgi-bin/carddisp.pl?gene=STS

NOTCH1	Notch Receptor 1	Protein Coding	P46531	55	GC09M138322	3.47489	https://www.genecards.org/cgi-bin/carddisp.pl?gene=NOTCH1
MSX1	Msh Homeobox 1	Protein Coding	P28360	48	GC04P004861	3.473568	https://www.genecards.org/cgi-bin/carddisp.pl?gene=MSX1
CDKN1A	Cyclin Dependent Kinase Inhibitor 1A	Protein Coding	P38936	53	GC06P112162	3.473223	https://www.genecards.org/cgi-bin/carddisp.pl?gene=CDKN1A
SULT1A1	Sulfotransferase Family 1A Member 1	Protein Coding	P50225	47	GC16M041444	3.472927	https://www.genecards.org/cgi-bin/carddisp.pl?gene=SULT1A1
FGR	FGR Proto-Oncogene, Src Family Tyrosine Kinase	Protein Coding	P09769	51	GC01M029805	3.466319	https://www.genecards.org/cgi-bin/carddisp.pl?gene=FGR
DKK1	Dickkopf WNT Signaling Pathway Inhibitor 1	Protein Coding	O94907	48	GC10P052314	3.461462	https://www.genecards.org/cgi-bin/carddisp.pl?gene=DKK1
GRN	Granulin Precursor	Protein Coding	P28799	52	GC17P044345	3.459856	https://www.genecards.org/cgi-bin/carddisp.pl?gene=GRN
LOC124902571	Uncharacterized LOC124902571	Pseudogene		11	GC10P029577	3.459493	https://www.genecards.org/cgi-bin/carddisp.pl?gene=LOC124902571
CYP24A1	Cytochrome P450 Family 24 Subfamily A Member 1	Protein Coding	Q07973	51	GC20M054153	3.453791	https://www.genecards.org/cgi-bin/carddisp.pl?gene=CYP24A1
ABCG1	ATP Binding Cassette Subfamily G Member 1	Protein Coding	P45844	47	GC21P042199	3.452179	https://www.genecards.org/cgi-bin/carddisp.pl?gene=ABCG1
VIP	Vasoactive Intestinal Peptide	Protein Coding	P01282	48	GC06P152750	3.451773	https://www.genecards.org/cgi-bin/carddisp.pl?gene=VIP
LBR	Lamin B Receptor	Protein Coding	Q14739	51	GC01M225401	3.451294	https://www.genecards.org/cgi-bin/carddisp.pl?gene=LBR

CUBN	Cubilin	Protein Coding	O60494	49	GC10M016824	3.448756	https://www.genecards.org/cgi-bin/carddisp.pl?gene=CUBN
YAP1	Yes1 Associated Transcriptional Regulator	Protein Coding	P46937	52	GC11P102110	3.448175	https://www.genecards.org/cgi-bin/carddisp.pl?gene=YAP1
LCOR	Ligand Dependent Nuclear Receptor Corepressor	Protein Coding	Q96JN0	42	GC10P096832	3.444993	https://www.genecards.org/cgi-bin/carddisp.pl?gene=LCOR
RARB	Retinoic Acid Receptor Beta	Protein Coding	P10826	53	GC03P024689	3.444194	https://www.genecards.org/cgi-bin/carddisp.pl?gene=RARB
SOX9	SRY-Box Transcription Factor 9	Protein Coding	P48436	50	GC17P072121	3.44274	https://www.genecards.org/cgi-bin/carddisp.pl?gene=SOX9
CCR1	C-C Motif Chemokine Receptor 1	Protein Coding	P32246	47	GC03M046218	3.442467	https://www.genecards.org/cgi-bin/carddisp.pl?gene=CCR1
CYP1B1	Cytochrome P450 Family 1 Subfamily B Member 1	Protein Coding	Q16678	52	GC02M038066	3.442047	https://www.genecards.org/cgi-bin/carddisp.pl?gene=CYP1B1
GTF2I	General Transcription Factor IIi	Protein Coding	P78347	44	GC07P081239	3.439634	https://www.genecards.org/cgi-bin/carddisp.pl?gene=GTF2I
MIR205	MicroRNA 205	RNA Gene		21	GC01P209432	3.438334	https://www.genecards.org/cgi-bin/carddisp.pl?gene=MIR205
KMT2A	Lysine Methyltransferase 2A	Protein Coding	Q03164	52	GC11P118436	3.436625	https://www.genecards.org/cgi-bin/carddisp.pl?gene=KMT2A
TBX19	T-Box Transcription Factor 19	Protein Coding	O60806	44	GC01P168280	3.43565	https://www.genecards.org/cgi-bin/carddisp.pl?gene=TBX19
AQP2	Aquaporin 2	Protein Coding	P41181	51	GC12P049950	3.433149	https://www.genecards.org/cgi-bin/carddisp.pl?gene=AQP2

MIR224	MicroRNA 224	RNA Gene		17	GC0XM151958	3.429747	https://www.genecards.org/cgi-bin/carddisp.pl?gene=MIR224
CCN1	Cellular Communication Network Factor 1	Protein Coding	O00622	45	GC01P085585	3.429637	https://www.genecards.org/cgi-bin/carddisp.pl?gene=CCN1
PTGER2	Prostaglandin E Receptor 2	Protein Coding	P43116	52	GC14P052314	3.429325	https://www.genecards.org/cgi-bin/carddisp.pl?gene=PTGER2
ZNF346	Zinc Finger Protein 346	Protein Coding	Q9UL40	40	GC05P177022	3.425421	https://www.genecards.org/cgi-bin/carddisp.pl?gene=ZNF346
ITGA2B	Integrin Subunit Alpha 2b	Protein Coding	P08514	55	GC17M064280	3.423805	https://www.genecards.org/cgi-bin/carddisp.pl?gene=ITGA2B
JAZF1	JAZF Zinc Finger 1	Protein Coding	Q86VZ6	42	GC07M027830	3.421879	https://www.genecards.org/cgi-bin/carddisp.pl?gene=JAZF1
LINC00261	Long Intergenic Non-Protein Coding RNA 261	RNA Gene		22	GC20M022529	3.42181	https://www.genecards.org/cgi-bin/carddisp.pl?gene=LINC00261
ANGPTL6	Angiopoietin Like 6	Protein Coding	Q8NI99	43	GC19M010092	3.421644	https://www.genecards.org/cgi-bin/carddisp.pl?gene=ANGPTL6
MKI67	Marker Of Proliferation Ki-67	Protein Coding	P46013	48	GC10M128096	3.420328	https://www.genecards.org/cgi-bin/carddisp.pl?gene=MKI67
SMARCB1	SWI/SNF Related, Matrix Associated, Actin Dependent Regulator Of Chromatin, Subfamily B, Member 1	Protein Coding	Q12824	51	GC22P023786	3.41883	https://www.genecards.org/cgi-bin/carddisp.pl?gene=SMARCB1
MIR433	MicroRNA 433	RNA Gene		22	GC14P112879	3.415495	https://www.genecards.org/cgi-bin/carddisp.pl?gene=MIR433
KDM6A	Lysine Demethylase 6A	Protein Coding	O15550	52	GC0XP044873	3.406994	https://www.genecards.org/cgi-bin/carddisp.pl?gene=KDM6A

CD40	CD40 Molecule	Protein Coding	P25942	54	GC20P046118	3.406502	https://www.genecards.org/cgi-bin/carddisp.pl?gene=CD40
COQ4	Coenzyme Q4	Protein Coding	Q9Y3A0	44	GC09P128322	3.401021	https://www.genecards.org/cgi-bin/carddisp.pl?gene=COQ4
L1CAM	L1 Cell Adhesion Molecule	Protein Coding	P32004	52	GC0XM153864	3.399628	https://www.genecards.org/cgi-bin/carddisp.pl?gene=L1CAM
RBFOX2	RNA Binding Fox-1 Homolog 2	Protein Coding	O43251	44	GC22M035738	3.394335	https://www.genecards.org/cgi-bin/carddisp.pl?gene=RBFOX2
DLL1	Delta Like Canonical Notch Ligand 1	Protein Coding	O00548	49	GC06M170282	3.3904	https://www.genecards.org/cgi-bin/carddisp.pl?gene=DLL1
IL6ST	Interleukin 6 Cytokine Family Signal Transducer	Protein Coding	P40189	54	GC05M055935	3.386654	https://www.genecards.org/cgi-bin/carddisp.pl?gene=IL6ST
EARS2	Glutamyl-TRNA Synthetase 2, Mitochondrial	Protein Coding	Q5JPH6	46	GC16M024534	3.383221	https://www.genecards.org/cgi-bin/carddisp.pl?gene=EARS2
TBX15	T-Box Transcription Factor 15	Protein Coding	Q96SF7	44	GC01M118883	3.379505	https://www.genecards.org/cgi-bin/carddisp.pl?gene=TBX15
MIR130A	MicroRNA 130a	RNA Gene		22	GC11P057641	3.372058	https://www.genecards.org/cgi-bin/carddisp.pl?gene=MIR130A
SCG2	Secretogranin II	Protein Coding	P13521	43	GC02M223596	3.371237	https://www.genecards.org/cgi-bin/carddisp.pl?gene=SCG2
PIK3CB	Phosphatidylinositol-4,5-Bisphosphate 3-Kinase Catalytic Subunit Beta	Protein Coding	P42338	52	GC03M138652	3.369865	https://www.genecards.org/cgi-bin/carddisp.pl?gene=PIK3CB
ITGB1	Integrin Subunit Beta 1	Protein Coding	P05556	54	GC10M034762	3.369523	https://www.genecards.org/cgi-bin/carddisp.pl?gene=ITGB1

ATP2B3	ATPase Plasma Membrane Ca2+ Transporting 3	Protein Coding	Q16720	50	GC0XP153517	3.369122	https://www.genecards.org/cgi-bin/carddisp.pl?gene=ATP2B3
DANCR	Differentiation Antagonizing Non-Protein Coding RNA	RNA Gene	P0C864	24	GC04P052712	3.366054	https://www.genecards.org/cgi-bin/carddisp.pl?gene=DANCR
MMP12	Matrix Metallopeptidase 12	Protein Coding	P39900	50	GC11M102862	3.362798	https://www.genecards.org/cgi-bin/carddisp.pl?gene=MMP12
GSR	Glutathione-Disulfide Reductase	Protein Coding	P00390	53	GC08M030678	3.359752	https://www.genecards.org/cgi-bin/carddisp.pl?gene=GSR
COL5A1	Collagen Type V Alpha 1 Chain	Protein Coding	P20908	51	GC09P134641	3.358178	https://www.genecards.org/cgi-bin/carddisp.pl?gene=COL5A1
CNTNAP2	Contactin Associated Protein 2	Protein Coding	Q9UHC6	50	GC07P146116	3.357764	https://www.genecards.org/cgi-bin/carddisp.pl?gene=CNTNAP2
HNF1A-AS1	HNF1A Antisense RNA 1	RNA Gene		21	GC12M122956	3.355696	https://www.genecards.org/cgi-bin/carddisp.pl?gene=HNF1A-AS1
MIR410	MicroRNA 410	RNA Gene		18	GC14P113604	3.351888	https://www.genecards.org/cgi-bin/carddisp.pl?gene=MIR410
MIR96	MicroRNA 96	RNA Gene		21	GC07M129774	3.345083	https://www.genecards.org/cgi-bin/carddisp.pl?gene=MIR96
CX3CR1	C-X3-C Motif Chemokine Receptor 1	Protein Coding	P49238	47	GC03M039279	3.343152	https://www.genecards.org/cgi-bin/carddisp.pl?gene=CX3CR1
BHMT2	Betaine--Homocysteine S-Methyltransferase 2	Protein Coding	Q9H2M3	45	GC05P079071	3.339047	https://www.genecards.org/cgi-bin/carddisp.pl?gene=BHMT2
ENSG00000232995	Regulator Of G Protein Signaling 5	Uncategorized		8	GC01M163214	3.338382	https://www.genecards.org/cgi-bin/carddisp.pl?gene=ENSG00000232995

PCK2	Phosphoenolpyruvate Carboxykinase 2, Mitochondrial	Protein Coding	Q16822	50	GC14P024094	3.337592	https://www.genecards.org/cgi-bin/carddisp.pl?gene=PCK2
SOD3	Superoxide Dismutase 3	Protein Coding	P08294	47	GC04P024798	3.333981	https://www.genecards.org/cgi-bin/carddisp.pl?gene=SOD3
KCNJ5	Potassium Inwardly Rectifying Channel Subfamily J Member 5	Protein Coding	P48544	51	GC11P128891	3.333438	https://www.genecards.org/cgi-bin/carddisp.pl?gene=KCNJ5
SOX11	SRY-Box Transcription Factor 11	Protein Coding	P35716	45	GC02P005707	3.332493	https://www.genecards.org/cgi-bin/carddisp.pl?gene=SOX11
DBH	Dopamine Beta-Hydroxylase	Protein Coding	P09172	55	GC09P133636	3.331475	https://www.genecards.org/cgi-bin/carddisp.pl?gene=DBH
EPHX2	Epoxide Hydrolase 2	Protein Coding	P34913	51	GC08P027490	3.32773	https://www.genecards.org/cgi-bin/carddisp.pl?gene=EPHX2
ADA	Adenosine Deaminase	Protein Coding	P00813	55	GC20M044620	3.325074	https://www.genecards.org/cgi-bin/carddisp.pl?gene=ADA
MIR28	MicroRNA 28	RNA Gene		20	GC03P188688	3.324885	https://www.genecards.org/cgi-bin/carddisp.pl?gene=MIR28
FLT3	Fms Related Receptor Tyrosine Kinase 3	Protein Coding	P36888	57	GC13M028003	3.321445	https://www.genecards.org/cgi-bin/carddisp.pl?gene=FLT3
GSK3B	Glycogen Synthase Kinase 3 Beta	Protein Coding	P49841	54	GC03M119821	3.316949	https://www.genecards.org/cgi-bin/carddisp.pl?gene=GSK3B
HOXA11-AS	HOXA11 Antisense RNA	RNA Gene		22	GC07P027184	3.315672	https://www.genecards.org/cgi-bin/carddisp.pl?gene=HOXA11-AS
CYP2C19	Cytochrome P450 Family 2 Subfamily C Member 19	Protein Coding	P33261	48	GC10P094762	3.312253	https://www.genecards.org/cgi-bin/carddisp.pl?gene=CYP2C19

MIR196A2	MicroRNA 196a-2	RNA Gene		23	GC12P058520	3.305949	https://www.genecards.org/cgi-bin/carddisp.pl?gene=MIR196A2
IL7	Interleukin 7	Protein Coding	P13232	48	GC08M078689	3.304387	https://www.genecards.org/cgi-bin/carddisp.pl?gene=IL7
LRRC56	Leucine Rich Repeat Containing 56	Protein Coding	Q8IYG6	39	GC11P004388	3.302514	https://www.genecards.org/cgi-bin/carddisp.pl?gene=LRRC56
LBP	Lipopolysaccharide Binding Protein	Protein Coding	P18428	47	GC20P038346	3.302459	https://www.genecards.org/cgi-bin/carddisp.pl?gene=LBP
PROP1	PROP Paired-Like Homeobox 1	Protein Coding	O75360	44	GC05M177992	3.299444	https://www.genecards.org/cgi-bin/carddisp.pl?gene=PROP1
TCF20	Transcription Factor 20	Protein Coding	Q9UGU0	42	GC22M042160	3.298199	https://www.genecards.org/cgi-bin/carddisp.pl?gene=TCF20
SOX2-OT	SOX2 Overlapping Transcript	RNA Gene		27	GC03P180989	3.29708	https://www.genecards.org/cgi-bin/carddisp.pl?gene=SOX2-OT
IL3	Interleukin 3	Protein Coding	P08700	49	GC05P132060	3.287596	https://www.genecards.org/cgi-bin/carddisp.pl?gene=IL3
SCNN1A	Sodium Channel Epithelial 1 Subunit Alpha	Protein Coding	P37088	53	GC12M006346	3.281563	https://www.genecards.org/cgi-bin/carddisp.pl?gene=SCNN1A
CRB2	Crumbs Cell Polarity Complex Component 2	Protein Coding	Q5IJ48	46	GC09P123356	3.281365	https://www.genecards.org/cgi-bin/carddisp.pl?gene=CRB2
TGFBR1	Transforming Growth Factor Beta Receptor 1	Protein Coding	P36897	58	GC09P099104	3.280301	https://www.genecards.org/cgi-bin/carddisp.pl?gene=TGFBR1
TSHB	Thyroid Stimulating Hormone Subunit Beta	Protein Coding	P01222	46	GC01P115029	3.279526	https://www.genecards.org/cgi-bin/carddisp.pl?gene=TSHB

LHCGR	Luteinizing Hormone/Choriogonadotropin Receptor	Protein Coding	P22888	51	GC02M048686	3.276577	https://www.genecards.org/cgi-bin/carddisp.pl?gene=LHCGR
MIR34C	MicroRNA 34c	RNA Gene		22	GC11P112199	3.276438	https://www.genecards.org/cgi-bin/carddisp.pl?gene=MIR34C
ERVW-1	Endogenous Retrovirus Group W Member 1, Envelope	Protein Coding	Q9UQF0	39	GC07M092468	3.273677	https://www.genecards.org/cgi-bin/carddisp.pl?gene=ERVW-1
PDGFB	Platelet Derived Growth Factor Subunit B	Protein Coding	P01127	55	GC22M071264	3.269273	https://www.genecards.org/cgi-bin/carddisp.pl?gene=PDGFB
EMX2	Empty Spiracles Homeobox 2	Protein Coding	Q04743	48	GC10P117542	3.26643	https://www.genecards.org/cgi-bin/carddisp.pl?gene=EMX2
SLC7A7	Solute Carrier Family 7 Member 7	Protein Coding	Q9UM01	50	GC14M022773	3.264526	https://www.genecards.org/cgi-bin/carddisp.pl?gene=SLC7A7
TCF4	Transcription Factor 4	Protein Coding	P15884	51	GC18M055222	3.262751	https://www.genecards.org/cgi-bin/carddisp.pl?gene=TCF4
PTPRC	Protein Tyrosine Phosphatase Receptor Type C	Protein Coding	P08575	56	GC01P198607	3.261838	https://www.genecards.org/cgi-bin/carddisp.pl?gene=PTPRC
SLC2A9	Solute Carrier Family 2 Member 9	Protein Coding	Q9NRM0	50	GC04M009772	3.257817	https://www.genecards.org/cgi-bin/carddisp.pl?gene=SLC2A9
SCG5	Secretogranin V	Protein Coding	P05408	42	GC15P032641	3.257514	https://www.genecards.org/cgi-bin/carddisp.pl?gene=SCG5
ADAM17	ADAM Metallopeptidase Domain 17	Protein Coding	P78536	54	GC02M009488	3.247606	https://www.genecards.org/cgi-bin/carddisp.pl?gene=ADAM17
MIR15B	MicroRNA 15b	RNA Gene		21	GC03P160404	3.247243	https://www.genecards.org/cgi-bin/carddisp.pl?gene=MIR15B

SCARNA5	Small Cajal Body-Specific RNA 5	RNA Gene		21	GC02P233275	3.244245	https://www.genecards.org/cgi-bin/carddisp.pl?gene=SCARNA5
NIPA1	NIPA Magnesium Transporter 1	Protein Coding	Q7RTP0	42	GC15P022773	3.240514	https://www.genecards.org/cgi-bin/carddisp.pl?gene=NIPA1
CES1	Carboxylesterase 1	Protein Coding	P23141	52	GC16M055836	3.236387	https://www.genecards.org/cgi-bin/carddisp.pl?gene=CES1
CYFIP1	Cytoplasmic FMR1 Interacting Protein 1	Protein Coding	Q7L576	44	GC15M022867	3.233381	https://www.genecards.org/cgi-bin/carddisp.pl?gene=CYFIP1
DRD1	Dopamine Receptor D1	Protein Coding	P21728	48	GC05M175440	3.232468	https://www.genecards.org/cgi-bin/carddisp.pl?gene=DRD1
MIR134	MicroRNA 134	RNA Gene		20	GC14P113582	3.232455	https://www.genecards.org/cgi-bin/carddisp.pl?gene=MIR134
NIPA2	NIPA Magnesium Transporter 2	Protein Coding	Q8N8Q9	41	GC15P056573	3.232318	https://www.genecards.org/cgi-bin/carddisp.pl?gene=NIPA2
PWAR5	Prader Willi/Angelman Region RNA 5	RNA Gene		16	GC15P056595	3.230255	https://www.genecards.org/cgi-bin/carddisp.pl?gene=PWAR5
SETBP1	SET Binding Protein 1	Protein Coding	Q9Y6X0	45	GC18P044680	3.228734	https://www.genecards.org/cgi-bin/carddisp.pl?gene=SETBP1
NPHS2	NPHS2 Stomatin Family Member, Podocin	Protein Coding	Q9NP85	46	GC01M179554	3.226671	https://www.genecards.org/cgi-bin/carddisp.pl?gene=NPHS2
COX5A	Cytochrome C Oxidase Subunit 5A	Protein Coding	P20674	48	GC15M074919	3.226159	https://www.genecards.org/cgi-bin/carddisp.pl?gene=COX5A
FSTL3	Follistatin Like 3	Protein Coding	O95633	44	GC19P000676	3.225005	https://www.genecards.org/cgi-bin/carddisp.pl?gene=FSTL3

PIK3C2A	Phosphatidylinositol-4-Phosphate 3-Kinase Catalytic Subunit Type 2 Alpha	Protein Coding	O00443	51	GC11M017815	3.224824	https://www.genecards.org/cgi-bin/carddisp.pl?gene=PIK3C2A
ERF	ETS2 Repressor Factor	Protein Coding	P50548	45	GC19M042247	3.212418	https://www.genecards.org/cgi-bin/carddisp.pl?gene=ERF
ADD1	Adducin 1	Protein Coding	P35611	48	GC04P002859	3.209708	https://www.genecards.org/cgi-bin/carddisp.pl?gene=ADD1
UQCRC2	Ubiquinol-Cytochrome C Reductase Core Protein 2	Protein Coding	P22695	50	GC16P053124	3.20908	https://www.genecards.org/cgi-bin/carddisp.pl?gene=UQCRC2
KLHL7	Kelch Like Family Member 7	Protein Coding	Q8IXQ5	45	GC07P023105	3.208352	https://www.genecards.org/cgi-bin/carddisp.pl?gene=KLHL7
FOXE1	Forkhead Box E1	Protein Coding	O00358	45	GC09P097853	3.204819	https://www.genecards.org/cgi-bin/carddisp.pl?gene=FOXE1
MAPK14	Mitogen-Activated Protein Kinase 14	Protein Coding	Q16539	54	GC06P112153	3.204346	https://www.genecards.org/cgi-bin/carddisp.pl?gene=MAPK14
PRLR	Prolactin Receptor	Protein Coding	P16471	53	GC05M035048	3.201301	https://www.genecards.org/cgi-bin/carddisp.pl?gene=PRLR
APOM	Apolipoprotein M	Protein Coding	O95445	45	GC06P112006	3.200334	https://www.genecards.org/cgi-bin/carddisp.pl?gene=APOM
GLUL	Glutamate-Ammonia Ligase	Protein Coding	P15104	52	GC01M182378	3.199464	https://www.genecards.org/cgi-bin/carddisp.pl?gene=GLUL
MIR191	MicroRNA 191	RNA Gene		22	GC03M052653	3.199205	https://www.genecards.org/cgi-bin/carddisp.pl?gene=MIR191
PKHD1	PKHD1 Ciliary IPT Domain Containing Fibrocystin/Polyductin	Protein Coding	P08F94	42	GC06M084330	3.197019	https://www.genecards.org/cgi-bin/carddisp.pl?gene=PKHD1

JAK3	Janus Kinase 3	Protein Coding	P52333	55	GC19M017824	3.191094	https://www.genecards.org/cgi-bin/carddisp.pl?gene=JAK3
SCD5	Stearoyl-CoA Desaturase 5	Protein Coding	Q86SK9	45	GC04M082629	3.189385	https://www.genecards.org/cgi-bin/carddisp.pl?gene=SCD5
EBF1	EBF Transcription Factor 1	Protein Coding	Q9UH73	45	GC05M158695	3.188185	https://www.genecards.org/cgi-bin/carddisp.pl?gene=EBF1
SPRY2	Sprouty RTK Signaling Antagonist 2	Protein Coding	O43597	51	GC13M080335	3.186918	https://www.genecards.org/cgi-bin/carddisp.pl?gene=SPRY2
JAG1	Jagged Canonical Notch Ligand 1	Protein Coding	P78504	54	GC20M010637	3.185503	https://www.genecards.org/cgi-bin/carddisp.pl?gene=JAG1
AURKA	Aurora Kinase A	Protein Coding	O14965	55	GC20M056370	3.185455	https://www.genecards.org/cgi-bin/carddisp.pl?gene=AURKA
PAX5	Paired Box 5	Protein Coding	Q02548	50	GC09M036968	3.184332	https://www.genecards.org/cgi-bin/carddisp.pl?gene=PAX5
PTPRN	Protein Tyrosine Phosphatase Receptor Type N	Protein Coding	Q16849	49	GC02M219289	3.184247	https://www.genecards.org/cgi-bin/carddisp.pl?gene=PTPRN
INSL3	Insulin Like 3	Protein Coding	P51460	44	GC19M017816	3.181809	https://www.genecards.org/cgi-bin/carddisp.pl?gene=INSL3
APP	Amyloid Beta Precursor Protein	Protein Coding	P05067	54	GC21M025880	3.177453	https://www.genecards.org/cgi-bin/carddisp.pl?gene=APP
BMPR2	Bone Morphogenetic Protein Receptor Type 2	Protein Coding	Q13873	54	GC02P202376	3.175323	https://www.genecards.org/cgi-bin/carddisp.pl?gene=BMPR2
TNFSF12	TNF Superfamily Member 12	Protein Coding	O43508	44	GC17P014020	3.171069	https://www.genecards.org/cgi-bin/carddisp.pl?gene=TNFSF12

NOD2	Nucleotide Binding Oligomerization Domain Containing 2	Protein Coding	Q9HC29	51	GC16P050693	3.168901	https://www.genecards.org/cgi-bin/carddisp.pl?gene=NOD2
SCARB2	Scavenger Receptor Class B Member 2	Protein Coding	Q14108	50	GC04M076158	3.168486	https://www.genecards.org/cgi-bin/carddisp.pl?gene=SCARB2
NEK2	NIMA Related Kinase 2	Protein Coding	P51955	54	GC01M211658	3.167928	https://www.genecards.org/cgi-bin/carddisp.pl?gene=NEK2
CDC123	Cell Division Cycle 123	Protein Coding	O75794	41	GC10P012195	3.166848	https://www.genecards.org/cgi-bin/carddisp.pl?gene=CDC123
CYP11B1	Cytochrome P450 Family 11 Subfamily B Member 1	Protein Coding	P15538	50	GC08M142872	3.163806	https://www.genecards.org/cgi-bin/carddisp.pl?gene=CYP11B1
RHEB	Ras Homolog, MTORC1 Binding	Protein Coding	Q15382	54	GC07M151466	3.159461	https://www.genecards.org/cgi-bin/carddisp.pl?gene=RHEB
RAB6A	RAB6A, Member RAS Oncogene Family	Protein Coding	P20340	45	GC11M113828	3.158973	https://www.genecards.org/cgi-bin/carddisp.pl?gene=RAB6A
CXCL11	C-X-C Motif Chemokine Ligand 11	Protein Coding	O14625	45	GC04M076033	3.155329	https://www.genecards.org/cgi-bin/carddisp.pl?gene=CXCL11
ENSG00000274430		RNA Gene		7	GC0XP073944	3.15248	https://www.genecards.org/cgi-bin/carddisp.pl?gene=ENSG00000274430
ENSG00000276784		RNA Gene		7	GC0XP074062	3.15248	https://www.genecards.org/cgi-bin/carddisp.pl?gene=ENSG00000276784
G6PC3	Glucose-6-Phosphatase Catalytic Subunit 3	Protein Coding	Q9BUM1	45	GC17P044070	3.151043	https://www.genecards.org/cgi-bin/carddisp.pl?gene=G6PC3
RPE65	Retinoid Isomerohydrolase RPE65	Protein Coding	Q16518	49	GC01M068428	3.150378	https://www.genecards.org/cgi-bin/carddisp.pl?gene=RPE65

FANCA	FA Complementation Group A	Protein Coding	O15360	54	GC16M089966	3.149511	https://www.genecards.org/cgi-bin/carddisp.pl?gene=FANCA
COL1A2	Collagen Type I Alpha 2 Chain	Protein Coding	P08123	52	GC07P094394	3.141945	https://www.genecards.org/cgi-bin/carddisp.pl?gene=COL1A2
TSPAN8	Tetraspanin 8	Protein Coding	P19075	44	GC12M071125	3.14181	https://www.genecards.org/cgi-bin/carddisp.pl?gene=TSPAN8
GRIN2A	Glutamate Ionotropic Receptor NMDA Type Subunit 2A	Protein Coding	Q12879	55	GC16M009753	3.13967	https://www.genecards.org/cgi-bin/carddisp.pl?gene=GRIN2A
MIR181D	MicroRNA 181d	RNA Gene		19	GC19P013874	3.138918	https://www.genecards.org/cgi-bin/carddisp.pl?gene=MIR181D
UCA1	Urothelial Cancer Associated 1	RNA Gene		24	GC19P015828	3.138038	https://www.genecards.org/cgi-bin/carddisp.pl?gene=UCA1
DNAH8	Dynein Axonemal Heavy Chain 8	Protein Coding	Q96JB1	41	GC06P112180	3.137508	https://www.genecards.org/cgi-bin/carddisp.pl?gene=DNAH8
ANGPT1	Angiopoietin 1	Protein Coding	Q15389	50	GC08M107246	3.136142	https://www.genecards.org/cgi-bin/carddisp.pl?gene=ANGPT1
CXCL12	C-X-C Motif Chemokine Ligand 12	Protein Coding	P48061	49	GC10M044370	3.135389	https://www.genecards.org/cgi-bin/carddisp.pl?gene=CXCL12
ADAMTS9	ADAM Metallopeptidase With Thrombospondin Type 1 Motif 9	Protein Coding	Q9P2N4	44	GC03M064501	3.135252	https://www.genecards.org/cgi-bin/carddisp.pl?gene=ADAMTS9
UQCRFS1	Ubiquinol-Cytochrome C Reductase, Rieske Iron-Sulfur Polypeptide 1	Protein Coding	P47985	51	GC19M029205	3.133631	https://www.genecards.org/cgi-bin/carddisp.pl?gene=UQCRFS1
SERPINA7	Serpin Family A Member 7	Protein Coding	P05543	45	GC0XM106032	3.132746	https://www.genecards.org/cgi-bin/carddisp.pl?gene=SERPINA7

MIR152	MicroRNA 152	RNA Gene		22	GC17M048037	3.131783	https://www.genecards.org/cgi-bin/carddisp.pl?gene=MIR152
HPRT1	Hypoxanthine Phosphoribosyltransferase 1	Protein Coding	P00492	52	GC0XP134460	3.131663	https://www.genecards.org/cgi-bin/carddisp.pl?gene=HPRT1
MAP3K8	Mitogen-Activated Protein Kinase Kinase Kinase 8	Protein Coding	P41279	50	GC10P030619	3.131367	https://www.genecards.org/cgi-bin/carddisp.pl?gene=MAP3K8
CD44	CD44 Molecule (Indian Blood Group)	Protein Coding	P16070	52	GC11P035139	3.130019	https://www.genecards.org/cgi-bin/carddisp.pl?gene=CD44
VEGFD	Vascular Endothelial Growth Factor D	Protein Coding	O43915	45	GC0XM015345	3.129422	https://www.genecards.org/cgi-bin/carddisp.pl?gene=VEGFD
MIR370	MicroRNA 370	RNA Gene		22	GC14P112882	3.128725	https://www.genecards.org/cgi-bin/carddisp.pl?gene=MIR370
TRPS1	Transcriptional Repressor GATA Binding 1	Protein Coding	Q9UHF7	49	GC08M115408	3.124541	https://www.genecards.org/cgi-bin/carddisp.pl?gene=TRPS1
TRC-GCA24-1	TRNA-Cys (GCA) 24-1	RNA Gene		8	GC17M064009	3.123747	https://www.genecards.org/cgi-bin/carddisp.pl?gene=TRC-GCA24-1
MIR218-1	MicroRNA 218-1	RNA Gene		19	GC04P022269	3.119442	https://www.genecards.org/cgi-bin/carddisp.pl?gene=MIR218-1
ZEB1	Zinc Finger E-Box Binding Homeobox 1	Protein Coding	P37275	53	GC10P031318	3.112307	https://www.genecards.org/cgi-bin/carddisp.pl?gene=ZEB1
GOT1	Glutamic-Oxaloacetic Transaminase 1	Protein Coding	P17174	50	GC10M099396	3.11117	https://www.genecards.org/cgi-bin/carddisp.pl?gene=GOT1
ATXN1	Ataxin 1	Protein Coding	P54253	48	GC06M016299	3.108536	https://www.genecards.org/cgi-bin/carddisp.pl?gene=ATXN1

PCCA	Propionyl-CoA Carboxylase Subunit Alpha	Protein Coding	P05165	51	GC13P100089	3.106516	https://www.genecards.org/cgi-bin/carddisp.pl?gene=PCCA
EIF2B4	Eukaryotic Translation Initiation Factor 2B Subunit Delta	Protein Coding	Q9UI10	47	GC02M027364	3.103596	https://www.genecards.org/cgi-bin/carddisp.pl?gene=EIF2B4
XRCC1	X-Ray Repair Cross Complementing 1	Protein Coding	P18887	48	GC19M043543	3.103535	https://www.genecards.org/cgi-bin/carddisp.pl?gene=XRCC1
PECAM1	Platelet And Endothelial Cell Adhesion Molecule 1	Protein Coding	P16284	45	GC17M064319	3.096055	https://www.genecards.org/cgi-bin/carddisp.pl?gene=PECAM1
LMX1B	LIM Homeobox Transcription Factor 1 Beta	Protein Coding	O60663	48	GC09P126614	3.093935	https://www.genecards.org/cgi-bin/carddisp.pl?gene=LMX1B
PITX2	Paired Like Homeodomain 2	Protein Coding	Q99697	50	GC04M110617	3.093162	https://www.genecards.org/cgi-bin/carddisp.pl?gene=PITX2
NFKBIA	NFKB Inhibitor Alpha	Protein Coding	P25963	54	GC14M035401	3.091275	https://www.genecards.org/cgi-bin/carddisp.pl?gene=NFKBIA
MIR512-1	MicroRNA 512-1	RNA Gene		17	GC19P090136	3.089888	https://www.genecards.org/cgi-bin/carddisp.pl?gene=MIR512-1
C1QTNF3	C1q And TNF Related 3	Protein Coding	Q9BXJ4	42	GC05M034017	3.087857	https://www.genecards.org/cgi-bin/carddisp.pl?gene=C1QTNF3
IRAK1	Interleukin 1 Receptor Associated Kinase 1	Protein Coding	P51617	53	GC0XM154010	3.087429	https://www.genecards.org/cgi-bin/carddisp.pl?gene=IRAK1
PLCE1	Phospholipase C Epsilon 1	Protein Coding	Q9P212	49	GC10P093993	3.083143	https://www.genecards.org/cgi-bin/carddisp.pl?gene=PLCE1
DLG4	Discs Large MAGUK Scaffold Protein 4	Protein Coding	P78352	52	GC17M014377	3.082546	https://www.genecards.org/cgi-bin/carddisp.pl?gene=DLG4

CEP128	Centrosomal Protein 128	Protein Coding	Q6ZU80	37	GC14M080476	3.082176	https://www.genecards.org/cgi-bin/carddisp.pl?gene=CEP128
NKX2-1	NK2 Homeobox 1	Protein Coding	P43699	52	GC14M036516	3.081325	https://www.genecards.org/cgi-bin/carddisp.pl?gene=NKX2-1
METRNL	Meteorin Like, Glial Cell Differentiation Regulator	Protein Coding	Q641Q3	39	GC17P083079	3.07979	https://www.genecards.org/cgi-bin/carddisp.pl?gene=METRNL
GCH1	GTP Cyclohydrolase 1	Protein Coding	P30793	52	GC14M054842	3.077039	https://www.genecards.org/cgi-bin/carddisp.pl?gene=GCH1
MIR24-2	MicroRNA 24-2	RNA Gene		21	GC19M015891	3.073802	https://www.genecards.org/cgi-bin/carddisp.pl?gene=MIR24-2
CLCNKA	Chloride Voltage-Gated Channel Ka	Protein Coding	P51800	45	GC01P016018	3.072083	https://www.genecards.org/cgi-bin/carddisp.pl?gene=CLCNKA
PROK2	Prokineticin 2	Protein Coding	Q9HC23	46	GC03M071771	3.069851	https://www.genecards.org/cgi-bin/carddisp.pl?gene=PROK2
SNORD115-2	Small Nucleolar RNA, C/D Box 115-2	RNA Gene		14	GC15P025172	3.068858	https://www.genecards.org/cgi-bin/carddisp.pl?gene=SNORD115-2
SNORD115-48	Small Nucleolar RNA, C/D Box 115-48	RNA Gene		14	GC15P056616	3.068858	https://www.genecards.org/cgi-bin/carddisp.pl?gene=SNORD115-48
SNORD115-10	Small Nucleolar RNA, C/D Box 115-10	RNA Gene		13	GC15P025187	3.068858	https://www.genecards.org/cgi-bin/carddisp.pl?gene=SNORD115-10
SNORD115-11	Small Nucleolar RNA, C/D Box 115-11	RNA Gene		13	GC15P025189	3.068858	https://www.genecards.org/cgi-bin/carddisp.pl?gene=SNORD115-11
SNORD115-12	Small Nucleolar RNA, C/D Box 115-12	RNA Gene		13	GC15P025191	3.068858	https://www.genecards.org/cgi-bin/carddisp.pl?gene=SNORD115-12

SNORD115-13	Small Nucleolar RNA, C/D Box 115-13	RNA Gene		13	GC15P056620	3.068858	https://www.genecards.org/cgi-bin/carddisp.pl?gene=SNORD115-13
SNORD115-14	Small Nucleolar RNA, C/D Box 115-14	RNA Gene		13	GC15P025194	3.068858	https://www.genecards.org/cgi-bin/carddisp.pl?gene=SNORD115-14
SNORD115-15	Small Nucleolar RNA, C/D Box 115-15	RNA Gene		13	GC15P025197	3.068858	https://www.genecards.org/cgi-bin/carddisp.pl?gene=SNORD115-15
SNORD115-16	Small Nucleolar RNA, C/D Box 115-16	RNA Gene		13	GC15P025199	3.068858	https://www.genecards.org/cgi-bin/carddisp.pl?gene=SNORD115-16
SNORD115-17	Small Nucleolar RNA, C/D Box 115-17	RNA Gene		13	GC15P025201	3.068858	https://www.genecards.org/cgi-bin/carddisp.pl?gene=SNORD115-17
SNORD115-18	Small Nucleolar RNA, C/D Box 115-18	RNA Gene		13	GC15P025203	3.068858	https://www.genecards.org/cgi-bin/carddisp.pl?gene=SNORD115-18
SNORD115-19	Small Nucleolar RNA, C/D Box 115-19	RNA Gene		13	GC15P025204	3.068858	https://www.genecards.org/cgi-bin/carddisp.pl?gene=SNORD115-19
SNORD115-20	Small Nucleolar RNA, C/D Box 115-20	RNA Gene		13	GC15P025206	3.068858	https://www.genecards.org/cgi-bin/carddisp.pl?gene=SNORD115-20
SNORD115-21	Small Nucleolar RNA, C/D Box 115-21	RNA Gene		13	GC15P025208	3.068858	https://www.genecards.org/cgi-bin/carddisp.pl?gene=SNORD115-21
SNORD115-22	Small Nucleolar RNA, C/D Box 115-22	RNA Gene		13	GC15P025209	3.068858	https://www.genecards.org/cgi-bin/carddisp.pl?gene=SNORD115-22
SNORD115-23	Small Nucleolar RNA, C/D Box 115-23	RNA Gene		13	GC15P056603	3.068858	https://www.genecards.org/cgi-bin/carddisp.pl?gene=SNORD115-23
SNORD115-25	Small Nucleolar RNA, C/D Box 115-25	RNA Gene		13	GC15P025215	3.068858	https://www.genecards.org/cgi-bin/carddisp.pl?gene=SNORD115-25

SNORD115-26	Small Nucleolar RNA, C/D Box 115-26	RNA Gene		13	GC15P056625	3.06885<br>8	https://www.genecards.org/cgi-bin/carddisp.pl?gene=SNORD115-26
SNORD115-29	Small Nucleolar RNA, C/D Box 115-29	RNA Gene		13	GC15P056618	3.06885<br>8	https://www.genecards.org/cgi-bin/carddisp.pl?gene=SNORD115-29
SNORD115-3	Small Nucleolar RNA, C/D Box 115-3	RNA Gene		13	GC15P025174	3.06885<br>8	https://www.genecards.org/cgi-bin/carddisp.pl?gene=SNORD115-3
SNORD115-30	Small Nucleolar RNA, C/D Box 115-30	RNA Gene		13	GC15P056619	3.06885<br>8	https://www.genecards.org/cgi-bin/carddisp.pl?gene=SNORD115-30
SNORD115-31	Small Nucleolar RNA, C/D Box 115-31	RNA Gene		13	GC15P056621	3.06885<br>8	https://www.genecards.org/cgi-bin/carddisp.pl?gene=SNORD115-31
SNORD115-32	Small Nucleolar RNA, C/D Box 115-32	RNA Gene		13	GC15P056622	3.06885<br>8	https://www.genecards.org/cgi-bin/carddisp.pl?gene=SNORD115-32
SNORD115-33	Small Nucleolar RNA, C/D Box 115-33	RNA Gene		13	GC15P056623	3.06885<br>8	https://www.genecards.org/cgi-bin/carddisp.pl?gene=SNORD115-33
SNORD115-34	Small Nucleolar RNA, C/D Box 115-34	RNA Gene		13	GC15P056624	3.06885<br>8	https://www.genecards.org/cgi-bin/carddisp.pl?gene=SNORD115-34
SNORD115-35	Small Nucleolar RNA, C/D Box 115-35	RNA Gene		13	GC15P056627	3.06885<br>8	https://www.genecards.org/cgi-bin/carddisp.pl?gene=SNORD115-35
SNORD115-36	Small Nucleolar RNA, C/D Box 115-36	RNA Gene		13	GC15P056628	3.06885<br>8	https://www.genecards.org/cgi-bin/carddisp.pl?gene=SNORD115-36
SNORD115-37	Small Nucleolar RNA, C/D Box 115-37	RNA Gene		13	GC15P056629	3.06885<br>8	https://www.genecards.org/cgi-bin/carddisp.pl?gene=SNORD115-37
SNORD115-38	Small Nucleolar RNA, C/D Box 115-38	RNA Gene		13	GC15P056605	3.06885<br>8	https://www.genecards.org/cgi-bin/carddisp.pl?gene=SNORD115-38

SNORD115-39	Small Nucleolar RNA, C/D Box 115-39	RNA Gene		13	GC15P056606	3.06885 8	https://www.genecards.org/cgi-bin/carddisp.pl?gene=SNORD115-39
SNORD115-4	Small Nucleolar RNA, C/D Box 115-4	RNA Gene		13	GC15P025176	3.06885 8	https://www.genecards.org/cgi-bin/carddisp.pl?gene=SNORD115-4
SNORD115-40	Small Nucleolar RNA, C/D Box 115-40	RNA Gene		13	GC15P056607	3.06885 8	https://www.genecards.org/cgi-bin/carddisp.pl?gene=SNORD115-40
SNORD115-41	Small Nucleolar RNA, C/D Box 115-41	RNA Gene		13	GC15P056608	3.06885 8	https://www.genecards.org/cgi-bin/carddisp.pl?gene=SNORD115-41
SNORD115-42	Small Nucleolar RNA, C/D Box 115-42	RNA Gene		13	GC15P056609	3.06885 8	https://www.genecards.org/cgi-bin/carddisp.pl?gene=SNORD115-42
SNORD115-43	Small Nucleolar RNA, C/D Box 115-43	RNA Gene		13	GC15P056611	3.06885 8	https://www.genecards.org/cgi-bin/carddisp.pl?gene=SNORD115-43
SNORD115-44	Small Nucleolar RNA, C/D Box 115-44	RNA Gene		13	GC15P056612	3.06885 8	https://www.genecards.org/cgi-bin/carddisp.pl?gene=SNORD115-44
SNORD115-5	Small Nucleolar RNA, C/D Box 115-5	RNA Gene		13	GC15P025178	3.06885 8	https://www.genecards.org/cgi-bin/carddisp.pl?gene=SNORD115-5
SNORD115-6	Small Nucleolar RNA, C/D Box 115-6	RNA Gene		13	GC15P025180	3.06885 8	https://www.genecards.org/cgi-bin/carddisp.pl?gene=SNORD115-6
SNORD115-7	Small Nucleolar RNA, C/D Box 115-7	RNA Gene		13	GC15P056604	3.06885 8	https://www.genecards.org/cgi-bin/carddisp.pl?gene=SNORD115-7
SNORD115-8	Small Nucleolar RNA, C/D Box 115-8	RNA Gene		13	GC15P025184	3.06885 8	https://www.genecards.org/cgi-bin/carddisp.pl?gene=SNORD115-8
SNORD115-9	Small Nucleolar RNA, C/D Box 115-9	RNA Gene		13	GC15P025185	3.06885 8	https://www.genecards.org/cgi-bin/carddisp.pl?gene=SNORD115-9

FGF9	Fibroblast Growth Factor 9	Protein Coding	P31371	48	GC13P021671	3.068301	https://www.genecards.org/cgi-bin/carddisp.pl?gene=FGF9
NOTCH2	Notch Receptor 2	Protein Coding	Q04721	55	GC01M119911	3.067433	https://www.genecards.org/cgi-bin/carddisp.pl?gene=NOTCH2
GRIA1	Glutamate Ionotropic Receptor AMPA Type Subunit 1	Protein Coding	P42261	53	GC05P153467	3.067195	https://www.genecards.org/cgi-bin/carddisp.pl?gene=GRIA1
GJA4	Gap Junction Protein Alpha 4	Protein Coding	P35212	46	GC01P034792	3.067096	https://www.genecards.org/cgi-bin/carddisp.pl?gene=GJA4
L2HGDH	L-2-Hydroxyglutarate Dehydrogenase	Protein Coding	Q9H9P8	44	GC14M050237	3.063555	https://www.genecards.org/cgi-bin/carddisp.pl?gene=L2HGDH
GHRHR	Growth Hormone Releasing Hormone Receptor	Protein Coding	Q02643	47	GC07P030938	3.059217	https://www.genecards.org/cgi-bin/carddisp.pl?gene=GHRHR
ROR2	Receptor Tyrosine Kinase Like Orphan Receptor 2	Protein Coding	Q01974	53	GC09M104363	3.057575	https://www.genecards.org/cgi-bin/carddisp.pl?gene=ROR2
ASS1	Argininosuccinate Synthase 1	Protein Coding	P00966	53	GC09P130444	3.056925	https://www.genecards.org/cgi-bin/carddisp.pl?gene=ASS1
SNORD116-13	Small Nucleolar RNA, C/D Box 116-13	RNA Gene		13	GC15P025079	3.055684	https://www.genecards.org/cgi-bin/carddisp.pl?gene=SNORD116-13
SNORD116-14	Small Nucleolar RNA, C/D Box 116-14	RNA Gene		13	GC15P025080	3.055684	https://www.genecards.org/cgi-bin/carddisp.pl?gene=SNORD116-14
SNORD116-17	Small Nucleolar RNA, C/D Box 116-17	RNA Gene		13	GC15P025083	3.055684	https://www.genecards.org/cgi-bin/carddisp.pl?gene=SNORD116-17
SNORD116-18	Small Nucleolar RNA, C/D Box 116-18	RNA Gene		13	GC15P025085	3.055684	https://www.genecards.org/cgi-bin/carddisp.pl?gene=SNORD116-18

PITX1	Paired Like Homeodomain 1	Protein Coding	P78337	48	GC05M135027	3.049842	https://www.genecards.org/cgi-bin/carddisp.pl?gene=PITX1
MIAT	Myocardial Infarction Associated Transcript	RNA Gene		25	GC22P026646	3.047906	https://www.genecards.org/cgi-bin/carddisp.pl?gene=MIAT
COL4A5	Collagen Type IV Alpha 5 Chain	Protein Coding	P29400	48	GC0XP108439	3.047488	https://www.genecards.org/cgi-bin/carddisp.pl?gene=COL4A5
NR0B1	Nuclear Receptor Subfamily 0 Group B Member 1	Protein Coding	P51843	50	GC0XM030304	3.044969	https://www.genecards.org/cgi-bin/carddisp.pl?gene=NR0B1
MT-TA	Mitochondrially Encoded TRNA-Ala (GCN)	RNA Gene		11	GCMTM005589	3.038597	https://www.genecards.org/cgi-bin/carddisp.pl?gene=MT-TA
MRPS22	Mitochondrial Ribosomal Protein S22	Protein Coding	P82650	46	GC03P139005	3.038505	https://www.genecards.org/cgi-bin/carddisp.pl?gene=MRPS22
THORLNC	Testis Associated Oncogenic LncRNA	RNA Gene		15	GC02M118133	3.036908	https://www.genecards.org/cgi-bin/carddisp.pl?gene=THORLNC
GTPBP3	GTP Binding Protein 3, Mitochondrial	Protein Coding	Q969Y2	45	GC19P089096	3.032867	https://www.genecards.org/cgi-bin/carddisp.pl?gene=GTPBP3
EYA1	EYA Transcriptional Coactivator And Phosphatase 1	Protein Coding	Q99502	48	GC08M071210	3.032071	https://www.genecards.org/cgi-bin/carddisp.pl?gene=EYA1
LOC107982234	WT1/WT1-AS Bi-Directional Promoter Region	Functional Element		4	GC11P032430	3.03178	https://www.genecards.org/cgi-bin/carddisp.pl?gene=LOC107982234
SCT	Secretin	Protein Coding	P09683	37	GC11M000626	3.030159	https://www.genecards.org/cgi-bin/carddisp.pl?gene=SCT
PWARSN	Prader Willi/Angelman Region RNA, SNRPN Neighbor	RNA Gene		13	GC15P056592	3.027946	https://www.genecards.org/cgi-bin/carddisp.pl?gene=PWARSN

OFD1	OFD1 Centriole And Centriolar Satellite Protein	Protein Coding	O75665	47	GC0XP013714	3.02417	https://www.genecards.org/cgi-bin/carddisp.pl?gene=OFD1
MIR7-1	MicroRNA 7-1	RNA Gene		17	GC09M104169	3.023743	https://www.genecards.org/cgi-bin/carddisp.pl?gene=MIR7-1
TNFSF10	TNF Superfamily Member 10	Protein Coding	P50591	48	GC03M172505	3.022705	https://www.genecards.org/cgi-bin/carddisp.pl?gene=TNFSF10
SLC38A2	Solute Carrier Family 38 Member 2	Protein Coding	Q96QD8	43	GC12M046358	3.022683	https://www.genecards.org/cgi-bin/carddisp.pl?gene=SLC38A2
IDE	Insulin Degrading Enzyme	Protein Coding	P14735	52	GC10M092451	3.020167	https://www.genecards.org/cgi-bin/carddisp.pl?gene=IDE
TUBGCP5	Tubulin Gamma Complex Component 5	Protein Coding	Q96RT8	39	GC15M022983	3.01858	https://www.genecards.org/cgi-bin/carddisp.pl?gene=TUBGCP5
NF2	NF2, Moesin-Ezrin-Radixin Like (MERLIN) Tumor Suppressor	Protein Coding	P35240	53	GC22P029603	3.017905	https://www.genecards.org/cgi-bin/carddisp.pl?gene=NF2
NBN	Nibrin	Protein Coding	O60934	52	GC08M089933	3.016535	https://www.genecards.org/cgi-bin/carddisp.pl?gene=NBN
CC2D2A	Coiled-Coil And C2 Domain Containing 2A	Protein Coding	Q9P2K1	44	GC04P022191	3.015657	https://www.genecards.org/cgi-bin/carddisp.pl?gene=CC2D2A
CACNA1S	Calcium Voltage-Gated Channel Subunit Alpha1 S	Protein Coding	Q13698	52	GC01M201008	3.009563	https://www.genecards.org/cgi-bin/carddisp.pl?gene=CACNA1S
ABCA4	ATP Binding Cassette Subfamily A Member 4	Protein Coding	P78363	48	GC01M093992	3.009192	https://www.genecards.org/cgi-bin/carddisp.pl?gene=ABCA4
EPAS1	Endothelial PAS Domain Protein 1	Protein Coding	Q99814	54	GC02P046293	3.005797	https://www.genecards.org/cgi-bin/carddisp.pl?gene=EPAS1

ESM1	Endothelial Cell Specific Molecule 1	Protein Coding	Q9NQ30	43	GC05M054977	2.999527	https://www.genecards.org/cgi-bin/carddisp.pl?gene=ESM1
SNORD116-12	Small Nucleolar RNA, C/D Box 116-12	RNA Gene		14	GC15P056599	2.997979	https://www.genecards.org/cgi-bin/carddisp.pl?gene=SNORD116-12
SNORD116-10	Small Nucleolar RNA, C/D Box 116-10	RNA Gene		13	GC15P056597	2.997979	https://www.genecards.org/cgi-bin/carddisp.pl?gene=SNORD116-10
SNORD116-11	Small Nucleolar RNA, C/D Box 116-11	RNA Gene		13	GC15P056598	2.997979	https://www.genecards.org/cgi-bin/carddisp.pl?gene=SNORD116-11
SNORD116-15	Small Nucleolar RNA, C/D Box 116-15	RNA Gene		13	GC15P025081	2.997979	https://www.genecards.org/cgi-bin/carddisp.pl?gene=SNORD116-15
SNORD116-16	Small Nucleolar RNA, C/D Box 116-16	RNA Gene		13	GC15P025082	2.997979	https://www.genecards.org/cgi-bin/carddisp.pl?gene=SNORD116-16
SNORD115-24	Small Nucleolar RNA, C/D Box 115-24	RNA Gene		12	GC15P025213	2.997979	https://www.genecards.org/cgi-bin/carddisp.pl?gene=SNORD115-24
SNORD115-27	Small Nucleolar RNA, C/D Box 115-27	RNA Gene		12	GC15P025220	2.997979	https://www.genecards.org/cgi-bin/carddisp.pl?gene=SNORD115-27
SNORD115-28	Small Nucleolar RNA, C/D Box 115-28	RNA Gene		12	GC15P025222	2.997979	https://www.genecards.org/cgi-bin/carddisp.pl?gene=SNORD115-28
SNORD115-45	Small Nucleolar RNA, C/D Box 115-45	RNA Gene		12	GC15P056613	2.997979	https://www.genecards.org/cgi-bin/carddisp.pl?gene=SNORD115-45
SNORD115-47	Small Nucleolar RNA, C/D Box 115-47	RNA Gene		11	GC15P056614	2.997979	https://www.genecards.org/cgi-bin/carddisp.pl?gene=SNORD115-47
BTK	Bruton Tyrosine Kinase	Protein Coding	Q06187	58	GC0XM101349	2.997737	https://www.genecards.org/cgi-bin/carddisp.pl?gene=BTK

SPG7	SPG7 Matrix AAA Peptidase Subunit, Paraplegin	Protein Coding	Q9UQ90	47	GC16P092247	2.996049	https://www.genecards.org/cgi-bin/carddisp.pl?gene=SPG7
MIR885	MicroRNA 885	RNA Gene		18	GC03M010698	2.993992	https://www.genecards.org/cgi-bin/carddisp.pl?gene=MIR885
ERCC2	ERCC Excision Repair 2, TFIIH Core Complex Helicase Subunit	Protein Coding	P18074	52	GC19M045349	2.99345	https://www.genecards.org/cgi-bin/carddisp.pl?gene=ERCC2
BDKRB2	Bradykinin Receptor B2	Protein Coding	P30411	48	GC14P096205	2.993054	https://www.genecards.org/cgi-bin/carddisp.pl?gene=BDKRB2
PIEZO1	Piezo Type Mechanosensitive Ion Channel Component 1	Protein Coding	Q92508	45	GC16M088715	2.990634	https://www.genecards.org/cgi-bin/carddisp.pl?gene=PIEZO1
CXCL9	C-X-C Motif Chemokine Ligand 9	Protein Coding	Q07325	44	GC04M076001	2.984543	https://www.genecards.org/cgi-bin/carddisp.pl?gene=CXCL9
ANOS1	Anosmin 1	Protein Coding	P23352	44	GC0XM008528	2.981907	https://www.genecards.org/cgi-bin/carddisp.pl?gene=ANOS1
KEAP1	Kelch Like ECH Associated Protein 1	Protein Coding	Q14145	53	GC19M010486	2.977464	https://www.genecards.org/cgi-bin/carddisp.pl?gene=KEAP1
IL1RL1	Interleukin 1 Receptor Like 1	Protein Coding	Q01638	46	GC02P102294	2.976473	https://www.genecards.org/cgi-bin/carddisp.pl?gene=IL1RL1
SLX1A-SULT1A3	SLX1A-SULT1A3 Readthrough (NMD Candidate)	RNA Gene		14	GC16P054141	2.976451	https://www.genecards.org/cgi-bin/carddisp.pl?gene=SLX1A-SULT1A3
FSHB	Follicle Stimulating Hormone Subunit Beta	Protein Coding	P01225	48	GC11P030210	2.972761	https://www.genecards.org/cgi-bin/carddisp.pl?gene=FSHB
PRKCA	Protein Kinase C Alpha	Protein Coding	P17252	54	GC17P066302	2.969725	https://www.genecards.org/cgi-bin/carddisp.pl?gene=PRKCA

TREM1	Triggering Receptor Expressed On Myeloid Cells 1	Protein Coding	Q9NP99	46	GC06M041267	2.967973	https://www.genecards.org/cgi-bin/carddisp.pl?gene=TREM1
RPGR	Retinitis Pigmentosa GTPase Regulator	Protein Coding	Q92834	45	GC0XM038269	2.966988	https://www.genecards.org/cgi-bin/carddisp.pl?gene=RPGR
GTF2IRD1	GTF2I Repeat Domain Containing 1	Protein Coding	Q9UHL9	46	GC07P081232	2.965086	https://www.genecards.org/cgi-bin/carddisp.pl?gene=GTF2IRD1
FKBP6	FKBP Prolyl Isomerase Family Member 6 (Inactive)	Protein Coding	O75344	43	GC07P073328	2.965086	https://www.genecards.org/cgi-bin/carddisp.pl?gene=FKBP6
BUD23	BUD23 RRNA Methyltransferase And Ribosome Maturation Factor	Protein Coding	O43709	40	GC07P081212	2.965086	https://www.genecards.org/cgi-bin/carddisp.pl?gene=BUD23
SMC1A	Structural Maintenance Of Chromosomes 1A	Protein Coding	Q14683	52	GC0XM053374	2.963739	https://www.genecards.org/cgi-bin/carddisp.pl?gene=SMC1A
SMC3	Structural Maintenance Of Chromosomes 3	Protein Coding	Q9UQE7	51	GC10P110567	2.963739	https://www.genecards.org/cgi-bin/carddisp.pl?gene=SMC3
SMARCE1	SWI/SNF Related, Matrix Associated, Actin Dependent Regulator Of Chromatin, Subfamily E, Member 1	Protein Coding	Q969G3	50	GC17M040624	2.960504	https://www.genecards.org/cgi-bin/carddisp.pl?gene=SMARCE1
LOC109461477	Dystrophia Myotonica Protein Kinase Repeat Instability Region	Functional Element		4	GC19P045770	2.958033	https://www.genecards.org/cgi-bin/carddisp.pl?gene=LOC109461477
HSPA6	Heat Shock Protein Family A (Hsp70) Member 6	Protein Coding	P17066	48	GC01P161524	2.955701	https://www.genecards.org/cgi-bin/carddisp.pl?gene=HSPA6
MIR802	MicroRNA 802	RNA Gene		18	GC21P035720	2.95539	https://www.genecards.org/cgi-bin/carddisp.pl?gene=MIR802
IGFBP5	Insulin Like Growth Factor Binding Protein 5	Protein Coding	P24593	47	GC02M216672	2.955197	https://www.genecards.org/cgi-bin/carddisp.pl?gene=IGFBP5

AMD1	Adenosylmethionine Decarboxylase 1	Protein Coding	P17707	48	GC06P110814	2.954866	https://www.genecards.org/cgi-bin/carddisp.pl?gene=AMD1
LARS2	Leucyl-TRNA Synthetase 2, Mitochondrial	Protein Coding	Q15031	48	GC03P053726	2.953119	https://www.genecards.org/cgi-bin/carddisp.pl?gene=LARS2
MIRLET7A2	MicroRNA Let-7a-2	RNA Gene		21	GC11M122146	2.951288	https://www.genecards.org/cgi-bin/carddisp.pl?gene=MIRLET7A2
MIRLET7A3	MicroRNA Let-7a-3	RNA Gene		20	GC22P046112	2.951288	https://www.genecards.org/cgi-bin/carddisp.pl?gene=MIRLET7A3
TBX20	T-Box Transcription Factor 20	Protein Coding	Q9UMR3	45	GC07M035237	2.949896	https://www.genecards.org/cgi-bin/carddisp.pl?gene=TBX20
CHUK	Component Of Inhibitor Of Nuclear Factor Kappa B Kinase Complex	Protein Coding	O15111	54	GC10M100311	2.948787	https://www.genecards.org/cgi-bin/carddisp.pl?gene=CHUK
CYBB	Cytochrome B-245 Beta Chain	Protein Coding	P04839	53	GC0XP037780	2.94296	https://www.genecards.org/cgi-bin/carddisp.pl?gene=CYBB
KIR2DL1	Killer Cell Immunoglobulin Like Receptor, Two Ig Domains And Long Cytoplasmic Tail 1	Protein Coding	P43626	40	GC19P090182	2.94242	https://www.genecards.org/cgi-bin/carddisp.pl?gene=KIR2DL1
LINC00473	Long Intergenic Non-Protein Coding RNA 473	RNA Gene	A8K010	23	GC06M165328	2.938528	https://www.genecards.org/cgi-bin/carddisp.pl?gene=LINC00473
ADAMTS13	ADAM Metallopeptidase With Thrombospondin Type 1 Motif 13	Protein Coding	Q76LX8	52	GC09P133414	2.936466	https://www.genecards.org/cgi-bin/carddisp.pl?gene=ADAMTS13
ERCC4	ERCC Excision Repair 4, Endonuclease Catalytic Subunit	Protein Coding	Q92889	50	GC16P013920	2.932925	https://www.genecards.org/cgi-bin/carddisp.pl?gene=ERCC4
CDCA3	Cell Division Cycle Associated 3	Protein Coding	Q99618	42	GC12M006844	2.932561	https://www.genecards.org/cgi-bin/carddisp.pl?gene=CDCA3

TLR5	Toll Like Receptor 5	Protein Coding	O60602	50	GC01M223109	2.928602	https://www.genecards.org/cgi-bin/carddisp.pl?gene=TLR5
GABRA5	Gamma-Aminobutyric Acid Type A Receptor Subunit Alpha5	Protein Coding	P31644	52	GC15P026866	2.928397	https://www.genecards.org/cgi-bin/carddisp.pl?gene=GABRA5
COL5A2	Collagen Type V Alpha 2 Chain	Protein Coding	P05997	48	GC02M189031	2.922345	https://www.genecards.org/cgi-bin/carddisp.pl?gene=COL5A2
PIGF	Phosphatidylinositol Glycan Anchor Biosynthesis Class F	Protein Coding	Q07326	42	GC02M046580	2.918381	https://www.genecards.org/cgi-bin/carddisp.pl?gene=PIGF
MIR375	MicroRNA 375	RNA Gene		21	GC02M219001	2.916954	https://www.genecards.org/cgi-bin/carddisp.pl?gene=MIR375
AHRR	Aryl Hydrocarbon Receptor Repressor	Protein Coding	A9YTQ3	40	GC05P000321	2.916424	https://www.genecards.org/cgi-bin/carddisp.pl?gene=AHRR
BMPR1A	Bone Morphogenetic Protein Receptor Type 1A	Protein Coding	P36894	55	GC10P100881	2.911281	https://www.genecards.org/cgi-bin/carddisp.pl?gene=BMPR1A
MAGED1	MAGE Family Member D1	Protein Coding	Q9Y5V3	46	GC0XP051803	2.910394	https://www.genecards.org/cgi-bin/carddisp.pl?gene=MAGED1
MT1A	Metallothionein 1A	Protein Coding	P04731	42	GC16P056638	2.908831	https://www.genecards.org/cgi-bin/carddisp.pl?gene=MT1A
STK11	Serine/Threonine Kinase 11	Protein Coding	Q15831	54	GC19P001177	2.908087	https://www.genecards.org/cgi-bin/carddisp.pl?gene=STK11
RBFOX1	RNA Binding Fox-1 Homolog 1	Protein Coding	Q9NWB1	44	GC16P052545	2.907381	https://www.genecards.org/cgi-bin/carddisp.pl?gene=RBFOX1
SNHG1	Small Nucleolar RNA Host Gene 1	RNA Gene		21	GC11M113407	2.903465	https://www.genecards.org/cgi-bin/carddisp.pl?gene=SNHG1

PDE3A	Phosphodiesterase 3A	Protein Coding	Q14432	52	GC12P030877	2.89905	https://www.genecards.org/cgi-bin/carddisp.pl?gene=PDE3A
CD55	CD55 Molecule (Cromer Blood Group)	Protein Coding	P08174	53	GC01P207321	2.897238	https://www.genecards.org/cgi-bin/carddisp.pl?gene=CD55
ENSG00000275307		RNA Gene		8	GC22P056442	2.897092	https://www.genecards.org/cgi-bin/carddisp.pl?gene=ENSG00000275307
ENSG00000276965		RNA Gene		8	GC22P056441	2.897092	https://www.genecards.org/cgi-bin/carddisp.pl?gene=ENSG00000276965
CYP2C9	Cytochrome P450 Family 2 Subfamily C Member 9	Protein Coding	P11712	50	GC10P094938	2.896517	https://www.genecards.org/cgi-bin/carddisp.pl?gene=CYP2C9
CFTR	CF Transmembrane Conductance Regulator	Protein Coding	P13569	55	GC07P117287	2.892633	https://www.genecards.org/cgi-bin/carddisp.pl?gene=CFTR
MIR373	MicroRNA 373	RNA Gene		20	GC19P090150	2.888738	https://www.genecards.org/cgi-bin/carddisp.pl?gene=MIR373
SMCHD1	Structural Maintenance Of Chromosomes Flexible Hinge Domain Containing 1	Protein Coding	A6NHR9	44	GC18P002649	2.888505	https://www.genecards.org/cgi-bin/carddisp.pl?gene=SMCHD1
IL7R	Interleukin 7 Receptor	Protein Coding	P16871	50	GC05P035852	2.866343	https://www.genecards.org/cgi-bin/carddisp.pl?gene=IL7R
ZFAS1	ZNFX1 Antisense RNA 1	RNA Gene		21	GC20P049276	2.864812	https://www.genecards.org/cgi-bin/carddisp.pl?gene=ZFAS1
SLCO1B1	Solute Carrier Organic Anion Transporter Family Member 1B1	Protein Coding	Q9Y6L6	51	GC12P030887	2.861444	https://www.genecards.org/cgi-bin/carddisp.pl?gene=SLCO1B1
SYP	Synaptophysin	Protein Coding	P08247	50	GC0XM049187	2.860292	https://www.genecards.org/cgi-bin/carddisp.pl?gene=SYP

MIRLET7G	MicroRNA Let-7g	RNA Gene		20	GC03M052268	2.857589	https://www.genecards.org/cgi-bin/carddisp.pl?gene=MIRLET7G
LOC113939944	Sharpr-MPRA Regulatory Region 9539	Functional Element		4	GC15P048520	2.857354	https://www.genecards.org/cgi-bin/carddisp.pl?gene=LOC113939944
SOST	Sclerostin	Protein Coding	Q9BQB4	47	GC17M043753	2.853761	https://www.genecards.org/cgi-bin/carddisp.pl?gene=SOST
NPPC	Natriuretic Peptide C	Protein Coding	P23582	45	GC02M231921	2.845136	https://www.genecards.org/cgi-bin/carddisp.pl?gene=NPPC
ACVR2A	Activin A Receptor Type 2A	Protein Coding	P27037	50	GC02P147844	2.84512	https://www.genecards.org/cgi-bin/carddisp.pl?gene=ACVR2A
KITLG	KIT Ligand	Protein Coding	P21583	51	GC12M088492	2.843486	https://www.genecards.org/cgi-bin/carddisp.pl?gene=KITLG
MIR340	MicroRNA 340	RNA Gene		20	GC05M180015	2.842694	https://www.genecards.org/cgi-bin/carddisp.pl?gene=MIR340
PLAC4	Placenta Enriched 4	RNA Gene	Q8WY50	27	GC21M041176	2.842456	https://www.genecards.org/cgi-bin/carddisp.pl?gene=PLAC4
ATXN3	Ataxin 3	Protein Coding	P54252	49	GC14M116370	2.838478	https://www.genecards.org/cgi-bin/carddisp.pl?gene=ATXN3
KLF14	KLF Transcription Factor 14	Protein Coding	Q8TD94	37	GC07M130731	2.838256	https://www.genecards.org/cgi-bin/carddisp.pl?gene=KLF14
HS6ST1	Heparan Sulfate 6-O-Sulfotransferase 1	Protein Coding	O60243	46	GC02M128236	2.83779	https://www.genecards.org/cgi-bin/carddisp.pl?gene=HS6ST1
PKP2	Plakophilin 2	Protein Coding	Q99959	48	GC12M032790	2.83584	https://www.genecards.org/cgi-bin/carddisp.pl?gene=PKP2

SGCE	Sarcoglycan Epsilon	Protein Coding	O43556	47	GC07M094524	2.835202	https://www.genecards.org/cgi-bin/carddisp.pl?gene=SGCE
TTN	Titin	Protein Coding	Q8WZ42	52	GC02M178525	2.833707	https://www.genecards.org/cgi-bin/carddisp.pl?gene=TTN
SHMT1	Serine Hydroxymethyltransferase 1	Protein Coding	P34896	48	GC17M063032	2.83322	https://www.genecards.org/cgi-bin/carddisp.pl?gene=SHMT1
PKD1	Polycystin 1, Transient Receptor Potential Channel Interacting	Protein Coding	P98161	50	GC16M013321	2.829761	https://www.genecards.org/cgi-bin/carddisp.pl?gene=PKD1
CYB5A	Cytochrome B5 Type A	Protein Coding	P00167	48	GC18M074250	2.827831	https://www.genecards.org/cgi-bin/carddisp.pl?gene=CYB5A
P2RX7	Purinergic Receptor P2X 7	Protein Coding	Q99572	51	GC12P129102	2.82442	https://www.genecards.org/cgi-bin/carddisp.pl?gene=P2RX7
MIR19A	MicroRNA 19a	RNA Gene		21	GC13P091631	2.821243	https://www.genecards.org/cgi-bin/carddisp.pl?gene=MIR19A
TNFRSF11A	TNF Receptor Superfamily Member 11a	Protein Coding	Q9Y6Q6	51	GC18P062325	2.82109	https://www.genecards.org/cgi-bin/carddisp.pl?gene=TNFRSF11A
MIR100HG	Mir-100-Let-7a-2-Mir-125b-1 Cluster Host Gene	RNA Gene		20	GC11M122029	2.818805	https://www.genecards.org/cgi-bin/carddisp.pl?gene=MIR100HG
VCAN	Versican	Protein Coding	P13611	51	GC05P083471	2.818127	https://www.genecards.org/cgi-bin/carddisp.pl?gene=VCAN
GPD2	Glycerol-3-Phosphate Dehydrogenase 2	Protein Coding	P43304	50	GC02P156401	2.805952	https://www.genecards.org/cgi-bin/carddisp.pl?gene=GPD2
RAB39B	RAB39B, Member RAS Oncogene Family	Protein Coding	Q96DA2	42	GC0XM155259	2.800295	https://www.genecards.org/cgi-bin/carddisp.pl?gene=RAB39B

CASP9	Caspase 9	Protein Coding	P55211	51	GC01M015491	2.799838	https://www.genecards.org/cgi-bin/carddisp.pl?gene=CASP9
SERPINB2	Serpin Family B Member 2	Protein Coding	P05120	48	GC18P063871	2.799487	https://www.genecards.org/cgi-bin/carddisp.pl?gene=SERPINB2
EIF4EBP1	Eukaryotic Translation Initiation Factor 4E Binding Protein 1	Protein Coding	Q13541	50	GC08P039055	2.79528	https://www.genecards.org/cgi-bin/carddisp.pl?gene=EIF4EBP1
SNORD116-26	Small Nucleolar RNA, C/D Box 116-26	RNA Gene		14	GC15P025099	2.795141	https://www.genecards.org/cgi-bin/carddisp.pl?gene=SNORD116-26
SNORD116-27	Small Nucleolar RNA, C/D Box 116-27	RNA Gene		14	GC15P056602	2.795141	https://www.genecards.org/cgi-bin/carddisp.pl?gene=SNORD116-27
SNORD116-19	Small Nucleolar RNA, C/D Box 116-19	RNA Gene		13	GC15P025086	2.795141	https://www.genecards.org/cgi-bin/carddisp.pl?gene=SNORD116-19
SNORD116-5	Small Nucleolar RNA, C/D Box 116-5	RNA Gene		13	GC15P025062	2.795141	https://www.genecards.org/cgi-bin/carddisp.pl?gene=SNORD116-5
SNORD116-6	Small Nucleolar RNA, C/D Box 116-6	RNA Gene		13	GC15P025065	2.795141	https://www.genecards.org/cgi-bin/carddisp.pl?gene=SNORD116-6
SNORD116-7	Small Nucleolar RNA, C/D Box 116-7	RNA Gene		13	GC15P025067	2.795141	https://www.genecards.org/cgi-bin/carddisp.pl?gene=SNORD116-7
MIR543	MicroRNA 543	RNA Gene		17	GC14P112925	2.791248	https://www.genecards.org/cgi-bin/carddisp.pl?gene=MIR543
HDAC1	Histone Deacetylase 1	Protein Coding	Q13547	54	GC01P032292	2.789365	https://www.genecards.org/cgi-bin/carddisp.pl?gene=HDAC1
ASPRV1	Aspartic Peptidase Retroviral Like 1	Protein Coding	Q53RT3	41	GC02M069932	2.785509	https://www.genecards.org/cgi-bin/carddisp.pl?gene=ASPRV1

MIR103A2	MicroRNA 103a-2	RNA Gene		19	GC20P003917	2.784953	https://www.genecards.org/cgi-bin/carddisp.pl?gene=MIR103A2
IKBKE	Inhibitor Of Nuclear Factor Kappa B Kinase Subunit Epsilon	Protein Coding	Q14164	48	GC01P206470	2.780311	https://www.genecards.org/cgi-bin/carddisp.pl?gene=IKBKE
IFNA1	Interferon Alpha 1	Protein Coding	P01562	43	GC09P021662	2.779641	https://www.genecards.org/cgi-bin/carddisp.pl?gene=IFNA1
CTF1	Cardiotrophin 1	Protein Coding	Q16619	42	GC16P054214	2.777923	https://www.genecards.org/cgi-bin/carddisp.pl?gene=CTF1
NME1	NME/NM23 Nucleoside Diphosphate Kinase 1	Protein Coding	P15531	49	GC17P086730	2.776002	https://www.genecards.org/cgi-bin/carddisp.pl?gene=NME1
MOG	Myelin Oligodendrocyte Glycoprotein	Protein Coding	Q16653	50	GC06P111935	2.773766	https://www.genecards.org/cgi-bin/carddisp.pl?gene=MOG
SLC27A4	Solute Carrier Family 27 Member 4	Protein Coding	Q6P1M0	48	GC09P128340	2.772743	https://www.genecards.org/cgi-bin/carddisp.pl?gene=SLC27A4
PTER	Phosphotriesterase Related	Protein Coding	Q96BW5	43	GC10P016436	2.771667	https://www.genecards.org/cgi-bin/carddisp.pl?gene=PTER
GUSB	Glucuronidase Beta	Protein Coding	P08236	52	GC07M065960	2.771131	https://www.genecards.org/cgi-bin/carddisp.pl?gene=GUSB
IL5RA	Interleukin 5 Receptor Subunit Alpha	Protein Coding	Q01344	51	GC03M003066	2.770785	https://www.genecards.org/cgi-bin/carddisp.pl?gene=IL5RA
IFT140	Intraflagellar Transport 140	Protein Coding	Q96RY7	45	GC16M013290	2.770514	https://www.genecards.org/cgi-bin/carddisp.pl?gene=IFT140
RAC1	Rac Family Small GTPase 1	Protein Coding	P63000	53	GC07P007923	2.769376	https://www.genecards.org/cgi-bin/carddisp.pl?gene=RAC1

CARMN	Cardiac Mesoderm Enhancer-Associated Non-Coding RNA	RNA Gene		21	GC05P149397	2.768408	https://www.genecards.org/cgi-bin/carddisp.pl?gene=CARMN
MT-TR	Mitochondrially Encoded TRNA-Arg (CGN)	RNA Gene		14	GCMTP010407	2.762213	https://www.genecards.org/cgi-bin/carddisp.pl?gene=MT-TR
FBN2	Fibrillin 2	Protein Coding	P35556	46	GC05M128257	2.756698	https://www.genecards.org/cgi-bin/carddisp.pl?gene=FBN2
CD163	CD163 Molecule	Protein Coding	Q86VB7	48	GC12M008723	2.756312	https://www.genecards.org/cgi-bin/carddisp.pl?gene=CD163
MSX2	Msh Homeobox 2	Protein Coding	P35548	50	GC05P174724	2.754979	https://www.genecards.org/cgi-bin/carddisp.pl?gene=MSX2
MT2A	Metallothionein 2A	Protein Coding	P02795	46	GC16P059582	2.754466	https://www.genecards.org/cgi-bin/carddisp.pl?gene=MT2A
COL7A1	Collagen Type VII Alpha 1 Chain	Protein Coding	Q02388	47	GC03M048564	2.753627	https://www.genecards.org/cgi-bin/carddisp.pl?gene=COL7A1
SNORD116-28	Small Nucleolar RNA, C/D Box 116-28	RNA Gene		11	GC15P025104	2.752732	https://www.genecards.org/cgi-bin/carddisp.pl?gene=SNORD116-28
EGR1	Early Growth Response 1	Protein Coding	P18146	48	GC05P138465	2.750415	https://www.genecards.org/cgi-bin/carddisp.pl?gene=EGR1
SMAD3	SMAD Family Member 3	Protein Coding	P84022	57	GC15P067063	2.748444	https://www.genecards.org/cgi-bin/carddisp.pl?gene=SMAD3
RBMX	RNA Binding Motif Protein X-Linked	Protein Coding	P38159	46	GC0XM136848	2.747057	https://www.genecards.org/cgi-bin/carddisp.pl?gene=RBMX
PIGA	Phosphatidylinositol Glycan Anchor Biosynthesis Class A	Protein Coding	P37287	48	GC0XM015319	2.744098	https://www.genecards.org/cgi-bin/carddisp.pl?gene=PIGA

SNORD116-2	Small Nucleolar RNA, C/D Box 116-2	RNA Gene		14	GC15P025054	2.737436	https://www.genecards.org/cgi-bin/carddisp.pl?gene=SNORD116-2
SNORD116-25	Small Nucleolar RNA, C/D Box 116-25	RNA Gene		14	GC15P025097	2.737436	https://www.genecards.org/cgi-bin/carddisp.pl?gene=SNORD116-25
SNORD116-29	Small Nucleolar RNA, C/D Box 116-29	RNA Gene		14	GC15P025106	2.737436	https://www.genecards.org/cgi-bin/carddisp.pl?gene=SNORD116-29
SNORD116-3	Small Nucleolar RNA, C/D Box 116-3	RNA Gene		14	GC15P025056	2.737436	https://www.genecards.org/cgi-bin/carddisp.pl?gene=SNORD116-3
SNORD116-21	Small Nucleolar RNA, C/D Box 116-21	RNA Gene		13	GC15P025088	2.737436	https://www.genecards.org/cgi-bin/carddisp.pl?gene=SNORD116-21
SNORD116-23	Small Nucleolar RNA, C/D Box 116-23	RNA Gene		13	GC15P025091	2.737436	https://www.genecards.org/cgi-bin/carddisp.pl?gene=SNORD116-23
SNORD116-24	Small Nucleolar RNA, C/D Box 116-24	RNA Gene		13	GC15P025094	2.737436	https://www.genecards.org/cgi-bin/carddisp.pl?gene=SNORD116-24
SNORD116-4	Small Nucleolar RNA, C/D Box 116-4	RNA Gene		13	GC15P025059	2.737436	https://www.genecards.org/cgi-bin/carddisp.pl?gene=SNORD116-4
SNORD116-8	Small Nucleolar RNA, C/D Box 116-8	RNA Gene		13	GC15P025070	2.737436	https://www.genecards.org/cgi-bin/carddisp.pl?gene=SNORD116-8
SNORD116-9	Small Nucleolar RNA, C/D Box 116-9	RNA Gene		13	GC15P056596	2.737436	https://www.genecards.org/cgi-bin/carddisp.pl?gene=SNORD116-9
SNORD116-20	Small Nucleolar RNA, C/D Box 116-20	RNA Gene		11	GC15P025087	2.737436	https://www.genecards.org/cgi-bin/carddisp.pl?gene=SNORD116-20
SNORD116-22	Small Nucleolar RNA, C/D Box 116-22	RNA Gene		11	GC15P025089	2.737436	https://www.genecards.org/cgi-bin/carddisp.pl?gene=SNORD116-22

KMT2C	Lysine Methyltransferase 2C	Protein Coding	Q8NEZ4	47	GC07M152134	2.732868	https://www.genecards.org/cgi-bin/carddisp.pl?gene=KMT2C
FGF10	Fibroblast Growth Factor 10	Protein Coding	O15520	51	GC05M044588	2.731997	https://www.genecards.org/cgi-bin/carddisp.pl?gene=FGF10
RAB8A	RAB8A, Member RAS Oncogene Family	Protein Coding	P61006	44	GC19P016111	2.731119	https://www.genecards.org/cgi-bin/carddisp.pl?gene=RAB8A
TPP1	Tripeptidyl Peptidase 1	Protein Coding	O14773	49	GC11M009249	2.729848	https://www.genecards.org/cgi-bin/carddisp.pl?gene=TPP1
NFKBIB	NFKB Inhibitor Beta	Protein Coding	Q15653	46	GC19P038899	2.723727	https://www.genecards.org/cgi-bin/carddisp.pl?gene=NFKBIB
LOC108281177	SOX2 5' Regulatory Region	Functional Element		3	GC03P181707	2.723495	https://www.genecards.org/cgi-bin/carddisp.pl?gene=LOC108281177
CAMK1D	Calcium/Calmodulin Dependent Protein Kinase ID	Protein Coding	Q8IU85	46	GC10P012349	2.722755	https://www.genecards.org/cgi-bin/carddisp.pl?gene=CAMK1D
CRELD1	Cysteine Rich With EGF Like Domains 1	Protein Coding	Q96HD1	45	GC03P018327	2.71774	https://www.genecards.org/cgi-bin/carddisp.pl?gene=CRELD1
MIR520A	MicroRNA 520a	RNA Gene		19	GC19P053690	2.710054	https://www.genecards.org/cgi-bin/carddisp.pl?gene=MIR520A
ABCC2	ATP Binding Cassette Subfamily C Member 2	Protein Coding	Q92887	52	GC10P099782	2.708915	https://www.genecards.org/cgi-bin/carddisp.pl?gene=ABCC2
PTGS1	Prostaglandin-Endoperoxide Synthase 1	Protein Coding	P23219	51	GC09P122370	2.707976	https://www.genecards.org/cgi-bin/carddisp.pl?gene=PTGS1
SNORD116-30	Small Nucleolar RNA, C/D Box 116-30	RNA Gene		12	GC15P025108	2.704596	https://www.genecards.org/cgi-bin/carddisp.pl?gene=SNORD116-30

SNORD115-46	Small Nucleolar RNA, C/D Box 115-46	RNA Gene		10	GC15P056610	2.704596	https://www.genecards.org/cgi-bin/carddisp.pl?gene=SNORD115-46
IL12RB1	Interleukin 12 Receptor Subunit Beta 1	Protein Coding	P42701	49	GC19M018058	2.703698	https://www.genecards.org/cgi-bin/carddisp.pl?gene=IL12RB1
COL4A4	Collagen Type IV Alpha 4 Chain	Protein Coding	P53420	48	GC02M226973	2.703698	https://www.genecards.org/cgi-bin/carddisp.pl?gene=COL4A4
EMD	Emerin	Protein Coding	P50402	49	GC0XP154379	2.703373	https://www.genecards.org/cgi-bin/carddisp.pl?gene=EMD
FHIT	Fragile Histidine Triad Diadenosine Triphosphatase	Protein Coding	P49789	48	GC03M059747	2.699688	https://www.genecards.org/cgi-bin/carddisp.pl?gene=FHIT
GABRA1	Gamma-Aminobutyric Acid Type A Receptor Subunit Alpha1	Protein Coding	P14867	51	GC05P161847	2.69635	https://www.genecards.org/cgi-bin/carddisp.pl?gene=GABRA1
IL2RG	Interleukin 2 Receptor Subunit Gamma	Protein Coding	P31785	52	GC0XM071108	2.695425	https://www.genecards.org/cgi-bin/carddisp.pl?gene=IL2RG
ARID5B	AT-Rich Interaction Domain 5B	Protein Coding	Q14865	44	GC10P061901	2.686251	https://www.genecards.org/cgi-bin/carddisp.pl?gene=ARID5B
COL4A1	Collagen Type IV Alpha 1 Chain	Protein Coding	P02462	52	GC13M110148	2.685959	https://www.genecards.org/cgi-bin/carddisp.pl?gene=COL4A1
BIRC5	Baculoviral IAP Repeat Containing 5	Protein Coding	O15392	50	GC17P078214	2.684602	https://www.genecards.org/cgi-bin/carddisp.pl?gene=BIRC5
LOC110121472	VISTA Enhancer Hs1980	Functional Element		4	GC10P112997	2.684165	https://www.genecards.org/cgi-bin/carddisp.pl?gene=LOC110121472
DARS2	Aspartyl-TRNA Synthetase 2, Mitochondrial	Protein Coding	Q6PI48	46	GC01P173824	2.683794	https://www.genecards.org/cgi-bin/carddisp.pl?gene=DARS2

TOP2A	DNA Topoisomerase II Alpha	Protein Coding	P11388	54	GC17M040388	2.682608	https://www.genecards.org/cgi-bin/carddisp.pl?gene=TOP2A
MIR199A2	MicroRNA 199a-2	RNA Gene		22	GC01M172235	2.682237	https://www.genecards.org/cgi-bin/carddisp.pl?gene=MIR199A2
DNMT3B	DNA Methyltransferase 3 Beta	Protein Coding	Q9UBC3	54	GC20P032762	2.680675	https://www.genecards.org/cgi-bin/carddisp.pl?gene=DNMT3B
AB196722-001		RNA Gene		5	GC05M080892	2.679529	https://www.genecards.org/cgi-bin/carddisp.pl?gene=AB196722-001
AF420032-001		RNA Gene		5	GC11M011346	2.679529	https://www.genecards.org/cgi-bin/carddisp.pl?gene=AF420032-001
MIR92A1	MicroRNA 92a-1	RNA Gene		20	GC13P091630	2.679356	https://www.genecards.org/cgi-bin/carddisp.pl?gene=MIR92A1
MT-RNR2	Mitochondrially Encoded 16S RRNA	RNA Gene	Q8IVG9	21	GCMTP000953	2.677806	https://www.genecards.org/cgi-bin/carddisp.pl?gene=MT-RNR2
MYCN	MYCN Proto-Oncogene, BHLH Transcription Factor	Protein Coding	P04198	51	GC02P015958	2.676576	https://www.genecards.org/cgi-bin/carddisp.pl?gene=MYCN
AMER1	APC Membrane Recruitment Protein 1	Protein Coding	Q5JTC6	41	GC0XM064185	2.675558	https://www.genecards.org/cgi-bin/carddisp.pl?gene=AMER1
ANKK1	Ankyrin Repeat And Kinase Domain Containing 1	Protein Coding	Q8NFD2	41	GC11P113387	2.674443	https://www.genecards.org/cgi-bin/carddisp.pl?gene=ANKK1
MNX1	Motor Neuron And Pancreas Homeobox 1	Protein Coding	P50219	45	GC07M156994	2.673334	https://www.genecards.org/cgi-bin/carddisp.pl?gene=MNX1
PCDH15	Protocadherin Related 15	Protein Coding	Q96QU1	46	GC10M053802	2.672025	https://www.genecards.org/cgi-bin/carddisp.pl?gene=PCDH15

TRA2B	Transformer 2 Beta Homolog	Protein Coding	P62995	45	GC03M185914	2.671843	https://www.genecards.org/cgi-bin/carddisp.pl?gene=TRA2B
LINC02250	Long Intergenic Non-Protein Coding RNA 2250	RNA Gene		14	GC15M025970	2.667853	https://www.genecards.org/cgi-bin/carddisp.pl?gene=LINC02250
LOC112272578	H3K27ac HESC Enhancers GRCh37_chr15:25017418-25018314 And GRCh37_chr15:25018315-25019210	Functional Element		3	GC15P056586	2.667853	https://www.genecards.org/cgi-bin/carddisp.pl?gene=LOC112272578
LOC112272579	Sharpr-MPRA Regulatory Region 849	Functional Element		3	GC15P056587	2.667853	https://www.genecards.org/cgi-bin/carddisp.pl?gene=LOC112272579
LOC121847940	Sharpr-MPRA Regulatory Region 7323	Functional Element		2	GC15P058056	2.667853	https://www.genecards.org/cgi-bin/carddisp.pl?gene=LOC121847940
LOC125078046	Sharpr-MPRA Regulatory Region 5757	Functional Element		2	GC15P058121	2.667853	https://www.genecards.org/cgi-bin/carddisp.pl?gene=LOC125078046
LOC125078047	Sharpr-MPRA Regulatory Region 718	Functional Element		2	GC15P058122	2.667853	https://www.genecards.org/cgi-bin/carddisp.pl?gene=LOC125078047
LOC126862076	CDK7 Strongly-Dependent Group 2 Enhancer GRCh37_chr15:25789643-25790842	Functional Element		2	GC15P058678	2.667853	https://www.genecards.org/cgi-bin/carddisp.pl?gene=LOC126862076
LHX3	LIM Homeobox 3	Protein Coding	Q9UBR4	46	GC09M136196	2.667052	https://www.genecards.org/cgi-bin/carddisp.pl?gene=LHX3
FUT2	Fucosyltransferase 2	Protein Coding	Q10981	49	GC19P048695	2.666522	https://www.genecards.org/cgi-bin/carddisp.pl?gene=FUT2
ADCY5	Adenylate Cyclase 5	Protein Coding	O95622	52	GC03M123282	2.665647	https://www.genecards.org/cgi-bin/carddisp.pl?gene=ADCY5
MAPT	Microtubule Associated Protein Tau	Protein Coding	P10636	54	GC17P045894	2.662979	https://www.genecards.org/cgi-bin/carddisp.pl?gene=MAPT

ENO2	Enolase 2	Protein Coding	P09104	51	GC12P006913	2.662657	https://www.genecards.org/cgi-bin/carddisp.pl?gene=ENO2
PRKCQ	Protein Kinase C Theta	Protein Coding	Q04759	53	GC10M006742	2.662214	https://www.genecards.org/cgi-bin/carddisp.pl?gene=PRKCQ
RAB11A	RAB11A, Member RAS Oncogene Family	Protein Coding	P62491	50	GC15P137156	2.662175	https://www.genecards.org/cgi-bin/carddisp.pl?gene=RAB11A
MIR30D	MicroRNA 30d	RNA Gene		19	GC08M134804	2.66164	https://www.genecards.org/cgi-bin/carddisp.pl?gene=MIR30D
CNTNAP1	Contactin Associated Protein 1	Protein Coding	P78357	47	GC17P042682	2.6607	https://www.genecards.org/cgi-bin/carddisp.pl?gene=CNTNAP1
HSPA5	Heat Shock Protein Family A (Hsp70) Member 5	Protein Coding	P11021	53	GC09M125234	2.659027	https://www.genecards.org/cgi-bin/carddisp.pl?gene=HSPA5
TULP3	TUB Like Protein 3	Protein Coding	O75386	45	GC12P002877	2.658537	https://www.genecards.org/cgi-bin/carddisp.pl?gene=TULP3
KIR3DL1	Killer Cell Immunoglobulin Like Receptor, Three Ig Domains And Long Cytoplasmic Tail 1	Protein Coding	P43629	43	GC19P090185	2.656986	https://www.genecards.org/cgi-bin/carddisp.pl?gene=KIR3DL1
TBXT	T-Box Transcription Factor T	Protein Coding	O15178	48	GC06M166158	2.6562	https://www.genecards.org/cgi-bin/carddisp.pl?gene=TBXT
TAC3	Tachykinin Precursor 3	Protein Coding	Q9UHF0	46	GC12M058787	2.655604	https://www.genecards.org/cgi-bin/carddisp.pl?gene=TAC3
MMUT	Methylmalonyl-CoA Mutase	Protein Coding	P22033	50	GC06M049430	2.654481	https://www.genecards.org/cgi-bin/carddisp.pl?gene=MMUT
KCNJ2	Potassium Inwardly Rectifying Channel Subfamily J Member 2	Protein Coding	P63252	52	GC17P070168	2.653961	https://www.genecards.org/cgi-bin/carddisp.pl?gene=KCNJ2

HOTTIP	HOXA Distal Transcript Antisense RNA	RNA Gene		23	GC07P027198	2.652318	https://www.genecards.org/cgi-bin/carddisp.pl?gene=HOTTIP
TRIM21	Tripartite Motif Containing 21	Protein Coding	P19474	47	GC11M004384	2.652253	https://www.genecards.org/cgi-bin/carddisp.pl?gene=TRIM21
G6PC2	Glucose-6-Phosphatase Catalytic Subunit 2	Protein Coding	Q9NQR9	42	GC02P168901	2.648235	https://www.genecards.org/cgi-bin/carddisp.pl?gene=G6PC2
FLII	FLII Actin Remodeling Protein	Protein Coding	Q13045	47	GC17M018244	2.647223	https://www.genecards.org/cgi-bin/carddisp.pl?gene=FLII
PROS1	Protein S	Protein Coding	P07225	53	GC03M093873	2.646311	https://www.genecards.org/cgi-bin/carddisp.pl?gene=PROS1
CREBRF	CREB3 Regulatory Factor	Protein Coding	Q8IUR6	38	GC05P173056	2.646222	https://www.genecards.org/cgi-bin/carddisp.pl?gene=CREBRF
FGF17	Fibroblast Growth Factor 17	Protein Coding	O60258	50	GC08P022042	2.641003	https://www.genecards.org/cgi-bin/carddisp.pl?gene=FGF17
CYCS	Cytochrome C, Somatic	Protein Coding	P99999	52	GC07M025118	2.634842	https://www.genecards.org/cgi-bin/carddisp.pl?gene=CYCS
RUNX2	RUNX Family Transcription Factor 2	Protein Coding	Q13950	50	GC06P112278	2.630477	https://www.genecards.org/cgi-bin/carddisp.pl?gene=RUNX2
COX4I1	Cytochrome C Oxidase Subunit 4I1	Protein Coding	P13073	50	GC16P085798	2.6297	https://www.genecards.org/cgi-bin/carddisp.pl?gene=COX4I1
DGCR8	DGCR8 Microprocessor Complex Subunit	Protein Coding	Q8WYQ5	47	GC22P020080	2.62935	https://www.genecards.org/cgi-bin/carddisp.pl?gene=DGCR8
TP63	Tumor Protein P63	Protein Coding	Q9H3D4	51	GC03P189598	2.621742	https://www.genecards.org/cgi-bin/carddisp.pl?gene=TP63

APOC4-APOC2	APOC4-APOC2 Readthrough (NMD Candidate)	RNA Gene		19	GC19P044942	2.621206	https://www.genecards.org/cgi-bin/carddisp.pl?gene=APOC4-APOC2
RNU5A-1	RNA, U5A Small Nuclear 1	RNA Gene		17	GC15P065296	2.62092	https://www.genecards.org/cgi-bin/carddisp.pl?gene=RNU5A-1
MIR365A	MicroRNA 365a	RNA Gene		20	GC16P014309	2.617648	https://www.genecards.org/cgi-bin/carddisp.pl?gene=MIR365A
TBX6	T-Box Transcription Factor 6	Protein Coding	O95947	45	GC16M030085	2.6171	https://www.genecards.org/cgi-bin/carddisp.pl?gene=TBX6
MED13L	Mediator Complex Subunit 13L	Protein Coding	Q71F56	44	GC12M115953	2.616457	https://www.genecards.org/cgi-bin/carddisp.pl?gene=MED13L
KLK3	Kallikrein Related Peptidase 3	Protein Coding	P07288	51	GC19P050854	2.615495	https://www.genecards.org/cgi-bin/carddisp.pl?gene=KLK3
MIR490	MicroRNA 490	RNA Gene		18	GC07P136903	2.615445	https://www.genecards.org/cgi-bin/carddisp.pl?gene=MIR490
PTGER3	Prostaglandin E Receptor 3	Protein Coding	P43115	50	GC01M070852	2.614287	https://www.genecards.org/cgi-bin/carddisp.pl?gene=PTGER3
CELSR1	Cadherin EGF LAG Seven-Pass G-Type Receptor 1	Protein Coding	Q9NYQ6	45	GC22M046360	2.603378	https://www.genecards.org/cgi-bin/carddisp.pl?gene=CELSR1
COQ9	Coenzyme Q9	Protein Coding	O75208	46	GC16P057447	2.603259	https://www.genecards.org/cgi-bin/carddisp.pl?gene=COQ9
CXCL1	C-X-C Motif Chemokine Ligand 1	Protein Coding	P09341	47	GC04P073869	2.602046	https://www.genecards.org/cgi-bin/carddisp.pl?gene=CXCL1
ATXN7	Ataxin 7	Protein Coding	O15265	46	GC03P063864	2.600285	https://www.genecards.org/cgi-bin/carddisp.pl?gene=ATXN7

RMST	Rhabdomyosarcoma 2 Associated Transcript	RNA Gene		23	GC12P097431	2.596568	https://www.genecards.org/cgi-bin/carddisp.pl?gene=RMST
C12orf43	Chromosome 12 Open Reading Frame 43	Protein Coding	Q96C57	37	GC12M121000	2.59192	https://www.genecards.org/cgi-bin/carddisp.pl?gene=C12orf43
VDAC1	Voltage Dependent Anion Channel 1	Protein Coding	P21796	50	GC05M133975	2.591632	https://www.genecards.org/cgi-bin/carddisp.pl?gene=VDAC1
UFD1	Ubiquitin Recognition Factor In ER Associated Degradation 1	Protein Coding	Q92890	48	GC22M019740	2.59077	https://www.genecards.org/cgi-bin/carddisp.pl?gene=UFD1
MT-ATP8	Mitochondrially Encoded ATP Synthase Membrane Subunit 8	Protein Coding	P03928	33	GCMTP008368	2.589841	https://www.genecards.org/cgi-bin/carddisp.pl?gene=MT-ATP8
MIR376B	MicroRNA 376b	RNA Gene		15	GC14P113595	2.589057	https://www.genecards.org/cgi-bin/carddisp.pl?gene=MIR376B
CBY1	Chibby Family Member 1, Beta Catenin Antagonist	Protein Coding	Q9Y3M2	44	GC22P038656	2.588603	https://www.genecards.org/cgi-bin/carddisp.pl?gene=CBY1
DIS3L2	DIS3 Like 3'-5' Exoribonuclease 2	Protein Coding	Q8IYB7	46	GC02P231961	2.586734	https://www.genecards.org/cgi-bin/carddisp.pl?gene=DIS3L2
RASSF1	Ras Association Domain Family Member 1	Protein Coding	Q9NS23	46	GC03M050329	2.585289	https://www.genecards.org/cgi-bin/carddisp.pl?gene=RASSF1
HTR3A	5-Hydroxytryptamine Receptor 3A	Protein Coding	P46098	50	GC11P113975	2.585019	https://www.genecards.org/cgi-bin/carddisp.pl?gene=HTR3A
MIR202	MicroRNA 202	RNA Gene		19	GC10M133247	2.583514	https://www.genecards.org/cgi-bin/carddisp.pl?gene=MIR202
UBE3C	Ubiquitin Protein Ligase E3C	Protein Coding	Q15386	46	GC07P157138	2.582812	https://www.genecards.org/cgi-bin/carddisp.pl?gene=UBE3C

F10	Coagulation Factor X	Protein Coding	P00742	54	GC13P113122	2.581113	https://www.genecards.org/cgi-bin/carddisp.pl?gene=F10
BGN	Biglycan	Protein Coding	P21810	48	GC0XP153494	2.579184	https://www.genecards.org/cgi-bin/carddisp.pl?gene=BGN
HCRTR1	Hypocretin Receptor 1	Protein Coding	O43613	47	GC01P031617	2.579004	https://www.genecards.org/cgi-bin/carddisp.pl?gene=HCRTR1
COQ6	Coenzyme Q6, Monooxygenase	Protein Coding	Q9Y2Z9	47	GC14P073949	2.578998	https://www.genecards.org/cgi-bin/carddisp.pl?gene=COQ6
COQ8B	Coenzyme Q8B	Protein Coding	Q96D53	44	GC19M082558	2.578998	https://www.genecards.org/cgi-bin/carddisp.pl?gene=COQ8B
IL9	Interleukin 9	Protein Coding	P15248	47	GC05M135891	2.578692	https://www.genecards.org/cgi-bin/carddisp.pl?gene=IL9
COL6A3	Collagen Type VI Alpha 3 Chain	Protein Coding	P12111	49	GC02M237324	2.577099	https://www.genecards.org/cgi-bin/carddisp.pl?gene=COL6A3
NR1I2	Nuclear Receptor Subfamily 1 Group I Member 2	Protein Coding	O75469	47	GC03P119780	2.576993	https://www.genecards.org/cgi-bin/carddisp.pl?gene=NR1I2
CDKN1B	Cyclin Dependent Kinase Inhibitor 1B	Protein Coding	P46527	53	GC12P030709	2.576132	https://www.genecards.org/cgi-bin/carddisp.pl?gene=CDKN1B
CMKLR1	Chemerin Chemokine-Like Receptor 1	Protein Coding	Q99788	45	GC12M108288	2.573111	https://www.genecards.org/cgi-bin/carddisp.pl?gene=CMKLR1
H3C14	H3 Clustered Histone 14	Protein Coding	Q71DI3	38	GC01M157479	2.573085	https://www.genecards.org/cgi-bin/carddisp.pl?gene=H3C14
MYBPC3	Myosin Binding Protein C3	Protein Coding	Q14896	51	GC11M113170	2.572041	https://www.genecards.org/cgi-bin/carddisp.pl?gene=MYBPC3

PAPPA-AS1	PAPPA Antisense RNA 1	RNA Gene	Q5QFB9	23	GC09M116398	2.570125	https://www.genecards.org/cgi-bin/carddisp.pl?gene=PAPPA-AS1
GABRD	Gamma-Aminobutyric Acid Type A Receptor Subunit Delta	Protein Coding	O14764	50	GC01P002019	2.569866	https://www.genecards.org/cgi-bin/carddisp.pl?gene=GABRD
NCOA2	Nuclear Receptor Coactivator 2	Protein Coding	Q15596	49	GC08M070109	2.568537	https://www.genecards.org/cgi-bin/carddisp.pl?gene=NCOA2
LOC126806658	BRD4-Independent Group 4 Enhancer GRCh37_chr3:41265899-41267098	Functional Element		3	GC03P041224	2.567478	https://www.genecards.org/cgi-bin/carddisp.pl?gene=LOC126806658
MIR148B	MicroRNA 148b	RNA Gene		22	GC12P054337	2.564706	https://www.genecards.org/cgi-bin/carddisp.pl?gene=MIR148B
CEP120	Centrosomal Protein 120	Protein Coding	Q8N960	44	GC05M123344	2.560319	https://www.genecards.org/cgi-bin/carddisp.pl?gene=CEP120
MIR186	MicroRNA 186	RNA Gene		21	GC01M071067	2.557641	https://www.genecards.org/cgi-bin/carddisp.pl?gene=MIR186
CRY2	Cryptochrome Circadian Regulator 2	Protein Coding	Q49AN0	44	GC11P047009	2.550979	https://www.genecards.org/cgi-bin/carddisp.pl?gene=CRY2
SLC16A4	Solute Carrier Family 16 Member 4	Protein Coding	O15374	43	GC01M110362	2.544044	https://www.genecards.org/cgi-bin/carddisp.pl?gene=SLC16A4
MIR522	MicroRNA 522	RNA Gene		15	GC19P053751	2.543699	https://www.genecards.org/cgi-bin/carddisp.pl?gene=MIR522
CACNA1F	Calcium Voltage-Gated Channel Subunit Alpha1 F	Protein Coding	O60840	47	GC0XM049205	2.543293	https://www.genecards.org/cgi-bin/carddisp.pl?gene=CACNA1F
USP7	Ubiquitin Specific Peptidase 7	Protein Coding	Q93009	53	GC16M008892	2.541786	https://www.genecards.org/cgi-bin/carddisp.pl?gene=USP7

BSCL2	BSCL2 Lipid Droplet Biogenesis Associated, Seipin	Protein Coding	Q96G97	47	GC11M113400	2.541249	https://www.genecards.org/cgi-bin/carddisp.pl?gene=BSCL2
KLF4	KLF Transcription Factor 4	Protein Coding	O43474	50	GC09M107484	2.540645	https://www.genecards.org/cgi-bin/carddisp.pl?gene=KLF4
CYC1	Cytochrome C1	Protein Coding	P08574	49	GC08P144095	2.539528	https://www.genecards.org/cgi-bin/carddisp.pl?gene=CYC1
IL18R1	Interleukin 18 Receptor 1	Protein Coding	Q13478	47	GC02P102311	2.536338	https://www.genecards.org/cgi-bin/carddisp.pl?gene=IL18R1
IL18BP	Interleukin 18 Binding Protein	Protein Coding	O95998	45	GC11P071998	2.535685	https://www.genecards.org/cgi-bin/carddisp.pl?gene=IL18BP
TYMS	Thymidylate Synthetase	Protein Coding	P04818	54	GC18P000657	2.531569	https://www.genecards.org/cgi-bin/carddisp.pl?gene=TYMS
MIRLET7F2	MicroRNA Let-7f-2	RNA Gene		18	GC0XM053668	2.528023	https://www.genecards.org/cgi-bin/carddisp.pl?gene=MIRLET7F2
PLEC	Plectin	Protein Coding	Q15149	47	GC08M146184	2.524722	https://www.genecards.org/cgi-bin/carddisp.pl?gene=PLEC
AMY1A	Amylase Alpha 1A	Protein Coding	P0DUB6	36	GC01P103651	2.524639	https://www.genecards.org/cgi-bin/carddisp.pl?gene=AMY1A
EPRS1	Glutamyl-Prolyl-TRNA Synthetase 1	Protein Coding	P07814	48	GC01M219969	2.524069	https://www.genecards.org/cgi-bin/carddisp.pl?gene=EPRS1
SPG11	SPG11 Vesicle Trafficking Associated, Spatacsin	Protein Coding	Q96JI7	44	GC15M044562	2.519776	https://www.genecards.org/cgi-bin/carddisp.pl?gene=SPG11
PKLR	Pyruvate Kinase L/R	Protein Coding	P30613	51	GC01M155289	2.5185	https://www.genecards.org/cgi-bin/carddisp.pl?gene=PKLR

RALGAPB	Ral GTPase Activating Protein Non-Catalytic Subunit Beta	Protein Coding	Q86X10	42	GC20P038472	2.516057	https://www.genecards.org/cgi-bin/carddisp.pl?gene=RALGAPB
KLRK1	Killer Cell Lectin Like Receptor K1	Protein Coding	P26718	45	GC12M025973	2.51554	https://www.genecards.org/cgi-bin/carddisp.pl?gene=KLRK1
WNT3A	Wnt Family Member 3A	Protein Coding	P56704	52	GC01P230312	2.515339	https://www.genecards.org/cgi-bin/carddisp.pl?gene=WNT3A
CRX	Cone-Rod Homeobox	Protein Coding	O43186	46	GC19P047819	2.513404	https://www.genecards.org/cgi-bin/carddisp.pl?gene=CRX
ADGRV1	Adhesion G Protein-Coupled Receptor V1	Protein Coding	Q8WXG9	46	GC05P090529	2.511087	https://www.genecards.org/cgi-bin/carddisp.pl?gene=ADGRV1
NAP1L5	Nucleosome Assembly Protein 1 Like 5	Protein Coding	Q96NT1	40	GC04M088695	2.510669	https://www.genecards.org/cgi-bin/carddisp.pl?gene=NAP1L5
ASPM	Assembly Factor For Spindle Microtubules	Protein Coding	Q8IZT6	42	GC01M197084	2.50834	https://www.genecards.org/cgi-bin/carddisp.pl?gene=ASPM
PTPN22	Protein Tyrosine Phosphatase Non-Receptor Type 22	Protein Coding	Q9Y2R2	51	GC01M113813	2.50618	https://www.genecards.org/cgi-bin/carddisp.pl?gene=PTPN22
LOX	Lysyl Oxidase	Protein Coding	P28300	52	GC05M122063	2.505963	https://www.genecards.org/cgi-bin/carddisp.pl?gene=LOX
ELAVL1	ELAV Like RNA Binding Protein 1	Protein Coding	Q15717	45	GC19M007958	2.501512	https://www.genecards.org/cgi-bin/carddisp.pl?gene=ELAVL1
NR4A2	Nuclear Receptor Subfamily 4 Group A Member 2	Protein Coding	P43354	50	GC02M156324	2.499255	https://www.genecards.org/cgi-bin/carddisp.pl?gene=NR4A2
DIRAS3	DIRAS Family GTPase 3	Protein Coding	O95661	35	GC01M068045	2.496101	https://www.genecards.org/cgi-bin/carddisp.pl?gene=DIRAS3

RAF1	Raf-1 Proto-Oncogene, Serine/Threonine Kinase	Protein Coding	P04049	58	GC03M012583	2.495597	https://www.genecards.org/cgi-bin/carddisp.pl?gene=RAF1
KRT14	Keratin 14	Protein Coding	P02533	51	GC17M041582	2.494575	https://www.genecards.org/cgi-bin/carddisp.pl?gene=KRT14
SERAC1	Serine Active Site Containing 1	Protein Coding	Q96JX3	40	GC06M158109	2.492293	https://www.genecards.org/cgi-bin/carddisp.pl?gene=SERAC1
MYH6	Myosin Heavy Chain 6	Protein Coding	P13533	50	GC14M023381	2.492146	https://www.genecards.org/cgi-bin/carddisp.pl?gene=MYH6
TRIM28	Tripartite Motif Containing 28	Protein Coding	Q13263	49	GC19P058544	2.491405	https://www.genecards.org/cgi-bin/carddisp.pl?gene=TRIM28
TLR6	Toll Like Receptor 6	Protein Coding	Q9Y2C9	49	GC04M038828	2.490471	https://www.genecards.org/cgi-bin/carddisp.pl?gene=TLR6
HAND2	Heart And Neural Crest Derivatives Expressed 2	Protein Coding	P61296	47	GC04M173524	2.490211	https://www.genecards.org/cgi-bin/carddisp.pl?gene=HAND2
MIR539	MicroRNA 539	RNA Gene		20	GC14P113615	2.487379	https://www.genecards.org/cgi-bin/carddisp.pl?gene=MIR539
FXN	Frataxin	Protein Coding	Q16595	51	GC09P069035	2.485089	https://www.genecards.org/cgi-bin/carddisp.pl?gene=FXN
ATP2A1	ATPase Sarcoplasmic/Endoplasmic Reticulum Ca2+ Transporting 1	Protein Coding	O14983	52	GC16P053989	2.484329	https://www.genecards.org/cgi-bin/carddisp.pl?gene=ATP2A1
CHRNA3	Cholinergic Receptor Nicotinic Alpha 3 Subunit	Protein Coding	P32297	49	GC15M137225	2.483113	https://www.genecards.org/cgi-bin/carddisp.pl?gene=CHRNA3
MIR362	MicroRNA 362	RNA Gene		15	GC0XP052675	2.483113	https://www.genecards.org/cgi-bin/carddisp.pl?gene=MIR362

MGMT	O-6-Methylguanine-DNA Methyltransferase	Protein Coding	P16455	53	GC10P129467	2.480375	https://www.genecards.org/cgi-bin/carddisp.pl?gene=MGMT
TYMP	Thymidine Phosphorylase	Protein Coding	P19971	51	GC22M050525	2.480157	https://www.genecards.org/cgi-bin/carddisp.pl?gene=TYMP
ABCC1	ATP Binding Cassette Subfamily C Member 1	Protein Coding	P33527	52	GC16P015949	2.479777	https://www.genecards.org/cgi-bin/carddisp.pl?gene=ABCC1
HBE1	Hemoglobin Subunit Epsilon 1	Protein Coding	P02100	42	GC11M005268	2.478272	https://www.genecards.org/cgi-bin/carddisp.pl?gene=HBE1
SLC25A13	Solute Carrier Family 25 Member 13	Protein Coding	Q9UJS0	50	GC07M096120	2.475379	https://www.genecards.org/cgi-bin/carddisp.pl?gene=SLC25A13
NNMT	Nicotinamide N-Methyltransferase	Protein Coding	P40261	48	GC11P114257	2.47096	https://www.genecards.org/cgi-bin/carddisp.pl?gene=NNMT
DSP	Desmoplakin	Protein Coding	P15924	54	GC06P007541	2.469157	https://www.genecards.org/cgi-bin/carddisp.pl?gene=DSP
ACADS	Acyl-CoA Dehydrogenase Short Chain	Protein Coding	P16219	49	GC12P129086	2.467891	https://www.genecards.org/cgi-bin/carddisp.pl?gene=ACADS
KCNMA1	Potassium Calcium-Activated Channel Subfamily M Alpha 1	Protein Coding	Q12791	52	GC10M076869	2.465798	https://www.genecards.org/cgi-bin/carddisp.pl?gene=KCNMA1
SERPINB1	Serpin Family B Member 1	Protein Coding	P30740	45	GC06M003508	2.464442	https://www.genecards.org/cgi-bin/carddisp.pl?gene=SERPINB1
RFC1	Replication Factor C Subunit 1	Protein Coding	P35251	50	GC04M039291	2.460186	https://www.genecards.org/cgi-bin/carddisp.pl?gene=RFC1
ABCB7	ATP Binding Cassette Subfamily B Member 7	Protein Coding	O75027	48	GC0XM075053	2.459267	https://www.genecards.org/cgi-bin/carddisp.pl?gene=ABCB7

TBX10	T-Box Transcription Factor 10	Protein Coding	O75333	39	GC11M067631	2.456777	https://www.genecards.org/cgi-bin/carddisp.pl?gene=TBX10
ATP12A	ATPase H+/K+ Transporting Non-Gastric Alpha2 Subunit	Protein Coding	P54707	46	GC13P024680	2.453569	https://www.genecards.org/cgi-bin/carddisp.pl?gene=ATP12A
EIF2B2	Eukaryotic Translation Initiation Factor 2B Subunit Beta	Protein Coding	P49770	47	GC14P075002	2.452659	https://www.genecards.org/cgi-bin/carddisp.pl?gene=EIF2B2
NR4A1	Nuclear Receptor Subfamily 4 Group A Member 1	Protein Coding	P22736	51	GC12P052022	2.44867	https://www.genecards.org/cgi-bin/carddisp.pl?gene=NR4A1
GNA15	G Protein Subunit Alpha 15	Protein Coding	P30679	45	GC19P008664	2.443936	https://www.genecards.org/cgi-bin/carddisp.pl?gene=GNA15
PLPPR3	Phospholipid Phosphatase Related 3	Protein Coding	Q6T4P5	38	GC19M010246	2.443936	https://www.genecards.org/cgi-bin/carddisp.pl?gene=PLPPR3
FGF13	Fibroblast Growth Factor 13	Protein Coding	Q92913	46	GC0XM138615	2.442552	https://www.genecards.org/cgi-bin/carddisp.pl?gene=FGF13
TXNIP	Thioredoxin Interacting Protein	Protein Coding	Q9H3M7	44	GC01M145992	2.440455	https://www.genecards.org/cgi-bin/carddisp.pl?gene=TXNIP
IL22	Interleukin 22	Protein Coding	Q9GZX6	46	GC12M068248	2.440364	https://www.genecards.org/cgi-bin/carddisp.pl?gene=IL22
TRADD	TNFRSF1A Associated Via Death Domain	Protein Coding	Q15628	47	GC16M067154	2.438663	https://www.genecards.org/cgi-bin/carddisp.pl?gene=TRADD
WAC	WW Domain Containing Adaptor With Coiled-Coil	Protein Coding	Q9BTA9	43	GC10P028557	2.434736	https://www.genecards.org/cgi-bin/carddisp.pl?gene=WAC
PTGFR	Prostaglandin F Receptor	Protein Coding	P43088	48	GC01P078303	2.433113	https://www.genecards.org/cgi-bin/carddisp.pl?gene=PTGFR

MVK	Mevalonate Kinase	Protein Coding	Q03426	51	GC12P109573	2.432379	https://www.genecards.org/cgi-bin/carddisp.pl?gene=MVK
MIR758	MicroRNA 758	RNA Gene		16	GC14P113623	2.43237	https://www.genecards.org/cgi-bin/carddisp.pl?gene=MIR758
FTL	Ferritin Light Chain	Protein Coding	P02792	50	GC19P048965	2.431426	https://www.genecards.org/cgi-bin/carddisp.pl?gene=FTL
GH2	Growth Hormone 2	Protein Coding	P01242	42	GC17M063880	2.431298	https://www.genecards.org/cgi-bin/carddisp.pl?gene=GH2
SLC27A6	Solute Carrier Family 27 Member 6	Protein Coding	Q9Y2P4	47	GC05P128538	2.425576	https://www.genecards.org/cgi-bin/carddisp.pl?gene=SLC27A6
SCN4A	Sodium Voltage-Gated Channel Alpha Subunit 4	Protein Coding	P35499	50	GC17M063938	2.423874	https://www.genecards.org/cgi-bin/carddisp.pl?gene=SCN4A
PHLPP1	PH Domain And Leucine Rich Repeat Protein Phosphatase 1	Protein Coding	O60346	45	GC18P062715	2.42166	https://www.genecards.org/cgi-bin/carddisp.pl?gene=PHLPP1
ZIC3	Zic Family Member 3	Protein Coding	O60481	49	GC0XP137566	2.420676	https://www.genecards.org/cgi-bin/carddisp.pl?gene=ZIC3
TFPI2	Tissue Factor Pathway Inhibitor 2	Protein Coding	P48307	46	GC07M093885	2.420003	https://www.genecards.org/cgi-bin/carddisp.pl?gene=TFPI2
GPER1	G Protein-Coupled Estrogen Receptor 1	Protein Coding	Q99527	45	GC07P006353	2.417379	https://www.genecards.org/cgi-bin/carddisp.pl?gene=GPER1
ITGB4	Integrin Subunit Beta 4	Protein Coding	P16144	53	GC17P075721	2.41734	https://www.genecards.org/cgi-bin/carddisp.pl?gene=ITGB4
DLX5	Distal-Less Homeobox 5	Protein Coding	P56178	47	GC07M097020	2.416881	https://www.genecards.org/cgi-bin/carddisp.pl?gene=DLX5

GSTM3	Glutathione S-Transferase Mu 3	Protein Coding	P21266	50	GC01M109733	2.415394	https://www.genecards.org/cgi-bin/carddisp.pl?gene=GSTM3
SMAD2	SMAD Family Member 2	Protein Coding	Q15796	56	GC18M047809	2.413882	https://www.genecards.org/cgi-bin/carddisp.pl?gene=SMAD2
GBA1	Glucosylceramidase Beta 1	Protein Coding	P04062	53	GC01M157762	2.412581	https://www.genecards.org/cgi-bin/carddisp.pl?gene=GBA1
FOXO3	Forkhead Box O3	Protein Coding	O43524	51	GC06P108559	2.411756	https://www.genecards.org/cgi-bin/carddisp.pl?gene=FOXO3
CDH13	Cadherin 13	Protein Coding	P55290	48	GC16P082626	2.410224	https://www.genecards.org/cgi-bin/carddisp.pl?gene=CDH13
GNA11	G Protein Subunit Alpha 11	Protein Coding	P29992	51	GC19P003094	2.409479	https://www.genecards.org/cgi-bin/carddisp.pl?gene=GNA11
RXRG	Retinoid X Receptor Gamma	Protein Coding	P48443	46	GC01M165401	2.40849	https://www.genecards.org/cgi-bin/carddisp.pl?gene=RXRG
COL4A2	Collagen Type IV Alpha 2 Chain	Protein Coding	P08572	51	GC13P110305	2.407677	https://www.genecards.org/cgi-bin/carddisp.pl?gene=COL4A2
CHRNG	Cholinergic Receptor Nicotinic Gamma Subunit	Protein Coding	P07510	45	GC02P232539	2.407486	https://www.genecards.org/cgi-bin/carddisp.pl?gene=CHRNG
MIR302C	MicroRNA 302c	RNA Gene		21	GC04M112870	2.407259	https://www.genecards.org/cgi-bin/carddisp.pl?gene=MIR302C
RNASEL	Ribonuclease L	Protein Coding	Q05823	48	GC01M182573	2.405738	https://www.genecards.org/cgi-bin/carddisp.pl?gene=RNASEL
MIR515-1	MicroRNA 515-1	RNA Gene		16	GC19P053679	2.400543	https://www.genecards.org/cgi-bin/carddisp.pl?gene=MIR515-1

SF3B1	Splicing Factor 3b Subunit 1	Protein Coding	O75533	49	GC02M197453	2.399803	https://www.genecards.org/cgi-bin/carddisp.pl?gene=SF3B1
SF3B2	Splicing Factor 3b Subunit 2	Protein Coding	Q13435	46	GC11P066050	2.399803	https://www.genecards.org/cgi-bin/carddisp.pl?gene=SF3B2
MUC1	Mucin 1, Cell Surface Associated	Protein Coding	P15941	52	GC01M155185	2.396643	https://www.genecards.org/cgi-bin/carddisp.pl?gene=MUC1
MTNR1A	Melatonin Receptor 1A	Protein Coding	P48039	46	GC04M186533	2.395084	https://www.genecards.org/cgi-bin/carddisp.pl?gene=MTNR1A
KIR2DL3	Killer Cell Immunoglobulin Like Receptor, Two Ig Domains And Long Cytoplasmic Tail 3	Protein Coding	P43628	41	GC19P091072	2.393827	https://www.genecards.org/cgi-bin/carddisp.pl?gene=KIR2DL3
ASIC1	Acid Sensing Ion Channel Subunit 1	Protein Coding	P78348	49	GC12P050057	2.393438	https://www.genecards.org/cgi-bin/carddisp.pl?gene=ASIC1
CCN4	Cellular Communication Network Factor 4	Protein Coding	O95388	42	GC08P133192	2.393053	https://www.genecards.org/cgi-bin/carddisp.pl?gene=CCN4
NORAD	Non-Coding RNA Activated By DNA Damage	RNA Gene		19	GC20M036392	2.39261	https://www.genecards.org/cgi-bin/carddisp.pl?gene=NORAD
IL16	Interleukin 16	Protein Coding	Q14005	45	GC15P081159	2.392229	https://www.genecards.org/cgi-bin/carddisp.pl?gene=IL16
SLC16A1	Solute Carrier Family 16 Member 1	Protein Coding	P53985	51	GC01M113058	2.390489	https://www.genecards.org/cgi-bin/carddisp.pl?gene=SLC16A1
PFKP	Phosphofructokinase, Platelet	Protein Coding	Q01813	50	GC10P003066	2.390128	https://www.genecards.org/cgi-bin/carddisp.pl?gene=PFKP
ACAD9	Acyl-CoA Dehydrogenase Family Member 9	Protein Coding	Q9H845	49	GC03P136802	2.388851	https://www.genecards.org/cgi-bin/carddisp.pl?gene=ACAD9

IMMT	Inner Membrane Mitochondrial Protein	Protein Coding	Q16891	45	GC02M086144	2.38827	https://www.genecards.org/cgi-bin/carddisp.pl?gene=IMMT
FRAS1	Fraser Extracellular Matrix Complex Subunit 1	Protein Coding	Q86XX4	42	GC04P078057	2.387492	https://www.genecards.org/cgi-bin/carddisp.pl?gene=FRAS1
SLC9A1	Solute Carrier Family 9 Member A1	Protein Coding	P19634	54	GC01M029770	2.385445	https://www.genecards.org/cgi-bin/carddisp.pl?gene=SLC9A1
DDIT3	DNA Damage Inducible Transcript 3	Protein Coding	P35638	50	GC12M057516	2.383985	https://www.genecards.org/cgi-bin/carddisp.pl?gene=DDIT3
MDK	Midkine	Protein Coding	P21741	48	GC11P046380	2.383396	https://www.genecards.org/cgi-bin/carddisp.pl?gene=MDK
AMT	Aminomethyltransferase	Protein Coding	P48728	48	GC03M052676	2.381719	https://www.genecards.org/cgi-bin/carddisp.pl?gene=AMT
CD1D	CD1d Molecule	Protein Coding	P15813	47	GC01P158178	2.38074	https://www.genecards.org/cgi-bin/carddisp.pl?gene=CD1D
MECOM	MDS1 And EVI1 Complex Locus	Protein Coding	Q03112	52	GC03M169083	2.380692	https://www.genecards.org/cgi-bin/carddisp.pl?gene=MECOM
CTNNA3	Catenin Alpha 3	Protein Coding	Q9UI47	46	GC10M065912	2.379838	https://www.genecards.org/cgi-bin/carddisp.pl?gene=CTNNA3
GRIK2	Glutamate Ionotropic Receptor Kainate Type Subunit 2	Protein Coding	Q13002	53	GC06P100962	2.374454	https://www.genecards.org/cgi-bin/carddisp.pl?gene=GRIK2
PRDX5	Peroxiredoxin 5	Protein Coding	P30044	47	GC11P064831	2.3729	https://www.genecards.org/cgi-bin/carddisp.pl?gene=PRDX5
INVS	Inversin	Protein Coding	Q9Y283	48	GC09P100099	2.371971	https://www.genecards.org/cgi-bin/carddisp.pl?gene=INVS

VIM	Vimentin	Protein Coding	P08670	55	GC10P017227	2.37183	https://www.genecards.org/cgi-bin/carddisp.pl?gene=VIM
SOCS2	Suppressor Of Cytokine Signaling 2	Protein Coding	O14508	47	GC12P093569	2.36781	https://www.genecards.org/cgi-bin/carddisp.pl?gene=SOCS2
GCM1	Glial Cells Missing Transcription Factor 1	Protein Coding	Q9NP62	42	GC06M084377	2.366645	https://www.genecards.org/cgi-bin/carddisp.pl?gene=GCM1
EGFR-AS1	EGFR Antisense RNA 1	RNA Gene		18	GC07M055179	2.361493	https://www.genecards.org/cgi-bin/carddisp.pl?gene=EGFR-AS1
SLC25A16	Solute Carrier Family 25 Member 16	Protein Coding	P16260	41	GC10M068477	2.361386	https://www.genecards.org/cgi-bin/carddisp.pl?gene=SLC25A16
ADAR	Adenosine Deaminase RNA Specific	Protein Coding	P55265	50	GC01M157726	2.361009	https://www.genecards.org/cgi-bin/carddisp.pl?gene=ADAR
DELEC1	Deleted In Esophageal Cancer 1	RNA Gene	Q9P2X7	28	GC09P121607	2.360764	https://www.genecards.org/cgi-bin/carddisp.pl?gene=DELEC1
CCND2	Cyclin D2	Protein Coding	P30279	52	GC12P030414	2.35753	https://www.genecards.org/cgi-bin/carddisp.pl?gene=CCND2
MIR208B	MicroRNA 208b	RNA Gene		22	GC14M023417	2.357311	https://www.genecards.org/cgi-bin/carddisp.pl?gene=MIR208B
KCNJ1	Potassium Inwardly Rectifying Channel Subfamily J Member 1	Protein Coding	P48048	52	GC11M128741	2.356603	https://www.genecards.org/cgi-bin/carddisp.pl?gene=KCNJ1
RHO	Rhodopsin	Protein Coding	P08100	51	GC03P136809	2.355957	https://www.genecards.org/cgi-bin/carddisp.pl?gene=RHO
EYS	Eyes Shut Homolog	Protein Coding	Q5T1H1	38	GC06M063719	2.355957	https://www.genecards.org/cgi-bin/carddisp.pl?gene=EYS

THRB	Thyroid Hormone Receptor Beta	Protein Coding	P10828	53	GC03M024117	2.353535	https://www.genecards.org/cgi-bin/carddisp.pl?gene=THRB
ACVR1	Activin A Receptor Type 1	Protein Coding	Q04771	55	GC02M157736	2.353299	https://www.genecards.org/cgi-bin/carddisp.pl?gene=ACVR1
SIX2	SIX Homeobox 2	Protein Coding	Q9NPC8	44	GC02M045005	2.352995	https://www.genecards.org/cgi-bin/carddisp.pl?gene=SIX2
RREB1	Ras Responsive Element Binding Protein 1	Protein Coding	Q92766	46	GC06P007107	2.352684	https://www.genecards.org/cgi-bin/carddisp.pl?gene=RREB1
DROSHA	Drosha Ribonuclease III	Protein Coding	Q9NRR4	48	GC05M031401	2.35266	https://www.genecards.org/cgi-bin/carddisp.pl?gene=DROSHA
CD47	CD47 Molecule	Protein Coding	Q08722	49	GC03M108043	2.352497	https://www.genecards.org/cgi-bin/carddisp.pl?gene=CD47
CACNA1D	Calcium Voltage-Gated Channel Subunit Alpha1 D	Protein Coding	Q01668	51	GC03P053328	2.352347	https://www.genecards.org/cgi-bin/carddisp.pl?gene=CACNA1D
CA2	Carbonic Anhydrase 2	Protein Coding	P00918	55	GC08P085463	2.351467	https://www.genecards.org/cgi-bin/carddisp.pl?gene=CA2
KIR2DS4	Killer Cell Immunoglobulin Like Receptor, Two Ig Domains And Short Cytoplasmic Tail 4	Protein Coding	P43632	36	GC19P090186	2.351418	https://www.genecards.org/cgi-bin/carddisp.pl?gene=KIR2DS4
IFNGR2	Interferon Gamma Receptor 2	Protein Coding	P38484	48	GC21P033402	2.3503	https://www.genecards.org/cgi-bin/carddisp.pl?gene=IFNGR2
MIR429	MicroRNA 429	RNA Gene		21	GC01P006059	2.349648	https://www.genecards.org/cgi-bin/carddisp.pl?gene=MIR429
WNT11	Wnt Family Member 11	Protein Coding	O96014	47	GC11M076186	2.349095	https://www.genecards.org/cgi-bin/carddisp.pl?gene=WNT11

MYH7	Myosin Heavy Chain 7	Protein Coding	P12883	51	GC14M023412	2.347666	https://www.genecards.org/cgi-bin/carddisp.pl?gene=MYH7
CFC1	Cryptic, EGF-CFC Family Member 1	Protein Coding	P0CG37	42	GC02M130592	2.347633	https://www.genecards.org/cgi-bin/carddisp.pl?gene=CFC1
SLC19A3	Solute Carrier Family 19 Member 3	Protein Coding	Q9BZV2	50	GC02M227685	2.346633	https://www.genecards.org/cgi-bin/carddisp.pl?gene=SLC19A3
MIR204	MicroRNA 204	RNA Gene		23	GC09M070809	2.34593	https://www.genecards.org/cgi-bin/carddisp.pl?gene=MIR204
PCNT	Pericentrin	Protein Coding	O95613	48	GC21P046324	2.343615	https://www.genecards.org/cgi-bin/carddisp.pl?gene=PCNT
CNGB3	Cyclic Nucleotide Gated Channel Subunit Beta 3	Protein Coding	Q9NQW8	45	GC08M086553	2.340129	https://www.genecards.org/cgi-bin/carddisp.pl?gene=CNGB3
MIR517B	MicroRNA 517b	RNA Gene		17	GC19P053721	2.336801	https://www.genecards.org/cgi-bin/carddisp.pl?gene=MIR517B
SLC25A4	Solute Carrier Family 25 Member 4	Protein Coding	P12235	53	GC04P185143	2.336751	https://www.genecards.org/cgi-bin/carddisp.pl?gene=SLC25A4
PLAU	Plasminogen Activator, Urokinase	Protein Coding	P00749	55	GC10P073909	2.336498	https://www.genecards.org/cgi-bin/carddisp.pl?gene=PLAU
NRP1	Neuropilin 1	Protein Coding	O14786	52	GC10M033177	2.3342	https://www.genecards.org/cgi-bin/carddisp.pl?gene=NRP1
BCKDHB	Branched Chain Keto Acid Dehydrogenase E1 Subunit Beta	Protein Coding	P21953	47	GC06P080106	2.333465	https://www.genecards.org/cgi-bin/carddisp.pl?gene=BCKDHB
KRT7	Keratin 7	Protein Coding	P08729	46	GC12P052232	2.332564	https://www.genecards.org/cgi-bin/carddisp.pl?gene=KRT7

COL6A1	Collagen Type VI Alpha 1 Chain	Protein Coding	P12109	49	GC21P045981	2.33104	https://www.genecards.org/cgi-bin/carddisp.pl?gene=COL6A1
MIR99AHG	Mir-99a-Let-7c Cluster Host Gene	RNA Gene		23	GC21P015928	2.330673	https://www.genecards.org/cgi-bin/carddisp.pl?gene=MIR99AHG
SNORD25	Small Nucleolar RNA, C/D Box 25	RNA Gene		16	GC11M113411	2.329841	https://www.genecards.org/cgi-bin/carddisp.pl?gene=SNORD25
ATF3	Activating Transcription Factor 3	Protein Coding	P18847	48	GC01P212565	2.329595	https://www.genecards.org/cgi-bin/carddisp.pl?gene=ATF3
PAX3	Paired Box 3	Protein Coding	P23760	50	GC02M222199	2.328646	https://www.genecards.org/cgi-bin/carddisp.pl?gene=PAX3
PCAT2	Prostate Cancer Associated Transcript 2	RNA Gene		17	GC08M127072	2.328062	https://www.genecards.org/cgi-bin/carddisp.pl?gene=PCAT2
CASC19	Cancer Susceptibility 19	RNA Gene		16	GC08M134298	2.328062	https://www.genecards.org/cgi-bin/carddisp.pl?gene=CASC19
FGG	Fibrinogen Gamma Chain	Protein Coding	P02679	53	GC04M154604	2.326787	https://www.genecards.org/cgi-bin/carddisp.pl?gene=FGG
SLC22A18	Solute Carrier Family 22 Member 18	Protein Coding	Q96BI1	47	GC11P002899	2.325618	https://www.genecards.org/cgi-bin/carddisp.pl?gene=SLC22A18
MIR503	MicroRNA 503	RNA Gene		21	GC0XM134735	2.32424	https://www.genecards.org/cgi-bin/carddisp.pl?gene=MIR503
PROX1	Prospero Homeobox 1	Protein Coding	Q92786	47	GC01P213983	2.322006	https://www.genecards.org/cgi-bin/carddisp.pl?gene=PROX1
MIR301B	MicroRNA 301b	RNA Gene		17	GC22P056110	2.319376	https://www.genecards.org/cgi-bin/carddisp.pl?gene=MIR301B

HOXD13	Homeobox D13	Protein Coding	P35453	46	GC02P176092	2.319242	https://www.genecards.org/cgi-bin/carddisp.pl?gene=HOXD13
CALCRL	Calcitonin Receptor Like Receptor	Protein Coding	Q16602	50	GC02M187341	2.318561	https://www.genecards.org/cgi-bin/carddisp.pl?gene=CALCRL
DACT1	Dishevelled Binding Antagonist Of Beta Catenin 1	Protein Coding	Q9NYF0	44	GC14P058633	2.317529	https://www.genecards.org/cgi-bin/carddisp.pl?gene=DACT1
TBXAS1	Thromboxane A Synthase 1	Protein Coding	P24557	52	GC07P139777	2.316352	https://www.genecards.org/cgi-bin/carddisp.pl?gene=TBXAS1
IFNA2	Interferon Alpha 2	Protein Coding	P01563	47	GC09M021384	2.312257	https://www.genecards.org/cgi-bin/carddisp.pl?gene=IFNA2
SLC5A5	Solute Carrier Family 5 Member 5	Protein Coding	Q92911	49	GC19P089105	2.310733	https://www.genecards.org/cgi-bin/carddisp.pl?gene=SLC5A5
CELF1	CUGBP Elav-Like Family Member 1	Protein Coding	Q92879	44	GC11M113175	2.310728	https://www.genecards.org/cgi-bin/carddisp.pl?gene=CELF1
BICC1	BicC Family RNA Binding Protein 1	Protein Coding	Q9H694	42	GC10P058513	2.309669	https://www.genecards.org/cgi-bin/carddisp.pl?gene=BICC1
IL12B	Interleukin 12B	Protein Coding	P29460	49	GC05M159314	2.307605	https://www.genecards.org/cgi-bin/carddisp.pl?gene=IL12B
BRD4	Bromodomain Containing 4	Protein Coding	O60885	52	GC19M015968	2.303535	https://www.genecards.org/cgi-bin/carddisp.pl?gene=BRD4
LEF1	Lymphoid Enhancer Binding Factor 1	Protein Coding	Q9UJU2	52	GC04M108047	2.297691	https://www.genecards.org/cgi-bin/carddisp.pl?gene=LEF1
MIR486-2	MicroRNA 486-2	RNA Gene		15	GC08P041784	2.296389	https://www.genecards.org/cgi-bin/carddisp.pl?gene=MIR486-2

AGXT	Alanine--Glyoxylate Aminotransferase	Protein Coding	P21549	50	GC02P240868	2.29614	https://www.genecards.org/cgi-bin/carddisp.pl?gene=AGXT
OPA1	OPA1 Mitochondrial Dynamin Like GTPase	Protein Coding	O60313	48	GC03P193594	2.29457	https://www.genecards.org/cgi-bin/carddisp.pl?gene=OPA1
ADGRG6	Adhesion G Protein-Coupled Receptor G6	Protein Coding	Q86SQ4	47	GC06P142301	2.292923	https://www.genecards.org/cgi-bin/carddisp.pl?gene=ADGRG6
AGPAT2	1-Acylglycerol-3-Phosphate O-Acyltransferase 2	Protein Coding	O15120	48	GC09M136673	2.290933	https://www.genecards.org/cgi-bin/carddisp.pl?gene=AGPAT2
CKM	Creatine Kinase, M-Type	Protein Coding	P06732	49	GC19M045306	2.289982	https://www.genecards.org/cgi-bin/carddisp.pl?gene=CKM
HSPA1A	Heat Shock Protein Family A (Hsp70) Member 1A	Protein Coding	P0DMV8	51	GC06P112019	2.287951	https://www.genecards.org/cgi-bin/carddisp.pl?gene=HSPA1A
ARVCF	ARVCF Delta Catenin Family Member	Protein Coding	O00192	42	GC22M019966	2.287043	https://www.genecards.org/cgi-bin/carddisp.pl?gene=ARVCF
ADH5	Alcohol Dehydrogenase 5 (Class III), Chi Polypeptide	Protein Coding	P11766	50	GC04M099070	2.28358	https://www.genecards.org/cgi-bin/carddisp.pl?gene=ADH5
GNAI1	G Protein Subunit Alpha I1	Protein Coding	P63096	51	GC07P081404	2.283485	https://www.genecards.org/cgi-bin/carddisp.pl?gene=GNAI1
TSPO	Translocator Protein	Protein Coding	B1AH88	48	GC22P043151	2.281986	https://www.genecards.org/cgi-bin/carddisp.pl?gene=TSPO
BMPER	BMP Binding Endothelial Regulator	Protein Coding	Q8N8U9	45	GC07P033944	2.281195	https://www.genecards.org/cgi-bin/carddisp.pl?gene=BMPER
SULT2A1	Sulfotransferase Family 2A Member 1	Protein Coding	Q06520	48	GC19M047870	2.278516	https://www.genecards.org/cgi-bin/carddisp.pl?gene=SULT2A1

MIR518E	MicroRNA 518e	RNA Gene		18	GC19P053729	2.277836	https://www.genecards.org/cgi-bin/carddisp.pl?gene=MIR518E
MIR1-2	MicroRNA 1-2	RNA Gene		17	GC18M031069	2.277207	https://www.genecards.org/cgi-bin/carddisp.pl?gene=MIR1-2
MIR409	MicroRNA 409	RNA Gene		19	GC14P113603	2.275576	https://www.genecards.org/cgi-bin/carddisp.pl?gene=MIR409
KDM3A	Lysine Demethylase 3A	Protein Coding	Q9Y4C1	46	GC02P086440	2.273896	https://www.genecards.org/cgi-bin/carddisp.pl?gene=KDM3A
ST3GAL4	ST3 Beta-Galactoside Alpha-2,3-Sialyltransferase 4	Protein Coding	Q11206	47	GC11P126355	2.272163	https://www.genecards.org/cgi-bin/carddisp.pl?gene=ST3GAL4
PMS2	PMS1 Homolog 2, Mismatch Repair System Component	Protein Coding	P54278	53	GC07M005973	2.264832	https://www.genecards.org/cgi-bin/carddisp.pl?gene=PMS2
AFM	Afamin	Protein Coding	P43652	44	GC04P073481	2.264715	https://www.genecards.org/cgi-bin/carddisp.pl?gene=AFM
LZTS1	Leucine Zipper Tumor Suppressor 1	Protein Coding	Q9Y250	42	GC08M020246	2.264418	https://www.genecards.org/cgi-bin/carddisp.pl?gene=LZTS1
ALAD	Aminolevulinate Dehydratase	Protein Coding	P13716	50	GC09M113386	2.26403	https://www.genecards.org/cgi-bin/carddisp.pl?gene=ALAD
MTMR9	Myotubularin Related Protein 9	Protein Coding	Q96QG7	42	GC08P011284	2.260653	https://www.genecards.org/cgi-bin/carddisp.pl?gene=MTMR9
HAX1	HCLS1 Associated Protein X-1	Protein Coding	O00165	47	GC01P157038	2.260237	https://www.genecards.org/cgi-bin/carddisp.pl?gene=HAX1
HPSE	Heparanase	Protein Coding	Q9Y251	48	GC04M083292	2.258909	https://www.genecards.org/cgi-bin/carddisp.pl?gene=HPSE

BAX	BCL2 Associated X, Apoptosis Regulator	Protein Coding	Q07812	55	GC19P048954	2.258657	https://www.genecards.org/cgi-bin/carddisp.pl?gene=BAX
TRL-TAG1-1	TRNA-Leu (Anticodon TAG) 1-1	RNA Gene		11	GC17M014432	2.256273	https://www.genecards.org/cgi-bin/carddisp.pl?gene=TRL-TAG1-1
MIR187	MicroRNA 187	RNA Gene		20	GC18M035904	2.256212	https://www.genecards.org/cgi-bin/carddisp.pl?gene=MIR187
KCNK3	Potassium Two Pore Domain Channel Subfamily K Member 3	Protein Coding	O14649	53	GC02P026692	2.250509	https://www.genecards.org/cgi-bin/carddisp.pl?gene=KCNK3
IL11	Interleukin 11	Protein Coding	P20809	45	GC19M055364	2.248898	https://www.genecards.org/cgi-bin/carddisp.pl?gene=IL11
YTHDF2	YTH N6-Methyladenosine RNA Binding Protein F2	Protein Coding	Q9Y5A9	41	GC01P030384	2.248562	https://www.genecards.org/cgi-bin/carddisp.pl?gene=YTHDF2
CASC15	Cancer Susceptibility 15	RNA Gene		19	GC06P021678	2.24619	https://www.genecards.org/cgi-bin/carddisp.pl?gene=CASC15
C4A	Complement C4A (Rodgers Blood Group)	Protein Coding	P0C0L4	47	GC06P112023	2.243423	https://www.genecards.org/cgi-bin/carddisp.pl?gene=C4A
PROM1	Prominin 1	Protein Coding	O43490	51	GC04M015965	2.241728	https://www.genecards.org/cgi-bin/carddisp.pl?gene=PROM1
PADI6	Peptidyl Arginine Deiminase 6	Protein Coding	Q6TGC4	43	GC01P018740	2.241611	https://www.genecards.org/cgi-bin/carddisp.pl?gene=PADI6
FXR1	FMR1 Autosomal Homolog 1	Protein Coding	P51114	48	GC03P180868	2.241359	https://www.genecards.org/cgi-bin/carddisp.pl?gene=FXR1
RUNX1	RUNX Family Transcription Factor 1	Protein Coding	Q01196	53	GC21M034787	2.240149	https://www.genecards.org/cgi-bin/carddisp.pl?gene=RUNX1

MIR324	MicroRNA 324	RNA Gene		21	GC17M007223	2.239722	https://www.genecards.org/cgi-bin/carddisp.pl?gene=MIR324
SLC19A2	Solute Carrier Family 19 Member 2	Protein Coding	O60779	49	GC01M169463	2.238593	https://www.genecards.org/cgi-bin/carddisp.pl?gene=SLC19A2
PANK2	Pantothenate Kinase 2	Protein Coding	Q9BZ23	50	GC20P006792	2.237983	https://www.genecards.org/cgi-bin/carddisp.pl?gene=PANK2
RPGRIP1	RPGR Interacting Protein 1	Protein Coding	Q96KN7	44	GC14P040002	2.236685	https://www.genecards.org/cgi-bin/carddisp.pl?gene=RPGRIP1
MLYCD	Malonyl-CoA Decarboxylase	Protein Coding	O95822	46	GC16P083899	2.23523	https://www.genecards.org/cgi-bin/carddisp.pl?gene=MLYCD
NOS1AP	Nitric Oxide Synthase 1 Adaptor Protein	Protein Coding	O75052	44	GC01P162069	2.230442	https://www.genecards.org/cgi-bin/carddisp.pl?gene=NOS1AP
MS4A2	Membrane Spanning 4-Domains A2	Protein Coding	Q01362	45	GC11P060088	2.229549	https://www.genecards.org/cgi-bin/carddisp.pl?gene=MS4A2
MIR4497	MicroRNA 4497	RNA Gene		14	GC12P109833	2.227764	https://www.genecards.org/cgi-bin/carddisp.pl?gene=MIR4497
CXCR1	C-X-C Motif Chemokine Receptor 1	Protein Coding	P25024	47	GC02M218162	2.226656	https://www.genecards.org/cgi-bin/carddisp.pl?gene=CXCR1
LIFR	LIF Receptor Subunit Alpha	Protein Coding	P42702	51	GC05M038475	2.224959	https://www.genecards.org/cgi-bin/carddisp.pl?gene=LIFR
ELANE	Elastase, Neutrophil Expressed	Protein Coding	P08246	55	GC19P008263	2.223248	https://www.genecards.org/cgi-bin/carddisp.pl?gene=ELANE
MBD2	Methyl-CpG Binding Domain Protein 2	Protein Coding	Q9UBB5	44	GC18M054151	2.217868	https://www.genecards.org/cgi-bin/carddisp.pl?gene=MBD2

FGF4	Fibroblast Growth Factor 4	Protein Coding	P08620	48	GC11M113743	2.217742	https://www.genecards.org/cgi-bin/carddisp.pl?gene=FGF4
NTN1	Netrin 1	Protein Coding	O95631	50	GC17P014107	2.217151	https://www.genecards.org/cgi-bin/carddisp.pl?gene=NTN1
DLAT	Dihydrolipoamide S-Acetyltransferase	Protein Coding	P10515	50	GC11P112234	2.216747	https://www.genecards.org/cgi-bin/carddisp.pl?gene=DLAT
GPLD1	Glycosylphosphatidylinositol Specific Phospholipase D1	Protein Coding	P80108	46	GC06M024527	2.216683	https://www.genecards.org/cgi-bin/carddisp.pl?gene=GPLD1
LGALS17A	Galectin 14 Pseudogene	Pseudogene		14	GC19P039679	2.214757	https://www.genecards.org/cgi-bin/carddisp.pl?gene=LGALS17A
TENT5A	Terminal Nucleotidyltransferase 5A	Protein Coding	Q96IP4	40	GC06M084733	2.214616	https://www.genecards.org/cgi-bin/carddisp.pl?gene=TENT5A
CNGB1	Cyclic Nucleotide Gated Channel Subunit Beta 1	Protein Coding	Q14028	47	GC16M057884	2.212023	https://www.genecards.org/cgi-bin/carddisp.pl?gene=CNGB1
ARID1B	AT-Rich Interaction Domain 1B	Protein Coding	Q8NFD5	48	GC06P156777	2.210453	https://www.genecards.org/cgi-bin/carddisp.pl?gene=ARID1B
C11orf65	Chromosome 11 Open Reading Frame 65	Protein Coding	Q8NCR3	34	GC11M108308	2.202733	https://www.genecards.org/cgi-bin/carddisp.pl?gene=C11orf65
MMP19	Matrix Metallopeptidase 19	Protein Coding	Q99542	52	GC12M055835	2.197109	https://www.genecards.org/cgi-bin/carddisp.pl?gene=MMP19
SNAI1	Snail Family Transcriptional Repressor 1	Protein Coding	O95863	48	GC20P049982	2.196795	https://www.genecards.org/cgi-bin/carddisp.pl?gene=SNAI1
SLC10A2	Solute Carrier Family 10 Member 2	Protein Coding	Q12908	46	GC13M103043	2.192692	https://www.genecards.org/cgi-bin/carddisp.pl?gene=SLC10A2

PDGFC	Platelet Derived Growth Factor C	Protein Coding	Q9NRA1	47	GC04M156760	2.191697	https://www.genecards.org/cgi-bin/carddisp.pl?gene=PDGFC
DENND1A	DENN Domain Containing 1A	Protein Coding	Q8TEH3	42	GC09M123379	2.19039	https://www.genecards.org/cgi-bin/carddisp.pl?gene=DENND1A
ANGPTL2	Angiopoietin Like 2	Protein Coding	Q9UKU9	42	GC09M127087	2.189584	https://www.genecards.org/cgi-bin/carddisp.pl?gene=ANGPTL2
APLNR	Apelin Receptor	Protein Coding	P35414	48	GC11M057233	2.186425	https://www.genecards.org/cgi-bin/carddisp.pl?gene=APLNR
MGP	Matrix Gla Protein	Protein Coding	P08493	47	GC12M026053	2.186347	https://www.genecards.org/cgi-bin/carddisp.pl?gene=MGP
SNHG12	Small Nucleolar RNA Host Gene 12	RNA Gene	Q9BXW3	25	GC01M029853	2.183706	https://www.genecards.org/cgi-bin/carddisp.pl?gene=SNHG12
NNAT	Neuronatin	Protein Coding	Q16517	34	GC20P037521	2.181905	https://www.genecards.org/cgi-bin/carddisp.pl?gene=NNAT
HSD3B2	Hydroxy-Delta-5-Steroid Dehydrogenase, 3 Beta- And Steroid Delta-Isomerase 2	Protein Coding	P26439	49	GC01P119414	2.18122	https://www.genecards.org/cgi-bin/carddisp.pl?gene=HSD3B2
TNNT2	Troponin T2, Cardiac Type	Protein Coding	P45379	52	GC01M201359	2.179345	https://www.genecards.org/cgi-bin/carddisp.pl?gene=TNNT2
SLC8A1	Solute Carrier Family 8 Member A1	Protein Coding	P32418	48	GC02M040078	2.178754	https://www.genecards.org/cgi-bin/carddisp.pl?gene=SLC8A1
LHX4	LIM Homeobox 4	Protein Coding	Q969G2	45	GC01P180230	2.176666	https://www.genecards.org/cgi-bin/carddisp.pl?gene=LHX4
ACTN1	Actinin Alpha 1	Protein Coding	P12814	53	GC14M068874	2.176611	https://www.genecards.org/cgi-bin/carddisp.pl?gene=ACTN1

CHRND	Cholinergic Receptor Nicotinic Delta Subunit	Protein Coding	Q07001	47	GC02P232525	2.175563	https://www.genecards.org/cgi-bin/carddisp.pl?gene=CHRND
SLC7A6	Solute Carrier Family 7 Member 6	Protein Coding	Q92536	45	GC16P068821	2.174329	https://www.genecards.org/cgi-bin/carddisp.pl?gene=SLC7A6
SLC6A6	Solute Carrier Family 6 Member 6	Protein Coding	P31641	48	GC03P014402	2.174036	https://www.genecards.org/cgi-bin/carddisp.pl?gene=SLC6A6
FZD3	Frizzled Class Receptor 3	Protein Coding	Q9NPG1	47	GC08P028494	2.173064	https://www.genecards.org/cgi-bin/carddisp.pl?gene=FZD3
TFPI	Tissue Factor Pathway Inhibitor	Protein Coding	P10646	50	GC02M187464	2.172983	https://www.genecards.org/cgi-bin/carddisp.pl?gene=TFPI
SESN2	Sestrin 2	Protein Coding	P58004	44	GC01P030358	2.171775	https://www.genecards.org/cgi-bin/carddisp.pl?gene=SESN2
MCCC1	Methylcrotonyl-CoA Carboxylase Subunit 1	Protein Coding	Q96RQ3	47	GC03M183015	2.171403	https://www.genecards.org/cgi-bin/carddisp.pl?gene=MCCC1
PROC	Protein C, Inactivator Of Coagulation Factors Va And VIIIa	Protein Coding	P04070	55	GC02P127418	2.168784	https://www.genecards.org/cgi-bin/carddisp.pl?gene=PROC
PINK1	PTEN Induced Kinase 1	Protein Coding	Q9BXM7	50	GC01P020634	2.168617	https://www.genecards.org/cgi-bin/carddisp.pl?gene=PINK1
SKI	SKI Proto-Oncogene	Protein Coding	P12755	50	GC01P006117	2.168275	https://www.genecards.org/cgi-bin/carddisp.pl?gene=SKI
RAB5A	RAB5A, Member RAS Oncogene Family	Protein Coding	P20339	48	GC03P019948	2.167435	https://www.genecards.org/cgi-bin/carddisp.pl?gene=RAB5A
MIR128-1	MicroRNA 128-1	RNA Gene		20	GC02P135665	2.166601	https://www.genecards.org/cgi-bin/carddisp.pl?gene=MIR128-1

GDF11	Growth Differentiation Factor 11	Protein Coding	O95390	46	GC12P055743	2.166452	https://www.genecards.org/cgi-bin/carddisp.pl?gene=GDF11
MSH3	MutS Homolog 3	Protein Coding	P20585	47	GC05P080654	2.165534	https://www.genecards.org/cgi-bin/carddisp.pl?gene=MSH3
BCL2A1	BCL2 Related Protein A1	Protein Coding	Q16548	46	GC15M137253	2.164659	https://www.genecards.org/cgi-bin/carddisp.pl?gene=BCL2A1
PRKAG2	Protein Kinase AMP-Activated Non-Catalytic Subunit Gamma 2	Protein Coding	Q9UGJ0	53	GC07M151556	2.16455	https://www.genecards.org/cgi-bin/carddisp.pl?gene=PRKAG2
CX3CL1	C-X3-C Motif Chemokine Ligand 1	Protein Coding	P78423	48	GC16P057372	2.164116	https://www.genecards.org/cgi-bin/carddisp.pl?gene=CX3CL1
DCLRE1C	DNA Cross-Link Repair 1C	Protein Coding	Q96SD1	48	GC10M014897	2.163549	https://www.genecards.org/cgi-bin/carddisp.pl?gene=DCLRE1C
ITGA6	Integrin Subunit Alpha 6	Protein Coding	P23229	54	GC02P172427	2.162744	https://www.genecards.org/cgi-bin/carddisp.pl?gene=ITGA6
C1QTNF9	C1q And TNF Related 9	Protein Coding	P0C862	38	GC13P024307	2.162526	https://www.genecards.org/cgi-bin/carddisp.pl?gene=C1QTNF9
CCN5	Cellular Communication Network Factor 5	Protein Coding	O76076	40	GC20P044720	2.16065	https://www.genecards.org/cgi-bin/carddisp.pl?gene=CCN5
ARG2	Arginase 2	Protein Coding	P78540	50	GC14P067619	2.154437	https://www.genecards.org/cgi-bin/carddisp.pl?gene=ARG2
CCL20	C-C Motif Chemokine Ligand 20	Protein Coding	P78556	47	GC02P227846	2.154164	https://www.genecards.org/cgi-bin/carddisp.pl?gene=CCL20
TNFRSF10B	TNF Receptor Superfamily Member 10b	Protein Coding	O14763	54	GC08M023020	2.153907	https://www.genecards.org/cgi-bin/carddisp.pl?gene=TNFRSF10B

CERS1	Ceramide Synthase 1	Protein Coding	P27544	47	GC19M018868	2.151973	https://www.genecards.org/cgi-bin/carddisp.pl?gene=CERS1
ITGAV	Integrin Subunit Alpha V	Protein Coding	P06756	52	GC02P186589	2.151638	https://www.genecards.org/cgi-bin/carddisp.pl?gene=ITGAV
MFN1	Mitofusin 1	Protein Coding	Q8IWA4	46	GC03P179347	2.150753	https://www.genecards.org/cgi-bin/carddisp.pl?gene=MFN1
CGB8	Chorionic Gonadotropin Subunit Beta 8	Protein Coding	P0DN86	32	GC19M049047	2.149082	https://www.genecards.org/cgi-bin/carddisp.pl?gene=CGB8
KCNQ1-AS1	KCNQ1 Antisense RNA 1	RNA Gene		19	GC11M009034	2.147513	https://www.genecards.org/cgi-bin/carddisp.pl?gene=KCNQ1-AS1
HK1	Hexokinase 1	Protein Coding	P19367	53	GC10P069269	2.147127	https://www.genecards.org/cgi-bin/carddisp.pl?gene=HK1
OVCH1-AS1	OVCH1 Antisense RNA 1	RNA Gene		16	GC12P029389	2.145443	https://www.genecards.org/cgi-bin/carddisp.pl?gene=OVCH1-AS1
TCF7	Transcription Factor 7	Protein Coding	P36402	47	GC05P134126	2.144577	https://www.genecards.org/cgi-bin/carddisp.pl?gene=TCF7
HIF3A	Hypoxia Inducible Factor 3 Subunit Alpha	Protein Coding	Q9Y2N7	45	GC19P046297	2.144223	https://www.genecards.org/cgi-bin/carddisp.pl?gene=HIF3A
RPS6KA3	Ribosomal Protein S6 Kinase A3	Protein Coding	P51812	57	GC0XM020149	2.143307	https://www.genecards.org/cgi-bin/carddisp.pl?gene=RPS6KA3
MIR99B	MicroRNA 99b	RNA Gene		21	GC19P051692	2.138583	https://www.genecards.org/cgi-bin/carddisp.pl?gene=MIR99B
LOC106029312	Williams-Beuren Syndrome Medial Block B Recombination Region	Functional Element		4	GC07P074733	2.133978	https://www.genecards.org/cgi-bin/carddisp.pl?gene=LOC106029312

LOC106029311	Williams-Beuren Syndrome Centromeric Block B Recombination Region	Functional Element		3	GC07P081200	2.133978	https://www.genecards.org/cgi-bin/carddisp.pl?gene=LOC106029311
LOC106029313	Williams-Beuren Syndrome Telomeric Block B Recombination Region	Functional Element		3	GC07P075074	2.133978	https://www.genecards.org/cgi-bin/carddisp.pl?gene=LOC106029313
ETS1	ETS Proto-Oncogene 1, Transcription Factor	Protein Coding	P14921	51	GC11M128458	2.131501	https://www.genecards.org/cgi-bin/carddisp.pl?gene=ETS1
SNORD24	Small Nucleolar RNA, C/D Box 24	RNA Gene		19	GC09P133349	2.130899	https://www.genecards.org/cgi-bin/carddisp.pl?gene=SNORD24
CA8	Carbonic Anhydrase 8	Protein Coding	P35219	50	GC08M060187	2.129705	https://www.genecards.org/cgi-bin/carddisp.pl?gene=CA8
MIR498	MicroRNA 498	RNA Gene		16	GC19P090134	2.129135	https://www.genecards.org/cgi-bin/carddisp.pl?gene=MIR498
MADCAM1	Mucosal Vascular Addressin Cell Adhesion Molecule 1	Protein Coding	Q13477	45	GC19P008232	2.129041	https://www.genecards.org/cgi-bin/carddisp.pl?gene=MADCAM1
EMP2	Epithelial Membrane Protein 2	Protein Coding	P54851	47	GC16M013659	2.128703	https://www.genecards.org/cgi-bin/carddisp.pl?gene=EMP2
TGM2	Transglutaminase 2	Protein Coding	P21980	51	GC20M038127	2.128202	https://www.genecards.org/cgi-bin/carddisp.pl?gene=TGM2
PCSK5	Proprotein Convertase Subtilisin/Kexin Type 5	Protein Coding	Q92824	46	GC09P075890	2.128149	https://www.genecards.org/cgi-bin/carddisp.pl?gene=PCSK5
PFKFB3	6-Phosphofructo-2-Kinase/Fructose-2,6-Biphosphatase 3	Protein Coding	Q16875	49	GC10P006144	2.126616	https://www.genecards.org/cgi-bin/carddisp.pl?gene=PFKFB3
LRP1B	LDL Receptor Related Protein 1B	Protein Coding	Q9NZR2	44	GC02M140231	2.124079	https://www.genecards.org/cgi-bin/carddisp.pl?gene=LRP1B

WHRN	Whirlin	Protein Coding	Q9P202	41	GC09M116819	2.123293	https://www.genecards.org/cgi-bin/carddisp.pl?gene=WHRN
DNAJC12	DnaJ Heat Shock Protein Family (Hsp40) Member C12	Protein Coding	Q9UKB3	43	GC10M067796	2.120658	https://www.genecards.org/cgi-bin/carddisp.pl?gene=DNAJC12
GALK1	Galactokinase 1	Protein Coding	P51570	52	GC17M075751	2.120326	https://www.genecards.org/cgi-bin/carddisp.pl?gene=GALK1
TAC1	Tachykinin Precursor 1	Protein Coding	P20366	45	GC07P097734	2.120248	https://www.genecards.org/cgi-bin/carddisp.pl?gene=TAC1
UGT2B7	UDP Glucuronosyltransferase Family 2 Member B7	Protein Coding	P16662	47	GC04P069051	2.119209	https://www.genecards.org/cgi-bin/carddisp.pl?gene=UGT2B7
TTF2	Transcription Termination Factor 2	Protein Coding	Q9UNY4	44	GC01P117060	2.117514	https://www.genecards.org/cgi-bin/carddisp.pl?gene=TTF2
ITGA1	Integrin Subunit Alpha 1	Protein Coding	P56199	48	GC05P052788	2.116215	https://www.genecards.org/cgi-bin/carddisp.pl?gene=ITGA1
POSTN	Periostin	Protein Coding	Q15063	47	GC13M037562	2.115251	https://www.genecards.org/cgi-bin/carddisp.pl?gene=POSTN
OSM	Oncostatin M	Protein Coding	P13725	47	GC22M030262	2.113422	https://www.genecards.org/cgi-bin/carddisp.pl?gene=OSM
HEIH	Hepatocellular Carcinoma Up-Regulated EZH2-Associated Long Non-Coding RNA	RNA Gene		16	GC05M182290	2.110108	https://www.genecards.org/cgi-bin/carddisp.pl?gene=HEIH
HSPA4	Heat Shock Protein Family A (Hsp70) Member 4	Protein Coding	P34932	47	GC05P133062	2.109349	https://www.genecards.org/cgi-bin/carddisp.pl?gene=HSPA4
KAT2B	Lysine Acetyltransferase 2B	Protein Coding	Q92831	52	GC03P020047	2.10894	https://www.genecards.org/cgi-bin/carddisp.pl?gene=KAT2B

MIR516B1	MicroRNA 516b-1	RNA Gene		17	GC19P053736	2.10802	https://www.genecards.org/cgi-bin/carddisp.pl?gene=MIR516B1
MIR516B2	MicroRNA 516b-2	RNA Gene		16	GC19P053725	2.10802	https://www.genecards.org/cgi-bin/carddisp.pl?gene=MIR516B2
CHIT1	Chitinase 1	Protein Coding	Q13231	47	GC01M203213	2.106245	https://www.genecards.org/cgi-bin/carddisp.pl?gene=CHIT1
TRPM6	Transient Receptor Potential Cation Channel Subfamily M Member 6	Protein Coding	Q9BX84	49	GC09M074725	2.104128	https://www.genecards.org/cgi-bin/carddisp.pl?gene=TRPM6
GAS6	Growth Arrest Specific 6	Protein Coding	Q14393	48	GC13M113820	2.102614	https://www.genecards.org/cgi-bin/carddisp.pl?gene=GAS6
DEFB1	Defensin Beta 1	Protein Coding	P60022	43	GC08M006870	2.102056	https://www.genecards.org/cgi-bin/carddisp.pl?gene=DEFB1
S100A8	S100 Calcium Binding Protein A8	Protein Coding	P05109	46	GC01M157633	2.10192	https://www.genecards.org/cgi-bin/carddisp.pl?gene=S100A8
GATA6	GATA Binding Protein 6	Protein Coding	Q92908	51	GC18P022169	2.101799	https://www.genecards.org/cgi-bin/carddisp.pl?gene=GATA6
CCN2	Cellular Communication Network Factor 2	Protein Coding	P29279	51	GC06M131948	2.10017	https://www.genecards.org/cgi-bin/carddisp.pl?gene=CCN2
MIR520G	MicroRNA 520g	RNA Gene		18	GC19P053722	2.098353	https://www.genecards.org/cgi-bin/carddisp.pl?gene=MIR520G
NOD1	Nucleotide Binding Oligomerization Domain Containing 1	Protein Coding	Q9Y239	47	GC07M030424	2.097856	https://www.genecards.org/cgi-bin/carddisp.pl?gene=NOD1
EHMT2	Euchromatic Histone Lysine Methyltransferase 2	Protein Coding	Q96KQ7	51	GC06M031879	2.095365	https://www.genecards.org/cgi-bin/carddisp.pl?gene=EHMT2

PLEK	Pleckstrin	Protein Coding	P08567	45	GC02P068365	2.094728	https://www.genecards.org/cgi-bin/carddisp.pl?gene=PLEK
MARS1	Methionyl-TRNA Synthetase 1	Protein Coding	P56192	49	GC12P058690	2.093683	https://www.genecards.org/cgi-bin/carddisp.pl?gene=MARS1
EIF2AK3	Eukaryotic Translation Initiation Factor 2 Alpha Kinase 3	Protein Coding	Q9NZJ5	53	GC02M088556	2.092807	https://www.genecards.org/cgi-bin/carddisp.pl?gene=EIF2AK3
BICD2	BICD Cargo Adaptor 2	Protein Coding	Q8TD16	45	GC09M092711	2.091465	https://www.genecards.org/cgi-bin/carddisp.pl?gene=BICD2
CRAT	Carnitine O-Acetyltransferase	Protein Coding	P43155	49	GC09M129094	2.09055	https://www.genecards.org/cgi-bin/carddisp.pl?gene=CRAT
SORL1	Sortilin Related Receptor 1	Protein Coding	Q92673	50	GC11P121452	2.090367	https://www.genecards.org/cgi-bin/carddisp.pl?gene=SORL1
MIR582	MicroRNA 582	RNA Gene		20	GC05M059703	2.090332	https://www.genecards.org/cgi-bin/carddisp.pl?gene=MIR582
ZMPSTE24	Zinc Metallopeptidase STE24	Protein Coding	O75844	45	GC01P040258	2.089258	https://www.genecards.org/cgi-bin/carddisp.pl?gene=ZMPSTE24
UQCRB	Ubiquinol-Cytochrome C Reductase Binding Protein	Protein Coding	P14927	47	GC08M096225	2.088718	https://www.genecards.org/cgi-bin/carddisp.pl?gene=UQCRB
UGT1A7	UDP Glucuronosyltransferase Family 1 Member A7	Protein Coding	Q9HAW7	44	GC02P233681	2.088209	https://www.genecards.org/cgi-bin/carddisp.pl?gene=UGT1A7
HLA-DRB4	Major Histocompatibility Complex, Class II, DR Beta 4	Protein Coding	P13762	32	GC06Mo03851	2.083786	https://www.genecards.org/cgi-bin/carddisp.pl?gene=HLA-DRB4
GATA2	GATA Binding Protein 2	Protein Coding	P23769	53	GC03M128479	2.083227	https://www.genecards.org/cgi-bin/carddisp.pl?gene=GATA2

SRF	Serum Response Factor	Protein Coding	P11831	47	GC06P043171	2.082628	https://www.genecards.org/cgi-bin/carddisp.pl?gene=SRF
ANXA1	Annexin A1	Protein Coding	P04083	51	GC09P073151	2.082523	https://www.genecards.org/cgi-bin/carddisp.pl?gene=ANXA1
ZNF365	Zinc Finger Protein 365	Protein Coding	Q70YC4	42	GC10P062374	2.082351	https://www.genecards.org/cgi-bin/carddisp.pl?gene=ZNF365
SORT1	Sortilin 1	Protein Coding	Q99523	48	GC01M109310	2.080841	https://www.genecards.org/cgi-bin/carddisp.pl?gene=SORT1
CACNA1C	Calcium Voltage-Gated Channel Subunit Alpha1 C	Protein Coding	Q13936	53	GC12P001970	2.080603	https://www.genecards.org/cgi-bin/carddisp.pl?gene=CACNA1C
CD80	CD80 Molecule	Protein Coding	P33681	47	GC03M119524	2.07519	https://www.genecards.org/cgi-bin/carddisp.pl?gene=CD80
PTF1A	Pancreas Associated Transcription Factor 1a	Protein Coding	Q7RTS3	46	GC10P023194	2.074309	https://www.genecards.org/cgi-bin/carddisp.pl?gene=PTF1A
RNY5	RNA, Ro60-Associated Y5	RNA Gene		13	GC07P148941	2.071983	https://www.genecards.org/cgi-bin/carddisp.pl?gene=RNY5
FGF7	Fibroblast Growth Factor 7	Protein Coding	P21781	47	GC15P049423	2.071629	https://www.genecards.org/cgi-bin/carddisp.pl?gene=FGF7
HSPD1	Heat Shock Protein Family D (Hsp60) Member 1	Protein Coding	P10809	53	GC02M197486	2.071622	https://www.genecards.org/cgi-bin/carddisp.pl?gene=HSPD1
SLC8A1-AS1	SLC8A1 Antisense RNA 1	RNA Gene		16	GC02P039752	2.069823	https://www.genecards.org/cgi-bin/carddisp.pl?gene=SLC8A1-AS1
COL4A3	Collagen Type IV Alpha 3 Chain	Protein Coding	Q01955	50	GC02P227164	2.064734	https://www.genecards.org/cgi-bin/carddisp.pl?gene=COL4A3

CHD8	Chromodomain Helicase DNA Binding Protein 8	Protein Coding	Q9HCK8	46	GC14M021385	2.064522	https://www.genecards.org/cgi-bin/carddisp.pl?gene=CHD8
IL10RB	Interleukin 10 Receptor Subunit Beta	Protein Coding	Q08334	49	GC21P033266	2.063934	https://www.genecards.org/cgi-bin/carddisp.pl?gene=IL10RB
PTGES	Prostaglandin E Synthase	Protein Coding	O14684	46	GC09M129738	2.06244	https://www.genecards.org/cgi-bin/carddisp.pl?gene=PTGES
CITED2	Cbp/P300 Interacting Transactivator With Glu/Asp Rich Carboxy-Terminal Domain 2	Protein Coding	Q99967	47	GC06M139371	2.061216	https://www.genecards.org/cgi-bin/carddisp.pl?gene=CITED2
NBAT1	Neuroblastoma Associated Transcript 1	RNA Gene		18	GC06M022275	2.060432	https://www.genecards.org/cgi-bin/carddisp.pl?gene=NBAT1
EVC	EvC Ciliary Complex Subunit 1	Protein Coding	P57679	42	GC04P005712	2.056038	https://www.genecards.org/cgi-bin/carddisp.pl?gene=EVC
GRIN1	Glutamate Ionotropic Receptor NMDA Type Subunit 1	Protein Coding	Q05586	54	GC09P137138	2.055031	https://www.genecards.org/cgi-bin/carddisp.pl?gene=GRIN1
ACAN	Aggrecan	Protein Coding	P16112	51	GC15P137698	2.052665	https://www.genecards.org/cgi-bin/carddisp.pl?gene=ACAN
HRH2	Histamine Receptor H2	Protein Coding	P25021	47	GC05P175659	2.052357	https://www.genecards.org/cgi-bin/carddisp.pl?gene=HRH2
FCGR3A	Fc Gamma Receptor IIIa	Protein Coding	P08637	51	GC01M161541	2.052125	https://www.genecards.org/cgi-bin/carddisp.pl?gene=FCGR3A
EIF2S2	Eukaryotic Translation Initiation Factor 2 Subunit Beta	Protein Coding	P20042	45	GC20M034088	2.051859	https://www.genecards.org/cgi-bin/carddisp.pl?gene=EIF2S2
NSUN2	NOP2/Sun RNA Methyltransferase 2	Protein Coding	Q08J23	47	GC05M006599	2.050871	https://www.genecards.org/cgi-bin/carddisp.pl?gene=NSUN2

CEACAM1	CEA Cell Adhesion Molecule 1	Protein Coding	P13688	49	GC19M042507	2.049662	https://www.genecards.org/cgi-bin/carddisp.pl?gene=CEACAM1
MYH11	Myosin Heavy Chain 11	Protein Coding	P35749	50	GC16M016798	2.049639	https://www.genecards.org/cgi-bin/carddisp.pl?gene=MYH11
MIR493	MicroRNA 493	RNA Gene		20	GC14P113610	2.049367	https://www.genecards.org/cgi-bin/carddisp.pl?gene=MIR493
PDZD2	PDZ Domain Containing 2	Protein Coding	O15018	41	GC05P031639	2.046149	https://www.genecards.org/cgi-bin/carddisp.pl?gene=PDZD2
CD151	CD151 Molecule (Raph Blood Group)	Protein Coding	P48509	48	GC11P004480	2.045925	https://www.genecards.org/cgi-bin/carddisp.pl?gene=CD151
FOXP1	Forkhead Box P1	Protein Coding	Q9H334	48	GC03M070954	2.045591	https://www.genecards.org/cgi-bin/carddisp.pl?gene=FOXP1
RORA	RAR Related Orphan Receptor A	Protein Coding	P35398	52	GC15M060488	2.044795	https://www.genecards.org/cgi-bin/carddisp.pl?gene=RORA
RNU4ATAC	RNA, U4atac Small Nuclear	RNA Gene		21	GC02P127225	2.044635	https://www.genecards.org/cgi-bin/carddisp.pl?gene=RNU4ATAC
JUP	Junction Plakoglobin	Protein Coding	P14923	53	GC17M041754	2.042118	https://www.genecards.org/cgi-bin/carddisp.pl?gene=JUP
WNK4	WNK Lysine Deficient Protein Kinase 4	Protein Coding	Q96J92	48	GC17P042780	2.041026	https://www.genecards.org/cgi-bin/carddisp.pl?gene=WNK4
MYOC	Myocilin	Protein Coding	Q99972	46	GC01M171604	2.039898	https://www.genecards.org/cgi-bin/carddisp.pl?gene=MYOC
OLFML3	Olfactomedin Like 3	Protein Coding	Q9NRN5	42	GC01P113979	2.038652	https://www.genecards.org/cgi-bin/carddisp.pl?gene=OLFML3

RICTOR	RPTOR Independent Companion Of MTOR Complex 2	Protein Coding	Q6R327	48	GC05M038937	2.038505	https://www.genecards.org/cgi-bin/carddisp.pl?gene=RICTOR
WNK1	WNK Lysine Deficient Protein Kinase 1	Protein Coding	Q9H4A3	52	GC12P000733	2.038279	https://www.genecards.org/cgi-bin/carddisp.pl?gene=WNK1
ETFA	Electron Transfer Flavoprotein Subunit Alpha	Protein Coding	P13804	50	GC15M137172	2.038038	https://www.genecards.org/cgi-bin/carddisp.pl?gene=ETFA
GDF1	Growth Differentiation Factor 1	Protein Coding	P27539	42	GC19M018843	2.03789	https://www.genecards.org/cgi-bin/carddisp.pl?gene=GDF1
EIF2B5	Eukaryotic Translation Initiation Factor 2B Subunit Epsilon	Protein Coding	Q13144	45	GC03P184135	2.035471	https://www.genecards.org/cgi-bin/carddisp.pl?gene=EIF2B5
CYP26A1	Cytochrome P450 Family 26 Subfamily A Member 1	Protein Coding	O43174	47	GC10P093073	2.035345	https://www.genecards.org/cgi-bin/carddisp.pl?gene=CYP26A1
COX14	Cytochrome C Oxidase Assembly Factor COX14	Protein Coding	Q96I36	41	GC12P050806	2.030417	https://www.genecards.org/cgi-bin/carddisp.pl?gene=COX14
MIR487A	MicroRNA 487a	RNA Gene		16	GC14P113608	2.030223	https://www.genecards.org/cgi-bin/carddisp.pl?gene=MIR487A
ZEB2-AS1	ZEB2 Antisense RNA 1	RNA Gene		21	GC02P144519	2.028069	https://www.genecards.org/cgi-bin/carddisp.pl?gene=ZEB2-AS1
MIR154	MicroRNA 154	RNA Gene		20	GC14P113583	2.028069	https://www.genecards.org/cgi-bin/carddisp.pl?gene=MIR154
SLC27A3	Solute Carrier Family 27 Member 3	Protein Coding	Q5K4L6	44	GC01P157011	2.027713	https://www.genecards.org/cgi-bin/carddisp.pl?gene=SLC27A3
CXCL2	C-X-C Motif Chemokine Ligand 2	Protein Coding	P19875	44	GC04M074097	2.02711	https://www.genecards.org/cgi-bin/carddisp.pl?gene=CXCL2

LOC126862536	BRD4-Independent Group 4 Enhancer GRCh37_chr17:32578883-32580082	Functional Element		3	GC17P088391	2.02566	https://www.genecards.org/cgi-bin/carddisp.pl?gene=LOC126862536
TCOF1	Treacle Ribosome Biogenesis Factor 1	Protein Coding	Q13428	46	GC05P150358	2.025321	https://www.genecards.org/cgi-bin/carddisp.pl?gene=TCOF1
PARP1	Poly(ADP-Ribose) Polymerase 1	Protein Coding	P09874	54	GC01M226360	2.023302	https://www.genecards.org/cgi-bin/carddisp.pl?gene=PARP1
LINC01094	Long Intergenic Non-Protein Coding RNA 1094	RNA Gene		16	GC04P078838	2.021797	https://www.genecards.org/cgi-bin/carddisp.pl?gene=LINC01094
ATP1A1	ATPase Na+/K+ Transporting Subunit Alpha 1	Protein Coding	P05023	54	GC01P116372	2.021325	https://www.genecards.org/cgi-bin/carddisp.pl?gene=ATP1A1
ADCY9	Adenylate Cyclase 9	Protein Coding	O60503	48	GC16M003953	2.019517	https://www.genecards.org/cgi-bin/carddisp.pl?gene=ADCY9
C5	Complement C5	Protein Coding	P01031	51	GC09M120933	2.019436	https://www.genecards.org/cgi-bin/carddisp.pl?gene=C5
RNY3	RNA, Ro60-Associated Y3	RNA Gene		16	GC07P153440	2.01907	https://www.genecards.org/cgi-bin/carddisp.pl?gene=RNY3
SELENOP	Selenoprotein P	Protein Coding	P49908	40	GC05M042800	2.018815	https://www.genecards.org/cgi-bin/carddisp.pl?gene=SELENOP
PRKCD	Protein Kinase C Delta	Protein Coding	Q05655	55	GC03P053156	2.017985	https://www.genecards.org/cgi-bin/carddisp.pl?gene=PRKCD
PLCG1	Phospholipase C Gamma 1	Protein Coding	P19174	52	GC20P041136	2.017306	https://www.genecards.org/cgi-bin/carddisp.pl?gene=PLCG1
FDX1	Ferredoxin 1	Protein Coding	P10109	45	GC11P110429	2.016309	https://www.genecards.org/cgi-bin/carddisp.pl?gene=FDX1

NOG	Noggin	Protein Coding	Q13253	51	GC17P056593	2.0151	https://www.genecards.org/cgi-bin/carddisp.pl?gene=NOG
RIPK3	Receptor Interacting Serine/Threonine Kinase 3	Protein Coding	Q9Y572	48	GC14M024336	2.01453	https://www.genecards.org/cgi-bin/carddisp.pl?gene=RIPK3
MCCC2	Methylcrotonyl-CoA Carboxylase Subunit 2	Protein Coding	Q9HCC0	48	GC05P075054	2.012173	https://www.genecards.org/cgi-bin/carddisp.pl?gene=MCCC2
HTRA1	HtrA Serine Peptidase 1	Protein Coding	Q92743	49	GC10P122461	2.011023	https://www.genecards.org/cgi-bin/carddisp.pl?gene=HTRA1
EXT1	Exostosin Glycosyltransferase 1	Protein Coding	Q16394	51	GC08M117798	2.008335	https://www.genecards.org/cgi-bin/carddisp.pl?gene=EXT1
CD86	CD86 Molecule	Protein Coding	P42081	48	GC03P122055	2.007606	https://www.genecards.org/cgi-bin/carddisp.pl?gene=CD86
MMP11	Matrix Metallopeptidase 11	Protein Coding	P24347	48	GC22P023768	2.006342	https://www.genecards.org/cgi-bin/carddisp.pl?gene=MMP11
SIX6	SIX Homeobox 6	Protein Coding	O95475	46	GC14P060508	2.005088	https://www.genecards.org/cgi-bin/carddisp.pl?gene=SIX6
CDC25C	Cell Division Cycle 25C	Protein Coding	P30307	51	GC05M138285	2.004119	https://www.genecards.org/cgi-bin/carddisp.pl?gene=CDC25C
TRN-GTT2-5	TRNA-Asn (Anticodon GTT) 2-5	RNA Gene		9	GC17M038751	2.00256	https://www.genecards.org/cgi-bin/carddisp.pl?gene=TRN-GTT2-5
TRN-GTT2-6	TRNA-Asn (Anticodon GTT) 2-6	RNA Gene		9	GC19P008536	2.00256	https://www.genecards.org/cgi-bin/carddisp.pl?gene=TRN-GTT2-6
TRN-GTT2-1	TRNA-Asn (Anticodon GTT) 2-1	RNA Gene		8	GC01P156625	2.00256	https://www.genecards.org/cgi-bin/carddisp.pl?gene=TRN-GTT2-1

TRN-GTT2-3	TRNA-Asn (Anticodon GTT) 2-3	RNA Gene		8	GC10M022229	2.00256	https://www.genecards.org/cgi-bin/carddisp.pl?gene=TRN-GTT2-3
TRN-GTT2-2	TRNA-Asn (Anticodon GTT) 2-2	RNA Gene		7	GC01M161428	2.00256	https://www.genecards.org/cgi-bin/carddisp.pl?gene=TRN-GTT2-2
TRN-GTT2-4	TRNA-Asn (Anticodon GTT) 2-4	RNA Gene		7	GC13M030673	2.00256	https://www.genecards.org/cgi-bin/carddisp.pl?gene=TRN-GTT2-4
TRN-GTT2-7	TRNA-Asn (Anticodon GTT) 2-7	RNA Gene		7	GC01P158038	2.00256	https://www.genecards.org/cgi-bin/carddisp.pl?gene=TRN-GTT2-7
TRN-GTT2-8	TRNA-Asn (Anticodon GTT) 2-8	RNA Gene		5	GC01M157107	2.00256	https://www.genecards.org/cgi-bin/carddisp.pl?gene=TRN-GTT2-8
DIP2C-AS1	DIP2C Antisense RNA 1	RNA Gene	Q8N8Z3	27	GC10P000651	2.000277	https://www.genecards.org/cgi-bin/carddisp.pl?gene=DIP2C-AS1
MAP3K5	Mitogen-Activated Protein Kinase Kinase Kinase 5	Protein Coding	Q99683	52	GC06M136557	1.997257	https://www.genecards.org/cgi-bin/carddisp.pl?gene=MAP3K5
FGF5	Fibroblast Growth Factor 5	Protein Coding	P12034	48	GC04P080266	1.997206	https://www.genecards.org/cgi-bin/carddisp.pl?gene=FGF5
HADH	Hydroxyacyl-CoA Dehydrogenase	Protein Coding	Q16836	51	GC04P107989	1.995828	https://www.genecards.org/cgi-bin/carddisp.pl?gene=HADH
PAX7	Paired Box 7	Protein Coding	P23759	47	GC01P018765	1.993511	https://www.genecards.org/cgi-bin/carddisp.pl?gene=PAX7
TRU-TCA1-1	TRNA-SeC (Anticodon TCA) 1-1	RNA Gene		12	GC19M045478	1.992316	https://www.genecards.org/cgi-bin/carddisp.pl?gene=TRU-TCA1-1
NCAM1	Neural Cell Adhesion Molecule 1	Protein Coding	P13591	52	GC11P112961	1.98445	https://www.genecards.org/cgi-bin/carddisp.pl?gene=NCAM1

CTSB	Cathepsin B	Protein Coding	P07858	55	GC08M011842	1.983609	https://www.genecards.org/cgi-bin/carddisp.pl?gene=CTSB
IRF1	Interferon Regulatory Factor 1	Protein Coding	P10914	50	GC05M132440	1.982788	https://www.genecards.org/cgi-bin/carddisp.pl?gene=IRF1
MIR654	MicroRNA 654	RNA Gene		19	GC14P113618	1.980971	https://www.genecards.org/cgi-bin/carddisp.pl?gene=MIR654
MIR1268A	MicroRNA 1268a	RNA Gene		11	GC15M022225	1.980851	https://www.genecards.org/cgi-bin/carddisp.pl?gene=MIR1268A
PCAT29	Prostate Cancer Associated Transcript 29	RNA Gene		17	GC15P140105	1.978464	https://www.genecards.org/cgi-bin/carddisp.pl?gene=PCAT29
DRAIC	Downregulated RNA In Cancer, Inhibitor Of Cell Invasion And Migration	RNA Gene		16	GC15P137167	1.978464	https://www.genecards.org/cgi-bin/carddisp.pl?gene=DRAIC
CCAT2	Colon Cancer Associated Transcript 2	RNA Gene		14	GC08P127400	1.978464	https://www.genecards.org/cgi-bin/carddisp.pl?gene=CCAT2
GPT2	Glutamic--Pyruvic Transaminase 2	Protein Coding	Q8TD30	49	GC16P054544	1.977942	https://www.genecards.org/cgi-bin/carddisp.pl?gene=GPT2
CYP3A7	Cytochrome P450 Family 3 Subfamily A Member 7	Protein Coding	P24462	44	GC07M099705	1.975006	https://www.genecards.org/cgi-bin/carddisp.pl?gene=CYP3A7
ADCY10	Adenylate Cyclase 10	Protein Coding	Q96PN6	48	GC01M167809	1.974002	https://www.genecards.org/cgi-bin/carddisp.pl?gene=ADCY10
ALOX15	Arachidonate 15-Lipoxygenase	Protein Coding	P16050	50	GC17M004630	1.971026	https://www.genecards.org/cgi-bin/carddisp.pl?gene=ALOX15
C1QTNF1	C1q And TNF Related 1	Protein Coding	Q9BXJ1	44	GC17P079022	1.970196	https://www.genecards.org/cgi-bin/carddisp.pl?gene=C1QTNF1

MT-TC	Mitochondrially Encoded TRNA-Cys (UGU/C)	RNA Gene		12	GCMTM0057 63	1.96993 9	https://www.genecards.org/cgi-bin/carddisp.pl?gene=MT-TC
DCAF8	DDB1 And CUL4 Associated Factor 8	Protein Coding	Q5TAQ9	42	GC01M16021 5	1.96938 5	https://www.genecards.org/cgi-bin/carddisp.pl?gene=DCAF8
IL1RAPL2	Interleukin 1 Receptor Accessory Protein Like 2	Protein Coding	Q9NP60	43	GC0XP10456 6	1.96898 6	https://www.genecards.org/cgi-bin/carddisp.pl?gene=IL1RAPL2
NCR3	Natural Cytotoxicity Triggering Receptor 3	Protein Coding	O14931	44	GC06M03158 8	1.96889 5	https://www.genecards.org/cgi-bin/carddisp.pl?gene=NCR3
SLC26A2	Solute Carrier Family 26 Member 2	Protein Coding	P50443	48	GC05P149944	1.96882	https://www.genecards.org/cgi-bin/carddisp.pl?gene=SLC26A2
MIR425	MicroRNA 425	RNA Gene		20	GC03M05265 2	1.96876 5	https://www.genecards.org/cgi-bin/carddisp.pl?gene=MIR425
SOX10	SRY-Box Transcription Factor 10	Protein Coding	P56693	50	GC22M07214 1	1.96869 5	https://www.genecards.org/cgi-bin/carddisp.pl?gene=SOX10
ST3GAL6-AS1	ST3GAL6 Antisense RNA 1	RNA Gene		17	GC03M09871 4	1.96731 2	https://www.genecards.org/cgi-bin/carddisp.pl?gene=ST3GAL6-AS1
LGALS4	Galectin 4	Protein Coding	P56470	42	GC19M08252 7	1.96464 5	https://www.genecards.org/cgi-bin/carddisp.pl?gene=LGALS4
PDGFA	Platelet Derived Growth Factor Subunit A	Protein Coding	P04085	48	GC07M00049 7	1.96358 5	https://www.genecards.org/cgi-bin/carddisp.pl?gene=PDGFA
PROK1	Prokineticin 1	Protein Coding	P58294	40	GC01P110451	1.96315 6	https://www.genecards.org/cgi-bin/carddisp.pl?gene=PROK1
MCU	Mitochondrial Calcium Uniporter	Protein Coding	Q8NE86	42	GC10P072692	1.96305 9	https://www.genecards.org/cgi-bin/carddisp.pl?gene=MCU

PNPLA4	Patatin Like Phospholipase Domain Containing 4	Protein Coding	P41247	40	GC0XM007898	1.961876	https://www.genecards.org/cgi-bin/carddisp.pl?gene=PNPLA4
UTS2	Urotensin 2	Protein Coding	O95399	44	GC01M007843	1.961102	https://www.genecards.org/cgi-bin/carddisp.pl?gene=UTS2
MYL2	Myosin Light Chain 2	Protein Coding	P10916	52	GC12M110910	1.958944	https://www.genecards.org/cgi-bin/carddisp.pl?gene=MYL2
CD63	CD63 Molecule	Protein Coding	P08962	47	GC12M055725	1.957837	https://www.genecards.org/cgi-bin/carddisp.pl?gene=CD63
MIR4454	MicroRNA 4454	RNA Gene		13	GC04M163093	1.957429	https://www.genecards.org/cgi-bin/carddisp.pl?gene=MIR4454
SAMHD1	SAM And HD Domain Containing Deoxynucleoside Triphosphate Triphosphohydrolase 1	Protein Coding	Q9Y3Z3	47	GC20M036890	1.956514	https://www.genecards.org/cgi-bin/carddisp.pl?gene=SAMHD1
BMP15	Bone Morphogenetic Protein 15	Protein Coding	O95972	46	GC0XP050910	1.951629	https://www.genecards.org/cgi-bin/carddisp.pl?gene=BMP15
LEFTY2	Left-Right Determination Factor 2	Protein Coding	O00292	48	GC01M225948	1.948717	https://www.genecards.org/cgi-bin/carddisp.pl?gene=LEFTY2
CEBPD	CCAAT Enhancer Binding Protein Delta	Protein Coding	P49716	44	GC08M047759	1.948495	https://www.genecards.org/cgi-bin/carddisp.pl?gene=CEBPD
NOX4	NADPH Oxidase 4	Protein Coding	Q9NPH5	47	GC11M089324	1.946199	https://www.genecards.org/cgi-bin/carddisp.pl?gene=NOX4
MIR197	MicroRNA 197	RNA Gene		20	GC01P109598	1.944596	https://www.genecards.org/cgi-bin/carddisp.pl?gene=MIR197
TMPRSS2	Transmembrane Serine Protease 2	Protein Coding	O15393	51	GC21M041464	1.941994	https://www.genecards.org/cgi-bin/carddisp.pl?gene=TMPRSS2

PRDX3	Peroxiredoxin 3	Protein Coding	P30048	49	GC10M119167	1.94159	https://www.genecards.org/cgi-bin/carddisp.pl?gene=PRDX3
CUL3	Cullin 3	Protein Coding	Q13618	52	GC02M224470	1.94117	https://www.genecards.org/cgi-bin/carddisp.pl?gene=CUL3
COPG2	COPI Coat Complex Subunit Gamma 2	Protein Coding	Q9UBF2	41	GC07M130506	1.938024	https://www.genecards.org/cgi-bin/carddisp.pl?gene=COPG2
INPP5K	Inositol Polyphosphate-5-Phosphatase K	Protein Coding	Q9BT40	46	GC17M001494	1.937354	https://www.genecards.org/cgi-bin/carddisp.pl?gene=INPP5K
KLHL3	Kelch Like Family Member 3	Protein Coding	Q9UH77	45	GC05M137617	1.937306	https://www.genecards.org/cgi-bin/carddisp.pl?gene=KLHL3
FANCL	FA Complementation Group L	Protein Coding	Q9NW38	51	GC02M058127	1.935174	https://www.genecards.org/cgi-bin/carddisp.pl?gene=FANCL
MYB	MYB Proto-Oncogene, Transcription Factor	Protein Coding	P10242	53	GC06P135180	1.933404	https://www.genecards.org/cgi-bin/carddisp.pl?gene=MYB
DCN	Decorin	Protein Coding	P07585	51	GC12M091140	1.931802	https://www.genecards.org/cgi-bin/carddisp.pl?gene=DCN
CRLF2	Cytokine Receptor Like Factor 2	Protein Coding	Q9HC73	42	GC0XM001190	1.931382	https://www.genecards.org/cgi-bin/carddisp.pl?gene=CRLF2
SNORD118	Small Nucleolar RNA, C/D Box 118	RNA Gene		21	GC17M014437	1.930605	https://www.genecards.org/cgi-bin/carddisp.pl?gene=SNORD118
MYLK	Myosin Light Chain Kinase	Protein Coding	Q15746	55	GC03M123610	1.930485	https://www.genecards.org/cgi-bin/carddisp.pl?gene=MYLK
MDM2	MDM2 Proto-Oncogene	Protein Coding	Q00987	57	GC12P068808	1.928314	https://www.genecards.org/cgi-bin/carddisp.pl?gene=MDM2

FUT3	Fucosyltransferase 3 (Lewis Blood Group)	Protein Coding	P21217	45	GC19M010867	1.92821	https://www.genecards.org/cgi-bin/carddisp.pl?gene=FUT3
CDX2	Caudal Type Homeobox 2	Protein Coding	Q99626	46	GC13M027962	1.926777	https://www.genecards.org/cgi-bin/carddisp.pl?gene=CDX2
SQSTM1	Sequestosome 1	Protein Coding	Q13501	53	GC05P179806	1.925857	https://www.genecards.org/cgi-bin/carddisp.pl?gene=SQSTM1
DVL1	Dishevelled Segment Polarity Protein 1	Protein Coding	O14640	50	GC01M001335	1.925539	https://www.genecards.org/cgi-bin/carddisp.pl?gene=DVL1
MIR519A1	MicroRNA 519a-1	RNA Gene		16	GC19P053752	1.924896	https://www.genecards.org/cgi-bin/carddisp.pl?gene=MIR519A1
TFPT	TCF3 Fusion Partner	Protein Coding	P0C1Z6	40	GC19M054107	1.924072	https://www.genecards.org/cgi-bin/carddisp.pl?gene=TFPT
MIR138-1	MicroRNA 138-1	RNA Gene		21	GC03P044115	1.923009	https://www.genecards.org/cgi-bin/carddisp.pl?gene=MIR138-1
AIRE	Autoimmune Regulator	Protein Coding	O43918	48	GC21P044285	1.921245	https://www.genecards.org/cgi-bin/carddisp.pl?gene=AIRE
ACD	ACD Shelterin Complex Subunit And Telomerase Recruitment Factor	Protein Coding	Q96AP0	46	GC16M067658	1.920881	https://www.genecards.org/cgi-bin/carddisp.pl?gene=ACD
ALKBH5	AlkB Homolog 5, RNA Demethylase	Protein Coding	Q6P6C2	38	GC17P018183	1.920335	https://www.genecards.org/cgi-bin/carddisp.pl?gene=ALKBH5
VEGFB	Vascular Endothelial Growth Factor B	Protein Coding	P49765	48	GC11P064234	1.919419	https://www.genecards.org/cgi-bin/carddisp.pl?gene=VEGFB
RECQL4	RecQ Like Helicase 4	Protein Coding	O94761	45	GC08M146219	1.918393	https://www.genecards.org/cgi-bin/carddisp.pl?gene=RECQL4

AGO2	Argonaute RISC Catalytic Component 2	Protein Coding	Q9UKV8	48	GC08M140522	1.918298	https://www.genecards.org/cgi-bin/carddisp.pl?gene=AGO2
TNFSF13B	TNF Superfamily Member 13b	Protein Coding	Q9Y275	49	GC13P108251	1.918115	https://www.genecards.org/cgi-bin/carddisp.pl?gene=TNFSF13B
SFRP1	Secreted Frizzled Related Protein 1	Protein Coding	Q8N474	48	GC08M041262	1.916235	https://www.genecards.org/cgi-bin/carddisp.pl?gene=SFRP1
PCYT1A	Phosphate Cytidylyltransferase 1A, Choline	Protein Coding	P49585	51	GC03M196214	1.916201	https://www.genecards.org/cgi-bin/carddisp.pl?gene=PCYT1A
RBFOX3	RNA Binding Fox-1 Homolog 3	Protein Coding	A6NFN3	41	GC17M079089	1.91602	https://www.genecards.org/cgi-bin/carddisp.pl?gene=RBFOX3
IFNB1	Interferon Beta 1	Protein Coding	P01574	47	GC09M021077	1.915571	https://www.genecards.org/cgi-bin/carddisp.pl?gene=IFNB1
FLNB	Filamin B	Protein Coding	O75369	51	GC03P058008	1.915316	https://www.genecards.org/cgi-bin/carddisp.pl?gene=FLNB
HKDC1	Hexokinase Domain Containing 1	Protein Coding	Q2TB90	45	GC10P069220	1.914321	https://www.genecards.org/cgi-bin/carddisp.pl?gene=HKDC1
CASC9	Cancer Susceptibility 9	RNA Gene		17	GC08M075132	1.912228	https://www.genecards.org/cgi-bin/carddisp.pl?gene=CASC9
HSD17B1	Hydroxysteroid 17-Beta Dehydrogenase 1	Protein Coding	P14061	46	GC17P086410	1.910475	https://www.genecards.org/cgi-bin/carddisp.pl?gene=HSD17B1
AFAP1-AS1	AFAP1 Antisense RNA 1	RNA Gene		19	GC04P007769	1.910292	https://www.genecards.org/cgi-bin/carddisp.pl?gene=AFAP1-AS1
FOLH1	Folate Hydrolase 1	Protein Coding	Q04609	51	GC11M113197	1.909322	https://www.genecards.org/cgi-bin/carddisp.pl?gene=FOLH1

HDAC2	Histone Deacetylase 2	Protein Coding	Q92769	56	GC06M113933	1.909062	https://www.genecards.org/cgi-bin/carddisp.pl?gene=HDAC2
PRKCB	Protein Kinase C Beta	Protein Coding	P05771	52	GC16P053182	1.908106	https://www.genecards.org/cgi-bin/carddisp.pl?gene=PRKCB
ACLY	ATP Citrate Lyase	Protein Coding	P53396	51	GC17M041866	1.90401	https://www.genecards.org/cgi-bin/carddisp.pl?gene=ACLY
CTH	Cystathionine Gamma-Lyase	Protein Coding	P32929	53	GC01P070411	1.903773	https://www.genecards.org/cgi-bin/carddisp.pl?gene=CTH
SLC40A1	Solute Carrier Family 40 Member 1	Protein Coding	Q9NP59	51	GC02M189560	1.901588	https://www.genecards.org/cgi-bin/carddisp.pl?gene=SLC40A1
CCNB1	Cyclin B1	Protein Coding	P14635	51	GC05P069167	1.900332	https://www.genecards.org/cgi-bin/carddisp.pl?gene=CCNB1
TNFRSF17	TNF Receptor Superfamily Member 17	Protein Coding	Q02223	51	GC16P011965	1.896664	https://www.genecards.org/cgi-bin/carddisp.pl?gene=TNFRSF17
RARG	Retinoic Acid Receptor Gamma	Protein Coding	P13631	51	GC12M053210	1.894716	https://www.genecards.org/cgi-bin/carddisp.pl?gene=RARG
VTRNA2-1	Vault RNA 2-1	RNA Gene		17	GC05M136081	1.893531	https://www.genecards.org/cgi-bin/carddisp.pl?gene=VTRNA2-1
HTR1B	5-Hydroxytryptamine Receptor 1B	Protein Coding	P28222	48	GC06M084631	1.893481	https://www.genecards.org/cgi-bin/carddisp.pl?gene=HTR1B
MFSD2A	MFSD2 Lysolipid Transporter A, Lysophospholipid	Protein Coding	Q8NA29	46	GC01P039955	1.893158	https://www.genecards.org/cgi-bin/carddisp.pl?gene=MFSD2A
SOX9-AS1	SOX9 Antisense RNA 1	RNA Gene		17	GC17M072070	1.892113	https://www.genecards.org/cgi-bin/carddisp.pl?gene=SOX9-AS1

ROCR	Regulator Of Chondrogenesis RNA	RNA Gene		15	GC17M072023	1.892113	https://www.genecards.org/cgi-bin/carddisp.pl?gene=ROCR
ENSG00000288605	Novel Transcript	RNA Gene		9	GC17M072037	1.892113	https://www.genecards.org/cgi-bin/carddisp.pl?gene=ENSG00000288605
LOC102723517	Uncharacterized LOC102723517	RNA Gene		7	GC17M072036	1.892113	https://www.genecards.org/cgi-bin/carddisp.pl?gene=LOC102723517
NRG1	Neuregulin 1	Protein Coding	Q02297	52	GC08P031639	1.892011	https://www.genecards.org/cgi-bin/carddisp.pl?gene=NRG1
SLC12A1	Solute Carrier Family 12 Member 1	Protein Coding	Q13621	51	GC15P057460	1.89186	https://www.genecards.org/cgi-bin/carddisp.pl?gene=SLC12A1
TTTY15	Testis Expressed Transcript, Y-Linked 15	RNA Gene		16	GC0YP012620	1.891528	https://www.genecards.org/cgi-bin/carddisp.pl?gene=TTTY15
AVPR2	Arginine Vasopressin Receptor 2	Protein Coding	P30518	50	GC0XP153902	1.890703	https://www.genecards.org/cgi-bin/carddisp.pl?gene=AVPR2
TMEM216	Transmembrane Protein 216	Protein Coding	Q9P0N5	39	GC11P061427	1.890554	https://www.genecards.org/cgi-bin/carddisp.pl?gene=TMEM216
MIR30C2	MicroRNA 30c-2	RNA Gene		20	GC06M084527	1.888337	https://www.genecards.org/cgi-bin/carddisp.pl?gene=MIR30C2
LILRB1	Leukocyte Immunoglobulin Like Receptor B1	Protein Coding	Q8NHL6	47	GC19P090177	1.881926	https://www.genecards.org/cgi-bin/carddisp.pl?gene=LILRB1
HSPA8	Heat Shock Protein Family A (Hsp70) Member 8	Protein Coding	P11142	53	GC11M123057	1.87993	https://www.genecards.org/cgi-bin/carddisp.pl?gene=HSPA8
NPHP3	Nephrocystin 3	Protein Coding	Q7Z494	44	GC03M132683	1.879787	https://www.genecards.org/cgi-bin/carddisp.pl?gene=NPHP3

MCL1	MCL1 Apoptosis Regulator, BCL2 Family Member	Protein Coding	Q07820	52	GC01M157509	1.877321	https://www.genecards.org/cgi-bin/carddisp.pl?gene=MCL1
MIR181B2	MicroRNA 181b-2	RNA Gene		21	GC09P124693	1.874942	https://www.genecards.org/cgi-bin/carddisp.pl?gene=MIR181B2
CDH5	Cadherin 5	Protein Coding	P33151	51	GC16P066366	1.872423	https://www.genecards.org/cgi-bin/carddisp.pl?gene=CDH5
LACTB	Lactamase Beta	Protein Coding	P83111	41	GC15P138392	1.871875	https://www.genecards.org/cgi-bin/carddisp.pl?gene=LACTB
CDK6	Cyclin Dependent Kinase 6	Protein Coding	Q00534	55	GC07M092604	1.871054	https://www.genecards.org/cgi-bin/carddisp.pl?gene=CDK6
PEX7	Peroxisomal Biogenesis Factor 7	Protein Coding	O00628	48	GC06P136822	1.869236	https://www.genecards.org/cgi-bin/carddisp.pl?gene=PEX7
MTERF1	Mitochondrial Transcription Termination Factor 1	Protein Coding	Q99551	39	GC07M091692	1.867543	https://www.genecards.org/cgi-bin/carddisp.pl?gene=MTERF1
TRIM27	Tripartite Motif Containing 27	Protein Coding	P14373	45	GC06M028903	1.867153	https://www.genecards.org/cgi-bin/carddisp.pl?gene=TRIM27
RYR2	Ryanodine Receptor 2	Protein Coding	Q92736	51	GC01P237042	1.866465	https://www.genecards.org/cgi-bin/carddisp.pl?gene=RYR2
TMEM231	Transmembrane Protein 231	Protein Coding	Q9H6L2	42	GC16M075536	1.866364	https://www.genecards.org/cgi-bin/carddisp.pl?gene=TMEM231
HOXA13	Homeobox A13	Protein Coding	P31271	45	GC07M027866	1.866117	https://www.genecards.org/cgi-bin/carddisp.pl?gene=HOXA13
SCGB1A1	Secretoglobin Family 1A Member 1	Protein Coding	P11684	45	GC11P062405	1.866025	https://www.genecards.org/cgi-bin/carddisp.pl?gene=SCGB1A1

BLM	BLM RecQ Like Helicase	Protein Coding	P54132	53	GC15P090717	1.865995	https://www.genecards.org/cgi-bin/carddisp.pl?gene=BLM
WT1-AS	WT1 Antisense RNA	RNA Gene	Q06250	30	GC11P032434	1.865994	https://www.genecards.org/cgi-bin/carddisp.pl?gene=WT1-AS
GLUD1	Glutamate Dehydrogenase 1	Protein Coding	P00367	54	GC10M087050	1.86456	https://www.genecards.org/cgi-bin/carddisp.pl?gene=GLUD1
PNOC	Prepronociceptin	Protein Coding	Q13519	41	GC08P028316	1.862631	https://www.genecards.org/cgi-bin/carddisp.pl?gene=PNOC
IHH	Indian Hedgehog Signaling Molecule	Protein Coding	Q14623	51	GC02M219054	1.861254	https://www.genecards.org/cgi-bin/carddisp.pl?gene=IHH
GALNT2	Polypeptide N-Acetylgalactosaminyltransferase 2	Protein Coding	Q10471	49	GC01P230057	1.86118	https://www.genecards.org/cgi-bin/carddisp.pl?gene=GALNT2
PPP1R3B	Protein Phosphatase 1 Regulatory Subunit 3B	Protein Coding	Q86XI6	40	GC08M009136	1.85751	https://www.genecards.org/cgi-bin/carddisp.pl?gene=PPP1R3B
BARX1	BARX Homeobox 1	Protein Coding	Q9HBU1	40	GC09M093951	1.857411	https://www.genecards.org/cgi-bin/carddisp.pl?gene=BARX1
ANG	Angiogenin	Protein Coding	P03950	49	GC14P039963	1.856959	https://www.genecards.org/cgi-bin/carddisp.pl?gene=ANG
MIR3196	MicroRNA 3196	RNA Gene		12	GC20P065701	1.856901	https://www.genecards.org/cgi-bin/carddisp.pl?gene=MIR3196
CHL1	Cell Adhesion Molecule L1 Like	Protein Coding	O00533	47	GC03P000213	1.85281	https://www.genecards.org/cgi-bin/carddisp.pl?gene=CHL1
BEGAIN	Brain Enriched Guanylate Kinase Associated	Protein Coding	Q9BUH8	40	GC14M116463	1.851882	https://www.genecards.org/cgi-bin/carddisp.pl?gene=BEGAIN

CADM1	Cell Adhesion Molecule 1	Protein Coding	Q9BY67	48	GC11M115169	1.851449	https://www.genecards.org/cgi-bin/carddisp.pl?gene=CADM1
MUSK	Muscle Associated Receptor Tyrosine Kinase	Protein Coding	O15146	53	GC09P110668	1.850886	https://www.genecards.org/cgi-bin/carddisp.pl?gene=MUSK
ALDOA	Aldolase, Fructose-Bisphosphate A	Protein Coding	P04075	52	GC16P030064	1.849319	https://www.genecards.org/cgi-bin/carddisp.pl?gene=ALDOA
AQP1	Aquaporin 1 (Colton Blood Group)	Protein Coding	P29972	51	GC07P030911	1.849185	https://www.genecards.org/cgi-bin/carddisp.pl?gene=AQP1
MAT1A	Methionine Adenosyltransferase 1A	Protein Coding	Q00266	50	GC10M080271	1.834123	https://www.genecards.org/cgi-bin/carddisp.pl?gene=MAT1A
GZMB	Granzyme B	Protein Coding	P10144	51	GC14M024630	1.833975	https://www.genecards.org/cgi-bin/carddisp.pl?gene=GZMB
TRNT1	TRNA Nucleotidyl Transferase 1	Protein Coding	Q96Q11	46	GC03P003126	1.833151	https://www.genecards.org/cgi-bin/carddisp.pl?gene=TRNT1
SPTAN1	Spectrin Alpha, Non-Erythrocytic 1	Protein Coding	Q13813	52	GC09P128552	1.832873	https://www.genecards.org/cgi-bin/carddisp.pl?gene=SPTAN1
CHRNA5	Cholinergic Receptor Nicotinic Alpha 5 Subunit	Protein Coding	P30532	49	GC15P078565	1.830531	https://www.genecards.org/cgi-bin/carddisp.pl?gene=CHRNA5
TCN1	Transcobalamin 1	Protein Coding	P20061	46	GC11M113293	1.8297	https://www.genecards.org/cgi-bin/carddisp.pl?gene=TCN1
MIR302D	MicroRNA 302d	RNA Gene		21	GC04M112648	1.829303	https://www.genecards.org/cgi-bin/carddisp.pl?gene=MIR302D
ADORA2B	Adenosine A2b Receptor	Protein Coding	P29275	52	GC17P085485	1.828343	https://www.genecards.org/cgi-bin/carddisp.pl?gene=ADORA2B

ACKR1	Atypical Chemokine Receptor 1 (Duffy Blood Group)	Protein Coding	Q16570	46	GC01P159203	1.828343	https://www.genecards.org/cgi-bin/carddisp.pl?gene=ACKR1
LMOD1	Leiomodin 1	Protein Coding	P29536	45	GC01M201896	1.827739	https://www.genecards.org/cgi-bin/carddisp.pl?gene=LMOD1
ACTA2-AS1	ACTA2 Antisense RNA 1	RNA Gene		19	GC10P088932	1.827325	https://www.genecards.org/cgi-bin/carddisp.pl?gene=ACTA2-AS1
MB	Myoglobin	Protein Coding	P02144	48	GC22M035606	1.827208	https://www.genecards.org/cgi-bin/carddisp.pl?gene=MB
HRH1	Histamine Receptor H1	Protein Coding	P35367	50	GC03P018373	1.827112	https://www.genecards.org/cgi-bin/carddisp.pl?gene=HRH1
RNF213	Ring Finger Protein 213	Protein Coding	Q63HN8	42	GC17P080260	1.825446	https://www.genecards.org/cgi-bin/carddisp.pl?gene=RNF213
DUSP1	Dual Specificity Phosphatase 1	Protein Coding	P28562	50	GC05M172768	1.824903	https://www.genecards.org/cgi-bin/carddisp.pl?gene=DUSP1
FLT4	Fms Related Receptor Tyrosine Kinase 4	Protein Coding	P35916	56	GC05M182282	1.822493	https://www.genecards.org/cgi-bin/carddisp.pl?gene=FLT4
SFRP4	Secreted Frizzled Related Protein 4	Protein Coding	Q6FHJ7	47	GC07M037912	1.820458	https://www.genecards.org/cgi-bin/carddisp.pl?gene=SFRP4
PGM1	Phosphoglucomutase 1	Protein Coding	P36871	52	GC01P063593	1.819937	https://www.genecards.org/cgi-bin/carddisp.pl?gene=PGM1
IFT81	Intraflagellar Transport 81	Protein Coding	Q8WYA0	45	GC12P110124	1.818665	https://www.genecards.org/cgi-bin/carddisp.pl?gene=IFT81
MIR361	MicroRNA 361	RNA Gene		17	GC0XM085903	1.817978	https://www.genecards.org/cgi-bin/carddisp.pl?gene=MIR361

SNX29	Sorting Nexin 29	Protein Coding	Q8TEQ0	39	GC16P011976	1.817887	https://www.genecards.org/cgi-bin/carddisp.pl?gene=SNX29
CCN3	Cellular Communication Network Factor 3	Protein Coding	P48745	44	GC08P119416	1.816938	https://www.genecards.org/cgi-bin/carddisp.pl?gene=CCN3
MSBP2	Minisatellite Binding Protein 2	Protein Coding		5	GC00U990214	1.81687	https://www.genecards.org/cgi-bin/carddisp.pl?gene=MSBP2
FHL1	Four And A Half LIM Domains 1	Protein Coding	Q13642	49	GC0XP136146	1.816342	https://www.genecards.org/cgi-bin/carddisp.pl?gene=FHL1
POU6F2	POU Class 6 Homeobox 2	Protein Coding	P78424	43	GC07P038977	1.815047	https://www.genecards.org/cgi-bin/carddisp.pl?gene=POU6F2
TENM3	Teneurin Transmembrane Protein 3	Protein Coding	Q9P273	41	GC04P181448	1.815047	https://www.genecards.org/cgi-bin/carddisp.pl?gene=TENM3
PEPD	Peptidase D	Protein Coding	P12955	50	GC19M033386	1.814855	https://www.genecards.org/cgi-bin/carddisp.pl?gene=PEPD
IGES	Immunoglobulin E Concentration, Serum	Genetic Locus		4	GC05U990033	1.814737	https://www.genecards.org/cgi-bin/carddisp.pl?gene=IGES
CEACAM5	CEA Cell Adhesion Molecule 5	Protein Coding	P06731	47	GC19P089622	1.814164	https://www.genecards.org/cgi-bin/carddisp.pl?gene=CEACAM5
FGFR4	Fibroblast Growth Factor Receptor 4	Protein Coding	P22455	55	GC05P177086	1.812034	https://www.genecards.org/cgi-bin/carddisp.pl?gene=FGFR4
BIRC3	Baculoviral IAP Repeat Containing 3	Protein Coding	Q13489	51	GC11P102317	1.811343	https://www.genecards.org/cgi-bin/carddisp.pl?gene=BIRC3
IGFBP7	Insulin Like Growth Factor Binding Protein 7	Protein Coding	Q16270	50	GC04M057030	1.810269	https://www.genecards.org/cgi-bin/carddisp.pl?gene=IGFBP7

MDFIC	MyoD Family Inhibitor Domain Containing	Protein Coding	Q9P1T7	41	GC07P114922	1.810096	https://www.genecards.org/cgi-bin/carddisp.pl?gene=MDFIC
OPRK1	Opioid Receptor Kappa 1	Protein Coding	P41145	48	GC08M053227	1.809955	https://www.genecards.org/cgi-bin/carddisp.pl?gene=OPRK1
TBX22	T-Box Transcription Factor 22	Protein Coding	Q9Y458	42	GC0XP080014	1.809025	https://www.genecards.org/cgi-bin/carddisp.pl?gene=TBX22
PEX6	Peroxisomal Biogenesis Factor 6	Protein Coding	Q13608	46	GC06M042963	1.808861	https://www.genecards.org/cgi-bin/carddisp.pl?gene=PEX6
CRABP1	Cellular Retinoic Acid Binding Protein 1	Protein Coding	P29762	46	GC15P078340	1.80574	https://www.genecards.org/cgi-bin/carddisp.pl?gene=CRABP1
MIR20B	MicroRNA 20b	RNA Gene		16	GC0XM134397	1.80528	https://www.genecards.org/cgi-bin/carddisp.pl?gene=MIR20B
SELENOS	Selenoprotein S	Protein Coding	Q9BQE4	41	GC15M137688	1.804453	https://www.genecards.org/cgi-bin/carddisp.pl?gene=SELENOS
SETD1A	SET Domain Containing 1A, Histone Lysine Methyltransferase	Protein Coding	O15047	46	GC16P054216	1.802022	https://www.genecards.org/cgi-bin/carddisp.pl?gene=SETD1A
MKX	Mohawk Homeobox	Protein Coding	Q8IYA7	40	GC10M028164	1.801918	https://www.genecards.org/cgi-bin/carddisp.pl?gene=MKX
DLX6	Distal-Less Homeobox 6	Protein Coding	P56179	43	GC07P097005	1.797819	https://www.genecards.org/cgi-bin/carddisp.pl?gene=DLX6
GK	Glycerol Kinase	Protein Coding	P32189	49	GC0XP031249	1.797712	https://www.genecards.org/cgi-bin/carddisp.pl?gene=GK
MIR3940	MicroRNA 3940	RNA Gene		14	GC19M010885	1.797531	https://www.genecards.org/cgi-bin/carddisp.pl?gene=MIR3940

PHTF1	Putative Homeodomain Transcription Factor 1	Protein Coding	Q9UMS5	40	GC01M113696	1.796337	https://www.genecards.org/cgi-bin/carddisp.pl?gene=PHTF1
LGR5	Leucine Rich Repeat Containing G Protein-Coupled Receptor 5	Protein Coding	O75473	48	GC12P071439	1.795208	https://www.genecards.org/cgi-bin/carddisp.pl?gene=LGR5
NRG3	Neuregulin 3	Protein Coding	P56975	47	GC10P100839	1.794656	https://www.genecards.org/cgi-bin/carddisp.pl?gene=NRG3
SLC11A2	Solute Carrier Family 11 Member 2	Protein Coding	P49281	52	GC12M050952	1.794032	https://www.genecards.org/cgi-bin/carddisp.pl?gene=SLC11A2
RAG1	Recombination Activating 1	Protein Coding	P15918	48	GC11P036551	1.789322	https://www.genecards.org/cgi-bin/carddisp.pl?gene=RAG1
TBX2	T-Box Transcription Factor 2	Protein Coding	Q13207	50	GC17P061399	1.78715	https://www.genecards.org/cgi-bin/carddisp.pl?gene=TBX2
CDK5	Cyclin Dependent Kinase 5	Protein Coding	Q00535	57	GC07M151053	1.785974	https://www.genecards.org/cgi-bin/carddisp.pl?gene=CDK5
SPRY4	Sprouty RTK Signaling Antagonist 4	Protein Coding	Q9C004	48	GC05M142310	1.785914	https://www.genecards.org/cgi-bin/carddisp.pl?gene=SPRY4
LRAT	Lecithin Retinol Acyltransferase	Protein Coding	O95237	48	GC04P154626	1.785864	https://www.genecards.org/cgi-bin/carddisp.pl?gene=LRAT
RFX6	Regulatory Factor X6	Protein Coding	Q8HWS3	42	GC06P116877	1.785567	https://www.genecards.org/cgi-bin/carddisp.pl?gene=RFX6
ANKRD11	Ankyrin Repeat Domain Containing 11	Protein Coding	Q6UB99	43	GC16M089267	1.783312	https://www.genecards.org/cgi-bin/carddisp.pl?gene=ANKRD11
MIR511	MicroRNA 511	RNA Gene		16	GC10P017845	1.781699	https://www.genecards.org/cgi-bin/carddisp.pl?gene=MIR511

ANKH	ANKH Inorganic Pyrophosphate Transport Regulator	Protein Coding	Q9HCJ1	44	GC05M014782	1.781574	https://www.genecards.org/cgi-bin/carddisp.pl?gene=ANKH
AVPR1B	Arginine Vasopressin Receptor 1B	Protein Coding	P47901	47	GC01M206109	1.781455	https://www.genecards.org/cgi-bin/carddisp.pl?gene=AVPR1B
PRDM2	PR/SET Domain 2	Protein Coding	Q13029	45	GC01P013700	1.780351	https://www.genecards.org/cgi-bin/carddisp.pl?gene=PRDM2
WNT9B	Wnt Family Member 9B	Protein Coding	O14905	46	GC17P046833	1.779159	https://www.genecards.org/cgi-bin/carddisp.pl?gene=WNT9B
MUC16	Mucin 16, Cell Surface Associated	Protein Coding	Q8WXI7	42	GC19M008848	1.779043	https://www.genecards.org/cgi-bin/carddisp.pl?gene=MUC16
WNT7A	Wnt Family Member 7A	Protein Coding	O00755	52	GC03M024417	1.77795	https://www.genecards.org/cgi-bin/carddisp.pl?gene=WNT7A
MIR124-2	MicroRNA 124-2	RNA Gene		20	GC08P064379	1.777503	https://www.genecards.org/cgi-bin/carddisp.pl?gene=MIR124-2
CHRNA4	Cholinergic Receptor Nicotinic Alpha 4 Subunit	Protein Coding	P43681	53	GC20M063343	1.77603	https://www.genecards.org/cgi-bin/carddisp.pl?gene=CHRNA4
MSH2	MutS Homolog 2	Protein Coding	P43246	53	GC02P047403	1.773557	https://www.genecards.org/cgi-bin/carddisp.pl?gene=MSH2
CHRNB4	Cholinergic Receptor Nicotinic Beta 4 Subunit	Protein Coding	P30926	48	GC15M078624	1.773432	https://www.genecards.org/cgi-bin/carddisp.pl?gene=CHRNB4
ACTN3	Actinin Alpha 3	Protein Coding	Q08043	44	GC11P066546	1.773121	https://www.genecards.org/cgi-bin/carddisp.pl?gene=ACTN3
PGRMC2	Progesterone Receptor Membrane Component 2	Protein Coding	O15173	44	GC04M128269	1.772853	https://www.genecards.org/cgi-bin/carddisp.pl?gene=PGRMC2

FOXL1	Forkhead Box L1	Protein Coding	Q12952	40	GC16P086576	1.771369	https://www.genecards.org/cgi-bin/carddisp.pl?gene=FOXL1
PTPRN2	Protein Tyrosine Phosphatase Receptor Type N2	Protein Coding	Q92932	48	GC07M157539	1.770675	https://www.genecards.org/cgi-bin/carddisp.pl?gene=PTPRN2
TXN	Thioredoxin	Protein Coding	P10599	50	GC09M110243	1.770402	https://www.genecards.org/cgi-bin/carddisp.pl?gene=TXN
EGR3	Early Growth Response 3	Protein Coding	Q06889	45	GC08M022687	1.769826	https://www.genecards.org/cgi-bin/carddisp.pl?gene=EGR3
PDHX	Pyruvate Dehydrogenase Complex Component X	Protein Coding	O00330	50	GC11P034894	1.769615	https://www.genecards.org/cgi-bin/carddisp.pl?gene=PDHX
SQOR	Sulfide Quinone Oxidoreductase	Protein Coding	Q9Y6N5	44	GC15P057404	1.769615	https://www.genecards.org/cgi-bin/carddisp.pl?gene=SQOR
MLH1	MutL Homolog 1	Protein Coding	P40692	53	GC03P036993	1.76864	https://www.genecards.org/cgi-bin/carddisp.pl?gene=MLH1
PTK2	Protein Tyrosine Kinase 2	Protein Coding	Q05397	52	GC08M140657	1.768089	https://www.genecards.org/cgi-bin/carddisp.pl?gene=PTK2
CYP4V2	Cytochrome P450 Family 4 Subfamily V Member 2	Protein Coding	Q6ZWL3	45	GC04P186191	1.767669	https://www.genecards.org/cgi-bin/carddisp.pl?gene=CYP4V2
SLC22A11	Solute Carrier Family 22 Member 11	Protein Coding	Q9NSA0	44	GC11P064862	1.767544	https://www.genecards.org/cgi-bin/carddisp.pl?gene=SLC22A11
LINC00523	Long Intergenic Non-Protein Coding RNA 523	RNA Gene	Q86TU6	24	GC14P113126	1.76753	https://www.genecards.org/cgi-bin/carddisp.pl?gene=LINC00523
DICER1	Dicer 1, Ribonuclease III	Protein Coding	Q9UPY3	52	GC14M095086	1.767398	https://www.genecards.org/cgi-bin/carddisp.pl?gene=DICER1

BMP7	Bone Morphogenetic Protein 7	Protein Coding	P18075	51	GC20M057168	1.764038	https://www.genecards.org/cgi-bin/carddisp.pl?gene=BMP7
ADM2	Adrenomedullin 2	Protein Coding	Q7Z4H4	38	GC22P050481	1.763266	https://www.genecards.org/cgi-bin/carddisp.pl?gene=ADM2
MCTP2	Multiple C2 And Transmembrane Domain Containing 2	Protein Coding	Q6DN12	45	GC15P140057	1.763218	https://www.genecards.org/cgi-bin/carddisp.pl?gene=MCTP2
DYNC1H1	Dynein Cytoplasmic 1 Heavy Chain 1	Protein Coding	Q14204	49	GC14P112931	1.762105	https://www.genecards.org/cgi-bin/carddisp.pl?gene=DYNC1H1
MIR941-1	MicroRNA 941-1	RNA Gene		15	GC20P065732	1.758862	https://www.genecards.org/cgi-bin/carddisp.pl?gene=MIR941-1
TREM2	Triggering Receptor Expressed On Myeloid Cells 2	Protein Coding	Q9NZC2	48	GC06M084142	1.757175	https://www.genecards.org/cgi-bin/carddisp.pl?gene=TREM2
SNAI2	Snail Family Transcriptional Repressor 2	Protein Coding	O43623	47	GC08M048917	1.75689	https://www.genecards.org/cgi-bin/carddisp.pl?gene=SNAI2
MIR331	MicroRNA 331	RNA Gene		21	GC12P095308	1.753262	https://www.genecards.org/cgi-bin/carddisp.pl?gene=MIR331
FOXC1	Forkhead Box C1	Protein Coding	Q12948	47	GC06P001610	1.752961	https://www.genecards.org/cgi-bin/carddisp.pl?gene=FOXC1
KLF9	KLF Transcription Factor 9	Protein Coding	Q13886	39	GC09M070384	1.752082	https://www.genecards.org/cgi-bin/carddisp.pl?gene=KLF9
SERPINB5	Serpin Family B Member 5	Protein Coding	P36952	48	GC18P063476	1.751564	https://www.genecards.org/cgi-bin/carddisp.pl?gene=SERPINB5
SNHG5	Small Nucleolar RNA Host Gene 5	RNA Gene		22	GC06M085650	1.751474	https://www.genecards.org/cgi-bin/carddisp.pl?gene=SNHG5

LRRTM1	Leucine Rich Repeat Transmembrane Neuronal 1	Protein Coding	Q86UE6	44	GC02M080264	1.750437	https://www.genecards.org/cgi-bin/carddisp.pl?gene=LRRTM1
MAP6D1	MAP6 Domain Containing 1	Protein Coding	Q9H9H5	35	GC03M183815	1.750356	https://www.genecards.org/cgi-bin/carddisp.pl?gene=MAP6D1
FKRP	Fukutin Related Protein	Protein Coding	Q9H9S5	44	GC19P089785	1.748117	https://www.genecards.org/cgi-bin/carddisp.pl?gene=FKRP
NDUFA8	NADH:Ubiquinone Oxidoreductase Subunit A8	Protein Coding	P51970	47	GC09M122132	1.748065	https://www.genecards.org/cgi-bin/carddisp.pl?gene=NDUFA8
XRCC3	X-Ray Repair Cross Complementing 3	Protein Coding	O43542	46	GC14M103697	1.747251	https://www.genecards.org/cgi-bin/carddisp.pl?gene=XRCC3
EIF2B3	Eukaryotic Translation Initiation Factor 2B Subunit Gamma	Protein Coding	Q9NR50	45	GC01M044850	1.746682	https://www.genecards.org/cgi-bin/carddisp.pl?gene=EIF2B3
PZP	PZP Alpha-2-Macroglobulin Like	Protein Coding	P20742	45	GC12M009148	1.746245	https://www.genecards.org/cgi-bin/carddisp.pl?gene=PZP
CYP27A1	Cytochrome P450 Family 27 Subfamily A Member 1	Protein Coding	Q02318	50	GC02P218781	1.745272	https://www.genecards.org/cgi-bin/carddisp.pl?gene=CYP27A1
P4HB	Prolyl 4-Hydroxylase Subunit Beta	Protein Coding	P07237	53	GC17M081843	1.741891	https://www.genecards.org/cgi-bin/carddisp.pl?gene=P4HB
ZNF274	Zinc Finger Protein 274	Protein Coding	Q96GC6	39	GC19P090333	1.741186	https://www.genecards.org/cgi-bin/carddisp.pl?gene=ZNF274
AK1	Adenylate Kinase 1	Protein Coding	P00568	50	GC09M127866	1.739574	https://www.genecards.org/cgi-bin/carddisp.pl?gene=AK1
CCL17	C-C Motif Chemokine Ligand 17	Protein Coding	Q92583	44	GC16P059631	1.737719	https://www.genecards.org/cgi-bin/carddisp.pl?gene=CCL17

NPSR1	Neuropeptide S Receptor 1	Protein Coding	Q6W5P4	45	GC07P034658	1.736297	https://www.genecards.org/cgi-bin/carddisp.pl?gene=NPSR1
ABCC9	ATP Binding Cassette Subfamily C Member 9	Protein Coding	O60706	49	GC12M021797	1.735865	https://www.genecards.org/cgi-bin/carddisp.pl?gene=ABCC9
GNMT	Glycine N-Methyltransferase	Protein Coding	Q14749	50	GC06P042960	1.735362	https://www.genecards.org/cgi-bin/carddisp.pl?gene=GNMT
IGSF1	Immunoglobulin Superfamily Member 1	Protein Coding	Q8N6C5	45	GC0XM131273	1.73519	https://www.genecards.org/cgi-bin/carddisp.pl?gene=IGSF1
STK25	Serine/Threonine Kinase 25	Protein Coding	O00506	45	GC02M241492	1.731335	https://www.genecards.org/cgi-bin/carddisp.pl?gene=STK25
ODC1	Ornithine Decarboxylase 1	Protein Coding	P11926	52	GC02M010432	1.730933	https://www.genecards.org/cgi-bin/carddisp.pl?gene=ODC1
HOXA10	Homeobox A10	Protein Coding	P31260	44	GC07M027865	1.730911	https://www.genecards.org/cgi-bin/carddisp.pl?gene=HOXA10
NECTIN2	Nectin Cell Adhesion Molecule 2	Protein Coding	Q92692	48	GC19P089738	1.728002	https://www.genecards.org/cgi-bin/carddisp.pl?gene=NECTIN2
SLC2A10	Solute Carrier Family 2 Member 10	Protein Coding	O95528	46	GC20P046709	1.727721	https://www.genecards.org/cgi-bin/carddisp.pl?gene=SLC2A10
PAX8-AS1	PAX8 Antisense RNA 1	RNA Gene		19	GC02P127093	1.726889	https://www.genecards.org/cgi-bin/carddisp.pl?gene=PAX8-AS1
SLPI	Secretory Leukocyte Peptidase Inhibitor	Protein Coding	P03973	45	GC20M045252	1.72611	https://www.genecards.org/cgi-bin/carddisp.pl?gene=SLPI
MIR525	MicroRNA 525	RNA Gene		17	GC19P053697	1.725163	https://www.genecards.org/cgi-bin/carddisp.pl?gene=MIR525

SLIT2	Slit Guidance Ligand 2	Protein Coding	O94813	50	GC04P022266	1.72171	https://www.genecards.org/cgi-bin/carddisp.pl?gene=SLIT2
GTF3A	General Transcription Factor IIIA	Protein Coding	Q92664	40	GC13P027518	1.71924	https://www.genecards.org/cgi-bin/carddisp.pl?gene=GTF3A
PNMT	Phenylethanolamine N-Methyltransferase	Protein Coding	P11086	47	GC17P039667	1.718731	https://www.genecards.org/cgi-bin/carddisp.pl?gene=PNMT
TRPV4	Transient Receptor Potential Cation Channel Subfamily V Member 4	Protein Coding	Q9HBA0	52	GC12M109783	1.718526	https://www.genecards.org/cgi-bin/carddisp.pl?gene=TRPV4
IGFL3	IGF Like Family Member 3	Protein Coding	Q6UXB1	33	GC19M046120	1.717988	https://www.genecards.org/cgi-bin/carddisp.pl?gene=IGFL3
FARS2	Phenylalanyl-TRNA Synthetase 2, Mitochondrial	Protein Coding	O95363	48	GC06P005499	1.717852	https://www.genecards.org/cgi-bin/carddisp.pl?gene=FARS2
FENDRR	FOXF1 Adjacent Non-Coding Developmental Regulatory RNA	RNA Gene		21	GC16M086511	1.717852	https://www.genecards.org/cgi-bin/carddisp.pl?gene=FENDRR
MIR29B1	MicroRNA 29b-1	RNA Gene		22	GC07M130877	1.717695	https://www.genecards.org/cgi-bin/carddisp.pl?gene=MIR29B1
FURIN	Furin, Paired Basic Amino Acid Cleaving Enzyme	Protein Coding	P09958	52	GC15P090868	1.716444	https://www.genecards.org/cgi-bin/carddisp.pl?gene=FURIN
SALL4	Spalt Like Transcription Factor 4	Protein Coding	Q9UJQ4	48	GC20M051784	1.715986	https://www.genecards.org/cgi-bin/carddisp.pl?gene=SALL4
HIRA	Histone Cell Cycle Regulator	Protein Coding	P54198	46	GC22M019330	1.71537	https://www.genecards.org/cgi-bin/carddisp.pl?gene=HIRA
MYO15A	Myosin XVA	Protein Coding	Q9UKN7	40	GC17P018108	1.714082	https://www.genecards.org/cgi-bin/carddisp.pl?gene=MYO15A

MIR30B	MicroRNA 30b	RNA Gene		22	GC08M134800	1.71353	https://www.genecards.org/cgi-bin/carddisp.pl?gene=MIR30B
SLC7A11	Solute Carrier Family 7 Member 11	Protein Coding	Q9UPY5	49	GC04M138164	1.711111	https://www.genecards.org/cgi-bin/carddisp.pl?gene=SLC7A11
EDNRB	Endothelin Receptor Type B	Protein Coding	P24530	54	GC13M077895	1.70998	https://www.genecards.org/cgi-bin/carddisp.pl?gene=EDNRB
COX10	Cytochrome C Oxidase Assembly Factor Heme A:Farnesyltransferase COX10	Protein Coding	Q12887	47	GC17P014069	1.708924	https://www.genecards.org/cgi-bin/carddisp.pl?gene=COX10
DISC1	DISC1 Scaffold Protein	Protein Coding	Q9NRI5	47	GC01P231626	1.708579	https://www.genecards.org/cgi-bin/carddisp.pl?gene=DISC1
MIR22HG	MIR22 Host Gene	RNA Gene	Q0VDD5	29	GC17M014099	1.707972	https://www.genecards.org/cgi-bin/carddisp.pl?gene=MIR22HG
NEAT1	Nuclear Paraspeckle Assembly Transcript 1	RNA Gene		26	GC11P079848	1.707972	https://www.genecards.org/cgi-bin/carddisp.pl?gene=NEAT1
CHKA	Choline Kinase Alpha	Protein Coding	P35790	48	GC11M068052	1.707187	https://www.genecards.org/cgi-bin/carddisp.pl?gene=CHKA
FGF14	Fibroblast Growth Factor 14	Protein Coding	Q92915	47	GC13M101710	1.706022	https://www.genecards.org/cgi-bin/carddisp.pl?gene=FGF14
CXCL14	C-X-C Motif Chemokine Ligand 14	Protein Coding	O95715	41	GC05M135617	1.704814	https://www.genecards.org/cgi-bin/carddisp.pl?gene=CXCL14
NRG4	Neuregulin 4	Protein Coding	Q8WWG1	42	GC15M075935	1.703094	https://www.genecards.org/cgi-bin/carddisp.pl?gene=NRG4
LOC654780	Splicing Factor Proline/Glutamine-Rich	RNA Gene		16	GC16M084193	1.703063	https://www.genecards.org/cgi-bin/carddisp.pl?gene=LOC654780

KRT5	Keratin 5	Protein Coding	P13647	50	GC12M052514	1.702057	https://www.genecards.org/cgi-bin/carddisp.pl?gene=KRT5
ST7-OT3	ST7 Overlapping Transcript 3	RNA Gene		17	GC07P117132	1.701829	https://www.genecards.org/cgi-bin/carddisp.pl?gene=ST7-OT3
ALPI	Alkaline Phosphatase, Intestinal	Protein Coding	P09923	51	GC02P232456	1.700122	https://www.genecards.org/cgi-bin/carddisp.pl?gene=ALPI
GDF9	Growth Differentiation Factor 9	Protein Coding	O60383	44	GC05M132861	1.69958	https://www.genecards.org/cgi-bin/carddisp.pl?gene=GDF9
CCL7	C-C Motif Chemokine Ligand 7	Protein Coding	P80098	46	GC17P034270	1.699319	https://www.genecards.org/cgi-bin/carddisp.pl?gene=CCL7
IL34	Interleukin 34	Protein Coding	Q6ZMJ4	43	GC16P073604	1.697874	https://www.genecards.org/cgi-bin/carddisp.pl?gene=IL34
CDH3	Cadherin 3	Protein Coding	P22223	51	GC16P068852	1.697788	https://www.genecards.org/cgi-bin/carddisp.pl?gene=CDH3
SERPINF2	Serpin Family F Member 2	Protein Coding	P08697	50	GC17P001742	1.697699	https://www.genecards.org/cgi-bin/carddisp.pl?gene=SERPINF2
MIR365B	MicroRNA 365b	RNA Gene		15	GC17P085944	1.696653	https://www.genecards.org/cgi-bin/carddisp.pl?gene=MIR365B
SELENON	Selenoprotein N	Protein Coding	Q9NZV5	41	GC01P025800	1.696494	https://www.genecards.org/cgi-bin/carddisp.pl?gene=SELENON
CD82	CD82 Molecule	Protein Coding	P27701	47	GC11P044586	1.696195	https://www.genecards.org/cgi-bin/carddisp.pl?gene=CD82
GLIS3	GLIS Family Zinc Finger 3	Protein Coding	Q8NEA6	42	GC09M003816	1.695094	https://www.genecards.org/cgi-bin/carddisp.pl?gene=GLIS3

MYO7A	Myosin VIIA	Protein Coding	Q13402	47	GC11P077128	1.694955	https://www.genecards.org/cgi-bin/carddisp.pl?gene=MYO7A
SOS1	SOS Ras/Rac Guanine Nucleotide Exchange Factor 1	Protein Coding	Q07889	54	GC02M039163	1.69479	https://www.genecards.org/cgi-bin/carddisp.pl?gene=SOS1
LAMA4	Laminin Subunit Alpha 4	Protein Coding	Q16363	50	GC06M112107	1.693886	https://www.genecards.org/cgi-bin/carddisp.pl?gene=LAMA4
OPN4	Opsin 4	Protein Coding	Q9UHM6	44	GC10P086654	1.693737	https://www.genecards.org/cgi-bin/carddisp.pl?gene=OPN4
VLDLR	Very Low Density Lipoprotein Receptor	Protein Coding	P98155	52	GC09P002611	1.693623	https://www.genecards.org/cgi-bin/carddisp.pl?gene=VLDLR
PLK1	Polo Like Kinase 1	Protein Coding	P53350	55	GC16P053178	1.692016	https://www.genecards.org/cgi-bin/carddisp.pl?gene=PLK1
YWHAG	Tyrosine 3-Monooxygenase/Tryptophan 5-Monooxygenase Activation Protein Gamma	Protein Coding	P61981	52	GC07M078080	1.690937	https://www.genecards.org/cgi-bin/carddisp.pl?gene=YWHAG
FTH1	Ferritin Heavy Chain 1	Protein Coding	P02794	54	GC11M061959	1.686798	https://www.genecards.org/cgi-bin/carddisp.pl?gene=FTH1
MIR501	MicroRNA 501	RNA Gene		17	GC0XP052676	1.683543	https://www.genecards.org/cgi-bin/carddisp.pl?gene=MIR501
CTCFL	CCCTC-Binding Factor Like	Protein Coding	Q8NI51	44	GC20M057495	1.683435	https://www.genecards.org/cgi-bin/carddisp.pl?gene=CTCFL
E2F4	E2F Transcription Factor 4	Protein Coding	Q16254	48	GC16P067192	1.680678	https://www.genecards.org/cgi-bin/carddisp.pl?gene=E2F4
MIR26A2	MicroRNA 26a-2	RNA Gene		21	GC12M057824	1.68018	https://www.genecards.org/cgi-bin/carddisp.pl?gene=MIR26A2

LRRC7	Leucine Rich Repeat Containing 7	Protein Coding	Q96NW7	42	GC01P069567	1.678295	https://www.genecards.org/cgi-bin/carddisp.pl?gene=LRRC7
MIR515-2	MicroRNA 515-2	RNA Gene		15	GC19P053685	1.677936	https://www.genecards.org/cgi-bin/carddisp.pl?gene=MIR515-2
ATF4	Activating Transcription Factor 4	Protein Coding	P18848	50	GC22P039519	1.677643	https://www.genecards.org/cgi-bin/carddisp.pl?gene=ATF4
DPP10	Dipeptidyl Peptidase Like 10	Protein Coding	Q8N608	46	GC02P114442	1.676333	https://www.genecards.org/cgi-bin/carddisp.pl?gene=DPP10
CSNK2A1	Casein Kinase 2 Alpha 1	Protein Coding	P68400	54	GC20M000472	1.676331	https://www.genecards.org/cgi-bin/carddisp.pl?gene=CSNK2A1
PML	PML Nuclear Body Scaffold	Protein Coding	P29590	50	GC15P073994	1.676201	https://www.genecards.org/cgi-bin/carddisp.pl?gene=PML
ACVR1C	Activin A Receptor Type 1C	Protein Coding	Q8NER5	44	GC02M157526	1.67515	https://www.genecards.org/cgi-bin/carddisp.pl?gene=ACVR1C
HAVCR1	Hepatitis A Virus Cellular Receptor 1	Protein Coding	Q96D42	46	GC05M157028	1.6751	https://www.genecards.org/cgi-bin/carddisp.pl?gene=HAVCR1
TBK1	TANK Binding Kinase 1	Protein Coding	Q9UHD2	53	GC12P064451	1.674859	https://www.genecards.org/cgi-bin/carddisp.pl?gene=TBK1
MEGF11	Multiple EGF Like Domains 11	Protein Coding	A6BM72	41	GC15M065895	1.673536	https://www.genecards.org/cgi-bin/carddisp.pl?gene=MEGF11
EPHB4	EPH Receptor B4	Protein Coding	P54760	57	GC07M104262	1.671201	https://www.genecards.org/cgi-bin/carddisp.pl?gene=EPHB4
TUBA1A	Tubulin Alpha 1a	Protein Coding	Q71U36	53	GC12M049184	1.6711	https://www.genecards.org/cgi-bin/carddisp.pl?gene=TUBA1A

ALX4	ALX Homeobox 4	Protein Coding	Q9H161	44	GC11M044238	1.667882	https://www.genecards.org/cgi-bin/carddisp.pl?gene=ALX4
IL27	Interleukin 27	Protein Coding	Q8NEV9	41	GC16M041438	1.667388	https://www.genecards.org/cgi-bin/carddisp.pl?gene=IL27
PTGER4	Prostaglandin E Receptor 4	Protein Coding	P35408	50	GC05P040679	1.664495	https://www.genecards.org/cgi-bin/carddisp.pl?gene=PTGER4
MMP16	Matrix Metallopeptidase 16	Protein Coding	P51512	49	GC08M088032	1.664344	https://www.genecards.org/cgi-bin/carddisp.pl?gene=MMP16
MMP15	Matrix Metallopeptidase 15	Protein Coding	P51511	48	GC16P058025	1.664344	https://www.genecards.org/cgi-bin/carddisp.pl?gene=MMP15
GYPA	Glycophorin A (MNS Blood Group)	Protein Coding	P02724	48	GC04M144109	1.664258	https://www.genecards.org/cgi-bin/carddisp.pl?gene=GYPA
MAP2K1	Mitogen-Activated Protein Kinase Kinase 1	Protein Coding	Q02750	58	GC15P066386	1.664222	https://www.genecards.org/cgi-bin/carddisp.pl?gene=MAP2K1
CBR1	Carbonyl Reductase 1	Protein Coding	P16152	48	GC21P036069	1.663576	https://www.genecards.org/cgi-bin/carddisp.pl?gene=CBR1
CALB2	Calbindin 2	Protein Coding	P22676	44	GC16P071358	1.663361	https://www.genecards.org/cgi-bin/carddisp.pl?gene=CALB2
CDK2	Cyclin Dependent Kinase 2	Protein Coding	P24941	55	GC12P055966	1.66321	https://www.genecards.org/cgi-bin/carddisp.pl?gene=CDK2
NUAK2	NUAK Family Kinase 2	Protein Coding	Q9H093	46	GC01M205302	1.659971	https://www.genecards.org/cgi-bin/carddisp.pl?gene=NUAK2
L3MBTL1	L3MBTL Histone Methyl-Lysine Binding Protein 1	Protein Coding	Q9Y468	44	GC20P043639	1.659464	https://www.genecards.org/cgi-bin/carddisp.pl?gene=L3MBTL1

ENSG00000243902	Extracellular Leucine Rich Repeat And Fibronectin Type III Domain Containing 2	Uncategorized		8	GC22M070416	1.659337	https://www.genecards.org/cgi-bin/carddisp.pl?gene=ENSG00000243902
MIR19B1	MicroRNA 19b-1	RNA Gene		19	GC13P091635	1.658793	https://www.genecards.org/cgi-bin/carddisp.pl?gene=MIR19B1
LAMA2	Laminin Subunit Alpha 2	Protein Coding	P24043	48	GC06P128863	1.658025	https://www.genecards.org/cgi-bin/carddisp.pl?gene=LAMA2
KLRD1	Killer Cell Lectin Like Receptor D1	Protein Coding	Q13241	45	GC12P010226	1.657814	https://www.genecards.org/cgi-bin/carddisp.pl?gene=KLRD1
NDUFAF1	NADH:Ubiquinone Oxidoreductase Complex Assembly Factor 1	Protein Coding	Q9Y375	46	GC15M041387	1.656169	https://www.genecards.org/cgi-bin/carddisp.pl?gene=NDUFAF1
ODAD2	Outer Dynein Arm Docking Complex Subunit 2	Protein Coding	Q5T2S8	40	GC10M028173	1.654458	https://www.genecards.org/cgi-bin/carddisp.pl?gene=ODAD2
SNORD43	Small Nucleolar RNA, C/D Box 43	RNA Gene		17	GC22M072140	1.653538	https://www.genecards.org/cgi-bin/carddisp.pl?gene=SNORD43
SNORD112	Small Nucleolar RNA, C/D Box 112	RNA Gene		16	GC14P112887	1.653164	https://www.genecards.org/cgi-bin/carddisp.pl?gene=SNORD112
MIR326	MicroRNA 326	RNA Gene		22	GC11M075335	1.651257	https://www.genecards.org/cgi-bin/carddisp.pl?gene=MIR326
PITX1-AS1	PITX1 Antisense RNA 1	RNA Gene		20	GC05P135042	1.649656	https://www.genecards.org/cgi-bin/carddisp.pl?gene=PITX1-AS1
SERPING1	Serpin Family G Member 1	Protein Coding	P05155	51	GC11P057597	1.647194	https://www.genecards.org/cgi-bin/carddisp.pl?gene=SERPING1
TAS1R2	Taste 1 Receptor Member 2	Protein Coding	Q8TE23	41	GC01M018839	1.647159	https://www.genecards.org/cgi-bin/carddisp.pl?gene=TAS1R2

IRF5	Interferon Regulatory Factor 5	Protein Coding	Q13568	51	GC07P128937	1.647114	https://www.genecards.org/cgi-bin/carddisp.pl?gene=IRF5
SRD5A3	Steroid 5 Alpha-Reductase 3	Protein Coding	Q9H8P0	46	GC04P055346	1.644986	https://www.genecards.org/cgi-bin/carddisp.pl?gene=SRD5A3
EIF4E	Eukaryotic Translation Initiation Factor 4E	Protein Coding	P06730	53	GC04M098879	1.644766	https://www.genecards.org/cgi-bin/carddisp.pl?gene=EIF4E
PODXL	Podocalyxin Like	Protein Coding	O00592	47	GC07M131500	1.64464	https://www.genecards.org/cgi-bin/carddisp.pl?gene=PODXL
MFGE8	Milk Fat Globule EGF And Factor V/VIII Domain Containing	Protein Coding	Q08431	47	GC15M088898	1.642526	https://www.genecards.org/cgi-bin/carddisp.pl?gene=MFGE8
MIR1207	MicroRNA 1207	RNA Gene		17	GC08P128049	1.638357	https://www.genecards.org/cgi-bin/carddisp.pl?gene=MIR1207
CXCL16	C-X-C Motif Chemokine Ligand 16	Protein Coding	Q9H2A7	43	GC17M004733	1.63779	https://www.genecards.org/cgi-bin/carddisp.pl?gene=CXCL16
YBX2	Y-Box Binding Protein 2	Protein Coding	Q9Y2T7	44	GC17M007288	1.635678	https://www.genecards.org/cgi-bin/carddisp.pl?gene=YBX2
IL13RA2	Interleukin 13 Receptor Subunit Alpha 2	Protein Coding	Q14627	43	GC0XM115003	1.635556	https://www.genecards.org/cgi-bin/carddisp.pl?gene=IL13RA2
SLC39A8	Solute Carrier Family 39 Member 8	Protein Coding	Q9C0K1	48	GC04M102252	1.635444	https://www.genecards.org/cgi-bin/carddisp.pl?gene=SLC39A8
NNT	Nicotinamide Nucleotide Transhydrogenase	Protein Coding	Q13423	48	GC05P043602	1.635348	https://www.genecards.org/cgi-bin/carddisp.pl?gene=NNT
MERTK	MER Proto-Oncogene, Tyrosine Kinase	Protein Coding	Q12866	55	GC02P111898	1.635163	https://www.genecards.org/cgi-bin/carddisp.pl?gene=MERTK

METTL15	Methyltransferase 15, Mitochondrial 12S RRNA N4-Cytidine	Protein Coding	A6NJ78	38	GC11P028109	1.633523	https://www.genecards.org/cgi-bin/carddisp.pl?gene=METTL15
SULT1E1	Sulfotransferase Family 1E Member 1	Protein Coding	P49888	48	GC04M069823	1.631574	https://www.genecards.org/cgi-bin/carddisp.pl?gene=SULT1E1
MIR941-2	MicroRNA 941-2	RNA Gene		15	GC20P065733	1.630658	https://www.genecards.org/cgi-bin/carddisp.pl?gene=MIR941-2
MIR941-4	MicroRNA 941-4	RNA Gene		15	GC20P065731	1.630658	https://www.genecards.org/cgi-bin/carddisp.pl?gene=MIR941-4
MIR941-3	MicroRNA 941-3	RNA Gene		12	GC20P065734	1.630658	https://www.genecards.org/cgi-bin/carddisp.pl?gene=MIR941-3
MIR941-5	MicroRNA 941-5	RNA Gene		9	GC20P063919	1.630658	https://www.genecards.org/cgi-bin/carddisp.pl?gene=MIR941-5
RAB3IP	RAB3A Interacting Protein	Protein Coding	Q96QF0	42	GC12P069738	1.628689	https://www.genecards.org/cgi-bin/carddisp.pl?gene=RAB3IP
MIR1323	MicroRNA 1323	RNA Gene		11	GC19P090133	1.626469	https://www.genecards.org/cgi-bin/carddisp.pl?gene=MIR1323
MIR495	MicroRNA 495	RNA Gene		17	GC14P113613	1.625644	https://www.genecards.org/cgi-bin/carddisp.pl?gene=MIR495
NRP2	Neuropilin 2	Protein Coding	O60462	50	GC02P205681	1.625593	https://www.genecards.org/cgi-bin/carddisp.pl?gene=NRP2
MIR376A1	MicroRNA 376a-1	RNA Gene		16	GC14P113593	1.623944	https://www.genecards.org/cgi-bin/carddisp.pl?gene=MIR376A1
HSD17B7	Hydroxysteroid 17-Beta Dehydrogenase 7	Protein Coding	P56937	47	GC01P162790	1.623201	https://www.genecards.org/cgi-bin/carddisp.pl?gene=HSD17B7

MIR92A2	MicroRNA 92a-2	RNA Gene		16	GC0XM134394	1.623192	https://www.genecards.org/cgi-bin/carddisp.pl?gene=MIR92A2
ESX1	ESX Homeobox 1	Protein Coding	Q8N693	39	GC0XM104250	1.621059	https://www.genecards.org/cgi-bin/carddisp.pl?gene=ESX1
LRP8	LDL Receptor Related Protein 8	Protein Coding	Q14114	47	GC01M053243	1.620623	https://www.genecards.org/cgi-bin/carddisp.pl?gene=LRP8
PHF8	PHD Finger Protein 8	Protein Coding	Q9UPP1	47	GC0XM053936	1.619597	https://www.genecards.org/cgi-bin/carddisp.pl?gene=PHF8
DDC	Dopa Decarboxylase	Protein Coding	P20711	54	GC07M050458	1.619113	https://www.genecards.org/cgi-bin/carddisp.pl?gene=DDC
USH1C	USH1 Protein Network Component Harmonin	Protein Coding	Q9Y6N9	47	GC11M017825	1.619027	https://www.genecards.org/cgi-bin/carddisp.pl?gene=USH1C
LY86	Lymphocyte Antigen 86	Protein Coding	O95711	42	GC06P006588	1.618607	https://www.genecards.org/cgi-bin/carddisp.pl?gene=LY86
NSMCE3	NSE3 Homolog, SMC5-SMC6 Complex Component	Protein Coding	Q96MG7	41	GC15M029934	1.616815	https://www.genecards.org/cgi-bin/carddisp.pl?gene=NSMCE3
MPZ	Myelin Protein Zero	Protein Coding	P25189	50	GC01M161304	1.615431	https://www.genecards.org/cgi-bin/carddisp.pl?gene=MPZ
ZNF295-AS1	ZNF295 Antisense RNA 1	RNA Gene	Q8N0V1	24	GC21P042009	1.614735	https://www.genecards.org/cgi-bin/carddisp.pl?gene=ZNF295-AS1
ERN1	Endoplasmic Reticulum To Nucleus Signaling 1	Protein Coding	O75460	51	GC17M064039	1.614311	https://www.genecards.org/cgi-bin/carddisp.pl?gene=ERN1
TRAF6	TNF Receptor Associated Factor 6	Protein Coding	Q9Y4K3	51	GC11M036467	1.612571	https://www.genecards.org/cgi-bin/carddisp.pl?gene=TRAF6

RLBP1	Retinaldehyde Binding Protein 1	Protein Coding	P12271	48	GC15M089209	1.611214	https://www.genecards.org/cgi-bin/carddisp.pl?gene=RLBP1
ADRA1A	Adrenoceptor Alpha 1A	Protein Coding	P35348	51	GC08M026747	1.609823	https://www.genecards.org/cgi-bin/carddisp.pl?gene=ADRA1A
XK	X-Linked Kx Blood Group Antigen, Kell And VPS13A Binding Protein	Protein Coding	P51811	45	GC0XP037685	1.608406	https://www.genecards.org/cgi-bin/carddisp.pl?gene=XK
COL6A2	Collagen Type VI Alpha 2 Chain	Protein Coding	P12110	49	GC21P046098	1.605188	https://www.genecards.org/cgi-bin/carddisp.pl?gene=COL6A2
GCDH	Glutaryl-CoA Dehydrogenase	Protein Coding	Q92947	50	GC19P012891	1.604857	https://www.genecards.org/cgi-bin/carddisp.pl?gene=GCDH
ACKR2	Atypical Chemokine Receptor 2	Protein Coding	O00590	45	GC03P042804	1.60468	https://www.genecards.org/cgi-bin/carddisp.pl?gene=ACKR2
PATJ	PATJ Crumbs Cell Polarity Complex Component	Protein Coding	Q8NI35	40	GC01P061743	1.603574	https://www.genecards.org/cgi-bin/carddisp.pl?gene=PATJ
SFTPA1	Surfactant Protein A1	Protein Coding	Q8IWL2	48	GC10P104132	1.601808	https://www.genecards.org/cgi-bin/carddisp.pl?gene=SFTPA1
MIR376A2	MicroRNA 376a-2	RNA Gene		13	GC14P113594	1.601808	https://www.genecards.org/cgi-bin/carddisp.pl?gene=MIR376A2
HSPB1	Heat Shock Protein Family B (Small) Member 1	Protein Coding	P04792	55	GC07P076302	1.601279	https://www.genecards.org/cgi-bin/carddisp.pl?gene=HSPB1
NSD2	Nuclear Receptor Binding SET Domain Protein 2	Protein Coding	O96028	48	GC04P002202	1.601191	https://www.genecards.org/cgi-bin/carddisp.pl?gene=NSD2
PLEKHG1	Pleckstrin Homology And RhoGEF Domain Containing G1	Protein Coding	Q9ULL1	40	GC06P150599	1.601159	https://www.genecards.org/cgi-bin/carddisp.pl?gene=PLEKHG1

AFF2	ALF Transcription Elongation Factor 2	Protein Coding	P51816	47	GC0XP148500	1.601006	https://www.genecards.org/cgi-bin/carddisp.pl?gene=AFF2
LOC110386947	CYP19A1 Promoter I.1	Functional Element		3	GC15P057581	1.592253	https://www.genecards.org/cgi-bin/carddisp.pl?gene=LOC110386947
CYP2R1	Cytochrome P450 Family 2 Subfamily R Member 1	Protein Coding	Q6VVX0	47	GC11M014877	1.59136	https://www.genecards.org/cgi-bin/carddisp.pl?gene=CYP2R1
CIITA	Class II Major Histocompatibility Complex Transactivator	Protein Coding	P33076	48	GC16P052668	1.5911	https://www.genecards.org/cgi-bin/carddisp.pl?gene=CIITA
CAVIN1	Caveolae Associated Protein 1	Protein Coding	Q6NZI2	46	GC17M064168	1.590063	https://www.genecards.org/cgi-bin/carddisp.pl?gene=CAVIN1
MIR4713	MicroRNA 4713	RNA Gene		12	GC15P051242	1.589715	https://www.genecards.org/cgi-bin/carddisp.pl?gene=MIR4713
TCTN1	Tectonic Family Member 1	Protein Coding	Q2MV58	44	GC12P110614	1.58822	https://www.genecards.org/cgi-bin/carddisp.pl?gene=TCTN1
TALDO1	Transaldolase 1	Protein Coding	P37837	51	GC11P004465	1.58772	https://www.genecards.org/cgi-bin/carddisp.pl?gene=TALDO1
EBAG9	Estrogen Receptor Binding Site Associated Antigen 9	Protein Coding	O00559	41	GC08P109536	1.586042	https://www.genecards.org/cgi-bin/carddisp.pl?gene=EBAG9
FLG	Filaggrin	Protein Coding	P20930	46	GC01M157608	1.585234	https://www.genecards.org/cgi-bin/carddisp.pl?gene=FLG
HYAL1	Hyaluronidase 1	Protein Coding	Q12794	51	GC03M050299	1.585144	https://www.genecards.org/cgi-bin/carddisp.pl?gene=HYAL1
HDAC6	Histone Deacetylase 6	Protein Coding	Q9UBN7	57	GC0XP048801	1.584348	https://www.genecards.org/cgi-bin/carddisp.pl?gene=HDAC6

ARNT	Aryl Hydrocarbon Receptor Nuclear Translocator	Protein Coding	P27540	50	GC01M150809	1.583512	https://www.genecards.org/cgi-bin/carddisp.pl?gene=ARNT
CHM	CHM Rab Escort Protein	Protein Coding	P24386	46	GC0XM085861	1.583385	https://www.genecards.org/cgi-bin/carddisp.pl?gene=CHM
C3AR1	Complement C3a Receptor 1	Protein Coding	Q16581	48	GC12M008745	1.583037	https://www.genecards.org/cgi-bin/carddisp.pl?gene=C3AR1
GYS2	Glycogen Synthase 2	Protein Coding	P54840	49	GC12M026119	1.582909	https://www.genecards.org/cgi-bin/carddisp.pl?gene=GYS2
MTRNR2L7	MT-RNR2 Like 7 (Pseudogene)	Pseudogene	P0CJ74	16	GC10M037601	1.582631	https://www.genecards.org/cgi-bin/carddisp.pl?gene=MTRNR2L7
FOLR2	Folate Receptor Beta	Protein Coding	P14207	47	GC11P072216	1.582128	https://www.genecards.org/cgi-bin/carddisp.pl?gene=FOLR2
PENK	Proenkephalin	Protein Coding	P01210	43	GC08M056436	1.581554	https://www.genecards.org/cgi-bin/carddisp.pl?gene=PENK
MSH6	MutS Homolog 6	Protein Coding	P52701	53	GC02P047695	1.57991	https://www.genecards.org/cgi-bin/carddisp.pl?gene=MSH6
SGCB	Sarcoglycan Beta	Protein Coding	Q16585	44	GC04M052019	1.579728	https://www.genecards.org/cgi-bin/carddisp.pl?gene=SGCB
SMARCAL1	SWI/SNF Related, Matrix Associated, Actin Dependent Regulator Of Chromatin, Subfamily A Like 1	Protein Coding	Q9NZC9	50	GC02P216412	1.579704	https://www.genecards.org/cgi-bin/carddisp.pl?gene=SMARCAL1
PRDM16-DT	PRDM16 Divergent Transcript	RNA Gene		21	GC01M010690	1.5792	https://www.genecards.org/cgi-bin/carddisp.pl?gene=PRDM16-DT
CSK	C-Terminal Src Kinase	Protein Coding	P41240	51	GC15P074782	1.578669	https://www.genecards.org/cgi-bin/carddisp.pl?gene=CSK

UGT1A9	UDP Glucuronosyltransferase Family 1 Member A9	Protein Coding	O60656	48	GC02P233671	1.578373	https://www.genecards.org/cgi-bin/carddisp.pl?gene=UGT1A9
HOTAIRM1	HOXA Transcript Antisense RNA, Myeloid-Specific 1	RNA Gene		21	GC07P027095	1.577323	https://www.genecards.org/cgi-bin/carddisp.pl?gene=HOTAIRM1
ACVR1B	Activin A Receptor Type 1B	Protein Coding	P36896	51	GC12P051951	1.577165	https://www.genecards.org/cgi-bin/carddisp.pl?gene=ACVR1B
CPQ	Carboxypeptidase Q	Protein Coding	Q9Y646	42	GC08P096645	1.57478	https://www.genecards.org/cgi-bin/carddisp.pl?gene=CPQ
PTGDS	Prostaglandin D2 Synthase	Protein Coding	P41222	50	GC09P137573	1.574164	https://www.genecards.org/cgi-bin/carddisp.pl?gene=PTGDS
LOC108663993	Ataxin 1 Repeat Instability Region	Functional Element		4	GC06P016675	1.573298	https://www.genecards.org/cgi-bin/carddisp.pl?gene=LOC108663993
BSG	Basigin (Ok Blood Group)	Protein Coding	P35613	49	GC19P000571	1.571798	https://www.genecards.org/cgi-bin/carddisp.pl?gene=BSG
GNAQ	G Protein Subunit Alpha Q	Protein Coding	P50148	53	GC09M077716	1.571357	https://www.genecards.org/cgi-bin/carddisp.pl?gene=GNAQ
STIN2-VNTR	Serotonin Transporter Intronic VNTR Enhancer	Functional Element		3	GC17P030221	1.571016	https://www.genecards.org/cgi-bin/carddisp.pl?gene=STIN2-VNTR
AXL	AXL Receptor Tyrosine Kinase	Protein Coding	P30530	55	GC19P041219	1.570861	https://www.genecards.org/cgi-bin/carddisp.pl?gene=AXL
ROBO1	Roundabout Guidance Receptor 1	Protein Coding	Q9Y6N7	48	GC03M078597	1.570271	https://www.genecards.org/cgi-bin/carddisp.pl?gene=ROBO1
MIR299	MicroRNA 299	RNA Gene		18	GC14P113585	1.569438	https://www.genecards.org/cgi-bin/carddisp.pl?gene=MIR299

TLE1	TLE Family Member 1, Transcriptional Corepressor	Protein Coding	Q04724	48	GC09M081583	1.569338	https://www.genecards.org/cgi-bin/carddisp.pl?gene=TLE1
FYN	FYN Proto-Oncogene, Src Family Tyrosine Kinase	Protein Coding	P06241	52	GC06M111660	1.568314	https://www.genecards.org/cgi-bin/carddisp.pl?gene=FYN
SP1	Sp1 Transcription Factor	Protein Coding	P08047	50	GC12P053380	1.564526	https://www.genecards.org/cgi-bin/carddisp.pl?gene=SP1
DGUOK	Deoxyguanosine Kinase	Protein Coding	Q16854	48	GC02P073926	1.562842	https://www.genecards.org/cgi-bin/carddisp.pl?gene=DGUOK
MOK	MOK Protein Kinase	Protein Coding	Q9UQ07	40	GC14M102214	1.562836	https://www.genecards.org/cgi-bin/carddisp.pl?gene=MOK
MIR369	MicroRNA 369	RNA Gene		20	GC14P113592	1.562275	https://www.genecards.org/cgi-bin/carddisp.pl?gene=MIR369
MIR3144	MicroRNA 3144	RNA Gene		13	GC06P120015	1.561952	https://www.genecards.org/cgi-bin/carddisp.pl?gene=MIR3144
ENSG00000145075	Novel Transcript	RNA Gene		10	GC03M180615	1.561952	https://www.genecards.org/cgi-bin/carddisp.pl?gene=ENSG00000145075
BAK1	BCL2 Antagonist/Killer 1	Protein Coding	Q16611	49	GC06M033572	1.560394	https://www.genecards.org/cgi-bin/carddisp.pl?gene=BAK1
LOC106780800	CYP21A2 Recombination Region	Functional Element		4	GC06P114403	1.560302	https://www.genecards.org/cgi-bin/carddisp.pl?gene=LOC106780800
UMOD	Uromodulin	Protein Coding	P07911	47	GC16M020344	1.559346	https://www.genecards.org/cgi-bin/carddisp.pl?gene=UMOD
MTHFD2	Methylenetetrahydrofolate Dehydrogenase (NADP+ Dependent) 2, Methenyltetrahydrofolate Cyclohydrolase	Protein Coding	P13995	45	GC02P074186	1.558508	https://www.genecards.org/cgi-bin/carddisp.pl?gene=MTHFD2

LIG4	DNA Ligase 4	Protein Coding	P49917	51	GC13M108207	1.557568	https://www.genecards.org/cgi-bin/carddisp.pl?gene=LIG4
MIR551A	MicroRNA 551a	RNA Gene		19	GC01M003560	1.557453	https://www.genecards.org/cgi-bin/carddisp.pl?gene=MIR551A
ATP4A	ATPase H+/K+ Transporting Subunit Alpha	Protein Coding	P20648	45	GC19M083298	1.554834	https://www.genecards.org/cgi-bin/carddisp.pl?gene=ATP4A
IL17RA	Interleukin 17 Receptor A	Protein Coding	Q96F46	51	GC22P055916	1.552844	https://www.genecards.org/cgi-bin/carddisp.pl?gene=IL17RA
OXA1L	OXA1L Mitochondrial Inner Membrane Protein	Protein Coding	Q15070	42	GC14P022766	1.55179	https://www.genecards.org/cgi-bin/carddisp.pl?gene=OXA1L
OGT	O-Linked N-Acetylglucosamine (GlcNAc) Transferase	Protein Coding	O15294	50	GC0XP071597	1.550092	https://www.genecards.org/cgi-bin/carddisp.pl?gene=OGT
DGKE	Diacylglycerol Kinase Epsilon	Protein Coding	P52429	51	GC17P056834	1.549244	https://www.genecards.org/cgi-bin/carddisp.pl?gene=DGKE
CPOX	Coproporphyrinogen Oxidase	Protein Coding	P36551	48	GC03M098576	1.549008	https://www.genecards.org/cgi-bin/carddisp.pl?gene=CPOX
GSTA4	Glutathione S-Transferase Alpha 4	Protein Coding	O15217	45	GC06M052977	1.548813	https://www.genecards.org/cgi-bin/carddisp.pl?gene=GSTA4
F2RL1	F2R Like Trypsin Receptor 1	Protein Coding	P55085	48	GC05P076818	1.546837	https://www.genecards.org/cgi-bin/carddisp.pl?gene=F2RL1
EEF1A2	Eukaryotic Translation Elongation Factor 1 Alpha 2	Protein Coding	Q05639	51	GC20M063488	1.546707	https://www.genecards.org/cgi-bin/carddisp.pl?gene=EEF1A2
SATB1	SATB Homeobox 1	Protein Coding	Q01826	48	GC03M024518	1.544915	https://www.genecards.org/cgi-bin/carddisp.pl?gene=SATB1

SRD5A2	Steroid 5 Alpha-Reductase 2	Protein Coding	P31213	46	GC02M031522	1.541573	https://www.genecards.org/cgi-bin/carddisp.pl?gene=SRD5A2
PTDSS2	Phosphatidylserine Synthase 2	Protein Coding	Q9BVG9	40	GC11P000448	1.540402	https://www.genecards.org/cgi-bin/carddisp.pl?gene=PTDSS2
LARS1	Leucyl-TRNA Synthetase 1	Protein Coding	Q9P2J5	49	GC05M146114	1.539995	https://www.genecards.org/cgi-bin/carddisp.pl?gene=LARS1
ENSG00000274760		RNA Gene		9	GC20P049279	1.539168	https://www.genecards.org/cgi-bin/carddisp.pl?gene=ENSG00000274760
ENSG00000277967		RNA Gene		9	GC20P049280	1.539168	https://www.genecards.org/cgi-bin/carddisp.pl?gene=ENSG00000277967
ETFB	Electron Transfer Flavoprotein Subunit Beta	Protein Coding	P38117	48	GC19M051345	1.538145	https://www.genecards.org/cgi-bin/carddisp.pl?gene=ETFB
TLR8	Toll Like Receptor 8	Protein Coding	Q9NR97	53	GC0XP012924	1.536708	https://www.genecards.org/cgi-bin/carddisp.pl?gene=TLR8
BICD1	BICD Cargo Adaptor 1	Protein Coding	Q96G01	42	GC12P033080	1.536458	https://www.genecards.org/cgi-bin/carddisp.pl?gene=BICD1
SPTLC1	Serine Palmitoyltransferase Long Chain Base Subunit 1	Protein Coding	O15269	50	GC09M104371	1.535976	https://www.genecards.org/cgi-bin/carddisp.pl?gene=SPTLC1
CD69	CD69 Molecule	Protein Coding	Q07108	46	GC12M025966	1.53468	https://www.genecards.org/cgi-bin/carddisp.pl?gene=CD69
VCL	Vinculin	Protein Coding	P18206	52	GC10P073995	1.53384	https://www.genecards.org/cgi-bin/carddisp.pl?gene=VCL
FAM161A	FAM161 Centrosomal Protein A	Protein Coding	Q3B820	39	GC02M061792	1.533759	https://www.genecards.org/cgi-bin/carddisp.pl?gene=FAM161A

ARID4A	AT-Rich Interaction Domain 4A	Protein Coding	P29374	42	GC14P058298	1.533083	https://www.genecards.org/cgi-bin/carddisp.pl?gene=ARID4A
BIRC2	Baculoviral IAP Repeat Containing 2	Protein Coding	Q13490	51	GC11P102347	1.532599	https://www.genecards.org/cgi-bin/carddisp.pl?gene=BIRC2
BBOX1	Gamma-Butyrobetaine Hydroxylase 1	Protein Coding	O75936	45	GC11P027019	1.531329	https://www.genecards.org/cgi-bin/carddisp.pl?gene=BBOX1
PSEN1	Presenilin 1	Protein Coding	P49768	56	GC14P073136	1.530802	https://www.genecards.org/cgi-bin/carddisp.pl?gene=PSEN1
BCL2L1	BCL2 Like 1	Protein Coding	Q07817	52	GC20M031664	1.530341	https://www.genecards.org/cgi-bin/carddisp.pl?gene=BCL2L1
SNHG6	Small Nucleolar RNA Host Gene 6	RNA Gene		21	GC08M066921	1.530336	https://www.genecards.org/cgi-bin/carddisp.pl?gene=SNHG6
CPS1-IT1	CPS1 Intronic Transcript 1	RNA Gene		19	GC02P210617	1.530336	https://www.genecards.org/cgi-bin/carddisp.pl?gene=CPS1-IT1
BANCR	BRAF-Activated Non-Protein Coding RNA	RNA Gene		15	GC09M069296	1.530336	https://www.genecards.org/cgi-bin/carddisp.pl?gene=BANCR
ELOVL5	ELOVL Fatty Acid Elongase 5	Protein Coding	Q9NYP7	47	GC06M053267	1.527747	https://www.genecards.org/cgi-bin/carddisp.pl?gene=ELOVL5
SLC34A1	Solute Carrier Family 34 Member 1	Protein Coding	Q06495	48	GC05P180010	1.527509	https://www.genecards.org/cgi-bin/carddisp.pl?gene=SLC34A1
COL10A1	Collagen Type X Alpha 1 Chain	Protein Coding	Q03692	45	GC06M116118	1.525132	https://www.genecards.org/cgi-bin/carddisp.pl?gene=COL10A1
NDUFS3	NADH:Ubiquinone Oxidoreductase Core Subunit S3	Protein Coding	O75489	50	GC11P047575	1.524978	https://www.genecards.org/cgi-bin/carddisp.pl?gene=NDUFS3

MIR4421	MicroRNA 4421	RNA Gene		12	GC01P051059	1.522476	https://www.genecards.org/cgi-bin/carddisp.pl?gene=MIR4421
CTNNA1	Catenin Alpha 1	Protein Coding	P35221	51	GC05P138627	1.520414	https://www.genecards.org/cgi-bin/carddisp.pl?gene=CTNNA1
SLC46A1	Solute Carrier Family 46 Member 1	Protein Coding	Q96NT5	46	GC17M063258	1.519754	https://www.genecards.org/cgi-bin/carddisp.pl?gene=SLC46A1
HIC1	HIC ZBTB Transcriptional Repressor 1	Protein Coding	Q14526	46	GC17P002054	1.519019	https://www.genecards.org/cgi-bin/carddisp.pl?gene=HIC1
DSG2	Desmoglein 2	Protein Coding	Q14126	50	GC18P031498	1.518908	https://www.genecards.org/cgi-bin/carddisp.pl?gene=DSG2
RAD51	RAD51 Recombinase	Protein Coding	Q06609	54	GC15P040694	1.518907	https://www.genecards.org/cgi-bin/carddisp.pl?gene=RAD51
CPS1	Carbamoyl-Phosphate Synthase 1	Protein Coding	P31327	53	GC02P210477	1.518799	https://www.genecards.org/cgi-bin/carddisp.pl?gene=CPS1
TGFBR3	Transforming Growth Factor Beta Receptor 3	Protein Coding	Q03167	51	GC01M091680	1.518363	https://www.genecards.org/cgi-bin/carddisp.pl?gene=TGFBR3
SLC29A1	Solute Carrier Family 29 Member 1 (Augustine Blood Group)	Protein Coding	Q99808	52	GC06P044219	1.518259	https://www.genecards.org/cgi-bin/carddisp.pl?gene=SLC29A1
MBP	Myelin Basic Protein	Protein Coding	P02686	48	GC18M076978	1.517932	https://www.genecards.org/cgi-bin/carddisp.pl?gene=MBP
ERP44	Endoplasmic Reticulum Protein 44	Protein Coding	Q9BS26	46	GC09M099979	1.516566	https://www.genecards.org/cgi-bin/carddisp.pl?gene=ERP44
POU5F1	POU Class 5 Homeobox 1	Protein Coding	Q01860	51	GC06M083899	1.515832	https://www.genecards.org/cgi-bin/carddisp.pl?gene=POU5F1

IQGAP1	IQ Motif Containing GTPase Activating Protein 1	Protein Coding	P46940	47	GC15P090388	1.514715	https://www.genecards.org/cgi-bin/carddisp.pl?gene=IQGAP1
NSMF	NMDA Receptor Synaptonuclear Signaling And Neuronal Migration Factor	Protein Coding	Q6X4W1	46	GC09M137447	1.514593	https://www.genecards.org/cgi-bin/carddisp.pl?gene=NSMF
BCKDHA	Branched Chain Keto Acid Dehydrogenase E1 Subunit Alpha	Protein Coding	P12694	48	GC19P089607	1.51014	https://www.genecards.org/cgi-bin/carddisp.pl?gene=BCKDHA
SMAD1	SMAD Family Member 1	Protein Coding	Q15797	48	GC04P145481	1.505424	https://www.genecards.org/cgi-bin/carddisp.pl?gene=SMAD1
TBP	TATA-Box Binding Protein	Protein Coding	P20226	53	GC06P170554	1.505291	https://www.genecards.org/cgi-bin/carddisp.pl?gene=TBP
TCL6	T Cell Leukemia/Lymphoma 6	RNA Gene		28	GC14P095650	1.505291	https://www.genecards.org/cgi-bin/carddisp.pl?gene=TCL6
AIM2	Absent In Melanoma 2	Protein Coding	O14862	45	GC01M159062	1.505032	https://www.genecards.org/cgi-bin/carddisp.pl?gene=AIM2
PRKAG1	Protein Kinase AMP-Activated Non-Catalytic Subunit Gamma 1	Protein Coding	P54619	51	GC12M049002	1.503307	https://www.genecards.org/cgi-bin/carddisp.pl?gene=PRKAG1
CD19	CD19 Molecule	Protein Coding	P15391	55	GC16P053997	1.502107	https://www.genecards.org/cgi-bin/carddisp.pl?gene=CD19
ADH1A	Alcohol Dehydrogenase 1A (Class I), Alpha Polypeptide	Protein Coding	P07327	46	GC04M099276	1.501972	https://www.genecards.org/cgi-bin/carddisp.pl?gene=ADH1A
BMP1	Bone Morphogenetic Protein 1	Protein Coding	P13497	53	GC08P022165	1.501147	https://www.genecards.org/cgi-bin/carddisp.pl?gene=BMP1
PDE4B	Phosphodiesterase 4B	Protein Coding	Q07343	48	GC01P065792	1.500774	https://www.genecards.org/cgi-bin/carddisp.pl?gene=PDE4B

CAMP	Cathelicidin Antimicrobial Peptide	Protein Coding	P49913	44	GC03P053872	1.499521	https://www.genecards.org/cgi-bin/carddisp.pl?gene=CAMP
THBS2	Thrombospondin 2	Protein Coding	P35442	50	GC06M169215	1.498324	https://www.genecards.org/cgi-bin/carddisp.pl?gene=THBS2
TGFBRAP1	Transforming Growth Factor Beta Receptor Associated Protein 1	Protein Coding	Q8WUH2	39	GC02M105250	1.49744	https://www.genecards.org/cgi-bin/carddisp.pl?gene=TGFBRAP1
NARS2	Asparaginyl-TRNA Synthetase 2, Mitochondrial	Protein Coding	Q96I59	47	GC11M078435	1.496447	https://www.genecards.org/cgi-bin/carddisp.pl?gene=NARS2
EFNB2	Ephrin B2	Protein Coding	P52799	48	GC13M106489	1.496301	https://www.genecards.org/cgi-bin/carddisp.pl?gene=EFNB2
MIR4530	MicroRNA 4530	RNA Gene		13	GC19M082535	1.496037	https://www.genecards.org/cgi-bin/carddisp.pl?gene=MIR4530
FSCN1	Fascin Actin-Bundling Protein 1	Protein Coding	Q16658	48	GC07P005592	1.495508	https://www.genecards.org/cgi-bin/carddisp.pl?gene=FSCN1
CPB2	Carboxypeptidase B2	Protein Coding	Q96IY4	48	GC13M046053	1.495409	https://www.genecards.org/cgi-bin/carddisp.pl?gene=CPB2
CIDEB	Cell Death Inducing DFFA Like Effector B	Protein Coding	Q9UHD4	40	GC14M024305	1.495409	https://www.genecards.org/cgi-bin/carddisp.pl?gene=CIDEB
AP3D1	Adaptor Related Protein Complex 3 Subunit Delta 1	Protein Coding	O14617	46	GC19M010520	1.494691	https://www.genecards.org/cgi-bin/carddisp.pl?gene=AP3D1
RNF2	Ring Finger Protein 2	Protein Coding	Q99496	49	GC01P185045	1.493943	https://www.genecards.org/cgi-bin/carddisp.pl?gene=RNF2
HSD3B1	Hydroxy-Delta-5-Steroid Dehydrogenase, 3 Beta- And Steroid Delta-Isomerase 1	Protein Coding	P14060	47	GC01P119507	1.493427	https://www.genecards.org/cgi-bin/carddisp.pl?gene=HSD3B1

PTGER1	Prostaglandin E Receptor 1	Protein Coding	P34995	45	GC19M015927	1.492647	https://www.genecards.org/cgi-bin/carddisp.pl?gene=PTGER1
BAG3	BAG Cochaperone 3	Protein Coding	O95817	47	GC10P119651	1.488903	https://www.genecards.org/cgi-bin/carddisp.pl?gene=BAG3
NOTCH3	Notch Receptor 3	Protein Coding	Q9UM47	55	GC19M015159	1.488397	https://www.genecards.org/cgi-bin/carddisp.pl?gene=NOTCH3
PAX1	Paired Box 1	Protein Coding	P15863	47	GC20P021705	1.488083	https://www.genecards.org/cgi-bin/carddisp.pl?gene=PAX1
CAPN3	Calpain 3	Protein Coding	P20807	52	GC15P042359	1.487268	https://www.genecards.org/cgi-bin/carddisp.pl?gene=CAPN3
TMEM107	Transmembrane Protein 107	Protein Coding	Q6UX40	41	GC17M014445	1.487248	https://www.genecards.org/cgi-bin/carddisp.pl?gene=TMEM107
SEMA3B	Semaphorin 3B	Protein Coding	Q13214	44	GC03P050267	1.485624	https://www.genecards.org/cgi-bin/carddisp.pl?gene=SEMA3B
MIR519B	MicroRNA 519b	RNA Gene		18	GC19P053695	1.484942	https://www.genecards.org/cgi-bin/carddisp.pl?gene=MIR519B
ADCYAP1	Adenylate Cyclase Activating Polypeptide 1	Protein Coding	P18509	45	GC18P000895	1.481126	https://www.genecards.org/cgi-bin/carddisp.pl?gene=ADCYAP1
YY1	YY1 Transcription Factor	Protein Coding	P25490	52	GC14P100238	1.480991	https://www.genecards.org/cgi-bin/carddisp.pl?gene=YY1
POMT1	Protein O-Mannosyltransferase 1	Protein Coding	Q9Y6A1	49	GC09P131502	1.478053	https://www.genecards.org/cgi-bin/carddisp.pl?gene=POMT1
F2RL2	Coagulation Factor II Thrombin Receptor Like 2	Protein Coding	O00254	45	GC05M076615	1.477884	https://www.genecards.org/cgi-bin/carddisp.pl?gene=F2RL2

TNFRSF13B	TNF Receptor Superfamily Member 13B	Protein Coding	O14836	51	GC17M016929	1.476466	https://www.genecards.org/cgi-bin/carddisp.pl?gene=TNFRSF13B
ABCB5	ATP Binding Cassette Subfamily B Member 5	Protein Coding	Q2M3G0	47	GC07P020615	1.476186	https://www.genecards.org/cgi-bin/carddisp.pl?gene=ABCB5
CPEB4	Cytoplasmic Polyadenylation Element Binding Protein 4	Protein Coding	Q17RY0	42	GC05P173888	1.476186	https://www.genecards.org/cgi-bin/carddisp.pl?gene=CPEB4
NR4A3	Nuclear Receptor Subfamily 4 Group A Member 3	Protein Coding	Q92570	47	GC09P099821	1.475935	https://www.genecards.org/cgi-bin/carddisp.pl?gene=NR4A3
MTHFSD	Methenyltetrahydrofolate Synthetase Domain Containing	Protein Coding	Q2M296	38	GC16M086530	1.473741	https://www.genecards.org/cgi-bin/carddisp.pl?gene=MTHFSD
RAD50	RAD50 Double Strand Break Repair Protein	Protein Coding	Q92878	53	GC05P132556	1.472326	https://www.genecards.org/cgi-bin/carddisp.pl?gene=RAD50
H3C1	H3 Clustered Histone 1	Protein Coding	P68431	42	GC06P111760	1.472326	https://www.genecards.org/cgi-bin/carddisp.pl?gene=H3C1
LOC126806659	BRD4-Independent Group 4 Enhancer GRCh37_chr3:41274918-41276117	Functional Element		3	GC03P041233	1.472326	https://www.genecards.org/cgi-bin/carddisp.pl?gene=LOC126806659
DYNC2H1	Dynein Cytoplasmic 2 Heavy Chain 1	Protein Coding	Q8NCM8	42	GC11P103109	1.471965	https://www.genecards.org/cgi-bin/carddisp.pl?gene=DYNC2H1
CD81	CD81 Molecule	Protein Coding	P60033	51	GC11P004550	1.471528	https://www.genecards.org/cgi-bin/carddisp.pl?gene=CD81
NEXMIF	Neurite Extension And Migration Factor	Protein Coding	Q5QGS0	36	GC0XM074760	1.467486	https://www.genecards.org/cgi-bin/carddisp.pl?gene=NEXMIF
SAMD4A	Sterile Alpha Motif Domain Containing 4A	Protein Coding	Q9UPU9	40	GC14P054567	1.466235	https://www.genecards.org/cgi-bin/carddisp.pl?gene=SAMD4A

PSMC3	Proteasome 26S Subunit, ATPase 3	Protein Coding	P17980	48	GC11M113172	1.465309	https://www.genecards.org/cgi-bin/carddisp.pl?gene=PSMC3
HEXA	Hexosaminidase Subunit Alpha	Protein Coding	P06865	51	GC15M072340	1.465127	https://www.genecards.org/cgi-bin/carddisp.pl?gene=HEXA
COL4A6	Collagen Type IV Alpha 6 Chain	Protein Coding	Q14031	47	GC0XM108155	1.464909	https://www.genecards.org/cgi-bin/carddisp.pl?gene=COL4A6
NRXN2	Neurexin 2	Protein Coding	Q9P2S2	43	GC11M113476	1.46436	https://www.genecards.org/cgi-bin/carddisp.pl?gene=NRXN2
RS1	Retinoschisin 1	Protein Coding	O15537	44	GC0XM018639	1.463458	https://www.genecards.org/cgi-bin/carddisp.pl?gene=RS1
MIR3663	MicroRNA 3663	RNA Gene		13	GC10M117167	1.462685	https://www.genecards.org/cgi-bin/carddisp.pl?gene=MIR3663
TUBG1	Tubulin Gamma 1	Protein Coding	P23258	52	GC17P042609	1.462544	https://www.genecards.org/cgi-bin/carddisp.pl?gene=TUBG1
CMKLR2-AS	CMKLR2 Antisense RNA	RNA Gene		14	GC02P206352	1.462544	https://www.genecards.org/cgi-bin/carddisp.pl?gene=CMKLR2-AS
TAT	Tyrosine Aminotransferase	Protein Coding	P17735	48	GC16M071565	1.460603	https://www.genecards.org/cgi-bin/carddisp.pl?gene=TAT
KIF11	Kinesin Family Member 11	Protein Coding	P52732	52	GC10P092574	1.459547	https://www.genecards.org/cgi-bin/carddisp.pl?gene=KIF11
ENPEP	Glutamyl Aminopeptidase	Protein Coding	Q07075	51	GC04P110365	1.45743	https://www.genecards.org/cgi-bin/carddisp.pl?gene=ENPEP
ECE1	Endothelin Converting Enzyme 1	Protein Coding	P42892	53	GC01M021217	1.456801	https://www.genecards.org/cgi-bin/carddisp.pl?gene=ECE1

LINC01618	Long Intergenic Non-Protein Coding RNA 1618	RNA Gene		15	GC04P052908	1.45606 3	https://www.genecards.org/cgi-bin/carddisp.pl?gene=LINC01618
MIR4721	MicroRNA 4721	RNA Gene		13	GC16M04149 7	1.45521 1	https://www.genecards.org/cgi-bin/carddisp.pl?gene=MIR4721
TRHR	Thyrotropin Releasing Hormone Receptor	Protein Coding	P34981	46	GC08P109084	1.45407 9	https://www.genecards.org/cgi-bin/carddisp.pl?gene=TRHR
ENO1	Enolase 1	Protein Coding	P06733	51	GC01M00886 1	1.45402 8	https://www.genecards.org/cgi-bin/carddisp.pl?gene=ENO1
HTR5A	5-Hydroxytryptamine Receptor 5A	Protein Coding	P47898	45	GC07P155070	1.45373 1	https://www.genecards.org/cgi-bin/carddisp.pl?gene=HTR5A
ANGPTL7	Angiopoietin Like 7	Protein Coding	O43827	44	GC01P011189	1.45208	https://www.genecards.org/cgi-bin/carddisp.pl?gene=ANGPTL7
PRICKLE1	Prickle Planar Cell Polarity Protein 1	Protein Coding	Q96MT3	49	GC12M04245 6	1.45123 2	https://www.genecards.org/cgi-bin/carddisp.pl?gene=PRICKLE1
A2M	Alpha-2-Macroglobulin	Protein Coding	P01023	50	GC12M00906 7	1.45065	https://www.genecards.org/cgi-bin/carddisp.pl?gene=A2M
THBS4	Thrombospondin 4	Protein Coding	P35443	46	GC05P079991	1.45023 8	https://www.genecards.org/cgi-bin/carddisp.pl?gene=THBS4
PIK3CD	Phosphatidylinositol-4,5-Bisphosphate 3-Kinase Catalytic Subunit Delta	Protein Coding	O00329	57	GC01P009628	1.45004 7	https://www.genecards.org/cgi-bin/carddisp.pl?gene=PIK3CD
CD59	CD59 Molecule (CD59 Blood Group)	Protein Coding	P13987	50	GC11M03370 4	1.44945 5	https://www.genecards.org/cgi-bin/carddisp.pl?gene=CD59
SHANK3	SH3 And Multiple Ankyrin Repeat Domains 3	Protein Coding	Q9BYB0	42	GC22P057033	1.44931 2	https://www.genecards.org/cgi-bin/carddisp.pl?gene=SHANK3

TUFM	Tu Translation Elongation Factor, Mitochondrial	Protein Coding	P49411	48	GC16M041496	1.44772	https://www.genecards.org/cgi-bin/carddisp.pl?gene=TUFM
CMKLR2	Chemerin Chemokine-Like Receptor 2	Protein Coding	P46091	41	GC02M206176	1.447625	https://www.genecards.org/cgi-bin/carddisp.pl?gene=CMKLR2
ARHGAP35	Rho GTPase Activating Protein 35	Protein Coding	Q9NRY4	44	GC19P046860	1.444809	https://www.genecards.org/cgi-bin/carddisp.pl?gene=ARHGAP35
MIR346	MicroRNA 346	RNA Gene		21	GC10M086264	1.443575	https://www.genecards.org/cgi-bin/carddisp.pl?gene=MIR346
PMP22	Peripheral Myelin Protein 22	Protein Coding	Q01453	47	GC17M015229	1.442415	https://www.genecards.org/cgi-bin/carddisp.pl?gene=PMP22
NEK8	NIMA Related Kinase 8	Protein Coding	Q86SG6	45	GC17P028725	1.441539	https://www.genecards.org/cgi-bin/carddisp.pl?gene=NEK8
TCTN2	Tectonic Family Member 2	Protein Coding	Q96GX1	44	GC12P123671	1.441539	https://www.genecards.org/cgi-bin/carddisp.pl?gene=TCTN2
PAK1	P21 (RAC1) Activated Kinase 1	Protein Coding	Q13153	54	GC11M113975	1.44026	https://www.genecards.org/cgi-bin/carddisp.pl?gene=PAK1
FOXE3	Forkhead Box E3	Protein Coding	Q13461	40	GC01P047416	1.43947	https://www.genecards.org/cgi-bin/carddisp.pl?gene=FOXE3
PDHA1	Pyruvate Dehydrogenase E1 Subunit Alpha 1	Protein Coding	P08559	51	GC0XP019343	1.439096	https://www.genecards.org/cgi-bin/carddisp.pl?gene=PDHA1
MIR516A1	MicroRNA 516a-1	RNA Gene		18	GC19P053756	1.438321	https://www.genecards.org/cgi-bin/carddisp.pl?gene=MIR516A1
MIR516A2	MicroRNA 516a-2	RNA Gene		16	GC19P053761	1.438321	https://www.genecards.org/cgi-bin/carddisp.pl?gene=MIR516A2

MIR4435-2HG	MIR4435-2 Host Gene	RNA Gene		20	GC02M112207	1.433549	https://www.genecards.org/cgi-bin/carddisp.pl?gene=MIR4435-2HG
SGCZ	Sarcoglycan Zeta	Protein Coding	Q96LD1	39	GC08M014089	1.433224	https://www.genecards.org/cgi-bin/carddisp.pl?gene=SGCZ
STAT5B	Signal Transducer And Activator Of Transcription 5B	Protein Coding	P51692	54	GC17M042199	1.431767	https://www.genecards.org/cgi-bin/carddisp.pl?gene=STAT5B
NLGN2	Neuroligin 2	Protein Coding	Q8NFZ4	45	GC17P014010	1.430892	https://www.genecards.org/cgi-bin/carddisp.pl?gene=NLGN2
RIOX2	Ribosomal Oxygenase 2	Protein Coding	Q8IUF8	41	GC03M097942	1.429364	https://www.genecards.org/cgi-bin/carddisp.pl?gene=RIOX2
CCDC150	Coiled-Coil Domain Containing 150	Protein Coding	Q8NCX0	36	GC02P196639	1.429364	https://www.genecards.org/cgi-bin/carddisp.pl?gene=CCDC150
LINC00173	Long Intergenic Non-Protein Coding RNA 173	RNA Gene	Q6ZV60	23	GC12P116533	1.429364	https://www.genecards.org/cgi-bin/carddisp.pl?gene=LINC00173
MIR219A1	MicroRNA 219a-1	RNA Gene		22	GC06P033207	1.429364	https://www.genecards.org/cgi-bin/carddisp.pl?gene=MIR219A1
HAGLR	HOXD Antisense Growth-Associated Long Non-Coding RNA	RNA Gene		19	GC02M176172	1.429364	https://www.genecards.org/cgi-bin/carddisp.pl?gene=HAGLR
GATA3-AS1	GATA3 Antisense RNA 1	RNA Gene		18	GC10M008016	1.429364	https://www.genecards.org/cgi-bin/carddisp.pl?gene=GATA3-AS1
KRTAP5-AS1	KRTAP5-1/KRTAP5-2 Antisense RNA 1	RNA Gene		18	GC11P001571	1.429364	https://www.genecards.org/cgi-bin/carddisp.pl?gene=KRTAP5-AS1
NNT-AS1	NNT Antisense RNA 1	RNA Gene		16	GC05M044570	1.429364	https://www.genecards.org/cgi-bin/carddisp.pl?gene=NNT-AS1

PCBP2-OT1	PCBP2 Overlapping Transcript 1	RNA Gene		11	GC12P053464	1.429364	https://www.genecards.org/cgi-bin/carddisp.pl?gene=PCBP2-OT1
LOC108663996	TATA-Box Binding Protein Repeat Instability Region	Functional Element		4	GC06P170561	1.429364	https://www.genecards.org/cgi-bin/carddisp.pl?gene=LOC108663996
LOC109504725	Androgen Receptor Repeat Instability Region	Functional Element		4	GC0XP067546	1.429364	https://www.genecards.org/cgi-bin/carddisp.pl?gene=LOC109504725
CHN2	Chimerin 2	Protein Coding	P52757	46	GC07P029186	1.429085	https://www.genecards.org/cgi-bin/carddisp.pl?gene=CHN2
TAF1	TATA-Box Binding Protein Associated Factor 1	Protein Coding	P21675	52	GC0XP071366	1.428956	https://www.genecards.org/cgi-bin/carddisp.pl?gene=TAF1
BEST1	Bestrophin 1	Protein Coding	O76090	47	GC11P061949	1.428956	https://www.genecards.org/cgi-bin/carddisp.pl?gene=BEST1
AMY2A	Amylase Alpha 2A	Protein Coding	P04746	42	GC01P103616	1.425478	https://www.genecards.org/cgi-bin/carddisp.pl?gene=AMY2A
ABCC3	ATP Binding Cassette Subfamily C Member 3	Protein Coding	O15438	49	GC17P050634	1.425421	https://www.genecards.org/cgi-bin/carddisp.pl?gene=ABCC3
FBP1	Fructose-Bisphosphatase 1	Protein Coding	P09467	53	GC09M094603	1.425228	https://www.genecards.org/cgi-bin/carddisp.pl?gene=FBP1
LHX1	LIM Homeobox 1	Protein Coding	P48742	45	GC17P086141	1.422138	https://www.genecards.org/cgi-bin/carddisp.pl?gene=LHX1
MIR133A2	MicroRNA 133a-2	RNA Gene		20	GC20P065681	1.422033	https://www.genecards.org/cgi-bin/carddisp.pl?gene=MIR133A2
SETDB1	SET Domain Bifurcated Histone Lysine Methyltransferase 1	Protein Coding	Q15047	47	GC01P150926	1.420442	https://www.genecards.org/cgi-bin/carddisp.pl?gene=SETDB1

HSP90B1	Heat Shock Protein 90 Beta Family Member 1	Protein Coding	P14625	51	GC12P103930	1.418691	https://www.genecards.org/cgi-bin/carddisp.pl?gene=HSP90B1
SATB2	SATB Homeobox 2	Protein Coding	Q9UPW6	49	GC02M199269	1.417753	https://www.genecards.org/cgi-bin/carddisp.pl?gene=SATB2
SH3BGR	SH3 Domain Binding Glutamate Rich Protein	Protein Coding	P55822	39	GC21P039445	1.416941	https://www.genecards.org/cgi-bin/carddisp.pl?gene=SH3BGR
FZD4	Frizzled Class Receptor 4	Protein Coding	Q9ULV1	53	GC11M086945	1.415873	https://www.genecards.org/cgi-bin/carddisp.pl?gene=FZD4
XCL1	X-C Motif Chemokine Ligand 1	Protein Coding	P47992	42	GC01P168576	1.415271	https://www.genecards.org/cgi-bin/carddisp.pl?gene=XCL1
CASC8	Cancer Susceptibility 8	RNA Gene		17	GC08M134299	1.414961	https://www.genecards.org/cgi-bin/carddisp.pl?gene=CASC8
PDYN	Prodynorphin	Protein Coding	P01213	48	GC20M001978	1.413637	https://www.genecards.org/cgi-bin/carddisp.pl?gene=PDYN
FXYD2	FXYD Domain Containing Ion Transport Regulator 2	Protein Coding	P54710	47	GC11M117800	1.41362	https://www.genecards.org/cgi-bin/carddisp.pl?gene=FXYD2
VAX1	Ventral Anterior Homeobox 1	Protein Coding	Q5SQQ9	41	GC10M117128	1.41362	https://www.genecards.org/cgi-bin/carddisp.pl?gene=VAX1
IGSF10	Immunoglobulin Superfamily Member 10	Protein Coding	Q6WRI0	40	GC03M151425	1.41362	https://www.genecards.org/cgi-bin/carddisp.pl?gene=IGSF10
HS2ST1	Heparan Sulfate 2-O-Sulfotransferase 1	Protein Coding	Q7LGA3	47	GC01P086914	1.413315	https://www.genecards.org/cgi-bin/carddisp.pl?gene=HS2ST1
DUSP4	Dual Specificity Phosphatase 4	Protein Coding	Q13115	45	GC08M029341	1.412188	https://www.genecards.org/cgi-bin/carddisp.pl?gene=DUSP4

KAT2A	Lysine Acetyltransferase 2A	Protein Coding	Q92830	53	GC17M042113	1.410212	https://www.genecards.org/cgi-bin/carddisp.pl?gene=KAT2A
MIR101-1	MicroRNA 101-1	RNA Gene		20	GC01M065058	1.40973	https://www.genecards.org/cgi-bin/carddisp.pl?gene=MIR101-1
CALB1	Calbindin 1	Protein Coding	P05937	46	GC08M090058	1.409681	https://www.genecards.org/cgi-bin/carddisp.pl?gene=CALB1
XIAP	X-Linked Inhibitor Of Apoptosis	Protein Coding	P98170	55	GC0XP123859	1.408109	https://www.genecards.org/cgi-bin/carddisp.pl?gene=XIAP
RARS2	Arginyl-TRNA Synthetase 2, Mitochondrial	Protein Coding	Q5T160	46	GC06M087514	1.407555	https://www.genecards.org/cgi-bin/carddisp.pl?gene=RARS2
ELAVL4	ELAV Like RNA Binding Protein 4	Protein Coding	P26378	44	GC01P050025	1.407555	https://www.genecards.org/cgi-bin/carddisp.pl?gene=ELAVL4
BRAT1	BRCA1 Associated ATM Activator 1	Protein Coding	Q6PJG6	42	GC07M002975	1.406242	https://www.genecards.org/cgi-bin/carddisp.pl?gene=BRAT1
KIF2B	Kinesin Family Member 2B	Protein Coding	Q8N4N8	41	GC17P053822	1.405198	https://www.genecards.org/cgi-bin/carddisp.pl?gene=KIF2B
TUT7	Terminal Uridylyl Transferase 7	Protein Coding	Q5VYS8	41	GC09M104212	1.403755	https://www.genecards.org/cgi-bin/carddisp.pl?gene=TUT7
STPG4	Sperm-Tail PG-Rich Repeat Containing 4	Protein Coding	Q8N801	29	GC02M047045	1.403755	https://www.genecards.org/cgi-bin/carddisp.pl?gene=STPG4
SIGLEC1	Sialic Acid Binding Ig Like Lectin 1	Protein Coding	Q9BZZ2	44	GC20M003686	1.403675	https://www.genecards.org/cgi-bin/carddisp.pl?gene=SIGLEC1
MIR499A	MicroRNA 499a	RNA Gene		22	GC20P034990	1.402693	https://www.genecards.org/cgi-bin/carddisp.pl?gene=MIR499A

SLC16A11	Solute Carrier Family 16 Member 11	Protein Coding	Q8NCK7	36	GC17M007041	1.402236	https://www.genecards.org/cgi-bin/carddisp.pl?gene=SLC16A11
MIR3178	MicroRNA 3178	RNA Gene		14	GC16M002531	1.399225	https://www.genecards.org/cgi-bin/carddisp.pl?gene=MIR3178
ITGB5	Integrin Subunit Beta 5	Protein Coding	P18084	51	GC03M124761	1.398749	https://www.genecards.org/cgi-bin/carddisp.pl?gene=ITGB5
REG3A	Regenerating Family Member 3 Alpha	Protein Coding	Q06141	41	GC02M079157	1.397851	https://www.genecards.org/cgi-bin/carddisp.pl?gene=REG3A
MIR523	MicroRNA 523	RNA Gene		14	GC19P053698	1.396955	https://www.genecards.org/cgi-bin/carddisp.pl?gene=MIR523
DLEU2	Deleted In Lymphocytic Leukemia 2	RNA Gene		25	GC13M049913	1.39644	https://www.genecards.org/cgi-bin/carddisp.pl?gene=DLEU2
ALG6	ALG6 Alpha-1,3-Glucosyltransferase	Protein Coding	Q9Y672	45	GC01P063367	1.394591	https://www.genecards.org/cgi-bin/carddisp.pl?gene=ALG6
TLR7	Toll Like Receptor 7	Protein Coding	Q9NYK1	52	GC0XP012867	1.392907	https://www.genecards.org/cgi-bin/carddisp.pl?gene=TLR7
PEG13	Paternally Expressed 13	RNA Gene		14	GC08M140095	1.392748	https://www.genecards.org/cgi-bin/carddisp.pl?gene=PEG13
E2F1	E2F Transcription Factor 1	Protein Coding	Q01094	48	GC20M033675	1.392464	https://www.genecards.org/cgi-bin/carddisp.pl?gene=E2F1
ALDH1A2	Aldehyde Dehydrogenase 1 Family Member A2	Protein Coding	O94788	53	GC15M136782	1.392296	https://www.genecards.org/cgi-bin/carddisp.pl?gene=ALDH1A2
ELOVL2	ELOVL Fatty Acid Elongase 2	Protein Coding	Q9NXB9	43	GC06M010980	1.390307	https://www.genecards.org/cgi-bin/carddisp.pl?gene=ELOVL2

LINC01191	Long Intergenic Non-Protein Coding RNA 1191	RNA Gene		16	GC02P127100	1.390307	https://www.genecards.org/cgi-bin/carddisp.pl?gene=LINC01191
LPCAT1	Lysophosphatidylcholine Acyltransferase 1	Protein Coding	Q8NF37	40	GC05M001456	1.388831	https://www.genecards.org/cgi-bin/carddisp.pl?gene=LPCAT1
ACO2	Aconitase 2	Protein Coding	Q99798	51	GC22P056803	1.388649	https://www.genecards.org/cgi-bin/carddisp.pl?gene=ACO2
TSN	Translin	Protein Coding	Q15631	44	GC02P121737	1.388316	https://www.genecards.org/cgi-bin/carddisp.pl?gene=TSN
EFTUD2	Elongation Factor Tu GTP Binding Domain Containing 2	Protein Coding	Q15029	47	GC17M064297	1.386767	https://www.genecards.org/cgi-bin/carddisp.pl?gene=EFTUD2
DUSP9	Dual Specificity Phosphatase 9	Protein Coding	Q99956	45	GC0XP153642	1.385864	https://www.genecards.org/cgi-bin/carddisp.pl?gene=DUSP9
NEB	Nebulin	Protein Coding	P20929	45	GC02M151485	1.385735	https://www.genecards.org/cgi-bin/carddisp.pl?gene=NEB
SEMA5A	Semaphorin 5A	Protein Coding	Q13591	46	GC05M009036	1.38483	https://www.genecards.org/cgi-bin/carddisp.pl?gene=SEMA5A
ARSL	Arylsulfatase L	Protein Coding	P51690	46	GC0XM002934	1.384678	https://www.genecards.org/cgi-bin/carddisp.pl?gene=ARSL
PRNP	Prion Protein	Protein Coding	F7VJQ1	52	GC20P004686	1.383975	https://www.genecards.org/cgi-bin/carddisp.pl?gene=PRNP
TREX1	Three Prime Repair Exonuclease 1	Protein Coding	Q9NSU2	46	GC03P053887	1.382923	https://www.genecards.org/cgi-bin/carddisp.pl?gene=TREX1
KHDRBS1	KH RNA Binding Domain Containing, Signal Transduction Associated 1	Protein Coding	Q07666	46	GC01P032013	1.382899	https://www.genecards.org/cgi-bin/carddisp.pl?gene=KHDRBS1

CYP2B6	Cytochrome P450 Family 2 Subfamily B Member 6	Protein Coding	P20813	49	GC19P040991	1.38245	https://www.genecards.org/cgi-bin/carddisp.pl?gene=CYP2B6
ABO	ABO, Alpha 1-3-N-Acetylgalactosaminyltransferase And Alpha 1-3-Galactosyltransferase	Protein Coding	P16442	42	GC09M133250	1.379418	https://www.genecards.org/cgi-bin/carddisp.pl?gene=ABO
SDC1	Syndecan 1	Protein Coding	P18827	47	GC02M020200	1.379307	https://www.genecards.org/cgi-bin/carddisp.pl?gene=SDC1
HSD3BP4	Hydroxy-Delta-5-Steroid Dehydrogenase, 3 Beta, Pseudogene 4	Pseudogene		12	GC01P119563	1.378534	https://www.genecards.org/cgi-bin/carddisp.pl?gene=HSD3BP4
CA10	Carbonic Anhydrase 10	Protein Coding	Q9NS85	44	GC17M051630	1.374963	https://www.genecards.org/cgi-bin/carddisp.pl?gene=CA10
KLF13	KLF Transcription Factor 13	Protein Coding	Q9Y2Y9	43	GC15P031326	1.374722	https://www.genecards.org/cgi-bin/carddisp.pl?gene=KLF13
FOLR3	Folate Receptor Gamma	Protein Coding	P41439	43	GC11P072114	1.374388	https://www.genecards.org/cgi-bin/carddisp.pl?gene=FOLR3
ALDOB	Aldolase, Fructose-Bisphosphate B	Protein Coding	P05062	50	GC09M101420	1.373453	https://www.genecards.org/cgi-bin/carddisp.pl?gene=ALDOB
PYCARD	PYD And CARD Domain Containing	Protein Coding	Q9ULZ3	46	GC16M031201	1.373329	https://www.genecards.org/cgi-bin/carddisp.pl?gene=PYCARD
IL32	Interleukin 32	Protein Coding	P24001	42	GC16P052475	1.373329	https://www.genecards.org/cgi-bin/carddisp.pl?gene=IL32
MIR665	MicroRNA 665	RNA Gene		15	GC14P113621	1.373118	https://www.genecards.org/cgi-bin/carddisp.pl?gene=MIR665
DYSF	Dysferlin	Protein Coding	O75923	47	GC02P071453	1.373089	https://www.genecards.org/cgi-bin/carddisp.pl?gene=DYSF

NOSTRIN	Nitric Oxide Synthase Trafficking	Protein Coding	Q8IVI9	41	GC02P168786	1.372188	https://www.genecards.org/cgi-bin/carddisp.pl?gene=NOSTRIN
RLN2	Relaxin 2	Protein Coding	P04090	38	GC09M005592	1.371661	https://www.genecards.org/cgi-bin/carddisp.pl?gene=RLN2
ANXA6	Annexin A6	Protein Coding	P08133	47	GC05M151100	1.371141	https://www.genecards.org/cgi-bin/carddisp.pl?gene=ANXA6
CADPS2	Calcium Dependent Secretion Activator 2	Protein Coding	Q86UW7	42	GC07M122318	1.369548	https://www.genecards.org/cgi-bin/carddisp.pl?gene=CADPS2
ACVR2B	Activin A Receptor Type 2B	Protein Coding	Q13705	53	GC03P038453	1.368385	https://www.genecards.org/cgi-bin/carddisp.pl?gene=ACVR2B
TAX1BP3	Tax1 Binding Protein 3	Protein Coding	O14907	41	GC17M014194	1.367881	https://www.genecards.org/cgi-bin/carddisp.pl?gene=TAX1BP3
AQP4	Aquaporin 4	Protein Coding	P55087	51	GC18M026852	1.366696	https://www.genecards.org/cgi-bin/carddisp.pl?gene=AQP4
ITGBL1	Integrin Subunit Beta Like 1	Protein Coding	O95965	41	GC13P101454	1.36553	https://www.genecards.org/cgi-bin/carddisp.pl?gene=ITGBL1
ZNHIT3	Zinc Finger HIT-Type Containing 3	Protein Coding	Q15649	40	GC17P036486	1.365134	https://www.genecards.org/cgi-bin/carddisp.pl?gene=ZNHIT3
NLRP1	NLR Family Pyrin Domain Containing 1	Protein Coding	Q9C000	47	GC17M005499	1.364345	https://www.genecards.org/cgi-bin/carddisp.pl?gene=NLRP1
AMPD1	Adenosine Monophosphate Deaminase 1	Protein Coding	P23109	50	GC01M114673	1.363647	https://www.genecards.org/cgi-bin/carddisp.pl?gene=AMPD1
TRA	T Cell Receptor Alpha Locus	Protein Coding	P0DSE1	24	GC14P021621	1.36328	https://www.genecards.org/cgi-bin/carddisp.pl?gene=TRA

PRRX1	Paired Related Homeobox 1	Protein Coding	P54821	46	GC01P170662	1.360224	https://www.genecards.org/cgi-bin/carddisp.pl?gene=PRRX1
MIR497	MicroRNA 497	RNA Gene		20	GC17M014368	1.358808	https://www.genecards.org/cgi-bin/carddisp.pl?gene=MIR497
ALOX12	Arachidonate 12-Lipoxygenase, 12S Type	Protein Coding	P18054	49	GC17P013964	1.358727	https://www.genecards.org/cgi-bin/carddisp.pl?gene=ALOX12
CFLAR	CASP8 And FADD Like Apoptosis Regulator	Protein Coding	O15519	48	GC02P201379	1.357044	https://www.genecards.org/cgi-bin/carddisp.pl?gene=CFLAR
SAMD5	Sterile Alpha Motif Domain Containing 5	Protein Coding	Q5TGI4	33	GC06P147508	1.356601	https://www.genecards.org/cgi-bin/carddisp.pl?gene=SAMD5
RELN	Reelin	Protein Coding	P78509	48	GC07M103471	1.356223	https://www.genecards.org/cgi-bin/carddisp.pl?gene=RELN
RPL27A	Ribosomal Protein L27a	Protein Coding	P46776	44	GC11P008682	1.355628	https://www.genecards.org/cgi-bin/carddisp.pl?gene=RPL27A
GABRA3	Gamma-Aminobutyric Acid Type A Receptor Subunit Alpha3	Protein Coding	P34903	51	GC0XM152166	1.354544	https://www.genecards.org/cgi-bin/carddisp.pl?gene=GABRA3
HOXB1	Homeobox B1	Protein Coding	P14653	47	GC17M048528	1.352942	https://www.genecards.org/cgi-bin/carddisp.pl?gene=HOXB1
PTPN3	Protein Tyrosine Phosphatase Non-Receptor Type 3	Protein Coding	P26045	47	GC09M109375	1.352638	https://www.genecards.org/cgi-bin/carddisp.pl?gene=PTPN3
MIR3648-1	MicroRNA 3648-1	RNA Gene		11	GC21P015092	1.352066	https://www.genecards.org/cgi-bin/carddisp.pl?gene=MIR3648-1
MIR3648-2	MicroRNA 3648-2	RNA Gene		8	GC21P008986	1.352066	https://www.genecards.org/cgi-bin/carddisp.pl?gene=MIR3648-2

LZTR1	Leucine Zipper Like Post Translational Regulator 1	Protein Coding	Q8N653	47	GC22P056081	1.351878	https://www.genecards.org/cgi-bin/carddisp.pl?gene=LZTR1
OPRD1	Opioid Receptor Delta 1	Protein Coding	P41143	47	GC01P028812	1.348387	https://www.genecards.org/cgi-bin/carddisp.pl?gene=OPRD1
GABBR2	Gamma-Aminobutyric Acid Type B Receptor Subunit 2	Protein Coding	O75899	52	GC09M098288	1.348099	https://www.genecards.org/cgi-bin/carddisp.pl?gene=GABBR2
MAT2A	Methionine Adenosyltransferase 2A	Protein Coding	P31153	50	GC02P086113	1.3456	https://www.genecards.org/cgi-bin/carddisp.pl?gene=MAT2A
MAT2B	Methionine Adenosyltransferase 2 Non-Catalytic Beta Subunit	Protein Coding	Q9NZL9	44	GC05P163504	1.3456	https://www.genecards.org/cgi-bin/carddisp.pl?gene=MAT2B
TRIB1	Tribbles Pseudokinase 1	Protein Coding	Q96RU8	42	GC08P125430	1.345503	https://www.genecards.org/cgi-bin/carddisp.pl?gene=TRIB1
ITIH4	Inter-Alpha-Trypsin Inhibitor Heavy Chain 4	Protein Coding	Q14624	45	GC03M052812	1.345374	https://www.genecards.org/cgi-bin/carddisp.pl?gene=ITIH4
NR2F2	Nuclear Receptor Subfamily 2 Group F Member 2	Protein Coding	P24468	53	GC15P096325	1.345181	https://www.genecards.org/cgi-bin/carddisp.pl?gene=NR2F2
FMO3	Flavin Containing Dimethylaniline Monoxygenase 3	Protein Coding	P31513	50	GC01P171090	1.344466	https://www.genecards.org/cgi-bin/carddisp.pl?gene=FMO3
BMPR1B	Bone Morphogenetic Protein Receptor Type 1B	Protein Coding	O00238	53	GC04P094757	1.343836	https://www.genecards.org/cgi-bin/carddisp.pl?gene=BMPR1B
ABL1	ABL Proto-Oncogene 1, Non-Receptor Tyrosine Kinase	Protein Coding	P00519	57	GC09P130713	1.343718	https://www.genecards.org/cgi-bin/carddisp.pl?gene=ABL1
ARSH	Arylsulfatase Family Member H	Protein Coding	Q5FYA8	36	GC0XP003006	1.343208	https://www.genecards.org/cgi-bin/carddisp.pl?gene=ARSH

CTSL	Cathepsin L	Protein Coding	P07711	51	GC09P087725	1.342541	https://www.genecards.org/cgi-bin/carddisp.pl?gene=CTSL
S100A4	S100 Calcium Binding Protein A4	Protein Coding	P26447	49	GC01M153543	1.342481	https://www.genecards.org/cgi-bin/carddisp.pl?gene=S100A4
CCL22	C-C Motif Chemokine Ligand 22	Protein Coding	O00626	41	GC16P059624	1.341805	https://www.genecards.org/cgi-bin/carddisp.pl?gene=CCL22
CCNE1	Cyclin E1	Protein Coding	P24864	52	GC19P029811	1.341338	https://www.genecards.org/cgi-bin/carddisp.pl?gene=CCNE1
WNT7B	Wnt Family Member 7B	Protein Coding	P56706	48	GC22M045920	1.341338	https://www.genecards.org/cgi-bin/carddisp.pl?gene=WNT7B
ALX3	ALX Homeobox 3	Protein Coding	O95076	38	GC01M110059	1.341338	https://www.genecards.org/cgi-bin/carddisp.pl?gene=ALX3
DENND1B	DENN Domain Containing 1B	Protein Coding	Q6P3S1	39	GC01M197473	1.341013	https://www.genecards.org/cgi-bin/carddisp.pl?gene=DENND1B
MIR576	MicroRNA 576	RNA Gene		16	GC04P109488	1.341013	https://www.genecards.org/cgi-bin/carddisp.pl?gene=MIR576
PROKR1	Prokineticin Receptor 1	Protein Coding	Q8TCW9	41	GC02P068643	1.340636	https://www.genecards.org/cgi-bin/carddisp.pl?gene=PROKR1
RGS2	Regulator Of G Protein Signaling 2	Protein Coding	P41220	46	GC01P192809	1.340215	https://www.genecards.org/cgi-bin/carddisp.pl?gene=RGS2
MIR505	MicroRNA 505	RNA Gene		19	GC0XM139924	1.339444	https://www.genecards.org/cgi-bin/carddisp.pl?gene=MIR505
IFI16	Interferon Gamma Inducible Protein 16	Protein Coding	Q16666	46	GC01P158969	1.33924	https://www.genecards.org/cgi-bin/carddisp.pl?gene=IFI16

MIR519C	MicroRNA 519c	RNA Gene		17	GC19P053686	1.336197	https://www.genecards.org/cgi-bin/carddisp.pl?gene=MIR519C
GART	Phosphoribosylglycinamide Formyltransferase, Phosphoribosylglycinamide Synthetase, Phosphoribosylaminoimidazole Synthetase	Protein Coding	P22102	48	GC21M033503	1.335257	https://www.genecards.org/cgi-bin/carddisp.pl?gene=GART
MIR432	MicroRNA 432	RNA Gene		19	GC14P112883	1.334651	https://www.genecards.org/cgi-bin/carddisp.pl?gene=MIR432
GSTA1	Glutathione S-Transferase Alpha 1	Protein Coding	P08263	46	GC06M052791	1.334632	https://www.genecards.org/cgi-bin/carddisp.pl?gene=GSTA1
GFM1	G Elongation Factor Mitochondrial 1	Protein Coding	Q96RP9	48	GC03P158644	1.334154	https://www.genecards.org/cgi-bin/carddisp.pl?gene=GFM1
CXADR	CXADR Ig-Like Cell Adhesion Molecule	Protein Coding	P78310	47	GC21P017513	1.334029	https://www.genecards.org/cgi-bin/carddisp.pl?gene=CXADR
SLC2A12	Solute Carrier Family 2 Member 12	Protein Coding	Q8TD20	42	GC06M133987	1.332001	https://www.genecards.org/cgi-bin/carddisp.pl?gene=SLC2A12
ERBB3	Erb-B2 Receptor Tyrosine Kinase 3	Protein Coding	P21860	58	GC12P058609	1.331707	https://www.genecards.org/cgi-bin/carddisp.pl?gene=ERBB3
COPG2IT1	COPG2 Imprinted Transcript 1	RNA Gene		14	GC07P133943	1.330749	https://www.genecards.org/cgi-bin/carddisp.pl?gene=COPG2IT1
HJV	Hemojuvelin BMP Co-Receptor	Protein Coding	Q6ZVN8	47	GC01M157196	1.330111	https://www.genecards.org/cgi-bin/carddisp.pl?gene=HJV
CHRM3	Cholinergic Receptor Muscarinic 3	Protein Coding	P20309	52	GC01P239386	1.327445	https://www.genecards.org/cgi-bin/carddisp.pl?gene=CHRM3

PPP1R13L	Protein Phosphatase 1 Regulatory Subunit 13 Like	Protein Coding	Q8WUF5	45	GC19M045379	1.325907	https://www.genecards.org/cgi-bin/carddisp.pl?gene=PPP1R13L
DDAH2	Dimethylarginine Dimethylaminohydrolase 2	Protein Coding	O95865	46	GC06M031727	1.324973	https://www.genecards.org/cgi-bin/carddisp.pl?gene=DDAH2
ABCA3	ATP Binding Cassette Subfamily A Member 3	Protein Coding	Q99758	52	GC16M002275	1.323987	https://www.genecards.org/cgi-bin/carddisp.pl?gene=ABCA3
VTN	Vitronectin	Protein Coding	P04004	48	GC17M063257	1.323751	https://www.genecards.org/cgi-bin/carddisp.pl?gene=VTN
NT5E	5'-Nucleotidase Ecto	Protein Coding	P21589	56	GC06P085449	1.322568	https://www.genecards.org/cgi-bin/carddisp.pl?gene=NT5E
HR	HR Lysine Demethylase And Nuclear Receptor Corepressor	Protein Coding	O43593	46	GC08M022114	1.322568	https://www.genecards.org/cgi-bin/carddisp.pl?gene=HR
RP2	RP2 Activator Of ARL3 GTPase	Protein Coding	O75695	46	GC0XP046837	1.321578	https://www.genecards.org/cgi-bin/carddisp.pl?gene=RP2
TNFSF4	TNF Superfamily Member 4	Protein Coding	P23510	46	GC01M173183	1.321578	https://www.genecards.org/cgi-bin/carddisp.pl?gene=TNFSF4
TRAF7	TNF Receptor Associated Factor 7	Protein Coding	Q6Q0C0	45	GC16P052441	1.321578	https://www.genecards.org/cgi-bin/carddisp.pl?gene=TRAF7
EBI3	Epstein-Barr Virus Induced 3	Protein Coding	Q14213	42	GC19P008710	1.319869	https://www.genecards.org/cgi-bin/carddisp.pl?gene=EBI3
CD28	CD28 Molecule	Protein Coding	P10747	52	GC02P203706	1.319202	https://www.genecards.org/cgi-bin/carddisp.pl?gene=CD28
CERS3	Ceramide Synthase 3	Protein Coding	Q8IU89	45	GC15M137675	1.319185	https://www.genecards.org/cgi-bin/carddisp.pl?gene=CERS3

PTCH2	Patched 2	Protein Coding	Q9Y6C5	50	GC01M044819	1.314901	https://www.genecards.org/cgi-bin/carddisp.pl?gene=PTCH2
HOXA7	Homeobox A7	Protein Coding	P31268	43	GC07M027153	1.314901	https://www.genecards.org/cgi-bin/carddisp.pl?gene=HOXA7
FOXF2	Forkhead Box F2	Protein Coding	Q12947	40	GC06P001389	1.314901	https://www.genecards.org/cgi-bin/carddisp.pl?gene=FOXF2
FIRRE	Firre Intergenic Repeating RNA Element	RNA Gene		17	GC0XM131691	1.314755	https://www.genecards.org/cgi-bin/carddisp.pl?gene=FIRRE
AMMECR1	AMMECR Nuclear Protein 1	Protein Coding	Q9Y4X0	43	GC0XM110194	1.311754	https://www.genecards.org/cgi-bin/carddisp.pl?gene=AMMECR1
CD38	CD38 Molecule	Protein Coding	P28907	51	GC04P022198	1.311113	https://www.genecards.org/cgi-bin/carddisp.pl?gene=CD38
MIR1275	MicroRNA 1275	RNA Gene		19	GC06M084073	1.308766	https://www.genecards.org/cgi-bin/carddisp.pl?gene=MIR1275
AMACR	Alpha-Methylacyl-CoA Racemase	Protein Coding	Q9UHK6	49	GC05M033986	1.308554	https://www.genecards.org/cgi-bin/carddisp.pl?gene=AMACR
HUWE1	HECT, UBA And WWE Domain Containing E3 Ubiquitin Protein Ligase 1	Protein Coding	Q7Z6Z7	50	GC0XM053532	1.308511	https://www.genecards.org/cgi-bin/carddisp.pl?gene=HUWE1
FZD6	Frizzled Class Receptor 6	Protein Coding	O60353	50	GC08P103298	1.308436	https://www.genecards.org/cgi-bin/carddisp.pl?gene=FZD6
HES7	Hes Family BHLH Transcription Factor 7	Protein Coding	Q9BYE0	40	GC17M008120	1.308436	https://www.genecards.org/cgi-bin/carddisp.pl?gene=HES7
MESP2	Mesoderm Posterior BHLH Transcription Factor 2	Protein Coding	Q0VG99	39	GC15P137738	1.308436	https://www.genecards.org/cgi-bin/carddisp.pl?gene=MESP2

MIR4516	MicroRNA 4516	RNA Gene		16	GC16P052438	1.308109	https://www.genecards.org/cgi-bin/carddisp.pl?gene=MIR4516
LGALS3BP	Galectin 3 Binding Protein	Protein Coding	Q08380	46	GC17M078971	1.307871	https://www.genecards.org/cgi-bin/carddisp.pl?gene=LGALS3BP
HOXB6	Homeobox B6	Protein Coding	P17509	40	GC17M064635	1.305702	https://www.genecards.org/cgi-bin/carddisp.pl?gene=HOXB6
LEAP2	Liver Enriched Antimicrobial Peptide 2	Protein Coding	Q969E1	36	GC05P132872	1.303965	https://www.genecards.org/cgi-bin/carddisp.pl?gene=LEAP2
CELF2	CUGBP Elav-Like Family Member 2	Protein Coding	O95319	49	GC10P010462	1.303835	https://www.genecards.org/cgi-bin/carddisp.pl?gene=CELF2
PFDN4	Prefoldin Subunit 4	Protein Coding	Q9NQP4	42	GC20P054207	1.303835	https://www.genecards.org/cgi-bin/carddisp.pl?gene=PFDN4
MBNL2	Muscleblind Like Splicing Regulator 2	Protein Coding	Q5VZF2	41	GC13P097141	1.303835	https://www.genecards.org/cgi-bin/carddisp.pl?gene=MBNL2
MIR671	MicroRNA 671	RNA Gene		18	GC07P151238	1.301686	https://www.genecards.org/cgi-bin/carddisp.pl?gene=MIR671
HPD	4-Hydroxyphenylpyruvate Dioxygenase	Protein Coding	P32754	50	GC12M121839	1.301351	https://www.genecards.org/cgi-bin/carddisp.pl?gene=HPD
FOXN1	Forkhead Box N1	Protein Coding	O15353	46	GC17P028506	1.301076	https://www.genecards.org/cgi-bin/carddisp.pl?gene=FOXN1
MSC	Musculin	Protein Coding	O60682	43	GC08M071836	1.301076	https://www.genecards.org/cgi-bin/carddisp.pl?gene=MSC
ERBB4	Erb-B2 Receptor Tyrosine Kinase 4	Protein Coding	Q15303	58	GC02M211375	1.299864	https://www.genecards.org/cgi-bin/carddisp.pl?gene=ERBB4

EDN3	Endothelin 3	Protein Coding	P14138	50	GC20P059300	1.299775	https://www.genecards.org/cgi-bin/carddisp.pl?gene=EDN3
BYSL	Bystin Like	Protein Coding	Q13895	42	GC06P112205	1.298555	https://www.genecards.org/cgi-bin/carddisp.pl?gene=BYSL
PSMD3	Proteasome 26S Subunit, Non-ATPase 3	Protein Coding	O43242	46	GC17P039980	1.29774	https://www.genecards.org/cgi-bin/carddisp.pl?gene=PSMD3
CHRD	Chordin	Protein Coding	Q9H2X0	45	GC03P184380	1.297464	https://www.genecards.org/cgi-bin/carddisp.pl?gene=CHRD
ESRRG	Estrogen Related Receptor Gamma	Protein Coding	P62508	47	GC01M216503	1.29719	https://www.genecards.org/cgi-bin/carddisp.pl?gene=ESRRG
SMAD6	SMAD Family Member 6	Protein Coding	O43541	51	GC15P066702	1.293097	https://www.genecards.org/cgi-bin/carddisp.pl?gene=SMAD6
TSC1	TSC Complex Subunit 1	Protein Coding	Q92574	52	GC09M132891	1.292965	https://www.genecards.org/cgi-bin/carddisp.pl?gene=TSC1
TSPY1	Testis Specific Protein Y-Linked 1	Protein Coding	Q01534	35	GC0YP009469	1.292765	https://www.genecards.org/cgi-bin/carddisp.pl?gene=TSPY1
THY1	Thy-1 Cell Surface Antigen	Protein Coding	P04216	47	GC11M119465	1.292379	https://www.genecards.org/cgi-bin/carddisp.pl?gene=THY1
IL36A	Interleukin 36 Alpha	Protein Coding	Q9UHA7	41	GC02P113005	1.291289	https://www.genecards.org/cgi-bin/carddisp.pl?gene=IL36A
SERPINH1	Serpin Family H Member 1	Protein Coding	P50454	51	GC11P075562	1.291	https://www.genecards.org/cgi-bin/carddisp.pl?gene=SERPINH1
EFHC1	EF-Hand Domain Containing 1	Protein Coding	Q5JVL4	45	GC06P052362	1.290304	https://www.genecards.org/cgi-bin/carddisp.pl?gene=EFHC1

CEP104	Centrosomal Protein 104	Protein Coding	O60308	42	GC01M003812	1.290304	https://www.genecards.org/cgi-bin/carddisp.pl?gene=CEP104
NOTCH4	Notch Receptor 4	Protein Coding	Q99466	50	GC06M083984	1.288745	https://www.genecards.org/cgi-bin/carddisp.pl?gene=NOTCH4
TNC	Tenascin C	Protein Coding	P24821	53	GC09M115019	1.288426	https://www.genecards.org/cgi-bin/carddisp.pl?gene=TNC
NAGLU	N-Acetyl-Alpha-Glucosaminidase	Protein Coding	P54802	47	GC17P086412	1.287804	https://www.genecards.org/cgi-bin/carddisp.pl?gene=NAGLU
ZBTB16	Zinc Finger And BTB Domain Containing 16	Protein Coding	Q05516	47	GC11P114059	1.287472	https://www.genecards.org/cgi-bin/carddisp.pl?gene=ZBTB16
RLN3	Relaxin 3	Protein Coding	Q8WXF3	39	GC19P088987	1.287029	https://www.genecards.org/cgi-bin/carddisp.pl?gene=RLN3
MIR518D	MicroRNA 518d	RNA Gene		14	GC19P053734	1.285423	https://www.genecards.org/cgi-bin/carddisp.pl?gene=MIR518D
FLVCR1	FLVCR Choline And Heme Transporter 1	Protein Coding	Q9Y5Y0	44	GC01P212858	1.284325	https://www.genecards.org/cgi-bin/carddisp.pl?gene=FLVCR1
ENSG00000276609		RNA Gene		8	GC07P027200	1.284265	https://www.genecards.org/cgi-bin/carddisp.pl?gene=ENSG00000276609
ENSG00000277469		RNA Gene		6	GC07P027202	1.284265	https://www.genecards.org/cgi-bin/carddisp.pl?gene=ENSG00000277469
ENSG00000277553		RNA Gene		6	GC07P027206	1.284265	https://www.genecards.org/cgi-bin/carddisp.pl?gene=ENSG00000277553

ENSG00000278708		RNA Gene		6	GC07P027201	1.284265	https://www.genecards.org/cgi-bin/carddisp.pl?gene=ENSG00000278708
SPAST	Spastin	Protein Coding	Q9UBP0	45	GC02P032063	1.284197	https://www.genecards.org/cgi-bin/carddisp.pl?gene=SPAST
CD9	CD9 Molecule	Protein Coding	P21926	48	GC12P030429	1.283952	https://www.genecards.org/cgi-bin/carddisp.pl?gene=CD9
HSF1	Heat Shock Transcription Factor 1	Protein Coding	Q00613	51	GC08P144291	1.283425	https://www.genecards.org/cgi-bin/carddisp.pl?gene=HSF1
AKAP1	A-Kinase Anchoring Protein 1	Protein Coding	Q92667	45	GC17P057085	1.281874	https://www.genecards.org/cgi-bin/carddisp.pl?gene=AKAP1
ID1	Inhibitor Of DNA Binding 1	Protein Coding	P41134	45	GC20P031605	1.280815	https://www.genecards.org/cgi-bin/carddisp.pl?gene=ID1
KDM1A	Lysine Demethylase 1A	Protein Coding	O60341	53	GC01P023019	1.280296	https://www.genecards.org/cgi-bin/carddisp.pl?gene=KDM1A
SRSF2	Serine And Arginine Rich Splicing Factor 2	Protein Coding	Q01130	46	GC17M076734	1.280296	https://www.genecards.org/cgi-bin/carddisp.pl?gene=SRSF2
CLDN19	Claudin 19	Protein Coding	Q8N6F1	46	GC01M042733	1.279957	https://www.genecards.org/cgi-bin/carddisp.pl?gene=CLDN19
CHDH	Choline Dehydrogenase	Protein Coding	Q8NE62	44	GC03M053812	1.279669	https://www.genecards.org/cgi-bin/carddisp.pl?gene=CHDH
PKD2	Polycystin 2, Transient Receptor Potential Cation Channel	Protein Coding	Q13563	52	GC04P088007	1.279599	https://www.genecards.org/cgi-bin/carddisp.pl?gene=PKD2
IDH3A	Isocitrate Dehydrogenase (NAD(+)) 3 Catalytic Subunit Alpha	Protein Coding	P50213	50	GC15P078131	1.278616	https://www.genecards.org/cgi-bin/carddisp.pl?gene=IDH3A

KCNAB2	Potassium Voltage-Gated Channel Subfamily A Regulatory Beta Subunit 2	Protein Coding	Q13303	47	GC01P006248	1.278616	https://www.genecards.org/cgi-bin/carddisp.pl?gene=KCNAB2
PRPF6	Pre-MRNA Processing Factor 6	Protein Coding	O94906	46	GC20P063981	1.278616	https://www.genecards.org/cgi-bin/carddisp.pl?gene=PRPF6
GREM1	Gremlin 1, DAN Family BMP Antagonist	Protein Coding	O60565	47	GC15P057003	1.27594	https://www.genecards.org/cgi-bin/carddisp.pl?gene=GREM1
ANXA2	Annexin A2	Protein Coding	P07355	51	GC15M060347	1.275696	https://www.genecards.org/cgi-bin/carddisp.pl?gene=ANXA2
PTGIR	Prostaglandin I2 Receptor	Protein Coding	P43119	50	GC19M082757	1.275656	https://www.genecards.org/cgi-bin/carddisp.pl?gene=PTGIR
SLC27A2	Solute Carrier Family 27 Member 2	Protein Coding	O14975	49	GC15P050182	1.275054	https://www.genecards.org/cgi-bin/carddisp.pl?gene=SLC27A2
MIR382	MicroRNA 382	RNA Gene		17	GC14P113602	1.275006	https://www.genecards.org/cgi-bin/carddisp.pl?gene=MIR382
TAP2	Transporter 2, ATP Binding Cassette Subfamily B Member	Protein Coding	Q03519	48	GC06M032821	1.272774	https://www.genecards.org/cgi-bin/carddisp.pl?gene=TAP2
MGAT3	Beta-1,4-Mannosyl-Glycoprotein 4-Beta-N-Acetylglucosaminyltransferase	Protein Coding	Q09327	44	GC22P056711	1.267656	https://www.genecards.org/cgi-bin/carddisp.pl?gene=MGAT3
CTBP2	C-Terminal Binding Protein 2	Protein Coding	P56545	47	GC10M124984	1.266471	https://www.genecards.org/cgi-bin/carddisp.pl?gene=CTBP2
AURKB	Aurora Kinase B	Protein Coding	Q96GD4	55	GC17M014442	1.266101	https://www.genecards.org/cgi-bin/carddisp.pl?gene=AURKB
STC2	Stanniocalcin 2	Protein Coding	O76061	46	GC05M173314	1.264909	https://www.genecards.org/cgi-bin/carddisp.pl?gene=STC2

GLRX3	Glutaredoxin 3	Protein Coding	O76003	44	GC10P130136	1.264318	https://www.genecards.org/cgi-bin/carddisp.pl?gene=GLRX3
ATOH7	Atonal BHLH Transcription Factor 7	Protein Coding	Q8N100	42	GC10M068230	1.264161	https://www.genecards.org/cgi-bin/carddisp.pl?gene=ATOH7
EXOSC4	Exosome Component 4	Protein Coding	Q9NPD3	41	GC08P145894	1.264161	https://www.genecards.org/cgi-bin/carddisp.pl?gene=EXOSC4
VAX2	Ventral Anterior Homeobox 2	Protein Coding	Q9UIW0	41	GC02P070900	1.264161	https://www.genecards.org/cgi-bin/carddisp.pl?gene=VAX2
FOXB1	Forkhead Box B1	Protein Coding	Q99853	40	GC15P060004	1.264161	https://www.genecards.org/cgi-bin/carddisp.pl?gene=FOXB1
HSP90AB1	Heat Shock Protein 90 Alpha Family Class B Member 1	Protein Coding	P08238	53	GC06P044246	1.263776	https://www.genecards.org/cgi-bin/carddisp.pl?gene=HSP90AB1
SUCLA2	Succinate-CoA Ligase ADP-Forming Subunit Beta	Protein Coding	Q9P2R7	50	GC13M047745	1.263644	https://www.genecards.org/cgi-bin/carddisp.pl?gene=SUCLA2
PIM1	Pim-1 Proto-Oncogene, Serine/Threonine Kinase	Protein Coding	P11309	53	GC06P112170	1.2619	https://www.genecards.org/cgi-bin/carddisp.pl?gene=PIM1
KLHDC2	Kelch Domain Containing 2	Protein Coding	Q9Y2U9	41	GC14P049889	1.261796	https://www.genecards.org/cgi-bin/carddisp.pl?gene=KLHDC2
TNFRSF8	TNF Receptor Superfamily Member 8	Protein Coding	P28908	47	GC01P012063	1.260722	https://www.genecards.org/cgi-bin/carddisp.pl?gene=TNFRSF8
EPCAM	Epithelial Cell Adhesion Molecule	Protein Coding	P16422	52	GC02P047345	1.260233	https://www.genecards.org/cgi-bin/carddisp.pl?gene=EPCAM
CUL1	Cullin 1	Protein Coding	Q13616	48	GC07P148697	1.2597	https://www.genecards.org/cgi-bin/carddisp.pl?gene=CUL1

MED12	Mediator Complex Subunit 12	Protein Coding	Q93074	48	GC0XP071118	1.256518	https://www.genecards.org/cgi-bin/carddisp.pl?gene=MED12
MIR135A1	MicroRNA 135a-1	RNA Gene		21	GC03M053016	1.256383	https://www.genecards.org/cgi-bin/carddisp.pl?gene=MIR135A1
LYST	Lysosomal Trafficking Regulator	Protein Coding	Q99698	42	GC01M235661	1.254196	https://www.genecards.org/cgi-bin/carddisp.pl?gene=LYST
CYP3A5	Cytochrome P450 Family 3 Subfamily A Member 5	Protein Coding	P20815	48	GC07M099648	1.25309	https://www.genecards.org/cgi-bin/carddisp.pl?gene=CYP3A5
LPIN2	Lipin 2	Protein Coding	Q92539	46	GC18M003513	1.25057	https://www.genecards.org/cgi-bin/carddisp.pl?gene=LPIN2
ENSG00000228741	Spermatogenesis Associated 13	Uncategorized		9	GC13P024065	1.250286	https://www.genecards.org/cgi-bin/carddisp.pl?gene=ENSG00000228741
ORM1	Orosomucoid 1	Protein Coding	P02763	45	GC09P114323	1.24942	https://www.genecards.org/cgi-bin/carddisp.pl?gene=ORM1
SLC16A2	Solute Carrier Family 16 Member 2	Protein Coding	P36021	47	GC0XP074676	1.247555	https://www.genecards.org/cgi-bin/carddisp.pl?gene=SLC16A2
RAB32	RAB32, Member RAS Oncogene Family	Protein Coding	Q13637	40	GC06P146543	1.246905	https://www.genecards.org/cgi-bin/carddisp.pl?gene=RAB32
TNXA	Tenascin XA (Pseudogene)	Pseudogene	Q16473	25	GC06M083973	1.245464	https://www.genecards.org/cgi-bin/carddisp.pl?gene=TNXA
BMP10	Bone Morphogenetic Protein 10	Protein Coding	O95393	42	GC02M068865	1.242864	https://www.genecards.org/cgi-bin/carddisp.pl?gene=BMP10
BCR	BCR Activator Of RhoGEF And GTPase	Protein Coding	P11274	55	GC22P023179	1.24106	https://www.genecards.org/cgi-bin/carddisp.pl?gene=BCR

ALDH1A1	Aldehyde Dehydrogenase 1 Family Member A1	Protein Coding	P00352	51	GC09M072900	1.240708	https://www.genecards.org/cgi-bin/carddisp.pl?gene=ALDH1A1
FZD7	Frizzled Class Receptor 7	Protein Coding	O75084	48	GC02P202228	1.240366	https://www.genecards.org/cgi-bin/carddisp.pl?gene=FZD7
JAG2	Jagged Canonical Notch Ligand 2	Protein Coding	Q9Y219	48	GC14M105140	1.240366	https://www.genecards.org/cgi-bin/carddisp.pl?gene=JAG2
AGRN	Agrin	Protein Coding	O00468	49	GC01P001020	1.239864	https://www.genecards.org/cgi-bin/carddisp.pl?gene=AGRN
APOBR	Apolipoprotein B Receptor	Protein Coding	Q0VD83	38	GC16P028494	1.239579	https://www.genecards.org/cgi-bin/carddisp.pl?gene=APOBR
SLC7A8	Solute Carrier Family 7 Member 8	Protein Coding	Q9UHI5	44	GC14M023125	1.238177	https://www.genecards.org/cgi-bin/carddisp.pl?gene=SLC7A8
RNA45SN1	RNA, 45S Pre-Ribosomal N1	RNA Gene		8	GC21P015828	1.236954	https://www.genecards.org/cgi-bin/carddisp.pl?gene=RNA45SN1
RNA45SN2	RNA, 45S Pre-Ribosomal N2	RNA Gene		8	GC21P015829	1.236954	https://www.genecards.org/cgi-bin/carddisp.pl?gene=RNA45SN2
RNA45SN3	RNA, 45S Pre-Ribosomal N3	RNA Gene		8	GC21P015830	1.236954	https://www.genecards.org/cgi-bin/carddisp.pl?gene=RNA45SN3
NR5A2	Nuclear Receptor Subfamily 5 Group A Member 2	Protein Coding	O00482	48	GC01P199996	1.232913	https://www.genecards.org/cgi-bin/carddisp.pl?gene=NR5A2
KIF6	Kinesin Family Member 6	Protein Coding	Q6ZMV9	41	GC06M084113	1.232124	https://www.genecards.org/cgi-bin/carddisp.pl?gene=KIF6
ETS2	ETS Proto-Oncogene 2, Transcription Factor	Protein Coding	P15036	45	GC21P038805	1.23104	https://www.genecards.org/cgi-bin/carddisp.pl?gene=ETS2

PON3	Paraoxonase 3	Protein Coding	Q15166	48	GC07M095359	1.230309	https://www.genecards.org/cgi-bin/carddisp.pl?gene=PON3
KCNE2	Potassium Voltage-Gated Channel Subfamily E Regulatory Subunit 2	Protein Coding	Q9Y6J6	46	GC21P034364	1.230309	https://www.genecards.org/cgi-bin/carddisp.pl?gene=KCNE2
PIWIL4	Piwi Like RNA-Mediated Gene Silencing 4	Protein Coding	Q7Z3Z4	42	GC11P094543	1.230171	https://www.genecards.org/cgi-bin/carddisp.pl?gene=PIWIL4
AHCY	Adenosylhomocysteinase	Protein Coding	P23526	53	GC20M035459	1.228633	https://www.genecards.org/cgi-bin/carddisp.pl?gene=AHCY
LYN	LYN Proto-Oncogene, Src Family Tyrosine Kinase	Protein Coding	P07948	53	GC08P055879	1.227834	https://www.genecards.org/cgi-bin/carddisp.pl?gene=LYN
DNAH5	Dynein Axonemal Heavy Chain 5	Protein Coding	Q8TE73	43	GC05M013693	1.217521	https://www.genecards.org/cgi-bin/carddisp.pl?gene=DNAH5
SELENOH	Selenoprotein H	Protein Coding	Q8IZQ5	33	GC11P058380	1.216859	https://www.genecards.org/cgi-bin/carddisp.pl?gene=SELENOH
GUCY2C	Guanylate Cyclase 2C	Protein Coding	P25092	50	GC12M014612	1.216801	https://www.genecards.org/cgi-bin/carddisp.pl?gene=GUCY2C
S100A6	S100 Calcium Binding Protein A6	Protein Coding	P06703	46	GC01M157655	1.216694	https://www.genecards.org/cgi-bin/carddisp.pl?gene=S100A6
EIF3A	Eukaryotic Translation Initiation Factor 3 Subunit A	Protein Coding	Q14152	48	GC10M119034	1.214026	https://www.genecards.org/cgi-bin/carddisp.pl?gene=EIF3A
AKR1A1	Aldo-Keto Reductase Family 1 Member A1	Protein Coding	P14550	48	GC01P045550	1.212597	https://www.genecards.org/cgi-bin/carddisp.pl?gene=AKR1A1
ABCC4	ATP Binding Cassette Subfamily C Member 4	Protein Coding	O15439	49	GC13M095019	1.212307	https://www.genecards.org/cgi-bin/carddisp.pl?gene=ABCC4

MESTIT1	MEST Intronic Transcript 1, Antisense RNA	RNA Gene		16	GC07M130471	1.210608	https://www.genecards.org/cgi-bin/carddisp.pl?gene=MESTIT1
FLAD1	Flavin Adenine Dinucleotide Synthetase 1	Protein Coding	Q8NFF5	45	GC01P154983	1.210156	https://www.genecards.org/cgi-bin/carddisp.pl?gene=FLAD1
SLC4A1	Solute Carrier Family 4 Member 1 (Diego Blood Group)	Protein Coding	P02730	52	GC17M064272	1.209893	https://www.genecards.org/cgi-bin/carddisp.pl?gene=SLC4A1
SP110	SP110 Nuclear Body Protein	Protein Coding	Q9HB58	44	GC02M230167	1.209893	https://www.genecards.org/cgi-bin/carddisp.pl?gene=SP110
HEPHL1	Hephaestin Like 1	Protein Coding	Q6MZM0	42	GC11P094021	1.209893	https://www.genecards.org/cgi-bin/carddisp.pl?gene=HEPHL1
BPGM	Bisphosphoglycerate Mutase	Protein Coding	P07738	49	GC07P134646	1.207924	https://www.genecards.org/cgi-bin/carddisp.pl?gene=BPGM
PEX10	Peroxisomal Biogenesis Factor 10	Protein Coding	O60683	46	GC01M002403	1.207523	https://www.genecards.org/cgi-bin/carddisp.pl?gene=PEX10
ADARB1	Adenosine Deaminase RNA Specific B1	Protein Coding	P78563	48	GC21P045073	1.207498	https://www.genecards.org/cgi-bin/carddisp.pl?gene=ADARB1
CARD9	Caspase Recruitment Domain Family Member 9	Protein Coding	Q9H257	49	GC09M136364	1.207137	https://www.genecards.org/cgi-bin/carddisp.pl?gene=CARD9
PPP3CA	Protein Phosphatase 3 Catalytic Subunit Alpha	Protein Coding	Q08209	55	GC04M101024	1.20576	https://www.genecards.org/cgi-bin/carddisp.pl?gene=PPP3CA
NBR2	Neighbor Of BRCA1 LncRNA 2	RNA Gene		25	GC17P043125	1.196617	https://www.genecards.org/cgi-bin/carddisp.pl?gene=NBR2
SLC26A4	Solute Carrier Family 26 Member 4	Protein Coding	O43511	47	GC07P107660	1.194114	https://www.genecards.org/cgi-bin/carddisp.pl?gene=SLC26A4

FLNA	Filamin A	Protein Coding	P21333	52	GC0XM154348	1.192679	https://www.genecards.org/cgi-bin/carddisp.pl?gene=FLNA
CTTN	Cortactin	Protein Coding	Q14247	48	GC11P070398	1.190173	https://www.genecards.org/cgi-bin/carddisp.pl?gene=CTTN
EIF4G1	Eukaryotic Translation Initiation Factor 4 Gamma 1	Protein Coding	Q04637	51	GC03P184314	1.188245	https://www.genecards.org/cgi-bin/carddisp.pl?gene=EIF4G1
MIR708	MicroRNA 708	RNA Gene		19	GC11M079402	1.187488	https://www.genecards.org/cgi-bin/carddisp.pl?gene=MIR708
DYNC2LI1	Dynein Cytoplasmic 2 Light Intermediate Chain 1	Protein Coding	Q8TCX1	44	GC02P043788	1.186585	https://www.genecards.org/cgi-bin/carddisp.pl?gene=DYNC2LI1
APCDD1	APC Down-Regulated 1	Protein Coding	Q8J025	45	GC18P010454	1.186056	https://www.genecards.org/cgi-bin/carddisp.pl?gene=APCDD1
PCDH19	Protocadherin 19	Protein Coding	Q8TAB3	47	GC0XM100291	1.185674	https://www.genecards.org/cgi-bin/carddisp.pl?gene=PCDH19
PDGFRB	Platelet Derived Growth Factor Receptor Beta	Protein Coding	P09619	57	GC05M150113	1.185443	https://www.genecards.org/cgi-bin/carddisp.pl?gene=PDGFRB
ILF3	Interleukin Enhancer Binding Factor 3	Protein Coding	Q12906	43	GC19P088783	1.184516	https://www.genecards.org/cgi-bin/carddisp.pl?gene=ILF3
PRKCI	Protein Kinase C Iota	Protein Coding	P41743	52	GC03P170222	1.183893	https://www.genecards.org/cgi-bin/carddisp.pl?gene=PRKCI
ACAT1	Acetyl-CoA Acetyltransferase 1	Protein Coding	P24752	53	GC11P108121	1.183309	https://www.genecards.org/cgi-bin/carddisp.pl?gene=ACAT1
RRM2B	Ribonucleotide Reductase Regulatory TP53 Inducible Subunit M2B	Protein Coding	Q7LG56	53	GC08M102204	1.182583	https://www.genecards.org/cgi-bin/carddisp.pl?gene=RRM2B

SLC52A2	Solute Carrier Family 52 Member 2	Protein Coding	Q9HAB3	42	GC08P144333	1.182226	https://www.genecards.org/cgi-bin/carddisp.pl?gene=SLC52A2
ASXL1	ASXL Transcriptional Regulator 1	Protein Coding	Q8IXJ9	47	GC20P035983	1.180665	https://www.genecards.org/cgi-bin/carddisp.pl?gene=ASXL1
CCNA2	Cyclin A2	Protein Coding	P20248	50	GC04M121816	1.17996	https://www.genecards.org/cgi-bin/carddisp.pl?gene=CCNA2
GDNF	Glial Cell Derived Neurotrophic Factor	Protein Coding	P39905	54	GC05M037812	1.176727	https://www.genecards.org/cgi-bin/carddisp.pl?gene=GDNF
MFAP5	Microfibril Associated Protein 5	Protein Coding	Q13361	48	GC12M008637	1.174635	https://www.genecards.org/cgi-bin/carddisp.pl?gene=MFAP5
SLC25A10	Solute Carrier Family 25 Member 10	Protein Coding	Q9UBX3	47	GC17P081712	1.173492	https://www.genecards.org/cgi-bin/carddisp.pl?gene=SLC25A10
APOD	Apolipoprotein D	Protein Coding	P05090	46	GC03M195568	1.173492	https://www.genecards.org/cgi-bin/carddisp.pl?gene=APOD
DPF2	Double PHD Fingers 2	Protein Coding	Q92785	47	GC11P079847	1.172862	https://www.genecards.org/cgi-bin/carddisp.pl?gene=DPF2
SEC24D	SEC24 Homolog D, COPII Coat Complex Component	Protein Coding	O94855	47	GC04M118722	1.172862	https://www.genecards.org/cgi-bin/carddisp.pl?gene=SEC24D
LITAF	Lipopolysaccharide Induced TNF Factor	Protein Coding	Q99732	47	GC16M011547	1.169413	https://www.genecards.org/cgi-bin/carddisp.pl?gene=LITAF
LAT2	Linker For Activation Of T Cells Family Member 2	Protein Coding	Q9GZY6	44	GC07P074199	1.168318	https://www.genecards.org/cgi-bin/carddisp.pl?gene=LAT2
SUMO1	Small Ubiquitin Like Modifier 1	Protein Coding	P63165	51	GC02M202206	1.168267	https://www.genecards.org/cgi-bin/carddisp.pl?gene=SUMO1

FPR2	Formyl Peptide Receptor 2	Protein Coding	P25090	50	GC19P051752	1.167442	https://www.genecards.org/cgi-bin/carddisp.pl?gene=FPR2
ADAMTS1	ADAM Metallopeptidase With Thrombospondin Type 1 Motif 1	Protein Coding	Q9UHI8	49	GC21M026835	1.166103	https://www.genecards.org/cgi-bin/carddisp.pl?gene=ADAMTS1
RUNX3	RUNX Family Transcription Factor 3	Protein Coding	Q13761	47	GC01M024899	1.163604	https://www.genecards.org/cgi-bin/carddisp.pl?gene=RUNX3
PLA2G10	Phospholipase A2 Group X	Protein Coding	O15496	47	GC16M014672	1.161872	https://www.genecards.org/cgi-bin/carddisp.pl?gene=PLA2G10
FGF3	Fibroblast Growth Factor 3	Protein Coding	P11487	49	GC11M113744	1.161182	https://www.genecards.org/cgi-bin/carddisp.pl?gene=FGF3
FBLN5	Fibulin 5	Protein Coding	Q9UBX5	51	GC14M091869	1.157996	https://www.genecards.org/cgi-bin/carddisp.pl?gene=FBLN5
TRV-AAC1-1	TRNA-Val (Anticodon AAC) 1-1	RNA Gene		9	GC03P169772	1.157733	https://www.genecards.org/cgi-bin/carddisp.pl?gene=TRV-AAC1-1
TRV-AAC1-3	TRNA-Val (Anticodon AAC) 1-3	RNA Gene		9	GC05P181169	1.157733	https://www.genecards.org/cgi-bin/carddisp.pl?gene=TRV-AAC1-3
TRV-AAC1-4	TRNA-Val (Anticodon AAC) 1-4	RNA Gene		9	GC05M181218	1.157733	https://www.genecards.org/cgi-bin/carddisp.pl?gene=TRV-AAC1-4
TRV-AAC1-2	TRNA-Val (Anticodon AAC) 1-2	RNA Gene		8	GC05P181164	1.157733	https://www.genecards.org/cgi-bin/carddisp.pl?gene=TRV-AAC1-2
TRV-AAC1-5	TRNA-Val (Anticodon AAC) 1-5	RNA Gene		8	GC06M027753	1.157733	https://www.genecards.org/cgi-bin/carddisp.pl?gene=TRV-AAC1-5
SLC3A2	Solute Carrier Family 3 Member 2	Protein Coding	P08195	47	GC11P062856	1.157111	https://www.genecards.org/cgi-bin/carddisp.pl?gene=SLC3A2

LAMA5	Laminin Subunit Alpha 5	Protein Coding	O15230	48	GC20M062307	1.156664	https://www.genecards.org/cgi-bin/carddisp.pl?gene=LAMA5
SLC35A2	Solute Carrier Family 35 Member A2	Protein Coding	P78381	45	GC0XM048903	1.156494	https://www.genecards.org/cgi-bin/carddisp.pl?gene=SLC35A2
CCL15-CCL14	CCL15-CCL14 Readthrough (NMD Candidate)	RNA Gene		12	GC17M035983	1.155518	https://www.genecards.org/cgi-bin/carddisp.pl?gene=CCL15-CCL14
CHEK1	Checkpoint Kinase 1	Protein Coding	O14757	54	GC11P125625	1.155004	https://www.genecards.org/cgi-bin/carddisp.pl?gene=CHEK1
KCNB1	Potassium Voltage-Gated Channel Subfamily B Member 1	Protein Coding	Q14721	51	GC20M049293	1.15467	https://www.genecards.org/cgi-bin/carddisp.pl?gene=KCNB1
SLMAP	Sarcolemma Associated Protein	Protein Coding	Q14BN4	43	GC03P057943	1.15467	https://www.genecards.org/cgi-bin/carddisp.pl?gene=SLMAP
SCARNA14	Small Cajal Body-Specific RNA 14	RNA Gene		14	GC15M066347	1.15467	https://www.genecards.org/cgi-bin/carddisp.pl?gene=SCARNA14
XPA	XPA, DNA Damage Recognition And Repair Factor	Protein Coding	P23025	49	GC09M097654	1.154522	https://www.genecards.org/cgi-bin/carddisp.pl?gene=XPA
PDHB	Pyruvate Dehydrogenase E1 Subunit Beta	Protein Coding	P11177	50	GC03M058428	1.154508	https://www.genecards.org/cgi-bin/carddisp.pl?gene=PDHB
ATP5MK	ATP Synthase Membrane Subunit K	Protein Coding	Q96IX5	39	GC10M103397	1.154508	https://www.genecards.org/cgi-bin/carddisp.pl?gene=ATP5MK
TRIM22	Tripartite Motif Containing 22	Protein Coding	Q8IYM9	44	GC11P005689	1.153145	https://www.genecards.org/cgi-bin/carddisp.pl?gene=TRIM22
NPM2	Nucleophosmin/Nucleoplasmin 2	Protein Coding	Q86SE8	40	GC08P022024	1.152794	https://www.genecards.org/cgi-bin/carddisp.pl?gene=NPM2

HPSE2	Heparanase 2 (Inactive)	Protein Coding	Q8WWQ2	46	GC10M098457	1.152208	https://www.genecards.org/cgi-bin/carddisp.pl?gene=HPSE2
DNM3OS	DNM3 Opposite Strand/Antisense RNA	RNA Gene		20	GC01M172226	1.152208	https://www.genecards.org/cgi-bin/carddisp.pl?gene=DNM3OS
PSMD4	Proteasome 26S Subunit Ubiquitin Receptor, Non-ATPase 4	Protein Coding	P55036	48	GC01P156840	1.152197	https://www.genecards.org/cgi-bin/carddisp.pl?gene=PSMD4
PSMD14	Proteasome 26S Subunit, Non-ATPase 14	Protein Coding	O00487	47	GC02P161308	1.152197	https://www.genecards.org/cgi-bin/carddisp.pl?gene=PSMD14
ECT2	Epithelial Cell Transforming 2	Protein Coding	Q9H8V3	46	GC03P172750	1.151942	https://www.genecards.org/cgi-bin/carddisp.pl?gene=ECT2
TAS1R3	Taste 1 Receptor Member 3	Protein Coding	Q7RTX0	42	GC01P001331	1.15155	https://www.genecards.org/cgi-bin/carddisp.pl?gene=TAS1R3
SLC25A20	Solute Carrier Family 25 Member 20	Protein Coding	O43772	49	GC03M048909	1.150105	https://www.genecards.org/cgi-bin/carddisp.pl?gene=SLC25A20
NID1	Nidogen 1	Protein Coding	P14543	47	GC01M235975	1.149314	https://www.genecards.org/cgi-bin/carddisp.pl?gene=NID1
CSF3R	Colony Stimulating Factor 3 Receptor	Protein Coding	Q99062	51	GC01M036466	1.149174	https://www.genecards.org/cgi-bin/carddisp.pl?gene=CSF3R
MAPK9	Mitogen-Activated Protein Kinase 9	Protein Coding	P45984	52	GC05M180283	1.148441	https://www.genecards.org/cgi-bin/carddisp.pl?gene=MAPK9
SCNN1B	Sodium Channel Epithelial 1 Subunit Beta	Protein Coding	P51168	52	GC16P023278	1.147084	https://www.genecards.org/cgi-bin/carddisp.pl?gene=SCNN1B
MRC1	Mannose Receptor C-Type 1	Protein Coding	P22897	44	GC10P017809	1.14685	https://www.genecards.org/cgi-bin/carddisp.pl?gene=MRC1

CCL26	C-C Motif Chemokine Ligand 26	Protein Coding	Q9Y258	42	GC07M075769	1.14685	https://www.genecards.org/cgi-bin/carddisp.pl?gene=CCL26
LMO3	LIM Domain Only 3	Protein Coding	Q8TAP4	42	GC12M016548	1.14685	https://www.genecards.org/cgi-bin/carddisp.pl?gene=LMO3
MIR218-2	MicroRNA 218-2	RNA Gene		21	GC05M168768	1.14685	https://www.genecards.org/cgi-bin/carddisp.pl?gene=MIR218-2
XRCC2	X-Ray Repair Cross Complementing 2	Protein Coding	O43543	45	GC07M152644	1.143231	https://www.genecards.org/cgi-bin/carddisp.pl?gene=XRCC2
BUB1	BUB1 Mitotic Checkpoint Serine/Threonine Kinase	Protein Coding	O43683	53	GC02M110637	1.140931	https://www.genecards.org/cgi-bin/carddisp.pl?gene=BUB1
EFHC2	EF-Hand Domain Containing 2	Protein Coding	Q5JST6	38	GC0XM044146	1.140845	https://www.genecards.org/cgi-bin/carddisp.pl?gene=EFHC2
SLC22A1	Solute Carrier Family 22 Member 1	Protein Coding	O15245	47	GC06P160121	1.139866	https://www.genecards.org/cgi-bin/carddisp.pl?gene=SLC22A1
KLK1	Kallikrein 1	Protein Coding	P06870	51	GC19M050819	1.13904	https://www.genecards.org/cgi-bin/carddisp.pl?gene=KLK1
NLRC4	NLR Family CARD Domain Containing 4	Protein Coding	Q9NPP4	48	GC02M032224	1.138194	https://www.genecards.org/cgi-bin/carddisp.pl?gene=NLRC4
CEP41	Centrosomal Protein 41	Protein Coding	Q9BYV8	46	GC07M130393	1.138098	https://www.genecards.org/cgi-bin/carddisp.pl?gene=CEP41
FLOT1	Flotillin 1	Protein Coding	O75955	46	GC06M083877	1.138098	https://www.genecards.org/cgi-bin/carddisp.pl?gene=FLOT1
COPB1	COPI Coat Complex Subunit Beta 1	Protein Coding	P53618	45	GC11M014436	1.137755	https://www.genecards.org/cgi-bin/carddisp.pl?gene=COPB1

ITGA3	Integrin Subunit Alpha 3	Protein Coding	P26006	52	GC17P050055	1.136708	https://www.genecards.org/cgi-bin/carddisp.pl?gene=ITGA3
ATR	ATR Serine/Threonine Kinase	Protein Coding	Q13535	57	GC03M142449	1.136174	https://www.genecards.org/cgi-bin/carddisp.pl?gene=ATR
GRM7	Glutamate Metabotropic Receptor 7	Protein Coding	Q14831	50	GC03P006770	1.136174	https://www.genecards.org/cgi-bin/carddisp.pl?gene=GRM7
ACO1	Aconitase 1	Protein Coding	P21399	48	GC09P032374	1.136144	https://www.genecards.org/cgi-bin/carddisp.pl?gene=ACO1
ETHE1	ETHE1 Persulfide Dioxygenase	Protein Coding	O95571	47	GC19M043506	1.135621	https://www.genecards.org/cgi-bin/carddisp.pl?gene=ETHE1
SELENOK	Selenoprotein K	Protein Coding	Q9Y6D0	34	GC03M053884	1.134773	https://www.genecards.org/cgi-bin/carddisp.pl?gene=SELENOK
TNXB	Tenascin XB	Protein Coding	P22105	47	GC06M083976	1.13401	https://www.genecards.org/cgi-bin/carddisp.pl?gene=TNXB
ELF3	E74 Like ETS Transcription Factor 3	Protein Coding	P78545	44	GC01P202007	1.133581	https://www.genecards.org/cgi-bin/carddisp.pl?gene=ELF3
SLCO1B3	Solute Carrier Organic Anion Transporter Family Member 1B3	Protein Coding	Q9NPD5	48	GC12P020810	1.132987	https://www.genecards.org/cgi-bin/carddisp.pl?gene=SLCO1B3
CBX5	Chromobox 5	Protein Coding	P45973	47	GC12M054230	1.13194	https://www.genecards.org/cgi-bin/carddisp.pl?gene=CBX5
MIR4286	MicroRNA 4286	RNA Gene		16	GC08P010666	1.130692	https://www.genecards.org/cgi-bin/carddisp.pl?gene=MIR4286
KCNN3	Potassium Calcium-Activated Channel Subfamily N Member 3	Protein Coding	Q9UGI6	47	GC01M154697	1.130658	https://www.genecards.org/cgi-bin/carddisp.pl?gene=KCNN3

LIN28B	Lin-28 Homolog B	Protein Coding	Q6ZN17	46	GC06P112941	1.129535	https://www.genecards.org/cgi-bin/carddisp.pl?gene=LIN28B
SC5D	Sterol-C5-Desaturase	Protein Coding	O75845	45	GC11P121292	1.128964	https://www.genecards.org/cgi-bin/carddisp.pl?gene=SC5D
KCNE1	Potassium Voltage-Gated Channel Subfamily E Regulatory Subunit 1	Protein Coding	P15382	50	GC21M034446	1.128958	https://www.genecards.org/cgi-bin/carddisp.pl?gene=KCNE1
TET1	Tet Methylcytosine Dioxygenase 1	Protein Coding	Q8NFU7	42	GC10P068560	1.128405	https://www.genecards.org/cgi-bin/carddisp.pl?gene=TET1
CLEC4A	C-Type Lectin Domain Family 4 Member A	Protein Coding	Q9UMR7	43	GC12P030528	1.128139	https://www.genecards.org/cgi-bin/carddisp.pl?gene=CLEC4A
CFHR2	Complement Factor H Related 2	Protein Coding	P36980	44	GC01P196943	1.127708	https://www.genecards.org/cgi-bin/carddisp.pl?gene=CFHR2
CRYAA	Crystallin Alpha A	Protein Coding	P02489	50	GC21P043169	1.12733	https://www.genecards.org/cgi-bin/carddisp.pl?gene=CRYAA
MIR874	MicroRNA 874	RNA Gene		19	GC05M137647	1.126451	https://www.genecards.org/cgi-bin/carddisp.pl?gene=MIR874
RAMP2	Receptor Activity Modifying Protein 2	Protein Coding	O60895	47	GC17P042758	1.126168	https://www.genecards.org/cgi-bin/carddisp.pl?gene=RAMP2
COL9A1	Collagen Type IX Alpha 1 Chain	Protein Coding	P20849	46	GC06M070215	1.125641	https://www.genecards.org/cgi-bin/carddisp.pl?gene=COL9A1
PIAS1	Protein Inhibitor Of Activated STAT 1	Protein Coding	O75925	50	GC15P068054	1.124549	https://www.genecards.org/cgi-bin/carddisp.pl?gene=PIAS1
ZFP36	ZFP36 Ring Finger Protein	Protein Coding	P26651	42	GC19P039406	1.122552	https://www.genecards.org/cgi-bin/carddisp.pl?gene=ZFP36

IRF3	Interferon Regulatory Factor 3	Protein Coding	Q14653	48	GC19M049659	1.121377	https://www.genecards.org/cgi-bin/carddisp.pl?gene=IRF3
GRIK1	Glutamate Ionotropic Receptor Kainate Type Subunit 1	Protein Coding	P39086	49	GC21M029536	1.119947	https://www.genecards.org/cgi-bin/carddisp.pl?gene=GRIK1
PDCD4	Programmed Cell Death 4	Protein Coding	Q53EL6	47	GC10P110871	1.119749	https://www.genecards.org/cgi-bin/carddisp.pl?gene=PDCD4
SLC22A18AS	SLC22A18 Antisense RNA	RNA Gene	Q8N1D0	33	GC11M009035	1.119639	https://www.genecards.org/cgi-bin/carddisp.pl?gene=SLC22A18AS
NPR2	Natriuretic Peptide Receptor 2	Protein Coding	P20594	52	GC09P053235	1.116588	https://www.genecards.org/cgi-bin/carddisp.pl?gene=NPR2
DNAI1	Dynein Axonemal Intermediate Chain 1	Protein Coding	Q9UI46	45	GC09P034457	1.116549	https://www.genecards.org/cgi-bin/carddisp.pl?gene=DNAI1
MIR496	MicroRNA 496	RNA Gene		16	GC14P113614	1.116402	https://www.genecards.org/cgi-bin/carddisp.pl?gene=MIR496
AF346975		RNA Gene		4	GC01M010490	1.116402	https://www.genecards.org/cgi-bin/carddisp.pl?gene=AF346975
STC1	Stanniocalcin 1	Protein Coding	P52823	45	GC08M023841	1.115772	https://www.genecards.org/cgi-bin/carddisp.pl?gene=STC1
TSG101	Tumor Susceptibility 101	Protein Coding	Q99816	48	GC11M018468	1.114172	https://www.genecards.org/cgi-bin/carddisp.pl?gene=TSG101
ITPK1	Inositol-Tetrakisphosphate 1-Kinase	Protein Coding	Q13572	42	GC14M092936	1.114172	https://www.genecards.org/cgi-bin/carddisp.pl?gene=ITPK1
CNGA3	Cyclic Nucleotide Gated Channel Subunit Alpha 3	Protein Coding	Q16281	47	GC02P098350	1.113371	https://www.genecards.org/cgi-bin/carddisp.pl?gene=CNGA3

GAP43	Growth Associated Protein 43	Protein Coding	P17677	47	GC03P115623	1.111747	https://www.genecards.org/cgi-bin/carddisp.pl?gene=GAP43
LAMA1	Laminin Subunit Alpha 1	Protein Coding	P25391	50	GC18M006941	1.11156	https://www.genecards.org/cgi-bin/carddisp.pl?gene=LAMA1
COL20A1	Collagen Type XX Alpha 1 Chain	Protein Coding	Q9P218	41	GC20P063293	1.11156	https://www.genecards.org/cgi-bin/carddisp.pl?gene=COL20A1
MIR138-2	MicroRNA 138-2	RNA Gene		21	GC16P059603	1.11156	https://www.genecards.org/cgi-bin/carddisp.pl?gene=MIR138-2
LRP6	LDL Receptor Related Protein 6	Protein Coding	O75581	51	GC12M026002	1.111171	https://www.genecards.org/cgi-bin/carddisp.pl?gene=LRP6
GP9	Glycoprotein IX Platelet	Protein Coding	P14770	51	GC03P136804	1.110755	https://www.genecards.org/cgi-bin/carddisp.pl?gene=GP9
KRT8	Keratin 8	Protein Coding	P05787	51	GC12M052897	1.109874	https://www.genecards.org/cgi-bin/carddisp.pl?gene=KRT8
MTA1	Metastasis Associated 1	Protein Coding	Q13330	46	GC14P105419	1.109616	https://www.genecards.org/cgi-bin/carddisp.pl?gene=MTA1
PPBP	Pro-Platelet Basic Protein	Protein Coding	P02775	46	GC04M073986	1.109614	https://www.genecards.org/cgi-bin/carddisp.pl?gene=PPBP
FOXG1	Forkhead Box G1	Protein Coding	P55316	49	GC14P040221	1.109469	https://www.genecards.org/cgi-bin/carddisp.pl?gene=FOXG1
PLOD2	Procollagen-Lysine,2-Oxoglutarate 5-Dioxygenase 2	Protein Coding	O00469	50	GC03M146035	1.108588	https://www.genecards.org/cgi-bin/carddisp.pl?gene=PLOD2
MACROH2A1	MacroH2A.1 Histone	Protein Coding	O75367	47	GC05M135334	1.108588	https://www.genecards.org/cgi-bin/carddisp.pl?gene=MACROH2A1

OGDH	Oxoglutarate Dehydrogenase	Protein Coding	Q02218	51	GC07P044606	1.108536	https://www.genecards.org/cgi-bin/carddisp.pl?gene=OGDH
ACAD8	Acyl-CoA Dehydrogenase Family Member 8	Protein Coding	Q9UKU7	48	GC11P134253	1.106174	https://www.genecards.org/cgi-bin/carddisp.pl?gene=ACAD8
DSC3	Desmocollin 3	Protein Coding	Q14574	47	GC18M031158	1.106174	https://www.genecards.org/cgi-bin/carddisp.pl?gene=DSC3
DEPTOR	DEP Domain Containing MTOR Interacting Protein	Protein Coding	Q8TB45	45	GC08P119873	1.106174	https://www.genecards.org/cgi-bin/carddisp.pl?gene=DEPTOR
UBE2E2	Ubiquitin Conjugating Enzyme E2 E2	Protein Coding	Q96LR5	44	GC03P023221	1.106003	https://www.genecards.org/cgi-bin/carddisp.pl?gene=UBE2E2
ST8SIA6-AS1	ST8SIA6 Antisense RNA 1	RNA Gene		17	GC10P017386	1.105953	https://www.genecards.org/cgi-bin/carddisp.pl?gene=ST8SIA6-AS1
GJB1	Gap Junction Protein Beta 1	Protein Coding	P08034	50	GC0XP071212	1.105428	https://www.genecards.org/cgi-bin/carddisp.pl?gene=GJB1
WWTR1	WW Domain Containing Transcription Regulator 1	Protein Coding	Q9GZV5	44	GC03M149517	1.105345	https://www.genecards.org/cgi-bin/carddisp.pl?gene=WWTR1
EGR2	Early Growth Response 2	Protein Coding	P11161	48	GC10M062811	1.105228	https://www.genecards.org/cgi-bin/carddisp.pl?gene=EGR2
MYLIP	Myosin Regulatory Light Chain Interacting Protein	Protein Coding	Q8WY64	44	GC06P016129	1.105228	https://www.genecards.org/cgi-bin/carddisp.pl?gene=MYLIP
COL4A2-AS2	COL4A2 Antisense RNA 2	RNA Gene		20	GC13M110455	1.105228	https://www.genecards.org/cgi-bin/carddisp.pl?gene=COL4A2-AS2
CD74	CD74 Molecule	Protein Coding	P04233	48	GC05M150378	1.101902	https://www.genecards.org/cgi-bin/carddisp.pl?gene=CD74

GPC3	Glypican 3	Protein Coding	P51654	51	GC0XM133535	1.098597	https://www.genecards.org/cgi-bin/carddisp.pl?gene=GPC3
GYG1	Glycogenin 1	Protein Coding	P46976	51	GC03P148991	1.097922	https://www.genecards.org/cgi-bin/carddisp.pl?gene=GYG1
HIBCH	3-Hydroxyisobutyryl-CoA Hydrolase	Protein Coding	Q6NVY1	46	GC02M190189	1.096984	https://www.genecards.org/cgi-bin/carddisp.pl?gene=HIBCH
CCT3	Chaperonin Containing TCP1 Subunit 3	Protein Coding	P49368	46	GC01M156308	1.096871	https://www.genecards.org/cgi-bin/carddisp.pl?gene=CCT3
IGF2BP1	Insulin Like Growth Factor 2 MRNA Binding Protein 1	Protein Coding	Q9NZI8	45	GC17P086672	1.096846	https://www.genecards.org/cgi-bin/carddisp.pl?gene=IGF2BP1
NOX5	NADPH Oxidase 5	Protein Coding	Q96PH1	41	GC15P137164	1.095976	https://www.genecards.org/cgi-bin/carddisp.pl?gene=NOX5
KPNB1	Karyopherin Subunit Beta 1	Protein Coding	Q14974	48	GC17P047649	1.095614	https://www.genecards.org/cgi-bin/carddisp.pl?gene=KPNB1
CCL8	C-C Motif Chemokine Ligand 8	Protein Coding	P80075	42	GC17P034319	1.094877	https://www.genecards.org/cgi-bin/carddisp.pl?gene=CCL8
PRDM9	PR/SET Domain 9	Protein Coding	Q9NQV7	40	GC05P023443	1.094808	https://www.genecards.org/cgi-bin/carddisp.pl?gene=PRDM9
TNFRSF10A	TNF Receptor Superfamily Member 10a	Protein Coding	O00220	49	GC08M023190	1.094575	https://www.genecards.org/cgi-bin/carddisp.pl?gene=TNFRSF10A
RASGRF2	Ras Protein Specific Guanine Nucleotide Releasing Factor 2	Protein Coding	O14827	42	GC05P080960	1.09435	https://www.genecards.org/cgi-bin/carddisp.pl?gene=RASGRF2
TXNDC16	Thioredoxin Domain Containing 16	Protein Coding	Q9P2K2	38	GC14M052430	1.09435	https://www.genecards.org/cgi-bin/carddisp.pl?gene=TXNDC16

PCMT1	Protein-L-Isoaspartate (D-Aspartate) O-Methyltransferase	Protein Coding	P22061	44	GC06P149749	1.092434	https://www.genecards.org/cgi-bin/carddisp.pl?gene=PCMT1
TPM1	Tropomyosin 1	Protein Coding	P09493	53	GC15P141633	1.091075	https://www.genecards.org/cgi-bin/carddisp.pl?gene=TPM1
CD247	CD247 Molecule	Protein Coding	P20963	53	GC01M167399	1.089687	https://www.genecards.org/cgi-bin/carddisp.pl?gene=CD247
TPBG	Trophoblast Glycoprotein	Protein Coding	Q13641	46	GC06P112639	1.089687	https://www.genecards.org/cgi-bin/carddisp.pl?gene=TPBG
PRODH	Proline Dehydrogenase 1	Protein Coding	O43272	49	GC22M018912	1.089398	https://www.genecards.org/cgi-bin/carddisp.pl?gene=PRODH
ADCY1	Adenylate Cyclase 1	Protein Coding	Q08828	52	GC07P045580	1.089386	https://www.genecards.org/cgi-bin/carddisp.pl?gene=ADCY1
LTB	Lymphotoxin Beta	Protein Coding	Q06643	45	GC06M083953	1.087936	https://www.genecards.org/cgi-bin/carddisp.pl?gene=LTB
PDXK	Pyridoxal Kinase	Protein Coding	O00764	51	GC21P043719	1.087735	https://www.genecards.org/cgi-bin/carddisp.pl?gene=PDXK
MIR431	MicroRNA 431	RNA Gene		22	GC14P112880	1.087735	https://www.genecards.org/cgi-bin/carddisp.pl?gene=MIR431
LOC108449888	PATRR22 Recombination Region	Functional Element		2	GC22U901439	1.087735	https://www.genecards.org/cgi-bin/carddisp.pl?gene=LOC108449888
ABCB11	ATP Binding Cassette Subfamily B Member 11	Protein Coding	O95342	50	GC02M168922	1.087072	https://www.genecards.org/cgi-bin/carddisp.pl?gene=ABCB11
GABRA2	Gamma-Aminobutyric Acid Type A Receptor Subunit Alpha2	Protein Coding	P47869	51	GC04M046243	1.086783	https://www.genecards.org/cgi-bin/carddisp.pl?gene=GABRA2

AMHR2	Anti-Mullerian Hormone Receptor Type 2	Protein Coding	Q16671	52	GC12P053423	1.085197	https://www.genecards.org/cgi-bin/carddisp.pl?gene=AMHR2
SSC4D	Scavenger Receptor Cysteine Rich Family Member With 4 Domains	Protein Coding	Q8WTU2	34	GC07M078083	1.083433	https://www.genecards.org/cgi-bin/carddisp.pl?gene=SSC4D
WARS1	Tryptophanyl-TRNA Synthetase 1	Protein Coding	P23381	51	GC14M116458	1.081518	https://www.genecards.org/cgi-bin/carddisp.pl?gene=WARS1
LINC00861	Long Intergenic Non-Protein Coding RNA 861	RNA Gene		16	GC08M134444	1.081518	https://www.genecards.org/cgi-bin/carddisp.pl?gene=LINC00861
CDK20	Cyclin Dependent Kinase 20	Protein Coding	Q8IZL9	45	GC09M087966	1.080088	https://www.genecards.org/cgi-bin/carddisp.pl?gene=CDK20
TRAPPC11	Trafficking Protein Particle Complex Subunit 11	Protein Coding	Q7Z392	40	GC04P183659	1.080088	https://www.genecards.org/cgi-bin/carddisp.pl?gene=TRAPPC11
ZNF318	Zinc Finger Protein 318	Protein Coding	Q5VUA4	40	GC06M084193	1.07959	https://www.genecards.org/cgi-bin/carddisp.pl?gene=ZNF318
DOCK8	Dedicator Of Cytokinesis 8	Protein Coding	Q8NF50	49	GC09P000225	1.078382	https://www.genecards.org/cgi-bin/carddisp.pl?gene=DOCK8
EFNA1	Ephrin A1	Protein Coding	P20827	47	GC01P155127	1.078382	https://www.genecards.org/cgi-bin/carddisp.pl?gene=EFNA1
GRIN3A	Glutamate Ionotropic Receptor NMDA Type Subunit 3A	Protein Coding	Q8TCU5	42	GC09M101569	1.078382	https://www.genecards.org/cgi-bin/carddisp.pl?gene=GRIN3A
SAMD4B	Sterile Alpha Motif Domain Containing 4B	Protein Coding	Q5PRF9	41	GC19P039342	1.078382	https://www.genecards.org/cgi-bin/carddisp.pl?gene=SAMD4B
LOC101927284	Uncharacterized LOC101927284	RNA Gene		7	GC13P094761	1.078382	https://www.genecards.org/cgi-bin/carddisp.pl?gene=LOC101927284

SIM2	SIM BHLH Transcription Factor 2	Protein Coding	Q14190	43	GC21P036699	1.076091	https://www.genecards.org/cgi-bin/carddisp.pl?gene=SIM2
COL11A1	Collagen Type XI Alpha 1 Chain	Protein Coding	P12107	47	GC01M102876	1.075403	https://www.genecards.org/cgi-bin/carddisp.pl?gene=COL11A1
PKP1	Plakophilin 1	Protein Coding	Q13835	45	GC01P201283	1.075403	https://www.genecards.org/cgi-bin/carddisp.pl?gene=PKP1
CHD4	Chromodomain Helicase DNA Binding Protein 4	Protein Coding	Q14839	49	GC12M006570	1.075183	https://www.genecards.org/cgi-bin/carddisp.pl?gene=CHD4
DLD	Dihydrolipoamide Dehydrogenase	Protein Coding	P09622	53	GC07P107890	1.075111	https://www.genecards.org/cgi-bin/carddisp.pl?gene=DLD
MIR1260A	MicroRNA 1260a	RNA Gene		15	GC14P077266	1.074735	https://www.genecards.org/cgi-bin/carddisp.pl?gene=MIR1260A
MIR664A	MicroRNA 664a	RNA Gene		13	GC01M220200	1.072587	https://www.genecards.org/cgi-bin/carddisp.pl?gene=MIR664A
DST	Dystonin	Protein Coding	Q03001	46	GC06M056457	1.067765	https://www.genecards.org/cgi-bin/carddisp.pl?gene=DST
ADORA3	Adenosine A3 Receptor	Protein Coding	P0DMS8	48	GC01M111499	1.066966	https://www.genecards.org/cgi-bin/carddisp.pl?gene=ADORA3
DBT	Dihydrolipoamide Branched Chain Transacylase E2	Protein Coding	P11182	47	GC01M100186	1.066966	https://www.genecards.org/cgi-bin/carddisp.pl?gene=DBT
VPS26A	VPS26 Retromer Complex Component A	Protein Coding	O75436	44	GC10P069123	1.066966	https://www.genecards.org/cgi-bin/carddisp.pl?gene=VPS26A
MIR320C2	MicroRNA 320c-2	RNA Gene		14	GC18P024321	1.066966	https://www.genecards.org/cgi-bin/carddisp.pl?gene=MIR320C2

AIFM1	Apoptosis Inducing Factor Mitochondria Associated 1	Protein Coding	O95831	54	GC0XM130129	1.066764	https://www.genecards.org/cgi-bin/carddisp.pl?gene=AIFM1
PPM1K	Protein Phosphatase, Mg2+/Mn2+ Dependent 1K	Protein Coding	Q8N3J5	45	GC04M088258	1.065949	https://www.genecards.org/cgi-bin/carddisp.pl?gene=PPM1K
MCPH1	Microcephalin 1	Protein Coding	Q8NEM0	45	GC08P006406	1.065053	https://www.genecards.org/cgi-bin/carddisp.pl?gene=MCPH1
SNHG16	Small Nucleolar RNA Host Gene 16	RNA Gene		20	GC17P076544	1.064715	https://www.genecards.org/cgi-bin/carddisp.pl?gene=SNHG16
PSMB8	Proteasome 20S Subunit Beta 8	Protein Coding	P28062	54	GC06M032840	1.064233	https://www.genecards.org/cgi-bin/carddisp.pl?gene=PSMB8
SEMA5B	Semaphorin 5B	Protein Coding	Q9P283	43	GC03M122909	1.06325	https://www.genecards.org/cgi-bin/carddisp.pl?gene=SEMA5B
AIMP1	Aminoacyl TRNA Synthetase Complex Interacting Multifunctional Protein 1	Protein Coding	Q12904	50	GC04P106315	1.063202	https://www.genecards.org/cgi-bin/carddisp.pl?gene=AIMP1
IL17F	Interleukin 17F	Protein Coding	Q96PD4	46	GC06M084340	1.063202	https://www.genecards.org/cgi-bin/carddisp.pl?gene=IL17F
TFEB	Transcription Factor EB	Protein Coding	P19484	46	GC06M084156	1.062578	https://www.genecards.org/cgi-bin/carddisp.pl?gene=TFEB
UBE2I	Ubiquitin Conjugating Enzyme E2 I	Protein Coding	P63279	52	GC16P052380	1.062266	https://www.genecards.org/cgi-bin/carddisp.pl?gene=UBE2I
PUM1	Pumilio RNA Binding Family Member 1	Protein Coding	Q14671	45	GC01M030931	1.062266	https://www.genecards.org/cgi-bin/carddisp.pl?gene=PUM1
AREG	Amphiregulin	Protein Coding	P15514	47	GC04P074445	1.061896	https://www.genecards.org/cgi-bin/carddisp.pl?gene=AREG

NFKB2	Nuclear Factor Kappa B Subunit 2	Protein Coding	Q00653	56	GC10P102394	1.061566	https://www.genecards.org/cgi-bin/carddisp.pl?gene=NFKB2
GAN	Gigaxonin	Protein Coding	Q9H2C0	44	GC16P081319	1.060842	https://www.genecards.org/cgi-bin/carddisp.pl?gene=GAN
DRD5	Dopamine Receptor D5	Protein Coding	P21918	50	GC04P009783	1.060071	https://www.genecards.org/cgi-bin/carddisp.pl?gene=DRD5
ZNF438	Zinc Finger Protein 438	Protein Coding	Q7Z4V0	35	GC10M030820	1.059332	https://www.genecards.org/cgi-bin/carddisp.pl?gene=ZNF438
LOC111589215	BRCA1 Promoter Region	Functional Element		4	GC17P088099	1.05758	https://www.genecards.org/cgi-bin/carddisp.pl?gene=LOC111589215
MIR371A	MicroRNA 371a	RNA Gene		17	GC19P053787	1.055503	https://www.genecards.org/cgi-bin/carddisp.pl?gene=MIR371A
UGT1A4	UDP Glucuronosyltransferase Family 1 Member A4	Protein Coding	P22310	46	GC02P233718	1.055282	https://www.genecards.org/cgi-bin/carddisp.pl?gene=UGT1A4
FZD1	Frizzled Class Receptor 1	Protein Coding	Q9UP38	51	GC07P091264	1.055047	https://www.genecards.org/cgi-bin/carddisp.pl?gene=FZD1
FOXN3	Forkhead Box N3	Protein Coding	O00409	41	GC14M116361	1.055047	https://www.genecards.org/cgi-bin/carddisp.pl?gene=FOXN3
SALL3	Spalt Like Transcription Factor 3	Protein Coding	Q9BXA9	39	GC18P078980	1.055047	https://www.genecards.org/cgi-bin/carddisp.pl?gene=SALL3
NLGN4X	Neuroligin 4 X-Linked	Protein Coding	Q8N0W4	47	GC0XM005840	1.054547	https://www.genecards.org/cgi-bin/carddisp.pl?gene=NLGN4X
MIR184	MicroRNA 184	RNA Gene		23	GC15P079209	1.054546	https://www.genecards.org/cgi-bin/carddisp.pl?gene=MIR184

MIR512-2	MicroRNA 512-2	RNA Gene		14	GC19P090137	1.054546	https://www.genecards.org/cgi-bin/carddisp.pl?gene=MIR512-2
EFEMP2	EGF Containing Fibulin Extracellular Matrix Protein 2	Protein Coding	O95967	48	GC11M113564	1.054276	https://www.genecards.org/cgi-bin/carddisp.pl?gene=EFEMP2
TOLLIP	Toll Interacting Protein	Protein Coding	Q9H0E2	48	GC11M001274	1.054222	https://www.genecards.org/cgi-bin/carddisp.pl?gene=TOLLIP
ARHGAP42	Rho GTPase Activating Protein 42	Protein Coding	A6NI28	40	GC11P100687	1.053727	https://www.genecards.org/cgi-bin/carddisp.pl?gene=ARHGAP42
RAB11FIP5	RAB11 Family Interacting Protein 5	Protein Coding	Q9BXF6	44	GC02M073148	1.052598	https://www.genecards.org/cgi-bin/carddisp.pl?gene=RAB11FIP5
CHEK2	Checkpoint Kinase 2	Protein Coding	O96017	58	GC22M028687	1.052544	https://www.genecards.org/cgi-bin/carddisp.pl?gene=CHEK2
DSCR4	Down Syndrome Critical Region 4	RNA Gene	P56555	30	GC21M037951	1.051202	https://www.genecards.org/cgi-bin/carddisp.pl?gene=DSCR4
CYP51A1	Cytochrome P450 Family 51 Subfamily A Member 1	Protein Coding	Q16850	47	GC07M092112	1.050902	https://www.genecards.org/cgi-bin/carddisp.pl?gene=CYP51A1
CRMA	Cardiomyocyte Maturation Associated LncRNA	RNA Gene		21	GC20M062545	1.047816	https://www.genecards.org/cgi-bin/carddisp.pl?gene=CRMA
GATM	Glycine Amidinotransferase	Protein Coding	P50440	50	GC15M045361	1.047674	https://www.genecards.org/cgi-bin/carddisp.pl?gene=GATM
CHD2	Chromodomain Helicase DNA Binding Protein 2	Protein Coding	O14647	47	GC15P137814	1.047674	https://www.genecards.org/cgi-bin/carddisp.pl?gene=CHD2
EZR	Ezrin	Protein Coding	P15311	50	GC06M158765	1.046321	https://www.genecards.org/cgi-bin/carddisp.pl?gene=EZR

KLKB1	Kallikrein B1	Protein Coding	P03952	50	GC04P186274	1.045416	https://www.genecards.org/cgi-bin/carddisp.pl?gene=KLKB1
GRID2	Glutamate Ionotropic Receptor Delta Type Subunit 2	Protein Coding	O43424	49	GC04P092304	1.045416	https://www.genecards.org/cgi-bin/carddisp.pl?gene=GRID2
ICOS	Inducible T Cell Costimulator	Protein Coding	Q9Y6W8	49	GC02P203937	1.045416	https://www.genecards.org/cgi-bin/carddisp.pl?gene=ICOS
SLC6A5	Solute Carrier Family 6 Member 5	Protein Coding	Q9Y345	49	GC11P020599	1.045416	https://www.genecards.org/cgi-bin/carddisp.pl?gene=SLC6A5
CHST11	Carbohydrate Sulfotransferase 11	Protein Coding	Q9NPF2	47	GC12P104455	1.045416	https://www.genecards.org/cgi-bin/carddisp.pl?gene=CHST11
GRPR	Gastrin Releasing Peptide Receptor	Protein Coding	P30550	47	GC0XP016141	1.045416	https://www.genecards.org/cgi-bin/carddisp.pl?gene=GRPR
NCKAP1L	NCK Associated Protein 1 Like	Protein Coding	P55160	44	GC12P054497	1.045416	https://www.genecards.org/cgi-bin/carddisp.pl?gene=NCKAP1L
AOPEP	Aminopeptidase O (Putative)	Protein Coding	Q8N6M6	43	GC09P096181	1.045416	https://www.genecards.org/cgi-bin/carddisp.pl?gene=AOPEP
LINC00520	Long Intergenic Non-Protein Coding RNA 520	RNA Gene		19	GC14M055781	1.045416	https://www.genecards.org/cgi-bin/carddisp.pl?gene=LINC00520
REG1A	Regenerating Family Member 1 Alpha	Protein Coding	P05451	44	GC02P079120	1.04431	https://www.genecards.org/cgi-bin/carddisp.pl?gene=REG1A
SYT1	Synaptotagmin 1	Protein Coding	P21579	52	GC12P078863	1.043821	https://www.genecards.org/cgi-bin/carddisp.pl?gene=SYT1
CACNA2D1	Calcium Voltage-Gated Channel Auxiliary Subunit Alpha2delta 1	Protein Coding	P54289	51	GC07M081946	1.043821	https://www.genecards.org/cgi-bin/carddisp.pl?gene=CACNA2D1

CACNB2	Calcium Voltage-Gated Channel Auxiliary Subunit Beta 2	Protein Coding	Q08289	51	GC10P018165	1.043821	https://www.genecards.org/cgi-bin/carddisp.pl?gene=CACNB2
NDRG1	N-Myc Downstream Regulated 1	Protein Coding	Q92597	50	GC08M133237	1.043821	https://www.genecards.org/cgi-bin/carddisp.pl?gene=NDRG1
THSD7A	Thrombospondin Type 1 Domain Containing 7A	Protein Coding	Q9UPZ6	42	GC07M011371	1.043821	https://www.genecards.org/cgi-bin/carddisp.pl?gene=THSD7A
PTTG1	PTTG1 Regulator Of Sister Chromatid Separation, Securin	Protein Coding	O95997	45	GC05P160422	1.043091	https://www.genecards.org/cgi-bin/carddisp.pl?gene=PTTG1
MIR502	MicroRNA 502	RNA Gene		16	GC0XP050014	1.04049	https://www.genecards.org/cgi-bin/carddisp.pl?gene=MIR502
GNA12	G Protein Subunit Alpha 12	Protein Coding	Q03113	44	GC07M002728	1.039965	https://www.genecards.org/cgi-bin/carddisp.pl?gene=GNA12
DAG1	Dystroglycan 1	Protein Coding	Q14118	50	GC03P053919	1.038392	https://www.genecards.org/cgi-bin/carddisp.pl?gene=DAG1
RBM10	RNA Binding Motif Protein 10	Protein Coding	P0DW28	45	GC0XP047988	1.037448	https://www.genecards.org/cgi-bin/carddisp.pl?gene=RBM10
MAD2L1	Mitotic Arrest Deficient 2 Like 1	Protein Coding	Q13257	48	GC04M120055	1.037212	https://www.genecards.org/cgi-bin/carddisp.pl?gene=MAD2L1
DYNLL1	Dynein Light Chain LC8-Type 1	Protein Coding	P63167	46	GC12P120469	1.037126	https://www.genecards.org/cgi-bin/carddisp.pl?gene=DYNLL1
DCDC2	Doublecortin Domain Containing 2	Protein Coding	Q9UHG0	43	GC06M024171	1.037126	https://www.genecards.org/cgi-bin/carddisp.pl?gene=DCDC2
ARMC9	Armadillo Repeat Containing 9	Protein Coding	Q7Z3E5	42	GC02P231198	1.037126	https://www.genecards.org/cgi-bin/carddisp.pl?gene=ARMC9

INSM2	INSM Transcriptional Repressor 2	Protein Coding	Q96T92	39	GC14P035534	1.037126	https://www.genecards.org/cgi-bin/carddisp.pl?gene=INSM2
LOC126806306	P300/CBP Strongly-Dependent Group 1 Enhancer GRCh37_chr2:110906815-110908014	Functional Element		3	GC02P110149	1.037126	https://www.genecards.org/cgi-bin/carddisp.pl?gene=LOC126806306
FOXL2	Forkhead Box L2	Protein Coding	P58012	45	GC03M138944	1.036089	https://www.genecards.org/cgi-bin/carddisp.pl?gene=FOXL2
EPOR	Erythropoietin Receptor	Protein Coding	P19235	52	GC19M011377	1.035908	https://www.genecards.org/cgi-bin/carddisp.pl?gene=EPOR
GPI	Glucose-6-Phosphate Isomerase	Protein Coding	P06744	52	GC19P034359	1.035336	https://www.genecards.org/cgi-bin/carddisp.pl?gene=GPI
MIR377	MicroRNA 377	RNA Gene		20	GC14P113597	1.035336	https://www.genecards.org/cgi-bin/carddisp.pl?gene=MIR377
LMNB2	Lamin B2	Protein Coding	Q03252	47	GC19M010701	1.033865	https://www.genecards.org/cgi-bin/carddisp.pl?gene=LMNB2
ACTG1	Actin Gamma 1	Protein Coding	P63261	52	GC17M081509	1.032384	https://www.genecards.org/cgi-bin/carddisp.pl?gene=ACTG1
RND3	Rho Family GTPase 3	Protein Coding	P61587	45	GC02M150468	1.028691	https://www.genecards.org/cgi-bin/carddisp.pl?gene=RND3
UGT1A6	UDP Glucuronosyltransferase Family 1 Member A6	Protein Coding	P19224	46	GC02P233691	1.02749	https://www.genecards.org/cgi-bin/carddisp.pl?gene=UGT1A6
SLC9A6	Solute Carrier Family 9 Member A6	Protein Coding	Q92581	50	GC0XP135985	1.026667	https://www.genecards.org/cgi-bin/carddisp.pl?gene=SLC9A6
UBE2L3	Ubiquitin Conjugating Enzyme E2 L3	Protein Coding	P68036	49	GC22P021549	1.026667	https://www.genecards.org/cgi-bin/carddisp.pl?gene=UBE2L3

PSAT1	Phosphoserine Aminotransferase 1	Protein Coding	Q9Y617	50	GC09P078297	1.024004	https://www.genecards.org/cgi-bin/carddisp.pl?gene=PSAT1
ACKR4	Atypical Chemokine Receptor 4	Protein Coding	Q9NPB9	38	GC03P132597	1.024004	https://www.genecards.org/cgi-bin/carddisp.pl?gene=ACKR4
DINOL	Damage Induced Long Noncoding RNA	RNA Gene		11	GC06M084850	1.024004	https://www.genecards.org/cgi-bin/carddisp.pl?gene=DINOL
TRPM5	Transient Receptor Potential Cation Channel Subfamily M Member 5	Protein Coding	Q9NZQ8	42	GC11M009032	1.023189	https://www.genecards.org/cgi-bin/carddisp.pl?gene=TRPM5
KCNJ15	Potassium Inwardly Rectifying Channel Subfamily J Member 15	Protein Coding	Q99712	46	GC21P038157	1.022518	https://www.genecards.org/cgi-bin/carddisp.pl?gene=KCNJ15
ATN1	Atrophin 1	Protein Coding	P54259	47	GC12P030499	1.022045	https://www.genecards.org/cgi-bin/carddisp.pl?gene=ATN1
CLTRN	Collectrin, Amino Acid Transport Regulator	Protein Coding	Q9HBJ8	42	GC0XM015629	1.021836	https://www.genecards.org/cgi-bin/carddisp.pl?gene=CLTRN
MIR541	MicroRNA 541	RNA Gene		20	GC14P113616	1.021634	https://www.genecards.org/cgi-bin/carddisp.pl?gene=MIR541
BMAL2	Basic Helix-Loop-Helix ARNT Like 2	Protein Coding	Q8WYA1	41	GC12P031000	1.02158	https://www.genecards.org/cgi-bin/carddisp.pl?gene=BMAL2
MIR616	MicroRNA 616	RNA Gene		20	GC12M057519	1.02158	https://www.genecards.org/cgi-bin/carddisp.pl?gene=MIR616
TMEM256-PLSCR3	TMEM256-PLSCR3 Readthrough (NMD Candidate)	RNA Gene		20	GC17M014396	1.02158	https://www.genecards.org/cgi-bin/carddisp.pl?gene=TMEM256-PLSCR3
CLN3	CLN3 Lysosomal/Endosomal Transmembrane Protein, Battenin	Protein Coding	Q13286	48	GC16M028466	1.021401	https://www.genecards.org/cgi-bin/carddisp.pl?gene=CLN3

CD276	CD276 Molecule	Protein Coding	Q5ZPR3	46	GC15P073683	1.021081	https://www.genecards.org/cgi-bin/carddisp.pl?gene=CD276
IDS	Iduronate 2-Sulfatase	Protein Coding	P22304	52	GC0XM149476	1.021034	https://www.genecards.org/cgi-bin/carddisp.pl?gene=IDS
L1TD1	LINE1 Type Transposase Domain Containing 1	Protein Coding	Q5T7N2	39	GC01P062194	1.020761	https://www.genecards.org/cgi-bin/carddisp.pl?gene=L1TD1
RARS1	Arginyl-TRNA Synthetase 1	Protein Coding	P54136	48	GC05P168487	1.020106	https://www.genecards.org/cgi-bin/carddisp.pl?gene=RARS1
MIR19B2	MicroRNA 19b-2	RNA Gene		16	GC0XM134396	1.018628	https://www.genecards.org/cgi-bin/carddisp.pl?gene=MIR19B2
LOC110121471	VISTA Enhancer Hs1977	Functional Element		4	GC11P017412	1.017894	https://www.genecards.org/cgi-bin/carddisp.pl?gene=LOC110121471
YBX1	Y-Box Binding Protein 1	Protein Coding	P67809	45	GC01P042682	1.0132	https://www.genecards.org/cgi-bin/carddisp.pl?gene=YBX1
MIR1247	MicroRNA 1247	RNA Gene		18	GC14M101560	1.0132	https://www.genecards.org/cgi-bin/carddisp.pl?gene=MIR1247
STXBP1	Syntaxin Binding Protein 1	Protein Coding	P61764	53	GC09P127605	1.007852	https://www.genecards.org/cgi-bin/carddisp.pl?gene=STXBP1
DARS1	Aspartyl-TRNA Synthetase 1	Protein Coding	P14868	47	GC02M135905	1.007601	https://www.genecards.org/cgi-bin/carddisp.pl?gene=DARS1
ETV4	ETS Variant Transcription Factor 4	Protein Coding	P43268	47	GC17M043527	1.005192	https://www.genecards.org/cgi-bin/carddisp.pl?gene=ETV4
LCN1	Lipocalin 1	Protein Coding	P31025	44	GC09P135521	1.005192	https://www.genecards.org/cgi-bin/carddisp.pl?gene=LCN1

KCNQ2	Potassium Voltage-Gated Channel Subfamily Q Member 2	Protein Coding	O43526	51	GC20M063400	1.004581	https://www.genecards.org/cgi-bin/carddisp.pl?gene=KCNQ2
MIR491	MicroRNA 491	RNA Gene		20	GC09P020716	1.003581	https://www.genecards.org/cgi-bin/carddisp.pl?gene=MIR491
MAD2L2	Mitotic Arrest Deficient 2 Like 2	Protein Coding	Q9UI95	47	GC01M011658	1.003118	https://www.genecards.org/cgi-bin/carddisp.pl?gene=MAD2L2
FIS1	Fission, Mitochondrial 1	Protein Coding	Q9Y3D6	42	GC07M101239	1.002884	https://www.genecards.org/cgi-bin/carddisp.pl?gene=FIS1
LRRK2	Leucine Rich Repeat Kinase 2	Protein Coding	Q5S007	53	GC12P040196	1.002454	https://www.genecards.org/cgi-bin/carddisp.pl?gene=LRRK2
ITPR1	Inositol 1,4,5-Trisphosphate Receptor Type 1	Protein Coding	Q14643	52	GC03P004486	1.002454	https://www.genecards.org/cgi-bin/carddisp.pl?gene=ITPR1
CDK9	Cyclin Dependent Kinase 9	Protein Coding	P50750	50	GC09P130694	1.002454	https://www.genecards.org/cgi-bin/carddisp.pl?gene=CDK9
KLF5	KLF Transcription Factor 5	Protein Coding	Q13887	50	GC13P073054	1.002454	https://www.genecards.org/cgi-bin/carddisp.pl?gene=KLF5
KMO	Kynurenine 3-Monooxygenase	Protein Coding	O15229	50	GC01P241532	1.002454	https://www.genecards.org/cgi-bin/carddisp.pl?gene=KMO
CPA1	Carboxypeptidase A1	Protein Coding	P15085	48	GC07P130380	1.002454	https://www.genecards.org/cgi-bin/carddisp.pl?gene=CPA1
MATK	Megakaryocyte-Associated Tyrosine Kinase	Protein Coding	P42679	48	GC19M003777	1.002454	https://www.genecards.org/cgi-bin/carddisp.pl?gene=MATK
SIGLEC7	Sialic Acid Binding Ig Like Lectin 7	Protein Coding	Q9Y286	47	GC19P051142	1.002454	https://www.genecards.org/cgi-bin/carddisp.pl?gene=SIGLEC7

ANAPC1	Anaphase Promoting Complex Subunit 1	Protein Coding	Q9H1A4	46	GC02M111611	1.002454	https://www.genecards.org/cgi-bin/carddisp.pl?gene=ANAPC1
HAS2	Hyaluronan Synthase 2	Protein Coding	Q92819	46	GC08M121612	1.002454	https://www.genecards.org/cgi-bin/carddisp.pl?gene=HAS2
NUP153	Nucleoporin 153	Protein Coding	P49790	46	GC06M017615	1.002454	https://www.genecards.org/cgi-bin/carddisp.pl?gene=NUP153
RAMP1	Receptor Activity Modifying Protein 1	Protein Coding	O60894	46	GC02P237858	1.002454	https://www.genecards.org/cgi-bin/carddisp.pl?gene=RAMP1
DSCAM	DS Cell Adhesion Molecule	Protein Coding	O60469	45	GC21M040010	1.002454	https://www.genecards.org/cgi-bin/carddisp.pl?gene=DSCAM
JDP2	Jun Dimerization Protein 2	Protein Coding	Q8WYK2	45	GC14P075427	1.002454	https://www.genecards.org/cgi-bin/carddisp.pl?gene=JDP2
SPTB	Spectrin Beta, Erythrocytic	Protein Coding	P11277	45	GC14M064746	1.002454	https://www.genecards.org/cgi-bin/carddisp.pl?gene=SPTB
CPLX2	Complexin 2	Protein Coding	Q6PUV4	44	GC05P175797	1.002454	https://www.genecards.org/cgi-bin/carddisp.pl?gene=CPLX2
MIP	Major Intrinsic Protein Of Lens Fiber	Protein Coding	P30301	44	GC12M056449	1.002454	https://www.genecards.org/cgi-bin/carddisp.pl?gene=MIP
RSU1	Ras Suppressor Protein 1	Protein Coding	Q15404	44	GC10M016672	1.002454	https://www.genecards.org/cgi-bin/carddisp.pl?gene=RSU1
SLCO3A1	Solute Carrier Organic Anion Transporter Family Member 3A1	Protein Coding	Q9UIG8	44	GC15P137810	1.002454	https://www.genecards.org/cgi-bin/carddisp.pl?gene=SLCO3A1
CPSF2	Cleavage And Polyadenylation Specific Factor 2	Protein Coding	Q9P2I0	42	GC14P092121	1.002454	https://www.genecards.org/cgi-bin/carddisp.pl?gene=CPSF2

IFNL3	Interferon Lambda 3	Protein Coding	Q8IZI9	40	GC19M039243	1.002454	https://www.genecards.org/cgi-bin/carddisp.pl?gene=IFNL3
SRL	Sarcalumenin	Protein Coding	Q86TD4	39	GC16M004189	1.002454	https://www.genecards.org/cgi-bin/carddisp.pl?gene=SRL
CRACR2B	Calcium Release Activated Channel Regulator 2B	Protein Coding	Q8N4Y2	38	GC11P004478	1.002454	https://www.genecards.org/cgi-bin/carddisp.pl?gene=CRACR2B
FHDC1	FH2 Domain Containing 1	Protein Coding	Q9C0D6	38	GC04P152911	1.002454	https://www.genecards.org/cgi-bin/carddisp.pl?gene=FHDC1
GASK1A	Golgi Associated Kinase 1A	Protein Coding	Q9UFP1	35	GC03P043575	1.002454	https://www.genecards.org/cgi-bin/carddisp.pl?gene=GASK1A
NLRP8	NLR Family Pyrin Domain Containing 8	Protein Coding	Q86W28	35	GC19P090223	1.002454	https://www.genecards.org/cgi-bin/carddisp.pl?gene=NLRP8
OR52K1	Olfactory Receptor Family 52 Subfamily K Member 1	Protein Coding	Q8NGK4	33	GC11P004482	1.002454	https://www.genecards.org/cgi-bin/carddisp.pl?gene=OR52K1
OR52M1	Olfactory Receptor Family 52 Subfamily M Member 1	Protein Coding	Q8NGK5	30	GC11P004545	1.002454	https://www.genecards.org/cgi-bin/carddisp.pl?gene=OR52M1
C16orf96	Chromosome 16 Open Reading Frame 96	Protein Coding	A6NNT2	29	GC16P052516	1.002454	https://www.genecards.org/cgi-bin/carddisp.pl?gene=C16orf96
C11orf21	Chromosome 11 Open Reading Frame 21	Protein Coding	Q9P2W6	28	GC11M002295	1.002454	https://www.genecards.org/cgi-bin/carddisp.pl?gene=C11orf21
DIRC1	Disrupted In Renal Carcinoma 1	RNA Gene	Q969H9	28	GC02M188733	1.002454	https://www.genecards.org/cgi-bin/carddisp.pl?gene=DIRC1
LINCMD1	Long Intergenic Non-Protein Coding RNA, Muscle Differentiation 1	RNA Gene		18	GC06M052146	1.002454	https://www.genecards.org/cgi-bin/carddisp.pl?gene=LINCMD1

ELDR	EGFR Long Non-Coding Downstream RNA	RNA Gene		15	GC07M055235	1.002454	https://www.genecards.org/cgi-bin/carddisp.pl?gene=ELDR
MIR3163	MicroRNA 3163	RNA Gene		12	GC11M066934	1.002454	https://www.genecards.org/cgi-bin/carddisp.pl?gene=MIR3163
HBG2	Hemoglobin Subunit Gamma 2	Protein Coding	P69892	45	GC11M009156	0.998574	https://www.genecards.org/cgi-bin/carddisp.pl?gene=HBG2
HIF1A-AS1	HIF1A Antisense RNA 1	RNA Gene		17	GC14M061681	0.998174	https://www.genecards.org/cgi-bin/carddisp.pl?gene=HIF1A-AS1
MIR1-1	MicroRNA 1-1	RNA Gene		21	GC20P065678	0.994524	https://www.genecards.org/cgi-bin/carddisp.pl?gene=MIR1-1
MIR1283-1	MicroRNA 1283-1	RNA Gene		16	GC19P053688	0.994524	https://www.genecards.org/cgi-bin/carddisp.pl?gene=MIR1283-1
MIR1283-2	MicroRNA 1283-2	RNA Gene		15	GC19P053758	0.994524	https://www.genecards.org/cgi-bin/carddisp.pl?gene=MIR1283-2
PDE4A	Phosphodiesterase 4A	Protein Coding	P27815	49	GC19P010416	0.993291	https://www.genecards.org/cgi-bin/carddisp.pl?gene=PDE4A
VARS2	Valyl-TRNA Synthetase 2, Mitochondrial	Protein Coding	Q5ST30	46	GC06P111978	0.992127	https://www.genecards.org/cgi-bin/carddisp.pl?gene=VARS2
TOP2B	DNA Topoisomerase II Beta	Protein Coding	Q02880	50	GC03M025598	0.991214	https://www.genecards.org/cgi-bin/carddisp.pl?gene=TOP2B
LEFTY1	Left-Right Determination Factor 1	Protein Coding	O75610	46	GC01M225891	0.991214	https://www.genecards.org/cgi-bin/carddisp.pl?gene=LEFTY1
MIR5090	MicroRNA 5090	RNA Gene		14	GC07P102465	0.990495	https://www.genecards.org/cgi-bin/carddisp.pl?gene=MIR5090

PRDM1	PR/SET Domain 1	Protein Coding	O75626	48	GC06P112962	0.990027	https://www.genecards.org/cgi-bin/carddisp.pl?gene=PRDM1
CHRNA6	Cholinergic Receptor Nicotinic Alpha 6 Subunit	Protein Coding	Q15825	47	GC08M042752	0.988805	https://www.genecards.org/cgi-bin/carddisp.pl?gene=CHRNA6
MIR612	MicroRNA 612	RNA Gene		18	GC11P079853	0.988668	https://www.genecards.org/cgi-bin/carddisp.pl?gene=MIR612
NPVF	Neuropeptide VF Precursor	Protein Coding	Q9HCQ7	37	GC07M025224	0.985968	https://www.genecards.org/cgi-bin/carddisp.pl?gene=NPVF
ZBTB18	Zinc Finger And BTB Domain Containing 18	Protein Coding	Q99592	45	GC01P244048	0.982269	https://www.genecards.org/cgi-bin/carddisp.pl?gene=ZBTB18
TJP1	Tight Junction Protein 1	Protein Coding	Q07157	48	GC15M029699	0.981033	https://www.genecards.org/cgi-bin/carddisp.pl?gene=TJP1
TMSB4X	Thymosin Beta 4 X-Linked	Protein Coding	P62328	44	GC0XP012975	0.980635	https://www.genecards.org/cgi-bin/carddisp.pl?gene=TMSB4X
BCL2L14	BCL2 Like 14	Protein Coding	Q9BZR8	43	GC12P012049	0.980635	https://www.genecards.org/cgi-bin/carddisp.pl?gene=BCL2L14
CD34	CD34 Molecule	Protein Coding	P28906	50	GC01M207880	0.980012	https://www.genecards.org/cgi-bin/carddisp.pl?gene=CD34
WDR25	WD Repeat Domain 25	Protein Coding	Q64LD2	37	GC14P112876	0.97981	https://www.genecards.org/cgi-bin/carddisp.pl?gene=WDR25
HAL	Histidine Ammonia-Lyase	Protein Coding	P42357	47	GC12M095972	0.978618	https://www.genecards.org/cgi-bin/carddisp.pl?gene=HAL
OSBP	Oxysterol Binding Protein	Protein Coding	P22059	45	GC11M113262	0.978618	https://www.genecards.org/cgi-bin/carddisp.pl?gene=OSBP

MSI2	Musashi RNA Binding Protein 2	Protein Coding	Q96DH6	44	GC17P057255	0.978618	https://www.genecards.org/cgi-bin/carddisp.pl?gene=MSI2
LEF1-AS1	LEF1 Antisense RNA 1	RNA Gene		20	GC04P108167	0.978618	https://www.genecards.org/cgi-bin/carddisp.pl?gene=LEF1-AS1
MIR635	MicroRNA 635	RNA Gene		17	GC17M068424	0.978618	https://www.genecards.org/cgi-bin/carddisp.pl?gene=MIR635
PIKFYVE	Phosphoinositide Kinase, FYVE-Type Zinc Finger Containing	Protein Coding	Q9Y2I7	53	GC02P208266	0.977871	https://www.genecards.org/cgi-bin/carddisp.pl?gene=PIKFYVE
SYNJ1	Synaptojanin 1	Protein Coding	O43426	50	GC21M032628	0.977871	https://www.genecards.org/cgi-bin/carddisp.pl?gene=SYNJ1
ARHGAP31	Rho GTPase Activating Protein 31	Protein Coding	Q2M1Z3	45	GC03P119294	0.977871	https://www.genecards.org/cgi-bin/carddisp.pl?gene=ARHGAP31
HYDIN	HYDIN Axonemal Central Pair Apparatus Protein	Protein Coding	Q4G0P3	42	GC16M072262	0.977871	https://www.genecards.org/cgi-bin/carddisp.pl?gene=HYDIN
KCNJ16	Potassium Inwardly Rectifying Channel Subfamily J Member 16	Protein Coding	Q9NPI9	42	GC17P070053	0.977871	https://www.genecards.org/cgi-bin/carddisp.pl?gene=KCNJ16
ZNF23	Zinc Finger Protein 23	Protein Coding	P17027	42	GC16M072275	0.977871	https://www.genecards.org/cgi-bin/carddisp.pl?gene=ZNF23
CFAP47	Cilia And Flagella Associated Protein 47	Protein Coding	Q6ZTR5	34	GC0XP035919	0.977871	https://www.genecards.org/cgi-bin/carddisp.pl?gene=CFAP47
LINC01619	Long Intergenic Non-Protein Coding RNA 1619	RNA Gene	G3V211	19	GC12M091993	0.977799	https://www.genecards.org/cgi-bin/carddisp.pl?gene=LINC01619
UBA5	Ubiquitin Like Modifier Activating Enzyme 5	Protein Coding	Q9GZZ9	45	GC03P132654	0.976222	https://www.genecards.org/cgi-bin/carddisp.pl?gene=UBA5

GABRR2	Gamma-Aminobutyric Acid Type A Receptor Subunit Rho2	Protein Coding	P28476	44	GC06M089257	0.975616	https://www.genecards.org/cgi-bin/carddisp.pl?gene=GABRR2
FANCM	FA Complementation Group M	Protein Coding	Q8IYD8	47	GC14P045135	0.975424	https://www.genecards.org/cgi-bin/carddisp.pl?gene=FANCM
SNRNP70	Small Nuclear Ribonucleoprotein U1 Subunit 70	Protein Coding	P08621	44	GC19P049085	0.974822	https://www.genecards.org/cgi-bin/carddisp.pl?gene=SNRNP70
HSPA1L	Heat Shock Protein Family A (Hsp70) Member 1 Like	Protein Coding	P34931	48	GC06M031809	0.973261	https://www.genecards.org/cgi-bin/carddisp.pl?gene=HSPA1L
RBX1	Ring-Box 1	Protein Coding	P62877	47	GC22P040951	0.970393	https://www.genecards.org/cgi-bin/carddisp.pl?gene=RBX1
MIR129-1	MicroRNA 129-1	RNA Gene		18	GC07P128207	0.965682	https://www.genecards.org/cgi-bin/carddisp.pl?gene=MIR129-1
ADTRP	Androgen Dependent TFPI Regulating Protein	Protein Coding	Q96IZ2	35	GC06M011712	0.964164	https://www.genecards.org/cgi-bin/carddisp.pl?gene=ADTRP
LOC114827851	VISTA Enhancer Hs2155	Functional Element		3	GC14P040656	0.964046	https://www.genecards.org/cgi-bin/carddisp.pl?gene=LOC114827851
MIR337	MicroRNA 337	RNA Gene		21	GC14P113591	0.963237	https://www.genecards.org/cgi-bin/carddisp.pl?gene=MIR337
IL25	Interleukin 25	Protein Coding	Q9H293	42	GC14P040152	0.962432	https://www.genecards.org/cgi-bin/carddisp.pl?gene=IL25
KAT6B	Lysine Acetyltransferase 6B	Protein Coding	Q8WYB5	48	GC10P074885	0.960525	https://www.genecards.org/cgi-bin/carddisp.pl?gene=KAT6B
U2AF1	U2 Small Nuclear RNA Auxiliary Factor 1	Protein Coding	Q01081	47	GC21M043092	0.960415	https://www.genecards.org/cgi-bin/carddisp.pl?gene=U2AF1

SLC30A10	Solute Carrier Family 30 Member 10	Protein Coding	Q6XR72	46	GC01M219685	0.960415	https://www.genecards.org/cgi-bin/carddisp.pl?gene=SLC30A10
HEATR5A	HEAT Repeat Containing 5A	Protein Coding	Q86XA9	35	GC14M031291	0.960415	https://www.genecards.org/cgi-bin/carddisp.pl?gene=HEATR5A
HNRNPDL	Heterogeneous Nuclear Ribonucleoprotein D Like	Protein Coding	O14979	47	GC04M082422	0.959732	https://www.genecards.org/cgi-bin/carddisp.pl?gene=HNRNPDL
ANK3	Ankyrin 3	Protein Coding	Q12955	46	GC10M060026	0.959531	https://www.genecards.org/cgi-bin/carddisp.pl?gene=ANK3
IL19	Interleukin 19	Protein Coding	Q9UHD0	45	GC01P206770	0.957567	https://www.genecards.org/cgi-bin/carddisp.pl?gene=IL19
ELOVL4	ELOVL Fatty Acid Elongase 4	Protein Coding	Q9GZR5	49	GC06M079914	0.956784	https://www.genecards.org/cgi-bin/carddisp.pl?gene=ELOVL4
STAT6	Signal Transducer And Activator Of Transcription 6	Protein Coding	P42226	54	GC12M057095	0.955279	https://www.genecards.org/cgi-bin/carddisp.pl?gene=STAT6
RASA1	RAS P21 Protein Activator 1	Protein Coding	P20936	51	GC05P087267	0.95482	https://www.genecards.org/cgi-bin/carddisp.pl?gene=RASA1
ASPA	Aspartoacylase	Protein Coding	P45381	47	GC17P003472	0.953573	https://www.genecards.org/cgi-bin/carddisp.pl?gene=ASPA
RNA5-8SN1	RNA, 5.8S Ribosomal N1	RNA Gene		9	GC21P015146	0.95269	https://www.genecards.org/cgi-bin/carddisp.pl?gene=RNA5-8SN1
RNA5-8SN2	RNA, 5.8S Ribosomal N2	RNA Gene		9	GC21P015013	0.95269	https://www.genecards.org/cgi-bin/carddisp.pl?gene=RNA5-8SN2
RNA5-8SN3	RNA, 5.8S Ribosomal N3	RNA Gene		9	GC21P015120	0.95269	https://www.genecards.org/cgi-bin/carddisp.pl?gene=RNA5-8SN3

RNA5-8SP10	RNA, 5.8S Ribosomal Pseudogene 10	Pseudogene		9	GC21P015831	0.95269	https://www.genecards.org/cgi-bin/carddisp.pl?gene=RNA5-8SP10
LOC124907114	5.8S Ribosomal RNA	RNA Gene		7	GC21P015449	0.95269	https://www.genecards.org/cgi-bin/carddisp.pl?gene=LOC124907114
LOC124907115	5.8S Ribosomal RNA	RNA Gene		7	GC21P015450	0.95269	https://www.genecards.org/cgi-bin/carddisp.pl?gene=LOC124907115
LOC124907116	5.8S Ribosomal RNA	RNA Gene		7	GC21P015452	0.95269	https://www.genecards.org/cgi-bin/carddisp.pl?gene=LOC124907116
LOC124907117	5.8S Ribosomal RNA	RNA Gene		7	GC21P015453	0.95269	https://www.genecards.org/cgi-bin/carddisp.pl?gene=LOC124907117
LOC124907118	5.8S Ribosomal RNA	RNA Gene		7	GC21P015456	0.95269	https://www.genecards.org/cgi-bin/carddisp.pl?gene=LOC124907118
LOC124907119	5.8S Ribosomal RNA	RNA Gene		7	GC21P015457	0.95269	https://www.genecards.org/cgi-bin/carddisp.pl?gene=LOC124907119
LOC124907120	5.8S Ribosomal RNA	RNA Gene		7	GC21P015458	0.95269	https://www.genecards.org/cgi-bin/carddisp.pl?gene=LOC124907120
LOC124907121	5.8S Ribosomal RNA	RNA Gene		7	GC21P015459	0.95269	https://www.genecards.org/cgi-bin/carddisp.pl?gene=LOC124907121
LOC124907122	5.8S Ribosomal RNA	RNA Gene		7	GC21P015460	0.95269	https://www.genecards.org/cgi-bin/carddisp.pl?gene=LOC124907122
LOC124907123	5.8S Ribosomal RNA	RNA Gene		7	GC21P015461	0.95269	https://www.genecards.org/cgi-bin/carddisp.pl?gene=LOC124907123
LOC124907124	5.8S Ribosomal RNA	RNA Gene		7	GC21P015462	0.95269	https://www.genecards.org/cgi-bin/carddisp.pl?gene=LOC124907124

LOC124907125	5.8S Ribosomal RNA	RNA Gene		7	GC21P015463	0.95269	https://www.genecards.org/cgi-bin/carddisp.pl?gene=LOC124907125
LOC124907126	5.8S Ribosomal RNA	RNA Gene		7	GC21P015464	0.95269	https://www.genecards.org/cgi-bin/carddisp.pl?gene=LOC124907126
LOC124907127	5.8S Ribosomal RNA	RNA Gene		7	GC21P015465	0.95269	https://www.genecards.org/cgi-bin/carddisp.pl?gene=LOC124907127
LOC124907128	5.8S Ribosomal RNA	RNA Gene		7	GC21P015466	0.95269	https://www.genecards.org/cgi-bin/carddisp.pl?gene=LOC124907128
LOC124907129	5.8S Ribosomal RNA	RNA Gene		7	GC21P015467	0.95269	https://www.genecards.org/cgi-bin/carddisp.pl?gene=LOC124907129
LOC124907130	5.8S Ribosomal RNA	RNA Gene		7	GC21P015468	0.95269	https://www.genecards.org/cgi-bin/carddisp.pl?gene=LOC124907130
LOC124907131	5.8S Ribosomal RNA	RNA Gene		7	GC21P015469	0.95269	https://www.genecards.org/cgi-bin/carddisp.pl?gene=LOC124907131
LOC124907132	5.8S Ribosomal RNA	RNA Gene		7	GC21P015470	0.95269	https://www.genecards.org/cgi-bin/carddisp.pl?gene=LOC124907132
LOC124907133	5.8S Ribosomal RNA	RNA Gene		7	GC21P015471	0.95269	https://www.genecards.org/cgi-bin/carddisp.pl?gene=LOC124907133
LOC124907134	5.8S Ribosomal RNA	RNA Gene		7	GC21P015472	0.95269	https://www.genecards.org/cgi-bin/carddisp.pl?gene=LOC124907134
LOC124907135	5.8S Ribosomal RNA	RNA Gene		7	GC21P015473	0.95269	https://www.genecards.org/cgi-bin/carddisp.pl?gene=LOC124907135
LOC124907136	5.8S Ribosomal RNA	RNA Gene		7	GC21P015474	0.95269	https://www.genecards.org/cgi-bin/carddisp.pl?gene=LOC124907136

LOC124907137	5.8S Ribosomal RNA	RNA Gene		7	GC21P015475	0.95269	https://www.genecards.org/cgi-bin/carddisp.pl?gene=LOC124907137
LOC124907138	5.8S Ribosomal RNA	RNA Gene		7	GC21P015476	0.95269	https://www.genecards.org/cgi-bin/carddisp.pl?gene=LOC124907138
LOC124907139	5.8S Ribosomal RNA	RNA Gene		7	GC21P015477	0.95269	https://www.genecards.org/cgi-bin/carddisp.pl?gene=LOC124907139
LOC124907140	5.8S Ribosomal RNA	RNA Gene		7	GC21P015478	0.95269	https://www.genecards.org/cgi-bin/carddisp.pl?gene=LOC124907140
LOC124907141	5.8S Ribosomal RNA	RNA Gene		7	GC21P015479	0.95269	https://www.genecards.org/cgi-bin/carddisp.pl?gene=LOC124907141
LOC124907142	5.8S Ribosomal RNA	RNA Gene		7	GC21P015480	0.95269	https://www.genecards.org/cgi-bin/carddisp.pl?gene=LOC124907142
LOC124907143	5.8S Ribosomal RNA	RNA Gene		7	GC21P015481	0.95269	https://www.genecards.org/cgi-bin/carddisp.pl?gene=LOC124907143
LOC124907144	5.8S Ribosomal RNA	RNA Gene		7	GC21P015482	0.95269	https://www.genecards.org/cgi-bin/carddisp.pl?gene=LOC124907144
LOC124907145	5.8S Ribosomal RNA	RNA Gene		7	GC21P015483	0.95269	https://www.genecards.org/cgi-bin/carddisp.pl?gene=LOC124907145
LOC124907146	5.8S Ribosomal RNA	RNA Gene		7	GC21P015484	0.95269	https://www.genecards.org/cgi-bin/carddisp.pl?gene=LOC124907146
LOC124907147	5.8S Ribosomal RNA	RNA Gene		7	GC21P015485	0.95269	https://www.genecards.org/cgi-bin/carddisp.pl?gene=LOC124907147
LOC124907148	5.8S Ribosomal RNA	RNA Gene		7	GC21P015486	0.95269	https://www.genecards.org/cgi-bin/carddisp.pl?gene=LOC124907148

LOC124907149	5.8S Ribosomal RNA	RNA Gene		7	GC21P015487	0.95269	https://www.genecards.org/cgi-bin/carddisp.pl?gene=LOC124907149
LOC124907150	5.8S Ribosomal RNA	RNA Gene		7	GC21P015488	0.95269	https://www.genecards.org/cgi-bin/carddisp.pl?gene=LOC124907150
LOC124907151	5.8S Ribosomal RNA	RNA Gene		7	GC21P015489	0.95269	https://www.genecards.org/cgi-bin/carddisp.pl?gene=LOC124907151
LOC124907152	5.8S Ribosomal RNA	RNA Gene		7	GC21P015490	0.95269	https://www.genecards.org/cgi-bin/carddisp.pl?gene=LOC124907152
LOC124907153	5.8S Ribosomal RNA	RNA Gene		7	GC21P015491	0.95269	https://www.genecards.org/cgi-bin/carddisp.pl?gene=LOC124907153
LOC124907154	5.8S Ribosomal RNA	RNA Gene		7	GC21P015493	0.95269	https://www.genecards.org/cgi-bin/carddisp.pl?gene=LOC124907154
LOC124907155	5.8S Ribosomal RNA	RNA Gene		7	GC21P015494	0.95269	https://www.genecards.org/cgi-bin/carddisp.pl?gene=LOC124907155
LOC124907160	5.8S Ribosomal RNA	RNA Gene		7	GC21P015495	0.95269	https://www.genecards.org/cgi-bin/carddisp.pl?gene=LOC124907160
LOC124907172	5.8S Ribosomal RNA	RNA Gene		7	GC21P015496	0.95269	https://www.genecards.org/cgi-bin/carddisp.pl?gene=LOC124907172
LOC124907183	5.8S Ribosomal RNA	RNA Gene		7	GC21P015497	0.95269	https://www.genecards.org/cgi-bin/carddisp.pl?gene=LOC124907183
LOC124907194	5.8S Ribosomal RNA	RNA Gene		7	GC21P015498	0.95269	https://www.genecards.org/cgi-bin/carddisp.pl?gene=LOC124907194
LOC124907205	5.8S Ribosomal RNA	RNA Gene		7	GC21P015499	0.95269	https://www.genecards.org/cgi-bin/carddisp.pl?gene=LOC124907205

LOC124907216	5.8S Ribosomal RNA	RNA Gene		7	GC21P015500	0.95269	https://www.genecards.org/cgi-bin/carddisp.pl?gene=LOC124907216
LOC124907227	5.8S Ribosomal RNA	RNA Gene		7	GC21P015501	0.95269	https://www.genecards.org/cgi-bin/carddisp.pl?gene=LOC124907227
LOC124907238	5.8S Ribosomal RNA	RNA Gene		7	GC21P015502	0.95269	https://www.genecards.org/cgi-bin/carddisp.pl?gene=LOC124907238
LOC124907248	5.8S Ribosomal RNA	RNA Gene		7	GC21P015503	0.95269	https://www.genecards.org/cgi-bin/carddisp.pl?gene=LOC124907248
LOC124907258	5.8S Ribosomal RNA	RNA Gene		7	GC21P015504	0.95269	https://www.genecards.org/cgi-bin/carddisp.pl?gene=LOC124907258
LOC124907269	5.8S Ribosomal RNA	RNA Gene		7	GC21P015505	0.95269	https://www.genecards.org/cgi-bin/carddisp.pl?gene=LOC124907269
LOC124907280	5.8S Ribosomal RNA	RNA Gene		7	GC21P015506	0.95269	https://www.genecards.org/cgi-bin/carddisp.pl?gene=LOC124907280
LOC124907291	5.8S Ribosomal RNA	RNA Gene		7	GC21P015507	0.95269	https://www.genecards.org/cgi-bin/carddisp.pl?gene=LOC124907291
LOC124907302	5.8S Ribosomal RNA	RNA Gene		7	GC21P015508	0.95269	https://www.genecards.org/cgi-bin/carddisp.pl?gene=LOC124907302
LOC124907313	5.8S Ribosomal RNA	RNA Gene		7	GC21P015509	0.95269	https://www.genecards.org/cgi-bin/carddisp.pl?gene=LOC124907313
LOC124907324	5.8S Ribosomal RNA	RNA Gene		7	GC21P015511	0.95269	https://www.genecards.org/cgi-bin/carddisp.pl?gene=LOC124907324
LOC124907327	5.8S Ribosomal RNA	RNA Gene		7	GC21P015512	0.95269	https://www.genecards.org/cgi-bin/carddisp.pl?gene=LOC124907327

LOC124907328	5.8S Ribosomal RNA	RNA Gene		7	GC21P015513	0.95269	https://www.genecards.org/cgi-bin/carddisp.pl?gene=LOC124907328
LOC124907329	5.8S Ribosomal RNA	RNA Gene		7	GC21P015514	0.95269	https://www.genecards.org/cgi-bin/carddisp.pl?gene=LOC124907329
LOC124907330	5.8S Ribosomal RNA	RNA Gene		7	GC21P015516	0.95269	https://www.genecards.org/cgi-bin/carddisp.pl?gene=LOC124907330
LOC124907331	5.8S Ribosomal RNA	RNA Gene		7	GC21P015517	0.95269	https://www.genecards.org/cgi-bin/carddisp.pl?gene=LOC124907331
LOC124907332	5.8S Ribosomal RNA	RNA Gene		7	GC21P015518	0.95269	https://www.genecards.org/cgi-bin/carddisp.pl?gene=LOC124907332
LOC124907333	5.8S Ribosomal RNA	RNA Gene		7	GC21P015519	0.95269	https://www.genecards.org/cgi-bin/carddisp.pl?gene=LOC124907333
LOC124907334	5.8S Ribosomal RNA	RNA Gene		7	GC21P015520	0.95269	https://www.genecards.org/cgi-bin/carddisp.pl?gene=LOC124907334
LOC124907335	5.8S Ribosomal RNA	RNA Gene		7	GC21P015521	0.95269	https://www.genecards.org/cgi-bin/carddisp.pl?gene=LOC124907335
LOC124907336	5.8S Ribosomal RNA	RNA Gene		7	GC21P015522	0.95269	https://www.genecards.org/cgi-bin/carddisp.pl?gene=LOC124907336
LOC124907337	5.8S Ribosomal RNA	RNA Gene		7	GC21P015523	0.95269	https://www.genecards.org/cgi-bin/carddisp.pl?gene=LOC124907337
LOC124907338	5.8S Ribosomal RNA	RNA Gene		7	GC21P015524	0.95269	https://www.genecards.org/cgi-bin/carddisp.pl?gene=LOC124907338
LOC124907339	5.8S Ribosomal RNA	RNA Gene		7	GC21P015525	0.95269	https://www.genecards.org/cgi-bin/carddisp.pl?gene=LOC124907339

LOC124907340	5.8S Ribosomal RNA	RNA Gene		7	GC21P015526	0.95269	https://www.genecards.org/cgi-bin/carddisp.pl?gene=LOC124907340
LOC124907341	5.8S Ribosomal RNA	RNA Gene		7	GC21P015527	0.95269	https://www.genecards.org/cgi-bin/carddisp.pl?gene=LOC124907341
LOC124907342	5.8S Ribosomal RNA	RNA Gene		7	GC21P015528	0.95269	https://www.genecards.org/cgi-bin/carddisp.pl?gene=LOC124907342
LOC124907343	5.8S Ribosomal RNA	RNA Gene		7	GC21P015529	0.95269	https://www.genecards.org/cgi-bin/carddisp.pl?gene=LOC124907343
LOC124907439	5.8S Ribosomal RNA	RNA Gene		7	GC21P015530	0.95269	https://www.genecards.org/cgi-bin/carddisp.pl?gene=LOC124907439
LOC124907440	5.8S Ribosomal RNA	RNA Gene		7	GC21P015531	0.95269	https://www.genecards.org/cgi-bin/carddisp.pl?gene=LOC124907440
LOC124907450	5.8S Ribosomal RNA	RNA Gene		7	GC21P015532	0.95269	https://www.genecards.org/cgi-bin/carddisp.pl?gene=LOC124907450
LOC124907458	5.8S Ribosomal RNA	RNA Gene		7	GC21P015533	0.95269	https://www.genecards.org/cgi-bin/carddisp.pl?gene=LOC124907458
LOC124907467	5.8S Ribosomal RNA	RNA Gene		7	GC21P015534	0.95269	https://www.genecards.org/cgi-bin/carddisp.pl?gene=LOC124907467
LOC124907475	5.8S Ribosomal RNA	RNA Gene		7	GC21P015535	0.95269	https://www.genecards.org/cgi-bin/carddisp.pl?gene=LOC124907475
LOC124907476	5.8S Ribosomal RNA	RNA Gene		7	GC21P015536	0.95269	https://www.genecards.org/cgi-bin/carddisp.pl?gene=LOC124907476
LOC124907477	5.8S Ribosomal RNA	RNA Gene		7	GC21P015537	0.95269	https://www.genecards.org/cgi-bin/carddisp.pl?gene=LOC124907477

LOC124907478	5.8S Ribosomal RNA	RNA Gene		7	GC21P015538	0.95269	https://www.genecards.org/cgi-bin/carddisp.pl?gene=LOC124907478
LOC124907479	5.8S Ribosomal RNA	RNA Gene		7	GC21P015539	0.95269	https://www.genecards.org/cgi-bin/carddisp.pl?gene=LOC124907479
LOC124907480	5.8S Ribosomal RNA	RNA Gene		7	GC21P015540	0.95269	https://www.genecards.org/cgi-bin/carddisp.pl?gene=LOC124907480
LOC124907481	5.8S Ribosomal RNA	RNA Gene		7	GC21P015541	0.95269	https://www.genecards.org/cgi-bin/carddisp.pl?gene=LOC124907481
LOC124907482	5.8S Ribosomal RNA	RNA Gene		7	GC21P015542	0.95269	https://www.genecards.org/cgi-bin/carddisp.pl?gene=LOC124907482
LOC124907483	5.8S Ribosomal RNA	RNA Gene		7	GC21P015544	0.95269	https://www.genecards.org/cgi-bin/carddisp.pl?gene=LOC124907483
LOC124907484	5.8S Ribosomal RNA	RNA Gene		7	GC21P015545	0.95269	https://www.genecards.org/cgi-bin/carddisp.pl?gene=LOC124907484
LOC124907572	5.8S Ribosomal RNA	RNA Gene		7	GC21P015546	0.95269	https://www.genecards.org/cgi-bin/carddisp.pl?gene=LOC124907572
LOC124907573	5.8S Ribosomal RNA	RNA Gene		7	GC21P015547	0.95269	https://www.genecards.org/cgi-bin/carddisp.pl?gene=LOC124907573
LOC124907574	5.8S Ribosomal RNA	RNA Gene		7	GC21P015548	0.95269	https://www.genecards.org/cgi-bin/carddisp.pl?gene=LOC124907574
LOC124907575	5.8S Ribosomal RNA	RNA Gene		7	GC21P015550	0.95269	https://www.genecards.org/cgi-bin/carddisp.pl?gene=LOC124907575
LOC124907576	5.8S Ribosomal RNA	RNA Gene		7	GC21P015551	0.95269	https://www.genecards.org/cgi-bin/carddisp.pl?gene=LOC124907576

LOC124907577	5.8S Ribosomal RNA	RNA Gene		7	GC21P015553	0.95269	https://www.genecards.org/cgi-bin/carddisp.pl?gene=LOC124907577
LOC124907578	5.8S Ribosomal RNA	RNA Gene		7	GC21P015554	0.95269	https://www.genecards.org/cgi-bin/carddisp.pl?gene=LOC124907578
LOC124907579	5.8S Ribosomal RNA	RNA Gene		7	GC21P015555	0.95269	https://www.genecards.org/cgi-bin/carddisp.pl?gene=LOC124907579
LOC124907580	5.8S Ribosomal RNA	RNA Gene		7	GC21P015556	0.95269	https://www.genecards.org/cgi-bin/carddisp.pl?gene=LOC124907580
LOC124907581	5.8S Ribosomal RNA	RNA Gene		7	GC21P015557	0.95269	https://www.genecards.org/cgi-bin/carddisp.pl?gene=LOC124907581
LOC124907584	5.8S Ribosomal RNA	RNA Gene		7	GC21P015558	0.95269	https://www.genecards.org/cgi-bin/carddisp.pl?gene=LOC124907584
LOC124907585	5.8S Ribosomal RNA	RNA Gene		7	GC21P015559	0.95269	https://www.genecards.org/cgi-bin/carddisp.pl?gene=LOC124907585
LOC124907586	5.8S Ribosomal RNA	RNA Gene		7	GC21P015560	0.95269	https://www.genecards.org/cgi-bin/carddisp.pl?gene=LOC124907586
LOC124907587	5.8S Ribosomal RNA	RNA Gene		7	GC21P015561	0.95269	https://www.genecards.org/cgi-bin/carddisp.pl?gene=LOC124907587
LOC124907588	5.8S Ribosomal RNA	RNA Gene		7	GC21P015562	0.95269	https://www.genecards.org/cgi-bin/carddisp.pl?gene=LOC124907588
LOC124907589	5.8S Ribosomal RNA	RNA Gene		7	GC21P015565	0.95269	https://www.genecards.org/cgi-bin/carddisp.pl?gene=LOC124907589
LOC124907590	5.8S Ribosomal RNA	RNA Gene		7	GC21P015567	0.95269	https://www.genecards.org/cgi-bin/carddisp.pl?gene=LOC124907590

LOC124907591	5.8S Ribosomal RNA	RNA Gene		7	GC21P015568	0.95269	https://www.genecards.org/cgi-bin/carddisp.pl?gene=LOC124907591
LOC124907592	5.8S Ribosomal RNA	RNA Gene		7	GC21P015570	0.95269	https://www.genecards.org/cgi-bin/carddisp.pl?gene=LOC124907592
LOC124907593	5.8S Ribosomal RNA	RNA Gene		7	GC21P015571	0.95269	https://www.genecards.org/cgi-bin/carddisp.pl?gene=LOC124907593
LOC124907594	5.8S Ribosomal RNA	RNA Gene		7	GC21P015572	0.95269	https://www.genecards.org/cgi-bin/carddisp.pl?gene=LOC124907594
LOC124907595	5.8S Ribosomal RNA	RNA Gene		7	GC21P015573	0.95269	https://www.genecards.org/cgi-bin/carddisp.pl?gene=LOC124907595
LOC124907596	5.8S Ribosomal RNA	RNA Gene		7	GC21P015574	0.95269	https://www.genecards.org/cgi-bin/carddisp.pl?gene=LOC124907596
LOC124907597	5.8S Ribosomal RNA	RNA Gene		7	GC21P015575	0.95269	https://www.genecards.org/cgi-bin/carddisp.pl?gene=LOC124907597
LOC124907598	5.8S Ribosomal RNA	RNA Gene		7	GC21P015576	0.95269	https://www.genecards.org/cgi-bin/carddisp.pl?gene=LOC124907598
LOC124907600	5.8S Ribosomal RNA	RNA Gene		7	GC21P015577	0.95269	https://www.genecards.org/cgi-bin/carddisp.pl?gene=LOC124907600
LOC124907611	5.8S Ribosomal RNA	RNA Gene		7	GC21P015578	0.95269	https://www.genecards.org/cgi-bin/carddisp.pl?gene=LOC124907611
LOC124907622	5.8S Ribosomal RNA	RNA Gene		7	GC21P015579	0.95269	https://www.genecards.org/cgi-bin/carddisp.pl?gene=LOC124907622
LOC124907633	5.8S Ribosomal RNA	RNA Gene		7	GC21P015580	0.95269	https://www.genecards.org/cgi-bin/carddisp.pl?gene=LOC124907633

LOC124907644	5.8S Ribosomal RNA	RNA Gene		7	GC21P015581	0.95269	https://www.genecards.org/cgi-bin/carddisp.pl?gene=LOC124907644
LOC124907655	5.8S Ribosomal RNA	RNA Gene		7	GC21P015582	0.95269	https://www.genecards.org/cgi-bin/carddisp.pl?gene=LOC124907655
LOC124907662	5.8S Ribosomal RNA	RNA Gene		7	GC21P015583	0.95269	https://www.genecards.org/cgi-bin/carddisp.pl?gene=LOC124907662
LOC124907673	5.8S Ribosomal RNA	RNA Gene		7	GC21P015584	0.95269	https://www.genecards.org/cgi-bin/carddisp.pl?gene=LOC124907673
LOC124907684	5.8S Ribosomal RNA	RNA Gene		7	GC21P015585	0.95269	https://www.genecards.org/cgi-bin/carddisp.pl?gene=LOC124907684
LOC124907694	5.8S Ribosomal RNA	RNA Gene		7	GC21P015586	0.95269	https://www.genecards.org/cgi-bin/carddisp.pl?gene=LOC124907694
LOC124907705	5.8S Ribosomal RNA	RNA Gene		7	GC21P015587	0.95269	https://www.genecards.org/cgi-bin/carddisp.pl?gene=LOC124907705
LOC124907709	5.8S Ribosomal RNA	RNA Gene		7	GC21P015589	0.95269	https://www.genecards.org/cgi-bin/carddisp.pl?gene=LOC124907709
LOC124907710	5.8S Ribosomal RNA	RNA Gene		7	GC21P015591	0.95269	https://www.genecards.org/cgi-bin/carddisp.pl?gene=LOC124907710
LOC124907711	5.8S Ribosomal RNA	RNA Gene		7	GC21P015592	0.95269	https://www.genecards.org/cgi-bin/carddisp.pl?gene=LOC124907711
LOC124907712	5.8S Ribosomal RNA	RNA Gene		7	GC21P015593	0.95269	https://www.genecards.org/cgi-bin/carddisp.pl?gene=LOC124907712
LOC124907713	5.8S Ribosomal RNA	RNA Gene		7	GC21P015594	0.95269	https://www.genecards.org/cgi-bin/carddisp.pl?gene=LOC124907713

LOC124907714	5.8S Ribosomal RNA	RNA Gene		7	GC21P015595	0.95269	https://www.genecards.org/cgi-bin/carddisp.pl?gene=LOC124907714
LOC124907715	5.8S Ribosomal RNA	RNA Gene		7	GC21P015596	0.95269	https://www.genecards.org/cgi-bin/carddisp.pl?gene=LOC124907715
LOC124907716	5.8S Ribosomal RNA	RNA Gene		7	GC21P015597	0.95269	https://www.genecards.org/cgi-bin/carddisp.pl?gene=LOC124907716
LOC124907717	5.8S Ribosomal RNA	RNA Gene		7	GC21P015598	0.95269	https://www.genecards.org/cgi-bin/carddisp.pl?gene=LOC124907717
LOC124907718	5.8S Ribosomal RNA	RNA Gene		7	GC21P015599	0.95269	https://www.genecards.org/cgi-bin/carddisp.pl?gene=LOC124907718
LOC124907719	5.8S Ribosomal RNA	RNA Gene		7	GC21P015600	0.95269	https://www.genecards.org/cgi-bin/carddisp.pl?gene=LOC124907719
LOC124907720	5.8S Ribosomal RNA	RNA Gene		7	GC21P015601	0.95269	https://www.genecards.org/cgi-bin/carddisp.pl?gene=LOC124907720
LOC124907721	5.8S Ribosomal RNA	RNA Gene		7	GC21P015602	0.95269	https://www.genecards.org/cgi-bin/carddisp.pl?gene=LOC124907721
LOC124908237	5.8S Ribosomal RNA	RNA Gene		7	GC21P015603	0.95269	https://www.genecards.org/cgi-bin/carddisp.pl?gene=LOC124908237
LOC124908238	5.8S Ribosomal RNA	RNA Gene		7	GC21P015604	0.95269	https://www.genecards.org/cgi-bin/carddisp.pl?gene=LOC124908238
LOC124908239	5.8S Ribosomal RNA	RNA Gene		7	GC21P015605	0.95269	https://www.genecards.org/cgi-bin/carddisp.pl?gene=LOC124908239
LOC124908240	5.8S Ribosomal RNA	RNA Gene		7	GC21P015606	0.95269	https://www.genecards.org/cgi-bin/carddisp.pl?gene=LOC124908240

LOC124908241	5.8S Ribosomal RNA	RNA Gene		7	GC21P015607	0.95269	https://www.genecards.org/cgi-bin/carddisp.pl?gene=LOC124908241
LOC124908242	5.8S Ribosomal RNA	RNA Gene		7	GC21P015609	0.95269	https://www.genecards.org/cgi-bin/carddisp.pl?gene=LOC124908242
LOC124908243	5.8S Ribosomal RNA	RNA Gene		7	GC21P015610	0.95269	https://www.genecards.org/cgi-bin/carddisp.pl?gene=LOC124908243
LOC124908244	5.8S Ribosomal RNA	RNA Gene		7	GC21P015611	0.95269	https://www.genecards.org/cgi-bin/carddisp.pl?gene=LOC124908244
LOC124908245	5.8S Ribosomal RNA	RNA Gene		7	GC21P015612	0.95269	https://www.genecards.org/cgi-bin/carddisp.pl?gene=LOC124908245
LOC124908246	5.8S Ribosomal RNA	RNA Gene		7	GC21P015613	0.95269	https://www.genecards.org/cgi-bin/carddisp.pl?gene=LOC124908246
LOC124908247	5.8S Ribosomal RNA	RNA Gene		7	GC21P015620	0.95269	https://www.genecards.org/cgi-bin/carddisp.pl?gene=LOC124908247
LOC124908248	5.8S Ribosomal RNA	RNA Gene		7	GC21P015621	0.95269	https://www.genecards.org/cgi-bin/carddisp.pl?gene=LOC124908248
LOC124908249	5.8S Ribosomal RNA	RNA Gene		7	GC21P015623	0.95269	https://www.genecards.org/cgi-bin/carddisp.pl?gene=LOC124908249
LOC124908257	5.8S Ribosomal RNA	RNA Gene		7	GC21P015624	0.95269	https://www.genecards.org/cgi-bin/carddisp.pl?gene=LOC124908257
LOC124908268	5.8S Ribosomal RNA	RNA Gene		7	GC21P015625	0.95269	https://www.genecards.org/cgi-bin/carddisp.pl?gene=LOC124908268
LOC124908278	5.8S Ribosomal RNA	RNA Gene		7	GC21P015626	0.95269	https://www.genecards.org/cgi-bin/carddisp.pl?gene=LOC124908278

LOC124908289	5.8S Ribosomal RNA	RNA Gene		7	GC21P015627	0.95269	https://www.genecards.org/cgi-bin/carddisp.pl?gene=LOC124908289
LOC124908300	5.8S Ribosomal RNA	RNA Gene		7	GC21P015628	0.95269	https://www.genecards.org/cgi-bin/carddisp.pl?gene=LOC124908300
LOC124908310	5.8S Ribosomal RNA	RNA Gene		7	GC21P015629	0.95269	https://www.genecards.org/cgi-bin/carddisp.pl?gene=LOC124908310
LOC124908316	5.8S Ribosomal RNA	RNA Gene		7	GC21P015630	0.95269	https://www.genecards.org/cgi-bin/carddisp.pl?gene=LOC124908316
LOC124908327	5.8S Ribosomal RNA	RNA Gene		7	GC21P015631	0.95269	https://www.genecards.org/cgi-bin/carddisp.pl?gene=LOC124908327
LOC124908336	5.8S Ribosomal RNA	RNA Gene		7	GC21P015632	0.95269	https://www.genecards.org/cgi-bin/carddisp.pl?gene=LOC124908336
LOC124908347	5.8S Ribosomal RNA	RNA Gene		7	GC21P015633	0.95269	https://www.genecards.org/cgi-bin/carddisp.pl?gene=LOC124908347
LOC124908358	5.8S Ribosomal RNA	RNA Gene		7	GC21P015634	0.95269	https://www.genecards.org/cgi-bin/carddisp.pl?gene=LOC124908358
LOC124908368	5.8S Ribosomal RNA	RNA Gene		7	GC21P015635	0.95269	https://www.genecards.org/cgi-bin/carddisp.pl?gene=LOC124908368
LOC124908369	5.8S Ribosomal RNA	RNA Gene		7	GC21P015636	0.95269	https://www.genecards.org/cgi-bin/carddisp.pl?gene=LOC124908369
LOC124908370	5.8S Ribosomal RNA	RNA Gene		7	GC21P015637	0.95269	https://www.genecards.org/cgi-bin/carddisp.pl?gene=LOC124908370
LOC124908371	5.8S Ribosomal RNA	RNA Gene		7	GC21P015638	0.95269	https://www.genecards.org/cgi-bin/carddisp.pl?gene=LOC124908371

LOC124908372	5.8S Ribosomal RNA	RNA Gene		7	GC21P015639	0.95269	https://www.genecards.org/cgi-bin/carddisp.pl?gene=LOC124908372
LOC124908373	5.8S Ribosomal RNA	RNA Gene		7	GC21P015640	0.95269	https://www.genecards.org/cgi-bin/carddisp.pl?gene=LOC124908373
LOC124908374	5.8S Ribosomal RNA	RNA Gene		7	GC21P015641	0.95269	https://www.genecards.org/cgi-bin/carddisp.pl?gene=LOC124908374
LOC124908375	5.8S Ribosomal RNA	RNA Gene		7	GC21P015642	0.95269	https://www.genecards.org/cgi-bin/carddisp.pl?gene=LOC124908375
LOC124908376	5.8S Ribosomal RNA	RNA Gene		7	GC21P015643	0.95269	https://www.genecards.org/cgi-bin/carddisp.pl?gene=LOC124908376
LOC124908377	5.8S Ribosomal RNA	RNA Gene		7	GC21P015644	0.95269	https://www.genecards.org/cgi-bin/carddisp.pl?gene=LOC124908377
LOC124908378	5.8S Ribosomal RNA	RNA Gene		7	GC21P015645	0.95269	https://www.genecards.org/cgi-bin/carddisp.pl?gene=LOC124908378
LOC124908379	5.8S Ribosomal RNA	RNA Gene		7	GC21P015648	0.95269	https://www.genecards.org/cgi-bin/carddisp.pl?gene=LOC124908379
LOC124908380	5.8S Ribosomal RNA	RNA Gene		7	GC21P015649	0.95269	https://www.genecards.org/cgi-bin/carddisp.pl?gene=LOC124908380
LOC124908381	5.8S Ribosomal RNA	RNA Gene		7	GC21P015650	0.95269	https://www.genecards.org/cgi-bin/carddisp.pl?gene=LOC124908381
LOC124908382	5.8S Ribosomal RNA	RNA Gene		7	GC21P015651	0.95269	https://www.genecards.org/cgi-bin/carddisp.pl?gene=LOC124908382
LOC124908383	5.8S Ribosomal RNA	RNA Gene		7	GC21P015652	0.95269	https://www.genecards.org/cgi-bin/carddisp.pl?gene=LOC124908383

LOC124908384	5.8S Ribosomal RNA	RNA Gene		7	GC21P015653	0.95269	https://www.genecards.org/cgi-bin/carddisp.pl?gene=LOC124908384
LOC124908385	5.8S Ribosomal RNA	RNA Gene		7	GC21P015654	0.95269	https://www.genecards.org/cgi-bin/carddisp.pl?gene=LOC124908385
LOC124908386	5.8S Ribosomal RNA	RNA Gene		7	GC21P015655	0.95269	https://www.genecards.org/cgi-bin/carddisp.pl?gene=LOC124908386
LOC124908387	5.8S Ribosomal RNA	RNA Gene		7	GC21P015656	0.95269	https://www.genecards.org/cgi-bin/carddisp.pl?gene=LOC124908387
LOC124908388	5.8S Ribosomal RNA	RNA Gene		7	GC21P015657	0.95269	https://www.genecards.org/cgi-bin/carddisp.pl?gene=LOC124908388
LOC124908389	5.8S Ribosomal RNA	RNA Gene		7	GC21P015658	0.95269	https://www.genecards.org/cgi-bin/carddisp.pl?gene=LOC124908389
LOC124908390	5.8S Ribosomal RNA	RNA Gene		7	GC21P015659	0.95269	https://www.genecards.org/cgi-bin/carddisp.pl?gene=LOC124908390
LOC124908391	5.8S Ribosomal RNA	RNA Gene		7	GC21P015660	0.95269	https://www.genecards.org/cgi-bin/carddisp.pl?gene=LOC124908391
LOC124908392	5.8S Ribosomal RNA	RNA Gene		7	GC21P015661	0.95269	https://www.genecards.org/cgi-bin/carddisp.pl?gene=LOC124908392
LOC124908393	5.8S Ribosomal RNA	RNA Gene		7	GC21P015662	0.95269	https://www.genecards.org/cgi-bin/carddisp.pl?gene=LOC124908393
LOC124908474	5.8S Ribosomal RNA	RNA Gene		7	GC21P015663	0.95269	https://www.genecards.org/cgi-bin/carddisp.pl?gene=LOC124908474
LOC124908494	5.8S Ribosomal RNA	RNA Gene		7	GC21P015664	0.95269	https://www.genecards.org/cgi-bin/carddisp.pl?gene=LOC124908494

LOC124908504	5.8S Ribosomal RNA	RNA Gene		7	GC21P015665	0.95269	https://www.genecards.org/cgi-bin/carddisp.pl?gene=LOC124908504
LOC124908512	5.8S Ribosomal RNA	RNA Gene		7	GC21P015666	0.95269	https://www.genecards.org/cgi-bin/carddisp.pl?gene=LOC124908512
LOC124908513	5.8S Ribosomal RNA	RNA Gene		7	GC21P015667	0.95269	https://www.genecards.org/cgi-bin/carddisp.pl?gene=LOC124908513
LOC124908514	5.8S Ribosomal RNA	RNA Gene		7	GC21P015668	0.95269	https://www.genecards.org/cgi-bin/carddisp.pl?gene=LOC124908514
LOC124908515	5.8S Ribosomal RNA	RNA Gene		7	GC21P015669	0.95269	https://www.genecards.org/cgi-bin/carddisp.pl?gene=LOC124908515
LOC124908516	5.8S Ribosomal RNA	RNA Gene		7	GC21P015670	0.95269	https://www.genecards.org/cgi-bin/carddisp.pl?gene=LOC124908516
LOC124908517	5.8S Ribosomal RNA	RNA Gene		7	GC21P015672	0.95269	https://www.genecards.org/cgi-bin/carddisp.pl?gene=LOC124908517
LOC124908518	5.8S Ribosomal RNA	RNA Gene		7	GC21P015673	0.95269	https://www.genecards.org/cgi-bin/carddisp.pl?gene=LOC124908518
LOC124908519	5.8S Ribosomal RNA	RNA Gene		7	GC21P015674	0.95269	https://www.genecards.org/cgi-bin/carddisp.pl?gene=LOC124908519
LOC124908520	5.8S Ribosomal RNA	RNA Gene		7	GC21P015675	0.95269	https://www.genecards.org/cgi-bin/carddisp.pl?gene=LOC124908520
LOC124908521	5.8S Ribosomal RNA	RNA Gene		7	GC21P015676	0.95269	https://www.genecards.org/cgi-bin/carddisp.pl?gene=LOC124908521
LOC124908522	5.8S Ribosomal RNA	RNA Gene		7	GC21P015677	0.95269	https://www.genecards.org/cgi-bin/carddisp.pl?gene=LOC124908522

LOC124908523	5.8S Ribosomal RNA	RNA Gene		7	GC21P015678	0.95269	https://www.genecards.org/cgi-bin/carddisp.pl?gene=LOC124908523
LOC124908524	5.8S Ribosomal RNA	RNA Gene		7	GC21P015679	0.95269	https://www.genecards.org/cgi-bin/carddisp.pl?gene=LOC124908524
LOC124908525	5.8S Ribosomal RNA	RNA Gene		7	GC21P015680	0.95269	https://www.genecards.org/cgi-bin/carddisp.pl?gene=LOC124908525
LOC124908527	5.8S Ribosomal RNA	RNA Gene		7	GC21P015681	0.95269	https://www.genecards.org/cgi-bin/carddisp.pl?gene=LOC124908527
MAP3K3	Mitogen-Activated Protein Kinase Kinase Kinase 3	Protein Coding	Q99759	51	GC17P063622	0.951563	https://www.genecards.org/cgi-bin/carddisp.pl?gene=MAP3K3
TNFRSF4	TNF Receptor Superfamily Member 4	Protein Coding	P43489	48	GC01M001211	0.951563	https://www.genecards.org/cgi-bin/carddisp.pl?gene=TNFRSF4
CAND2	Cullin Associated And Neddylation Dissociated 2 (Putative)	Protein Coding	O75155	41	GC03P018407	0.951563	https://www.genecards.org/cgi-bin/carddisp.pl?gene=CAND2
MIR151B	MicroRNA 151b	RNA Gene		14	GC14M100109	0.951563	https://www.genecards.org/cgi-bin/carddisp.pl?gene=MIR151B
ST2	Suppression Of Tumorigenicity 2	Genetic Locus		7	GC11U990127	0.951563	https://www.genecards.org/cgi-bin/carddisp.pl?gene=ST2
MBD4	Methyl-CpG Binding Domain 4, DNA Glycosylase	Protein Coding	O95243	47	GC03M129430	0.950739	https://www.genecards.org/cgi-bin/carddisp.pl?gene=MBD4
H4C1	H4 Clustered Histone 1	Protein Coding	P62805	39	GC06P111761	0.950329	https://www.genecards.org/cgi-bin/carddisp.pl?gene=H4C1
LRPPRC	Leucine Rich Pentatricopeptide Repeat Containing	Protein Coding	P42704	47	GC02M043886	0.950303	https://www.genecards.org/cgi-bin/carddisp.pl?gene=LRPPRC

SLC25A46	Solute Carrier Family 25 Member 46	Protein Coding	Q96AG3	43	GC05P110738	0.950303	https://www.genecards.org/cgi-bin/carddisp.pl?gene=SLC25A46
SLIRP	SRA Stem-Loop Interacting RNA Binding Protein	Protein Coding	Q9GZT3	41	GC14P077708	0.950303	https://www.genecards.org/cgi-bin/carddisp.pl?gene=SLIRP
COG5	Component Of Oligomeric Golgi Complex 5	Protein Coding	Q9UP83	44	GC07M107201	0.948593	https://www.genecards.org/cgi-bin/carddisp.pl?gene=COG5
STAB1	Stabilin 1	Protein Coding	Q9NY15	45	GC03P052495	0.948479	https://www.genecards.org/cgi-bin/carddisp.pl?gene=STAB1
KYNU	Kynureninase	Protein Coding	Q16719	52	GC02P142877	0.947669	https://www.genecards.org/cgi-bin/carddisp.pl?gene=KYNU
COL24A1	Collagen Type XXIV Alpha 1 Chain	Protein Coding	Q17RW2	40	GC01M085729	0.946844	https://www.genecards.org/cgi-bin/carddisp.pl?gene=COL24A1
ASL	Argininosuccinate Lyase	Protein Coding	P04424	49	GC07P066075	0.945573	https://www.genecards.org/cgi-bin/carddisp.pl?gene=ASL
TRP-AGG2-5	TRNA-Pro (Anticodon AGG) 2-5	RNA Gene		13	GC14M025619	0.945109	https://www.genecards.org/cgi-bin/carddisp.pl?gene=TRP-AGG2-5
TTPA	Alpha Tocopherol Transfer Protein	Protein Coding	P49638	46	GC08M063048	0.94475	https://www.genecards.org/cgi-bin/carddisp.pl?gene=TTPA
SMARCA1	SWI/SNF Related, Matrix Associated, Actin Dependent Regulator Of Chromatin, Subfamily A, Member 1	Protein Coding	P28370	45	GC0XM129447	0.943256	https://www.genecards.org/cgi-bin/carddisp.pl?gene=SMARCA1
KLF2	KLF Transcription Factor 2	Protein Coding	Q9Y5W3	44	GC19P089073	0.941658	https://www.genecards.org/cgi-bin/carddisp.pl?gene=KLF2
TGM1	Transglutaminase 1	Protein Coding	P22735	50	GC14M024249	0.940418	https://www.genecards.org/cgi-bin/carddisp.pl?gene=TGM1

PHOX2B	Paired Like Homeobox 2B	Protein Coding	Q99453	47	GC04M041746	0.939966	https://www.genecards.org/cgi-bin/carddisp.pl?gene=PHOX2B
IFNAR1	Interferon Alpha And Beta Receptor Subunit 1	Protein Coding	P17181	53	GC21P033324	0.939044	https://www.genecards.org/cgi-bin/carddisp.pl?gene=IFNAR1
ADPRH	ADP-Ribosylarginine Hydrolase	Protein Coding	P54922	41	GC03P119579	0.931114	https://www.genecards.org/cgi-bin/carddisp.pl?gene=ADPRH
CCNL1	Cyclin L1	Protein Coding	Q9UK58	44	GC03M157146	0.930925	https://www.genecards.org/cgi-bin/carddisp.pl?gene=CCNL1
CHRNB2	Cholinergic Receptor Nicotinic Beta 2 Subunit	Protein Coding	P17787	50	GC01P157062	0.930456	https://www.genecards.org/cgi-bin/carddisp.pl?gene=CHRNB2
PLCB4	Phospholipase C Beta 4	Protein Coding	Q15147	49	GC20P009067	0.928223	https://www.genecards.org/cgi-bin/carddisp.pl?gene=PLCB4
PTMA	Prothymosin Alpha	Protein Coding	P06454	46	GC02P232105	0.927958	https://www.genecards.org/cgi-bin/carddisp.pl?gene=PTMA
PMM2	Phosphomannomutase 2	Protein Coding	O15305	51	GC16P008788	0.927919	https://www.genecards.org/cgi-bin/carddisp.pl?gene=PMM2
GSN	Gelsolin	Protein Coding	P06396	53	GC09P121201	0.927736	https://www.genecards.org/cgi-bin/carddisp.pl?gene=GSN
LDHB	Lactate Dehydrogenase B	Protein Coding	P07195	51	GC12M021635	0.926571	https://www.genecards.org/cgi-bin/carddisp.pl?gene=LDHB
SMC2	Structural Maintenance Of Chromosomes 2	Protein Coding	O95347	44	GC09P104088	0.925172	https://www.genecards.org/cgi-bin/carddisp.pl?gene=SMC2
MRTFA	Myocardin Related Transcription Factor A	Protein Coding	Q969V6	45	GC22M071257	0.922222	https://www.genecards.org/cgi-bin/carddisp.pl?gene=MRTFA

PRDX4	Peroxiredoxin 4	Protein Coding	Q13162	46	GC0XP023665	0.92132	https://www.genecards.org/cgi-bin/carddisp.pl?gene=PRDX4
MUC5AC	Mucin 5AC, Oligomeric Mucus/Gel-Forming	Protein Coding	P98088	43	GC11P004491	0.920608	https://www.genecards.org/cgi-bin/carddisp.pl?gene=MUC5AC
TAS2R43	Taste 2 Receptor Member 43	Protein Coding	P59537	37	GC12M011091	0.920608	https://www.genecards.org/cgi-bin/carddisp.pl?gene=TAS2R43
6NU2_B		RNA Gene		4	GC11M011317	0.920608	https://www.genecards.org/cgi-bin/carddisp.pl?gene=6NU2_B
ANMA	Anisomastia	Genetic Locus		2	GC16U990214	0.920608	https://www.genecards.org/cgi-bin/carddisp.pl?gene=ANMA
NFATC2	Nuclear Factor Of Activated T Cells 2	Protein Coding	Q13469	50	GC20M051386	0.91947	https://www.genecards.org/cgi-bin/carddisp.pl?gene=NFATC2
SACS	Sacsin Molecular Chaperone	Protein Coding	Q9NZJ4	41	GC13M023288	0.91947	https://www.genecards.org/cgi-bin/carddisp.pl?gene=SACS
NCOA6	Nuclear Receptor Coactivator 6	Protein Coding	Q14686	45	GC20M035441	0.919181	https://www.genecards.org/cgi-bin/carddisp.pl?gene=NCOA6
TDRD3	Tudor Domain Containing 3	Protein Coding	Q9H7E2	39	GC13P060396	0.918301	https://www.genecards.org/cgi-bin/carddisp.pl?gene=TDRD3
TDRD5	Tudor Domain Containing 5	Protein Coding	Q8NAT2	38	GC01P179591	0.918301	https://www.genecards.org/cgi-bin/carddisp.pl?gene=TDRD5
DSCAM-AS1	DSCAM Antisense RNA 1	RNA Gene		17	GC21P040383	0.918054	https://www.genecards.org/cgi-bin/carddisp.pl?gene=DSCAM-AS1
BRIP1	BRCA1 Interacting Helicase 1	Protein Coding	Q9BX63	53	GC17M061679	0.917445	https://www.genecards.org/cgi-bin/carddisp.pl?gene=BRIP1

CLTCL1	Clathrin Heavy Chain Like 1	Protein Coding	P53675	44	GC22M019692	0.917445	https://www.genecards.org/cgi-bin/carddisp.pl?gene=CLTCL1
IMPDH2	Inosine Monophosphate Dehydrogenase 2	Protein Coding	P12268	52	GC03M052654	0.915265	https://www.genecards.org/cgi-bin/carddisp.pl?gene=IMPDH2
FANCC	FA Complementation Group C	Protein Coding	Q00597	52	GC09M095099	0.914667	https://www.genecards.org/cgi-bin/carddisp.pl?gene=FANCC
TRIP4	Thyroid Hormone Receptor Interactor 4	Protein Coding	Q15650	45	GC15P137141	0.913233	https://www.genecards.org/cgi-bin/carddisp.pl?gene=TRIP4
KRT19	Keratin 19	Protein Coding	P08727	48	GC17M041523	0.912383	https://www.genecards.org/cgi-bin/carddisp.pl?gene=KRT19
LINC00958	Long Intergenic Non-Protein Coding RNA 958	RNA Gene		18	GC11M012877	0.909677	https://www.genecards.org/cgi-bin/carddisp.pl?gene=LINC00958
ATP2A1-AS1	ATP2A1 Antisense RNA 1	RNA Gene		17	GC16M041500	0.909677	https://www.genecards.org/cgi-bin/carddisp.pl?gene=ATP2A1-AS1
NTF3	Neurotrophin 3	Protein Coding	P20783	48	GC12P030424	0.909444	https://www.genecards.org/cgi-bin/carddisp.pl?gene=NTF3
HLCS	Holocarboxylase Synthetase	Protein Coding	P50747	47	GC21M036750	0.904619	https://www.genecards.org/cgi-bin/carddisp.pl?gene=HLCS
SCN2B	Sodium Voltage-Gated Channel Beta Subunit 2	Protein Coding	O60939	50	GC11M118163	0.903882	https://www.genecards.org/cgi-bin/carddisp.pl?gene=SCN2B
RPS3	Ribosomal Protein S3	Protein Coding	P23396	47	GC11P080303	0.903882	https://www.genecards.org/cgi-bin/carddisp.pl?gene=RPS3
TPM4	Tropomyosin 4	Protein Coding	P67936	45	GC19P089069	0.903882	https://www.genecards.org/cgi-bin/carddisp.pl?gene=TPM4

BCL10	BCL10 Immune Signaling Adaptor	Protein Coding	O95999	48	GC01M085265	0.903336	https://www.genecards.org/cgi-bin/carddisp.pl?gene=BCL10
ARHGAP1	Rho GTPase Activating Protein 1	Protein Coding	Q07960	47	GC11M113155	0.903336	https://www.genecards.org/cgi-bin/carddisp.pl?gene=ARHGAP1
TRP-AGG2-6	TRNA-Pro (Anticodon AGG) 2-6	RNA Gene		12	GC14M025622	0.902617	https://www.genecards.org/cgi-bin/carddisp.pl?gene=TRP-AGG2-6
TRP-AGG2-1	TRNA-Pro (Anticodon AGG) 2-1	RNA Gene		10	GC01M167715	0.902617	https://www.genecards.org/cgi-bin/carddisp.pl?gene=TRP-AGG2-1
TRP-AGG2-2	TRNA-Pro (Anticodon AGG) 2-2	RNA Gene		9	GC06P118628	0.902617	https://www.genecards.org/cgi-bin/carddisp.pl?gene=TRP-AGG2-2
TRP-AGG2-3	TRNA-Pro (Anticodon AGG) 2-3	RNA Gene		9	GC07P133911	0.902617	https://www.genecards.org/cgi-bin/carddisp.pl?gene=TRP-AGG2-3
TRP-AGG2-4	TRNA-Pro (Anticodon AGG) 2-4	RNA Gene		9	GC11P080320	0.902617	https://www.genecards.org/cgi-bin/carddisp.pl?gene=TRP-AGG2-4
TRP-AGG2-7	TRNA-Pro (Anticodon AGG) 2-7	RNA Gene		8	GC16M013454	0.902617	https://www.genecards.org/cgi-bin/carddisp.pl?gene=TRP-AGG2-7
TRP-AGG2-8	TRNA-Pro (Anticodon AGG) 2-8	RNA Gene		8	GC16P052486	0.902617	https://www.genecards.org/cgi-bin/carddisp.pl?gene=TRP-AGG2-8
ASXL3	ASXL Transcriptional Regulator 3	Protein Coding	Q9C0F0	38	GC18P033578	0.900295	https://www.genecards.org/cgi-bin/carddisp.pl?gene=ASXL3
JAK1	Janus Kinase 1	Protein Coding	P23458	58	GC01M064833	0.898558	https://www.genecards.org/cgi-bin/carddisp.pl?gene=JAK1
MIR296	MicroRNA 296	RNA Gene		19	GC20M058817	0.897693	https://www.genecards.org/cgi-bin/carddisp.pl?gene=MIR296

TRAF2	TNF Receptor Associated Factor 2	Protein Coding	Q12933	49	GC09P136881	0.89769	https://www.genecards.org/cgi-bin/carddisp.pl?gene=TRAF2
MIB1	MIB E3 Ubiquitin Protein Ligase 1	Protein Coding	Q86YT6	48	GC18P021704	0.897664	https://www.genecards.org/cgi-bin/carddisp.pl?gene=MIB1
MKRN2	Makorin Ring Finger Protein 2	Protein Coding	Q9H000	42	GC03P018398	0.896809	https://www.genecards.org/cgi-bin/carddisp.pl?gene=MKRN2
TPH2	Tryptophan Hydroxylase 2	Protein Coding	Q8IWU9	52	GC12P071938	0.896593	https://www.genecards.org/cgi-bin/carddisp.pl?gene=TPH2
HNRNPH1	Heterogeneous Nuclear Ribonucleoprotein H1	Protein Coding	P31943	46	GC05M179614	0.895834	https://www.genecards.org/cgi-bin/carddisp.pl?gene=HNRNPH1
DHRS9	Dehydrogenase/Reductase 9	Protein Coding	Q9BPW9	45	GC02P169064	0.895765	https://www.genecards.org/cgi-bin/carddisp.pl?gene=DHRS9
MIR877	MicroRNA 877	RNA Gene		20	GC06P030584	0.89477	https://www.genecards.org/cgi-bin/carddisp.pl?gene=MIR877
ASMER1	Adipocyte Associated Metabolic Related LncRNA 1	RNA Gene		10	GC21M015101	0.89335	https://www.genecards.org/cgi-bin/carddisp.pl?gene=ASMER1
TPT1-AS1	TPT1 Antisense RNA 1	RNA Gene		18	GC13P045341	0.892482	https://www.genecards.org/cgi-bin/carddisp.pl?gene=TPT1-AS1
CSTA	Cystatin A	Protein Coding	P01040	48	GC03P122325	0.887474	https://www.genecards.org/cgi-bin/carddisp.pl?gene=CSTA
HOXB7	Homeobox B7	Protein Coding	P09629	45	GC17M048607	0.887004	https://www.genecards.org/cgi-bin/carddisp.pl?gene=HOXB7
MYH8	Myosin Heavy Chain 8	Protein Coding	P13535	43	GC17M010390	0.886029	https://www.genecards.org/cgi-bin/carddisp.pl?gene=MYH8

CUL7	Cullin 7	Protein Coding	Q14999	45	GC06M043037	0.885004	https://www.genecards.org/cgi-bin/carddisp.pl?gene=CUL7
TNFRSF10C	TNF Receptor Superfamily Member 10c	Protein Coding	O14798	44	GC08P023102	0.884996	https://www.genecards.org/cgi-bin/carddisp.pl?gene=TNFRSF10C
MIR520D	MicroRNA 520d	RNA Gene		18	GC19P053720	0.884996	https://www.genecards.org/cgi-bin/carddisp.pl?gene=MIR520D
MIR363	MicroRNA 363	RNA Gene		16	GC0XM134393	0.884996	https://www.genecards.org/cgi-bin/carddisp.pl?gene=MIR363
LINC00668	Long Intergenic Non-Protein Coding RNA 668	RNA Gene		17	GC18M006922	0.882578	https://www.genecards.org/cgi-bin/carddisp.pl?gene=LINC00668
PRPS1	Phosphoribosyl Pyrophosphate Synthetase 1	Protein Coding	P60891	51	GC0XP107628	0.87904	https://www.genecards.org/cgi-bin/carddisp.pl?gene=PRPS1
GALNT17	Polypeptide N-Acetylgalactosaminyltransferase 17	Protein Coding	Q6IS24	43	GC07P081079	0.87904	https://www.genecards.org/cgi-bin/carddisp.pl?gene=GALNT17
PYGM	Glycogen Phosphorylase, Muscle Associated	Protein Coding	P11217	51	GC11M064746	0.878197	https://www.genecards.org/cgi-bin/carddisp.pl?gene=PYGM
HRG	Histidine Rich Glycoprotein	Protein Coding	P04196	50	GC03P186660	0.877313	https://www.genecards.org/cgi-bin/carddisp.pl?gene=HRG
SMPD1	Sphingomyelin Phosphodiesterase 1	Protein Coding	P17405	53	GC11P006390	0.876675	https://www.genecards.org/cgi-bin/carddisp.pl?gene=SMPD1
LAMB2	Laminin Subunit Beta 2	Protein Coding	P55268	50	GC03M049121	0.873098	https://www.genecards.org/cgi-bin/carddisp.pl?gene=LAMB2
HPX	Hemopexin	Protein Coding	P02790	46	GC11M009232	0.87227	https://www.genecards.org/cgi-bin/carddisp.pl?gene=HPX

FANCG	FA Complementation Group G	Protein Coding	O15287	48	GC09M035073	0.871705	https://www.genecards.org/cgi-bin/carddisp.pl?gene=FANCG
DOCK6	Dedicator Of Cytokinesis 6	Protein Coding	Q96HP0	47	GC19M011199	0.871705	https://www.genecards.org/cgi-bin/carddisp.pl?gene=DOCK6
B3GALT6	Beta-1,3-Galactosyltransferase 6	Protein Coding	Q96L58	44	GC01P001232	0.871705	https://www.genecards.org/cgi-bin/carddisp.pl?gene=B3GALT6
PGAP2	Post-GPI Attachment To Proteins 2	Protein Coding	Q9UHJ9	39	GC11P003797	0.871705	https://www.genecards.org/cgi-bin/carddisp.pl?gene=PGAP2
PALB2	Partner And Localizer Of BRCA2	Protein Coding	Q86YC2	48	GC16M023603	0.869741	https://www.genecards.org/cgi-bin/carddisp.pl?gene=PALB2
DDX11	DEAD/H-Box Helicase 11	Protein Coding	Q96FC9	46	GC12P031073	0.869645	https://www.genecards.org/cgi-bin/carddisp.pl?gene=DDX11
PDCD1LG2	Programmed Cell Death 1 Ligand 2	Protein Coding	Q9BQ51	47	GC09P005510	0.867457	https://www.genecards.org/cgi-bin/carddisp.pl?gene=PDCD1LG2
SGCA	Sarcoglycan Alpha	Protein Coding	Q16586	46	GC17P050164	0.864821	https://www.genecards.org/cgi-bin/carddisp.pl?gene=SGCA
DIAPH1	Diaphanous Related Formin 1	Protein Coding	O60610	50	GC05M141516	0.863282	https://www.genecards.org/cgi-bin/carddisp.pl?gene=DIAPH1
SNAP25	Synaptosome Associated Protein 25	Protein Coding	P60880	53	GC20P010211	0.854306	https://www.genecards.org/cgi-bin/carddisp.pl?gene=SNAP25
ARMS2	Age-Related Maculopathy Susceptibility 2	Protein Coding	P0C7Q2	34	GC10P122454	0.854306	https://www.genecards.org/cgi-bin/carddisp.pl?gene=ARMS2
PLA2G4D	Phospholipase A2 Group IVD	Protein Coding	Q86XP0	43	GC15M042067	0.853848	https://www.genecards.org/cgi-bin/carddisp.pl?gene=PLA2G4D

HP1BP3	Heterochromatin Protein 1 Binding Protein 3	Protein Coding	Q5SSJ5	41	GC01M020742	0.853126	https://www.genecards.org/cgi-bin/carddisp.pl?gene=HP1BP3
HNRNPUL2-BSCL2	HNRNPUL2-BSCL2 Readthrough (NMD Candidate)	RNA Gene		17	GC11M113401	0.85228	https://www.genecards.org/cgi-bin/carddisp.pl?gene=HNRNPUL2-BSCL2
HPR	Haptoglobin-Related Protein	Protein Coding	P00739	43	GC16P073657	0.851893	https://www.genecards.org/cgi-bin/carddisp.pl?gene=HPR
CNTN4	Contactin 4	Protein Coding	Q8IWV2	45	GC03P002117	0.851808	https://www.genecards.org/cgi-bin/carddisp.pl?gene=CNTN4
RNF8	Ring Finger Protein 8	Protein Coding	O76064	46	GC06P112173	0.84702	https://www.genecards.org/cgi-bin/carddisp.pl?gene=RNF8
SLC45A2	Solute Carrier Family 45 Member 2	Protein Coding	Q9UMX9	45	GC05M033944	0.84702	https://www.genecards.org/cgi-bin/carddisp.pl?gene=SLC45A2
NIPAL4	NIPA Like Domain Containing 4	Protein Coding	Q0D2K0	44	GC05P157460	0.84702	https://www.genecards.org/cgi-bin/carddisp.pl?gene=NIPAL4
PSME4	Proteasome Activator Subunit 4	Protein Coding	Q14997	44	GC02M053864	0.84702	https://www.genecards.org/cgi-bin/carddisp.pl?gene=PSME4
GABRR3	Gamma-Aminobutyric Acid Type A Receptor Subunit Rho3	Protein Coding	A8MPY1	40	GC03M097986	0.84702	https://www.genecards.org/cgi-bin/carddisp.pl?gene=GABRR3
NIPAL1	NIPA Like Domain Containing 1	Protein Coding	Q6NVV3	39	GC04P047917	0.84702	https://www.genecards.org/cgi-bin/carddisp.pl?gene=NIPAL1
NEURL4	Neuralized E3 Ubiquitin Protein Ligase 4	Protein Coding	Q96JN8	36	GC17M007315	0.84702	https://www.genecards.org/cgi-bin/carddisp.pl?gene=NEURL4
DEXI	Dexi Homolog	Protein Coding	O95424	33	GC16M010928	0.84702	https://www.genecards.org/cgi-bin/carddisp.pl?gene=DEXI

PAM	Peptidylglycine Alpha-Amidating Monooxygenase	Protein Coding	P19021	47	GC05P102753	0.846533	https://www.genecards.org/cgi-bin/carddisp.pl?gene=PAM
CLEC3B	C-Type Lectin Domain Family 3 Member B	Protein Coding	P05452	46	GC03P053722	0.845872	https://www.genecards.org/cgi-bin/carddisp.pl?gene=CLEC3B
MSN	Moesin	Protein Coding	P26038	52	GC0XP065588	0.84565	https://www.genecards.org/cgi-bin/carddisp.pl?gene=MSN
MIR323B	MicroRNA 323b	RNA Gene		17	GC14P113588	0.845221	https://www.genecards.org/cgi-bin/carddisp.pl?gene=MIR323B
NALT1	NOTCH1 Associated LncRNA In T Cell Acute Lymphoblastic Leukemia 1	RNA Gene		16	GC09P136547	0.845221	https://www.genecards.org/cgi-bin/carddisp.pl?gene=NALT1
ALK	ALK Receptor Tyrosine Kinase	Protein Coding	Q9UM73	54	GC02M029190	0.845031	https://www.genecards.org/cgi-bin/carddisp.pl?gene=ALK
DTNBP1	Dystrobrevin Binding Protein 1	Protein Coding	Q96EV8	46	GC06M015561	0.845014	https://www.genecards.org/cgi-bin/carddisp.pl?gene=DTNBP1
BMI1	BMI1 Proto-Oncogene, Polycomb Ring Finger	Protein Coding	P35226	47	GC10P022326	0.839665	https://www.genecards.org/cgi-bin/carddisp.pl?gene=BMI1
DOCK3	Dedicator Of Cytokinesis 3	Protein Coding	Q8IZD9	46	GC03P053968	0.838507	https://www.genecards.org/cgi-bin/carddisp.pl?gene=DOCK3
MMP26	Matrix Metallopeptidase 26	Protein Coding	Q9NRE1	41	GC11P005023	0.838507	https://www.genecards.org/cgi-bin/carddisp.pl?gene=MMP26
SKAP1-AS2	SKAP1 Antisense RNA 2	RNA Gene		16	GC17P086631	0.83576	https://www.genecards.org/cgi-bin/carddisp.pl?gene=SKAP1-AS2
SHMT2	Serine Hydroxymethyltransferase 2	Protein Coding	P34897	50	GC12P057229	0.835588	https://www.genecards.org/cgi-bin/carddisp.pl?gene=SHMT2

IGF2BP3	Insulin Like Growth Factor 2 MRNA Binding Protein 3	Protein Coding	O00425	46	GC07M023477	0.835387	https://www.genecards.org/cgi-bin/carddisp.pl?gene=IGF2BP3
GLRX	Glutaredoxin	Protein Coding	P35754	47	GC05M095752	0.83424	https://www.genecards.org/cgi-bin/carddisp.pl?gene=GLRX
TJP2	Tight Junction Protein 2	Protein Coding	Q9UDY2	49	GC09P069121	0.834202	https://www.genecards.org/cgi-bin/carddisp.pl?gene=TJP2
MIR345	MicroRNA 345	RNA Gene		20	GC14P100307	0.831929	https://www.genecards.org/cgi-bin/carddisp.pl?gene=MIR345
CNN1	Calponin 1	Protein Coding	P51911	44	GC19P088839	0.827881	https://www.genecards.org/cgi-bin/carddisp.pl?gene=CNN1
SLC2A1-DT	SLC2A1 Divergent Transcript	RNA Gene		18	GC01P043439	0.827881	https://www.genecards.org/cgi-bin/carddisp.pl?gene=SLC2A1-DT
C2CD3	C2 Domain Containing 3 Centriole Elongation Regulator	Protein Coding	Q4AC94	44	GC11M074012	0.826763	https://www.genecards.org/cgi-bin/carddisp.pl?gene=C2CD3
PDLIM1	PDZ And LIM Domain 1	Protein Coding	O00151	44	GC10M095237	0.826763	https://www.genecards.org/cgi-bin/carddisp.pl?gene=PDLIM1
CAP1	Cyclase Associated Actin Cytoskeleton Regulatory Protein 1	Protein Coding	Q01518	46	GC01P040262	0.825728	https://www.genecards.org/cgi-bin/carddisp.pl?gene=CAP1
SAA3P	Serum Amyloid A3, Pseudogene	Pseudogene		17	GC11M018112	0.824486	https://www.genecards.org/cgi-bin/carddisp.pl?gene=SAA3P
MEF2C	Myocyte Enhancer Factor 2C	Protein Coding	Q06413	52	GC05M088718	0.821736	https://www.genecards.org/cgi-bin/carddisp.pl?gene=MEF2C
DAZL	Deleted In Azoospermia Like	Protein Coding	Q92904	44	GC03M016586	0.821442	https://www.genecards.org/cgi-bin/carddisp.pl?gene=DAZL

UQCRC1	Ubiquinol-Cytochrome C Reductase Core Protein 1	Protein Coding	P31930	47	GC03M048598	0.820552	https://www.genecards.org/cgi-bin/carddisp.pl?gene=UQCRC1
MIR4697	MicroRNA 4697	RNA Gene		12	GC11M133898	0.819799	https://www.genecards.org/cgi-bin/carddisp.pl?gene=MIR4697
RNF217-AS1	RNF217 Antisense RNA 1 (Head To Head)	RNA Gene		18	GC06M124563	0.818984	https://www.genecards.org/cgi-bin/carddisp.pl?gene=RNF217-AS1
TUBB	Tubulin Beta Class I	Protein Coding	P07437	53	GC06P111971	0.817429	https://www.genecards.org/cgi-bin/carddisp.pl?gene=TUBB
GNG12	G Protein Subunit Gamma 12	Protein Coding	Q9UBI6	41	GC01M067701	0.815171	https://www.genecards.org/cgi-bin/carddisp.pl?gene=GNG12
HSPA14	Heat Shock Protein Family A (Hsp70) Member 14	Protein Coding	Q0VDF9	44	GC10P014860	0.813295	https://www.genecards.org/cgi-bin/carddisp.pl?gene=HSPA14
ATL1	Atlastin GTPase 1	Protein Coding	Q8WXF7	45	GC14P050532	0.811546	https://www.genecards.org/cgi-bin/carddisp.pl?gene=ATL1
HNRNPA1	Heterogeneous Nuclear Ribonucleoprotein A1	Protein Coding	P09651	52	GC12P054280	0.81125	https://www.genecards.org/cgi-bin/carddisp.pl?gene=HNRNPA1
IRAIN	IGF1R Antisense Imprinted Non-Protein Coding RNA	RNA Gene		17	GC15M098645	0.810716	https://www.genecards.org/cgi-bin/carddisp.pl?gene=IRAIN
SAA4	Serum Amyloid A4, Constitutive	Protein Coding	P35542	42	GC11M018234	0.810535	https://www.genecards.org/cgi-bin/carddisp.pl?gene=SAA4
PROCR	Protein C Receptor	Protein Coding	Q9UNN8	46	GC20P035171	0.806739	https://www.genecards.org/cgi-bin/carddisp.pl?gene=PROCR
LINC00649	Long Intergenic Non-Protein Coding RNA 649	RNA Gene		16	GC21P033915	0.803397	https://www.genecards.org/cgi-bin/carddisp.pl?gene=LINC00649

CKS1BP2	CDC28 Protein Kinase Regulatory Subunit 1B Pseudogene 2	Pseudogene		9	GC10P029697	0.803397	https://www.genecards.org/cgi-bin/carddisp.pl?gene=CKS1BP2
USP14	Ubiquitin Specific Peptidase 14	Protein Coding	P54578	48	GC18P000158	0.802421	https://www.genecards.org/cgi-bin/carddisp.pl?gene=USP14
HOMER1	Homer Scaffold Protein 1	Protein Coding	Q86YM7	46	GC05M079372	0.797143	https://www.genecards.org/cgi-bin/carddisp.pl?gene=HOMER1
RPS6	Ribosomal Protein S6	Protein Coding	P62753	48	GC09M019375	0.796159	https://www.genecards.org/cgi-bin/carddisp.pl?gene=RPS6
ITGA4	Integrin Subunit Alpha 4	Protein Coding	P13612	52	GC02P181456	0.793797	https://www.genecards.org/cgi-bin/carddisp.pl?gene=ITGA4
PORCN	Porcupine O-Acyltransferase	Protein Coding	Q9H237	46	GC0XP052553	0.793797	https://www.genecards.org/cgi-bin/carddisp.pl?gene=PORCN
SOX17	SRY-Box Transcription Factor 17	Protein Coding	Q9H6I2	46	GC08P054457	0.793797	https://www.genecards.org/cgi-bin/carddisp.pl?gene=SOX17
CLDN4	Claudin 4	Protein Coding	O14493	45	GC07P073799	0.793797	https://www.genecards.org/cgi-bin/carddisp.pl?gene=CLDN4
SEMA6A	Semaphorin 6A	Protein Coding	Q9H2E6	44	GC05M116443	0.793797	https://www.genecards.org/cgi-bin/carddisp.pl?gene=SEMA6A
NKX6-2	NK6 Homeobox 2	Protein Coding	Q9C056	42	GC10M132783	0.793797	https://www.genecards.org/cgi-bin/carddisp.pl?gene=NKX6-2
LOC106020709	Distal SMS-REP Block C Recombination Region	Functional Element		3	GC17P016843	0.793797	https://www.genecards.org/cgi-bin/carddisp.pl?gene=LOC106020709
LOC106020710	Proximal SMS-REP Block C Recombination Region	Functional Element		3	GC17P088088	0.793797	https://www.genecards.org/cgi-bin/carddisp.pl?gene=LOC106020710

LOC108745275	Distal SMS-REP Block A Recombination Region	Functional Element		3	GC17P016808	0.793797	https://www.genecards.org/cgi-bin/carddisp.pl?gene=LOC108745275
LOC108745276	Proximal SMS-REP Block A Recombination Region	Functional Element		3	GC17P088095	0.793797	https://www.genecards.org/cgi-bin/carddisp.pl?gene=LOC108745276
KRIT1	KRIT1 Ankyrin Repeat Containing	Protein Coding	O00522	46	GC07M092198	0.782676	https://www.genecards.org/cgi-bin/carddisp.pl?gene=KRIT1
PXN	Paxillin	Protein Coding	P49023	50	GC12M120210	0.781277	https://www.genecards.org/cgi-bin/carddisp.pl?gene=PXN
CR1	Complement C3b/C4b Receptor 1 (Knops Blood Group)	Protein Coding	P17927	48	GC01P207496	0.781277	https://www.genecards.org/cgi-bin/carddisp.pl?gene=CR1
SFPQ	Splicing Factor Proline And Glutamine Rich	Protein Coding	P23246	48	GC01M035176	0.779973	https://www.genecards.org/cgi-bin/carddisp.pl?gene=SFPQ
ETV7	ETS Variant Transcription Factor 7	Protein Coding	Q9Y603	38	GC06M084097	0.776674	https://www.genecards.org/cgi-bin/carddisp.pl?gene=ETV7
PTGIS	Prostaglandin I2 Synthase	Protein Coding	Q16647	48	GC20M049503	0.774836	https://www.genecards.org/cgi-bin/carddisp.pl?gene=PTGIS
FCGR1A	Fc Gamma Receptor Ia	Protein Coding	P12314	46	GC01P156688	0.774149	https://www.genecards.org/cgi-bin/carddisp.pl?gene=FCGR1A
SUN2	Sad1 And UNC84 Domain Containing 2	Protein Coding	Q9UH99	44	GC22M070423	0.77165	https://www.genecards.org/cgi-bin/carddisp.pl?gene=SUN2
YBX3	Y-Box Binding Protein 3	Protein Coding	P16989	44	GC12M025982	0.771115	https://www.genecards.org/cgi-bin/carddisp.pl?gene=YBX3
CNTN2	Contactin 2	Protein Coding	Q02246	50	GC01P205043	0.770209	https://www.genecards.org/cgi-bin/carddisp.pl?gene=CNTN2

WDR82	WD Repeat Domain 82	Protein Coding	Q6UXN9	40	GC03M052254	0.769722	https://www.genecards.org/cgi-bin/carddisp.pl?gene=WDR82
FANCF	FA Complementation Group F	Protein Coding	Q9NPI8	46	GC11M022600	0.767986	https://www.genecards.org/cgi-bin/carddisp.pl?gene=FANCF
PIGY	Phosphatidylinositol Glycan Anchor Biosynthesis Class Y	Protein Coding	Q3MUY2	38	GC04M088520	0.767986	https://www.genecards.org/cgi-bin/carddisp.pl?gene=PIGY
PIP5K1C	Phosphatidylinositol-4-Phosphate 5-Kinase Type 1 Gamma	Protein Coding	O60331	52	GC19M010753	0.765926	https://www.genecards.org/cgi-bin/carddisp.pl?gene=PIP5K1C
MPDZ	Multiple PDZ Domain Crumbs Cell Polarity Complex Component	Protein Coding	O75970	47	GC09M013105	0.765926	https://www.genecards.org/cgi-bin/carddisp.pl?gene=MPDZ
SCARF2	Scavenger Receptor Class F Member 2	Protein Coding	Q96GP6	44	GC22M020424	0.765926	https://www.genecards.org/cgi-bin/carddisp.pl?gene=SCARF2
LAG3	Lymphocyte Activating 3	Protein Coding	P18627	45	GC12P030488	0.763738	https://www.genecards.org/cgi-bin/carddisp.pl?gene=LAG3
TKT	Transketolase	Protein Coding	P29401	50	GC03M053224	0.758802	https://www.genecards.org/cgi-bin/carddisp.pl?gene=TKT
CXCR6	C-X-C Motif Chemokine Receptor 6	Protein Coding	O00574	44	GC03P053740	0.758802	https://www.genecards.org/cgi-bin/carddisp.pl?gene=CXCR6
MRPL23	Mitochondrial Ribosomal Protein L23	Protein Coding	Q16540	42	GC11P004516	0.755344	https://www.genecards.org/cgi-bin/carddisp.pl?gene=MRPL23
ACTC1	Actin Alpha Cardiac Muscle 1	Protein Coding	P68032	48	GC15M034790	0.754029	https://www.genecards.org/cgi-bin/carddisp.pl?gene=ACTC1
EML1	EMAP Like 1	Protein Coding	O00423	45	GC14P099737	0.753334	https://www.genecards.org/cgi-bin/carddisp.pl?gene=EML1

ADAM10	ADAM Metallopeptidase Domain 10	Protein Coding	O14672	57	GC15M058588	0.750836	https://www.genecards.org/cgi-bin/carddisp.pl?gene=ADAM10
CD27	CD27 Molecule	Protein Coding	P26842	51	GC12P030440	0.750836	https://www.genecards.org/cgi-bin/carddisp.pl?gene=CD27
NOP2	NOP2 Nucleolar Protein	Protein Coding	P46087	43	GC12M006556	0.750836	https://www.genecards.org/cgi-bin/carddisp.pl?gene=NOP2
BHLHA15	Basic Helix-Loop-Helix Family Member A15	Protein Coding	Q7RTS1	40	GC07P098211	0.750836	https://www.genecards.org/cgi-bin/carddisp.pl?gene=BHLHA15
OR51A4	Olfactory Receptor Family 51 Subfamily A Member 4	Protein Coding	Q8NGJ6	28	GC11M009134	0.750836	https://www.genecards.org/cgi-bin/carddisp.pl?gene=OR51A4
BLZF1	Basic Leucine Zipper Nuclear Factor 1	Protein Coding	Q9H2G9	42	GC01P169367	0.749664	https://www.genecards.org/cgi-bin/carddisp.pl?gene=BLZF1
MIR1304	MicroRNA 1304	RNA Gene		17	GC11M114183	0.748871	https://www.genecards.org/cgi-bin/carddisp.pl?gene=MIR1304
H2BC21	H2B Clustered Histone 21	Protein Coding	Q16778	44	GC01M157482	0.746124	https://www.genecards.org/cgi-bin/carddisp.pl?gene=H2BC21
H2AC20	H2A Clustered Histone 20	Protein Coding	Q16777	41	GC01P156718	0.746124	https://www.genecards.org/cgi-bin/carddisp.pl?gene=H2AC20
FUNDC2	FUN14 Domain Containing 2	Protein Coding	Q9BWH2	39	GC0XP155025	0.746124	https://www.genecards.org/cgi-bin/carddisp.pl?gene=FUNDC2
AS3MT	Arsenite Methyltransferase	Protein Coding	Q9HBK9	44	GC10P102869	0.742639	https://www.genecards.org/cgi-bin/carddisp.pl?gene=AS3MT
CORT	Cortistatin	Protein Coding	O00230	38	GC01P010719	0.742639	https://www.genecards.org/cgi-bin/carddisp.pl?gene=CORT

MIR651	MicroRNA 651	RNA Gene		17	GC0XP008127	0.742639	https://www.genecards.org/cgi-bin/carddisp.pl?gene=MIR651
MIR6721	MicroRNA 6721	RNA Gene		10	GC06M032170	0.742639	https://www.genecards.org/cgi-bin/carddisp.pl?gene=MIR6721
LOC108663985	Calcium Voltage-Gated Channel Subunit Alpha1 A Repeat Instability Region	Functional Element		4	GC19P088957	0.742639	https://www.genecards.org/cgi-bin/carddisp.pl?gene=LOC108663985
CORO7	Coronin 7	Protein Coding	P57737	40	GC16M013506	0.742286	https://www.genecards.org/cgi-bin/carddisp.pl?gene=CORO7
EDC4	Enhancer Of MRNA Decapping 4	Protein Coding	Q6P2E9	42	GC16P067873	0.741501	https://www.genecards.org/cgi-bin/carddisp.pl?gene=EDC4
XRCC6	X-Ray Repair Cross Complementing 6	Protein Coding	P12956	49	GC22P056811	0.738149	https://www.genecards.org/cgi-bin/carddisp.pl?gene=XRCC6
SKP2	S-Phase Kinase Associated Protein 2	Protein Coding	Q13309	48	GC05P036151	0.738149	https://www.genecards.org/cgi-bin/carddisp.pl?gene=SKP2
GRHL2	Grainyhead Like Transcription Factor 2	Protein Coding	Q6ISB3	45	GC08P101492	0.738149	https://www.genecards.org/cgi-bin/carddisp.pl?gene=GRHL2
MIR124-1	MicroRNA 124-1	RNA Gene		20	GC08M009903	0.738149	https://www.genecards.org/cgi-bin/carddisp.pl?gene=MIR124-1
CSF1R	Colony Stimulating Factor 1 Receptor	Protein Coding	P07333	56	GC05M150053	0.737771	https://www.genecards.org/cgi-bin/carddisp.pl?gene=CSF1R
NAGA	Alpha-N-Acetylgalactosaminidase	Protein Coding	P17050	50	GC22M042058	0.737011	https://www.genecards.org/cgi-bin/carddisp.pl?gene=NAGA
CYP2J2	Cytochrome P450 Family 2 Subfamily J Member 2	Protein Coding	P51589	46	GC01M059893	0.735284	https://www.genecards.org/cgi-bin/carddisp.pl?gene=CYP2J2

LOC105371046	Uncharacterized LOC105371046	RNA Gene		11	GC16P052391	0.734988	https://www.genecards.org/cgi-bin/carddisp.pl?gene=LOC105371046
NAP1L4	Nucleosome Assembly Protein 1 Like 4	Protein Coding	Q99733	42	GC11M002944	0.734791	https://www.genecards.org/cgi-bin/carddisp.pl?gene=NAP1L4
NUP98	Nucleoporin 98 And 96 Precursor	Protein Coding	P52948	49	GC11M003671	0.734788	https://www.genecards.org/cgi-bin/carddisp.pl?gene=NUP98
HIPK3	Homeodomain Interacting Protein Kinase 3	Protein Coding	Q9H422	45	GC11P033355	0.734788	https://www.genecards.org/cgi-bin/carddisp.pl?gene=HIPK3
PPA2	Inorganic Pyrophosphatase 2	Protein Coding	Q9H2U2	45	GC04M105369	0.734788	https://www.genecards.org/cgi-bin/carddisp.pl?gene=PPA2
KLRB1	Killer Cell Lectin Like Receptor B1	Protein Coding	Q12918	44	GC12M025965	0.734788	https://www.genecards.org/cgi-bin/carddisp.pl?gene=KLRB1
PRDM7	PR/SET Domain 7	Protein Coding	Q9NQW5	38	GC16M090056	0.734788	https://www.genecards.org/cgi-bin/carddisp.pl?gene=PRDM7
OR51V1	Olfactory Receptor Family 51 Subfamily V Member 1	Protein Coding	Q9H2C8	34	GC11M009147	0.734788	https://www.genecards.org/cgi-bin/carddisp.pl?gene=OR51V1
LINC02861	Long Intergenic Non-Protein Coding RNA 2861	RNA Gene		15	GC16M013494	0.734788	https://www.genecards.org/cgi-bin/carddisp.pl?gene=LINC02861
SNORD81	Small Nucleolar RNA, C/D Box 81	RNA Gene		13	GC01M174805	0.734788	https://www.genecards.org/cgi-bin/carddisp.pl?gene=SNORD81
PEE2	Preeclampsia/Eclampsia 2	Genetic Locus		2	GC02U901135	0.730521	https://www.genecards.org/cgi-bin/carddisp.pl?gene=PEE2
PEE3	Preeclampsia/Eclampsia 3	Genetic Locus		2	GC09U900850	0.730521	https://www.genecards.org/cgi-bin/carddisp.pl?gene=PEE3

CERT1	Ceramide Transporter 1	Protein Coding	Q9Y5P4	45	GC05M075356	0.72873	https://www.genecards.org/cgi-bin/carddisp.pl?gene=CERT1
LMNB1	Lamin B1	Protein Coding	P20700	51	GC05P126776	0.728688	https://www.genecards.org/cgi-bin/carddisp.pl?gene=LMNB1
NT5DC1	5'-Nucleotidase Domain Containing 1	Protein Coding	Q5TFE4	39	GC06P116100	0.728582	https://www.genecards.org/cgi-bin/carddisp.pl?gene=NT5DC1
SLCO2A1	Solute Carrier Organic Anion Transporter Family Member 2A1	Protein Coding	Q92959	48	GC03M133932	0.726671	https://www.genecards.org/cgi-bin/carddisp.pl?gene=SLCO2A1
HSD17B3	Hydroxysteroid 17-Beta Dehydrogenase 3	Protein Coding	P37058	50	GC09M104536	0.725106	https://www.genecards.org/cgi-bin/carddisp.pl?gene=HSD17B3
PRKCE	Protein Kinase C Epsilon	Protein Coding	Q02156	52	GC02P045651	0.724948	https://www.genecards.org/cgi-bin/carddisp.pl?gene=PRKCE
TNFAIP3	TNF Alpha Induced Protein 3	Protein Coding	P21580	52	GC06P137866	0.72456	https://www.genecards.org/cgi-bin/carddisp.pl?gene=TNFAIP3
FRMD7	FERM Domain Containing 7	Protein Coding	Q6ZUT3	43	GC0XM132077	0.719567	https://www.genecards.org/cgi-bin/carddisp.pl?gene=FRMD7
SLC12A2	Solute Carrier Family 12 Member 2	Protein Coding	P55011	53	GC05P128083	0.718017	https://www.genecards.org/cgi-bin/carddisp.pl?gene=SLC12A2
PTPRB	Protein Tyrosine Phosphatase Receptor Type B	Protein Coding	P23467	49	GC12M070516	0.718017	https://www.genecards.org/cgi-bin/carddisp.pl?gene=PTPRB
SLC12A4	Solute Carrier Family 12 Member 4	Protein Coding	Q9UP95	48	GC16M072100	0.718017	https://www.genecards.org/cgi-bin/carddisp.pl?gene=SLC12A4
PCDHA9	Protocadherin Alpha 9	Protein Coding	Q9Y5H5	40	GC05P148706	0.718017	https://www.genecards.org/cgi-bin/carddisp.pl?gene=PCDHA9

RNF148	Ring Finger Protein 148	Protein Coding	Q8N7C7	35	GC07M122701	0.718017	https://www.genecards.org/cgi-bin/carddisp.pl?gene=RNF148
AMOT	Angiomotin	Protein Coding	Q4VCS5	44	GC0XM112774	0.717733	https://www.genecards.org/cgi-bin/carddisp.pl?gene=AMOT
CD209	CD209 Molecule	Protein Coding	Q9NNX6	47	GC19M007739	0.716201	https://www.genecards.org/cgi-bin/carddisp.pl?gene=CD209
MIR548C	MicroRNA 548c	RNA Gene		16	GC12P064622	0.713287	https://www.genecards.org/cgi-bin/carddisp.pl?gene=MIR548C
HSPA9	Heat Shock Protein Family A (Hsp70) Member 9	Protein Coding	P38646	52	GC05M138554	0.712824	https://www.genecards.org/cgi-bin/carddisp.pl?gene=HSPA9
IARS1	Isoleucyl-TRNA Synthetase 1	Protein Coding	P41252	48	GC09M104378	0.712337	https://www.genecards.org/cgi-bin/carddisp.pl?gene=IARS1
SLC25A24	Solute Carrier Family 25 Member 24	Protein Coding	Q6NUK1	47	GC01M108134	0.712337	https://www.genecards.org/cgi-bin/carddisp.pl?gene=SLC25A24
FARSA	Phenylalanyl-TRNA Synthetase Subunit Alpha	Protein Coding	Q9Y285	46	GC19M012922	0.712337	https://www.genecards.org/cgi-bin/carddisp.pl?gene=FARSA
S1PR2	Sphingosine-1-Phosphate Receptor 2	Protein Coding	O95136	48	GC19M011900	0.71181	https://www.genecards.org/cgi-bin/carddisp.pl?gene=S1PR2
HNRNPK	Heterogeneous Nuclear Ribonucleoprotein K	Protein Coding	P61978	49	GC09M104168	0.711712	https://www.genecards.org/cgi-bin/carddisp.pl?gene=HNRNPK
HELLS	Helicase, Lymphoid Specific	Protein Coding	Q9NRZ9	49	GC10P101122	0.71106	https://www.genecards.org/cgi-bin/carddisp.pl?gene=HELLS
CAPZB	Capping Actin Protein Of Muscle Z-Line Subunit Beta	Protein Coding	P47756	45	GC01M019339	0.710277	https://www.genecards.org/cgi-bin/carddisp.pl?gene=CAPZB

OBSCN	Obscurin, Cytoskeletal Calmodulin And Titin-Interacting RhoGEF	Protein Coding	Q5VST9	44	GC01P228208	0.710277	https://www.genecards.org/cgi-bin/carddisp.pl?gene=OBSCN
CDIPT	CDP-Diacylglycerol--Inositol 3-Phosphatidyltransferase	Protein Coding	O14735	45	GC16M041620	0.709889	https://www.genecards.org/cgi-bin/carddisp.pl?gene=CDIPT
DNAH9	Dynein Axonemal Heavy Chain 9	Protein Coding	Q9NYC9	41	GC17P011598	0.706469	https://www.genecards.org/cgi-bin/carddisp.pl?gene=DNAH9
GPR143	G Protein-Coupled Receptor 143	Protein Coding	P51810	46	GC0XM009725	0.704824	https://www.genecards.org/cgi-bin/carddisp.pl?gene=GPR143
TRIM72	Tripartite Motif Containing 72	Protein Coding	Q6ZMU5	40	GC16P054240	0.704743	https://www.genecards.org/cgi-bin/carddisp.pl?gene=TRIM72
MGAT5	Alpha-1,6-Mannosylglycoprotein 6-Beta-N-Acetylglucosaminyltransferase	Protein Coding	Q09328	45	GC02P134119	0.703502	https://www.genecards.org/cgi-bin/carddisp.pl?gene=MGAT5
EREG	Epiregulin	Protein Coding	O14944	46	GC04P074366	0.699677	https://www.genecards.org/cgi-bin/carddisp.pl?gene=EREG
LHFPL6	LHFPL Tetraspan Subfamily Member 6	Protein Coding	Q9Y693	35	GC13M039209	0.699677	https://www.genecards.org/cgi-bin/carddisp.pl?gene=LHFPL6
LOC110011216	Paired Like Homeobox 2b Polyalanine Repeat Instability Region	Functional Element		4	GC04P041745	0.699191	https://www.genecards.org/cgi-bin/carddisp.pl?gene=LOC110011216
PNP	Purine Nucleoside Phosphorylase	Protein Coding	P00491	53	GC14P039927	0.698238	https://www.genecards.org/cgi-bin/carddisp.pl?gene=PNP
IGHMBP2	Immunoglobulin Mu DNA Binding Protein 2	Protein Coding	P38935	46	GC11P068903	0.694578	https://www.genecards.org/cgi-bin/carddisp.pl?gene=IGHMBP2
CHRNA1	Cholinergic Receptor Nicotinic Alpha 1 Subunit	Protein Coding	P02708	50	GC02M174747	0.69389	https://www.genecards.org/cgi-bin/carddisp.pl?gene=CHRNA1

CHRNB1	Cholinergic Receptor Nicotinic Beta 1 Subunit	Protein Coding	P11230	50	GC17P014014	0.69389	https://www.genecards.org/cgi-bin/carddisp.pl?gene=CHRNB1
WDR73	WD Repeat Domain 73	Protein Coding	Q6P4I2	42	GC15M084639	0.693451	https://www.genecards.org/cgi-bin/carddisp.pl?gene=WDR73
CD79B	CD79b Molecule	Protein Coding	P40259	50	GC17M063928	0.691391	https://www.genecards.org/cgi-bin/carddisp.pl?gene=CD79B
NLGN3	Neuroligin 3	Protein Coding	Q9NZ94	48	GC0XP071144	0.689932	https://www.genecards.org/cgi-bin/carddisp.pl?gene=NLGN3
SLC34A3	Solute Carrier Family 34 Member 3	Protein Coding	Q8N130	47	GC09P137623	0.688114	https://www.genecards.org/cgi-bin/carddisp.pl?gene=SLC34A3
KCNJ8	Potassium Inwardly Rectifying Channel Subfamily J Member 8	Protein Coding	Q15842	47	GC12M021764	0.687521	https://www.genecards.org/cgi-bin/carddisp.pl?gene=KCNJ8
JPH3	Junctophilin 3	Protein Coding	Q8WXH2	45	GC16P087601	0.68572	https://www.genecards.org/cgi-bin/carddisp.pl?gene=JPH3
TCF12	Transcription Factor 12	Protein Coding	Q99081	51	GC15P056918	0.68397	https://www.genecards.org/cgi-bin/carddisp.pl?gene=TCF12
GRM6	Glutamate Metabotropic Receptor 6	Protein Coding	O15303	50	GC05M178978	0.683269	https://www.genecards.org/cgi-bin/carddisp.pl?gene=GRM6
DAB2IP	DAB2 Interacting Protein	Protein Coding	Q5VWQ8	44	GC09P121715	0.678101	https://www.genecards.org/cgi-bin/carddisp.pl?gene=DAB2IP
ATP7B	ATPase Copper Transporting Beta	Protein Coding	P35670	52	GC13M051930	0.674372	https://www.genecards.org/cgi-bin/carddisp.pl?gene=ATP7B
ADH7	Alcohol Dehydrogenase 7 (Class IV), Mu Or Sigma Polypeptide	Protein Coding	P40394	47	GC04M099412	0.673871	https://www.genecards.org/cgi-bin/carddisp.pl?gene=ADH7

TIMM8A	Translocase Of Inner Mitochondrial Membrane 8A	Protein Coding	O60220	46	GC0XM101345	0.673871	https://www.genecards.org/cgi-bin/carddisp.pl?gene=TIMM8A
PARL	Presenilin Associated Rhomboid Like	Protein Coding	Q9H300	44	GC03M183826	0.673871	https://www.genecards.org/cgi-bin/carddisp.pl?gene=PARL
RHBG	Rh Family B Glycoprotein	Protein Coding	Q9H310	42	GC01P157382	0.673871	https://www.genecards.org/cgi-bin/carddisp.pl?gene=RHBG
LEKR1	Leucine, Glutamate And Lysine Rich 1	Protein Coding	Q6ZMV7	33	GC03P156825	0.672456	https://www.genecards.org/cgi-bin/carddisp.pl?gene=LEKR1
ASCL2	Achaete-Scute Family BHLH Transcription Factor 2	Protein Coding	Q99929	40	GC11M009018	0.671983	https://www.genecards.org/cgi-bin/carddisp.pl?gene=ASCL2
MT-TY	Mitochondrially Encoded TRNA-Tyr (UAU/C)	RNA Gene		13	GCMTM005828	0.670381	https://www.genecards.org/cgi-bin/carddisp.pl?gene=MT-TY
STMN1	Stathmin 1	Protein Coding	P16949	48	GC01M025884	0.667882	https://www.genecards.org/cgi-bin/carddisp.pl?gene=STMN1
MRE11	MRE11 Homolog, Double Strand Break Repair Nuclease	Protein Coding	P49959	52	GC11M114195	0.664471	https://www.genecards.org/cgi-bin/carddisp.pl?gene=MRE11
MCAM	Melanoma Cell Adhesion Molecule	Protein Coding	P43121	46	GC11M119308	0.662097	https://www.genecards.org/cgi-bin/carddisp.pl?gene=MCAM
CENPC	Centromere Protein C	Protein Coding	Q03188	40	GC04M067491	0.657759	https://www.genecards.org/cgi-bin/carddisp.pl?gene=CENPC
LEMD3	LEM Domain Containing 3	Protein Coding	Q9Y2U8	45	GC12P065169	0.656966	https://www.genecards.org/cgi-bin/carddisp.pl?gene=LEMD3
TBC1D24	TBC1 Domain Family Member 24	Protein Coding	Q9ULP9	43	GC16P002475	0.656966	https://www.genecards.org/cgi-bin/carddisp.pl?gene=TBC1D24

TEX11	Testis Expressed 11	Protein Coding	Q8IYF3	41	GC0XM070635	0.656966	https://www.genecards.org/cgi-bin/carddisp.pl?gene=TEX11
DAZ1	Deleted In Azoospermia 1	Protein Coding	Q9NQZ3	35	GC0YM023129	0.656966	https://www.genecards.org/cgi-bin/carddisp.pl?gene=DAZ1
B9D1	B9 Domain Containing 1	Protein Coding	Q9UPM9	40	GC17M019334	0.654813	https://www.genecards.org/cgi-bin/carddisp.pl?gene=B9D1
MIR196B	MicroRNA 196b	RNA Gene		22	GC07M027843	0.653802	https://www.genecards.org/cgi-bin/carddisp.pl?gene=MIR196B
ITGA8	Integrin Subunit Alpha 8	Protein Coding	P53708	49	GC10M015513	0.653676	https://www.genecards.org/cgi-bin/carddisp.pl?gene=ITGA8
ELAVL2	ELAV Like RNA Binding Protein 2	Protein Coding	Q12926	44	GC09M023690	0.651616	https://www.genecards.org/cgi-bin/carddisp.pl?gene=ELAVL2
CCR4	C-C Motif Chemokine Receptor 4	Protein Coding	P51679	49	GC03P032951	0.65027	https://www.genecards.org/cgi-bin/carddisp.pl?gene=CCR4
ALX1	ALX Homeobox 1	Protein Coding	Q15699	44	GC12P085279	0.648238	https://www.genecards.org/cgi-bin/carddisp.pl?gene=ALX1
POLB	DNA Polymerase Beta	Protein Coding	P06746	50	GC08P042338	0.645152	https://www.genecards.org/cgi-bin/carddisp.pl?gene=POLB
WNT1	Wnt Family Member 1	Protein Coding	P04628	51	GC12P050729	0.642551	https://www.genecards.org/cgi-bin/carddisp.pl?gene=WNT1
ANK2	Ankyrin 2	Protein Coding	Q01484	45	GC04P112722	0.639839	https://www.genecards.org/cgi-bin/carddisp.pl?gene=ANK2
SDF2	Stromal Cell Derived Factor 2	Protein Coding	Q99470	41	GC17M063260	0.639839	https://www.genecards.org/cgi-bin/carddisp.pl?gene=SDF2

MIR548X	MicroRNA 548x	RNA Gene		11	GC21M018686	0.638263	https://www.genecards.org/cgi-bin/carddisp.pl?gene=MIR548X
EDA	Ectodysplasin A	Protein Coding	Q92838	47	GC0XP069618	0.638072	https://www.genecards.org/cgi-bin/carddisp.pl?gene=EDA
CD3E	CD3 Epsilon Subunit Of T-Cell Receptor Complex	Protein Coding	P07766	52	GC11P118304	0.637782	https://www.genecards.org/cgi-bin/carddisp.pl?gene=CD3E
SGCG	Sarcoglycan Gamma	Protein Coding	Q13326	47	GC13P023160	0.637424	https://www.genecards.org/cgi-bin/carddisp.pl?gene=SGCG
LDLRAP1	Low Density Lipoprotein Receptor Adaptor Protein 1	Protein Coding	Q5SW96	45	GC01P025543	0.637415	https://www.genecards.org/cgi-bin/carddisp.pl?gene=LDLRAP1
CDC42	Cell Division Cycle 42	Protein Coding	P60953	55	GC01P022052	0.636525	https://www.genecards.org/cgi-bin/carddisp.pl?gene=CDC42
GAB2	GRB2 Associated Binding Protein 2	Protein Coding	Q9UQC2	47	GC11M078215	0.634312	https://www.genecards.org/cgi-bin/carddisp.pl?gene=GAB2
NFAT5	Nuclear Factor Of Activated T Cells 5	Protein Coding	O94916	46	GC16P069565	0.634312	https://www.genecards.org/cgi-bin/carddisp.pl?gene=NFAT5
STX16-NPEPL1	STX16-NPEPL1 Readthrough (NMD Candidate)	RNA Gene		17	GC20P058651	0.63343	https://www.genecards.org/cgi-bin/carddisp.pl?gene=STX16-NPEPL1
ERFE	Erythroferrone	Protein Coding	Q4G0M1	36	GC02P238159	0.632301	https://www.genecards.org/cgi-bin/carddisp.pl?gene=ERFE
GATA1	GATA Binding Protein 1	Protein Coding	P15976	51	GC0XP048786	0.62761	https://www.genecards.org/cgi-bin/carddisp.pl?gene=GATA1
MN1	MN1 Proto-Oncogene, Transcriptional Regulator	Protein Coding	Q10571	46	GC22M027748	0.62761	https://www.genecards.org/cgi-bin/carddisp.pl?gene=MN1

HEPACAM	Hepatic And Glial Cell Adhesion Molecule	Protein Coding	Q14CZ8	45	GC11M124919	0.62761	https://www.genecards.org/cgi-bin/carddisp.pl?gene=HEPACAM
VSX2	Visual System Homeobox 2	Protein Coding	P58304	44	GC14P074239	0.62761	https://www.genecards.org/cgi-bin/carddisp.pl?gene=VSX2
GNPTG	N-Acetylglucosamine-1-Phosphate Transferase Subunit Gamma	Protein Coding	Q9UJJ9	41	GC16P001351	0.62761	https://www.genecards.org/cgi-bin/carddisp.pl?gene=GNPTG
KIAA0586	KIAA0586	Protein Coding	Q9BVV6	39	GC14P058427	0.62761	https://www.genecards.org/cgi-bin/carddisp.pl?gene=KIAA0586
LOC126860395	BRD4-Independent Group 4 Enhancer GRCh37_chr8:57079228-57080427	Functional Element		3	GC08P056242	0.62761	https://www.genecards.org/cgi-bin/carddisp.pl?gene=LOC126860395
GRK2	G Protein-Coupled Receptor Kinase 2	Protein Coding	P25098	51	GC11P067266	0.627226	https://www.genecards.org/cgi-bin/carddisp.pl?gene=GRK2
WDR62	WD Repeat Domain 62	Protein Coding	O43379	44	GC19P036054	0.621566	https://www.genecards.org/cgi-bin/carddisp.pl?gene=WDR62
AMY1C	Amylase Alpha 1C	Protein Coding	P0DTE8	33	GC01P103745	0.618289	https://www.genecards.org/cgi-bin/carddisp.pl?gene=AMY1C
AMY1B	Amylase Alpha 1B	Protein Coding	P0DTE7	30	GC01M103687	0.618289	https://www.genecards.org/cgi-bin/carddisp.pl?gene=AMY1B
RANBP1	RAN Binding Protein 1	Protein Coding	P43487	47	GC22P020115	0.614004	https://www.genecards.org/cgi-bin/carddisp.pl?gene=RANBP1
SSR2	Signal Sequence Receptor Subunit 2	Protein Coding	P43308	44	GC01M156009	0.614004	https://www.genecards.org/cgi-bin/carddisp.pl?gene=SSR2
DGCR6L	DiGeorge Syndrome Critical Region Gene 6 Like	Protein Coding	Q9BY27	37	GC22M020314	0.614004	https://www.genecards.org/cgi-bin/carddisp.pl?gene=DGCR6L

DGCR6	DiGeorge Syndrome Critical Region Gene 6	Protein Coding	Q14129	35	GC22P018906	0.614004	https://www.genecards.org/cgi-bin/carddisp.pl?gene=DGCR6
FAM3B	FAM3 Metabolism Regulating Signaling Molecule B	Protein Coding	P58499	42	GC21P041304	0.610359	https://www.genecards.org/cgi-bin/carddisp.pl?gene=FAM3B
SYK	Spleen Associated Tyrosine Kinase	Protein Coding	P43405	54	GC09P095977	0.609792	https://www.genecards.org/cgi-bin/carddisp.pl?gene=SYK
TPI1	Triosephosphate Isomerase 1	Protein Coding	P60174	51	GC12P006867	0.609104	https://www.genecards.org/cgi-bin/carddisp.pl?gene=TPI1
MEFV	MEFV Innate Immunity Regulator, Pyrin	Protein Coding	O15553	48	GC16M013462	0.608734	https://www.genecards.org/cgi-bin/carddisp.pl?gene=MEFV
MTHFD1L	Methylenetetrahydrofolate Dehydrogenase (NADP+ Dependent) 1 Like	Protein Coding	Q6UB35	45	GC06P150865	0.608734	https://www.genecards.org/cgi-bin/carddisp.pl?gene=MTHFD1L
TRIM37	Tripartite Motif Containing 37	Protein Coding	O94972	47	GC17M065717	0.608479	https://www.genecards.org/cgi-bin/carddisp.pl?gene=TRIM37
MMAA	Metabolism Of Cobalamin Associated A	Protein Coding	Q8IVH4	43	GC04P145623	0.608479	https://www.genecards.org/cgi-bin/carddisp.pl?gene=MMAA
L1CAM-AS1	L1CAM Antisense RNA 1	RNA Gene		15	GC0XP153894	0.608479	https://www.genecards.org/cgi-bin/carddisp.pl?gene=L1CAM-AS1
RHOD	Ras Homolog Family Member D	Protein Coding	O00212	41	GC11P079973	0.607861	https://www.genecards.org/cgi-bin/carddisp.pl?gene=RHOD
YWHAE	Tyrosine 3-Monooxygenase/Tryptophan 5-Monooxygenase Activation Protein Epsilon	Protein Coding	P62258	53	GC17M014055	0.607341	https://www.genecards.org/cgi-bin/carddisp.pl?gene=YWHAE
MYOG	Myogenin	Protein Coding	P15173	44	GC01M203083	0.607341	https://www.genecards.org/cgi-bin/carddisp.pl?gene=MYOG

MT3	Metallothionein 3	Protein Coding	P25713	43	GC16P056589	0.607341	https://www.genecards.org/cgi-bin/carddisp.pl?gene=MT3
HCFC1	Host Cell Factor C1	Protein Coding	P51610	51	GC0XM153947	0.60617	https://www.genecards.org/cgi-bin/carddisp.pl?gene=HCFC1
AQP3	Aquaporin 3 (Gill Blood Group)	Protein Coding	Q92482	49	GC09M033431	0.604561	https://www.genecards.org/cgi-bin/carddisp.pl?gene=AQP3
THSD4	Thrombospondin Type 1 Domain Containing 4	Protein Coding	Q6ZMP0	43	GC15P071096	0.602653	https://www.genecards.org/cgi-bin/carddisp.pl?gene=THSD4
EEFSEC	Eukaryotic Elongation Factor, Selenocysteine-TRNA Specific	Protein Coding	P57772	43	GC03P128153	0.601346	https://www.genecards.org/cgi-bin/carddisp.pl?gene=EEFSEC
SLC22A3	Solute Carrier Family 22 Member 3	Protein Coding	O75751	48	GC06P160348	0.60106	https://www.genecards.org/cgi-bin/carddisp.pl?gene=SLC22A3
GDF2	Growth Differentiation Factor 2	Protein Coding	Q9UK05	48	GC10P047322	0.600209	https://www.genecards.org/cgi-bin/carddisp.pl?gene=GDF2
TGFBI	Transforming Growth Factor Beta Induced	Protein Coding	Q15582	50	GC05P136027	0.599589	https://www.genecards.org/cgi-bin/carddisp.pl?gene=TGFBI
C5AR1	Complement C5a Receptor 1	Protein Coding	P21730	49	GC19P047290	0.598216	https://www.genecards.org/cgi-bin/carddisp.pl?gene=C5AR1
BMP5	Bone Morphogenetic Protein 5	Protein Coding	P22003	47	GC06M055753	0.5972	https://www.genecards.org/cgi-bin/carddisp.pl?gene=BMP5
XCR1	X-C Motif Chemokine Receptor 1	Protein Coding	P46094	41	GC03M046016	0.595958	https://www.genecards.org/cgi-bin/carddisp.pl?gene=XCR1
MICB	MHC Class I Polypeptide-Related Sequence B	Protein Coding	Q29980	45	GC06P111991	0.594701	https://www.genecards.org/cgi-bin/carddisp.pl?gene=MICB

BACE2	Beta-Secretase 2	Protein Coding	Q9Y5Z0	46	GC21P041168	0.592749	https://www.genecards.org/cgi-bin/carddisp.pl?gene=BACE2
DDX4	DEAD-Box Helicase 4	Protein Coding	Q9NQI0	45	GC05P055738	0.592749	https://www.genecards.org/cgi-bin/carddisp.pl?gene=DDX4
ESPL1	Extra Spindle Pole Bodies Like 1, Separase	Protein Coding	Q14674	45	GC12P053268	0.592749	https://www.genecards.org/cgi-bin/carddisp.pl?gene=ESPL1
PTN	Pleiotrophin	Protein Coding	P21246	45	GC07M137227	0.592749	https://www.genecards.org/cgi-bin/carddisp.pl?gene=PTN
UPF3B	UPF3B Regulator Of Nonsense Mediated MRNA Decay	Protein Coding	Q9BZI7	45	GC0XM119805	0.592749	https://www.genecards.org/cgi-bin/carddisp.pl?gene=UPF3B
CD177	CD177 Molecule	Protein Coding	Q8N6Q3	43	GC19P043353	0.588831	https://www.genecards.org/cgi-bin/carddisp.pl?gene=CD177
SLC11A1	Solute Carrier Family 11 Member 1	Protein Coding	P49279	50	GC02P218382	0.586969	https://www.genecards.org/cgi-bin/carddisp.pl?gene=SLC11A1
GCLC	Glutamate-Cysteine Ligase Catalytic Subunit	Protein Coding	P48506	49	GC06M053497	0.586969	https://www.genecards.org/cgi-bin/carddisp.pl?gene=GCLC
CBX2	Chromobox 2	Protein Coding	Q14781	46	GC17P087390	0.586969	https://www.genecards.org/cgi-bin/carddisp.pl?gene=CBX2
KLK7	Kallikrein Related Peptidase 7	Protein Coding	P49862	46	GC19M082963	0.586532	https://www.genecards.org/cgi-bin/carddisp.pl?gene=KLK7
CD248	CD248 Molecule	Protein Coding	Q9HCU0	44	GC11M066314	0.585796	https://www.genecards.org/cgi-bin/carddisp.pl?gene=CD248
AP008439		RNA Gene		3	GC01P006260	0.585075	https://www.genecards.org/cgi-bin/carddisp.pl?gene=AP008439

PPT1	Palmitoyl-Protein Thioesterase 1	Protein Coding	P50897	51	GC01M040144	0.584648	https://www.genecards.org/cgi-bin/carddisp.pl?gene=PPT1
GALC	Galactosylceramidase	Protein Coding	P54803	49	GC14M087837	0.584648	https://www.genecards.org/cgi-bin/carddisp.pl?gene=GALC
LOC112806037	Sharpr-MPRA Regulatory Region 3720	Functional Element		4	GC02P127697	0.584648	https://www.genecards.org/cgi-bin/carddisp.pl?gene=LOC112806037
ITGA5	Integrin Subunit Alpha 5	Protein Coding	P08648	52	GC12M055371	0.584523	https://www.genecards.org/cgi-bin/carddisp.pl?gene=ITGA5
ASCL1	Achaete-Scute Family BHLH Transcription Factor 1	Protein Coding	P50553	46	GC12P102957	0.582049	https://www.genecards.org/cgi-bin/carddisp.pl?gene=ASCL1
CCNG1	Cyclin G1	Protein Coding	P51959	45	GC05P163441	0.579653	https://www.genecards.org/cgi-bin/carddisp.pl?gene=CCNG1
CORO1A	Coronin 1A	Protein Coding	P31146	48	GC16P054135	0.579592	https://www.genecards.org/cgi-bin/carddisp.pl?gene=CORO1A
PREPL	Prolyl Endopeptidase Like	Protein Coding	Q4J6C6	46	GC02M044316	0.578955	https://www.genecards.org/cgi-bin/carddisp.pl?gene=PREPL
FUT1	Fucosyltransferase 1 (H Blood Group)	Protein Coding	P19526	44	GC19M048748	0.578924	https://www.genecards.org/cgi-bin/carddisp.pl?gene=FUT1
RNR1	RNA, Ribosomal 45S Cluster 1	RNA Gene		9	GC13U990028	0.578924	https://www.genecards.org/cgi-bin/carddisp.pl?gene=RNR1
ATXN2L	Ataxin 2 Like	Protein Coding	Q8WWM7	44	GC16P053986	0.576667	https://www.genecards.org/cgi-bin/carddisp.pl?gene=ATXN2L
UHRF1	Ubiquitin Like With PHD And Ring Finger Domains 1	Protein Coding	Q96T88	44	GC19P008882	0.576667	https://www.genecards.org/cgi-bin/carddisp.pl?gene=UHRF1

EIF2AK2	Eukaryotic Translation Initiation Factor 2 Alpha Kinase 2	Protein Coding	P19525	54	GC02M037099	0.575768	https://www.genecards.org/cgi-bin/carddisp.pl?gene=EIF2AK2
VCP	Valosin Containing Protein	Protein Coding	P55072	53	GC09M035056	0.575768	https://www.genecards.org/cgi-bin/carddisp.pl?gene=VCP
DDX3X	DEAD-Box Helicase 3 X-Linked	Protein Coding	O00571	52	GC0XP041333	0.569408	https://www.genecards.org/cgi-bin/carddisp.pl?gene=DDX3X
PI4KA	Phosphatidylinositol 4-Kinase Alpha	Protein Coding	P42356	52	GC22M020707	0.568264	https://www.genecards.org/cgi-bin/carddisp.pl?gene=PI4KA
SLC3A1	Solute Carrier Family 3 Member 1	Protein Coding	Q07837	52	GC02P044275	0.568264	https://www.genecards.org/cgi-bin/carddisp.pl?gene=SLC3A1
ABCC6	ATP Binding Cassette Subfamily C Member 6	Protein Coding	O95255	51	GC16M016809	0.568264	https://www.genecards.org/cgi-bin/carddisp.pl?gene=ABCC6
PPP2R5D	Protein Phosphatase 2 Regulatory Subunit B'Delta	Protein Coding	Q14738	50	GC06P112220	0.568264	https://www.genecards.org/cgi-bin/carddisp.pl?gene=PPP2R5D
DCX	Doublecortin	Protein Coding	O43602	48	GC0XM111293	0.568264	https://www.genecards.org/cgi-bin/carddisp.pl?gene=DCX
PLXNA3	Plexin A3	Protein Coding	P51805	43	GC0XP154458	0.568264	https://www.genecards.org/cgi-bin/carddisp.pl?gene=PLXNA3
MAGOH	Mago Homolog, Exon Junction Complex Subunit	Protein Coding	P61326	41	GC01M053226	0.568264	https://www.genecards.org/cgi-bin/carddisp.pl?gene=MAGOH
USP26	Ubiquitin Specific Peptidase 26	Protein Coding	Q9BXU7	40	GC0XM133024	0.568264	https://www.genecards.org/cgi-bin/carddisp.pl?gene=USP26
MIR1249	MicroRNA 1249	RNA Gene		19	GC22M045200	0.568264	https://www.genecards.org/cgi-bin/carddisp.pl?gene=MIR1249

DLL4	Delta Like Canonical Notch Ligand 4	Protein Coding	Q9NR61	51	GC15P040929	0.567397	https://www.genecards.org/cgi-bin/carddisp.pl?gene=DLL4
ARRB2	Arrestin Beta 2	Protein Coding	P32121	49	GC17P013851	0.567397	https://www.genecards.org/cgi-bin/carddisp.pl?gene=ARRB2
ITGAL	Integrin Subunit Alpha L	Protein Coding	P20701	51	GC16P030472	0.56691	https://www.genecards.org/cgi-bin/carddisp.pl?gene=ITGAL
PTPRO	Protein Tyrosine Phosphatase Receptor Type O	Protein Coding	Q16827	51	GC12P030780	0.56691	https://www.genecards.org/cgi-bin/carddisp.pl?gene=PTPRO
ALDH9A1	Aldehyde Dehydrogenase 9 Family Member A1	Protein Coding	P49189	46	GC01M165685	0.56691	https://www.genecards.org/cgi-bin/carddisp.pl?gene=ALDH9A1
GLIS2	GLIS Family Zinc Finger 2	Protein Coding	Q9BZE0	45	GC16P052513	0.565517	https://www.genecards.org/cgi-bin/carddisp.pl?gene=GLIS2
DCAF1	DDB1 And CUL4 Associated Factor 1	Protein Coding	Q9Y4B6	42	GC03M051395	0.565517	https://www.genecards.org/cgi-bin/carddisp.pl?gene=DCAF1
ERCC1	ERCC Excision Repair 1, Endonuclease Non-Catalytic Subunit	Protein Coding	P07992	50	GC19M082704	0.564899	https://www.genecards.org/cgi-bin/carddisp.pl?gene=ERCC1
MUTYH	MutY DNA Glycosylase	Protein Coding	Q9UIF7	49	GC01M045329	0.563553	https://www.genecards.org/cgi-bin/carddisp.pl?gene=MUTYH
GAA	Alpha Glucosidase	Protein Coding	P10253	53	GC17P080101	0.562971	https://www.genecards.org/cgi-bin/carddisp.pl?gene=GAA
MIR7-3	MicroRNA 7-3	RNA Gene		19	GC19P004770	0.561599	https://www.genecards.org/cgi-bin/carddisp.pl?gene=MIR7-3
MIR7-2	MicroRNA 7-2	RNA Gene		18	GC15P088611	0.561599	https://www.genecards.org/cgi-bin/carddisp.pl?gene=MIR7-2

MAP1B	Microtubule Associated Protein 1B	Protein Coding	P46821	48	GC05P072107	0.561112	https://www.genecards.org/cgi-bin/carddisp.pl?gene=MAP1B
ALOX15B	Arachidonate 15-Lipoxygenase Type B	Protein Coding	O15296	45	GC17P008039	0.561112	https://www.genecards.org/cgi-bin/carddisp.pl?gene=ALOX15B
GNAL	G Protein Subunit Alpha L	Protein Coding	P38405	47	GC18P011689	0.560706	https://www.genecards.org/cgi-bin/carddisp.pl?gene=GNAL
SCN9A	Sodium Voltage-Gated Channel Alpha Subunit 9	Protein Coding	Q15858	51	GC02M166195	0.559236	https://www.genecards.org/cgi-bin/carddisp.pl?gene=SCN9A
RPS24	Ribosomal Protein S24	Protein Coding	P62847	47	GC10P078033	0.559236	https://www.genecards.org/cgi-bin/carddisp.pl?gene=RPS24
SRR	Serine Racemase	Protein Coding	Q9GZT4	46	GC17P002303	0.559236	https://www.genecards.org/cgi-bin/carddisp.pl?gene=SRR
SUGCT	Succinyl-CoA:Glutarate-CoA Transferase	Protein Coding	Q9HAC7	44	GC07P040830	0.558741	https://www.genecards.org/cgi-bin/carddisp.pl?gene=SUGCT
TRIB2	Tribbles Pseudokinase 2	Protein Coding	Q92519	42	GC02P012721	0.558385	https://www.genecards.org/cgi-bin/carddisp.pl?gene=TRIB2
PRDX2	Peroxiredoxin 2	Protein Coding	P32119	50	GC19M012796	0.555254	https://www.genecards.org/cgi-bin/carddisp.pl?gene=PRDX2
SELPLG	Selectin P Ligand	Protein Coding	Q14242	48	GC12M108621	0.555254	https://www.genecards.org/cgi-bin/carddisp.pl?gene=SELPLG
SLC18A2	Solute Carrier Family 18 Member A2	Protein Coding	Q05940	51	GC10P117241	0.55444	https://www.genecards.org/cgi-bin/carddisp.pl?gene=SLC18A2
SLC6A1	Solute Carrier Family 6 Member 1	Protein Coding	P30531	51	GC03P018372	0.55444	https://www.genecards.org/cgi-bin/carddisp.pl?gene=SLC6A1

ATP5F1A	ATP Synthase F1 Subunit Alpha	Protein Coding	P25705	50	GC18M046081	0.55444	https://www.genecards.org/cgi-bin/carddisp.pl?gene=ATP5F1A
CTBP1	C-Terminal Binding Protein 1	Protein Coding	Q13363	51	GC04M001211	0.55283	https://www.genecards.org/cgi-bin/carddisp.pl?gene=CTBP1
TRIM33	Tripartite Motif Containing 33	Protein Coding	Q9UPN9	48	GC01M114392	0.55283	https://www.genecards.org/cgi-bin/carddisp.pl?gene=TRIM33
AGO1	Argonaute RISC Component 1	Protein Coding	Q9UL18	45	GC01P035869	0.55283	https://www.genecards.org/cgi-bin/carddisp.pl?gene=AGO1
RNY1	RNA, Ro60-Associated Y1	RNA Gene		17	GC07M148987	0.55283	https://www.genecards.org/cgi-bin/carddisp.pl?gene=RNY1
OTOA	Otoancorin	Protein Coding	Q7RTW8	42	GC16P053108	0.551557	https://www.genecards.org/cgi-bin/carddisp.pl?gene=OTOA
PGAP3	Post-GPI Attachment To Proteins Phospholipase 3	Protein Coding	Q96FM1	44	GC17M064054	0.549956	https://www.genecards.org/cgi-bin/carddisp.pl?gene=PGAP3
PLCG2	Phospholipase C Gamma 2	Protein Coding	P16885	55	GC16P081773	0.547897	https://www.genecards.org/cgi-bin/carddisp.pl?gene=PLCG2
PHOX2A	Paired Like Homeobox 2A	Protein Coding	O14813	47	GC11M072239	0.547897	https://www.genecards.org/cgi-bin/carddisp.pl?gene=PHOX2A
TCAP	Titin-Cap	Protein Coding	O15273	46	GC17P086310	0.547897	https://www.genecards.org/cgi-bin/carddisp.pl?gene=TCAP
SYNGAP1	Synaptic Ras GTPase Activating Protein 1	Protein Coding	Q96PV0	51	GC06P112091	0.545639	https://www.genecards.org/cgi-bin/carddisp.pl?gene=SYNGAP1
SRGAP1	SLIT-ROBO Rho GTPase Activating Protein 1	Protein Coding	Q7Z6B7	47	GC12P063844	0.545639	https://www.genecards.org/cgi-bin/carddisp.pl?gene=SRGAP1

FREM1	FRAS1 Related Extracellular Matrix 1	Protein Coding	Q5H8C1	46	GC09M014734	0.545639	https://www.genecards.org/cgi-bin/carddisp.pl?gene=FREM1
STRA6	Signaling Receptor And Transporter Of Retinol STRA6	Protein Coding	Q9BX79	46	GC15M074179	0.545413	https://www.genecards.org/cgi-bin/carddisp.pl?gene=STRA6
GGCX	Gamma-Glutamyl Carboxylase	Protein Coding	P38435	52	GC02M085544	0.540473	https://www.genecards.org/cgi-bin/carddisp.pl?gene=GGCX
KCNA2	Potassium Voltage-Gated Channel Subfamily A Member 2	Protein Coding	P16389	50	GC01M110519	0.540473	https://www.genecards.org/cgi-bin/carddisp.pl?gene=KCNA2
GJB6	Gap Junction Protein Beta 6	Protein Coding	O95452	46	GC13M020221	0.540473	https://www.genecards.org/cgi-bin/carddisp.pl?gene=GJB6
HMGN1	High Mobility Group Nucleosome Binding Domain 1	Protein Coding	P05114	45	GC21M039342	0.540473	https://www.genecards.org/cgi-bin/carddisp.pl?gene=HMGN1
MX1	MX Dynamin Like GTPase 1	Protein Coding	P20591	45	GC21P041420	0.540473	https://www.genecards.org/cgi-bin/carddisp.pl?gene=MX1
PSMG1	Proteasome Assembly Chaperone 1	Protein Coding	O95456	42	GC21M039174	0.540473	https://www.genecards.org/cgi-bin/carddisp.pl?gene=PSMG1
MAGOHB	Mago Homolog B, Exon Junction Complex Subunit	Protein Coding	Q96A72	40	GC12M025980	0.540473	https://www.genecards.org/cgi-bin/carddisp.pl?gene=MAGOHB
RPS4Y1	Ribosomal Protein S4 Y-Linked 1	Protein Coding	P22090	36	GC0YP002841	0.540473	https://www.genecards.org/cgi-bin/carddisp.pl?gene=RPS4Y1
ELOA3P	Elongin A3, Pseudogene	Pseudogene		20	GC18M047173	0.540473	https://www.genecards.org/cgi-bin/carddisp.pl?gene=ELOA3P
SAE1	SUMO1 Activating Enzyme Subunit 1	Protein Coding	Q9UBE0	48	GC19P089811	0.537289	https://www.genecards.org/cgi-bin/carddisp.pl?gene=SAE1

USP4	Ubiquitin Specific Peptidase 4	Protein Coding	Q13107	47	GC03M049277	0.536443	https://www.genecards.org/cgi-bin/carddisp.pl?gene=USP4
UGT2B17	UDP Glucuronosyltransferase Family 2 Member B17	Protein Coding	O75795	45	GC04M068537	0.536443	https://www.genecards.org/cgi-bin/carddisp.pl?gene=UGT2B17
MAP3K1	Mitogen-Activated Protein Kinase Kinase Kinase 1	Protein Coding	Q13233	53	GC05P056815	0.535305	https://www.genecards.org/cgi-bin/carddisp.pl?gene=MAP3K1
PPP1CA	Protein Phosphatase 1 Catalytic Subunit Alpha	Protein Coding	P62136	51	GC11M113648	0.535305	https://www.genecards.org/cgi-bin/carddisp.pl?gene=PPP1CA
RACK1	Receptor For Activated C Kinase 1	Protein Coding	P63244	48	GC05M182318	0.535305	https://www.genecards.org/cgi-bin/carddisp.pl?gene=RACK1
APCS	Amyloid P Component, Serum	Protein Coding	P02743	47	GC01P159587	0.535305	https://www.genecards.org/cgi-bin/carddisp.pl?gene=APCS
MFAP3	Microfibril Associated Protein 3	Protein Coding	P55082	42	GC05P154014	0.535305	https://www.genecards.org/cgi-bin/carddisp.pl?gene=MFAP3
PTGDR2	Prostaglandin D2 Receptor 2	Protein Coding	Q9Y5Y4	47	GC11M060850	0.533944	https://www.genecards.org/cgi-bin/carddisp.pl?gene=PTGDR2
MIR372	MicroRNA 372	RNA Gene		16	GC19P090149	0.533944	https://www.genecards.org/cgi-bin/carddisp.pl?gene=MIR372
FUS	FUS RNA Binding Protein	Protein Coding	P35637	48	GC16P031180	0.533705	https://www.genecards.org/cgi-bin/carddisp.pl?gene=FUS
LINC02860	Long Intergenic Non-Protein Coding RNA 2860	RNA Gene		20	GC07P026819	0.533705	https://www.genecards.org/cgi-bin/carddisp.pl?gene=LINC02860
MIR3909	MicroRNA 3909	RNA Gene		16	GC22P035335	0.533705	https://www.genecards.org/cgi-bin/carddisp.pl?gene=MIR3909

HMMR	Hyaluronan Mediated Motility Receptor	Protein Coding	O75330	48	GC05P163480	0.532806	https://www.genecards.org/cgi-bin/carddisp.pl?gene=HMMR
JUND	JunD Proto-Oncogene, AP-1 Transcription Factor Subunit	Protein Coding	P17535	45	GC19M018279	0.532806	https://www.genecards.org/cgi-bin/carddisp.pl?gene=JUND
HMOX2	Heme Oxygenase 2	Protein Coding	P30519	49	GC16P004474	0.532194	https://www.genecards.org/cgi-bin/carddisp.pl?gene=HMOX2
ADAMTS5	ADAM Metallopeptidase With Thrombospondin Type 1 Motif 5	Protein Coding	Q9UNA0	48	GC21M026918	0.529933	https://www.genecards.org/cgi-bin/carddisp.pl?gene=ADAMTS5
ATP2B1	ATPase Plasma Membrane Ca2+ Transporting 1	Protein Coding	P20020	48	GC12M089588	0.526648	https://www.genecards.org/cgi-bin/carddisp.pl?gene=ATP2B1
AF465954-001		RNA Gene		4	GC05M094569	0.526648	https://www.genecards.org/cgi-bin/carddisp.pl?gene=AF465954-001
AB464896		RNA Gene		3	GC05M135211	0.526648	https://www.genecards.org/cgi-bin/carddisp.pl?gene=AB464896
PHEX	Phosphate Regulating Endopeptidase X-Linked	Protein Coding	P78562	47	GC0XP022032	0.525775	https://www.genecards.org/cgi-bin/carddisp.pl?gene=PHEX
CDC42EP3	CDC42 Effector Protein 3	Protein Coding	Q9UKI2	44	GC02M037641	0.525775	https://www.genecards.org/cgi-bin/carddisp.pl?gene=CDC42EP3
CCL24	C-C Motif Chemokine Ligand 24	Protein Coding	O00175	42	GC07M078061	0.525775	https://www.genecards.org/cgi-bin/carddisp.pl?gene=CCL24
MYDGF	Myeloid Derived Growth Factor	Protein Coding	Q969H8	40	GC19M004641	0.525775	https://www.genecards.org/cgi-bin/carddisp.pl?gene=MYDGF
ASB4	Ankyrin Repeat And SOCS Box Containing 4	Protein Coding	Q9Y574	39	GC07P095517	0.525775	https://www.genecards.org/cgi-bin/carddisp.pl?gene=ASB4

RPL11	Ribosomal Protein L11	Protein Coding	P62913	52	GC01P023691	0.512615	https://www.genecards.org/cgi-bin/carddisp.pl?gene=RPL11
MYO6	Myosin VI	Protein Coding	Q9UM54	48	GC06P075749	0.512615	https://www.genecards.org/cgi-bin/carddisp.pl?gene=MYO6
SLC5A6	Solute Carrier Family 5 Member 6	Protein Coding	Q9Y289	48	GC02M027201	0.512615	https://www.genecards.org/cgi-bin/carddisp.pl?gene=SLC5A6
FCN2	Ficolin 2	Protein Coding	Q15485	47	GC09P134864	0.512615	https://www.genecards.org/cgi-bin/carddisp.pl?gene=FCN2
RXFP2	Relaxin Family Peptide Receptor 2	Protein Coding	Q8WXD0	46	GC13P031739	0.512615	https://www.genecards.org/cgi-bin/carddisp.pl?gene=RXFP2
RBP1	Retinol Binding Protein 1	Protein Coding	P09455	45	GC03M139517	0.512615	https://www.genecards.org/cgi-bin/carddisp.pl?gene=RBP1
LTBP1	Latent Transforming Growth Factor Beta Binding Protein 1	Protein Coding	Q14766	49	GC02P032946	0.509868	https://www.genecards.org/cgi-bin/carddisp.pl?gene=LTBP1
ARHGEF11	Rho Guanine Nucleotide Exchange Factor 11	Protein Coding	O15085	46	GC01M156934	0.509868	https://www.genecards.org/cgi-bin/carddisp.pl?gene=ARHGEF11
DUSP5	Dual Specificity Phosphatase 5	Protein Coding	Q16690	46	GC10P110497	0.509868	https://www.genecards.org/cgi-bin/carddisp.pl?gene=DUSP5
LCORL	Ligand Dependent Nuclear Receptor Corepressor Like	Protein Coding	Q8N3X6	41	GC04M017844	0.509868	https://www.genecards.org/cgi-bin/carddisp.pl?gene=LCORL
QRFPR	Pyroglutamylated RFamide Peptide Receptor	Protein Coding	Q96P65	40	GC04M121328	0.509868	https://www.genecards.org/cgi-bin/carddisp.pl?gene=QRFPR
SURF6	Surfeit 6	Protein Coding	O75683	39	GC09M133331	0.509868	https://www.genecards.org/cgi-bin/carddisp.pl?gene=SURF6

OPHN1	Oligophrenin 1	Protein Coding	O60890	47	GC0XM067949	0.509202	https://www.genecards.org/cgi-bin/carddisp.pl?gene=OPHN1
EPHA2	EPH Receptor A2	Protein Coding	P29317	57	GC01M016124	0.508595	https://www.genecards.org/cgi-bin/carddisp.pl?gene=EPHA2
ILK	Integrin Linked Kinase	Protein Coding	Q13418	48	GC11P006604	0.508595	https://www.genecards.org/cgi-bin/carddisp.pl?gene=ILK
MIR508	MicroRNA 508	RNA Gene		17	GC0XM147236	0.508595	https://www.genecards.org/cgi-bin/carddisp.pl?gene=MIR508
GRIP1	Glutamate Receptor Interacting Protein 1	Protein Coding	Q9Y3R0	47	GC12M066347	0.508132	https://www.genecards.org/cgi-bin/carddisp.pl?gene=GRIP1
ALG9	ALG9 Alpha-1,2-Mannosyltransferase	Protein Coding	Q9H6U8	46	GC11M114462	0.508132	https://www.genecards.org/cgi-bin/carddisp.pl?gene=ALG9
VPS33B	VPS33B Late Endosome And Lysosome Associated	Protein Coding	Q9H267	46	GC15M090998	0.508132	https://www.genecards.org/cgi-bin/carddisp.pl?gene=VPS33B
TCTN3	Tectonic Family Member 3	Protein Coding	Q6NUS6	45	GC10M095663	0.508132	https://www.genecards.org/cgi-bin/carddisp.pl?gene=TCTN3
B9D2	B9 Domain Containing 2	Protein Coding	Q9BPU9	44	GC19M041354	0.508132	https://www.genecards.org/cgi-bin/carddisp.pl?gene=B9D2
NDUFB7	NADH:Ubiquinone Oxidoreductase Subunit B7	Protein Coding	P17568	44	GC19M014566	0.508132	https://www.genecards.org/cgi-bin/carddisp.pl?gene=NDUFB7
PIGL	Phosphatidylinositol Glycan Anchor Biosynthesis Class L	Protein Coding	Q9Y2B2	43	GC17P016217	0.508132	https://www.genecards.org/cgi-bin/carddisp.pl?gene=PIGL
FREM2	FRAS1 Related Extracellular Matrix 2	Protein Coding	Q5SZK8	42	GC13P038687	0.508132	https://www.genecards.org/cgi-bin/carddisp.pl?gene=FREM2

TMEM237	Transmembrane Protein 237	Protein Coding	Q96Q45	41	GC02M201620	0.508132	https://www.genecards.org/cgi-bin/carddisp.pl?gene=TMEM237
FRA10AC1	FRA10A Associated CGG Repeat 1	Protein Coding	Q70Z53	40	GC10M093667	0.508132	https://www.genecards.org/cgi-bin/carddisp.pl?gene=FRA10AC1
POLR3A	RNA Polymerase III Subunit A	Protein Coding	O14802	49	GC10M078926	0.507507	https://www.genecards.org/cgi-bin/carddisp.pl?gene=POLR3A
PDCD10	Programmed Cell Death 10	Protein Coding	Q9BUL8	46	GC03M167683	0.507507	https://www.genecards.org/cgi-bin/carddisp.pl?gene=PDCD10
SLC24A3	Solute Carrier Family 24 Member 3	Protein Coding	Q9HC58	43	GC20P019212	0.507507	https://www.genecards.org/cgi-bin/carddisp.pl?gene=SLC24A3
BOLL	Boule Homolog, RNA Binding Protein	Protein Coding	Q8N9W6	41	GC02M197726	0.507507	https://www.genecards.org/cgi-bin/carddisp.pl?gene=BOLL
NANOS2	Nanos C2HC-Type Zinc Finger 2	Protein Coding	P60321	38	GC19M045913	0.507507	https://www.genecards.org/cgi-bin/carddisp.pl?gene=NANOS2
RAD21L1	RAD21 Cohesin Complex Component Like 1	Protein Coding	Q9H4I0	33	GC20P001226	0.507507	https://www.genecards.org/cgi-bin/carddisp.pl?gene=RAD21L1
MIR449B	MicroRNA 449b	RNA Gene		19	GC05M055172	0.507507	https://www.genecards.org/cgi-bin/carddisp.pl?gene=MIR449B
MFF-DT	MFF Divergent Transcript	RNA Gene		15	GC02M227223	0.507507	https://www.genecards.org/cgi-bin/carddisp.pl?gene=MFF-DT
LOC101928994	Uncharacterized LOC101928994	RNA Gene		13	GC10M069333	0.507507	https://www.genecards.org/cgi-bin/carddisp.pl?gene=LOC101928994
MIR548AM	MicroRNA 548am	RNA Gene		12	GC0XM016627	0.507507	https://www.genecards.org/cgi-bin/carddisp.pl?gene=MIR548AM

ITK	IL2 Inducible T Cell Kinase	Protein Coding	Q08881	55	GC05P157158	0.506073	https://www.genecards.org/cgi-bin/carddisp.pl?gene=ITK
FOSB	FosB Proto-Oncogene, AP-1 Transcription Factor Subunit	Protein Coding	P53539	47	GC19P045467	0.503588	https://www.genecards.org/cgi-bin/carddisp.pl?gene=FOSB
ACSL3	Acyl-CoA Synthetase Long Chain Family Member 3	Protein Coding	O95573	45	GC02P222860	0.503588	https://www.genecards.org/cgi-bin/carddisp.pl?gene=ACSL3
LINC01082	Long Intergenic Non-Protein Coding RNA 1082	RNA Gene		17	GC16P086065	0.502878	https://www.genecards.org/cgi-bin/carddisp.pl?gene=LINC01082
GALT	Galactose-1-Phosphate Uridylyltransferase	Protein Coding	P07902	50	GC09P053179	0.496228	https://www.genecards.org/cgi-bin/carddisp.pl?gene=GALT
HSD17B4	Hydroxysteroid 17-Beta Dehydrogenase 4	Protein Coding	P51659	50	GC05P119452	0.496228	https://www.genecards.org/cgi-bin/carddisp.pl?gene=HSD17B4
MFHAS1	Multifunctional ROCO Family Signaling Regulator 1	Protein Coding	Q9Y4C4	40	GC08M008783	0.496228	https://www.genecards.org/cgi-bin/carddisp.pl?gene=MFHAS1
APRT	Adenine Phosphoribosyltransferase	Protein Coding	P07741	51	GC16M088810	0.493729	https://www.genecards.org/cgi-bin/carddisp.pl?gene=APRT
TOR1A	Torsin Family 1 Member A	Protein Coding	O14656	50	GC09M129812	0.493729	https://www.genecards.org/cgi-bin/carddisp.pl?gene=TOR1A
ST6GAL1	ST6 Beta-Galactoside Alpha-2,6-Sialyltransferase 1	Protein Coding	P15907	48	GC03P186930	0.493729	https://www.genecards.org/cgi-bin/carddisp.pl?gene=ST6GAL1
CYP26B1	Cytochrome P450 Family 26 Subfamily B Member 1	Protein Coding	Q9NR63	47	GC02M072129	0.493729	https://www.genecards.org/cgi-bin/carddisp.pl?gene=CYP26B1
CYP26C1	Cytochrome P450 Family 26 Subfamily C Member 1	Protein Coding	Q6V0L0	46	GC10P093060	0.493729	https://www.genecards.org/cgi-bin/carddisp.pl?gene=CYP26C1

SERPINB3	Serpin Family B Member 3	Protein Coding	P29508	45	GC18M063655	0.493729	https://www.genecards.org/cgi-bin/carddisp.pl?gene=SERPINB3
HTR7	5-Hydroxytryptamine Receptor 7	Protein Coding	P34969	51	GC10M090740	0.493682	https://www.genecards.org/cgi-bin/carddisp.pl?gene=HTR7
RAN	RAN, Member RAS Oncogene Family	Protein Coding	P62826	48	GC12P130874	0.493682	https://www.genecards.org/cgi-bin/carddisp.pl?gene=RAN
RXFP1	Relaxin Family Peptide Receptor 1	Protein Coding	Q9HBX9	48	GC04P158315	0.493682	https://www.genecards.org/cgi-bin/carddisp.pl?gene=RXFP1
MAP1LC3A	Microtubule Associated Protein 1 Light Chain 3 Alpha	Protein Coding	Q9H492	45	GC20P034546	0.493682	https://www.genecards.org/cgi-bin/carddisp.pl?gene=MAP1LC3A
MIR124-3	MicroRNA 124-3	RNA Gene		19	GC20P065697	0.493682	https://www.genecards.org/cgi-bin/carddisp.pl?gene=MIR124-3
HLA-S	Major Histocompatibility Complex, Class I, S (Pseudogene)	Pseudogene		10	GC06M031381	0.493682	https://www.genecards.org/cgi-bin/carddisp.pl?gene=HLA-S
ACVRL1	Activin A Receptor Like Type 1	Protein Coding	P37023	54	GC12P051906	0.493481	https://www.genecards.org/cgi-bin/carddisp.pl?gene=ACVRL1
RPL4	Ribosomal Protein L4	Protein Coding	P36578	46	GC15M066498	0.493481	https://www.genecards.org/cgi-bin/carddisp.pl?gene=RPL4
PSMD8	Proteasome 26S Subunit, Non-ATPase 8	Protein Coding	P48556	45	GC19P038374	0.493481	https://www.genecards.org/cgi-bin/carddisp.pl?gene=PSMD8
GLS	Glutaminase	Protein Coding	O94925	53	GC02P190880	0.490982	https://www.genecards.org/cgi-bin/carddisp.pl?gene=GLS
CDC25A	Cell Division Cycle 25A	Protein Coding	P30304	51	GC03M048260	0.490982	https://www.genecards.org/cgi-bin/carddisp.pl?gene=CDC25A

BCL2L11	BCL2 Like 11	Protein Coding	O43521	49	GC02P111119	0.490982	https://www.genecards.org/cgi-bin/carddisp.pl?gene=BCL2L11
PTPA	Protein Phosphatase 2 Phosphatase Activator	Protein Coding	Q15257	47	GC09P130804	0.490982	https://www.genecards.org/cgi-bin/carddisp.pl?gene=PTPA
CCL21	C-C Motif Chemokine Ligand 21	Protein Coding	O00585	45	GC09M034709	0.490982	https://www.genecards.org/cgi-bin/carddisp.pl?gene=CCL21
SPINT1	Serine Peptidase Inhibitor, Kunitz Type 1	Protein Coding	O43278	45	GC15P040844	0.490982	https://www.genecards.org/cgi-bin/carddisp.pl?gene=SPINT1
MARCKS	Myristoylated Alanine Rich Protein Kinase C Substrate	Protein Coding	P29966	44	GC06P113857	0.490982	https://www.genecards.org/cgi-bin/carddisp.pl?gene=MARCKS
TRPV5	Transient Receptor Potential Cation Channel Subfamily V Member 5	Protein Coding	Q9NQA5	48	GC07M142908	0.487066	https://www.genecards.org/cgi-bin/carddisp.pl?gene=TRPV5
RPL5	Ribosomal Protein L5	Protein Coding	P46777	52	GC01P092832	0.484824	https://www.genecards.org/cgi-bin/carddisp.pl?gene=RPL5
SCN2A	Sodium Voltage-Gated Channel Alpha Subunit 2	Protein Coding	Q99250	52	GC02P165194	0.484824	https://www.genecards.org/cgi-bin/carddisp.pl?gene=SCN2A
BRWD1	Bromodomain And WD Repeat Domain Containing 1	Protein Coding	Q9NSI6	43	GC21M039184	0.484824	https://www.genecards.org/cgi-bin/carddisp.pl?gene=BRWD1
PHACTR1	Phosphatase And Actin Regulator 1	Protein Coding	Q9C0D0	42	GC06P012717	0.484824	https://www.genecards.org/cgi-bin/carddisp.pl?gene=PHACTR1
ZC3HAV1	Zinc Finger CCCH-Type Containing, Antiviral 1	Protein Coding	Q7Z2W4	42	GC07M139096	0.484824	https://www.genecards.org/cgi-bin/carddisp.pl?gene=ZC3HAV1
RBL2	RB Transcriptional Corepressor Like 2	Protein Coding	Q08999	50	GC16P053433	0.482813	https://www.genecards.org/cgi-bin/carddisp.pl?gene=RBL2

CAV2	Caveolin 2	Protein Coding	P51636	46	GC07P116287	0.482813	https://www.genecards.org/cgi-bin/carddisp.pl?gene=CAV2
CCNE2	Cyclin E2	Protein Coding	O96020	45	GC08M094879	0.482813	https://www.genecards.org/cgi-bin/carddisp.pl?gene=CCNE2
NOVA2	NOVA Alternative Splicing Regulator 2	Protein Coding	Q9UNW9	42	GC19M045933	0.482813	https://www.genecards.org/cgi-bin/carddisp.pl?gene=NOVA2
ATAD5	ATPase Family AAA Domain Containing 5	Protein Coding	Q96QE3	40	GC17P085923	0.482813	https://www.genecards.org/cgi-bin/carddisp.pl?gene=ATAD5
PLAC8	Placenta Associated 8	Protein Coding	Q9NZF1	39	GC04M083090	0.482813	https://www.genecards.org/cgi-bin/carddisp.pl?gene=PLAC8
MIR3936HG	MIR3936 Host Gene	RNA Gene		16	GC05M132312	0.482813	https://www.genecards.org/cgi-bin/carddisp.pl?gene=MIR3936HG
MIR421	MicroRNA 421	RNA Gene		16	GC0XM074218	0.482813	https://www.genecards.org/cgi-bin/carddisp.pl?gene=MIR421
SNORD38B	Small Nucleolar RNA, C/D Box 38B	RNA Gene		15	GC01P045711	0.482813	https://www.genecards.org/cgi-bin/carddisp.pl?gene=SNORD38B
EBP	EBP Cholestenol Delta-Isomerase	Protein Coding	Q15125	48	GC0XP048521	0.47862	https://www.genecards.org/cgi-bin/carddisp.pl?gene=EBP
PTK2B	Protein Tyrosine Kinase 2 Beta	Protein Coding	Q14289	53	GC08P027311	0.465938	https://www.genecards.org/cgi-bin/carddisp.pl?gene=PTK2B
FUCA1	Alpha-L-Fucosidase 1	Protein Coding	P04066	52	GC01M023845	0.465938	https://www.genecards.org/cgi-bin/carddisp.pl?gene=FUCA1
MAG	Myelin Associated Glycoprotein	Protein Coding	P20916	51	GC19P035292	0.465938	https://www.genecards.org/cgi-bin/carddisp.pl?gene=MAG

WNT3	Wnt Family Member 3	Protein Coding	P56703	51	GC17M046762	0.465938	https://www.genecards.org/cgi-bin/carddisp.pl?gene=WNT3
SLC44A1	Solute Carrier Family 44 Member 1	Protein Coding	Q8WWI5	47	GC09P105244	0.465938	https://www.genecards.org/cgi-bin/carddisp.pl?gene=SLC44A1
DAAM2	Dishevelled Associated Activator Of Morphogenesis 2	Protein Coding	Q86T65	44	GC06P039792	0.465938	https://www.genecards.org/cgi-bin/carddisp.pl?gene=DAAM2
UTP6	UTP6 Small Subunit Processome Component	Protein Coding	Q9NYH9	42	GC17M031860	0.465938	https://www.genecards.org/cgi-bin/carddisp.pl?gene=UTP6
MIR1915	MicroRNA 1915	RNA Gene		17	GC10M021496	0.465938	https://www.genecards.org/cgi-bin/carddisp.pl?gene=MIR1915
DCD	Dermcidin	Protein Coding	P81605	41	GC12M054644	0.465239	https://www.genecards.org/cgi-bin/carddisp.pl?gene=DCD
ATP2A2	ATPase Sarcoplasmic/Endoplasmic Reticulum Ca2+ Transporting 2	Protein Coding	P16615	55	GC12P110280	0.464545	https://www.genecards.org/cgi-bin/carddisp.pl?gene=ATP2A2
RPA1	Replication Protein A1	Protein Coding	P27694	52	GC17P001829	0.464545	https://www.genecards.org/cgi-bin/carddisp.pl?gene=RPA1
RDX	Radixin	Protein Coding	P35241	51	GC11M109864	0.464545	https://www.genecards.org/cgi-bin/carddisp.pl?gene=RDX
EWSR1	EWS RNA Binding Protein 1	Protein Coding	Q01844	49	GC22P056383	0.464545	https://www.genecards.org/cgi-bin/carddisp.pl?gene=EWSR1
NLGN1	Neuroligin 1	Protein Coding	Q8N2Q7	49	GC03P173396	0.464545	https://www.genecards.org/cgi-bin/carddisp.pl?gene=NLGN1
NFIX	Nuclear Factor I X	Protein Coding	Q14938	48	GC19P088938	0.464545	https://www.genecards.org/cgi-bin/carddisp.pl?gene=NFIX

TNKS2	Tankyrase 2	Protein Coding	Q9H2K2	48	GC10P091798	0.464545	https://www.genecards.org/cgi-bin/carddisp.pl?gene=TNKS2
TRAP1	TNF Receptor Associated Protein 1	Protein Coding	Q12931	48	GC16M013491	0.464545	https://www.genecards.org/cgi-bin/carddisp.pl?gene=TRAP1
MCM6	Minichromosome Maintenance Complex Component 6	Protein Coding	Q14566	47	GC02M135839	0.464545	https://www.genecards.org/cgi-bin/carddisp.pl?gene=MCM6
KPNA1	Karyopherin Subunit Alpha 1	Protein Coding	P52294	46	GC03M122421	0.464545	https://www.genecards.org/cgi-bin/carddisp.pl?gene=KPNA1
NAGS	N-Acetylglutamate Synthase	Protein Coding	Q8N159	46	GC17P044004	0.464545	https://www.genecards.org/cgi-bin/carddisp.pl?gene=NAGS
SEMA3F	Semaphorin 3F	Protein Coding	Q13275	46	GC03P053946	0.464545	https://www.genecards.org/cgi-bin/carddisp.pl?gene=SEMA3F
SYCP3	Synaptonemal Complex Protein 3	Protein Coding	Q8IZU3	46	GC12M101728	0.464545	https://www.genecards.org/cgi-bin/carddisp.pl?gene=SYCP3
GUK1	Guanylate Kinase 1	Protein Coding	Q16774	45	GC01P228139	0.464545	https://www.genecards.org/cgi-bin/carddisp.pl?gene=GUK1
KMT2E	Lysine Methyltransferase 2E (Inactive)	Protein Coding	Q8IZD2	45	GC07P105102	0.464545	https://www.genecards.org/cgi-bin/carddisp.pl?gene=KMT2E
SGO1	Shugoshin 1	Protein Coding	Q5FBB7	45	GC03M024542	0.464545	https://www.genecards.org/cgi-bin/carddisp.pl?gene=SGO1
TOR1AIP1	Torsin 1A Interacting Protein 1	Protein Coding	Q5JTV8	44	GC01P179882	0.464545	https://www.genecards.org/cgi-bin/carddisp.pl?gene=TOR1AIP1
CD1C	CD1c Molecule	Protein Coding	P29017	42	GC01P158289	0.464545	https://www.genecards.org/cgi-bin/carddisp.pl?gene=CD1C

CHAF1A	Chromatin Assembly Factor 1 Subunit A	Protein Coding	Q13111	42	GC19P004402	0.464545	https://www.genecards.org/cgi-bin/carddisp.pl?gene=CHAF1A
DR1	Down-Regulator Of Transcription 1	Protein Coding	Q01658	42	GC01P093345	0.464545	https://www.genecards.org/cgi-bin/carddisp.pl?gene=DR1
GUCY2F	Guanylate Cyclase 2F, Retinal	Protein Coding	P51841	42	GC0XM109372	0.464545	https://www.genecards.org/cgi-bin/carddisp.pl?gene=GUCY2F
REC8	REC8 Meiotic Recombination Protein	Protein Coding	O95072	42	GC14P024171	0.464545	https://www.genecards.org/cgi-bin/carddisp.pl?gene=REC8
SYCE1	Synaptonemal Complex Central Element Protein 1	Protein Coding	Q8N0S2	42	GC10M133553	0.464545	https://www.genecards.org/cgi-bin/carddisp.pl?gene=SYCE1
TOP3B	DNA Topoisomerase III Beta	Protein Coding	O95985	42	GC22M021957	0.464545	https://www.genecards.org/cgi-bin/carddisp.pl?gene=TOP3B
AMOTL2	Angiomotin Like 2	Protein Coding	Q9Y2J4	41	GC03M134355	0.464545	https://www.genecards.org/cgi-bin/carddisp.pl?gene=AMOTL2
TRIM68	Tripartite Motif Containing 68	Protein Coding	Q6AZZ1	41	GC11M004598	0.464545	https://www.genecards.org/cgi-bin/carddisp.pl?gene=TRIM68
HES5	Hes Family BHLH Transcription Factor 5	Protein Coding	Q5TA89	40	GC01M002528	0.464545	https://www.genecards.org/cgi-bin/carddisp.pl?gene=HES5
NANOS3	Nanos C2HC-Type Zinc Finger 3	Protein Coding	P60323	40	GC19P013862	0.464545	https://www.genecards.org/cgi-bin/carddisp.pl?gene=NANOS3
SEPTIN12	Septin 12	Protein Coding	Q8IYM1	40	GC16M013532	0.464545	https://www.genecards.org/cgi-bin/carddisp.pl?gene=SEPTIN12
VGLL4	Vestigial Like Family Member 4	Protein Coding	Q14135	40	GC03M011934	0.464545	https://www.genecards.org/cgi-bin/carddisp.pl?gene=VGLL4

PUDP	Pseudouridine 5'-Phosphatase	Protein Coding	Q08623	39	GC0XM006668	0.464545	https://www.genecards.org/cgi-bin/carddisp.pl?gene=PUDP
VCX	Variable Charge X-Linked	Protein Coding	Q9H320	32	GC0XP007842	0.464545	https://www.genecards.org/cgi-bin/carddisp.pl?gene=VCX
VCX2	Variable Charge X-Linked 2	Protein Coding	Q9H322	30	GC0XM008169	0.464545	https://www.genecards.org/cgi-bin/carddisp.pl?gene=VCX2
VCX3A	Variable Charge X-Linked 3A	Protein Coding	Q9NNX9	30	GC0XM006533	0.464545	https://www.genecards.org/cgi-bin/carddisp.pl?gene=VCX3A
MIR135B	MicroRNA 135b	RNA Gene		22	GC01M205448	0.464545	https://www.genecards.org/cgi-bin/carddisp.pl?gene=MIR135B
ZFYVE16	Zinc Finger FYVE-Type Containing 16	Protein Coding	Q7Z3T8	44	GC05P080407	0.459834	https://www.genecards.org/cgi-bin/carddisp.pl?gene=ZFYVE16
FOXO4	Forkhead Box O4	Protein Coding	P98177	46	GC0XP071095	0.457412	https://www.genecards.org/cgi-bin/carddisp.pl?gene=FOXO4
DES	Desmin	Protein Coding	P17661	52	GC02P219418	0.453954	https://www.genecards.org/cgi-bin/carddisp.pl?gene=DES
CTSC	Cathepsin C	Protein Coding	P53634	51	GC11M114127	0.453954	https://www.genecards.org/cgi-bin/carddisp.pl?gene=CTSC
TOP1	DNA Topoisomerase I	Protein Coding	P11387	53	GC20P041028	0.451858	https://www.genecards.org/cgi-bin/carddisp.pl?gene=TOP1
TUBB3	Tubulin Beta 3 Class III	Protein Coding	Q13509	53	GC16P092275	0.451858	https://www.genecards.org/cgi-bin/carddisp.pl?gene=TUBB3
SCN3A	Sodium Voltage-Gated Channel Alpha Subunit 3	Protein Coding	Q9NY46	52	GC02M165087	0.451858	https://www.genecards.org/cgi-bin/carddisp.pl?gene=SCN3A

FLNC	Filamin C	Protein Coding	Q14315	48	GC07P128830	0.451858	https://www.genecards.org/cgi-bin/carddisp.pl?gene=FLNC
PSMA7	Proteasome 20S Subunit Alpha 7	Protein Coding	O14818	48	GC20M062136	0.451858	https://www.genecards.org/cgi-bin/carddisp.pl?gene=PSMA7
RPL18	Ribosomal Protein L18	Protein Coding	Q07020	48	GC19M048615	0.451858	https://www.genecards.org/cgi-bin/carddisp.pl?gene=RPL18
RPS10	Ribosomal Protein S10	Protein Coding	P46783	48	GC06M084077	0.451858	https://www.genecards.org/cgi-bin/carddisp.pl?gene=RPS10
HNRNPD	Heterogeneous Nuclear Ribonucleoprotein D	Protein Coding	Q14103	47	GC04M082352	0.451858	https://www.genecards.org/cgi-bin/carddisp.pl?gene=HNRNPD
SRSF1	Serine And Arginine Rich Splicing Factor 1	Protein Coding	Q07955	47	GC17M065669	0.451858	https://www.genecards.org/cgi-bin/carddisp.pl?gene=SRSF1
WDR1	WD Repeat Domain 1	Protein Coding	O75083	45	GC04M010073	0.451858	https://www.genecards.org/cgi-bin/carddisp.pl?gene=WDR1
BLVRB	Biliverdin Reductase B	Protein Coding	P30043	44	GC19M040447	0.451858	https://www.genecards.org/cgi-bin/carddisp.pl?gene=BLVRB
SNRNP40	Small Nuclear Ribonucleoprotein U5 Subunit 40	Protein Coding	Q96DI7	41	GC01M031259	0.451858	https://www.genecards.org/cgi-bin/carddisp.pl?gene=SNRNP40
MIR1224	MicroRNA 1224	RNA Gene		19	GC03P184241	0.451858	https://www.genecards.org/cgi-bin/carddisp.pl?gene=MIR1224
UBA1	Ubiquitin Like Modifier Activating Enzyme 1	Protein Coding	P22314	53	GC0XP047190	0.45072	https://www.genecards.org/cgi-bin/carddisp.pl?gene=UBA1
GRM3	Glutamate Metabotropic Receptor 3	Protein Coding	Q14832	49	GC07P086643	0.45072	https://www.genecards.org/cgi-bin/carddisp.pl?gene=GRM3

ARRB1	Arrestin Beta 1	Protein Coding	P49407	48	GC11M113899	0.45072	https://www.genecards.org/cgi-bin/carddisp.pl?gene=ARRB1
HDAC11	Histone Deacetylase 11	Protein Coding	Q96DB2	48	GC03P018424	0.45072	https://www.genecards.org/cgi-bin/carddisp.pl?gene=HDAC11
FUT6	Fucosyltransferase 6	Protein Coding	P51993	45	GC19M005830	0.45072	https://www.genecards.org/cgi-bin/carddisp.pl?gene=FUT6
MGRN1	Mahogunin Ring Finger 1	Protein Coding	O60291	44	GC16P052517	0.45072	https://www.genecards.org/cgi-bin/carddisp.pl?gene=MGRN1
CCNG2	Cyclin G2	Protein Coding	Q16589	41	GC04P077158	0.45072	https://www.genecards.org/cgi-bin/carddisp.pl?gene=CCNG2
MIR4766	MicroRNA 4766	RNA Gene		16	GC22M040813	0.45072	https://www.genecards.org/cgi-bin/carddisp.pl?gene=MIR4766
MTA3	Metastasis Associated 1 Family Member 3	Protein Coding	Q9BTC8	46	GC02P042494	0.449598	https://www.genecards.org/cgi-bin/carddisp.pl?gene=MTA3
CMA1	Chymase 1	Protein Coding	P23946	48	GC14M025834	0.44497	https://www.genecards.org/cgi-bin/carddisp.pl?gene=CMA1
UBE4A	Ubiquitination Factor E4A	Protein Coding	Q14139	45	GC11P118359	0.443787	https://www.genecards.org/cgi-bin/carddisp.pl?gene=UBE4A
GABRB2	Gamma-Aminobutyric Acid Type A Receptor Subunit Beta2	Protein Coding	P47870	52	GC05M161288	0.43547	https://www.genecards.org/cgi-bin/carddisp.pl?gene=GABRB2
CHRNE	Cholinergic Receptor Nicotinic Epsilon Subunit	Protein Coding	Q04844	49	GC17M004897	0.43547	https://www.genecards.org/cgi-bin/carddisp.pl?gene=CHRNE
CPD	Carboxypeptidase D	Protein Coding	O75976	46	GC17P030378	0.43547	https://www.genecards.org/cgi-bin/carddisp.pl?gene=CPD

TYK2	Tyrosine Kinase 2	Protein Coding	P29597	57	GC19M010350	0.432972	https://www.genecards.org/cgi-bin/carddisp.pl?gene=TYK2
IFNAR2	Interferon Alpha And Beta Receptor Subunit 2	Protein Coding	P48551	53	GC21P033229	0.432972	https://www.genecards.org/cgi-bin/carddisp.pl?gene=IFNAR2
IKBKG	Inhibitor Of Nuclear Factor Kappa B Kinase Regulatory Subunit Gamma	Protein Coding	Q9Y6K9	52	GC0XP154541	0.432972	https://www.genecards.org/cgi-bin/carddisp.pl?gene=IKBKG
BCL6	BCL6 Transcription Repressor	Protein Coding	P41182	50	GC03M187721	0.432972	https://www.genecards.org/cgi-bin/carddisp.pl?gene=BCL6
CDC6	Cell Division Cycle 6	Protein Coding	Q99741	50	GC17P040287	0.432972	https://www.genecards.org/cgi-bin/carddisp.pl?gene=CDC6
BTRC	Beta-Transducin Repeat Containing E3 Ubiquitin Protein Ligase	Protein Coding	Q9Y297	49	GC10P101354	0.432972	https://www.genecards.org/cgi-bin/carddisp.pl?gene=BTRC
NONO	Non-POU Domain Containing Octamer Binding	Protein Coding	Q15233	49	GC0XP071255	0.432972	https://www.genecards.org/cgi-bin/carddisp.pl?gene=NONO
ZDHHC17	Zinc Finger DHHC-Type Palmitoyltransferase 17	Protein Coding	Q8IUH5	42	GC12P076763	0.432972	https://www.genecards.org/cgi-bin/carddisp.pl?gene=ZDHHC17
SHROOM3	Shroom Family Member 3	Protein Coding	Q8TF72	41	GC04P076435	0.432972	https://www.genecards.org/cgi-bin/carddisp.pl?gene=SHROOM3
MIR873	MicroRNA 873	RNA Gene		19	GC09M028888	0.432972	https://www.genecards.org/cgi-bin/carddisp.pl?gene=MIR873
MIR506	MicroRNA 506	RNA Gene		16	GC0XM147230	0.432972	https://www.genecards.org/cgi-bin/carddisp.pl?gene=MIR506
COL6A5	Collagen Type VI Alpha 5 Chain	Protein Coding	A8TX70	39	GC03P130345	0.428948	https://www.genecards.org/cgi-bin/carddisp.pl?gene=COL6A5

MIR190B	MicroRNA 190b	RNA Gene		18	GC01M154193	0.428948	https://www.genecards.org/cgi-bin/carddisp.pl?gene=MIR190B
MMAB	Metabolism Of Cobalamin Associated B	Protein Coding	Q96EY8	48	GC12M109553	0.426977	https://www.genecards.org/cgi-bin/carddisp.pl?gene=MMAB
MAP2K2	Mitogen-Activated Protein Kinase Kinase 2	Protein Coding	P36507	58	GC19M004090	0.417212	https://www.genecards.org/cgi-bin/carddisp.pl?gene=MAP2K2
VAPA	VAMP Associated Protein A	Protein Coding	Q9P0L0	46	GC18P009904	0.41213	https://www.genecards.org/cgi-bin/carddisp.pl?gene=VAPA
CYP21A1P	Cytochrome P450 Family 21 Subfamily A Member 1, Pseudogene	Pseudogene		16	GC06P032005	0.41213	https://www.genecards.org/cgi-bin/carddisp.pl?gene=CYP21A1P
PRKDC	Protein Kinase, DNA-Activated, Catalytic Subunit	Protein Coding	P78527	54	GC08M047773	0.408896	https://www.genecards.org/cgi-bin/carddisp.pl?gene=PRKDC
PPP1CC	Protein Phosphatase 1 Catalytic Subunit Gamma	Protein Coding	P36873	50	GC12M110709	0.408896	https://www.genecards.org/cgi-bin/carddisp.pl?gene=PPP1CC
GAB1	GRB2 Associated Binding Protein 1	Protein Coding	Q13480	49	GC04P143336	0.408896	https://www.genecards.org/cgi-bin/carddisp.pl?gene=GAB1
RPL35	Ribosomal Protein L35	Protein Coding	P42766	48	GC09M124857	0.408896	https://www.genecards.org/cgi-bin/carddisp.pl?gene=RPL35
TUBB2B	Tubulin Beta 2B Class IIb	Protein Coding	Q9BVA1	48	GC06M003540	0.408896	https://www.genecards.org/cgi-bin/carddisp.pl?gene=TUBB2B
HNRNPU	Heterogeneous Nuclear Ribonucleoprotein U	Protein Coding	Q00839	47	GC01M244844	0.408896	https://www.genecards.org/cgi-bin/carddisp.pl?gene=HNRNPU
LIAS	Lipoic Acid Synthetase	Protein Coding	O43766	47	GC04P039512	0.408896	https://www.genecards.org/cgi-bin/carddisp.pl?gene=LIAS

PPP2R5C	Protein Phosphatase 2 Regulatory Subunit B'Gamma	Protein Coding	Q13362	47	GC14P113687	0.408896	https://www.genecards.org/cgi-bin/carddisp.pl?gene=PPP2R5C
PSMA4	Proteasome 20S Subunit Alpha 4	Protein Coding	P25789	47	GC15P078540	0.408896	https://www.genecards.org/cgi-bin/carddisp.pl?gene=PSMA4
PSMB5	Proteasome 20S Subunit Beta 5	Protein Coding	P28074	47	GC14M023016	0.408896	https://www.genecards.org/cgi-bin/carddisp.pl?gene=PSMB5
PSMC1	Proteasome 26S Subunit, ATPase 1	Protein Coding	P62191	47	GC14P090256	0.408896	https://www.genecards.org/cgi-bin/carddisp.pl?gene=PSMC1
CNOT1	CCR4-NOT Transcription Complex Subunit 1	Protein Coding	A5YKK6	46	GC16M058519	0.408896	https://www.genecards.org/cgi-bin/carddisp.pl?gene=CNOT1
HNRNPR	Heterogeneous Nuclear Ribonucleoprotein R	Protein Coding	O43390	46	GC01M023303	0.408896	https://www.genecards.org/cgi-bin/carddisp.pl?gene=HNRNPR
PLS3	Plastin 3	Protein Coding	P13797	46	GC0XP115560	0.408896	https://www.genecards.org/cgi-bin/carddisp.pl?gene=PLS3
DNAJA3	DnaJ Heat Shock Protein Family (Hsp40) Member A3	Protein Coding	Q96EY1	45	GC16P004425	0.408896	https://www.genecards.org/cgi-bin/carddisp.pl?gene=DNAJA3
RPL13A	Ribosomal Protein L13a	Protein Coding	P40429	45	GC19P049487	0.408896	https://www.genecards.org/cgi-bin/carddisp.pl?gene=RPL13A
RPL22	Ribosomal Protein L22	Protein Coding	P35268	45	GC01M010745	0.408896	https://www.genecards.org/cgi-bin/carddisp.pl?gene=RPL22
RPS9	Ribosomal Protein S9	Protein Coding	P46781	45	GC19P094646	0.408896	https://www.genecards.org/cgi-bin/carddisp.pl?gene=RPS9
CHP1	Calcineurin Like EF-Hand Protein 1	Protein Coding	Q99653	44	GC15P041230	0.408896	https://www.genecards.org/cgi-bin/carddisp.pl?gene=CHP1

DDX17	DEAD-Box Helicase 17	Protein Coding	Q92841	44	GC22M038483	0.408896	https://www.genecards.org/cgi-bin/carddisp.pl?gene=DDX17
DNAJC7	DnaJ Heat Shock Protein Family (Hsp40) Member C7	Protein Coding	Q99615	44	GC17M064144	0.408896	https://www.genecards.org/cgi-bin/carddisp.pl?gene=DNAJC7
PSMD6	Proteasome 26S Subunit, Non-ATPase 6	Protein Coding	Q15008	44	GC03M063973	0.408896	https://www.genecards.org/cgi-bin/carddisp.pl?gene=PSMD6
RBM14	RNA Binding Motif Protein 14	Protein Coding	Q96PK6	44	GC11P079948	0.408896	https://www.genecards.org/cgi-bin/carddisp.pl?gene=RBM14
RPL24	Ribosomal Protein L24	Protein Coding	P83731	44	GC03M101681	0.408896	https://www.genecards.org/cgi-bin/carddisp.pl?gene=RPL24
RPL6	Ribosomal Protein L6	Protein Coding	Q02878	44	GC12M112320	0.408896	https://www.genecards.org/cgi-bin/carddisp.pl?gene=RPL6
RPLP2	Ribosomal Protein Lateral Stalk Subunit P2	Protein Coding	P05387	44	GC11P004477	0.408896	https://www.genecards.org/cgi-bin/carddisp.pl?gene=RPLP2
HNRNPA3	Heterogeneous Nuclear Ribonucleoprotein A3	Protein Coding	P51991	43	GC02P177212	0.408896	https://www.genecards.org/cgi-bin/carddisp.pl?gene=HNRNPA3
KLF12	KLF Transcription Factor 12	Protein Coding	Q9Y4X4	42	GC13M073686	0.408896	https://www.genecards.org/cgi-bin/carddisp.pl?gene=KLF12
U2AF2	U2 Small Nuclear RNA Auxiliary Factor 2	Protein Coding	P26368	42	GC19P055654	0.408896	https://www.genecards.org/cgi-bin/carddisp.pl?gene=U2AF2
ZMYM3	Zinc Finger MYM-Type Containing 3	Protein Coding	Q14202	42	GC0XM071239	0.408896	https://www.genecards.org/cgi-bin/carddisp.pl?gene=ZMYM3
CGN	Cingulin	Protein Coding	Q9P2M7	41	GC01P156854	0.408896	https://www.genecards.org/cgi-bin/carddisp.pl?gene=CGN

VSIG4	V-Set And Immunoglobulin Domain Containing 4	Protein Coding	Q9Y279	41	GC0XM066021	0.408896	https://www.genecards.org/cgi-bin/carddisp.pl?gene=VSIG4
PSPC1	Paraspeckle Component 1	Protein Coding	Q8WXF1	40	GC13M022028	0.408896	https://www.genecards.org/cgi-bin/carddisp.pl?gene=PSPC1
UTP14A	UTP14A Small Subunit Processome Component	Protein Coding	Q9BVJ6	40	GC0XP129906	0.408896	https://www.genecards.org/cgi-bin/carddisp.pl?gene=UTP14A
MIR520H	MicroRNA 520h	RNA Gene		18	GC19P090147	0.408896	https://www.genecards.org/cgi-bin/carddisp.pl?gene=MIR520H
MIR193BHG	MIR193b-365a Host Gene	RNA Gene		15	GC16P052738	0.408896	https://www.genecards.org/cgi-bin/carddisp.pl?gene=MIR193BHG
LYVE1	Lymphatic Vessel Endothelial Hyaluronan Receptor 1	Protein Coding	Q9Y5Y7	46	GC11M011321	0.406812	https://www.genecards.org/cgi-bin/carddisp.pl?gene=LYVE1
PPOX	Protoporphyrinogen Oxidase	Protein Coding	P50336	48	GC01P162045	0.396855	https://www.genecards.org/cgi-bin/carddisp.pl?gene=PPOX
EFS	Embryonal Fyn-Associated Substrate	Protein Coding	O43281	40	GC14M023356	0.396855	https://www.genecards.org/cgi-bin/carddisp.pl?gene=EFS
UGT2B4	UDP Glucuronosyltransferase Family 2 Member B4	Protein Coding	P06133	45	GC04M069484	0.392508	https://www.genecards.org/cgi-bin/carddisp.pl?gene=UGT2B4
CHRNA10	Cholinergic Receptor Nicotinic Alpha 10 Subunit	Protein Coding	Q9GZZ6	44	GC11M009072	0.392508	https://www.genecards.org/cgi-bin/carddisp.pl?gene=CHRNA10
FMN2	Formin 2	Protein Coding	Q9NZ56	43	GC01P240014	0.392508	https://www.genecards.org/cgi-bin/carddisp.pl?gene=FMN2
INHBC	Inhibin Subunit Beta C	Protein Coding	P55103	41	GC12P057434	0.392508	https://www.genecards.org/cgi-bin/carddisp.pl?gene=INHBC

IRF7	Interferon Regulatory Factor 7	Protein Coding	Q92985	52	GC11M000612	0.39001	https://www.genecards.org/cgi-bin/carddisp.pl?gene=IRF7
STAT2	Signal Transducer And Activator Of Transcription 2	Protein Coding	P52630	52	GC12M056341	0.39001	https://www.genecards.org/cgi-bin/carddisp.pl?gene=STAT2
EPHA1	EPH Receptor A1	Protein Coding	P21709	51	GC07M143390	0.39001	https://www.genecards.org/cgi-bin/carddisp.pl?gene=EPHA1
PPP2R1B	Protein Phosphatase 2 Scaffold Subunit Abeta	Protein Coding	P30154	51	GC11M114459	0.39001	https://www.genecards.org/cgi-bin/carddisp.pl?gene=PPP2R1B
SGPL1	Sphingosine-1-Phosphate Lyase 1	Protein Coding	O95470	51	GC10P070815	0.39001	https://www.genecards.org/cgi-bin/carddisp.pl?gene=SGPL1
ALDH3A1	Aldehyde Dehydrogenase 3 Family Member A1	Protein Coding	P30838	50	GC17M019737	0.39001	https://www.genecards.org/cgi-bin/carddisp.pl?gene=ALDH3A1
FADD	Fas Associated Via Death Domain	Protein Coding	Q13158	50	GC11P070203	0.39001	https://www.genecards.org/cgi-bin/carddisp.pl?gene=FADD
ANLN	Anillin, Actin Binding Protein	Protein Coding	Q9NQW6	48	GC07P036389	0.39001	https://www.genecards.org/cgi-bin/carddisp.pl?gene=ANLN
NUP107	Nucleoporin 107	Protein Coding	P57740	48	GC12P068686	0.39001	https://www.genecards.org/cgi-bin/carddisp.pl?gene=NUP107
TMPRSS6	Transmembrane Serine Protease 6	Protein Coding	Q8IU80	48	GC22M070404	0.39001	https://www.genecards.org/cgi-bin/carddisp.pl?gene=TMPRSS6
CRABP2	Cellular Retinoic Acid Binding Protein 2	Protein Coding	P29373	47	GC01M157930	0.39001	https://www.genecards.org/cgi-bin/carddisp.pl?gene=CRABP2
WIF1	WNT Inhibitory Factor 1	Protein Coding	Q9Y5W5	47	GC12M065050	0.39001	https://www.genecards.org/cgi-bin/carddisp.pl?gene=WIF1

COPS5	COP9 Signalosome Subunit 5	Protein Coding	Q92905	46	GC08M067043	0.39001	https://www.genecards.org/cgi-bin/carddisp.pl?gene=COPS5
LASP1	LIM And SH3 Protein 1	Protein Coding	Q14847	46	GC17P038869	0.39001	https://www.genecards.org/cgi-bin/carddisp.pl?gene=LASP1
NUP85	Nucleoporin 85	Protein Coding	Q9BW27	46	GC17P075205	0.39001	https://www.genecards.org/cgi-bin/carddisp.pl?gene=NUP85
RHOC	Ras Homolog Family Member C	Protein Coding	P08134	46	GC01M112701	0.39001	https://www.genecards.org/cgi-bin/carddisp.pl?gene=RHOC
TCL1A	TCL1 Family AKT Coactivator A	Protein Coding	P56279	46	GC14M095709	0.39001	https://www.genecards.org/cgi-bin/carddisp.pl?gene=TCL1A
AGGF1	Angiogenic Factor With G-Patch And FHA Domains 1	Protein Coding	Q8N302	45	GC05P077029	0.39001	https://www.genecards.org/cgi-bin/carddisp.pl?gene=AGGF1
BNIP3	BCL2 Interacting Protein 3	Protein Coding	Q12983	45	GC10M131966	0.39001	https://www.genecards.org/cgi-bin/carddisp.pl?gene=BNIP3
CSE1L	Chromosome Segregation 1 Like	Protein Coding	P55060	45	GC20P049046	0.39001	https://www.genecards.org/cgi-bin/carddisp.pl?gene=CSE1L
NES	Nestin	Protein Coding	P48681	45	GC01M156668	0.39001	https://www.genecards.org/cgi-bin/carddisp.pl?gene=NES
PIGR	Polymeric Immunoglobulin Receptor	Protein Coding	P01833	45	GC01M206928	0.39001	https://www.genecards.org/cgi-bin/carddisp.pl?gene=PIGR
KRT20	Keratin 20	Protein Coding	P35900	44	GC17M040875	0.39001	https://www.genecards.org/cgi-bin/carddisp.pl?gene=KRT20
TIGIT	T Cell Immunoreceptor With Ig And ITIM Domains	Protein Coding	Q495A1	44	GC03P114276	0.39001	https://www.genecards.org/cgi-bin/carddisp.pl?gene=TIGIT

SLC16A9	Solute Carrier Family 16 Member 9	Protein Coding	Q7RTY1	41	GC10M059650	0.39001	https://www.genecards.org/cgi-bin/carddisp.pl?gene=SLC16A9
GAS6-AS1	GAS6 Antisense RNA 1	RNA Gene		19	GC13P113815	0.39001	https://www.genecards.org/cgi-bin/carddisp.pl?gene=GAS6-AS1
MIR542	MicroRNA 542	RNA Gene		18	GC0XM134734	0.39001	https://www.genecards.org/cgi-bin/carddisp.pl?gene=MIR542
LRRC3-DT	LRRC3 Divergent Transcript	RNA Gene		14	GC21M044451	0.39001	https://www.genecards.org/cgi-bin/carddisp.pl?gene=LRRC3-DT
RPS27	Ribosomal Protein S27	Protein Coding	P42677	47	GC01P157024	0.389312	https://www.genecards.org/cgi-bin/carddisp.pl?gene=RPS27
MICA	MHC Class I Polypeptide-Related Sequence A	Protein Coding	Q29983	43	GC06P031399	0.382327	https://www.genecards.org/cgi-bin/carddisp.pl?gene=MICA
GH-LCR	Growth Hormone Locus Control Region	Functional Element		4	GC17P063917	0.382327	https://www.genecards.org/cgi-bin/carddisp.pl?gene=GH-LCR
XPO1	Exportin 1	Protein Coding	O14980	52	GC02M061445	0.381591	https://www.genecards.org/cgi-bin/carddisp.pl?gene=XPO1
WNT2	Wnt Family Member 2	Protein Coding	P09544	48	GC07M117330	0.381591	https://www.genecards.org/cgi-bin/carddisp.pl?gene=WNT2
GRIA3	Glutamate Ionotropic Receptor AMPA Type Subunit 3	Protein Coding	P42263	54	GC0XP123184	0.375387	https://www.genecards.org/cgi-bin/carddisp.pl?gene=GRIA3
CYB5R3	Cytochrome B5 Reductase 3	Protein Coding	P00387	50	GC22M070446	0.375387	https://www.genecards.org/cgi-bin/carddisp.pl?gene=CYB5R3
HLA-DRA	Major Histocompatibility Complex, Class II, DR Alpha	Protein Coding	P01903	49	GC06P032439	0.374719	https://www.genecards.org/cgi-bin/carddisp.pl?gene=HLA-DRA

TRIO	Trio Rho Guanine Nucleotide Exchange Factor	Protein Coding	O75962	48	GC05P014143	0.374719	https://www.genecards.org/cgi-bin/carddisp.pl?gene=TRIO
PFN1	Profilin 1	Protein Coding	P07737	50	GC17M004945	0.372462	https://www.genecards.org/cgi-bin/carddisp.pl?gene=PFN1
AKT1S1	AKT1 Substrate 1	Protein Coding	Q96B36	44	GC19M049869	0.358862	https://www.genecards.org/cgi-bin/carddisp.pl?gene=AKT1S1
CHML	CHM Like Rab Escort Protein	Protein Coding	P26374	41	GC01M241628	0.358862	https://www.genecards.org/cgi-bin/carddisp.pl?gene=CHML
KLLN	Killin, P53 Regulated DNA Replication Inhibitor	Protein Coding	B2CW77	34	GC10M088238	0.358862	https://www.genecards.org/cgi-bin/carddisp.pl?gene=KLLN
NEU1	Neuraminidase 1	Protein Coding	Q99519	51	GC06M031857	0.356907	https://www.genecards.org/cgi-bin/carddisp.pl?gene=NEU1
MS4A1	Membrane Spanning 4-Domains A1	Protein Coding	P11836	52	GC11P060571	0.355031	https://www.genecards.org/cgi-bin/carddisp.pl?gene=MS4A1
CLEC4M	C-Type Lectin Domain Family 4 Member M	Protein Coding	Q9H2X3	44	GC19P007763	0.355031	https://www.genecards.org/cgi-bin/carddisp.pl?gene=CLEC4M
ZNF385B	Zinc Finger Protein 385B	Protein Coding	Q569K4	38	GC02M179441	0.355031	https://www.genecards.org/cgi-bin/carddisp.pl?gene=ZNF385B
NPHP3-ACAD11	NPHP3-ACAD11 Readthrough (NMD Candidate)	RNA Gene		16	GC03M132558	0.355031	https://www.genecards.org/cgi-bin/carddisp.pl?gene=NPHP3-ACAD11
AOX1	Aldehyde Oxidase 1	Protein Coding	Q06278	48	GC02P200585	0.35105	https://www.genecards.org/cgi-bin/carddisp.pl?gene=AOX1
ACAA2	Acetyl-CoA Acyltransferase 2	Protein Coding	P42765	47	GC18M049782	0.35105	https://www.genecards.org/cgi-bin/carddisp.pl?gene=ACAA2

SERPIND1	Serpin Family D Member 1	Protein Coding	P05546	51	GC22P056074	0.350886	https://www.genecards.org/cgi-bin/carddisp.pl?gene=SERPIND1
UBE2H	Ubiquitin Conjugating Enzyme E2 H	Protein Coding	P62256	47	GC07M129830	0.350886	https://www.genecards.org/cgi-bin/carddisp.pl?gene=UBE2H
LAPTM4A	Lysosomal Protein Transmembrane 4 Alpha	Protein Coding	Q15012	40	GC02M020032	0.350886	https://www.genecards.org/cgi-bin/carddisp.pl?gene=LAPTM4A
ERCC5	ERCC Excision Repair 5, Endonuclease	Protein Coding	P28715	48	GC13P102845	0.350235	https://www.genecards.org/cgi-bin/carddisp.pl?gene=ERCC5
TNFAIP6	TNF Alpha Induced Protein 6	Protein Coding	P98066	45	GC02P151357	0.350235	https://www.genecards.org/cgi-bin/carddisp.pl?gene=TNFAIP6
LOC126862571	BRD4-Independent Group 4 Enhancer GRCh37_chr17:41243136-41244335	Functional Element		3	GC17P088426	0.350235	https://www.genecards.org/cgi-bin/carddisp.pl?gene=LOC126862571
PRDX1	Peroxiredoxin 1	Protein Coding	Q06830	53	GC01M045658	0.348625	https://www.genecards.org/cgi-bin/carddisp.pl?gene=PRDX1
LOXL2	Lysyl Oxidase Like 2	Protein Coding	Q9Y4K0	50	GC08M023296	0.332895	https://www.genecards.org/cgi-bin/carddisp.pl?gene=LOXL2
GADD45A	Growth Arrest And DNA Damage Inducible Alpha	Protein Coding	P24522	47	GC01P067685	0.332895	https://www.genecards.org/cgi-bin/carddisp.pl?gene=GADD45A
EDAR	Ectodysplasin A Receptor	Protein Coding	Q9UNE0	46	GC02M108894	0.332895	https://www.genecards.org/cgi-bin/carddisp.pl?gene=EDAR
MELTF-AS1	MELTF Antisense RNA 1	RNA Gene		16	GC03P197169	0.3295	https://www.genecards.org/cgi-bin/carddisp.pl?gene=MELTF-AS1
CDC73	Cell Division Cycle 73	Protein Coding	Q6P1J9	50	GC01P193121	0.322443	https://www.genecards.org/cgi-bin/carddisp.pl?gene=CDC73

LINC01565	Long Intergenic Non-Protein Coding RNA 1565	RNA Gene	O15544	26	GC03M128620	0.322443	https://www.genecards.org/cgi-bin/carddisp.pl?gene=LINC01565
SNHG7	Small Nucleolar RNA Host Gene 7	RNA Gene		22	GC09M138332	0.322443	https://www.genecards.org/cgi-bin/carddisp.pl?gene=SNHG7
MIR320B1	MicroRNA 320b-1	RNA Gene		17	GC01P116671	0.32157	https://www.genecards.org/cgi-bin/carddisp.pl?gene=MIR320B1
MIR320C1	MicroRNA 320c-1	RNA Gene		17	GC18P021683	0.32157	https://www.genecards.org/cgi-bin/carddisp.pl?gene=MIR320C1
MIR320B2	MicroRNA 320b-2	RNA Gene		15	GC01M224257	0.32157	https://www.genecards.org/cgi-bin/carddisp.pl?gene=MIR320B2
REL	REL Proto-Oncogene, NF-KB Subunit	Protein Coding	Q04864	51	GC02P060881	0.308411	https://www.genecards.org/cgi-bin/carddisp.pl?gene=REL
AGA	Aspartylglucosaminidase	Protein Coding	P20933	50	GC04M177430	0.308411	https://www.genecards.org/cgi-bin/carddisp.pl?gene=AGA
CNP	2',3'-Cyclic Nucleotide 3' Phosphodiesterase	Protein Coding	P09543	48	GC17P041966	0.308411	https://www.genecards.org/cgi-bin/carddisp.pl?gene=CNP
MIPEP	Mitochondrial Intermediate Peptidase	Protein Coding	Q99797	46	GC13M023730	0.308411	https://www.genecards.org/cgi-bin/carddisp.pl?gene=MIPEP
TTN-AS1	TTN Antisense RNA 1	RNA Gene		17	GC02P178521	0.308411	https://www.genecards.org/cgi-bin/carddisp.pl?gene=TTN-AS1
LINC02595	Long Intergenic Non-Protein Coding RNA 2595	RNA Gene		14	GC0XM045849	0.308411	https://www.genecards.org/cgi-bin/carddisp.pl?gene=LINC02595
MIR1269A	MicroRNA 1269a	RNA Gene		13	GC04P066276	0.308411	https://www.genecards.org/cgi-bin/carddisp.pl?gene=MIR1269A

NGFR	Nerve Growth Factor Receptor	Protein Coding	P08138	51	GC17P049495	0.307924	https://www.genecards.org/cgi-bin/carddisp.pl?gene=NGFR
RPSA	Ribosomal Protein SA	Protein Coding	P08865	51	GC03P039406	0.307924	https://www.genecards.org/cgi-bin/carddisp.pl?gene=RPSA
RAPGEF3	Rap Guanine Nucleotide Exchange Factor 3	Protein Coding	O95398	48	GC12M047736	0.307924	https://www.genecards.org/cgi-bin/carddisp.pl?gene=RAPGEF3
RBBP4	RB Binding Protein 4, Chromatin Remodeling Factor	Protein Coding	Q09028	48	GC01P032651	0.307924	https://www.genecards.org/cgi-bin/carddisp.pl?gene=RBBP4
TUBA1B	Tubulin Alpha 1b	Protein Coding	P68363	48	GC12M049127	0.307924	https://www.genecards.org/cgi-bin/carddisp.pl?gene=TUBA1B
IER3	Immediate Early Response 3	Protein Coding	P46695	41	GC06M030743	0.307924	https://www.genecards.org/cgi-bin/carddisp.pl?gene=IER3
IGF2BP2-AS1	IGF2BP2 Antisense RNA 1	RNA Gene	Q96M15	24	GC03P185620	0.307924	https://www.genecards.org/cgi-bin/carddisp.pl?gene=IGF2BP2-AS1
ATP2A3	ATPase Sarcoplasmic/Endoplasmic Reticulum Ca2+ Transporting 3	Protein Coding	Q93084	50	GC17M003923	0.305664	https://www.genecards.org/cgi-bin/carddisp.pl?gene=ATP2A3
TOMM40	Translocase Of Outer Mitochondrial Membrane 40	Protein Coding	O96008	45	GC19P044890	0.305664	https://www.genecards.org/cgi-bin/carddisp.pl?gene=TOMM40
MIR129-2	MicroRNA 129-2	RNA Gene		20	GC11P043864	0.305664	https://www.genecards.org/cgi-bin/carddisp.pl?gene=MIR129-2
OIP5-AS1	OIP5 Antisense RNA 1	RNA Gene		20	GC15P057239	0.305664	https://www.genecards.org/cgi-bin/carddisp.pl?gene=OIP5-AS1
MIR205HG	MIR205 Host Gene	RNA Gene		19	GC01P209949	0.305664	https://www.genecards.org/cgi-bin/carddisp.pl?gene=MIR205HG

LOC110973015	NOS3 5' Regulatory Region	Functional Element		3	GC07P150988	0.305664	https://www.genecards.org/cgi-bin/carddisp.pl?gene=LOC110973015
F11	Coagulation Factor XI	Protein Coding	P03951	51	GC04P186287	0.289477	https://www.genecards.org/cgi-bin/carddisp.pl?gene=F11
CANX	Calnexin	Protein Coding	P27824	50	GC05P179678	0.289477	https://www.genecards.org/cgi-bin/carddisp.pl?gene=CANX
RANBP2	RAN Binding Protein 2	Protein Coding	P49792	50	GC02P108719	0.289477	https://www.genecards.org/cgi-bin/carddisp.pl?gene=RANBP2
AARS1	Alanyl-TRNA Synthetase 1	Protein Coding	P49588	48	GC16M072240	0.289477	https://www.genecards.org/cgi-bin/carddisp.pl?gene=AARS1
CHST3	Carbohydrate Sulfotransferase 3	Protein Coding	Q7LGC8	48	GC10P071964	0.289477	https://www.genecards.org/cgi-bin/carddisp.pl?gene=CHST3
BCOR	BCL6 Corepressor	Protein Coding	Q6W2J9	47	GC0XM040049	0.289477	https://www.genecards.org/cgi-bin/carddisp.pl?gene=BCOR
PKMYT1	Protein Kinase, Membrane Associated Tyrosine/Threonine 1	Protein Coding	Q99640	46	GC16M013431	0.289477	https://www.genecards.org/cgi-bin/carddisp.pl?gene=PKMYT1
NAIP	NLR Family Apoptosis Inhibitory Protein	Protein Coding	Q13075	43	GC05M070968	0.289477	https://www.genecards.org/cgi-bin/carddisp.pl?gene=NAIP
METTL14	Methyltransferase 14, N6-Adenosine-Methyltransferase Subunit	Protein Coding	Q9HCE5	42	GC04P118685	0.289477	https://www.genecards.org/cgi-bin/carddisp.pl?gene=METTL14
RNLS	Renalase, FAD Dependent Amine Oxidase	Protein Coding	Q5VYX0	42	GC10M088245	0.289477	https://www.genecards.org/cgi-bin/carddisp.pl?gene=RNLS
AHSP	Alpha Hemoglobin Stabilizing Protein	Protein Coding	Q9NZD4	40	GC16P031527	0.289477	https://www.genecards.org/cgi-bin/carddisp.pl?gene=AHSP

CCL27	C-C Motif Chemokine Ligand 27	Protein Coding	Q9Y4X3	40	GC09M034662	0.289477	https://www.genecards.org/cgi-bin/carddisp.pl?gene=CCL27
PTPRQ	Protein Tyrosine Phosphatase Receptor Type Q	Protein Coding	Q9UMZ3	37	GC12P080402	0.289477	https://www.genecards.org/cgi-bin/carddisp.pl?gene=PTPRQ
MIMT1	MER1 Repeat Containing Imprinted Transcript 1	RNA Gene		20	GC19P056840	0.289477	https://www.genecards.org/cgi-bin/carddisp.pl?gene=MIMT1
LOC126807125	MED14-Independent Group 3 Enhancer GRCh37_chr4:103188587-103189786	Functional Element		3	GC04P102267	0.289477	https://www.genecards.org/cgi-bin/carddisp.pl?gene=LOC126807125
ADAM19	ADAM Metallopeptidase Domain 19	Protein Coding	Q9H013	47	GC05M157395	0.280619	https://www.genecards.org/cgi-bin/carddisp.pl?gene=ADAM19
ID2	Inhibitor Of DNA Binding 2	Protein Coding	Q02363	47	GC02P008678	0.280619	https://www.genecards.org/cgi-bin/carddisp.pl?gene=ID2
GZMA	Granzyme A	Protein Coding	P12544	46	GC05P055102	0.280619	https://www.genecards.org/cgi-bin/carddisp.pl?gene=GZMA
H3-4	H3.4 Histone, Cluster Member	Protein Coding	Q16695	41	GC01M228427	0.280619	https://www.genecards.org/cgi-bin/carddisp.pl?gene=H3-4
SHOC1	Shortage In Chiasmata 1	Protein Coding	Q5VXU9	32	GC09M111689	0.280619	https://www.genecards.org/cgi-bin/carddisp.pl?gene=SHOC1
NRAD1	Non-Coding RNA In The Aldehyde Dehydrogenase 1A Pathway	RNA Gene		16	GC13P043910	0.280619	https://www.genecards.org/cgi-bin/carddisp.pl?gene=NRAD1
MIR1290	MicroRNA 1290	RNA Gene		15	GC01M018897	0.280619	https://www.genecards.org/cgi-bin/carddisp.pl?gene=MIR1290
CALCR	Calcitonin Receptor	Protein Coding	P30988	52	GC07M093424	0.278608	https://www.genecards.org/cgi-bin/carddisp.pl?gene=CALCR

CCNH	Cyclin H	Protein Coding	P51946	50	GC05M087311	0.278608	https://www.genecards.org/cgi-bin/carddisp.pl?gene=CCNH
TRAF4	TNF Receptor Associated Factor 4	Protein Coding	Q9BUZ4	47	GC17P085851	0.278608	https://www.genecards.org/cgi-bin/carddisp.pl?gene=TRAF4
MIR135A2	MicroRNA 135a-2	RNA Gene		18	GC12P097563	0.278608	https://www.genecards.org/cgi-bin/carddisp.pl?gene=MIR135A2
PRMT1	Protein Arginine Methyltransferase 1	Protein Coding	Q99873	53	GC19P049675	0.247653	https://www.genecards.org/cgi-bin/carddisp.pl?gene=PRMT1
CACNA1A	Calcium Voltage-Gated Channel Subunit Alpha1 A	Protein Coding	O00555	52	GC19M013206	0.247653	https://www.genecards.org/cgi-bin/carddisp.pl?gene=CACNA1A
GRK6	G Protein-Coupled Receptor Kinase 6	Protein Coding	P43250	50	GC05P177403	0.247653	https://www.genecards.org/cgi-bin/carddisp.pl?gene=GRK6
NUMB	NUMB Endocytic Adaptor Protein	Protein Coding	P49757	50	GC14M073275	0.247653	https://www.genecards.org/cgi-bin/carddisp.pl?gene=NUMB
VAV1	Vav Guanine Nucleotide Exchange Factor 1	Protein Coding	P15498	49	GC19P006772	0.247653	https://www.genecards.org/cgi-bin/carddisp.pl?gene=VAV1
EIF4A2	Eukaryotic Translation Initiation Factor 4A2	Protein Coding	Q14240	48	GC03P186783	0.247653	https://www.genecards.org/cgi-bin/carddisp.pl?gene=EIF4A2
SERPINA5	Serpin Family A Member 5	Protein Coding	P05154	48	GC14P094563	0.247653	https://www.genecards.org/cgi-bin/carddisp.pl?gene=SERPINA5
PTPRT	Protein Tyrosine Phosphatase Receptor Type T	Protein Coding	O14522	47	GC20M042073	0.247653	https://www.genecards.org/cgi-bin/carddisp.pl?gene=PTPRT
SP3	Sp3 Transcription Factor	Protein Coding	Q02447	47	GC02M173882	0.247653	https://www.genecards.org/cgi-bin/carddisp.pl?gene=SP3

DDAH1	Dimethylarginine Dimethylaminohydrolase 1	Protein Coding	O94760	46	GC01M085318	0.247653	https://www.genecards.org/cgi-bin/carddisp.pl?gene=DDAH1
MEIS2	Meis Homeobox 2	Protein Coding	O14770	46	GC15M036889	0.247653	https://www.genecards.org/cgi-bin/carddisp.pl?gene=MEIS2
TRPV3	Transient Receptor Potential Cation Channel Subfamily V Member 3	Protein Coding	Q8NET8	46	GC17M014176	0.247653	https://www.genecards.org/cgi-bin/carddisp.pl?gene=TRPV3
BHLHE40	Basic Helix-Loop-Helix Family Member E40	Protein Coding	O14503	45	GC03P004980	0.247653	https://www.genecards.org/cgi-bin/carddisp.pl?gene=BHLHE40
DNAJB6	DnaJ Heat Shock Protein Family (Hsp40) Member B6	Protein Coding	O75190	45	GC07P157335	0.247653	https://www.genecards.org/cgi-bin/carddisp.pl?gene=DNAJB6
ST8SIA4	ST8 Alpha-N-Acetyl-Neuraminide Alpha-2,8-Sialyltransferase 4	Protein Coding	Q92187	45	GC05M100806	0.247653	https://www.genecards.org/cgi-bin/carddisp.pl?gene=ST8SIA4
ATP5PF	ATP Synthase Peripheral Stalk Subunit F6	Protein Coding	P18859	44	GC21M026086	0.247653	https://www.genecards.org/cgi-bin/carddisp.pl?gene=ATP5PF
NTN4	Netrin 4	Protein Coding	Q9HB63	44	GC12M095657	0.247653	https://www.genecards.org/cgi-bin/carddisp.pl?gene=NTN4
POLR2L	RNA Polymerase II, I And III Subunit L	Protein Coding	P62875	44	GC11M008916	0.247653	https://www.genecards.org/cgi-bin/carddisp.pl?gene=POLR2L
LALBA	Lactalbumin Alpha	Protein Coding	P00709	42	GC12M048567	0.247653	https://www.genecards.org/cgi-bin/carddisp.pl?gene=LALBA
RIT2	Ras Like Without CAAX 2	Protein Coding	Q99578	42	GC18M042743	0.247653	https://www.genecards.org/cgi-bin/carddisp.pl?gene=RIT2
TAS1R1	Taste 1 Receptor Member 1	Protein Coding	Q7RTX1	42	GC01P006555	0.247653	https://www.genecards.org/cgi-bin/carddisp.pl?gene=TAS1R1

CNTNAP5	Contactin Associated Protein Family Member 5	Protein Coding	Q8WYK1	39	GC02P124025	0.247653	https://www.genecards.org/cgi-bin/carddisp.pl?gene=CNTNAP5
CPEB2	Cytoplasmic Polyadenylation Element Binding Protein 2	Protein Coding	Q7Z5Q1	39	GC04P022183	0.247653	https://www.genecards.org/cgi-bin/carddisp.pl?gene=CPEB2
CPLANE1	Ciliogenesis And Planar Polarity Effector Complex Subunit 1	Protein Coding	Q9H799	38	GC05M038138	0.247653	https://www.genecards.org/cgi-bin/carddisp.pl?gene=CPLANE1
DAOA-AS1	DAOA Antisense RNA 1	RNA Gene		20	GC13M105459	0.247653	https://www.genecards.org/cgi-bin/carddisp.pl?gene=DAOA-AS1
LINC00511	Long Intergenic Non-Protein Coding RNA 511	RNA Gene		18	GC17M072323	0.247653	https://www.genecards.org/cgi-bin/carddisp.pl?gene=LINC00511
LINC00673	Long Intergenic Non-Protein Coding RNA 673	RNA Gene		16	GC17M072290	0.247653	https://www.genecards.org/cgi-bin/carddisp.pl?gene=LINC00673
LOC157273	Uncharacterized LOC157273	RNA Gene		10	GC08P009325	0.247653	https://www.genecards.org/cgi-bin/carddisp.pl?gene=LOC157273
MIR6769A	MicroRNA 6769a	RNA Gene		9	GC16P004671	0.247653	https://www.genecards.org/cgi-bin/carddisp.pl?gene=MIR6769A
MIR6777	MicroRNA 6777	RNA Gene		8	GC17M017814	0.247653	https://www.genecards.org/cgi-bin/carddisp.pl?gene=MIR6777
PTPN6	Protein Tyrosine Phosphatase Non-Receptor Type 6	Protein Coding	P29350	53	GC12P030500	0.246515	https://www.genecards.org/cgi-bin/carddisp.pl?gene=PTPN6
GABBR1	Gamma-Aminobutyric Acid Type B Receptor Subunit 1	Protein Coding	Q9UBS5	52	GC06M029555	0.246515	https://www.genecards.org/cgi-bin/carddisp.pl?gene=GABBR1
HYOU1	Hypoxia Up-Regulated 1	Protein Coding	Q9Y4L1	50	GC11M119442	0.246515	https://www.genecards.org/cgi-bin/carddisp.pl?gene=HYOU1

PKN2	Protein Kinase N2	Protein Coding	Q16513	50	GC01P088684	0.246515	https://www.genecards.org/cgi-bin/carddisp.pl?gene=PKN2
MBTPS1	Membrane Bound Transcription Factor Peptidase, Site 1	Protein Coding	Q14703	48	GC16M084053	0.246515	https://www.genecards.org/cgi-bin/carddisp.pl?gene=MBTPS1
POLK	DNA Polymerase Kappa	Protein Coding	Q9UBT6	47	GC05P075511	0.246515	https://www.genecards.org/cgi-bin/carddisp.pl?gene=POLK
VDAC2	Voltage Dependent Anion Channel 2	Protein Coding	P45880	47	GC10P075210	0.246515	https://www.genecards.org/cgi-bin/carddisp.pl?gene=VDAC2
DKK3	Dickkopf WNT Signaling Pathway Inhibitor 3	Protein Coding	Q9UBP4	45	GC11M011962	0.246515	https://www.genecards.org/cgi-bin/carddisp.pl?gene=DKK3
IKZF2	IKAROS Family Zinc Finger 2	Protein Coding	Q9UKS7	45	GC02M213001	0.246515	https://www.genecards.org/cgi-bin/carddisp.pl?gene=IKZF2
CAND1	Cullin Associated And Neddylation Dissociated 1	Protein Coding	Q86VP6	44	GC12P067270	0.246515	https://www.genecards.org/cgi-bin/carddisp.pl?gene=CAND1
AP2A2	Adaptor Related Protein Complex 2 Subunit Alpha 2	Protein Coding	O94973	42	GC11P000924	0.246515	https://www.genecards.org/cgi-bin/carddisp.pl?gene=AP2A2
GPS1	G Protein Pathway Suppressor 1	Protein Coding	Q13098	41	GC17P082050	0.246515	https://www.genecards.org/cgi-bin/carddisp.pl?gene=GPS1
H1-1	H1.1 Linker Histone, Cluster Member	Protein Coding	Q02539	41	GC06M083565	0.246515	https://www.genecards.org/cgi-bin/carddisp.pl?gene=H1-1
ZNF512	Zinc Finger Protein 512	Protein Coding	Q96ME7	36	GC02P027582	0.246515	https://www.genecards.org/cgi-bin/carddisp.pl?gene=ZNF512
CCDC26	CCDC26 Long Non-Coding RNA	RNA Gene		27	GC08M134319	0.246515	https://www.genecards.org/cgi-bin/carddisp.pl?gene=CCDC26

MTLN	Mitoregulin	Protein Coding	Q8NCU8	25	GC02M112151	0.246515	https://www.genecards.org/cgi-bin/carddisp.pl?gene=MTLN
LINC00977	Long Intergenic Non-Protein Coding RNA 977	RNA Gene		16	GC08M134317	0.246515	https://www.genecards.org/cgi-bin/carddisp.pl?gene=LINC00977
MIR4687	MicroRNA 4687	RNA Gene		15	GC11P003856	0.246515	https://www.genecards.org/cgi-bin/carddisp.pl?gene=MIR4687
LINC00976	Long Intergenic Non-Protein Coding RNA 976	RNA Gene		14	GC08M128634	0.246515	https://www.genecards.org/cgi-bin/carddisp.pl?gene=LINC00976
LNCTAM34A	Long Non Coding Transcriptional Activator Of MiR34a	RNA Gene		14	GC01P009183	0.246515	https://www.genecards.org/cgi-bin/carddisp.pl?gene=LNCTAM34A
LOC112340393	Sharpr-MPRA Regulatory Region 4004	Functional Element		2	GC16P055525	0.246515	https://www.genecards.org/cgi-bin/carddisp.pl?gene=LOC112340393
GRM1	Glutamate Metabotropic Receptor 1	Protein Coding	Q13255	54	GC06P145973	0.204691	https://www.genecards.org/cgi-bin/carddisp.pl?gene=GRM1
PLCB1	Phospholipase C Beta 1	Protein Coding	Q9NQ66	52	GC20P008061	0.204691	https://www.genecards.org/cgi-bin/carddisp.pl?gene=PLCB1
VKORC1	Vitamin K Epoxide Reductase Complex Subunit 1	Protein Coding	Q9BQB6	52	GC16M041735	0.204691	https://www.genecards.org/cgi-bin/carddisp.pl?gene=VKORC1
ARHGDIA	Rho GDP Dissociation Inhibitor Alpha	Protein Coding	P52565	51	GC17M081867	0.204691	https://www.genecards.org/cgi-bin/carddisp.pl?gene=ARHGDIA
CSTB	Cystatin B	Protein Coding	P04080	51	GC21M043772	0.204691	https://www.genecards.org/cgi-bin/carddisp.pl?gene=CSTB
PTPRA	Protein Tyrosine Phosphatase Receptor Type A	Protein Coding	P18433	50	GC20P002864	0.204691	https://www.genecards.org/cgi-bin/carddisp.pl?gene=PTPRA

TBL1XR1	TBL1X/Y Related 1	Protein Coding	Q9BZK7	50	GC03M177019	0.204691	https://www.genecards.org/cgi-bin/carddisp.pl?gene=TBL1XR1
DAXX	Death Domain Associated Protein	Protein Coding	Q9UER7	48	GC06M033318	0.204691	https://www.genecards.org/cgi-bin/carddisp.pl?gene=DAXX
ORAI1	ORAI Calcium Release-Activated Calcium Modulator 1	Protein Coding	Q96D31	48	GC12P129107	0.204691	https://www.genecards.org/cgi-bin/carddisp.pl?gene=ORAI1
SDC2	Syndecan 2	Protein Coding	P34741	48	GC08P096499	0.204691	https://www.genecards.org/cgi-bin/carddisp.pl?gene=SDC2
SMURF1	SMAD Specific E3 Ubiquitin Protein Ligase 1	Protein Coding	Q9HCE7	48	GC07M099027	0.204691	https://www.genecards.org/cgi-bin/carddisp.pl?gene=SMURF1
DAB2	DAB Adaptor Protein 2	Protein Coding	P98082	47	GC05M039371	0.204691	https://www.genecards.org/cgi-bin/carddisp.pl?gene=DAB2
CTNND2	Catenin Delta 2	Protein Coding	Q9UQB3	46	GC05M010971	0.204691	https://www.genecards.org/cgi-bin/carddisp.pl?gene=CTNND2
CYB5R1	Cytochrome B5 Reductase 1	Protein Coding	Q9UHQ9	46	GC01M202964	0.204691	https://www.genecards.org/cgi-bin/carddisp.pl?gene=CYB5R1
MARK1	Microtubule Affinity Regulating Kinase 1	Protein Coding	Q9P0L2	46	GC01P220528	0.204691	https://www.genecards.org/cgi-bin/carddisp.pl?gene=MARK1
MUC5B	Mucin 5B, Oligomeric Mucus/Gel-Forming	Protein Coding	Q9HC84	46	GC11P004493	0.204691	https://www.genecards.org/cgi-bin/carddisp.pl?gene=MUC5B
RPS29	Ribosomal Protein S29	Protein Coding	P62273	46	GC14M049570	0.204691	https://www.genecards.org/cgi-bin/carddisp.pl?gene=RPS29
C4BPA	Complement Component 4 Binding Protein Alpha	Protein Coding	P04003	45	GC01P207105	0.204691	https://www.genecards.org/cgi-bin/carddisp.pl?gene=C4BPA

MTSS1	MTSS I-BAR Domain Containing 1	Protein Coding	O43312	45	GC08M124550	0.204691	https://www.genecards.org/cgi-bin/carddisp.pl?gene=MTSS1
PAWR	Pro-Apoptotic WT1 Regulator	Protein Coding	Q96IZ0	45	GC12M079574	0.204691	https://www.genecards.org/cgi-bin/carddisp.pl?gene=PAWR
RNGTT	RNA Guanylyltransferase And 5'-Phosphatase	Protein Coding	O60942	45	GC06M088609	0.204691	https://www.genecards.org/cgi-bin/carddisp.pl?gene=RNGTT
CDC5L	Cell Division Cycle 5 Like	Protein Coding	Q99459	44	GC06P044387	0.204691	https://www.genecards.org/cgi-bin/carddisp.pl?gene=CDC5L
FUT9	Fucosyltransferase 9	Protein Coding	Q9Y231	44	GC06P096015	0.204691	https://www.genecards.org/cgi-bin/carddisp.pl?gene=FUT9
PPP2R3C	Protein Phosphatase 2 Regulatory Subunit B''Gamma	Protein Coding	Q969Q6	44	GC14M035085	0.204691	https://www.genecards.org/cgi-bin/carddisp.pl?gene=PPP2R3C
AANAT	Aralkylamine N-Acetyltransferase	Protein Coding	Q16613	43	GC17P076453	0.204691	https://www.genecards.org/cgi-bin/carddisp.pl?gene=AANAT
ATF6B	Activating Transcription Factor 6 Beta	Protein Coding	Q99941	43	GC06M032115	0.204691	https://www.genecards.org/cgi-bin/carddisp.pl?gene=ATF6B
CD7	CD7 Molecule	Protein Coding	P09564	43	GC17M082314	0.204691	https://www.genecards.org/cgi-bin/carddisp.pl?gene=CD7
GPR84	G Protein-Coupled Receptor 84	Protein Coding	Q9NQS5	43	GC12M055402	0.204691	https://www.genecards.org/cgi-bin/carddisp.pl?gene=GPR84
ID4	Inhibitor Of DNA Binding 4	Protein Coding	P47928	43	GC06P019837	0.204691	https://www.genecards.org/cgi-bin/carddisp.pl?gene=ID4
RIPOR2	RHO Family Interacting Cell Polarization Regulator 2	Protein Coding	Q9Y4F9	43	GC06M024879	0.204691	https://www.genecards.org/cgi-bin/carddisp.pl?gene=RIPOR2

KIF20B	Kinesin Family Member 20B	Protein Coding	Q96Q89	42	GC10P089701	0.204691	https://www.genecards.org/cgi-bin/carddisp.pl?gene=KIF20B
TNFAIP8	TNF Alpha Induced Protein 8	Protein Coding	O95379	42	GC05P119268	0.204691	https://www.genecards.org/cgi-bin/carddisp.pl?gene=TNFAIP8
CCL1	C-C Motif Chemokine Ligand 1	Protein Coding	P22362	41	GC17M063518	0.204691	https://www.genecards.org/cgi-bin/carddisp.pl?gene=CCL1
KCMF1	Potassium Channel Modulatory Factor 1	Protein Coding	Q9P0J7	41	GC02P084971	0.204691	https://www.genecards.org/cgi-bin/carddisp.pl?gene=KCMF1
NANOG	Nanog Homeobox	Protein Coding	Q9H9S0	41	GC12P007787	0.204691	https://www.genecards.org/cgi-bin/carddisp.pl?gene=NANOG
PRPS1L1	Phosphoribosyl Pyrophosphate Synthetase 1 Like 1	Protein Coding	P21108	41	GC07M018026	0.204691	https://www.genecards.org/cgi-bin/carddisp.pl?gene=PRPS1L1
WDR72	WD Repeat Domain 72	Protein Coding	Q3MJ13	41	GC15M140464	0.204691	https://www.genecards.org/cgi-bin/carddisp.pl?gene=WDR72
CENPM	Centromere Protein M	Protein Coding	Q9NSP4	40	GC22M070440	0.204691	https://www.genecards.org/cgi-bin/carddisp.pl?gene=CENPM
SAMD3	Sterile Alpha Motif Domain Containing 3	Protein Coding	Q8N6K7	39	GC06M130144	0.204691	https://www.genecards.org/cgi-bin/carddisp.pl?gene=SAMD3
ZNF264	Zinc Finger Protein 264	Protein Coding	O43296	38	GC19P057191	0.204691	https://www.genecards.org/cgi-bin/carddisp.pl?gene=ZNF264
MIR17HG	MiR-17-92a-1 Cluster Host Gene	RNA Gene	Q75NE6	28	GC13P091347	0.204691	https://www.genecards.org/cgi-bin/carddisp.pl?gene=MIR17HG
ZNF503-AS2	ZNF503 Antisense RNA 2	RNA Gene	A6NEH8	23	GC10P075401	0.204691	https://www.genecards.org/cgi-bin/carddisp.pl?gene=ZNF503-AS2

PSORS1C3	Psoriasis Susceptibility 1 Candidate 3	RNA Gene		22	GC06M083897	0.204691	https://www.genecards.org/cgi-bin/carddisp.pl?gene=PSORS1C3
HAS2-AS1	HAS2 Antisense RNA 1	RNA Gene		20	GC08P121639	0.204691	https://www.genecards.org/cgi-bin/carddisp.pl?gene=HAS2-AS1
HCG27	HLA Complex Group 27	RNA Gene		20	GC06P031197	0.204691	https://www.genecards.org/cgi-bin/carddisp.pl?gene=HCG27
RN7SL1	RNA Component Of Signal Recognition Particle 7SL1	RNA Gene		20	GC14P049546	0.204691	https://www.genecards.org/cgi-bin/carddisp.pl?gene=RN7SL1
LINC01133	Long Intergenic Non-Protein Coding RNA 1133	RNA Gene		17	GC01P159959	0.204691	https://www.genecards.org/cgi-bin/carddisp.pl?gene=LINC01133
MIR1305	MicroRNA 1305	RNA Gene		17	GC04P182169	0.204691	https://www.genecards.org/cgi-bin/carddisp.pl?gene=MIR1305
FAM225A	Family With Sequence Similarity 225 Member A	RNA Gene		16	GC09P113112	0.204691	https://www.genecards.org/cgi-bin/carddisp.pl?gene=FAM225A
LINC00607	Long Intergenic Non-Protein Coding RNA 607	RNA Gene		16	GC02M215611	0.204691	https://www.genecards.org/cgi-bin/carddisp.pl?gene=LINC00607
CYP2U1-AS1	CYP2U1 And SGMS2 Antisense RNA 1	RNA Gene		15	GC04M107866	0.204691	https://www.genecards.org/cgi-bin/carddisp.pl?gene=CYP2U1-AS1
MHRT	Myosin Heavy Chain Associated RNA Transcript	RNA Gene		15	GC14P041083	0.204691	https://www.genecards.org/cgi-bin/carddisp.pl?gene=MHRT
MIR222HG	MiR222/221 Cluster Host Gene	RNA Gene		15	GC0XM045745	0.204691	https://www.genecards.org/cgi-bin/carddisp.pl?gene=MIR222HG
MIR644A	MicroRNA 644a	RNA Gene		13	GC20P036256	0.204691	https://www.genecards.org/cgi-bin/carddisp.pl?gene=MIR644A

MIR4778	MicroRNA 4778	RNA Gene		11	GC02M066358	0.204691	https://www.genecards.org/cgi-bin/carddisp.pl?gene=MIR4778
MYOPARR	Myogenin Promoter Associated Myogenic Regulatory Antisense Long Non Coding RNA	RNA Gene		9	GC01P203109	0.204691	https://www.genecards.org/cgi-bin/carddisp.pl?gene=MYOPARR
NONHSAG046336.2		RNA Gene		5	GC06P112086	0.204691	https://www.genecards.org/cgi-bin/carddisp.pl?gene=NONHSAG046336.2
RF03967-002		RNA Gene		3	GC19P094642	0.204691	https://www.genecards.org/cgi-bin/carddisp.pl?gene=RF03967-002

Supplementary table S5. Targets of genes associated in maternal obesity from OMIM (http://www.omim.org/)

Cytogenetic location	Gene/Locus	Gene/Locus name	Gene/Locus MIM number	Approved Symbol	Entrez Gene ID	Ensembl Gene ID	Phenotype	Phenotype MIM number	Inheritance	Mouse Gene (from MGI)
1p36	DEL1p36, C1DELp36	Chromosome 1p36 deletion syndrome, distal	607872				Chromosome 1p36 deletion syndrome, distal	607872	Isolated cases	
1p36.32	PEX10, NALD, PBD6A, PBD6B	Peroxisome biogenesis factor 10	602859	PEX10	5192	ENSG00000157911,ENST00000447513;;;ENST00000447513	Peroxisome biogenesis disorder 6A (Zellweger)	614870	Autosomal recessive	Pex10
1p36.32	PEX10, NALD, PBD6A, PBD6B	Peroxisome biogenesis factor 10	602859	PEX10	5192	ENSG00000157911,ENST00000447513;;;ENST00000447513	Peroxisome biogenesis disorder 6B	614871	Autosomal recessive	Pex10
1p36.32	PRDM16, MEL1, LVNC8, CMD1LL	PR domain-containing protein 16	605557	PRDM16	63976	ENSG00000142611,ENST00000270722;;;ENST00000270722	Cardiomyopathy, dilated, 1LL	615373	Autosomal dominant	Prdm16
1p36.32	PRDM16, MEL1, LVNC8, CMD1LL	PR domain-containing protein 16	605557	PRDM16	63976	ENSG00000142611,ENST00000270722;;;ENST00000270722	Left ventricular noncompaction 8	615373	Autosomal dominant	Prdm16

1p36.31	NPHP4, SLSN4	Nephrocystin 4	607215	NPHP4	261734	ENSG00000131697,ENST00000378156;;;ENST00000378156	Nephronophthisis 4	606966	Autosomal recessive	Nphp4
1p36.31	NPHP4, SLSN4	Nephrocystin 4	607215	NPHP4	261734	ENSG00000131697,ENST00000378156;;;ENST00000378156	Senior-Loken syndrome 4	606996	Autosomal recessive	Nphp4
1p36.31-p36.23	CAMTA1, KIAA0833, CECBA	Calmodulin-binding transcription activator 1	611501	CAMTA1	23261	ENSG00000171735,ENST00000303635;;;ENST00000303635	Cerebellar dysfunction with variable cognitive and behavioral abnormalities	614756	Autosomal dominant	Camta1
1p36.2	SCZD12	Schizophrenia 12	608543		619488		{Schizophrenia 12}	181500	Autosomal dominant	
1p36.23	PER3, FASPS3	Period circadian regulator 3	603427	PER3	8863	ENSG00000049246,ENST00000377532;;;ENST00000377532	?Advanced sleep phase syndrome, familial, 3	616882	Autosomal dominant	Per3
1p36.22	H6PD, GDH, G6PDH, CORTRD1	Hexose-6-phosphate dehydrogenase	138090	H6PD	9563	ENSG00000049239,ENST00000377403;;;ENST00000377403	Cortisone reductase deficiency 1	604931	Autosomal recessive	H6pd
1p36.22	KIF1B, CMT2A, CMT2A1, NBLST1	Kinesin family member 1B	605995	KIF1B	23095	ENSG00000054523,ENST00000676179;;;ENST00000676179	Charcot-Marie-Tooth disease, type 2A1	118210	Autosomal dominant	Kif1b

1p36.22	KIF1B, CMT2A, CMT2A1, NBLST1	Kinesin family member 1B	605995	KIF1B	23095	ENSG00000054523,ENST00000676179;;;ENST00000676179	{Neuroblastoma, susceptibility to, 1}	256700	Autosomal dominant; Somatic mutation	Kif1b
1p36.22	MTOR, FRAP1, SKS	Mechanistic target of rapamycin	601231	MTOR	2475	ENSG00000198793,ENST00000361445;;;ENST00000361445	Focal cortical dysplasia, type II, somatic	607341		Mtor
1p36.22	MTOR, FRAP1, SKS	Mechanistic target of rapamycin	601231	MTOR	2475	ENSG00000198793,ENST00000361445;;;ENST00000361445	Smith-Kingsmore syndrome	616638	Autosomal dominant	Mtor
1p36.22	ANGPTL7, CDT6	Angiopoietin-like 7	618517	ANGPTL7	10218	ENSG00000171819,ENST00000376819;;;ENST00000376819				
1p36.22	MTHFR	Methylenetetrahydrofolate reductase	607093	MTHFR	4524	ENSG00000177000,ENST00000376590;;;ENST00000376590	Homocystinuria due to MTHFR deficiency	236250	Autosomal recessive	Mthfr
1p36.22	MTHFR	Methylenetetrahydrofolate reductase	607093	MTHFR	4524	ENSG00000177000,ENST00000376590;;;ENST00000376590	{Neural tube defects, susceptibility to}	601634	Autosomal recessive	Mthfr
1p36.22	MTHFR	Methylenetetrahydrofolate reductase	607093	MTHFR	4524	ENSG00000177000,ENST000003765	{Schizophrenia, susceptibility to}	181500	Autosomal dominant	Mthfr

						90;;;ENST00000376590				
1p36.22	MTHFR	Methylenetetrahydrofolate reductase	607093	MTHFR	4524	ENSG00000177000,ENST00000376590;;;ENST00000376590	{Thromboembolism, susceptibility to}	188050	Autosomal dominant	Mthfr
1p36.22	MTHFR	Methylenetetrahydrofolate reductase	607093	MTHFR	4524	ENSG00000177000,ENST00000376590;;;ENST00000376590	{Vascular disease, susceptibility to}	Mthfr		
1p36.22	PLOD1, LH1, LLH, EDSKCL1	Procollagen-lysine, 2-oxoglutarate 5-dioxygenase (lysine hydroxylase)	153454	PLOD1	5351	ENSG00000083444,ENST00000196061;;;ENST00000196061	Ehlers-Danlos syndrome, kyphoscoliotic type, 1	225400	Autosomal recessive	Plod1
1p36.22	MFN2, KIAA0214, CMT2A2A, HMSN6A, CMT2A2B, MSL	Mitofusin 2	608507	MFN2	9927	ENSG00000116688,ENST00000235329;;;ENST00000235329	Charcot-Marie-Tooth disease, axonal, type 2A2A	609260	Autosomal dominant	Mfn2
1p36.22	MFN2, KIAA0214, CMT2A2A, HMSN6A, CMT2A2B, MSL	Mitofusin 2	608507	MFN2	9927	ENSG00000116688,ENST00000235329;;;ENST00000235329	Charcot-Marie-Tooth disease, axonal, type 2A2B	617087	Autosomal recessive	Mfn2
1p36.22	MFN2, KIAA0214, CMT2A2A, HMSN6A,	Mitofusin 2	608507	MFN2	9927	ENSG00000116688,ENST000002353	Hereditary motor and sensory neuropathy VIA	601152	Autosomal dominant	Mfn2

	CMT2A2B, MSL					29;;;ENST00 000235329				
1p3 6.22	MFN2, KIAA0214, CMT2A2A, HMSN6A, CMT2A2B, MSL	Mitofusin 2	6085 07	MF N2	99 27	ENSG00000 116688,ENS T000002353 29;;;ENST00 000235329	Lipomatosis, multiple symmetric, with or without peripheral neuropathy	1518 00	Autosomal recessive	Mfn2
1p3 6.21	CELA2A, ELA2A, AOMS4	Chymotrypsin-like elastase family, member 2A	6094 43	CE LA 2A	63 03 6	ENSG00000 142615,ENS T000003596 21;;;ENST00 000359621	Abdominal obesity-metabolic syndrome 4	6186 20	Autosomal dominant	Cela2a
1p3 6.21 - p36. 13	SPEN, MINT, SHARP, HIAA0929, RATARS	Spen family transcriptional repressor	6134 84	SP EN	23 01 3	ENSG00000 065526,ENS T000003757 59;;;ENST00 000375759	Radio-Tartaglia syndrome	6193 12	Autosomal dominant	Spen
1p3 6.13	CLCNKA	Chloride channel, kidney, A	6020 24	CL CN KA	11 87	ENSG00000 186510,ENS T000003314 33;;;ENST00 000331433	Bartter syndrome, type 4b, digenic	6130 90	Digenic recessive	Clcnka,Clcnk b
1p3 6.13	CLCNKB	Chloride channel, kidney, B	6020 23	CL CN KB	11 88	ENSG00000 184908,ENS T000003756 79;;;ENST00 000375679	Bartter syndrome, type 3	6073 64	Autosomal recessive	Clcnka,Clcnk b
1p3 6.13	CLCNKB	Chloride channel, kidney, B	6020 23	CL CN KB	11 88	ENSG00000 184908,ENS T000003756 79;;;ENST00 000375679	Bartter syndrome, type 4b, digenic	6130 90	Digenic recessive	Clcnka,Clcnk b

1p3 6.13	SDHB, SDH2, SDHIP, PGL4, MC2DN4	Succinate dehydrogenase complex, subunit B, iron sulfur (Ip)	1854 70	SD HB	63 90	ENSG00000 117118,ENS T000003754 99;;;ENST00 000375499	Gastrointestinal stromal tumor	6067 64	Autosomal dominant; Isolated cases	Sdhb
1p3 6.13	SDHB, SDH2, SDHIP, PGL4, MC2DN4	Succinate dehydrogenase complex, subunit B, iron sulfur (Ip)	1854 70	SD HB	63 90	ENSG00000 117118,ENS T000003754 99;;;ENST00 000375499	Mitochondrial complex II deficiency, nuclear type 4	6192 24	Autosomal recessive	Sdhb
1p3 6.13	SDHB, SDH2, SDHIP, PGL4, MC2DN4	Succinate dehydrogenase complex, subunit B, iron sulfur (Ip)	1854 70	SD HB	63 90	ENSG00000 117118,ENS T000003754 99;;;ENST00 000375499	Paraganglioma and gastric stromal sarcoma	6068 64		Sdhb
1p3 6.13	SDHB, SDH2, SDHIP, PGL4, MC2DN4	Succinate dehydrogenase complex, subunit B, iron sulfur (Ip)	1854 70	SD HB	63 90	ENSG00000 117118,ENS T000003754 99;;;ENST00 000375499	Paragangliomas 4	1153 10	Autosomal dominant	Sdhb
1p3 6.13	SDHB, SDH2, SDHIP, PGL4, MC2DN4	Succinate dehydrogenase complex, subunit B, iron sulfur (Ip)	1854 70	SD HB	63 90	ENSG00000 117118,ENS T000003754 99;;;ENST00 000375499	Pheochromocytoma	1713 00	Autosomal dominant	Sdhb
1p3 6.13	PADI6, OZEMA16	Peptidylarginine deiminase, type VI	6103 63	PA DI6	35 32 38	ENSG00000 276747,ENS T000006196 09;;;ENST00 000619609	Oocyte/zygote/embryo maturation arrest 16	6172 34	Autosomal recessive	Padi6
1p3 6.13	PLA2G2E	Secretory phospholipase A2, group IIE	6183 20	PL A2	30 81 4	ENSG00000 188784,ENS T000003751				

				G2 E		16;;;ENST00 000375116				
1p3 6.12	ECE1	Endothelin converting enzyme 1	6004 23	EC E1	18 89	ENSG00000 117298,ENS T000003748 93;;;ENST00 000374893	?Hirschsprung disease, cardiac defects, and autonomic dysfunction	6138 70	Autosomal dominant	Ece1
1p3 6.12	ECE1	Endothelin converting enzyme 1	6004 23	EC E1	18 89	ENSG00000 117298,ENS T000003748 93;;;ENST00 000374893	{Hypertension, essential, susceptibility to}	1455 00	Multifactoria l	Ece1
1p3 6.12	ALPL, HOPS, TNSALP, HPPA, HPPO, HPPI, HPPC	Alkaline phosphatase, liver/bone/kidney	1717 60	AL PL	24 9	ENSG00000 162551,ENS T000003748 40;;;ENST00 000374840	Hypophosphatasia, adult	1463 00	Autosomal dominant; Autosomal recessive	Alpl
1p3 6.12	ALPL, HOPS, TNSALP, HPPA, HPPO, HPPI, HPPC	Alkaline phosphatase, liver/bone/kidney	1717 60	AL PL	24 9	ENSG00000 162551,ENS T000003748 40;;;ENST00 000374840	Hypophosphatasia, childhood	2415 10	Autosomal recessive	Alpl
1p3 6.12	ALPL, HOPS, TNSALP, HPPA, HPPO, HPPI, HPPC	Alkaline phosphatase, liver/bone/kidney	1717 60	AL PL	24 9	ENSG00000 162551,ENS T000003748 40;;;ENST00 000374840	Hypophosphatasia, infantile	2415 00	Autosomal recessive	Alpl
1p3 6.12	ALPL, HOPS, TNSALP, HPPA, HPPO, HPPI, HPPC	Alkaline phosphatase, liver/bone/kidney	1717 60	AL PL	24 9	ENSG00000 162551,ENS T000003748 40;;;ENST00 000374840	Odontohypophosphatasia	1463 00	Autosomal dominant; Autosomal recessive	Alpl

1p36.12	USP48, USP31, DFNA85	Ubiquitin-specific peptidase 48	617445	USP48	84196	ENSG00000090686,ENST00000308271;;;ENST00000308271	Deafness, autosomal dominant 85	620227	Autosomal dominant	Usp48
1p36.12	HSPG2, PLC, SJS, SJA, SJS1	Heparan sulfate proteoglycan of basement membrane (perlecan)	142461	HSPG2	3339	ENSG00000142798,ENST00000374695;;;ENST00000374695	Dyssegmental dysplasia, Silverman-Handmaker type	224410	Autosomal recessive	Hspg2
1p36.12	HSPG2, PLC, SJS, SJA, SJS1	Heparan sulfate proteoglycan of basement membrane (perlecan)	142461	HSPG2	3339	ENSG00000142798,ENST00000374695;;;ENST00000374695	Schwartz-Jampel syndrome, type 1	255800	Autosomal recessive	Hspg2
1p36.11	HMGCL	3-hydroxy-3-methylglutaryl-Coenzyme A lyase	613898	HMGCL	3155	ENSG00000117305,ENST00000374490;;;ENST00000374490	HMG-CoA lyase deficiency	246450	Autosomal recessive	Hmgcl
1p36.11	FUCA1	Fucosidase, alpha-L- 1, tissue	612280	FUCA1	2517	ENSG00000179163,ENST00000374479;;;ENST00000374479	Fucosidosis	230000	Autosomal recessive	Fuca1
1p36.11	PNRC2	Proline-rich nuclear receptor coactivator 2	611882	PNRC2	55629	ENSG00000189266,ENST00000334351;;;ENST00000334351				
1p36.11	RHD, HDFNRH	Rhesus system D polypeptide	111680	RHD	6007	ENSG00000187010,ENST000003286	[Blood group, RH system]	111690		Rhd

						64;;;ENST00 000328664				
1p3 6.11	RHD, HDFNRH	Rhesus system D polypeptide	1116 80	RH D	60 07	ENSG00000 187010,ENS T000003286 64;;;ENST00 000328664	{Hemolytic disease of fetus and newborn, RH-induced}	6194 62	Isolated cases	Rhd
1p3 6.11	STMN1, LAP18, SMN	Stathmin	1514 42	ST M N1	39 25	ENSG00000 117632,ENS T000004557 85;;;ENST00 000455785				
1p3 6.11	SLC30A2, ZNT2, TNZD	Solute carrier family 30 (zinc transporter), member 2	6096 17	SL C30 A2	77 80	ENSG00000 158014,ENS T000003742 76;;;ENST00 000374276	Zinc deficiency, transient neonatal	6081 18	Autosomal dominant	Slc30a2
1p3 6.11	NR0B2, SHP	Nuclear receptor subfamily 0, group B, member 2	6046 30	NR 0B2	84 31	ENSG00000 131910,ENS T000002542 27;;;ENST00 000254227	Obesity, mild, early-onset	6016 65	Autosomal dominant; Autosomal recessive; Multifactoria l	Nr0b2
1p3 5.3	EYA3	EYA transcriptional coactivator and phosphatase 3	6016 55	EY A3	21 40	ENSG00000 158161,ENS T000003738 71;;;ENST00 000373871				
1p3 5.3	SEST2, HI95	Sestrin 2	6077 67	SES N2	83 66 7	ENSG00000 130766,ENS T000002530 63;;;ENST00 000253063				

1p35.3	YTHDF2	YTH N6-methyladenosine RNA-binding protein 2	610640	YTHDF2	51441	ENSG00000198492,ENST00000373812;;;ENST00000373812				
1p35.2	SDC3, SYND3, SDCN	Syndecan 3	186357	SDC3	9672	ENSG00000162512,ENST00000339394;;;ENST00000339394	{Obesity, association with}	601665	Autosomal dominant; Autosomal recessive; Multifactorial	Sdc3
1p35.2	CCDC28B, MGC1203	Coiled-coil domain-containing protein 28B	610162	CCDC28B	79140	ENSG00000160050,ENST00000373602;;;ENST00000373602	{Bardet-Biedl syndrome 1, modifier of}	209900	Autosomal recessive; Digenic recessive	Ccdc28b
1p35.2	LCK, IMD22	Lymphocyte-specific protein tyrosine kinase	153390	LCK	3932	ENSG00000182866,ENST00000336890;;;ENST00000336890	?Immunodeficiency 22	615758	Autosomal recessive	Lck
1p35.1	FNDC5, FRCP2	Fibronectin type III domain-containing protein 5	611906	FNDC5	252995	ENSG00000160097,ENST00000373471;;;ENST00000373471				
1p34.3	GJB4, CX30.3, EKVP2	Gap junction protein, beta-4	605425	GJB4	127534	ENSG00000189433,ENST00000339480;;;ENST00000339480	Erythrokeratodermia variabilis et progressiva 2	617524	Autosomal dominant	Gjb4

1p34.3	GJB3, CX31, DFNA2B, EKVP1	Gap junction protein, beta-3	603324	GJB3	2707	ENSG00000188910,ENST00000373366;;;ENST00000373366	Deafness, autosomal dominant 2B	612644	Autosomal dominant	Gjb3
1p34.3	GJB3, CX31, DFNA2B, EKVP1	Gap junction protein, beta-3	603324	GJB3	2707	ENSG00000188910,ENST00000373366;;;ENST00000373366	Deafness, autosomal dominant, with peripheral neuropathy	Gjb3		
1p34.3	GJB3, CX31, DFNA2B, EKVP1	Gap junction protein, beta-3	603324	GJB3	2707	ENSG00000188910,ENST00000373366;;;ENST00000373366	Deafness, autosomal recessive	Gjb3		
1p34.3	GJB3, CX31, DFNA2B, EKVP1	Gap junction protein, beta-3	603324	GJB3	2707	ENSG00000188910,ENST00000373366;;;ENST00000373366	Deafness, digenic, GJB2/GJB3	220290	Autosomal recessive; Digenic dominant	Gjb3
1p34.3	GJB3, CX31, DFNA2B, EKVP1	Gap junction protein, beta-3	603324	GJB3	2707	ENSG00000188910,ENST00000373366;;;ENST00000373366	Erythrokeratodermia variabilis et progressiva 1	133200	Autosomal dominant; Autosomal recessive	Gjb3
1p34.2	ZFP69, ZNF642	Zinc finger protein 69	617939	ZFP69	339559	ENSG00000187815,ENST00000372706;;;ENST00000372706				
1p34.2	ERMAP, SC, RD	Erythroblast membrane-associated protein	609017	ERMAP	114625	ENSG00000164010,ENST000003725	[Blood group, Radin]	111620		Ermap

						17;;;ENST00 000372517				
1p3 4.2	ERMAP, SC, RD	Erythroblast membrane-associated protein	6090 17	ER MA P	11 46 25	ENSG00000 164010,ENS T000003725 17;;;ENST00 000372517	[Blood group, Scianna system]	1117 50		Ermap
1p3 4.2	SLC2A1, GLUT1, HTLVR, DYT18, PED, GLUT1DS, EIG12, DYT9, SDCHCN	Solute carrier family 2 (facilitated glucose transporter), member 1	1381 40	SL C2 A1	65 13	ENSG00000 117394,ENS T000004262 63;;;ENST00 000426263	Dystonia 9	6010 42	Autosomal dominant	Slc2a1
1p3 4.2	SLC2A1, GLUT1, HTLVR, DYT18, PED, GLUT1DS, EIG12, DYT9, SDCHCN	Solute carrier family 2 (facilitated glucose transporter), member 1	1381 40	SL C2 A1	65 13	ENSG00000 117394,ENS T000004262 63;;;ENST00 000426263	GLUT1 deficiency syndrome 1, infantile onset, severe	6067 77	Autosomal dominant; Autosomal recessive	Slc2a1
1p3 4.2	SLC2A1, GLUT1, HTLVR, DYT18, PED, GLUT1DS, EIG12, DYT9, SDCHCN	Solute carrier family 2 (facilitated glucose transporter), member 1	1381 40	SL C2 A1	65 13	ENSG00000 117394,ENS T000004262 63;;;ENST00 000426263	GLUT1 deficiency syndrome 2, childhood onset	6121 26	Autosomal dominant	Slc2a1
1p3 4.2	SLC2A1, GLUT1, HTLVR, DYT18, PED, GLUT1DS,	Solute carrier family 2 (facilitated glucose transporter), member 1	1381 40	SL C2 A1	65 13	ENSG00000 117394,ENS T000004262 63;;;ENST00 000426263	Stomatin-deficient cryohydrocytosis with neurologic defects	6088 85	Autosomal dominant	Slc2a1

	EIG12, DYT9, SDCHCN									
1p34.2	SLC2A1, GLUT1, HTLVR, DYT18, PED, GLUT1DS, EIG12, DYT9, SDCHCN	Solute carrier family 2 (facilitated glucose transporter), member 1	138140	SLC2A1	6513	ENSG00000117394,ENST00000426263;;;ENST00000426263	{Epilepsy, idiopathic generalized, susceptibility to, 12}	614847	Autosomal dominant	Slc2a1
1p34.2	TIE, JTK14, TIE1, LMPHM11	Tyrosine kinase with immunoglobulin and epidermal growth factor homology domains	600222	TIE1	7075	ENSG00000066056,ENST00000372476;;;ENST00000372476	Lymphatic malformation 11	619401	Autosomal dominant	Tie1
1p34.2	PTPRF, LAR, BNAH2	Protein tyrosine phosphatase, receptor type, f polypeptide	179590	PTPRF	5792	ENSG00000142949,ENST00000359947;;;ENST00000359947	?Breasts and/or nipples, aplasia or hypoplasia of, 2	616001	Autosomal recessive	Ptprf
1p34.1	PTCH2	Patched 2	603673	PTCH2	8643	ENSG00000117425,ENST00000372192;;;ENST00000372192	Basal cell carcinoma, somatic	605462		Ptch2
1p34.1	PTCH2	Patched 2	603673	PTCH2	8643	ENSG00000117425,ENST00000372192;;;ENST00000372192	Medulloblastoma, somatic	155255		Ptch2
1p34.1	HPDL, NEDSWMA, SPG83	4-hydroxyphenylpyruvate dioxygenase-like	618994	HPDL	84842	ENSG00000186603,ENST000003348	Neurodevelopmental disorder with progressive spasticity and brain white matter abnormalities	619026	Autosomal recessive	Hpdl

						15;;;ENST00000334815				
1p34.1	HPDL, NEDSWMA, SPG83	4-hydroxyphenylpyruvate dioxygenase-like	618994	HPDL	84842	ENSG00000186603,ENST00000334815;;;ENST00000334815	Spastic paraplegia 83, autosomal recessive	619027	Autosomal recessive	Hpdl
1p34.1	RAD54L, HR54, HRAD54	RAD54 like	603615	RAD54L	8438	ENSG00000085999,ENST00000371975;;;ENST00000371975	Adenocarcinoma, colonic, somatic	Rad54l		
1p34.1	RAD54L, HR54, HRAD54	RAD54 like	603615	RAD54L	8438	ENSG00000085999,ENST00000371975;;;ENST00000371975	Lymphoma, non-Hodgkin, somatic	605027		Rad54l
1p34.1	RAD54L, HR54, HRAD54	RAD54 like	603615	RAD54L	8438	ENSG00000085999,ENST00000371975;;;ENST00000371975	{Breast cancer, invasive ductal}	114480	Autosomal dominant; Somatic mutation	Rad54l
1p33	FAAH	Fatty acid amide hydrolase	602935	FAAH	2166	ENSG00000117480,ENST00000243167;;;ENST00000243167	{Drug addiction, susceptibility to}	606581		Faah
1p32.3	TUT4, ZCCHC11, KIAA0191	Terminal uridylyl transferase 4	613692	TUT4	23318	ENSG00000134744,ENST00000257177;;;ENST00000257177				

1p32.3	LRP8, APOER2, MCI1	Low density lipoprotein receptor-related protein 8 (Apolipoprotein E receptor 2)	602600	LRP8	7804	ENSG00000157193,ENST00000306052;;;ENST00000306052	{Myocardial infarction, susceptibility to}	608446		Lrp8
1p32.3	GLIS1	Glis family zinc finger protein 1	610378	GLIS1	148979	ENSG00000174332,ENST00000628545;;;ENST00000628545				
1p32.3	DIO1, TXDI1, THMA2	Deiodinase, iodothyronine, type I	147892	DIO1	1733	ENSG00000211452,ENST00000361921;;;ENST00000361921	Thyroid hormone metabolism, abnormal, 2	619855	Autosomal dominant	Dio1
1p32.3	ACOT11, THEA, BFIT, BFIT1, BFIT2, KIAA0707	Acyl-CoA thioesterase 11	606803	ACOT11	26027	ENSG00000162390,ENST00000343744;;;ENST00000343744				
1p32.3	BSND	Barttin	606412	BSND	7809	ENSG00000162399,ENST00000651561;;;ENST00000651561	Bartter syndrome, type 4a	602522	Autosomal recessive	Bsnd
1p32.3	BSND	Barttin	606412	BSND	7809	ENSG00000162399,ENST00000651561;;;ENST00000651561	Sensorineural deafness with mild renal dysfunction	602522	Autosomal recessive	Bsnd
1p32.3	PCSK9, NARC1, HCHOLA3,	Proprotein convertase, subtilisin/kexin-type, 9	607786	PCSK9	255738	ENSG00000169174,ENST000003021	Hypercholesterolemia, familial, 3	603776	Autosomal dominant	Pcsk9

	FH3, LDLCQ1, FHCL3					18;;;ENST00 000302118				
1p3 2.3	PCSK9, NARC1, HCHOLA3, FH3, LDLCQ1, FHCL3	Proprotein convertase, subtilisin/kexin-type, 9	6077 86	PC SK 9	25 57 38	ENSG00000 169174,ENS T000003021 18;;;ENST00 000302118	{Low density lipoprotein cholesterol level QTL 1}	6037 76	Autosomal dominant	Pcsk9
1p3 2.2-p32. 1	OMA1, MPRP1	OMA1 zinc metallopeptidase	6170 81	O MA 1	11 52 09	ENSG00000 162600,ENS T000003712 26;;;ENST00 000371226				
1p3 1.3	OPHLC, C1DUPp31.3, DUP1p31.3	Omphalocele due to duplication of 1p31.3	1647 50				Omphalocele due to duplication of 1p31.3	1647 50	Isolated cases	
1p3 1.3	PGM1, GSD14, CDG1T	Phosphoglucomutase-1	1719 00	PG M1	52 36	ENSG00000 079739,ENS T000003710 84;;;ENST00 000371084	Congenital disorder of glycosylation, type It	6149 21	Autosomal recessive	Pgm1
1p3 1.3	LEPR, OBR, LEPRD	Leptin receptor	6010 07	LE PR	39 53	ENSG00000 116678,ENS T000003495 33;;;ENST00 000349533	Obesity, morbid, due to leptin receptor deficiency	6149 63	Autosomal recessive	Lepr
1p3 1.3	LEPROT, OBRGRP	Leptin receptor overlapping transcript	6134 61	LE PR OT	54 74 1	ENSG00000 213625,ENS T000003710 65;;;ENST00 000371065				
1p3 1.3	SGIP1	SH3-domain GRB2-like (endophilin)-interacting protein 1	6115 40	SGI P1	84 25 1	ENSG00000 118473,ENS T000003710				

						37;;;ENST00000371037				
1p31.3	GNG12AS1	GNG12 antisense RNA 1	615406	GNG12-AS1	100289178	ENSG00000232284,ENST00000420587				
1p31.3	DIRAS3, ARHI	DIRAS family, GTP-binding RAS-like protein 3	605193	DIRAS3	9077	ENSG00000162595,ENST00000646789;;;ENST00000646789				
1p31.3	RPE65, RP20, LCA2	RPE65 retinoid isomerohydrolase	180069	RPE65	6121	ENSG00000116745,ENST00000262340;;;ENST00000262340	Leber congenital amaurosis 2	204100	Autosomal recessive	Rpe65
1p31.3	RPE65, RP20, LCA2	RPE65 retinoid isomerohydrolase	180069	RPE65	6121	ENSG00000116745,ENST00000262340;;;ENST00000262340	Retinitis pigmentosa 20	613794	Autosomal recessive	Rpe65
1p31.3	RPE65, RP20, LCA2	RPE65 retinoid isomerohydrolase	180069	RPE65	6121	ENSG00000116745,ENST00000262340;;;ENST00000262340	Retinitis pigmentosa 87 with choroidal involvement	618697	Autosomal dominant	Rpe65
1p31.1	PTGER3, EP3	Prostaglandin E receptor 3, EP3 subtype	176806	PTGER3	5733	ENSG00000050628,ENST00000306666;;;ENST00000306666				

1p31.1	ZRANB2, ZNF265, ZIS	Zinc finger RANBP2-type domain-containing protein 2	604347	ZRANB2	9406	ENSG00000132485,ENST00000370920;;;ENST00000370920				
1p31.1	TNNI3K, CCDD	TNNI3-interacting kinase	613932	TNNI3K	51086	ENSG00000116783,ENST00000326637;;;ENST00000326637	Cardiac conduction disease with or without dilated cardiomyopathy	616117	Autosomal dominant	Tnni3k
1p31.1	MSH4, POF20, SPGF2	mutS homolog 4	602105	MSH4	4438	ENSG00000057468,ENST00000263187;;;ENST00000263187	Premature ovarian failure 20	619938	Autosomal recessive	Msh4
1p31.1	MSH4, POF20, SPGF2	mutS homolog 4	602105	MSH4	4438	ENSG00000057468,ENST00000263187;;;ENST00000263187	Spermatogenic failure 2	108420	Autosomal recessive	Msh4
1p22.3	BCL10, IMD37	B-cell leukemia/lymphoma 10	603517	BCL10	8915	ENSG00000142867,ENST00000648566;;;ENST00000648566	?Immunodeficiency 37	616098	Autosomal recessive	Bcl10
1p22.3	BCL10, IMD37	B-cell leukemia/lymphoma 10	603517	BCL10	8915	ENSG00000142867,ENST00000648566;;;ENST00000648566	Lymphoma, MALT, somatic	137245		Bcl10
1p22.3	BCL10, IMD37	B-cell leukemia/lymphoma 10	603517	BCL10	8915	ENSG00000142867,ENST000006485	{Lymphoma, follicular, somatic}	605027		Bcl10

						66;;;ENST00000648566				
1p22.3	BCL10, IMD37	B-cell leukemia/lymphoma 10	603517	BCL10	8915	ENSG00000142867,ENST00000648566;;;ENST00000648566	{Male germ cell tumor, somatic}	273300		Bcl10
1p22.3	BCL10, IMD37	B-cell leukemia/lymphoma 10	603517	BCL10	8915	ENSG00000142867,ENST00000648566;;;ENST00000648566	{Mesothelioma, somatic}	156240		Bcl10
1p22.3	BCL10, IMD37	B-cell leukemia/lymphoma 10	603517	BCL10	8915	ENSG00000142867,ENST00000648566;;;ENST00000648566	{Sezary syndrome, somatic}	Bcl10		
1p22.1	ABCA4, ABCR, STGD1, FFM, RP19, CORD3, ARMD2	ATP-binding transporter, retina-specific	601691	ABCA4	24	ENSG00000198691,ENST00000370225;;;ENST00000370225	Cone-rod dystrophy 3	604116	Autosomal recessive	Abca4
1p22.1	ABCA4, ABCR, STGD1, FFM, RP19, CORD3, ARMD2	ATP-binding transporter, retina-specific	601691	ABCA4	24	ENSG00000198691,ENST00000370225;;;ENST00000370225	Fundus flavimaculatus	248200	Autosomal recessive	Abca4
1p22.1	ABCA4, ABCR, STGD1, FFM, RP19, CORD3, ARMD2	ATP-binding transporter, retina-specific	601691	ABCA4	24	ENSG00000198691,ENST00000370225;;;ENST00000370225	Retinal dystrophy, early-onset severe	248200	Autosomal recessive	Abca4

1p2 2.1	ABCA4, ABCR, STGD1, FFM, RP19, CORD3, ARMD2	ATP-binding transporter, retina-specific	6016 91	AB CA 4	24	ENSG00000 198691,ENS T000003702 25;;;ENST00 000370225	Retinitis pigmentosa 19	6017 18	Autosomal recessive	Abca4
1p2 2.1	ABCA4, ABCR, STGD1, FFM, RP19, CORD3, ARMD2	ATP-binding transporter, retina-specific	6016 91	AB CA 4	24	ENSG00000 198691,ENS T000003702 25;;;ENST00 000370225	Stargardt disease 1	2482 00	Autosomal recessive	Abca4
1p2 2.1	ABCA4, ABCR, STGD1, FFM, RP19, CORD3, ARMD2	ATP-binding transporter, retina-specific	6016 91	AB CA 4	24	ENSG00000 198691,ENS T000003702 25;;;ENST00 000370225	{Macular degeneration, age-related, 2}	1538 00	Autosomal dominant	Abca4
1p2 1.3	F3, TFA	Coagulation factor III	1343 90	F3	21 52	ENSG00000 117525,ENS T000003340 47;;;ENST00 000334047				
1p2 1.2	AGL, GDE	Amylo-1,6-glucosidase, 4-alpha-glucanotransferase (glycogen debranching enzyme)	6108 60	AG L	17 8	ENSG00000 162688,ENS T000003619 15;;;ENST00 000361915	Glycogen storage disease IIIa	2324 00	Autosomal recessive	Agl
1p2 1.2	AGL, GDE	Amylo-1,6-glucosidase, 4-alpha-glucanotransferase (glycogen debranching enzyme)	6108 60	AG L	17 8	ENSG00000 162688,ENS T000003619 15;;;ENST00 000361915	Glycogen storage disease IIIb	2324 00	Autosomal recessive	Agl
1p2 1.2	DBT, BCATE2	Dihydrolipoamide branched chain transacylase (E2	2486 10	DB T	16 29	ENSG00000 137992,ENS T000003701	Maple syrup urine disease, type II	2486 00	Autosomal recessive	Dbt

		component of branched chain keto acid dehydrogenase complex)				32;;;ENST00000370132				
1p21.1	COL11A1, STL2, DFNA37	Collagen XI, alpha-1 polypeptide	120280	COL11A1	1301	ENSG00000060718,ENST00000370096;;;ENST00000370096	Deafness, autosomal dominant 37	618533	Autosomal dominant	Col11a1
1p21.1	COL11A1, STL2, DFNA37	Collagen XI, alpha-1 polypeptide	120280	COL11A1	1301	ENSG00000060718,ENST00000370096;;;ENST00000370096	Fibrochondrogenesis 1	228520	Autosomal recessive	Col11a1
1p21.1	COL11A1, STL2, DFNA37	Collagen XI, alpha-1 polypeptide	120280	COL11A1	1301	ENSG00000060718,ENST00000370096;;;ENST00000370096	Marshall syndrome	154780	Autosomal dominant	Col11a1
1p21.1	COL11A1, STL2, DFNA37	Collagen XI, alpha-1 polypeptide	120280	COL11A1	1301	ENSG00000060718,ENST00000370096;;;ENST00000370096	Stickler syndrome, type II	604841	Autosomal dominant	Col11a1
1p21.1	COL11A1, STL2, DFNA37	Collagen XI, alpha-1 polypeptide	120280	COL11A1	1301	ENSG00000060718,ENST00000370096;;;ENST00000370096	{Lumbar disc herniation, susceptibility to}	603932		Col11a1
1p21.1	RNPC3, SNRNP65, KIAA1839, CPHD7	RNA-binding region-containing protein 3	618016	RNPC3	55599	ENSG00000185946,ENST000004238	Pituitary hormone deficiency, combined or isolated, 7	618160	Autosomal recessive	Rnpc3

						55;;;ENST00000423855				
1p21.1	AMY2B	Amylase, pancreatic, alpha-2B	104660	AMY2B	280	ENSG00000240038,ENST00000684275;;;ENST00000684275				
1p21.1	AMY2A	Amylase, pancreatic, alpha-2A	104650	AMY2A	279	ENSG00000243480,ENST00000414303;;;ENST00000414303				
1p21.1	AMY1A	Amylase, salivary, alpha-1A	104700	AMY1A	276	ENSG00000237763,ENST00000370083;;;ENST00000370083				
1p21.1	AMY1B	Amylase, salivary, alpha-1B	104701	AMY1B	277	ENSG00000174876,ENST00000330330;;;ENST00000330330				
1p21.1	AMY1C	Amylase, salivary, alpha-1C	104702	AMY1C	278	ENSG00000187733,ENST00000622339;;;ENST00000622339				
1p13.3	GPSM2, LGN, PINS, DFNB82, CMCS	G-protein signaling modulator 2	609245	GPSM2	29899	ENSG00000121957,ENST00000264126;;;ENST00000264126	Chudley-McCullough syndrome	604213	Autosomal recessive	Gpsm2

1p13.3	MYBPHL	Myosin-binding protein H-like	619807	MYBPHL	343263	ENSG00000221986,ENST00000357155;;;ENST00000357155				
1p13.3	KCNA3	Potassium voltage-gated channel, shaker-related subfamily, member 3	176263	KCNA3	3738	ENSG00000177272,ENST00000369769;;;ENST00000369769				
1p13.2	PTPN22, PEP, PTPN8, LYP	Protein tyrosine phosphatase, nonreceptor-type 22	600716	PTPN22	26191	ENSG00000134242,ENST00000538253	{Diabetes, type 1, susceptibility to}	222100	Autosomal recessive	Ptpn22
1p13.2	PTPN22, PEP, PTPN8, LYP	Protein tyrosine phosphatase, nonreceptor-type 22	600716	PTPN22	26191	ENSG00000134242,ENST00000538253	{Rheumatoid arthritis, susceptibility to}	180300		Ptpn22
1p13.2	PTPN22, PEP, PTPN8, LYP	Protein tyrosine phosphatase, nonreceptor-type 22	600716	PTPN22	26191	ENSG00000134242,ENST00000538253	{Systemic lupus erythematosus susceptibility to}	152700	Autosomal dominant	Ptpn22
1p13.2	OLFML3, OLF44	Olfactomedin-like 3	610088	OLFML3	56944	ENSG00000116774,ENST00000320334;;;ENST00000320334				
1p13.2	NRAS, ALPS4, NS6, CMNS, NCMS	NRAS protooncogene, GTPase	164790	NRAS	4893	ENSG00000213281,ENST00000369535;;;ENST00000369535	?RAS-associated autoimmune lymphoproliferative syndrome type IV, somatic	614470		Nras

1p1 3.2	NRAS, ALPS4, NS6, CMNS, NCMS	NRAS protooncogene, GTPase	1647 90	NR AS	48 93	ENSG00000 213281,ENS T000003695 35;;;ENST00 000369535	Colorectal cancer, somatic	1145 00		Nras
1p1 3.2	NRAS, ALPS4, NS6, CMNS, NCMS	NRAS protooncogene, GTPase	1647 90	NR AS	48 93	ENSG00000 213281,ENS T000003695 35;;;ENST00 000369535	Epidermal nevus, somatic	1629 00		Nras
1p1 3.2	NRAS, ALPS4, NS6, CMNS, NCMS	NRAS protooncogene, GTPase	1647 90	NR AS	48 93	ENSG00000 213281,ENS T000003695 35;;;ENST00 000369535	Melanocytic nevus syndrome, congenital, somatic	1375 50		Nras
1p1 3.2	NRAS, ALPS4, NS6, CMNS, NCMS	NRAS protooncogene, GTPase	1647 90	NR AS	48 93	ENSG00000 213281,ENS T000003695 35;;;ENST00 000369535	Neurocutaneous melanosis, somatic	2494 00		Nras
1p1 3.2	NRAS, ALPS4, NS6, CMNS, NCMS	NRAS protooncogene, GTPase	1647 90	NR AS	48 93	ENSG00000 213281,ENS T000003695 35;;;ENST00 000369535	Noonan syndrome 6	6132 24	Autosomal dominant	Nras
1p1 3.2	NRAS, ALPS4, NS6, CMNS, NCMS	NRAS protooncogene, GTPase	1647 90	NR AS	48 93	ENSG00000 213281,ENS T000003695 35;;;ENST00 000369535	Schimmelpenning-Feuerstein-Mims syndrome, somatic mosaic	1632 00		Nras
1p1 3.2	NRAS, ALPS4, NS6, CMNS, NCMS	NRAS protooncogene, GTPase	1647 90	NR AS	48 93	ENSG00000 213281,ENS T000003695	Thyroid carcinoma, follicular, somatic	1884 70		Nras

						35;;;ENST00000369535				
1p13.1	VANGL1, STBM2	Vang-like 1	610132	VANGL1	81839	ENSG00000173218,ENST00000355485;;;ENST00000355485	Caudal regression syndrome	600145	Autosomal dominant	Vangl1
1p13.1	VANGL1, STBM2	Vang-like 1	610132	VANGL1	81839	ENSG00000173218,ENST00000355485;;;ENST00000355485	{Neural tube defects, susceptibility to}	182940	Autosomal dominant	Vangl1
1p13.1	CASQ2	Calsequestrin, fast-twitch, skeletal muscle-2	114251	CASQ2	845	ENSG00000118729,ENST00000261448;;;ENST00000261448	Ventricular tachycardia, catecholaminergic polymorphic, 2	611938	Autosomal recessive	Casq2
1p13.1	NHLH2, HEN2, HH27	Nescient helix loop helix 2	162361	NHLH2	4808	ENSG00000177551,ENST00000320238;;;ENST00000320238	?Hypogonadotropic hypogonadism 27 without anosmia	619755	Autosomal recessive	Nhlh2
1p13.1	TTF2	Transcription termination factor 2	604718	TTF2	8458	ENSG00000116830,ENST00000369466;;;ENST00000369466				
1p12	HSD3B2	Hydroxy-delta-5-steroid dehydrogenase, 3 beta- and steroid delta-isomerase, type 2 (adrenal, gonadal)	613890	HSD3B2	3284	ENSG00000203859,ENST00000369416;;;ENST00000369416	Adrenal hyperplasia, congenital, due to 3-beta-hydroxysteroid dehydrogenase 2 deficiency	201810	Autosomal recessive	Hsd3b1,Hsd3b2,Hsd3b3,Hsd3b4,Hsd3b5,Hsd3b6,Hsd3b8,Hsd3b9

1p12	NOTCH2, AGS2, HJCYS	Notch receptor 2	600275	NOTCH2	4853	ENSG00000134250,ENST00000256646;;;ENST00000256646	Alagille syndrome 2	610205	Autosomal dominant	Notch2
1p12	NOTCH2, AGS2, HJCYS	Notch receptor 2	600275	NOTCH2	4853	ENSG00000134250,ENST00000256646;;;ENST00000256646	Hajdu-Cheney syndrome	102500	Autosomal dominant	Notch2
1q21.1	DEL1q21, C1DELq21	Chromosome 1q21.1 deletion syndrome	612474				Chromosome 1q21.1 deletion syndrome	612474	Autosomal dominant; Isolated cases	
1q21.1	RBM8A, RBM8B, TAR, C1DELq21.1, DEL1q21.1	RNA-binding motif protein 8A	605313	RBM8A	9939	ENSG00000265241,ENST00000583313;;;ENST00000583313	Thrombocytopenia-absent radius syndrome	274000	Autosomal recessive	Rbm8a,Rbm8a2
1q21.1	HJV, HFE2A	Hemojuvelin	608374	HJV	148738	ENSG00000168509,ENST00000336751;;;ENST00000336751	Hemochromatosis, type 2A	602390	Autosomal recessive	Hjv
1q21.2	GJA5, CX40, ATFB11	Gap junction protein, alpha-5, 40kD (connexin 40)	121013	GJA5	2702	ENSG00000265107,ENST00000579774;;;ENST00000579774	Atrial fibrillation, familial, 11	614049	Autosomal dominant	Gja5
1q21.2	GJA5, CX40, ATFB11	Gap junction protein, alpha-5, 40kD (connexin 40)	121013	GJA5	2702	ENSG00000265107,ENST00000579774;;;ENST00000579774	Atrial standstill, digenic (GJA5/SCN5A)	108770	Autosomal dominant	Gja5

1q2 1.2	GJA8, CX50, CTRCT1, CZP1, CAE1	Gap junction membrane channel protein alpha-8 (connexin 50)	6008 97	GJ A8	27 03	ENSG00000 121634,ENS T000003692 35;;;ENST00 000369235	Cataract 1, multiple types	1162 00	Autosomal dominant	Gja8
1q2 1.2	SF3B4, SF3B49, SAP49, AFD1	Splicing factor 3B, subunit 4	6055 93	SF3 B4	10 26 2	ENSG00000 143368,ENS T000002716 28;;;ENST00 000271628	Acrofacial dysostosis 1, Nager type	1544 00	Autosomal dominant	Sf3b4
1q2 1.3	CTSK	Cathepsin K	6011 05	CT SK	15 13	ENSG00000 143387,ENS T000002716 51;;;ENST00 000271651	Pycnodysostosis	2658 00	Autosomal recessive	Ctsk
1q2 1.3	RORC, RORG, RZRG, IMD42	RAR-related orphan receptor C	6029 43	RO RC	60 97	ENSG00000 143365,ENS T000003182 47;;;ENST00 000318247	Immunodeficiency 42	6166 22	Autosomal recessive	Rorc
1q2 1.3	CRTC2, TORC2	CREB-regulated transcription coactivator 2	6089 72	CR TC 2	20 01 86	ENSG00000 160741,ENS T000003686 33;;;ENST00 000368633				
1q2 1.3	TPM3, NEM1, CMYP4A, CMYP4B	Tropomyosin 3	1910 30	TP M3	71 70	ENSG00000 143549,ENS T000006516 41;;;ENST00 000651641	Congenital myopathy 4A, autosomal dominant	2553 10	Autosomal dominant	Tpm3
1q2 1.3	TPM3, NEM1, CMYP4A, CMYP4B	Tropomyosin 3	1910 30	TP M3	71 70	ENSG00000 143549,ENS T000006516	Congenital myopathy 4B, autosomal recessive	6092 84	Autosomal recessive	Tpm3

						41;;;ENST00 000651641				
1q2 1.3	IL6R, IL6RQ, IL6QTL, HIES5	Interleukin-6 receptor	1478 80	IL6 R	35 70	ENSG00000 160712,ENS T000003684 85;;;ENST00 000368485	Hyper-IgE recurrent infection syndrome 5, autosomal recessive	6189 44	Autosomal recessive	Il6ra
1q2 1.3	IL6R, IL6RQ, IL6QTL, HIES5	Interleukin-6 receptor	1478 80	IL6 R	35 70	ENSG00000 160712,ENS T000003684 85;;;ENST00 000368485	[Interleukin 6, serum level of, QTL]	6147 52		Il6ra
1q2 1.3	IL6R, IL6RQ, IL6QTL, HIES5	Interleukin-6 receptor	1478 80	IL6 R	35 70	ENSG00000 160712,ENS T000003684 85;;;ENST00 000368485	[Interleukin-6 receptor, soluble, serum level of, QTL]	6146 89		Il6ra
1q2 1.3	ADAR, DRADA, DSH, DSRAD, IFI4, G1P1, AGS6	Adenosine deaminase, RNA-specific	1469 20	AD AR	10 3	ENSG00000 160710,ENS T000003684 74;;;ENST00 000368474	Aicardi-Goutieres syndrome 6	6150 10	Autosomal recessive	Adar
1q2 1.3	ADAR, DRADA, DSH, DSRAD, IFI4, G1P1, AGS6	Adenosine deaminase, RNA-specific	1469 20	AD AR	10 3	ENSG00000 160710,ENS T000003684 74;;;ENST00 000368474	Dyschromatosis symmetrica hereditaria	1274 00	Autosomal dominant	Adar
1q2 2	GBA	Glucosidase, acid beta	6064 63	GB A1	26 29	ENSG00000 177628,ENS T000003683 73;;;ENST00 000368373	Gaucher disease, perinatal lethal	6080 13	Autosomal recessive	Gba1

1q2 2	GBA	Glucosidase, acid beta	6064 63	GB A1	26 29	ENSG00000 177628,ENS T000003683 73;;;ENST00 000368373	Gaucher disease, type I	2308 00	Autosomal recessive	Gba1
1q2 2	GBA	Glucosidase, acid beta	6064 63	GB A1	26 29	ENSG00000 177628,ENS T000003683 73;;;ENST00 000368373	Gaucher disease, type II	2309 00	Autosomal recessive	Gba1
1q2 2	GBA	Glucosidase, acid beta	6064 63	GB A1	26 29	ENSG00000 177628,ENS T000003683 73;;;ENST00 000368373	Gaucher disease, type III	2310 00	Autosomal recessive	Gba1
1q2 2	GBA	Glucosidase, acid beta	6064 63	GB A1	26 29	ENSG00000 177628,ENS T000003683 73;;;ENST00 000368373	Gaucher disease, type IIIC	2310 05	Autosomal recessive	Gba1
1q2 2	GBA	Glucosidase, acid beta	6064 63	GB A1	26 29	ENSG00000 177628,ENS T000003683 73;;;ENST00 000368373	{Lewy body dementia, susceptibility to}	1277 50	Autosomal dominant	Gba1
1q2 2	GBA	Glucosidase, acid beta	6064 63	GB A1	26 29	ENSG00000 177628,ENS T000003683 73;;;ENST00 000368373	{Parkinson disease, late-onset, susceptibility to}	1686 00	Autosomal dominant; Multifactoria l	Gba1
1q2 2	PKLR, PK1	Pyruvate kinase, liver and RBC type	6097 12	PK LR	53 13	ENSG00000 143627,ENS T000003427	Adenosine triphosphate, elevated, of erythrocytes	1029 00	Autosomal dominant	Pklr

						41;;;ENST00 000342741				
1q2 2	PKLR, PK1	Pyruvate kinase, liver and RBC type	6097 12	PK LR	53 13	ENSG00000 143627,ENS T000003427 41;;;ENST00 000342741	Pyruvate kinase deficiency	2662 00	Autosomal recessive	Pklr
1q2 2	YY1AP1, YAP, HCCA2, GRNG	YY1 associated protein 1	6078 60	YY 1A P1	55 24 9	ENSG00000 163374,ENS T000003554 99;;;ENST00 000355499	Grange syndrome	6025 31	Autosomal recessive	Gon4l
1q2 2	LMNA, LMN1, EMD2, FPLD2, CMD1A, HGPS	Lamin A/C	1503 30	LM NA	40 00	ENSG00000 160789,ENS T000003683 00;;;ENST00 000368300	Cardiomyopathy, dilated, 1A	1152 00	Autosomal dominant	Lmna
1q2 2	LMNA, LMN1, EMD2, FPLD2, CMD1A, HGPS	Lamin A/C	1503 30	LM NA	40 00	ENSG00000 160789,ENS T000003683 00;;;ENST00 000368300	Charcot-Marie-Tooth disease, type 2B1	6055 88	Autosomal recessive	Lmna
1q2 2	LMNA, LMN1, EMD2, FPLD2, CMD1A, HGPS	Lamin A/C	1503 30	LM NA	40 00	ENSG00000 160789,ENS T000003683 00;;;ENST00 000368300	Emery-Dreifuss muscular dystrophy 2, autosomal dominant	1813 50	Autosomal dominant	Lmna
1q2 2	LMNA, LMN1, EMD2, FPLD2, CMD1A, HGPS	Lamin A/C	1503 30	LM NA	40 00	ENSG00000 160789,ENS T000003683 00;;;ENST00 000368300	Emery-Dreifuss muscular dystrophy 3, autosomal recessive	6165 16	Autosomal recessive	Lmna

1q22	LMNA, LMN1, EMD2, FPLD2, CMD1A, HGPS	Lamin A/C	150330	LMNA	4000	ENSG00000160789,ENST00000368300;;;ENST00000368300	Heart-hand syndrome, Slovenian type	610140	Autosomal dominant	Lmna
1q22	LMNA, LMN1, EMD2, FPLD2, CMD1A, HGPS	Lamin A/C	150330	LMNA	4000	ENSG00000160789,ENST00000368300;;;ENST00000368300	Hutchinson-Gilford progeria	176670	Autosomal dominant	Lmna
1q22	LMNA, LMN1, EMD2, FPLD2, CMD1A, HGPS	Lamin A/C	150330	LMNA	4000	ENSG00000160789,ENST00000368300;;;ENST00000368300	Lipodystrophy, familial partial, type 2	151660	Autosomal dominant	Lmna
1q22	LMNA, LMN1, EMD2, FPLD2, CMD1A, HGPS	Lamin A/C	150330	LMNA	4000	ENSG00000160789,ENST00000368300;;;ENST00000368300	Malouf syndrome	212112	Autosomal dominant	Lmna
1q22	LMNA, LMN1, EMD2, FPLD2, CMD1A, HGPS	Lamin A/C	150330	LMNA	4000	ENSG00000160789,ENST00000368300;;;ENST00000368300	Mandibuloacral dysplasia	248370	Autosomal recessive	Lmna
1q22	LMNA, LMN1, EMD2, FPLD2, CMD1A, HGPS	Lamin A/C	150330	LMNA	4000	ENSG00000160789,ENST00000368300;;;ENST00000368300	Muscular dystrophy, congenital	613205	Autosomal dominant	Lmna
1q22	LMNA, LMN1, EMD2, FPLD2, CMD1A, HGPS	Lamin A/C	150330	LMNA	4000	ENSG00000160789,ENST000003683	Restrictive dermopathy 2	619793		Lmna

						00;;;ENST00 000368300				
1q2 2	SLC25A44	Solute carrier family 25, member 44	6108 24	SL C25 A4 4	96 73	ENSG00000 160785,ENS T000003595 11;;;ENST00 000359511				
1q2 2	BGLAP	Bone gamma-carboxyglutamic acid protein	1122 60	BG LA P	63 2	ENSG00000 242252,ENS T000003682 72;;;ENST00 000368272				
1q2 3.1	NTRK1, TRKA	Neurotrophic tyrosine kinase, receptor, type 1	1913 15	NT RK 1	49 14	ENSG00000 198400,ENS T000005243 77;;;ENST00 000524377	Insensitivity to pain, congenital, with anhidrosis	2568 00	Autosomal recessive	Ntrk1
1q2 3.1	SPTA1, EL2, SPH3, HS3, HPP	Spectrin, alpha, erythrocytic-1	1828 60	SP TA 1	67 08	ENSG00000 163554,ENS T000006437 59;;;ENST00 000643759	Elliptocytosis-2	1306 00	Autosomal dominant	Spta1
1q2 3.1	SPTA1, EL2, SPH3, HS3, HPP	Spectrin, alpha, erythrocytic-1	1828 60	SP TA 1	67 08	ENSG00000 163554,ENS T000006437 59;;;ENST00 000643759	Pyropoikilocytosis	2661 40	Autosomal recessive	Spta1
1q2 3.1	SPTA1, EL2, SPH3, HS3, HPP	Spectrin, alpha, erythrocytic-1	1828 60	SP TA 1	67 08	ENSG00000 163554,ENS T000006437 59;;;ENST00 000643759	Spherocytosis, type 3	2709 70	Autosomal recessive	Spta1

1q2 3.2	ACKR1, DARC, FY, GPD, WBCQ1	Atypical chemokine receptor 1 (Duffy antigen receptor for chemokines)	6136 65	AC KR 1	25 32	ENSG00000 213088,ENS T000003681 22;;;ENST00 000368122	[Blood group, Duffy system]	1107 00	Autosomal dominant; Autosomal recessive	Ackr1
1q2 3.2	ACKR1, DARC, FY, GPD, WBCQ1	Atypical chemokine receptor 1 (Duffy antigen receptor for chemokines)	6136 65	AC KR 1	25 32	ENSG00000 213088,ENS T000003681 22;;;ENST00 000368122	[White blood cell count QTL]	6118 62	Autosomal recessive	Ackr1
1q2 3.2	ACKR1, DARC, FY, GPD, WBCQ1	Atypical chemokine receptor 1 (Duffy antigen receptor for chemokines)	6136 65	AC KR 1	25 32	ENSG00000 213088,ENS T000003681 22;;;ENST00 000368122	{Malaria, vivax, protection against}	6111 62		Ackr1
1q2 3.2	CRP	C-reactive protein	1232 60	CR P	14 01	ENSG00000 132693,ENS T000002550 30;;;ENST00 000255030				
1q2 3.2	ATP1A2, FHM2, MHP2, FARIMPD, DEE98	ATPase, Na+K+ transporting, alpha-2 polypeptide	1823 40	AT P1 A2	47 7	ENSG00000 018625,ENS T000003612 16;;;ENST00 000361216	Alternating hemiplegia of childhood 1	1042 90	Autosomal dominant	Atp1a2
1q2 3.2	ATP1A2, FHM2, MHP2, FARIMPD, DEE98	ATPase, Na+K+ transporting, alpha-2 polypeptide	1823 40	AT P1 A2	47 7	ENSG00000 018625,ENS T000003612 16;;;ENST00 000361216	Developmental and epileptic encephalopathy 98	6196 05	Autosomal dominant	Atp1a2
1q2 3.2	ATP1A2, FHM2, MHP2,	ATPase, Na+K+ transporting, alpha-2 polypeptide	1823 40	AT P1 A2	47 7	ENSG00000 018625,ENS T000003612	Fetal akinesia, respiratory insufficiency, microcephaly,	6196 02	Autosomal recessive	Atp1a2

	FARIMPD, DEE98					16;;;ENST00 000361216	polymicrogyria, and dysmorphic facies			
1q2 3.2	ATP1A2, FHM2, MHP2, FARIMPD, DEE98	ATPase, Na+K+ transporting, alpha-2 polypeptide	1823 40	AT P1 A2	47 7	ENSG00000 018625,ENS T000003612 16;;;ENST00 000361216	Migraine, familial basilar	6024 81	Autosomal dominant	Atp1a2
1q2 3.2	ATP1A2, FHM2, MHP2, FARIMPD, DEE98	ATPase, Na+K+ transporting, alpha-2 polypeptide	1823 40	AT P1 A2	47 7	ENSG00000 018625,ENS T000003612 16;;;ENST00 000361216	Migraine, familial hemiplegic, 2	6024 81	Autosomal dominant	Atp1a2
1q2 3.2	NCSTN	Nicastrin	6052 54	NC ST N	23 38 5	ENSG00000 162736,ENS T000002947 85;;;ENST00 000294785	Acne inversa, familial, 1	1426 90	Autosomal dominant	Ncstn
1q2 3.2	VANGL2, LTAP	VANGL planar cell polarity protein 2	6005 33	VA NG L2	57 21 6	ENSG00000 162738,ENS T000003680 61;;;ENST00 000368061	Neural tube defects	1829 40	Autosomal dominant	Vangl2
1q2 3.3	USF1, HYPLIP1	Upstream transcription factor 1	1915 23	US F1	73 91	ENSG00000 158773,ENS T000003680 21;;;ENST00 000368021	{Hyperlipidemia, familial combined, susceptibility to}	6024 91		Usf1
1q2 3.3	MPZ, CMT1B, CMTDID, DSS, CHN2	Myelin protein zero	1594 40	MP Z	43 59	ENSG00000 158887,ENS T000005333 57;;;ENST00 000533357	Charcot-Marie-Tooth disease, dominant intermediate D	6077 91	Autosomal dominant	Mpz

1q2 3.3	MPZ, CMT1B, CMTDID, DSS, CHN2	Myelin protein zero	1594 40	MP Z	43 59	ENSG00000 158887,ENS T000005333 57;;;ENST00 000533357	Charcot-Marie-Tooth disease, type 1B	1182 00	Autosomal dominant	Mpz
1q2 3.3	MPZ, CMT1B, CMTDID, DSS, CHN2	Myelin protein zero	1594 40	MP Z	43 59	ENSG00000 158887,ENS T000005333 57;;;ENST00 000533357	Charcot-Marie-Tooth disease, type 2I	6076 77	Autosomal dominant	Mpz
1q2 3.3	MPZ, CMT1B, CMTDID, DSS, CHN2	Myelin protein zero	1594 40	MP Z	43 59	ENSG00000 158887,ENS T000005333 57;;;ENST00 000533357	Charcot-Marie-Tooth disease, type 2J	6077 36	Autosomal dominant	Mpz
1q2 3.3	MPZ, CMT1B, CMTDID, DSS, CHN2	Myelin protein zero	1594 40	MP Z	43 59	ENSG00000 158887,ENS T000005333 57;;;ENST00 000533357	Dejerine-Sottas disease	1459 00	Autosomal dominant; Autosomal recessive	Mpz
1q2 3.3	MPZ, CMT1B, CMTDID, DSS, CHN2	Myelin protein zero	1594 40	MP Z	43 59	ENSG00000 158887,ENS T000005333 57;;;ENST00 000533357	Hypomyelinating neuropathy, congenital, 2	6181 84	Autosomal dominant	Mpz
1q2 3.3	MPZ, CMT1B, CMTDID, DSS, CHN2	Myelin protein zero	1594 40	MP Z	43 59	ENSG00000 158887,ENS T000005333 57;;;ENST00 000533357	Roussy-Levy syndrome	1808 00	Autosomal dominant	Mpz
1q2 3.3	SDHC, PGL3	Succinate dehydrogenase complex, subunit C,	6024 13	SD HC	63 91	ENSG00000 143252,ENS T000003679	Gastrointestinal stromal tumor	6067 64	Autosomal dominant;	Sdhc

		integral membrane protein, 15kD				75;;;ENST00000367975			Isolated cases	
1q23.3	SDHC, PGL3	Succinate dehydrogenase complex, subunit C, integral membrane protein, 15kD	602413	SDHC	6391	ENSG00000143252,ENST00000367975;;;ENST00000367975	Paraganglioma and gastric stromal sarcoma	606864		Sdhc
1q23.3	SDHC, PGL3	Succinate dehydrogenase complex, subunit C, integral membrane protein, 15kD	602413	SDHC	6391	ENSG00000143252,ENST00000367975;;;ENST00000367975	Paragangliomas 3	605373	Autosomal dominant	Sdhc
1q23.3	FCGR2A, IGFR2, CD32	Fc fragment of IgG receptor IIa	146790	FCGR2A	2212	ENSG00000143226,ENST00000271450;;;ENST00000271450	{Lupus nephritis, susceptibility to}	152700	Autosomal dominant	Fcgr2b,Fcgr3
1q23.3	FCGR2A, IGFR2, CD32	Fc fragment of IgG receptor IIa	146790	FCGR2A	2212	ENSG00000143226,ENST00000271450;;;ENST00000271450	{Malaria, severe, susceptibility to}	611162		Fcgr2b,Fcgr3
1q23.3	FCGR2A, IGFR2, CD32	Fc fragment of IgG receptor IIa	146790	FCGR2A	2212	ENSG00000143226,ENST00000271450;;;ENST00000271450	{Pseudomonas aeruginosa, susceptibility to chronic infection by, in cystic fibrosis}	219700	Autosomal recessive	Fcgr2b,Fcgr3
1q23.3	FCGR3B	Fc fragment of IgG receptor IIIb	610665	FCGR3B	2215	ENSG00000162747,ENST00000650385;;;ENST00000650385				

1q23.3	FCGR2B, CD32	Fc fragment of IgG receptor Ia	604590	FCGR2B	2213	ENSG00000072694,ENST00000358671;;;ENST00000358671	{Malaria, resistance to}	611162		Fcgr2b
1q23.3	FCGR2B, CD32	Fc fragment of IgG receptor Ia	604590	FCGR2B	2213	ENSG00000072694,ENST00000358671;;;ENST00000358671	{Systemic lupus erythematosus, susceptibility to}	152700	Autosomal dominant	Fcgr2b
1q23.3	HSD17B7	17-beta-hydroxysteroid dehydrogenase VII	606756	HSD17B7	51478	ENSG00000132196,ENST00000254521;;;ENST00000254521				
1q23.3	RGS5	Regulator of G protein signaling-5	603276	RGS5	8490	ENSG00000143248,ENST00000313961;;;ENST00000313961	[Blood pressure regulation QTL]	145500	Multifactorial	Rgs5
1q24.2	GPR161	G protein-coupled receptor 161	612250	GPR161	23432	ENSG00000143147,ENST00000682931;;;ENST00000682931	{Medulloblastoma predisposition syndrome}	155255	Autosomal dominant; Autosomal recessive; Somatic mutation	Gpr161
1q24.2	TBX19	T-box 19	604614	TBX19	9095	ENSG00000143178,ENST00000367821;;;ENST00000367821	Adrenocorticotropic hormone deficiency	201400	Autosomal recessive	Tbx19

1q24.2	ATP1B1	ATPase, Na+K+ transporting, beta-1 polypeptide	182330	ATP1B1	481	ENSG00000143153,ENST00000367815;;;ENST00000367815	[Blood pressure regulation QTL]	145500	Multifactorial	Atp1b1
1q24.2	F5, THPH2, RPRGL1	Coagulation factor V (proaccelerin, labile factor)	612309	F5	2153	ENSG00000198734,ENST00000367797;;;ENST00000367797	Factor V deficiency	227400	Autosomal recessive	F5
1q24.2	F5, THPH2, RPRGL1	Coagulation factor V (proaccelerin, labile factor)	612309	F5	2153	ENSG00000198734,ENST00000367797;;;ENST00000367797	Thrombophilia 2 due to activated protein C resistance	188055	Autosomal dominant	F5
1q24.2	F5, THPH2, RPRGL1	Coagulation factor V (proaccelerin, labile factor)	612309	F5	2153	ENSG00000198734,ENST00000367797;;;ENST00000367797	{Budd-Chiari syndrome}	600880	Autosomal recessive	F5
1q24.2	F5, THPH2, RPRGL1	Coagulation factor V (proaccelerin, labile factor)	612309	F5	2153	ENSG00000198734,ENST00000367797;;;ENST00000367797	{Pregnancy loss, recurrent, susceptibility to, 1}	614389	Autosomal dominant	F5
1q24.2	F5, THPH2, RPRGL1	Coagulation factor V (proaccelerin, labile factor)	612309	F5	2153	ENSG00000198734,ENST00000367797;;;ENST00000367797	{Stroke, ischemic, susceptibility to}	601367	Multifactorial	F5
1q24.2	F5, THPH2, RPRGL1	Coagulation factor V (proaccelerin, labile factor)	612309	F5	2153	ENSG00000198734,ENST000003677	{Thrombophilia, susceptibility to, due to factor V Leiden}	188055	Autosomal dominant	F5

						97;;;ENST00 000367797				
1q2 4.2	SELL, LYAM1, LAM1, LNHR	Selectin L (lymphocyte adhesion molecule 1)	1532 40	SE LL	64 02	ENSG00000 188404,ENS T000002361 47;;;ENST00 000236147				
1q2 4.3	MYOC, TIGR, GLC1A, JOAG, GPOA	Myocilin (trabecular meshwork-induced glucocorticoid response protein)	6016 52	MY OC	46 53	ENSG00000 034971,ENS T000000375 02;;;ENST00 000037502	Glaucoma 1A, primary open angle	1377 50	Autosomal dominant	Myoc
1q2 5.1	SERPINC1, AT3, AT3D, THPH7	Antithrombin III	1073 00	SE RPI NC 1	46 2	ENSG00000 117601,ENS T000003676 98;;;ENST00 000367698	Thrombophilia 7 due to antithrombin III deficiency	6131 18	Autosomal dominant; Autosomal recessive	Serpinc1
1q2 5.1- q25. 2	RFWD2, COP1	Ring finger- and WD repeat domain-containing protein 2	6080 67	CO P1	64 32 6	ENSG00000 143207,ENS T000003676 69;;;ENST00 000367669				
1q2 5.2	PAPPA2, PAPPE, SSDA	Pappalysin 2	6194 85	PA PP A2	60 67 6	ENSG00000 116183,ENS T000003676 62;;;ENST00 000367662	Short stature, Dauber-Argente type	6194 89	Autosomal recessive	Pappa2
1q2 5.2	PDCN, NPHS2, SRN1	Podocin	6047 66	NP HS 2	78 27	ENSG00000 116218,ENS T000003676 15;;;ENST00 000367615	Nephrotic syndrome, type 2	6009 95	Autosomal recessive	Nphs2

1q25.2	LHX4, CPHD4	LIM homeobox gene 4	602146	LHX4	89884	ENSG00000121454,ENST00000263726;;;ENST00000263726	Pituitary hormone deficiency, combined, 4	262700	Autosomal dominant	Lhx4
1q25.3	MR1, HLALS	Major histocompatibility complex, class I-related	600764	MR1	3140	ENSG00000153029,ENST00000367580;;;ENST00000367580				
1q25.3	NCF2	Neutrophil cytosolic factor-2, 65kD	608515	NCF2	4688	ENSG00000116701,ENST00000367535;;;ENST00000367535	Chronic granulomatous disease 2, autosomal recessive	233710	Autosomal recessive	Ncf2
1q25.3	EDEM3, C1orf22, CDG2V	Endoplasmic reticulum degradation-enhancing alpha-mannosidase-like protein 3	610214	EDEM3	80267	ENSG00000116406,ENST00000318130;;;ENST00000318130	Congenital disorder of glycosylation, type IIv	619493	Autosomal recessive	Edem3
1q31	PVOP1	Pelvic organ prolapse, susceptibility to, 1	176780		100312952		{Pelvic organ prolapse, susceptibility to, 1}	176780	Autosomal dominant	
1q31.1	PTGS2	Prostaglandin-endoperoxide synthase 2 (prostaglandin G/H synthase and cyclooxygenase)	600262	PTGS2	5743	ENSG00000073756,ENST00000367468;;;ENST00000367468				
1q31.2	CDC73, HRPT2, C1orf28	Cell division cycle 73	607393	CDC73	79577	ENSG00000134371,ENST000003674	Hyperparathyroidism, familial primary	145000	Autosomal dominant	Cdc73

						35;;;ENST00 000367435				
1q3 1.2	CDC73, HRPT2, C1orf28	Cell division cycle 73	6073 93	CD C73	79 57 7	ENSG00000 134371,ENS T000003674 35;;;ENST00 000367435	Hyperparathyroidism-jaw tumor syndrome	1450 01	Autosomal dominant	Cdc73
1q3 1.2	CDC73, HRPT2, C1orf28	Cell division cycle 73	6073 93	CD C73	79 57 7	ENSG00000 134371,ENS T000003674 35;;;ENST00 000367435	Parathyroid adenoma with cystic changes	1450 01	Autosomal dominant	Cdc73
1q3 1.2	CDC73, HRPT2, C1orf28	Cell division cycle 73	6073 93	CD C73	79 57 7	ENSG00000 134371,ENS T000003674 35;;;ENST00 000367435	Parathyroid carcinoma	6082 66		Cdc73
1q3 1.3	CFH, HF1, HUS, ARMD4, AHUS1	Complement factor H	1343 70	CF H	30 75	ENSG00000 000971,ENS T000003674 29;;;ENST00 000367429	Basal laminar drusen	1267 00	Autosomal dominant	Cfh,Cfhr4
1q3 1.3	CFH, HF1, HUS, ARMD4, AHUS1	Complement factor H	1343 70	CF H	30 75	ENSG00000 000971,ENS T000003674 29;;;ENST00 000367429	Complement factor H deficiency	6098 14	Autosomal dominant; Autosomal recessive	Cfh,Cfhr4
1q3 1.3	CFH, HF1, HUS, ARMD4, AHUS1	Complement factor H	1343 70	CF H	30 75	ENSG00000 000971,ENS T000003674 29;;;ENST00 000367429	{Hemolytic uremic syndrome, atypical, susceptibility to, 1}	2354 00	Autosomal dominant; Autosomal recessive	Cfh,Cfhr4

1q31.3	CFH, HF1, HUS, ARMD4, AHUS1	Complement factor H	134370	CFH	3075	ENSG00000000971,ENST00000367429;;;ENST00000367429	{Macular degeneration, age-related, 4}	610698	Autosomal dominant	Cfh,Cfhr4
1q31.3	ASPM, MCPH5	Abnormal spindle-like, microcephaly-associated	605481	ASPM	259266	ENSG00000066279,ENST00000367409;;;ENST00000367409	Microcephaly 5, primary, autosomal recessive	608716	Autosomal recessive	Aspm
1q31.3-q32.1	PTPRC, CD45, LCA, IMD105	Protein tyrosine phosphatase, receptor type, c polypeptide	151460	PTPRC	5788	ENSG00000081237,ENST00000442510;;;ENST00000442510	Immunodeficiency 105, severe combined	619924	Autosomal recessive	Ptprc
1q32	GFND1	Glomerulopathy with fibronectin deposits 1	137950		100689213		Glomerulopathy with fibronectin deposits 1	137950	Autosomal dominant	
1q32.1	PKP1, EDSFS	Plakophilin-1	601975	PKP1	5317	ENSG00000081277,ENST00000367324;;;ENST00000367324	Ectodermal dysplasia/skin fragility syndrome	604536	Autosomal recessive	Pkp1
1q32.1	TNNT2, CMH2, CMD1D, RCM3, LVNC6	Troponin-T2, cardiac	191045	TNNT2	7139	ENSG00000118194,ENST00000656932;;;ENST00000656932	Cardiomyopathy, dilated, 1D	601494	Autosomal dominant	Tnnt2
1q32.1	TNNT2, CMH2,	Troponin-T2, cardiac	191045	TNNT2	7139	ENSG00000118194,ENST000006569	Cardiomyopathy, familial restrictive, 3	612422	Autosomal dominant	Tnnt2

	CMD1D, RCM3, LVNC6					32;;;ENST00000656932				
1q32.1	TNNT2, CMH2, CMD1D, RCM3, LVNC6	Troponin-T2, cardiac	191045	TNNT2	7139	ENSG00000118194,ENST00000656932;;;ENST00000656932	Cardiomyopathy, hypertrophic, 2	115195	Autosomal dominant	Tnnt2
1q32.1	TNNT2, CMH2, CMD1D, RCM3, LVNC6	Troponin-T2, cardiac	191045	TNNT2	7139	ENSG00000118194,ENST00000656932;;;ENST00000656932	Left ventricular noncompaction 6	601494	Autosomal dominant	Tnnt2
1q32.1	KDM5B, JARID1B, PUT1, PLU1, RBBP2H1A, MRT65	Lysine-specific demethylase 5B	605393	KDM5B	10765	ENSG00000117139,ENST00000367265;;;ENST00000367265	Intellectual developmental disorder, autosomal recessive 65	618109	Autosomal recessive	Kdm5b
1q32.1	ADIPOR1, CGI45	Adiponectin receptor 1	607945	ADIPOR1	51094	ENSG00000159346,ENST00000340990;;;ENST00000340990				
1q32.1	CHI3L1, GP39, YKL40, ASRT7	Chitinase 3-like 1 (cartilage glycoprotein-39)	601525	CHI3L1	1116	ENSG00000133048,ENST00000255409;;;ENST00000255409	{Asthma-related traits, susceptibility to, 7}	611960		Chi3l1
1q32.1	CHI3L1, GP39, YKL40, ASRT7	Chitinase 3-like 1 (cartilage glycoprotein-39)	601525	CHI3L1	1116	ENSG00000133048,ENST00000255409;;;ENST00000255409	{Schizophrenia, susceptibility to}	181500	Autosomal dominant	Chi3l1

1q32.1	REN, RTD, ADTKD4	Renin	179820	REN	5972	ENSG00000143839,ENST00000272190;;;ENST00000272190	Renal tubular dysgenesis	267430	Autosomal recessive	Ren1,Ren2
1q32.1	REN, RTD, ADTKD4	Renin	179820	REN	5972	ENSG00000143839,ENST00000272190;;;ENST00000272190	Tubulointerstitial kidney disease, autosomal dominant, 4	613092	Autosomal dominant	Ren1,Ren2
1q32.1	REN, RTD, ADTKD4	Renin	179820	REN	5972	ENSG00000143839,ENST00000272190;;;ENST00000272190	[Hyperproreninemia]	Ren1,Ren2		
1q32.1	KISS1, HH13	KISS1 metastasis suppressor	603286	KISS1	3814	ENSG00000170498,ENST00000367194;;;ENST00000367194	?Hypogonadotropic hypogonadism 13 with or without anosmia	614842	Autosomal recessive	Kiss1
1q32.1	MDM4, BMFS6	MDM4 regulator of p53	602704	MDM4	4194	ENSG00000198625,ENST00000367182;;;ENST00000367182	?Bone marrow failure syndrome 6	618849	Autosomal dominant	Mdm4
1q32.1	DSTYK, KIAA0472, RIP5, DUSTYPK, CAKUT1, SPG23	Dual serine/threonine and tyrosine protein kinase	612666	DSTYK	25778	ENSG00000133059,ENST00000367162;;;ENST00000367162	Congenital anomalies of kidney and urinary tract 1	610805	Autosomal dominant	Dstyk

1q32.1	DSTYK, KIAA0472, RIP5, DUSTYPK, CAKUT1, SPG23	Dual serine/threonine and tyrosine protein kinase	612666	DSTYK	25778	ENSG00000133059,ENST00000367162;;;ENST00000367162	Spastic paraplegia 23	270750	Autosomal recessive	Dstyk
1q32.1	PM20D1	Peptidase M20 domain-containing protein 1	617124	PM20D1	148811	ENSG00000162877,ENST00000367136;;;ENST00000367136				
1q32.1	IKBKE	Inhibitor of nuclear factor kappa-B kinase, subunit epsilon	605048	IKBKE	9641	ENSG00000263528,ENST00000581977;;;ENST00000581977				
1q32.2	CD55, DAF, CROM, CHAPLE	CD55 antigen (blood group Cromer)	125240	CD55	1604	ENSG00000196352,ENST00000367064;;;ENST00000367064	Complement hyperactivation, angiopathic thrombosis, and protein-losing enteropathy	226300	Autosomal recessive	Cd55,Cd55b
1q32.2	CD55, DAF, CROM, CHAPLE	CD55 antigen (blood group Cromer)	125240	CD55	1604	ENSG00000196352,ENST00000367064;;;ENST00000367064	[Blood group Cromer]	613793	Autosomal recessive	Cd55,Cd55b
1q32.2	MCP, CD46, AHUS2	Membrane cofactor protein (CD46, trophoblast-lymphocyte cross-reactive antigen)	120920	CD46	4179	ENSG00000117335,ENST00000367042;;;ENST00000367042	{Hemolytic uremic syndrome, atypical, susceptibility to, 2}	612922	Autosomal dominant; Autosomal recessive	Cd46

1q32.2	LAMB3, AI1A, JEB1B, JEB1A	Laminin, beta-3 (nicein, 125kD; kalinin, 140kD; BM600, 125kD)	150310	LAMB3	3914	ENSG00000196878,ENST00000356082;;;ENST00000356082	Amelogenesis imperfecta, type IA	104530	Autosomal dominant	Lamb3
1q32.2	LAMB3, AI1A, JEB1B, JEB1A	Laminin, beta-3 (nicein, 125kD; kalinin, 140kD; BM600, 125kD)	150310	LAMB3	3914	ENSG00000196878,ENST00000356082;;;ENST00000356082	Epidermolysis bullosa, junctional 1A, intermediate	226650	Autosomal recessive	Lamb3
1q32.2	LAMB3, AI1A, JEB1B, JEB1A	Laminin, beta-3 (nicein, 125kD; kalinin, 140kD; BM600, 125kD)	150310	LAMB3	3914	ENSG00000196878,ENST00000356082;;;ENST00000356082	Epidermolysis bullosa, junctional 1B, severe	226700	Autosomal recessive	Lamb3
1q32.2	HSD11B1, HSD11, HSD11L, CORTRD2	Hydroxysteroid, 11-beta, dehydrogenase 1	600713	HSD11B1	3290	ENSG00000117594,ENST00000367027;;;ENST00000367027	Cortisone reductase deficiency 2	614662	Autosomal dominant	Hsd11b1
1q32.3	PROX1	Prospero homeobox 1	601546	PROX1	5629	ENSG00000117707,ENST00000366958;;;ENST00000366958				
1q41	MIA3, TANGO1, TANGO, KIAA0268, ODCD2	MIA SH3 domain ER export factor 3	613455	MIA3	375056	ENSG00000154305,ENST00000344922;;;ENST00000344922	?Ondontochondrodysplasia 2 with hearing loss and diabetes	619269	Autosomal recessive	Mia3
1q41	TLR5, TIL3, SLEB1, MELIOS	Toll-like receptor-5	603031	TLR5	7100	ENSG00000187554,ENST000006426	{Legionnaire disease, susceptibility to}	608556		Tlr5

						03;;;ENST00 000642603				
1q4 1	TLR5, TIL3, SLEB1, MELIOS	Toll-like receptor-5	6030 31	TL R5	71 00	ENSG00000 187554,ENS T000006426 03;;;ENST00 000642603	{Melioidosis, susceptibility to}	6155 57	Autosomal dominant	Tlr5
1q4 1	TLR5, TIL3, SLEB1, MELIOS	Toll-like receptor-5	6030 31	TL R5	71 00	ENSG00000 187554,ENS T000006426 03;;;ENST00 000642603	{Systemic lupus erythematosus, resistance to}	6017 44		Tlr5
1q4 1	TLR5, TIL3, SLEB1, MELIOS	Toll-like receptor-5	6030 31	TL R5	71 00	ENSG00000 187554,ENS T000006426 03;;;ENST00 000642603	{Systemic lupus erythematosus, susceptibility to, 1}	6017 44		Tlr5
1q4 2.11	DEGS1, DES1, MLD, HLD18	Delta(4)-desaturase, sphingolipid, 1	6158 43	DE GS 1	85 60	ENSG00000 143753,ENS T000003236 99;;;ENST00 000323699	Leukodystrophy, hypomyelinating, 18	6184 04	Autosomal recessive	Degs1,Degs1l
1q4 2.13	PSEN2, AD4, STM2, CMD1V	Presenilin 2	6007 59	PS EN 2	56 64	ENSG00000 143801,ENS T000003667 83;;;ENST00 000366783	Alzheimer disease-4	6068 89	Autosomal dominant	Psen2
1q4 2.13	PSEN2, AD4, STM2, CMD1V	Presenilin 2	6007 59	PS EN 2	56 64	ENSG00000 143801,ENS T000003667 83;;;ENST00 000366783	Cardiomyopathy, dilated, 1V	6136 97	Autosomal dominant	Psen2

1q42.13	ADCK3, COQ8, CABC1, SCAR9, ARCA2, COQ10D4	AARF domain-containing kinase 3	606980	COQ8A	56997	ENSG00000163050,ENST00000366777;;;ENST00000366777	Coenzyme Q10 deficiency, primary, 4	612016	Autosomal recessive	Coq8a
1q42.13	GJC2, GJA12, CX47, PMLDAR, HLD2, SPG44, LMPHM3	Gap junction protein, gamma-2 (47kD)	608803	GJC2	57165	ENSG00000198835,ENST00000366714;;;ENST00000366714	?Spastic paraplegia 44, autosomal recessive	613206	Autosomal recessive	Gjc2
1q42.13	GJC2, GJA12, CX47, PMLDAR, HLD2, SPG44, LMPHM3	Gap junction protein, gamma-2 (47kD)	608803	GJC2	57165	ENSG00000198835,ENST00000366714;;;ENST00000366714	Leukodystrophy, hypomyelinating, 2	608804	Autosomal recessive	Gjc2
1q42.13	GJC2, GJA12, CX47, PMLDAR, HLD2, SPG44, LMPHM3	Gap junction protein, gamma-2 (47kD)	608803	GJC2	57165	ENSG00000198835,ENST00000366714;;;ENST00000366714	Lymphatic malformation 3	613480	Autosomal dominant	Gjc2
1q42.13	ACTA1, ASMA, NEM3, CMYP2A, CMYP2B, SHPM, CMYP2C	Actin, alpha-1, skeletal muscle	102610	ACTA1	58	ENSG00000143632,ENST00000366684;;;ENST00000366684	?Myopathy, scapulohumeroperoneal	616852	Autosomal dominant	Acta1
1q42.13	ACTA1, ASMA, NEM3, CMYP2A, CMYP2B, SHPM, CMYP2C	Actin, alpha-1, skeletal muscle	102610	ACTA1	58	ENSG00000143632,ENST00000366684;;;ENST00000366684	Congenital myopathy 2A, typical, autosomal dominant	161800	Autosomal dominant	Acta1

1q42.13	ACTA1, ASMA, NEM3, CMYP2A, CMYP2B, SHPM, CMYP2C	Actin, alpha-1, skeletal muscle	102610	ACTA1	58	ENSG00000143632,ENST00000366684;;;ENST00000366684	Congenital myopathy 2B, severe infantile, autosomal recessive	620265	Autosomal recessive	Acta1
1q42.13	ACTA1, ASMA, NEM3, CMYP2A, CMYP2B, SHPM, CMYP2C	Actin, alpha-1, skeletal muscle	102610	ACTA1	58	ENSG00000143632,ENST00000366684;;;ENST00000366684	Congenital myopathy 2C, severe infantile, autosomal dominant	620278	Autosomal dominant	Acta1
1q42.2	AGT, SERPINA8	Angiotensinogen	106150	AGT	183	ENSG00000135744,ENST00000366667;;;ENST00000366667	Renal tubular dysgenesis	267430	Autosomal recessive	Agt
1q42.2	AGT, SERPINA8	Angiotensinogen	106150	AGT	183	ENSG00000135744,ENST00000366667;;;ENST00000366667	{Hypertension, essential, susceptibility to}	145500	Multifactorial	Agt
1q42.2	AGT, SERPINA8	Angiotensinogen	106150	AGT	183	ENSG00000135744,ENST00000366667;;;ENST00000366667	{Preeclampsia, susceptibility to}	Agt		
1q42.2	SPRTN, DVC1, C1orf124, RJALS	SprT-like N-terminal domain protein	616086	SPRTN	83932	ENSG00000010072,ENST00000295050;;;ENST00000295050	Ruijs-Aalfs syndrome	616200	Autosomal recessive	Sprtn

1q4 2.2	DISC2	Disrupted in schizophrenia 2	6062 71	DIS C2	27 18 4		Schizophrenia	1815 00	Autosomal dominant	
1q4 2.3	ARID4B, SAP180, RBP1L1, RBBP1L1, BRCAA1	AT-rich interactive domain-containing protein 4B	6096 96	AR ID4 B	51 74 2	ENSG00000 054267,ENS T000002641 83;;;ENST00 000264183				
1q4 2.3	GGPS1, GGPPS1, MDHLO	Geranylgeranyl diphosphate synthase 1	6069 82	GG PS1	94 53	ENSG00000 152904,ENS T000002828 41;;;ENST00 000282841	Muscular dystrophy, congenital hearing loss, and ovarian insufficiency syndrome	6195 18	Autosomal recessive	Ggps1
1q4 2.3	LYST, CHS1	Lysosomal trafficking regulator	6068 97	LY ST	11 30	ENSG00000 143669,ENS T000003897 93;;;ENST00 000389793	Chediak-Higashi syndrome	2145 00	Autosomal recessive	Lyst
1q4 2.3-q43	EDARADD, ED3, EDA3, ECTD11B, ECTD11A	EDAR-associated death domain	6066 03	ED AR AD D	12 81 78	ENSG00000 186197,ENS T000003342 32;;;ENST00 000334232	Ectodermal dysplasia 11A, hypohidrotic/hair/tooth type, autosomal dominant	6149 40	Autosomal dominant	Edaradd
1q4 2.3-q43	EDARADD, ED3, EDA3, ECTD11B, ECTD11A	EDAR-associated death domain	6066 03	ED AR AD D	12 81 78	ENSG00000 186197,ENS T000003342 32;;;ENST00 000334232	Ectodermal dysplasia 11B, hypohidrotic/hair/tooth type, autosomal recessive	6149 41	Autosomal recessive	Edaradd
1q4 3	MTR, HMAG	5-methyltetrahydrofolate-homocysteine methyltransferase 1	1565 70	MT R	45 48	ENSG00000 116984,ENS T000003665 77;;;ENST00 000366577	Homocystinuria-megaloblastic anemia, cblG complementation type	2509 40	Autosomal recessive	Mtr

1q43	MTR, HMAG	5-methyltetrahydrofolate-homocysteine methyltransferase 1	156570	MTR	4548	ENSG00000116984,ENST00000366577;;;ENST00000366577	{Neural tube defects, folate-sensitive, susceptibility to}	601634	Autosomal recessive	Mtr
1q43	RYR2, VTSIP, ARVC2, VACRDS	Ryanodine receptor-2 (cardiac)	180902	RYR2	6262	ENSG00000198626,ENST00000366574;;;ENST00000366574	Ventricular arrhythmias due to cardiac ryanodine receptor calcium release deficiency syndrome	115000	Autosomal dominant	Ryr2
1q43	RYR2, VTSIP, ARVC2, VACRDS	Ryanodine receptor-2 (cardiac)	180902	RYR2	6262	ENSG00000198626,ENST00000366574;;;ENST00000366574	Ventricular tachycardia, catecholaminergic polymorphic, 1	604772	Autosomal dominant	Ryr2
1q43	CHRM3, PBS, EGBRS	Cholinergic receptor, muscarinic, 3	118494	CHRM3	1131	ENSG00000133019,ENST00000676153;;;ENST00000676153	Prune belly syndrome	100100	Autosomal recessive	Chrm3
1q43	FMN2, MRT47	Formin 2	606373	FMN2	56776	ENSG00000155816,ENST00000319653;;;ENST00000319653	Intellectual developmental disorder, autosomal recessive 47	616193	Autosomal recessive	Fmn2
1q43	BECN2, BECN1L1	Beclin 2	615687	BECN2	441925	ENSG00000196289,ENST00000419583;;;ENST00000419583				
1q43-q44	SDCCAG8, CCCAP, SLSN7, BBS16	SHH signaling and ciliogenesis regulator SDCCAG8	613524	SDCC	10806	ENSG00000054282,ENST000003665	Bardet-Biedl syndrome 16	615993	Autosomal recessive	Sdccag8

				AG 8		41;;;ENST00 000366541				
1q4 3-q44	SDCCAG8, CCCAP, SLSN7, BBS16	SHH signaling and ciliogenesis regulator SDCCAG8	6135 24	SD CC AG 8	10 80 6	ENSG00000 054282,ENS T000003665 41;;;ENST00 000366541	Senior-Loken syndrome 7	6136 15	Autosomal recessive	Sdccag8
1q4 4	NLRP3, CIAS1, FCU, FCAS1, NALP3, PYPAF1, DFNA34, KEFH	NLR family, pyrin domain containing 3	6064 16	NL RP 3	11 45 48	ENSG00000 162711,ENS T000003361 19;;;ENST00 000336119	CINCA syndrome	6071 15	Autosomal dominant	Nlrp3
1q4 4	NLRP3, CIAS1, FCU, FCAS1, NALP3, PYPAF1, DFNA34, KEFH	NLR family, pyrin domain containing 3	6064 16	NL RP 3	11 45 48	ENSG00000 162711,ENS T000003361 19;;;ENST00 000336119	Deafness, autosomal dominant 34, with or without inflammation	6177 72	Autosomal dominant	Nlrp3
1q4 4	NLRP3, CIAS1, FCU, FCAS1, NALP3, PYPAF1, DFNA34, KEFH	NLR family, pyrin domain containing 3	6064 16	NL RP 3	11 45 48	ENSG00000 162711,ENS T000003361 19;;;ENST00 000336119	Familial cold inflammatory syndrome 1	1201 00	Autosomal dominant	Nlrp3
1q4 4	NLRP3, CIAS1, FCU, FCAS1, NALP3, PYPAF1, DFNA34, KEFH	NLR family, pyrin domain containing 3	6064 16	NL RP 3	11 45 48	ENSG00000 162711,ENS T000003361 19;;;ENST00 000336119	Keratoendothelitis fugax hereditaria	1482 00	Autosomal dominant	Nlrp3

1q44	NLRP3, CIAS1, FCU, FCAS1, NALP3, PYPAF1, DFNA34, KEFH	NLR family, pyrin domain containing 3	606416	NLRP3	114548	ENSG00000162711,ENST00000336119;;;ENST00000336119	Muckle-Wells syndrome	191900	Autosomal dominant	Nlrp3
2p25-p24	HYT3	Hypertension, essential, susceptibility to, 3	607329		387575		{Hypertension, essential, susceptibility to, 3}	145500	Multifactorial	
2p25	PEE2	Preeclampsia/eclampsia 2	609402		780908		Preeclampsia/eclampsia 2	609402		
2p25.3	TMEM18	Transmembrane protein 18	613220	TMEM18	129787	ENSG00000151353,ENST00000281017;;;ENST00000281017				
2p25.3	TPO, TPX, TDH2A	Thyroid peroxidase	606765	TPO	7173	ENSG00000115705,ENST00000329066;;;ENST00000329066	Thyroid dyshormonogenesis 2A	274500	Autosomal recessive	Tpo
2p25.3	MYT1L, KIAA1106, MRD39	Myelin transcription factor 1-like	613084	MYT1L	23040	ENSG00000186487,ENST00000647738;;;ENST00000647738	Intellectual developmental disorder, autosomal dominant 39	616521	Autosomal dominant	Myt1l
2p25.2	SOX11, MRD27, IDDMOH	SRY-box 11	600898	SOX11	6664	ENSG00000176887,ENST00000322002;;;ENST00000322002	Intellectual developmental disorder with microcephaly and with or without ocular malformations or hypogonadotropic hypogonadism	615866	Autosomal dominant	Sox11

2p25.1	KIDINS220, ARMS, SINO, VENARG	Kinase D-interacting substrate, 220kD	615759	KIDINS220	57498	ENSG00000134313,ENST00000256707;;;ENST00000256707	Spastic paraplegia, intellectual disability, nystagmus, and obesity	617296	Autosomal dominant	Kidins220
2p25.1	KIDINS220, ARMS, SINO, VENARG	Kinase D-interacting substrate, 220kD	615759	KIDINS220	57498	ENSG00000134313,ENST00000256707;;;ENST00000256707	Ventriculomegaly and arthrogryposis	619501	Autosomal recessive	Kidins220
2p25.1	LPIN1	Lipin 1	605518	LPIN1	23175	ENSG00000134324,ENST00000674199;;;ENST00000674199	Myoglobinuria, acute recurrent, autosomal recessive	268200	Autosomal recessive	Lpin1
2p24.3	MYCN, NMYC, ODED, MODED	Oncogene NMYC	164840	MYCN	4613	ENSG00000134323,ENST00000281043;;;ENST00000281043	Feingold syndrome 1	164280	Autosomal dominant	Mycn,Mycs
2p24.1	SDC1	Syndecan 1	186355	SDC1	6382	ENSG00000115884,ENST00000254351;;;ENST00000254351				
2p24.1	APOB, FLDB, LDLCQ4, FCHL2	Apolipoprotein B (including Ag(x) antigen)	107730	APOB	338	ENSG00000084674,ENST00000233242;;;ENST00000233242	Hypercholesterolemia, familial, 2	144010	Autosomal dominant	Apob
2p24.1	APOB, FLDB, LDLCQ4, FCHL2	Apolipoprotein B (including Ag(x) antigen)	107730	APOB	338	ENSG00000084674,ENST000002332	Hypobetalipoproteinemia	615558	Autosomal recessive	Apob

						42;;;ENST00000233242				
2p23.3	NCOA1, SRC1	Nuclear receptor coactivator 1	602691	NCOA1	8648	ENSG00000084676,ENST00000348332;;;ENST00000348332				
2p23.3	ADCY3, BMIQ19	Adenylate cyclase-3	600291	ADCY3	109	ENSG00000138031,ENST00000679454;;;ENST00000679454	{Obesity, susceptibility to, BMIQ19}	617885	Autosomal recessive	Adcy3
2p23.3	POMC, OBAIRH	Proopiomelanocortin (adrenocorticotropin/beta-lipotropin)	176830	POMC	5443	ENSG00000115138,ENST00000395826;;;ENST00000395826	Obesity, adrenal insufficiency, and red hair due to POMC deficiency	609734	Autosomal recessive	Pomc
2p23.3	POMC, OBAIRH	Proopiomelanocortin (adrenocorticotropin/beta-lipotropin)	176830	POMC	5443	ENSG00000115138,ENST00000395826;;;ENST00000395826	{Obesity, early-onset, susceptibility to}	601665	Autosomal dominant; Autosomal recessive; Multifactorial	Pomc
2p23.3	DNMT3A, TBRS, HESJAS	DNA methyltransferase 3A	602769	DNMT3A	1788	ENSG00000119772,ENST00000321117;;;ENST00000321117	Acute myeloid leukemia, somatic	601626		Dnmt3a
2p23.3	DNMT3A, TBRS, HESJAS	DNA methyltransferase 3A	602769	DNMT3A	1788	ENSG00000119772,ENST00000321117;;;ENST00000321117	Heyn-Sproul-Jackson syndrome	618724	Autosomal dominant	Dnmt3a

2p2 3.3	DNMT3A, TBRS, HESJAS	DNA methyltransferase 3A	6027 69	DN MT 3A	17 88	ENSG00000 119772,ENS T000003211 17;;;ENST00 000321117	Tatton-Brown-Rahman syndrome	6158 79	Autosomal dominant	Dnmt3a

Supplementary table S6. Targets of genes associated with maternal obesity from DisGeNet (http://www.disgenet.org/)

Disease	Disease_id	Gene	Gene_id	UniProt	Gene_Full_Name	Protein_Class	N_diseases_g	DSI_g	DPI_g	pLI	Score_gda
early pregnancy	C0747845	VEGFA	7422	P15692	vascular endothelial growth factor A	Signaling	1899	0.266	0.923	2.41E-05	0.09
early pregnancy	C0747845	MTHFR	4524	P42898	methylenetetrahydrofolate reductase		985	0.337	0.885	3.21E-10	0.08
early pregnancy	C0747845	PGF	5228	P49763	placental growth factor	Signaling	288	0.46	0.846	0.1288	0.05
early pregnancy	C0747845	IL10	3586	P22301	interleukin 10		1679	0.281	0.923	0.005887	0.04
early pregnancy	C0747845	PROK1	84432	P58294	prokineticin 1	Signaling	57	0.636	0.462	6.17E-06	0.04
early pregnancy	C0747845	FGF2	2247	P09038	fibroblast growth factor 2	Signaling	635	0.383	0.923	0.0184	0.03
early pregnancy	C0747845	IFNG	3458	P01579	interferon gamma		1519	0.288	0.962	0.47156	0.03
early pregnancy	C0747845	IGF1	3479	P05019	insulin like growth factor 1		1206	0.318	0.885	0.2716	0.03
early pregnancy	C0747845	IL6	3569	P05231	interleukin 6		2367	0.248	0.962	0.31536	0.03

early pregnanc y	C0747 845	LEP	3952	P41159	leptin		931	0.3 49	0.8 46	0.46 491	0.03
early pregnanc y	C0747 845	LGALS 9	3965	O00182	galectin 9	Signaling	197	0.4 96	0.7 69	6.55 E-06	0.03
early pregnanc y	C0747 845	PGR	5241	P06401	progesterone receptor	Nuclear receptor	392	0.4 26	0.8 85	0.05 1042	0.03
early pregnanc y	C0747 845	BCL2	596	P10415	BCL2 apoptosis regulator	Signaling	1456	0.2 91	0.8 85	0.55 903	0.03
early pregnanc y	C0747 845	BDNF	627	P23560	brain derived neurotrophic factor	Signaling	992	0.3 45	0.9 23	0.65 626	0.03
early pregnanc y	C0747 845	SHBG	6462	P04278	sex hormone binding globulin		368	0.4 38	0.8 08	1.25 E-06	0.03
early pregnanc y	C0747 845	TNF	7124	P01375	tumor necrosis factor	Signaling	2724	0.2 31	0.9 62	0.80 33	0.03
early pregnanc y	C0747 845	TP53	7157	P04637	tumor protein p53	Transcription factor	2494	0.2 36	0.9 62	0.53 235	0.03
early pregnanc y	C0747 845	CXCR4	7852	P61073	C-X-C motif chemokine receptor 4	G-protein coupled receptor	739	0.3 62	0.9 23	0.01 7804	0.03
early pregnanc y	C0747 845	HAVCR 2	8486 8	Q8TDQ0	hepatitis A virus cellular receptor 2		299	0.4 5	0.8 08	0.03 866	0.03
early pregnanc y	C0747 845	ADIPO Q	9370	Q15848	adiponectin, C1Q and collagen domain containing		679	0.3 76	0.8 85	4.64 E-09	0.03

early pregnanc y	C0747 845	FST	1046 8	P19883	follistatin	Enzyme modulator	182	0.5 13	0.7 31	0.98 014	0.02
early pregnanc y	C0747 845	PROKR 1	1088 7	Q8TCW9	prokineticin receptor 1	G-protein coupled receptor	27	0.8 61	0.1 92	1.16 E-07	0.02
early pregnanc y	C0747 845	PROKR 2	1286 74	Q8NFJ6	prokineticin receptor 2	G-protein coupled receptor	164	0.5 38	0.7 31	2.44 E-06	0.02
early pregnanc y	C0747 845	CRP	1401	P02741	C-reactive protein		1483	0.2 99	0.9 62	0.00 3697	0.02
early pregnanc y	C0747 845	CYP11 A1	1583	P05108	cytochrome P450 family 11 subfamily A member 1		166	0.5 47	0.7 31	5.49 E-07	0.02
early pregnanc y	C0747 845	CYP26 A1	1592	O43174	cytochrome P450 family 26 subfamily A member 1	Enzyme	59	0.6 19	0.5 77	8.43 E-11	0.02
early pregnanc y	C0747 845	DSG2	1829	Q14126	desmoglein 2	Cell adhesion	83	0.6 01	0.6 92	2.79 E-10	0.02
early pregnanc y	C0747 845	EGFR	1956	P00533	epidermal growth factor receptor	Kinase	1394	0.2 95	0.8 85	0.36 837	0.02
early pregnanc y	C0747 845	ESR1	2099	P03372	estrogen receptor 1	Nuclear receptor	1101	0.3 24	0.9 62	0.99 92	0.02
early pregnanc y	C0747 845	FGFR2	2263	P21802	fibroblast growth factor receptor 2	Kinase	731	0.3 8	0.8 08	0.99 731	0.02
early pregnanc y	C0747 845	AMH	268	P03971	anti-Mullerian hormone		242	0.4 76	0.8 46	3.4E- 12	0.02

early pregnancy	C0747845	GJA1	2697	P17302	gap junction protein alpha 1	Cell-cell junction	662	0.393	0.885	0.15523	0.02
early pregnancy	C0747845	CD274	29126	Q9NZQ7	CD274 molecule	Receptor	1011	0.324	0.923	0.01916	0.02
early pregnancy	C0747845	HSD11B2	3291	P80365	hydroxysteroid 11-beta dehydrogenase 2	Enzyme	163	0.532	0.808	0.11777	0.02
early pregnancy	C0747845	IFNA1	3439	P01562	interferon alpha 1		662	0.371	0.923		0.02
early pregnancy	C0747845	IFNA13	3447	P01562	interferon alpha 13		646	0.374	0.923		0.02
early pregnancy	C0747845	IL1B	3553	P01584	interleukin 1 beta		1801	0.276	0.962	0.13005	0.02
early pregnancy	C0747845	IL2	3558	P60568	interleukin 2		950	0.336	0.885	0.47968	0.02
early pregnancy	C0747845	IL4	3565	P05112	interleukin 4		996	0.332	0.962	0.004737	0.02
early pregnancy	C0747845	LIF	3976	P15018	LIF interleukin 6 family cytokine	Signaling	236	0.483	0.769	0.5242	0.02
early pregnancy	C0747845	MMP9	4318	P14780	matrix metallopeptidase 9	Enzyme	1337	0.305	0.923	1.89E-17	0.02
early pregnancy	C0747845	NOS3	4846	P29474	nitric oxide synthase 3		706	0.378	0.885	6.72E-07	0.02

early pregnancy	C0747845	FOXP3	50943	Q9BZS1	forkhead box P3		688	0.368	0.846	0.99424	0.02
early pregnancy	C0747845	PPARG	5468	P37231	peroxisome proliferator activated receptor gamma	Nuclear receptor	877	0.358	0.885	0.029194	0.02
early pregnancy	C0747845	CHDH	55349	Q8NE62	choline dehydrogenase	Enzyme	158	0.529	0.769	1.69E-06	0.02
early pregnancy	C0747845	PRKAR1A	5573	P10644	protein kinase cAMP-dependent type I regulatory subunit alpha		597	0.401	0.846	0.99985	0.02
early pregnancy	C0747845	PRKAR2A	5576	P13861	protein kinase cAMP-dependent type II regulatory subunit alpha		16	0.805	0.269	0.11865	0.02
early pregnancy	C0747845	PRL	5617	P01236	prolactin	Signaling	506	0.406	0.885	2.9E-07	0.02
early pregnancy	C0747845	RARRES2	5919	Q99969	retinoic acid receptor responder 2		180	0.519	0.731	2.72E-05	0.02
early pregnancy	C0747845	RHD	6007	Q02161	Rh blood group D antigen		67	0.612	0.654	2.85E-05	0.02
early pregnancy	C0747845	BRD2	6046	P25440	bromodomain containing 2	Epigenetic regulator	303	0.45	0.808	0.99957	0.02
early pregnancy	C0747845	CCL2	6347	P13500	C-C motif chemokine ligand 2	Signaling	1157	0.321	0.962	0.60786	0.02
early pregnancy	C0747845	CXCL12	6387	P48061	C-X-C motif chemokine ligand 12		626	0.379	0.846	0.11851	0.02

early pregnanc y	C0747 845	BMP2	650	P12643	bone morphogenetic protein 2	Signaling	428	0.4 32	0.8 46	0.98 799	0.02
early pregnanc y	C0747 845	TLR4	7099	O00206	toll like receptor 4		1174	0.3 21	0.9 62	4.61 E-09	0.02
early pregnanc y	C0747 845	VDR	7421	P11473	vitamin D receptor	Nuclear receptor	852	0.3 52	0.8 85	1.68 E-05	0.02
early pregnanc y	C0747 845	ARID1 A	8289	O14497	AT-rich interaction domain 1A		341	0.4 55	0.8 46	1	0.02
early pregnanc y	C0747 845	IL33	9086 5	O95760	interleukin 33		487	0.4 09	0.8 85	8.29 E-10	0.02
early pregnanc y	C0747 845	CD28	940	P10747	CD28 molecule		364	0.4 36	0.8 85	0.35 779	0.02
early pregnanc y	C0747 845	HOTAI R	1E+0 8		HOX transcript antisense RNA		234	0.4 75	0.7 69		0.01
early pregnanc y	C0747 845	LINC01 672	1.01 E+08		long intergenic non-protein coding RNA 1672		268	0.4 62	0.8 46		0.01
early pregnanc y	C0747 845	CERT1	1008 7	Q9Y5P4	ceramide transporter 1	Transporter	71	0.6 74	0.5	0.66 702	0.01
early pregnanc y	C0747 845	NAMP T	1013 5	P43490	nicotinamide phosphoribosyltransferase	Signaling	199	0.5 02	0.8 08	0.97 914	0.01
early pregnanc y	C0747 845	ADGRG 2	1014 9	Q8IZP9	adhesion G protein-coupled receptor G2	G-protein coupled receptor	24	0.7 36	0.2 31	0.92 694	0.01

early pregnancy	C0747845	CALCRL	10203	Q16602	calcitonin receptor like receptor	G-protein coupled receptor	47	0.653	0.692	0.9853	0.01
early pregnancy	C0747845	RAMP1	10267	O60894	receptor activity modifying protein 1	Receptor	37	0.663	0.5	0.13181	0.01
early pregnancy	C0747845	DSCR4	10281	P56555	Down syndrome critical region 4		7	0.89	0.115	0.000778	0.01
early pregnancy	C0747845	CRISP3	10321	P54108	cysteine rich secretory protein 3	Immune response	31	0.716	0.577	2.57E-06	0.01
early pregnancy	C0747845	NDRG1	10397	Q92597	N-myc downstream regulated 1	Enzyme	225	0.488	0.731	0.011324	0.01
early pregnancy	C0747845	NCOA2	10499	Q15596	nuclear receptor coactivator 2	Enzyme	95	0.575	0.615	1	0.01
early pregnancy	C0747845	TXNIP	10628	Q9H3M7	thioredoxin interacting protein		208	0.49	0.731	0.15918	0.01
early pregnancy	C0747845	EBP	10682	Q15125	EBP cholestenol delta-isomerase	Enzyme	243	0.494	0.846	0.93645	0.01
early pregnancy	C0747845	CORIN	10699	Q9Y5Q5	corin, serine peptidase		55	0.695	0.538	6.51E-35	0.01
early pregnancy	C0747845	KDM5B	10765	Q9UGL1	lysine demethylase 5B	Epigenetic regulator	162	0.532	0.731	7.91E-15	0.01
early pregnancy	C0747845	CGA	1081	P01215	glycoprotein hormones, alpha polypeptide		180	0.508	0.808	0.34518	0.01

early pregnancy	C0747845	CGB3	1082	P0DN86	chorionic gonadotropin subunit beta 3	Signaling	93	0.57	0.654	0.098257	0.01
early pregnancy	C0747845	COPS5	10987	Q92905	COP9 signalosome subunit 5	Enzyme	101	0.566	0.769	0.99925	0.01
early pregnancy	C0747845	IL24	11009	Q13007	interleukin 24		202	0.498	0.769	5.88E-07	0.01
early pregnancy	C0747845	PDCD10	11235	Q9BUL8	programmed cell death 10		68	0.631	0.692	0.96197	0.01
early pregnancy	C0747845	KLF8	11279	O95600	Kruppel like factor 8	Nucleic acid binding	55	0.628	0.462	0.002247	0.01
early pregnancy	C0747845	PIK3IP1	113791	Q96FE7	phosphoinositide-3-kinase interacting protein 1	Enzyme	19	0.769	0.385	0.015067	0.01
early pregnancy	C0747845	CCR5	1234	P51681	C-C motif chemokine receptor 5 (gene/pseudogene)	G-protein coupled receptor	508	0.398	0.885	4.31E-10	0.01
early pregnancy	C0747845	CCR6	1235	P51684	C-C motif chemokine receptor 6	G-protein coupled receptor	231	0.485	0.808	0.005787	0.01
early pregnancy	C0747845	CCR7	1236	P32248	C-C motif chemokine receptor 7	G-protein coupled receptor	320	0.443	0.769	0.057623	0.01
early pregnancy	C0747845	CMKLR1	1240	Q99788	chemerin chemokine-like receptor 1	G-protein coupled receptor	75	0.612	0.692	0.010767	0.01
early pregnancy	C0747845	CRH	1392	P06850	corticotropin releasing hormone	Signaling	402	0.439	0.808	0.71614	0.01

early pregnancy	C0747845	CSF3	1440	P09919	colony stimulating factor 3		687	0.377	0.885	0.38046	0.01
early pregnancy	C0747845	CTNNB1	1499	P35222	catenin beta 1		1368	0.303	0.885	0.99999	0.01
early pregnancy	C0747845	CTSE	1510	P14091	cathepsin E	Enzyme	56	0.631	0.615	1.44E-14	0.01
early pregnancy	C0747845	CREBRF	153222	Q8IUR6	CREB3 regulatory factor		50	0.636	0.5	0.99997	0.01
early pregnancy	C0747845	CYP1A1	1543	P04798	cytochrome P450 family 1 subfamily A member 1	Enzyme	379	0.436	0.846	1.06E-17	0.01
early pregnancy	C0747845	CYP19A1	1588	P11511	cytochrome P450 family 19 subfamily A member 1	Enzyme	519	0.41	0.885	1.7E-05	0.01
early pregnancy	C0747845	DCN	1634	P07585	decorin		297	0.457	0.808	0.31231	0.01
early pregnancy	C0747845	GADD45A	1647	P24522	growth arrest and DNA damage inducible alpha		151	0.526	0.769	0.044075	0.01
early pregnancy	C0747845	DHFR	1719	P00374	dihydrofolate reductase	Enzyme	191	0.513	0.808	0.0343	0.01
early pregnancy	C0747845	AFP	174	P02771	alpha fetoprotein	Transporter	392	0.429	0.885	1.14E-11	0.01
early pregnancy	C0747845	DOCK1	1793	Q14185	dedicator of cytokinesis 1		58	0.641	0.577	0.006812	0.01

early pregnancy	C0747845	AGTR1	185	P30556	angiotensin II receptor type 1	G-protein coupled receptor	440	0.423	0.846	0.000532	0.01
early pregnancy	C0747845	AHR	196	P35869	aryl hydrocarbon receptor	Transcription factor	532	0.41	0.923	0.99999	0.01
early pregnancy	C0747845	STX2	2054	P32856	syntaxin 2	Transporter	71	0.626	0.577	2.11E-07	0.01
early pregnancy	C0747845	ERBB4	2066	Q15303	erb-b2 receptor tyrosine kinase 4	Kinase	317	0.455	0.846	0.99912	0.01
early pregnancy	C0747845	ERN1	2081	O75460	endoplasmic reticulum to nucleus signaling 1	Kinase	163	0.513	0.808	0.99027	0.01
early pregnancy	C0747845	ESR2	2100	Q92731	estrogen receptor 2	Nuclear receptor	528	0.4	0.923	4.45E-08	0.01
early pregnancy	C0747845	ETV4	2118	P43268	ETS variant transcription factor 4	Transcription factor	120	0.545	0.5	2.87E-08	0.01
early pregnancy	C0747845	F5	2153	P12259	coagulation factor V	Enzyme	369	0.441	0.846	5.92E-15	0.01
early pregnancy	C0747845	FAAH	2166	O00519	fatty acid amide hydrolase		177	0.545	0.692	4.44E-11	0.01
early pregnancy	C0747845	FABP4	2167	P15090	fatty acid binding protein 4		194	0.5	0.808	5.18E-05	0.01
early pregnancy	C0747845	FCGR3A	2214	P08637	Fc fragment of IgG receptor IIIa	Cell adhesion	352	0.432	0.962	3.7E-06	0.01

early pregnancy	C0747845	FCGR3B	2215	O75015	Fc fragment of IgG receptor IIIb	Cell adhesion	291	0.452	0.962	3.03E-07	0.01
early pregnancy	C0747845	FGF7	2252	P21781	fibroblast growth factor 7	Signaling	167	0.519	0.846	0.77089	0.01
early pregnancy	C0747845	FGF9	2254	P31371	fibroblast growth factor 9	Signaling	153	0.528	0.846	0.94704	0.01
early pregnancy	C0747845	FGFR1	2260	P11362	fibroblast growth factor receptor 1	Kinase	816	0.362	0.885	0.99984	0.01
early pregnancy	C0747845	FGR	2268	P09769	FGR proto-oncogene, Src family tyrosine kinase	Kinase	56	0.647	0.615	0.48408	0.01
early pregnancy	C0747845	VEGFD	2277	O43915	vascular endothelial growth factor D	Signaling	132	0.538	0.769	2.5E-05	0.01
early pregnancy	C0747845	FKBP4	2288	Q02790	FKBP prolyl isomerase 4	Enzyme	149	0.532	0.769	0.15539	0.01
early pregnancy	C0747845	DKK1	22943	O94907	dickkopf WNT signaling pathway inhibitor 1		372	0.439	0.885	0.17423	0.01
early pregnancy	C0747845	PALLD	23022	Q8WX93	palladin, cytoskeletal associated protein		69	0.628	0.615	5.25E-06	0.01
early pregnancy	C0747845	FOXO3	2309	O43524	forkhead box O3		381	0.431	0.808	0.98806	0.01
early pregnancy	C0747845	CRTC1	23373	Q6UUV9	CREB regulated transcription coactivator 1	Transcription factor	115	0.565	0.692	0.531	0.01

early pregnanc y	C0747 845	LY96	2364 3	Q9Y6Y9	lymphocyte antigen 96	Receptor	54	0.6 36	0.6 15	7.42 E-05	0.01
early pregnanc y	C0747 845	FUT4	2526	P22083	fucosyltransferase 4	Enzyme	168	0.5 05	0.7 31	7.73 E-06	0.01
early pregnanc y	C0747 845	MTHFD 1L	2590 2	Q6UB35	methylenetetrahydrofolate dehydrogenase (NADP+ dependent) 1 like	Enzyme	41	0.6 74	0.5 38	1.94 E-12	0.01
early pregnanc y	C0747 845	GH1	2688	P01241	growth hormone 1	Signaling	686	0.3 73	0.9 23	0.03 0057	0.01
early pregnanc y	C0747 845	GHRH	2691	P01286	growth hormone releasing hormone	Signaling	176	0.5 03	0.7 69	0.14 964	0.01
early pregnanc y	C0747 845	ACAD8	2703 4	Q9UKU7	acyl-CoA dehydrogenase family member 8	Enzyme	203	0.5 06	0.8 85	5.64 E-14	0.01
early pregnanc y	C0747 845	PALD1	2714 3	Q9ULE6	phosphatase domain containing paladin 1		33	0.6 86	0.4 23	3.07 E-15	0.01
early pregnanc y	C0747 845	PDCD4	2725 0	Q53EL6	programmed cell death 4	Nucleic acid binding	198	0.4 94	0.7 69	3.36 E-07	0.01
early pregnanc y	C0747 845	CSDC2	2725 4	Q9Y534	cold shock domain containing C2	Nucleic acid binding	4	0.9 31	0.1 54	0.00 3122	0.01
early pregnanc y	C0747 845	GNB3	2784	P16520	G protein subunit beta 3	Enzyme	192	0.5 18	0.8 08	3.33 E-11	0.01
early pregnanc y	C0747 845	ANGPT 1	284	Q15389	angiopoietin 1	Signaling	340	0.4 46	0.8 08	0.95 208	0.01

early pregnancy	C0747845	ANGPT2	285	O15123	angiopoietin 2	Signaling	457	0.41	0.846	0.83208	0.01
early pregnancy	C0747845	LGALS13	29124	Q9UHV8	galectin 13	Signaling	25	0.716	0.462	0.001127	0.01
early pregnancy	C0747845	GSTM1	2944	P09488	glutathione S-transferase mu 1		627	0.38	0.923	0.002064	0.01
early pregnancy	C0747845	GSTT1	2952	P30711	glutathione S-transferase theta 1		541	0.393	0.923	0.000148	0.01
early pregnancy	C0747845	PARVB	29780	Q9HBI1	parvin beta	Cellular structure	25	0.736	0.385	2.38E-13	0.01
early pregnancy	C0747845	ANXA1	301	P04083	annexin A1		336	0.442	0.885	1.2E-07	0.01
early pregnancy	C0747845	HDAC2	3066	Q92769	histone deacetylase 2	Epigenetic regulator	257	0.476	0.846	0.99997	0.01
early pregnancy	C0747845	ANXA5	308	P08758	annexin A5		283	0.458	0.769	3.8E-15	0.01
early pregnancy	C0747845	ERVW-1	30816	Q9UQF0	endogenous retrovirus group W member 1, envelope		193	0.505	0.692		0.01
early pregnancy	C0747845	HIF1A	3091	Q16665	hypoxia inducible factor 1 subunit alpha	Transcription factor	1044	0.327	0.923	0.9777	0.01
early pregnancy	C0747845	HLA-F	3134	P30511	major histocompatibility complex, class I, F		51	0.631	0.654	3.28E-05	0.01

early pregnancy	C0747845	NR4A1	3164	P22736	nuclear receptor subfamily 4 group A member 1	Nuclear receptor	216	0.49	0.731	0.37074	0.01
early pregnancy	C0747845	HSD3B1	3283	P14060	hydroxy-delta-5-steroid dehydrogenase, 3 beta- and steroid delta-isomerase 1	Enzyme	60	0.619	0.462	2.4E-07	0.01
early pregnancy	C0747845	HSD11B1	3290	P28845	hydroxysteroid 11-beta dehydrogenase 1		163	0.52	0.731	0.41696	0.01
early pregnancy	C0747845	HTC2	3342		hypertrichosis 2 (generalized, congenital)		511	0.392	0.808		0.01
early pregnancy	C0747845	HSD17B13	345275	Q7Z5P4	hydroxysteroid 17-beta dehydrogenase 13		58	0.623	0.538	2.74E-05	0.01
early pregnancy	C0747845	IFNGR1	3459	P15260	interferon gamma receptor 1	Receptor	153	0.529	0.692	0.015503	0.01
early pregnancy	C0747845	IFNGR2	3460	P38484	interferon gamma receptor 2	Receptor	71	0.597	0.577	0.95281	0.01
early pregnancy	C0747845	IL4R	3566	P24394	interleukin 4 receptor	Receptor	242	0.474	0.846	0.00023	0.01
early pregnancy	C0747845	AQP4	361	P55087	aquaporin 4	Ion channel	311	0.456	0.885	0.002292	0.01
early pregnancy	C0747845	ILK	3611	Q13418	integrin linked kinase	Kinase	192	0.503	0.769	3.79E-05	0.01
early pregnancy	C0747845	KDR	3791	P35968	kinase insert domain receptor	Kinase	623	0.378	0.885	0.99982	0.01

early pregnancy	C0747845	LIN28B	389421	Q6ZN17	lin-28 homolog B		126	0.556	0.654	0.55513	0.01
early pregnancy	C0747845	LASP1	3927	Q14847	LIM and SH3 protein 1		85	0.572	0.615	0.9827	0.01
early pregnancy	C0747845	LGALS3	3958	P17931	galectin 3	Signaling	557	0.392	0.846	2.32E-05	0.01
early pregnancy	C0747845	FADS3	3995	Q9Y5Q0	fatty acid desaturase 3		13	0.821	0.308	0.002533	0.01
early pregnancy	C0747845	MIR145	406937		microRNA 145		366	0.431	0.846		0.01
early pregnancy	C0747845	MIR210	406992		microRNA 210		277	0.46	0.846		0.01
early pregnancy	C0747845	MIR96	407053		microRNA 96		134	0.536	0.769		0.01
early pregnancy	C0747845	MDM2	4193	Q00987	MDM2 proto-oncogene	Nucleic acid binding	702	0.362	0.846	0.99981	0.01
early pregnancy	C0747845	MMP2	4313	P08253	matrix metallopeptidase 2	Enzyme	1021	0.333	0.923	0.83878	0.01
early pregnancy	C0747845	MMP3	4314	P08254	matrix metallopeptidase 3	Enzyme	473	0.408	0.885	5.74E-15	0.01
early pregnancy	C0747845	MMP7	4316	P09237	matrix metallopeptidase 7	Enzyme	320	0.446	0.885	1.04E-09	0.01

early pregnancy	C0747845	MMP10	4319	P09238	matrix metallopeptidase 10	Enzyme	123	0.553	0.808	5.91E-15	0.01
early pregnancy	C0747845	MMP13	4322	P45452	matrix metallopeptidase 13	Enzyme	328	0.452	0.923	7.88E-18	0.01
early pregnancy	C0747845	MMP15	4324	P51511	matrix metallopeptidase 15	Enzyme	54	0.644	0.5	0.073354	0.01
early pregnancy	C0747845	COX2	4513	P00403	cytochrome c oxidase subunit II	Enzyme	875	0.352	0.962		0.01
early pregnancy	C0747845	MTR	4548	Q99707	5-methyltetrahydrofolate-homocysteine methyltransferase		245	0.482	0.808	2.43E-12	0.01
early pregnancy	C0747845	MTRR	4552	Q9UBK8	5-methyltetrahydrofolate-homocysteine methyltransferase reductase		207	0.502	0.808	2.18E-14	0.01
early pregnancy	C0747845	NCAM1	4684	P13591	neural cell adhesion molecule 1		445	0.415	0.885	0.99999	0.01
early pregnancy	C0747845	NME1	4830	P15531	NME/NM23 nucleoside diphosphate kinase 1	Kinase	329	0.443	0.808	0.003176	0.01
early pregnancy	C0747845	NTRK2	4915	Q16620	neurotrophic receptor tyrosine kinase 2	Kinase	284	0.479	0.808	1	0.01
early pregnancy	C0747845	ODC1	4953	P11926	ornithine decarboxylase 1	Enzyme	184	0.51	0.731	0.16966	0.01
early pregnancy	C0747845	OSM	5008	P13725	oncostatin M	Signaling	188	0.505	0.769	0.58916	0.01

early pregnancy	C0747845	OTC	5009	P00480	ornithine carbamoyltransferase		135	0.565	0.846	0.87295	0.01
early pregnancy	C0747845	OVGP1	5016	Q12889	oviductal glycoprotein 1		21	0.729	0.538	0.000673	0.01
early pregnancy	C0747845	PAEP	5047	P09466	progestagen associated endometrial protein	Enzyme	397	0.43	0.846	3.11E-08	0.01
early pregnancy	C0747845	PRDX1	5052	Q06830	peroxiredoxin 1	Enzyme	237	0.485	0.846	7.7E-10	0.01
early pregnancy	C0747845	SERPINE1	5054	P05121	serpin family E member 1	Enzyme modulator	770	0.359	0.885	0.043609	0.01
early pregnancy	C0747845	ADIPOR1	51094	Q96A54	adiponectin receptor 1	Receptor	148	0.529	0.808	0.6584	0.01
early pregnancy	C0747845	PCSK5	5125	Q92824	proprotein convertase subtilisin/kexin type 5	Enzyme	57	0.659	0.538	2.54E-12	0.01
early pregnancy	C0747845	PDCD1	5133	Q15116	programmed cell death 1		497	0.402	0.846	0.41706	0.01
early pregnancy	C0747845	HSD17B7	51478	P56937	hydroxysteroid 17-beta dehydrogenase 7		81	0.582	0.5	6.65E-06	0.01
early pregnancy	C0747845	TPPP3	51673	Q9BW30	tubulin polymerization promoting protein family member 3	Cellular structure	22	0.736	0.385	0.37331	0.01
early pregnancy	C0747845	PECAM1	5175	P16284	platelet and endothelial cell adhesion molecule 1	Cell adhesion	396	0.426	0.846		0.01

early pregnancy	C0747845	CFP	5199	P27918	complement factor properdin		108	0.573	0.692	0.68778	0.01
early pregnancy	C0747845	PFKFB3	5209	Q16875	6-phosphofructo-2-kinase/fructose-2,6-biphosphatase 3	Kinase	104	0.561	0.808	0.24579	0.01
early pregnancy	C0747845	ABCB1	5243	P08183	ATP binding cassette subfamily B member 1	Transporter	933	0.344	0.885	1.26E-05	0.01
early pregnancy	C0747845	SERPINB5	5268	P36952	serpin family B member 5	Enzyme modulator	87	0.582	0.577	0.003649	0.01
early pregnancy	C0747845	PIGF	5281	Q07326	phosphatidylinositol glycan anchor biosynthesis class F	Enzyme	26	0.716	0.385	0.00072	0.01
early pregnancy	C0747845	PIK3CA	5290	P42336	phosphatidylinositol-4,5-bisphosphate 3-kinase catalytic subunit alpha	Kinase	1511	0.292	0.923	1	0.01
early pregnancy	C0747845	PIK3CB	5291	P42338	phosphatidylinositol-4,5-bisphosphate 3-kinase catalytic subunit beta	Kinase	1083	0.322	0.885	0.99964	0.01
early pregnancy	C0747845	PIK3CD	5293	O00329	phosphatidylinositol-4,5-bisphosphate 3-kinase catalytic subunit delta	Kinase	1119	0.319	0.885	0.99999	0.01
early pregnancy	C0747845	PIK3CG	5294	P48736	phosphatidylinositol-4,5-bisphosphate 3-kinase catalytic subunit gamma	Kinase	1101	0.32	0.885	1.41E-06	0.01
early pregnancy	C0747845	PLXNB1	5364	O43157	plexin B1		65	0.621	0.692	0.000383	0.01
early pregnancy	C0747845	PML	5371	P29590	promyelocytic leukemia		274	0.477	0.846	0.21291	0.01

early pregnancy	C0747845	POMC	5443	P01189	proopiomelanocortin		873	0.356	0.846	0.000117	0.01
early pregnancy	C0747845	PPARA	5465	Q07869	peroxisome proliferator activated receptor alpha	Nuclear receptor	408	0.432	0.885	0.030182	0.01
early pregnancy	C0747845	ANO1	55107	Q5XXA6	anoctamin 1	Ion channel	175	0.516	0.769	0.87426	0.01
early pregnancy	C0747845	EXOC2	55770	Q96KP1	exocyst complex component 2		24	0.751	0.423	2.87E-09	0.01
early pregnancy	C0747845	PAG1	55824	Q9NWQ8	phosphoprotein membrane anchor with glycosphingolipid microdomains 1		174	0.505	0.808	0.017764	0.01
early pregnancy	C0747845	ANGPTL8	55908	Q6UXH0	angiopoietin like 8		54	0.65	0.577	6.88E-12	0.01
early pregnancy	C0747845	PRKG2	5593	Q13237	protein kinase cGMP-dependent 2	Kinase	31	0.705	0.308	0.64685	0.01
early pregnancy	C0747845	MYDGF	56005	Q969H8	myeloid derived growth factor		295	0.451	0.846	3.49E-06	0.01
early pregnancy	C0747845	LGALS14	56891	Q8TCE9	galectin 14	Signaling	35	0.695	0.423	0.000275	0.01
early pregnancy	C0747845	ACKR3	57007	P25106	atypical chemokine receptor 3	G-protein coupled receptor	400	0.428	0.885	0.39134	0.01
early pregnancy	C0747845	PTGIR	5739	P43119	prostaglandin I2 receptor	G-protein coupled receptor	27	0.711	0.423	0.000888	0.01

early pregnanc y	C0747 845	PTGIS	5740	Q16647	prostaglandin I2 synthase		70	0.6 17	0.6 54	1.2E- 09	0.01
early pregnanc y	C0747 845	PTGS2	5743	P35354	prostaglandin-endoperoxide synthase 2	Enzyme	1234	0.3 14	0.9 62	0.99 597	0.01
early pregnanc y	C0747 845	MIR499 A	5745 01		microRNA 499a		156	0.5 19	0.8 46		0.01
early pregnanc y	C0747 845	SCAF1	5850 6	Q9H7N4	SR-related CTD associated factor 1		43	0.6 66	0.5	0.99 819	0.01
early pregnanc y	C0747 845	BCL6	604	P41182	BCL6 transcription repressor		309	0.4 44	0.8 08	0.96 038	0.01
early pregnanc y	C0747 845	PAPPA 2	6067 6	Q9BXP8	pappalysin 2		28	0.7 51	0.4 23	7.79 E-08	0.01
early pregnanc y	C0747 845	S100A8	6279	P05109	S100 calcium binding protein A8	Calcium-binding protein	369	0.4 3	0.9 62	0.13 761	0.01
early pregnanc y	C0747 845	S100A9	6280	P06702	S100 calcium binding protein A9	Calcium-binding protein	363	0.4 33	0.8 85	0.47 77	0.01
early pregnanc y	C0747 845	CCL4	6351	P13236;Q 8NHW4	C-C motif chemokine ligand 4	Signaling	276	0.4 66	0.9 23	0.24 134	0.01
early pregnanc y	C0747 845	CCL5	6352	P13501	C-C motif chemokine ligand 5	Signaling	514	0.4 03	0.8 85	0.00 1752	0.01
early pregnanc y	C0747 845	CCL14	6358	Q16627	C-C motif chemokine ligand 14	Signaling	29	0.6 95	0.4 23	0.00 0109	0.01

early pregnancy	C0747845	CCL20	6364	P78556	C-C motif chemokine ligand 20	Signaling	242	0.474	0.846	0.000242	0.01
early pregnancy	C0747845	CCL21	6366	O00585	C-C motif chemokine ligand 21	Signaling	186	0.502	0.769	0.036239	0.01
early pregnancy	C0747845	CCL23	6368	P55773	C-C motif chemokine ligand 23	Signaling	18	0.76	0.346	0.000597	0.01
early pregnancy	C0747845	CXCL6	6372	P80162	C-X-C motif chemokine ligand 6	Signaling	90	0.582	0.692	2.71E-09	0.01
early pregnancy	C0747845	SDC4	6385	P31431	syndecan 4	Extracellular structure	71	0.603	0.615	0.004148	0.01
early pregnancy	C0747845	SELENOP	6414	P49908	selenoprotein P	Extracellular structure	138	0.545	0.769	8.29E-07	0.01
early pregnancy	C0747845	SGK1	6446	O00141	serum/glucocorticoid regulated kinase 1	Kinase	188	0.506	0.769	0.002305	0.01
early pregnancy	C0747845	PRSS56	646960	P0CW18	serine protease 56		19	0.743	0.115	0.000341	0.01
early pregnancy	C0747845	BMI1	648	P35226	BMI1 proto-oncogene, polycomb ring finger		309	0.448	0.808	0.94286	0.01
early pregnancy	C0747845	IL25	64806	Q9H293	interleukin 25		139	0.529	0.846	0.000923	0.01
early pregnancy	C0747845	NDRG4	65009	Q9ULP0	NDRG family member 4	Enzyme	36	0.67	0.462	0.011627	0.01

early pregnanc y	C0747 845	SLC2A1	6513	P11166	solute carrier family 2 member 1	Transporter	687	0.3 88	0.8 08	0.99 41	0.01
early pregnanc y	C0747 845	UCA1	6529 95		urothelial cancer associated 1		152	0.5 12	0.8 08		0.01
early pregnanc y	C0747 845	BMPR1 A	657	P36894	bone morphogenetic protein receptor type 1A	Kinase	203	0.5 22	0.7 31	0.90 333	0.01
early pregnanc y	C0747 845	BMPR1 B	658	O00238	bone morphogenetic protein receptor type 1B	Kinase	145	0.5 73	0.6 15	0.99 863	0.01
early pregnanc y	C0747 845	BMPR2	659	Q13873	bone morphogenetic protein receptor type 2	Kinase	146	0.5 38	0.6 54	0.99 998	0.01
early pregnanc y	C0747 845	DST	667	Q03001	dystonin	Cellular structure	147	0.5 52	0.8 46	1	0.01
early pregnanc y	C0747 845	TRBV20 OR9-2	6962		T cell receptor beta variable 20/OR9-2 (non-functional)		456	0.4 03	0.9 23		0.01
early pregnanc y	C0747 845	TDO2	6999	P48775	tryptophan 2,3-dioxygenase	Enzyme	89	0.5 99	0.6 54	2.04 E-10	0.01
early pregnanc y	C0747 845	PRDX2	7001	P32119	peroxiredoxin 2	Enzyme	243	0.4 74	0.8 85	0.08 6513	0.01
early pregnanc y	C0747 845	TG	7038	P01266	thyroglobulin	Enzyme modulator	240	0.4 8	0.8 08	2.88 E-59	0.01
early pregnanc y	C0747 845	TGFB1	7040	P01137	transforming growth factor beta 1	Signaling	1558	0.2 87	0.9 62	0.03 685	0.01

early pregnancy	C0747845	THBS1	7057	P07996	thrombospondin 1		480	0.407	0.885	0.99985	0.01
early pregnancy	C0747845	TIMP1	7076	P01033	TIMP metallopeptidase inhibitor 1	Enzyme modulator	603	0.38	0.885	0.4993	0.01
early pregnancy	C0747845	TPO	7173	P07202	thyroid peroxidase	Enzyme	306	0.455	0.846	1.82E-21	0.01
early pregnancy	C0747845	TRC-GCA24-1	7183		tRNA-Cys (GCA) 24-1		21	0.729	0.346		0.01
early pregnancy	C0747845	TTN	7273	Q8WZ42	titin	Kinase	366	0.47	0.885	2.56E-96	0.01
early pregnancy	C0747845	WNT5A	7474	P41221	Wnt family member 5A	Signaling	375	0.443	0.808	0.98732	0.01
early pregnancy	C0747845	PXDN	7837	Q92626	peroxidasin	Enzyme	51	0.647	0.5	2.51E-07	0.01
early pregnancy	C0747845	USP7	7874	Q93009	ubiquitin specific peptidase 7	Enzyme	153	0.538	0.808	1	0.01
early pregnancy	C0747845	FTO	79068	Q9C0B1	FTO alpha-ketoglutarate dependent dioxygenase		286	0.486	0.885	8.36E-08	0.01
early pregnancy	C0747845	DEK	7913	P35659	DEK proto-oncogene		122	0.542	0.769	0.13065	0.01
early pregnancy	C0747845	DHRS11	79154	Q6UWP2	dehydrogenase/reductase 11	Enzyme	43	0.653	0.423	2.24E-06	0.01

early pregnancy	C0747845	MMEL1	79258	Q495T6	membrane metalloendopeptidase like 1	Enzyme	74	0.606	0.692	1.24E-22	0.01
early pregnancy	C0747845	ADIPOR2	79602	Q86V24	adiponectin receptor 2	Receptor	103	0.564	0.731	0.36218	0.01
early pregnancy	C0747845	FXR1	8087	P51114	FMR1 autosomal homolog 1	Nucleic acid binding	53	0.656	0.615	1	0.01
early pregnancy	C0747845	CALR	811	P27797	calreticulin	Calcium-binding protein	487	0.413	0.923	0.89133	0.01
early pregnancy	C0747845	H3-4	8290	Q16695	H3.4 histone		26	0.711	0.538	1.65E-05	0.01
early pregnancy	C0747845	CCM2	83605	Q9BSQ5	CCM2 scaffold protein		50	0.636	0.385	0.009281	0.01
early pregnancy	C0747845	SPARCL1	8404	Q14515	SPARC like 1	Signaling	69	0.604	0.615	5.64E-10	0.01
early pregnancy	C0747845	KISS1R	84634	Q969F8	KISS1 receptor	G-protein coupled receptor	195	0.515	0.769	0.43649	0.01
early pregnancy	C0747845	PROM1	8842	O43490	prominin 1	Transporter	477	0.41	0.846	1.95E-22	0.01
early pregnancy	C0747845	CCNA2	890	P20248	cyclin A2	Enzyme modulator	90	0.578	0.654	0.99962	0.01
early pregnancy	C0747845	NAT1	9	P18440	N-acetyltransferase 1	Enzyme	133	0.536	0.846	1.93E-14	0.01

early pregnancy	C0747845	WDR20	91833	Q8TBZ3	WD repeat domain 20		77	0.595	0.769	0.91532	0.01
early pregnancy	C0747845	DEDD	9191	O75618	death effector domain containing		24	0.711	0.346	0.95912	0.01
early pregnancy	C0747845	SLIT2	9353	O94813	slit guidance ligand 2		177	0.511	0.769	1	0.01
early pregnancy	C0747845	CGB5	93659	P0DN86	chorionic gonadotropin subunit beta 5		83	0.579	0.654	0.025362	0.01
early pregnancy	C0747845	MUC16	94025	Q8WXI7	mucin 16, cell surface associated		236	0.479	0.808	8.3E-112	0.01
early pregnancy	C0747845	HTRA3	94031	P83110	HtrA serine peptidase 3	Enzyme	42	0.674	0.5	6.58E-08	0.01
early pregnancy	C0747845	CD80	941	P33681	CD80 molecule		233	0.475	0.769	0.010898	0.01
early pregnancy	C0747845	CGB8	94115	P0DN86	chorionic gonadotropin subunit beta 8		79	0.585	0.654	0.015956	0.01
early pregnancy	C0747845	CD86	942	P42081	CD86 molecule		219	0.483	0.769	0.94879	0.01
early pregnancy	C0747845	MDC1	9656	Q14676	mediator of DNA damage checkpoint 1		56	0.628	0.692	2.27E-08	0.01
early pregnancy	C0747845	PCLAF	9768	Q15004	PCNA clamp associated factor		125	0.538	0.769	0.16276	0.01

Familial obesity	C1281440	TBC1D1	23216	Q86TI0	TBC1 domain family member 1	Enzyme	25	0.716	0.423	1.86E-13	0.03
Familial obesity	C1281440	SLCO4C1	353189	Q6ZQN7	solute carrier organic anion transporter family member 4C1		11	0.805	0.231	1.06E-08	0.01
Familial obesity	C1281440	MC4R	4160	P32245	melanocortin 4 receptor	G-protein coupled receptor	149	0.535	0.692	0.000917	0.01
Familial obesity	C1281440	SLC2A1	6513	P11166	solute carrier family 2 member 1	Transporter	687	0.388	0.808	0.9941	0.01
